# A cross-national analysis of demographic variation in daily smoking across 22 countries

**DOI:** 10.1038/s41598-024-76318-9

**Published:** 2025-04-30

**Authors:** Sung Joon Jang, Pedro A. de la Rosa, R. Noah Padgett, Matt Bradshaw, Tyler J. VanderWeele, Byron R. Johnson

**Affiliations:** 1 Baylor University, Waco, USA; 2 Pepperdine University, Malibu, USA; 3 University of Navarra, Pamplona, Spain; 4 Harvard University, Cambridge, USA; 5 Harvard T.H. Chan School of Public Health, Boston, USA

**Keywords:** Cigarette smoking, Global flourishing study, Cross-national, Random effects meta-analysis, Risk factors, Human behaviour

## Abstract

Cigarette use, a leading cause of preventable disease and millions of deaths worldwide each year, is not often studied separately from other tobacco use, and global data and previous studies on cigarette smoking are based mostly on its prevalence rather than quantity. To address these oversights, we analyze the first wave of data from Global Flourishing Study, a study of over 200,000 adults nationally representative of 22 countries. We measured cigarette smoking by daily consumption per capita (mean) and per individual who smoked (intensity) as well as prevalence. The mean of daily smoking was positively correlated with the prevalence, whereas the intensity was not significantly related to the prevalence. Similarly, we found that random effects meta-analysis results of country-specific means of daily smoking in each category of demographic variables (age, gender, marital status, employment, religious service attendance, education, and immigration status) were more consistent with the results of prevalence than was the case with intensity, though there was variation across countries. Overall findings indicate the importance of studying the intensity as well as prevalence of smoking, as they are distinct and thus both required in order to assess tobacco-associated health risks and establish informed tobacco control policies.

**Supplementary Information:**

The online version contains supplementary material available at 10.1038/s41598-024-76318-9.

## Introduction

According to the World Health Organization (WHO), 20.9% of all persons 15 years of age or older (estimated to be 1.245 billion) used tobacco in 2022^2^, and 80.0% of them smoked tobacco with 89.4% of those who smoked tobacco using cigarettes (estimated to be 890 million). That is, cigarette smoking is the most common form of tobacco use worldwide and a leading cause of preventable disease. In fact, more than 8 million deaths (including an estimated 1.3 million deaths by non-smokers exposed to second-hand smoke) caused by tobacco use each year, are attributed mostly to smoking cigarettes^1^. As a result, cigarettes have received more attention from researchers than any other tobacco products, but studies have tended to focus on prevalence of lifetime or “current” use of cigarettes, failing to isolate the more harmful form of cigarette use, daily smoking^2–4^. Also, global research on the intensity of daily smoking (i.e., daily cigarette consumption per individual who smokes cigarettes) is scarce despite its deleterious impact on health, as well as the fact that intensity is a measure of cigarette use among individuals who smoke and thus not necessarily expected to be positively correlated with the population-based measure, prevalence^4^. Moreover, unlike country-specific studies, cross-national research on demographic variations in cigarette smoking has been confined to only a few variables—such as age, gender, and, to a lesser extent, income^4–7^—although examining other variables (e.g., marital status, employment, education, religion, and immigration status) could contribute to formulating global health policies.

To address these gaps in global research on cigarette smoking, we analyzed data from the first wave of Global Flourishing Study (GFS), a panel study of over 200,000 adults sampled to be representative of 22 countries (including a territory, Hong Kong). Data for Wave 1 were collected principally in 2023. Before presenting our results, we briefly review previous studies on demographic variations in smoking, followed by a preliminary examination of a relationship between prevalence and intensity of smoking in those 22 countries based on existing global data.

### Prior research on demographic variations in smoking

Due to data limitations, previous country-specific and cross-national studies were only able to examine demographic variations in the prevalence of smoking.

The 2024 WHO report on the prevalence of tobacco use shows that the age group 45–54 had the highest rates across the years 2000–2022, with the most recent rate being 26.4% in 2022^2^ when the next two highest rates were of age groups, 55–64 (24.8%) and 35–44 (24.7%), followed by their adjacent groups, 65–74 (21.1%) and 25–34 (20.4%), and the next two age groups, 75–84 (16.9%) and 15–24 (13.3%), with the age group 85 or older (12.9%) showing the lowest rate. This curvilinear relationship—attributable in part to age differences in health-risk perception and psychological addiction^8,9^ as well as financial ability to support smoking habits—was found over the past two decades. Previous studies show variations across countries, but they cannot be compared due to differences in age categorization, type of tobacco use, and sampling^4,10–14^.

Tobacco use is predominantly a male behavior worldwide due to traditional sex roles (e.g., social disapproval of women’s smoking) and gender socialization (e.g., less rebellious, risk-taking tendency among girls than boys) rather than gender differences in nicotine metabolism or health-related concerns^15,16^. The most recent WHO report shows that the global prevalence of current cigarette smoking has been higher among males than females for the past 23 years including 2022 (25.5% vs. 4.4%), while the difference tends to be greater in developing countries rather than developed countries^2^.

In the U.S., “divorced/separated or widowed” (16.8%) individuals were found to have higher prevalence of cigarette use than those “married or living with partner” (10.4%) with the “single/never married or not living with partner” (10.9%) category being in-between^11^. When the non-married categories were examined separately, the widowed had lower prevalence than not only the “divorced/separated” but also those married or living with partner”^17^. Similar patterns were observed in other countries^10,12,18^. The lower rates of smoking among the married compared to the non-married (except the widowed) are explained partly by the social capital-related protective effects of marriage in terms of social support, economic resources, and stress reduction^10,12^.

Using English survey data, Hussain et al.^19^ found that unemployed individuals were less likely to currently smoke cigarettes than their employed peers, while controlling for other demographic variables. Similarly, in a multivariate analysis Fukuda et al.^13^ found that the employed reported a higher prevalence of current smoking than the unemployed among Japanese adults. While the lower rate among the unemployed may be due to limited financial resources to support a smoking habit, Cornelius et al.^11^ found income to be inversely related to smoking prevalence in the U.S. They also found an inverse relationship between education and cigarette smoking: specifically, prevalence of current smoking tended to decline as we moved from the lowest to the highest level of education: “0–12 years, no diploma” (20.1%); “GED” (30.7%); “high school diploma” (17.1%); “some college, no degree” (16.1%); “associate degree, academic, technical, or vocational” (13.7%); “bachelor’s degree” (5.3%); and “graduate degree, master’s doctoral, or professional” (3.2%)^11^. An inverse relationship was also found in England and Romania^19,20^. Education is inversely related to smoking partly because it is positively related to perceived health-risk of smoking and investment in health^21^.

Although most major religions have no specific precepts against smoking, they do tend to discourage health-risk behaviors by teaching that the physical body is a sacred gift from God or a deity^22^. As a result, people reporting a religious affiliation are less likely to smoke compared to those without any religion^19,23–25^. However, since not all of those with a religion do not smoke^23^, it is better to focus on actual religious involvement than mere affiliation. According to the most updated review, over 85% of longitudinal studies reported that greater religiosity predicted a lower likelihood of cigarette smoking, consistent with most cross-sectional studies to date^22^. Religious service attendance has been found to be more consistently related inversely to smoking than other indicators of religiosity, such as private practices or religious belief^22^.

Prior research shows, while immigrants are less likely to smoke than their native-born counterparts, the difference depends on other factors, such as the country of birth, sex, and duration of residence^26–29^. Specifically, the degree of low prevalence of smoking among foreign-born compared to native-born individuals was found to be greater among (1) immigrants from economically developing non-western countries than those from economically developed western countries and (2) first than later generations of immigrants. Also, more acculturated women were likely to smoke relative to their less acculturated counterparts, whereas the opposite was observed among men.

### Existing data on prevalence and intensity of smoking in GFS countries

Table 1 presents the 2022 estimates of age-standardized and crude-adjusted prevalence of “current cigarette smoking” (i.e., smoking on a daily or non-daily basis) from the most recent WHO report^2^, as well as rarely available intensity estimates of daily cigarette smoking for 2019 from the *OECD Health Statistics 2023* (https://stats.oecd.org/) and 2012 from Ng et al.’s study^4^. Table 1 also includes the prevalence of daily smoking that the WHO reported for 2010 (tobacco smoking) and 2016 (cigarette smoking), which were relatively close to the year of Ng et al.’s estimates^6,30^.

**Table 1:** Global data on the prevalence and intensity (daily cigarette consumption per individual who smoked) of current/daily cigarette/tobacco smoking among persons 15 or older.

Country (WHO region)	WHO, 2022		OECD, 2019		Ng et al., 2012		WHO, 2010	WHO, 2016	
	Age-standardized prevalence (ASP) of current cigarette smoking ^a^	Crude adjusted prevalence (CAP) of current cigarette smoking	% of people who daily smoke cigarettes	Mean daily cigarette consumption per individual who smoked			CAP of daily tobacco smoking	ASP of daily cigarette smoking	CAP of daily cigarette smoking
Kenya (1)	7.2	6.5	-	-	12.0		9.7	7.0	6.9
Nigeria (1)	2.5	2.4	-	-	14.7		6.5	3.0	3.3
S. Africa (1)	16.8	16.5	20.2	-	11.8		16.5	14.0	13.4
Tanzania (1)	5.9	5.0	-	-	7.8		12.0	9.0	8.8
Argentina (2)	21.7	21.1	24.3	-	19.7		19.5	15.0	14.4
Brazil (2)	10.9	11.0	9.8	-	22.8		13.9	10.0	10.0
Mexico (2)	13.7	13.4	-	-	11.8		9.5	8.0	7.7
United States (2)	16.4	15.5	10.9	13.7	22.5		-	14.0	13.2
Egypt (3)	19.3	19.2	-	-	19.1		18.3	16.0	16.2
Germany (4)	19.1	16.9	-	-	15.1		21.9	23.0	20.2
Israel (4)	18.2	17.7	16.4	15.7	18.9		-	19.0	18.8
Poland (4)	22.0	21.6	17.1	15.2	17.4		24.5	22.0	21.3
Spain (4)	26.6	23.3	-	-	20.0		24.9	22.0	19.7
Sweden (4)	9.2	9.0	10.4	-	22.5		12.6	9.0	8.6
Türkiye (4)	28.9	29.1	28.0	17.1	19.4		24.8	22.0	21.7
United Kingdom (4)	12.3	11.4	15.8	10.0	13.6		-	17.0	15.8
India (5)	4.6	4.6	8.6	-	8.2		11.6	3.0	2.9
Indonesia (5)	33.2	33.4	32.6	-	11.0		30.9	32.0	32.0
Australia (6)	10.6	10.1	11.2	12.9	20.1		14.7	10.0	9.7
China (6)	23.3	24.8	25.4	-	22.3		23.5	20.0	20.8
Japan (6)	18.5	16.2	16.7	15.5	23.8		-	18.0	16.0
Philippines (6)	18.9	19.0	-	-	21.4		21.6	17.0	16.7

WHO = World Health Organization; OECD = Organization for Economic Cooperation and Development, Ng et al. = Ng. et al. (2014); (1) = African Region, (2) = Region of Americas, (3) = Eastern Mediterranean Region, (4) = European Region, (5) = South-East Asia Region, (6) = Western Pacific Region.

^a^ Refers to smoking cigarettes at the time of survey on a daily or non-daily basis.

Sources: World Health Organization. *WHO global report on trends in prevalence of tobacco use 2000–2030*. (2024); *OECD Health Statistics 2023* (https://stats.oecd.org/*);* World Health Organization. *WHO global report on trends in prevalence of tobacco smoking 2000–2025*, second Edition. (2018); World Health Organization. *WHO global report on trends in prevalence of tobacco smoking 2015*. (2015); Ng, M. et al. Smoking prevalence and cigarette consumption in 187 countries, 1980–2012. *JAMA* **311**, 183–192 (2014).

This existing dataset, compiled from multiple sources, allows us to preliminarily examine a relationship between the prevalence and intensity of smoking before presenting our findings about the relationship based on the GFS data. The same type of measures, whether prevalence or intensity, are expected to be positively correlated with its strength being determined by how different they are in the type of tobacco measured (all tobacco products vs. cigarette only), the frequency of smoking (daily vs. non-daily), and the year of data collection. Different measures are not necessarily; and, if they are, likely to be correlated not as strongly as the same measures because they use different bases for calculation: that is, prevalence is calculated based on a population, whereas intensity is based on a subpopulation of at-risk individuals (i.e., those who smoke and thus at risk of tobacco-associated diseases). So, the prevalence-intensity relationship is an empirical question: whether and how they are correlated. If intensity is found to be not correlated—at least, not strongly—with prevalence, it would indicate the importance of using intensity as well as prevalence as a measure of smoking because they are distinct measurements, and the intensity of daily consumption is a key predictor of tobacco-associated disease risk^7,31^.

Based on the estimated population statistics presented in Table 1, prevalence estimates across sources were highly correlated with each other despite differences in calculation, year, and source. Specifically, Spearman’s correlation between the WHO 2022 age-standardized prevalence rates of *current* cigarette smoking and the OECD 2019 rates of *daily* cigarette smoking was strongly positive (0.863, *p* < .001). When the age-standardized rate was replaced by crude-adjusted prevalence, the correlation remained high (0.880, *p* < .001). In contrast, the two sets of intensity estimates were not significantly correlated (0.283, *p* = .460), perhaps due in part to the difference in the year of data collection as well as the OECD estimates being available only for seven of the 22 countries in the GFS. More importantly, intensity and prevalence measures were not correlated, whether we used the OECD data (0.527, *p* = .117) or correlated the three WHO rates for 2010 and 2016 with Ng et al.’s 2012 estimates (0.266, 0.193, and 0.192; *p* = .285, 0.388, and 0.393).

However, since we do not know the extent to which those non-significant relationships are attributable to missing data and/or differences in the year of data collection, properly examining the prevalence-intensity relationship requires data on both measures of the same type of tobacco use, collected from multiple countries, which is possible with data collected in the GFS. This new global dataset also enables us to examine demographic variations in the intensity as well as prevalence of daily cigarette smoking across countries.

Specifically, we tested the following working hypotheses, focusing on daily cigarette consumption per capita (mean) and per individual who smoked (intensity) in comparison with prevalence of daily cigarette smoking:

#### Hypothesis 1

 The distributions and descriptive statistics of key demographic features (age, gender, marital status, employment, religious service attendance, education, immigration status) will reveal diverse patterns across our international sample from 22 countries.

#### Hypothesis 2

 The mean [and intensity, which was not pre-registered with the Center for Open Science] levels of daily smoking will vary meaningfully across different countries [in such a way that observed cross-country variations will be generally consistent with previous findings and/or amenable to *post hoc* interpretation of the observed variations].

#### Hypothesis 3

 Daily smoking will exhibit variations across different demographic categories such as age, gender, marital status, employment, religious service attendance, education, and immigration status. These differences across demographic categories will themselves vary by country.

## Methods

The following description of our methods has been adapted from VanderWeele et al.^32^. Further methodological details are available elsewhere^33–39^.

### Data

The Global Flourishing Study (GFS) is a longitudinal study of over 200,000 adults (age 18 or older) from 22 geographically and culturally diverse countries, with nationally representative sampling within each country, concerning the distribution of determinants of well-being. Data for Wave 1 were collected principally during 2023, while some countries began data collection in 2022^36^. Four additional waves of panel data on study participants will be collected annually from 2024 to 2027. A total of 202,898 individuals participated in Wave 1 survey in Argentina, Australia, Brazil, Egypt, Germany, Hong Kong, India, Indonesia, Israel, Japan, Kenya, Mexico, Nigeria, the Philippines, Poland, South Africa, Spain, Sweden, Tanzania, Türkiye, the United Kingdom, and the United States. These countries were selected to (1) maximize coverage of the world’s population, (2) ensure geographic, cultural, and religious diversity, and (3) prioritize feasibility and existing data collection infrastructure.

Data collection was carried out by Gallup, Inc., and the precise sampling design to ensure nationally representative samples varied by country^36,39^. While there are concerns with the sampling design varying by country, the issue boils down to how representative the samples are to the country’s population. To ensure the representativeness of each country’s sample, weights were constructed and post-stratified and adjusted for nonresponse to align the observed samples with the target populations. Survey items included various aspects of well-being such as happiness and life satisfaction, physical and mental health, meaning and purpose, character and virtue, close social relationships, and financial and material stability^40^, along with other demographic, social, economic, political, religious, personality, childhood, community, health, and well-being variables. The data are publicly available through the Center for Open Science (COS) (https://www.cos.io/gfs)^36^. During the translation process, Gallup adhered to TRAPD model (translation, review, adjudication, pretesting, and documentation) for cross-cultural survey research (https://ccsg.isr.umich.edu/chapters/translation/overview/).

### Measures

Daily smoking was measured by an item, asking “About how many cigarettes do you smoke each day, if any?” (0 = None/Do not smoke, 1 = one, 2 = two, … 97 = 97+). For primary analysis, we used the item as a continuous variable to assess not only *per capita daily smoking* but also *intensity* of smoking by examining the distribution of cigarette use for those reporting to smoke at least once per day (i.e., daily smokers). To supplement this primary analysis, we dichotomized the item (0 = None/Do not smoke, 1 = 1+) to evaluate *prevalence* of daily smoking.

Continuous age was classified as 18–24, 25–34, 35–44, 45–54, 55–64, 65–74, 75–84, and 85 or older, the same categories that WHO reports use except the minimum age being 18, instead of 15. Gender was assessed as male, female, and other. Marital status was assessed as single/never married, married, separated, divorced, widowed, and domestic partner. Employment was assessed as employed, self-employed, retired, student, homemaker, unemployed and searching, and other. Education was assessed as up to 8 years, 9–15 years, and 16 + years. Religious service attendance was assessed as more than once/week, once/week, one-to-three times/month, a few times/year, or never. Immigration status was dichotomously assessed as foreign- or native-born. Religious tradition/affiliation had categories of Christianity, Islam, Hinduism, Buddhism, Judaism, Sikhism, Baha’i, Jainism, Shinto, Taoism, Confucianism, Primal/Animist/Folk religion, Spiritism, African-Derived, some other religion, or no religion/atheist/agnostic with precise response categories varying by country^41^. Racial/ethnic identity was assessed in some, but not all, countries with response categories varying by country. For additional details on the assessments, see the GFS codebook (https://osf.io/7uj6y/) or Crabtree et al.^33^.

### Analysis

Descriptive statistics for the full sample, weighted to be nationally representative within each country, were estimated for each demographic variable. Two averages of daily cigarette consumption—one for the total sample (mean) and another for smokers only (intensity)—and proportion (prevalence) of daily cigarette smoking were estimated separately for each country along with 95% confidence intervals (CIs), standard deviations, and Gini coefficients (which measure the inequality among the values of daily cigarette smoking in a country’s population, ranging from 0 to 1 with 0 and 1 representing perfect equality and inequality, respectively) for the mean and intensity, and 95% CIs and standard errors for the prevalence. Variation in averages and proportions of daily cigarette smoking across demographic categories were estimated, with all analyses initially conducted by country (online supplement). Key results consisted of random effects meta-analyses of country-specific averages and proportions of daily cigarette smoking in each specific demographic category^42,43^ along with 95% CIs, standard errors, lower and upper limits of a 95% prediction interval across countries, heterogeneity (τ), and I^2^ for evidence concerning variation within a particular demographic variable across countries^44^. Forest plots of estimates are available in the online supplement. All meta-analyses were conducted in **R**^45^ using the *metafor* package^46^. Within each country, a global test of variation of daily smoking across levels of each demographic variable was conducted for both the total sample (mean and prevalence) and the smoker sample (intensity), and a pooled *p*-value^47^ across countries reported concerning evidence for variation within any country. Bonferroni-corrected *p*-value threshold is provided based on the number of demographic variables^48,49^. Religious tradition/affiliation and race/ethnicity were used, when available, as control variables within country, but were not included in the meta-analyses since the availability of these response categories varied by country. As a supplementary analysis, population weighted meta-analyses were also conducted. All analyses were pre-registered with the COS prior to data access (https://osf.io/fajrz/) except for the analysis of intensity, which followed the preregistered, same plan for the analysis of mean; all code needed to reproduce analyses are available in an online repository^35^.

### Missing data

Missing data on all variables were imputed using multivariate imputation by chained equations, and five imputed datasets were created and analyzed^50–53^. To account for variation in the assessment of certain variables across countries (e.g., religious tradition/affiliation and race/ethnicity), the imputation process was conducted separately in each country. This within-country imputation approach ensured that the imputation models accurately reflected country-specific contexts and assessment methods. Sampling weights were included in the imputation model to account for missingness to be related to probability of inclusion.

## Accounting for complex sampling design

The GFS used different sampling schemes across countries based on availability of existing panels and recruitment needs^36^. All analyses account for the complex survey design components by including weights, primary sampling units, and strata. For analyses involving the subpopulation of smokers (analyses involving intensity), corrected weights were used to properly conduct domain estimation. Additional methodological detail, including accounting for the complex sampling design is provided elsewhere^34,39^.

### Supplemental Analyses (Not Pre-Registered)

Relationships among the three measures of daily smoking (mean, intensity, and prevalence) were assessed based on Spearman’s rank-order correlation coefficient.

## Results

Table 2 reports frequency distributions of the demographic variables, first, for the total sample of all 22 countries combined (*N* = 202,898) and then a subsample including only respondents who smoked at least one cigarette daily (*N* = 38,290) and thus were at higher risk of smoking-related diseases compared to those who did not smoke daily including occasional smokers (henceforth, “at-risk subsample”). Country sample size varied from Türkiye’s 1,473 (0.7%) to 38,312 (19%) in the United States. As anticipated (Hypothesis 1), the table shows diverse patterns of demographic characteristics of respondents with their modal category being ages 25–34 (21%), female (51%), married (53%), employed for an employer (39%), never attending religious services (37%), having 9–15 years of education (57%), and native-born (94%) in the total sample. The same categories were found to be modal in the at-risk subsample except for age (35–44, 21%) and gender (male, 63%). Missing data were minimal, being 1.0% and 1.3% at most (immigration status) in the total sample and at-risk subsample, respectively.

Table 3 presents proportion (prevalence) of daily smoking as well as average daily cigarette consumption per capita (mean) and per individual who smoked daily (intensity) of 22 GFS countries in descending order. Türkiye had the highest mean (9.79), substantially larger than the overall mean (1.98), with next two highest countries being Argentina (3.88) and Indonesia (3.83). The three lowest were all African countries: Tanzania (0.23), Nigeria (0.26), and Kenya (0.28). Türkiye’s lowest Gini coefficient (0.67) indicated that the number of cigarettes smoked daily was more evenly dispersed among a wider range of survey participants compared to other countries, like Tanzania with the highest Gini coefficient being close to 1.00 (0.98), where daily smoking was reported only by a relatively few participants, consistent with its low prevalence (0.04). The top and bottom three countries in the mean remained the same in the prevalence, whereas the rank of in-between countries changed in a relatively minor manner. When the intensity was used to rank GFS countries, Türkiye remained the top with almost one pack of cigarettes (18.40) being consumed daily, but Egypt and Germany—which ranked numbers 7 and 5 in the mean (per capita) analysis—came in second (14.88) and third (13.75). Although Kenya (5.26, the lowest) and Tanzania (5.72) were found again among the bottom three countries, Nigeria (5.76)—which slightly moved up from number 21 in the mean to number 19 in the intensity—was replaced by Mexico (5.31), which moved down from number 17 in the mean to number 21 in the intensity. The rank of other countries changed between the two quantity measures of smoking in a varying degree with the largest move up and down being the U.S. (from numbers 16 to 8) and Indonesia (from numbers 3 to 13), respectively. As anticipated, the rank-order in the prevalence was more similar to that in the mean than the intensity. Specifically, the mean had a strong, positive Spearman’s correlation with the prevalence (0.889, *p* < .001), but the intensity was not significantly related to the prevalence (0.421, *p* = .051), as found previously^4^, though positively associated with the mean (0.730, *p* < .001). This finding indicates the potential usefulness of using intensity to assess country-level smoking along with prevalence or mean. In sum, the mean and intensity of daily smoking varied across countries (Hypothesis 2).

**Table 2:** Frequency distributions of demographic variables and GFS-participating country.

Variable	Total sample (*N* = 202,898)	At-risk subsample (*N* = 38,290)
Age group		
18–24	27,007 (13%)	3,846 (10%)
25–34	42,106 (21%)	7,893 (21%)
35–44	36,980 (18%)	8,039 (21%)
45–54	32,524 (16%)	7,095 (19%)
55–64	29,400 (14%)	6,351 (17%)
65–74	24,778 (12%)	4,101 (11%)
75–84	8,722 (4.3%)	864 (2.3%)
85 or older	1,361 (0.7%)	100 (0.3%)
(Missing)	20 (< 0.1%)	2 (< 0.1%)
Gender		
Male	98,411 (49%)	24,063 (63%)
Female	103,488 (51%)	14,060 (37%)
Other	602 (0.3%)	81 (0.2%)
(Missing)	397 (0.2%)	87 (0.2%)
Marital status		
Married	107,354 (53%)	18,569 (48%)
Separated	5,195 (2.6%)	1,402 (3.7%)
Divorced	11,654 (5.7%)	2,933 (7.7%)
Widowed	9,823 (4.8%)	1,501 (3.9%)
Single, never married	52,115 (26%)	9,874 (26%)
Domestic Partner	14,931 (7.4%)	3,558 (9.3%)
(Missing)	1,826 (0.9%)	453 (1.2%)
Employment		
Employed for an employer	78,815 (39%)	17,122 (45%)
Self-employed	36,362 (18%)	7,488 (20%)
Retired	29,303 (14%)	4,571 (12%)
Student	10,726 (5.3%)	1,257 (3.3%)
Homemaker	21,677 (11%)	2,386 (6.2%)
Unemployed and looking for a job	16,790 (8.3%)	3,410 (8.9%)
None of these/Other	8,431 (4.2%)	1,895 (4.9%)
(Missing)	793 (0.4%)	162 (0.4%)
Religious service attendance		
More than 1/week	26,537 (13%)	4,068 (11%)
1/week	39,157 (19%)	6,017 (16%)
1–3/month	19,749 (9.7%)	4,138 (11%)
A few times a year	41,436 (20%)	8,817 (23%)
Never	75,297 (37%)	15,104 (39%)
(Missing)	722 (0.4%)	146 (0.4%)
Education		
Up to 8 years	45,078 (22%)	8,383 (22%)
9–15 years	115,097 (57%)	24,116 (63%)
16 + years	42,578 (21%)	5,768 (15%)
(Missing)	146 (< 0.1%)	23 (< 0.1%)
Immigration status		
Born in this country	190,998 (94%)	36,281 (95%)
Born in another country	9,791 (4.8%)	1,493 (3.9%)
(Missing)	2,110 (1.0%)	517 (1.3%)
Country		
Argentina	6,724 (3.3%)	2,293 (6.0%)
Australia	3,844 (1.9%)	483 (1.3%)
Brazil	13,204 (6.5%)	2,785 (7.3%)
Egypt	4,729 (2.3%)	1,085 (2.8%)
Germany	9,506 (4.7%)	2,590 (6.8%)
Hong Kong	3,012 (1.5%)	956 (2.5%)
India	12,765 (6.3%)	1,484 (3.9%)
Indonesia	6,992 (3.4%)	2,944 (7.7%)
Israel	3,669 (1.8%)	882 (2.3%)
Japan	20,543 (10%)	4,766 (12%)
Kenya	11,389 (5.6%)	713 (1.9%)
Mexico	5,776 (2.8%)	1,438 (3.8%)
Nigeria	6,827 (3.4%)	368 (1.0%)
Philippines	5,292 (2.6%)	1,214 (3.2%)
Poland	10,389 (5.1%)	3,230 (8.4%)
South Africa	2,651 (1.3%)	669 (1.7%)
Spain	6,290 (3.1%)	2,082 (5.4%)
Sweden	15,068 (7.4%)	1,858 (4.9%)
Tanzania	9,075 (4.5%)	557 (1.5%)
Türkiye	1,473 (0.7%)	789 (2.1%)
United Kingdom	5,368 (2.6%)	928 (2.4%)
United States	38,312 (19%)	4,176 (11%)

At-risk subsample represents the subpopulation with each country that reported smoking at least once per day.

**Table 3:** Ordered average daily cigarette consumption per capita (mean) and proportions of daily smoking (prevalence) for the total sample and average daily cigarette consumption per individual who smoked daily (intensity) for the at-risk subsample.

Total sample (*N* = 202,898)									At-risk subsample (*N* = 38,290)				
Country	Mean	95% CI	S.D.	Gini	Country	Proportion	95% CI	S.E.	Country	Intensity	95% CI	S.D.	Gini
1. Türkiye	9.79	(8.99, 10.58)	13.25	0.67	1. Türkiye	0.53	(0.502, 0.564)	0.016	1. Türkiye	18.40	(17.32, 19.48)	13.14	0.37
2. Argentina	3.88	(3.59, 4.17)	8.01	0.81	2. Indonesia	0.38	(0.358, 0.394)	0.009	2. Egypt	14.88	(14.12, 15.63)	9.45	0.33
3. Indonesia	3.83	(3.59, 4.06)	6.55	0.76	3. Argentina	0.33	(0.313, 0.346)	0.008	3. Germany	13.75	(13.27, 14.22)	9.34	0.36
4. Poland	3.70	(3.44, 3.96)	6.98	0.79	4. Spain	0.32	(0.308, 0.339)	0.008	4. Japan	13.56	(13.29, 13.84)	8.21	0.31
5. Germany	3.65	(3.45, 3.86)	7.75	0.83	5. Hong Kong	0.31	(0.292, 0.335)	0.011	5. Israel	13.11	(11.96, 14.25)	9.81	0.37
6. Spain	3.60	(3.38, 3.83)	6.82	0.80	6. Poland	0.30	(0.286, 0.323)	0.010	6. Brazil	12.20	(11.73, 12.68)	9.57	0.39
7. Egypt	3.33	(3.01, 3.65)	7.64	0.85	7. Germany	0.27	(0.254, 0.277)	0.006	7. Poland	12.16	(11.56, 12.75)	7.58	0.30
8. Israel	3.08	(2.71, 3.45)	7.32	0.85	8. Israel	0.24	(0.214, 0.256)	0.011	8. U.S.	12.05	(11.34, 12.76)	8.64	0.39
9. Japan	3.02	(2.91, 3.12)	6.85	0.85	8. S. Africa	0.24	(0.213, 0.270)	0.014	9. Argentina	11.77	(11.14, 12.39)	10.08	0.43
10. Brazil	2.40	(2.25, 2.54)	6.40	0.88	10. Mexico	0.23	(0.219, 0.248)	0.007	10. Australia	11.37	(10.30, 12.44)	8.28	0.40
11. Hong Kong	2.01	(1.79, 2.23)	4.20	0.82	11. Egypt	0.22	(0.207, 0.241)	0.009	11. Spain	11.16	(10.72, 11.60)	7.70	0.37
12. U.K.	1.76	(1.57, 1.95)	5.09	0.90	11. Japan	0.22	(0.216, 0.229)	0.003	12. U.K.	10.74	(10.05, 11.44)	7.85	0.40
13. Philippines	1.73	(1.56, 1.91)	4.54	0.87	11. Philippines	0.22	(0.201, 0.234)	0.008	13. Indonesia	10.15	(9.75, 10.55)	7.03	0.35
14. S. Africa	1.70	(1.34, 2.06)	4.60	0.86	14. Brazil	0.20	(0.188, 0.206)	0.005	14. Sweden	8.77	(8.34, 9.21)	6.83	0.40
15. Australia	1.32	(1.12, 1.52)	4.60	0.93	15. U.K.	0.16	(0.150, 0.178)	0.007	15. India	8.28	(7.12, 9.44)	14.75	0.63
16. U.S.	1.25	(1.13, 1.38)	4.61	0.94	16. Australia	0.12	(0.102, 0.130)	0.007	16. Philippines	8.02	(7.49, 8.55)	6.73	0.41
17. Mexico	1.24	(1.08, 1.40)	4.14	0.89	17. Sweden	0.10	(0.090, 0.102)	0.003	17. S. Africa	7.04	(5.94, 8.15)	7.08	0.42
18. Sweden	0.84	(0.78, 0.91)	3.34	0.94	17. U.S.	0.10	(0.096, 0.112)	0.004	18. Hong Kong	6.43	(5.95, 6.91)	5.30	0.42
19. India	0.65	(0.55, 0.76)	4.69	0.97	19. India	0.08	(0.072, 0.086)	0.004	19. Nigeria	5.76	(4.51, 7.01)	10.71	0.53
20. Kenya	0.28	(0.24, 0.32)	1.92	0.97	20. Kenya	0.05	(0.047, 0.060)	0.003	20. Tanzania	5.72	(4.90, 6.54)	6.96	0.41
21. Nigeria	0.26	(0.19, 0.32)	2.52	0.98	21. Nigeria	0.04	(0.037, 0.053)	0.004	21. Mexico	5.31	(4.74, 5.89)	7.17	0.52
22. Tanzania	0.23	(0.19, 0.28)	1.81	0.98	21. Tanzania	0.04	(0.035, 0.047)	0.003	22. Kenya	5.26	(4.69, 5.83)	6.58	0.47

Overall mean = 1.98 (95% CI = 1.94, 2.02; S.D. = 5.73; Gini coefficient = 0.90), overall proportion = 0.175 (95% CI = 0.173, 0.178; SE = 0.0014), and overall intensity = 11.29 (95% CI = 11.14, 11.44; S.D. = 9.07; Gini coefficient = 0.40).

Table 4 shows results from random effects meta-analysis of country-specific means of daily cigarette smoking in each demographic category. Consistent with the WHO report on the prevalence of current smoking^2^, the relationship between age and the mean of daily smoking was curvilinear. That is, one of the two middle age groups, 45–54, had the highest overall mean [95% CI] (2.77 [1.84, 3.70]) with age groups 35–44 and 55–64 being a close second (2.76 [1.86, 3.66] and 2.76 [1.91, 3.61]). Adjacent age groups, 25–34 (2.35 [1.44, 3.26]) and 65–74 (1.77 [1.22, 2.33]), came in next, followed by age groups 18–24 (1.52 [0.79, 2.24]) and 75–84 (0.80 [0.54, 1.06]) with the age group 85 or older showing the lowest mean (0.28 [0.10, 0.47]). Various estimates (including 95% CI and standard error for the mean), computed separately for each age group, are reported next to each mean. For example, approximately 95% of the means of 22 countries for the age group 45–54 were estimated between 0.24 and 10.33, according to the “prediction interval,” constructed using an estimate of heterogeneity (2.20), the standard deviation of the distribution of means across those countries. Also, I^2^ estimate of the group “85 or older” (30.3) was substantially smaller than those of other groups (ranging from 90.8 to 99.8), indicating that the oldest group’s variability of means was due more to sample variability than heterogeneity across countries compared to their younger counterparts. Global *p*-value was significant (*p* < .001) and below the Bonferroni-corrected threshold (*p* < .007), showing that this demographic variable was significantly related to the mean of daily cigarette smoking at least in one of the 22 countries. In fact, the age group variable was significant (*p* < .001) in 20 of 22 countries with the exceptions being Mexico and Nigeria (Tables S12b and S13b of the online supplement).

**Table 4:** Random effects meta-analysis of average daily cigarette consumption per capita (mean) by demographic category: total sample (*N* = 202,898).

					*Prediction Interval*				
Variable	Category	Est	95% CI	SE	LL	UL	Heterogeneity (τ)	I^2^	Global p-value
Age group									< 0.001**
	18–24	1.52	(0.79,2.24)	0.37	0.09	7.98	1.72	99.8	
	25–34	2.35	(1.44,3.26)	0.47	0.21	10.24	2.17	99.8	
	35–44	2.76	(1.86,3.66)	0.46	0.31	9.81	2.14	99.6	
	45–54	2.77	(1.84,3.70)	0.47	0.24	10.33	2.20	99.6	
	55–64	2.76	(1.91,3.61)	0.43	0.11	8.53	2.01	99.3	
	65–74	1.77	(1.22,2.33)	0.28	0.07	4.88	1.27	98.5	
	75–84	0.80	(0.54,1.06)	0.13	0.08	1.80	0.47	90.8	
	85 or older	0.28	(0.10,0.47)	0.09	0.11	0.51	0.15	30.3	
Gender									< 0.001**
	Male	3.42	(2.18,4.66)	0.63	0.44	13.39	2.97	99.9	
	Female	1.41	(0.82,2.00)	0.30	0.05	5.61	1.40	99.9	
	Other	0.86	(0.40,1.33)	0.24	0.41	1.64	0.42	36.4	
Marital status									< 0.001**
	Married	2.23	(1.44,3.01)	0.40	0.24	8.54	1.87	99.8	
	Separated	2.74	(1.96,3.52)	0.40	0.51	6.17	1.71	95.9	
	Divorced	3.24	(1.99,4.50)	0.64	0.06	14.09	2.91	99.2	
	Widowed	1.57	(1.07,2.08)	0.26	0.07	3.46	1.11	97.1	
	Domestic partner	2.18	(1.58,2.78)	0.31	0.18	4.38	1.30	98.0	
	Single, never married	2.40	(1.40,3.41)	0.51	0.17	11.40	2.40	99.9	
Employment									< 0.001**
	Employed for an employer	2.76	(1.76,3.76)	0.51	0.18	10.49	2.38	99.8	
	Self-employed	3.23	(1.92,4.54)	0.67	0.30	14.63	3.11	99.8	
	Retired	1.96	(1.29,2.64)	0.34	0.22	7.27	1.57	99.0	
	Student	0.98	(0.52,1.44)	0.23	0.01	4.67	1.07	99.8	
	Homemaker	1.65	(1.00,2.30)	0.33	0.02	4.73	1.49	99.8	
	Unemployed and looking for a job	3.22	(2.07,4.37)	0.59	0.14	11.73	2.68	99.8	
	None of these/other	2.85	(1.84,3.86)	0.52	0.28	9.88	2.27	97.8	
Education									< 0.001**
	Up to 8 years	3.00	(2.10,3.90)	0.46	0.25	9.11	2.06	99.7	
	9–15 years	2.53	(1.65,3.41)	0.45	0.17	9.84	2.10	99.9	
	16 + years	1.65	(0.87,2.43)	0.40	0.09	8.92	1.85	99.8	
Religious service attendance									< 0.001**
	> 1/week	1.91	(1.12,2.70)	0.40	0.12	8.10	1.87	99.7	
	1/week	2.15	(1.31,2.98)	0.43	0.17	9.05	1.98	99.8	
	1–3/month	2.30	(1.51,3.09)	0.40	0.29	7.86	1.86	99.3	
	A few times a year	2.55	(1.57,3.53)	0.50	0.36	11.30	2.33	99.6	
	Never	2.74	(1.86,3.63)	0.45	0.58	10.04	2.10	99.6	
Immigration status									< 0.001**
	Born in this country	2.44	(1.59,3.30)	0.44	0.24	9.61	2.04	99.9	
	Born in another country	1.87	(1.16,2.59)	0.36	0.07	6.34	1.57	98.8	

τ = the standard deviation of the distribution of means across those countries; I^2^ = an estimate of how much of the variability in means is due to heterogeneity across countries vs. sampling variability.

* *p* < .05; ** *p* < .007 (Bonferroni corrected threshold).

As expected, average daily cigarette consumption per capita was higher among males (3.42 [2.18, 4.66]) than females (1.41 [0.82, 2.00]). Also, consistent with previous studies based on prevalence^11,17^, the mean of those who were married (2.23 [1.44, 3.01]) or living with a domestic partner (2.18 [1.58, 2.78]) was lower than the mean of those who were divorced (3.24 [1.97, 4.51]), separated (2.74 [1.96, 3.52]), or single/never married (2.40 [1.40, 3.41]), whereas the mean of the widowed was the lowest (1.57 [1.07, 2.08]).

Although prior research reported that unemployed individuals were less likely to smoke than their employed counterparts^13,19^, we found that respondents who were “unemployed and looking for a job” had the highest mean (3.22 [2.07, 4.37]) with the self-employed (3.23 [1.92, 4.54]) being a close second. The next two highest means were for respondents who chose the “none of these/other” category (2.85 [1.84, 3.86]) and those employed for an employer (2.76 [1.76, 3.76]), followed by the mean of retirees (1.96 [1.29, 2.64]) and homemakers (1.65 [1.00, 2.30]) with the lowest being found for students (0.98 [0.52, 1.44]). The relatively high mean for the employed, whether self-employed or employed for an employer, was anticipated. However, the top and third highest mean for the unemployed and the “none of these/other” were not expected, while these two categories’ high means are likely due to outliers found for each category in Türkiye (12.94 [9.97, 15.91] and 14.11 [8.54, 19.69]; Figures S23 and S24 of the online supplement).

Consistent with previous studies on prevalence^11,19,20^, education was inversely related to the mean of daily smoking. That is, average daily cigarette consumption per capita decreased as we moved from “up to 8 years of education” (3.00 [2.10, 3.90]) to “9–15 years” (2.53 [1.65, 3.42]) and to “16 or more years of education” (1.65 [0.87, 2.43]). The mean was also inversely related to religious service attendance, consistent with prior research^22^. Specifically, the mean increased as we moved from the most to the least frequent category: “more than once a week” (1.91 [1.12, 2.70]), “once a week” (2.15 [1.31, 2.98]), “one to three times a month” (2.30 [1.51, 3.09]), “a few times a year” (2.55 [1.57, 3.53]), and “never” (2.74 [1.86, 3.63]). Finally, we found that immigrants, that is, foreign-born respondents had a lower mean of daily cigarette smoking (1.87 [1.16, 2.59]) than their native-born counterparts (2.44 [1.59, 3.30]), as found in previous studies on prevalence^26–29^.

Table 5 presents results from meta-analyses of the intensity of daily smoking, which showed that the pattern of variations across categories remained the same in four of the seven demographic variables. Specifically, average daily cigarette consumption per individual who smoked was higher among males (10.66 [8.93, 12.39]) than females (9.64 [8.52, 10.75]) or the “other” gender (7.89 [3.72, 12.06]), whereas the intensity decreased as we moved from “up to 8 years” of education (11.22 [9.41, 13.03]) to “9–15 years” (10.39 [8.87, 11.91]) and to “16 or more years” (9.65 [8.22, 11.09]). Also, respondents who “never” attended religious services had higher intensity (11.11 [9.67, 12.55]) than those who did attend even “a few times a year” (10.65 [9.10, 12.21]), although respondents who attended more than once a week (9.29 [7.64, 10.95]) consumed slightly more, not fewer, cigarettes than those who attended once a week (9.14 [7.80, 10.48]) or 1–3 times a month (9.16 [7.78, 10.53]). The intensity was lower among foreign-born (9.18 [7.71, 10.65]) than native-born respondents (10.31 [8.82, 11.79]).

However, differences were observed in age, marital status, and employment. First, while age was still curvilinearly related to daily smoking in intensity, the peak was found for ages 55–64 (11.30 [9.60, 13.00]), instead of ages 45–54 (10.96 [9.50, 12.42]) that came in second. In addition, the youngest group, ages 18–24 (7.60 [6.22, 8.97]), had the lowest intensity with the oldest, ages 85 or older (8.31 [4.70, 11.92])—which had the lowest mean—showing next-to-lowest intensity. Second, although being divorced (12.33 [10.23, 14.43]) was the highest in the intensity just as it was in the mean, the rank of other categories changed. The most notable was the widowed (11.57 [9.80, 13.34]), which had the lowest mean, was the second highest in intensity. Third, the employment categories of “none of these/other” (11.37 [9.23, 13.50]) and “retired” (10.69 [9.03, 12.34]), which ranked numbers 3 and 5 in the mean both moved up to numbers 1 and 3 in the intensity, whereas “unemployed,” the top category in the mean, slightly moved down to the second highest (10.80 [9.12, 12.47]). The “student” category (7.14 [5.75, 8.53]) remained the lowest.

**Table 5:** Random effects meta-analysis of average daily cigarette consumption per individual who smoked daily (intensity) by demographic category: At-risk subsample (*N* = 38,290).

					*Prediction Interval*				
Variable	Category	Est	95% CI	SE	LL	UL	Heterogeneity (τ)	I^2^	Global p-value
Age group									< 0.001**
	18–24	7.60	(6.22,8.97)	0.70	3.66	16.29	3.15	95.1	
	25–34	9.22	(7.72,10.73)	0.77	4.49	18.28	3.52	97.8	
	35–44	10.48	(9.04,11.91)	0.73	5.27	18.38	3.34	97.4	
	45–54	10.96	(9.50,12.42)	0.75	5.21	18.84	3.40	97.3	
	55–64	11.30	(9.60,13.00)	0.87	2.82	17.42	3.96	97.8	
	65–74	10.87	(9.28,12.47)	0.81	4.68	16.34	3.51	94.4	
	75–84	9.21	(7.43,10.99)	0.91	2.38	12.42	3.08	92.7	
	85 or older	8.31	(4.70,11.92)	1.84	3.64	11.63	3.39	88.3	
Gender									< 0.001**
	Male	10.66	(8.93,12.39)	0.88	5.16	21.42	4.11	99.3	
	Female	9.64	(8.52,10.75)	0.57	4.09	13.33	2.42	96.9	
	Other	7.89	(3.72,12.06)	2.13	4.28	17.26	4.78	87.8	
Marital status									< 0.001**
	Married	10.17	(8.71,11.63)	0.74	5.13	17.21	3.45	98.8	
	Separated	9.99	(8.67,11.30)	0.67	5.16	13.92	2.48	80.2	
	Divorced	12.33	(10.23,14.43)	1.07	5.80	23.03	4.38	95.9	
	Widowed	11.57	(9.80,13.34)	0.90	5.63	16.42	3.45	89.6	
	Domestic partner	9.57	(8.10,11.03)	0.75	3.72	12.88	2.86	95.4	
	Single, never married	9.90	(8.37,11.42)	0.78	4.15	18.96	3.56	97.8	
Employment status									< 0.001**
	Employed for an employer	9.91	(8.40,11.42)	0.77	4.86	17.64	3.56	98.9	
	Self-employed	10.57	(8.90,12.23)	0.85	5.24	21.42	3.90	97.4	
	Retired	10.69	(9.03,12.34)	0.84	2.70	17.88	3.80	96.1	
	Student	7.14	(5.75,8.53)	0.71	2.88	13.02	2.86	93.1	
	Homemaker	10.28	(8.75,11.80)	0.78	4.50	14.67	3.16	89.3	
	Unemployed and looking for a job	10.80	(9.12,12.47)	0.85	3.99	18.40	3.79	95.9	
	None of these/other	11.37	(9.23,13.50)	1.09	3.68	22.88	4.59	95.7	
Education									< 0.001**
	Up to 8 years	11.22	(9.41,13.03)	0.92	4.60	18.93	4.08	97.2	
	9–15 years	10.39	(8.87,11.91)	0.78	5.13	17.84	3.60	99.2	
	16 + years	9.65	(8.22,11.09)	0.73	4.69	17.87	3.11	97.3	
Religious service attendance									< 0.001**
	> 1/week	9.29	(7.64,10.95)	0.85	4.19	18.11	3.74	96.5	
	1/week	9.14	(7.80,10.48)	0.68	4.81	16.77	3.06	95.7	
	1–3/month	9.16	(7.78,10.53)	0.70	4.36	15.62	3.11	95.4	
	A few times a year	10.65	(9.10,12.21)	0.79	5.26	19.12	3.59	97.7	
	Never	11.11	(9.67,12.55)	0.74	6.36	18.96	3.33	98.1	
Immigration status									< 0.001**
	Born in this country	10.31	(8.82,11.79)	0.76	5.21	18.15	3.53	99.3	
	Born in another country	9.18	(7.71,10.65)	0.75	2.90	17.03	2.88	93.7	

τ = the standard deviation of the distribution of means across those countries; I^2^ = an estimate of how much of the variability in means is due to heterogeneity across countries vs. sampling variability.

* *p* < .05; ** *p* < .007 (Bonferroni corrected threshold).

Results from supplementary meta-analysis of the prevalence (Table S47 of the online supplement) were more consistent with those of the mean than intensity in terms of the top category with a minor exception for marital status. That is, the highest prevalence was found for those being 45–54 (0.21 [0.16, 0.27]) as well as 35–44 years old (0.21 [0.16, 0.28]), male (0.26 [0.19, 0.34]), self-employed (0.24 [0.17, 0.32]), educated up to 8 years (0.22 [0.16, 0.28]), and native-born (0.19 [0.14, 0.25]) as well as having never attended religious services (0.22 [0.17, 0.27]). In terms of marital status, however, the top prevalence was found for the separated (0.25 [0.20, 0.32]), which switched the position with the divorced that had the highest mean.

In sum, daily cigarette smoking exhibited variations across demographic categories, and measures of dispersion indicated that those variations differed by country (Hypothesis 3).

While meta-analysis estimated overall mean, synthesizing 22 country-specific results of daily cigarette smoking for each demographic category, the results varied around the overall mean across those countries. For example, the overall mean of daily smoking intensity was similar between male (10.66 [8.93, 12.39]) and female smokers (9.64 [8.52, 10.75]), but male intensity varied across GFS countries more than female intensity, as indicated by larger heterogeneity of male (4.11) than female intensity (2.42) or wider 95% prediction interval for males (5.16–21.42) than females (4.09–13.33). Forest plots visualize that the greater heterogeneity was due to Türkiye’s male intensity (21.60 [20.15, 23.04]) being distant from all other countries’, which varied between 5.14 ([4.61, 5.67], Kenya) and 14.95 ([14.20, 15.71], Egypt), with female intensity varying between 4.06 ([3.59, 4.54], Mexico) to 13.48 ([12.15, 14.81], Türkiye) with no outlier (Figures S42 and S43 of the online supplement).

Furthermore, although males were found to consume daily only about one more cigarette than female smokers globally, gender differences in the intensity varied widely across those countries. Specifically, when we calculated a male-to-female ratio of the intensity, it ranged from 0.48 to 1.68 with females consuming more cigarettes daily than males in eight of 22 GFS countries—Tanzania (0.48), Nigeria (0.60), India (0.78), Kenya (0.79), South Africa (0.85), the U.K. (0.86), Sweden (0.90), and Hong Kong (0.95)—and the other way around in the remining countries—Australia (1.08), Germany (1.09), the U.S. (1.10), Brazil (1.11), Japan (1.17), Spain (1.17), Argentina (1.18), Poland (1.23), Mexico (1.45), Indonesia (1.46), Israel (1.52), Türkiye (1.60), Egypt (1.66), and the Philippines (1.68) (Tables S24b to S45b of the online supplement). When we calculated the ratio using the prevalence instead (Tables S48 to S69 of the online supplement), only Sweden was identified as a country where female smoking rate was higher than male rate (0.88), as reported by a recent study based on 2019 estimates of tobacco smoking prevalence^7^. Sweden was also the only country with the gender ratio less than 1.00 (0.79) when the mean was used (Tables S1b to S22b of the online supplement). These results further illustrate the importance of studying intensity as well as the common measure of smoking, prevalence.

Examining variation across countries also helped us see why meta-analytic results for the categories of employment were so different between the intensity and prevalence. For instance, the self-employed, which had the highest overall proportion of all seven categories (Table S47 of the online supplement), was found to have the highest prevalence in seven of 22 GFS countries (Brazil, Egypt, Hong Kong, Israel, Japan, South Africa, and Türkiye; Tables S50, S51, S53, S56, S57, S63, and S67 of the online supplement), but that category had the highest intensity only in one of the seven (Japan) besides two other countries (Nigeria and Tanzania) (Tables S33b, S36b, S42b of the online supplement).

## Discussion

According to a recent study that estimated global trends in smoking among persons aged 15 or over between 1990 and 2019 for 204 countries and territories (including all 22 GFS countries), the prevalence of current tobacco smoking decreased by 29.5%, from 27.8 to 19.6%, with the 2019 prevalence being 32.7% (27.5% decrease) among males and 6.6% (37.7% decrease) among females^7,54^. This progress was likely due in part to the WHO Framework Convention on Tobacco Control (FCTC)—which was the first international public health treaty, ratified by 182 countries since 2003—and MPOWER, which consists of six measures designed to implement the FCTC, adopted at least partly by 151 countries since 2007^2^. For example, the largest decreases were observed in Brazil, a GFS country that had fully adopted the MPOWER by 2011: 73.4% reduction (72.5% and 74.7% reductions among males and females, respectively). However, despite the important progress made in prevalence, during the same 30-year period the number of individuals who smoked increased by 15.2%, from 0.99 to 1.14 billion, as a result of population growth. This increased number and a large tobacco consumption worldwide (e.g., 7.41 trillion cigarette-equivalents of tobacco consumed in 2019) point to the importance of studying not only prevalence but also daily cigarette consumption, a key predictor of disease risk even when only a few cigarettes are smoked daily^7,31^.

In this context, we analyzed nationally representative data from 22 countries—which participated in the Global Flourishing Study’s first of five panel surveys, conducted primarily in 2023—to examine those countries’ average daily cigarette consumption per capita (mean) and per individual who smoked (intensity) in comparison with the common measure, prevalence of daily cigarette smoking. We found that the rank-order of GFS countries and meta-analytic results of variations across demographic categories using the mean were generally similar to those using the prevalence, whereas the intensity was not correlated with the prevalence, as found previously in a rare investigation of these two measures^4^. These findings indicated that the average per capita was largely consistent with the prevalence, whereas the intensity was distinct from the most widely used measure of smoking. Thus, it is important to use intensity as well as prevalence of smoking to establish informed tobacco control policies as well as assessing tobacco-associated health risks^4^.

To illustrate, we divided GFS countries into three approximately equal groups in terms of prevalence and intensity (low, middle, and high) to create a 3 × 3 typology: (1) low-prevalence-low-intensity (Kenya, Nigeria, and Tanzania), (2) low-prevalence-middle-intensity (Australia, India, Sweden, and the U.S.), (3) low-prevalence-high-intensity (no country), (4) middle-prevalence-low-intensity (Mexico, the Philippines, and South Africa), (5) middle-prevalence-middle-intensity (the U.K.), (6) middle-prevalence-high-intensity (Brazil, Egypt, Israel, and Japan), (7) high-prevalence-low intensity (Hong Kong), (8) high-prevalence-middle-intensity (Argentina, Indonesia, and Spain), and (9) high-prevalence-high-intensity countries (Germany, Poland, and Türkiye). Various factors, such as a country’s socioeconomic status, smoking culture, and tobacco control policy, are likely to determine where the country belongs in the typology. For example, the low-prevalence-low-intensity category consists of low-income countries^2^, while some high-income countries were found in a low-prevalence category as well perhaps due to their public health programs restricting drug use (e.g., Australia). It is interesting that prevalence of daily smoking is relatively high in Hong Kong, but intensity is low. We speculate that this may have to do with its smoking culture: for example, cigarette smoking may be a part of daily social interactions, particularly, among men as is the case with Asian countries, (e.g., Japan and South Korea), but high level of daily consumption is not. Also, it was not surprising to see Germany in the high-prevalence-high-intensity category given that the country has limited ban on smoking in public places and relatively low tax on cigarettes^1^.

This typological approach is important because it has implications for formulating global as well as national health policies. That is, a typology like this actually captures different aspects simultaneously, thereby helping to identify countries that are at higher health-risk than others which may otherwise be overlooked or underrecognized if only prevalence were used to measure a country’s state of smoking. Specifically, compared to those in the low-low, middle-middle, and high-and high categories, countries in other categories are likely subject to incorrect assessment of health-risk. For example, the health-risk of low-prevalence-middle-intensity (Australia, India, Sweden, and the U.S.) and middle-prevalence-high-intensity countries (Brazil, Egypt, Israel, and Japan) will be underestimated with a prevalence measure only, as they will be assessed to be at lower risk based on prevalence rather than intensity as well as prevalence. Furthermore, using the two distinct measures jointly will help a country to determine how different anti-tobacco programs should be prioritized. For instance, the present finding suggests that high-prevalence-low-intensity Hong Kong should assign more resources to smoking initiation prevention than reduction and cessation programs. Similarly, a low-prevalence-high-intensity country would benefit from programs that focus on at-risk individuals (i.e., those who daily smoke cigarettes intensely) although no GFS country was found in that category.

Comparisons of meta-analytic results revealed noteworthy differences between the prevalence and intensity of daily smoking in variations across categories of three demographic variables. First, the age group 45–54 that had the highest prevalence was found to be lower in intensity than two older groups, 55–64 and 65–74. Based solely on prevalence, these older individuals who smoked would receive lower priority than they deserve in policy and research on smoking reduction and cessation, while their lower prevalence may be due partly to those who smoke intensely being likely to die at those ages. Second, the widowed, the lowest in prevalence, was found to have the second highest in intensity. If we did not use intensity, we would have not known the importance of this low-prevalence-but-high-intensity category for health research on smoking. Third, although previous studies reported that the unemployed had lower prevalence of smoking than the employed^13,19^, we found that the unemployed was not much different from the self-employed and even higher than those employed for an employer in the prevalence of daily cigarette smoking. This observed difference may be due to our having meta-analyzed without controlling for other demographic variables (e.g., education and income, likely confounders of the employment-smoking relationship), unlike previous studies. Fourth, like the older age groups or the widowed, the retired was found to be a low-prevalence-high-intensity category, which would have been overlooked if we relied only on prevalence, while their low prevalence could be due in part to a survival bias. In sum, these observed differences in meta-analytic results provide additional evidence of the importance of utilizing the intensity as well as prevalence of daily smoking for health research.

As a supplement to random effects meta-analysis, we conducted population-weighted meta-analysis, where each country’s results were weighted by its population size in 2023, equally treating each person in the 22 countries instead of each country. As a result, India, the largest country that constituted about 40% of all GFS country populations combined, received the most weight^34^. These alternative meta-analysis results were consistent with their random effects counterparts for some variables but not for others, reflecting greater weights given to the results of larger countries. For example, the top-intensity category remained the same for education (up to 8 years) and religious service attendance (never) in the population-weighted meta-analysis but were different for age (45–54, not 55–64), gender (female, not male), marital status (widowed, not divorced), employment (self-employed, not none of these/other), and immigration status (foreign-born, not native-born) (Table S46 of the online supplement). Three of the five new top categories (gender, marital status, and immigration status) were consistent with what we found for India (Table S30b of the online supplement).

Finally, there are two important caveats to keep in mind when our results are interpreted. First, although self-report is a common method to measure smoking in population-based studies, like the current study, prior research using biomarkers (e.g., cotinine) showed the method’s underestimation bias due to socially desirable reporting^55–57^; however, the bias does not necessarily negate the validity of self-reported smoking^58,59^. For example, in examining the meta-analytic results and ordered estimates of daily smoking among GFS countries, it needs to be considered that respondents in countries with aggressive anti-tobacco policies and legislation—such as Brazil, Spain, and Türkiye^1^—might have underreported smoking to a greater extent than those in countries without such policies or legislation. Second, while the Global Flourishing Study took necessary steps (including cognitive and pretest interviews) to ensure that survey items would be understood similarly across countries^41,60,61^, respondents’ interpretation of some items might have varied across different cultures. For this reason, the present findings should be interpreted with caution given various issues related to language translations and culture-influenced responses.

In conclusion, based on new data from 22 GFS-participating countries, this study contributes to global research on tobacco use by examining country-level cigarette consumption per individual who smoked daily, which is rarely studied in cross-national research. This intensity measure of daily cigarette smoking being unrelated to daily prevalence and observed differences in meta-analysis results between the two measures is evidence of the importance of using this understudied measure in health research, as it is a key predictor of smoking-related diseases. Thus, we propose that future surveys on smoking include an item asking about the quantity of cigarette consumption, like the one used in this study or the Global Adult Tobacco Survey (GATS; https://www.who.int/teams/noncommunicable-diseases/surveillance/systems-tools/global-adult-tobacco-survey), which will enable researchers to create both intensity and prevalence measures so they can compare them and also use both measures to develop a typology that will provide policy makers with a refined classification of a country’s health-risk due to smoking. When such item is included, it should inquire about *daily* consumption given that even less than one cigarette, let alone only a few, smoked daily significantly increases a risk of tobacco-associated diseases^7,31^. Future surveys should also ask about understudied demographic characteristics, such as religiousness that we found to be a protective factor for smoking, and immigration status, which has a potential contribution to studying cigarette use in a time of global migration. In addition, employment, studied previously but crudely (e.g., employed vs. unemployed), needs to be inquired about using diverse response options—as it was in the GFS survey—for nuanced comparisons among various employment statuses. Finally, future research on demographic variations in smoking should utilize intensity as well as prevalence measure to detect high-risk demographic groups flying under the radar, such as the widowed and the retired, as the present study showed.

## Electronic supplementary material

Below is the link to the electronic supplementary material.

Supplementary Material 1

## Data Availability

The data are available through the Center for Open Science (https://www.cos.io/gfs).
