# Author Correction: A cross-national analysis of demographic variation in daily smoking across 22 countries

**DOI:** 10.1038/s41598-025-05433-y

**Published:** 2025-06-16

**Authors:** Sung Joon Jang, Pedro A. de la Rosa, R. Noah Padgett, Matt Bradshaw, Tyler J. VanderWeele, Byron R. Johnson

**Affiliations:** 1 Baylor University, Waco, USA; 2 Pepperdine University, Malibu, USA; 3 University of Navarra, Pamplona, Spain; 4 Harvard University, Cambridge, USA; 5 Harvard T.H. Chan School of Public Health, Boston, USA

Correction to: *Scientific Reports* 10.1038/s41598-024-76318-9, published online 30 April 2025

The original version of this article contained errors.

In the original version of this article, Table 1 contained an incomplete value description.

Incorrect:

**Table:** 

Country (WHO region)	WHO, 2022		OECD, 2019	Ng et al., 2012	WHO, 2010	WHO, 2016	
	Age-	Crude adjusted prevalence (CAP) of current cigarette smoking	% of people who smoke cigarettes daily	Mean daily cigarette consumption per individual who smoked	CAP of daily tobacco smoking	ASP of daily tobacco smoking	CAP of daily cigarette smoking

Correct:

**Table:** 

Country (WHO region)	WHO, 2022		OECD, 2019	Ng et al., 2012	WHO, 2010	WHO, 2016	
	Age-standardised prevalence (ASP) of current cigarette smoking^a^	Crude adjusted prevalence (CAP) of current cigarette smoking	% of people who smoke cigarettes daily	Mean daily cigarette consumption per individual who smoked	CAP of daily tobacco smoking	ASP of daily tobacco smoking	CAP of daily cigarette smoking

In addition, a set of mean values was incorrectly typeset under the Results section.

“Table 4 shows results from a random effects meta-analysis of country-specific means of daily cigarette smoking in each demographic category. Consistent with the WHO report on the prevalence of current smoking^2^, the relationship between age and the mean of daily smoking was curvilinear. That is, one of the two middle age groups, 45–54, had the highest overall mean [95% CI] (2.78 [1.85, 3.71]) with age groups 35–44 and 55–64 being a close second (2.76 [1.86, 3.66] and 2.76 [1.91, 3.61]). Adjacent age groups, 25–34 (2.35 [1.44, 3.27]) and 65–74 (1.76 [1.20, 2.32]), came in next, followed by age groups 18–24 (1.52 [0.80, 2.24]) and 75–84 (0.80 [0.54, 1.06]) with the age group 85 or older showing the lowest mean (0.24 [0.09, 0.39]). Various estimates (including 95% CI and standard error for the mean), computed separately for each age group, are reported next to each mean. For example, approximately 95% of the means of 22 countries for the age group 45–54 were estimated between 0.24 and 10.38, according to the “prediction interval,” constructed using an estimate of heterogeneity (2.21), the standard deviation of the distribution of means across those countries. Also, I^2^ estimate of the group “85 or older” (14.1) was substantially smaller than those of other groups (ranging from 90.9 to 99.8), indicating that the oldest group’s variability of means was due more to sample variability than heterogeneity across countries compared to their younger counterparts. Global *p*-value was significant (*p* < .001) and below the Bonferroni-corrected threshold (*p* < .007), showing that this demographic variable was significantly related to the mean of daily cigarette smoking at least in one of the 22 countries. In fact, the age group variable was significant (*p* < .001) in 20 of 22 countries, with the exceptions being Mexico and Nigeria (Tables S12b and S13b of the online supplement).

As expected, average daily cigarette consumption per capita was higher among males (3.43 [2.19, 4.68]) than females (1.42 [0.83, 2.01]). Also, consistent with previous studies based on prevalence^11,17^, the mean of those who were married (2.23 [1.44, 3.01]) or living with a domestic partner (2.18 [1.57, 2.79]) was lower than the mean of those who were divorced (3.24 [1.97, 4.51]), separated (2.75 [1.98, 3.52]), or single/never married (2.43 [1.43, 3.43]), whereas the mean of the widowed was the lowest (1.60 [1.09, 2.11]).

Although prior research reported that unemployed individuals were less likely to smoke than their employed counterparts^13,19^, we found that respondents who were “unemployed and looking for a job” had the highest mean (3.24 [2.10, 4.39]) with the self-employed (3.23 [1.93, 4.54]) being a close second. The next two highest means were for respondents who chose the “none of these/other” category (2.87 [1.86, 3.88]) and those employed for an employer (2.76 [1.76, 3.76]), followed by the mean of retirees (1.95 [1.28, 2.63]) and homemakers (1.65 [1.00, 2.30]) with the lowest being found for students (0.99 [0.53, 1.44]). The relatively high mean for the employed, whether self-employed or employed for an employer, was anticipated. However, the top and third highest mean for the unemployed and the “none of these/other” were not expected, while these two categories’ high means are likely due to outliers found for each category in Türkiye (12.94 [9.97, 15.91] and 14.11 [8.54, 19.69]; Figures S23 and S24 of the online supplement).

Consistent with previous studies on prevalence^11,19,20^, education was inversely related to the mean of daily smoking. That is, average daily cigarette consumption per capita decreased as we moved from “up to 8 years of education” (3.02 [2.11, 3.93]) to “9–15 years” (2.54 [1.65, 3.42]) and to “16 or more years of education” (1.66 [0.89, 2.44]). The mean was also inversely related to religious service attendance, consistent with prior research^22^. Specifically, the mean increased as we moved from the most to the least frequent category: “more than once a week” (1.90 [1.11, 2.70]), “once a week” (2.16 [1.32, 3.00]), “one to three times a month” (2.31 [1.51, 3.11]), “a few times a year” (2.56 [1.58, 3.54]), and “never” (2.75 [1.87, 3.64]). Finally, we found that immigrants, that is, foreign-born respondents had a lower mean of daily cigarette smoking (1.89 [1.18, 2.61]) than their native-born counterparts (2.45 [1.60, 3.31]), as found in previous studies on prevalence^26,27,28,29^.”

now reads,

“Table 4 shows results from a random effects meta-analysis of country-specific means of daily cigarette smoking in each demographic category. Consistent with the WHO report on the prevalence of current smoking^2^, the relationship between age and the mean of daily smoking was curvilinear. That is, one of the two middle age groups, 45–54, had the highest overall mean [95% CI] (2.77 [1.84, 3.70]) with age groups 35–44 and 55–64 being a close second (2.76 [1.86, 3.66] and 2.76 [1.91, 3.61]). Adjacent age groups, 25–34 (2.35 [1.44, 3.26]) and 65–74 (1.77 [1.22, 2.33]), came in next, followed by age groups 18–24 (1.52 [0.79, 2.24]) and 75–84 (0.80 [0.54, 1.06]) with the age group 85 or older showing the lowest mean (0.28 [0.10, 0.47]). Various estimates (including 95% CI and standard error for the mean), computed separately for each age group, are reported next to each mean. For example, approximately 95% of the means of 22 countries for the age group 45–54 were estimated between 0.24 and 10.33, according to the “prediction interval,” constructed using an estimate of heterogeneity (2.20), the standard deviation of the distribution of means across those countries. Also, I^2^ estimate of the group “85 or older” (30.3) was substantially smaller than those of other groups (ranging from 90.8 to 99.8), indicating that the oldest group’s variability of means was due more to sample variability than heterogeneity across countries compared to their younger counterparts. Global *p*-value was significant (*p* < .001) and below the Bonferroni-corrected threshold (*p* < .007), showing that this demographic variable was significantly related to the mean of daily cigarette smoking at least in one of the 22 countries. In fact, the age group variable was significant (*p* < .001) in 20 of 22 countries, with the exceptions being Mexico and Nigeria (Tables S12b and S13b of the online supplement).

As expected, average daily cigarette consumption per capita was higher among males (3.42 [2.18, 4.66]) than females (1.41 [0.82, 2.00]). Also, consistent with previous studies based on prevalence^11,17^, the mean of those who were married (2.23 [1.44, 3.01]) or living with a domestic partner (2.18 [1.58, 2.78]) was lower than the mean of those who were divorced (3.24 [1.99, 4.50]), separated (2.74 [1.96, 3.52]), or single/never married (2.40 [1.40, 3.41]), whereas the mean of the widowed was the lowest (1.57 [1.07, 2.08]).

Although prior research reported that unemployed individuals were less likely to smoke than their employed counterparts^13,19^, we found that respondents who were “unemployed and looking for a job” had the highest mean (3.22 [2.07, 4.37]) with the self-employed (3.23 [1.92, 4.54]) being a close second. The next two highest means were for respondents who chose the “none of these/other” category (2.85 [1.84, 3.86]) and those employed for an employer (2.76 [1.76, 3.76]), followed by the mean of retirees (1.96 [1.29, 2.64]) and homemakers (1.65 [1.00, 2.30]) with the lowest being found for students (0.98 [0.52, 1.44]). The relatively high mean for the employed, whether self-employed or employed for an employer, was anticipated. However, the top and third highest mean for the unemployed and the “none of these/other” were not expected, while these two categories’ high means are likely due to outliers found for each category in Türkiye (12.94 [9.97, 15.91] and 14.11 [8.54, 19.69]; Figures S23 and S24 of the online supplement).

Consistent with previous studies on prevalence^11,19,20^, education was inversely related to the mean of daily smoking. That is, average daily cigarette consumption per capita decreased as we moved from “up to 8 years of education” (3.00 [2.10, 3.90]) to “9–15 years” (2.53 [1.65, 3.41]) and to “16 or more years of education” (1.65 [0.87, 2.43]). The mean was also inversely related to religious service attendance, consistent with prior research^22^. Specifically, the mean increased as we moved from the most to the least frequent category: “more than once a week” (1.91 [1.12, 2.70]), “once a week” (2.15 [1.31, 2.98]), “one to three times a month” (2.30 [1.51, 3.09]), “a few times a year” (2.55 [1.57, 3.53]), and “never” (2.74 [1.86, 3.63]). Finally, we found that immigrants, that is, foreign-born respondents had a lower mean of daily cigarette smoking (1.87 [1.16, 2.59]) than their native-born counterparts (2.44 [1.59, 3.30]), as found in previous studies on prevalence^26,27,28,29^.”

The original Article has been corrected.

