# Supplementary material for: A cross-national analysis of demographic variation in daily smoking across 22 countries
Source: Sci Rep. 2025 Apr 30;15:14324. doi: 10.1038/s41598-024-76318-9 (PMC12043897; doi:10.1038/s41598-024-76318-9)
Supplement: Supplementary file 1 — Supplementary Material 1 [file 41598_2024_76318_MOESM1_ESM.docx]

**Supplementary Tables and Figures**

A Cross-National Analysis of Demographic Variation in Daily Smoking Across 22 Countries

Table of Contents

[1. Table S1a. Nationally representative descriptive statistics for Argentina 5](#_Toc173834592)

[2. Table S1b. Means by demographic category for Argentina 7](#_Toc173834593)

[3. Table S2a. Nationally representative descriptive statistics for Australia 9](#_Toc173834594)

[4. Table S2b. Means by demographic category for Australia 12](#_Toc173834595)

[5. Table S3a. Nationally representative descriptive statistics for Brazil 14](#_Toc173834596)

[6. Table S3b. Means by demographic category for Brazil 16](#_Toc173834597)

[7. Table S4a. Nationally representative descriptive statistics for Egypt 18](#_Toc173834598)

[8. Table S4b. Means by demographic category for Egypt 20](#_Toc173834599)

[9. Table S5a. Nationally representative descriptive statistics for Germany 22](#_Toc173834600)

[10. Table S5b. Means by demographic category for Germany 24](#_Toc173834601)

[11. Table S6a. Nationally representative descriptive statistics for Hong Kong 26](#_Toc173834602)

[12. Table S6b. Means by demographic category for Hong Kong 28](#_Toc173834603)

[13. Table S7a. Nationally representative descriptive statistics for India 30](#_Toc173834604)

[14. Table S7b. Means by demographic category for India 32](#_Toc173834605)

[15. Table S8a. Nationally representative descriptive statistics for Indonesia 34](#_Toc173834606)

[16. Table S8b. Means by demographic category for Indonesia 36](#_Toc173834607)

[17. Table S9a. Nationally representative descriptive statistics for Israel 38](#_Toc173834608)

[18. Table S9b. Means by demographic category for Israel 40](#_Toc173834609)

[19. Table S10a. Nationally representative descriptive statistics for Japan 42](#_Toc173834610)

[20. Table S10b. Means by demographic category for Japan 44](#_Toc173834611)

[21. Table S11a. Nationally representative descriptive statistics for Kenya 46](#_Toc173834612)

[22. Table S11b. Means by demographic category for Kenya 48](#_Toc173834613)

[23. Table S12a. Nationally representative descriptive statistics for Mexico 50](#_Toc173834614)

[24. Table S12b. Means by demographic category for Mexico 52](#_Toc173834615)

[25. Table S13a. Nationally representative descriptive statistics for Nigeria 54](#_Toc173834616)

[26. Table S13b. Means by demographic category for Nigeria 56](#_Toc173834617)

[27. Table S14a. Nationally representative descriptive statistics for Philippines 58](#_Toc173834618)

[28. Table S14b. Means by demographic category for Philippines 61](#_Toc173834619)

[29. Table S15a. Nationally representative descriptive statistics for Poland 63](#_Toc173834620)

[30. Table S15b. Means by demographic category for Poland 65](#_Toc173834621)

[31. Table S16a. Nationally representative descriptive statistics for South Africa 67](#_Toc173834622)

[32. Table S16b. Means by demographic category for South Africa 69](#_Toc173834623)

[33. Table S17a. Nationally representative descriptive statistics for Spain 71](#_Toc173834624)

[34. Table S17b. Means by demographic category for Spain 73](#_Toc173834625)

[35. Table S18a. Nationally representative descriptive statistics for Sweden 75](#_Toc173834626)

[36. Table S18b. Means by demographic category for Sweden 77](#_Toc173834627)

[37. Table S19a. Nationally representative descriptive statistics for Tanzania 79](#_Toc173834628)

[38. Table S19b. Means by demographic category for Tanzania 81](#_Toc173834629)

[39. Table S20a. Nationally representative descriptive statistics for Türkiye 83](#_Toc173834630)

[40. Table S20b. Means by demographic category for Türkiye 85](#_Toc173834631)

[41. Table S21a. Nationally representative descriptive statistics for United Kingdom 87](#_Toc173834632)

[42. Table S21b. Means by demographic category for United Kingdom 89](#_Toc173834633)

[43. Table S22a. Nationally representative descriptive statistics for United States 91](#_Toc173834634)

[44. Table S22b. Means by demographic category for United States 93](#_Toc173834635)

[45. Table S23. Population weighted meta-analysis of daily cigarette consumption per capita (mean) by demographic category 95](#_Toc173834636)

[46. Table S24a. Descriptive statistics for Argentina conditional on smokers 96](#_Toc173834637)

[47. Table S24b. Intensity by demographic category for Argentina conditional on smokers 98](#_Toc173834638)

[48. Table S25a. Descriptive statistics for Australia conditional on smokers 100](#_Toc173834639)

[49. Table S25b. Intensity by demographic category for Australia conditional on smokers 103](#_Toc173834640)

[50. Table S26a. Descriptive statistics for Brazil conditional on smokers 105](#_Toc173834641)

[51. Table S26b. Intensity by demographic category for Brazil conditional on smokers 107](#_Toc173834642)

[52. Table S27a. Descriptive statistics for Egypt conditional on smokers 109](#_Toc173834643)

[53. Table S27b. Intensity by demographic category for Egypt conditional on smokers 111](#_Toc173834644)

[54. Table S28a. Descriptive statistics for Germany conditional on smokers 113](#_Toc173834645)

[55. Table S28b. Intensity by demographic category for Germany conditional on smokers 115](#_Toc173834646)

[56. Table S29a. Descriptive statistics for Hong Kong conditional on smokers 117](#_Toc173834647)

[57. Table S29b. Intensity by demographic category for Hong Kong conditional on smokers 119](#_Toc173834648)

[58. Table S30a. Descriptive statistics for India conditional on smokers 121](#_Toc173834649)

[59. Table S30b. Intensity by demographic category for India conditional on smokers 123](#_Toc173834650)

[60. Table S31a. Descriptive statistics for Indonesia conditional on smokers 125](#_Toc173834651)

[61. Table S31b. Intensity by demographic category for Indonesia conditional on smokers 127](#_Toc173834652)

[62. Table S32a. Descriptive statistics for Israel conditional on smokers 129](#_Toc173834653)

[63. Table S32b. Intensity by demographic category for Israel conditional on smokers 131](#_Toc173834654)

[64. Table S33a. Descriptive statistics for Japan conditional on smokers 133](#_Toc173834655)

[65. Table S33b. Intensity by demographic category for Japan conditional on smokers 135](#_Toc173834656)

[66. Table S34a. Descriptive statistics for Kenya conditional on smokers 137](#_Toc173834657)

[67. Table S34b. Intensity by demographic category for Kenya conditional on smokers 139](#_Toc173834658)

[68. Table S35a. Descriptive statistics for Mexico conditional on smokers 141](#_Toc173834659)

[69. Table S35b. Intensity by demographic category for Mexico conditional on smokers 143](#_Toc173834660)

[70. Table S36a. Descriptive statistics for Nigeria conditional on smokers 145](#_Toc173834661)

[71. Table S36b. Intensity by demographic category for Nigeria conditional on smokers 147](#_Toc173834662)

[72. Table S37a. Descriptive statistics for Philippines conditional on smokers 149](#_Toc173834663)

[73. Table S37b. Intensity by demographic category for Philippines conditional on smokers 152](#_Toc173834664)

[74. Table S38a. Descriptive statistics for Poland conditional on smokers 154](#_Toc173834665)

[75. Table S38b. Intensity by demographic category for Poland conditional on smokers 156](#_Toc173834666)

[76. Table S39a. Descriptive statistics for South Africa conditional on smokers 158](#_Toc173834667)

[77. Table S39b. Intensity by demographic category for South Africa conditional on smokers 160](#_Toc173834668)

[78. Table S40a. Descriptive statistics for Spain conditional on smokers 162](#_Toc173834669)

[79. Table S40b. Intensity by demographic category for Spain conditional on smokers 164](#_Toc173834670)

[80. Table S41a. Descriptive statistics for Sweden conditional on smokers 166](#_Toc173834671)

[81. Table S41b. Intensity by demographic category for Sweden conditional on smokers 168](#_Toc173834672)

[82. Table S42a. Descriptive statistics for Tanzania conditional on smokers 170](#_Toc173834673)

[83. Table S42b. Intensity by demographic category for Tanzania conditional on smokers 172](#_Toc173834674)

[84. Table S43a. Descriptive statistics for Türkiye conditional on smokers 174](#_Toc173834675)

[85. Table S43b. Intensity by demographic category for Türkiye conditional on smokers 176](#_Toc173834676)

[86. Table S44a. Descriptive statistics for United Kingdom conditional on smokers 178](#_Toc173834677)

[87. Table S44b. Intensity by demographic category for United Kingdom conditional on smokers 180](#_Toc173834678)

[88. Table S45a. Descriptive statistics for United States conditional on smokers 182](#_Toc173834679)

[89. Table S45b. Intensity by demographic category for United States conditional on smokers 184](#_Toc173834680)

[90. Table S46. Population weighted meta-analysis of daily cigarette consumption per smoker (intensity) by demographic category 186](#_Toc173834681)

[91. Table S47. Random effects meta-analysis of proportion (prevalence) of daily cigarette smoking by demographic category. 187](#_Toc173834682)

[92. Table S48. Proportions by demographic category for Argentina 188](#_Toc173834683)

[93. Table S49. Proportions by demographic category for Australia 190](#_Toc173834684)

[94. Table S50. Proportions by demographic category for Brazil 192](#_Toc173834685)

[95. Table S51. Proportions by demographic category for Egypt 194](#_Toc173834686)

[96. Table S52. Proportions by demographic category for Germany 196](#_Toc173834687)

[97. Table S53. Proportions by demographic category for Hong Kong 198](#_Toc173834688)

[98. Table S54. Proportions by demographic category for India 200](#_Toc173834689)

[99. Table S55. Proportions by demographic category for Indonesia 202](#_Toc173834690)

[100. Table S56. Proportions by demographic category for Israel 204](#_Toc173834691)

[101. Table S57. Proportions by demographic category for Japan 206](#_Toc173834692)

[102. Table S58. Proportions by demographic category for Kenya 208](#_Toc173834693)

[103. Table S59. Proportions by demographic category for Mexico 210](#_Toc173834694)

[104. Table S60. Proportions by demographic category for Nigeria 212](#_Toc173834695)

[105. Table S61. Proportions by demographic category for Philippines 214](#_Toc173834696)

[106. Table S62. Proportions by demographic category for Poland 216](#_Toc173834697)

[107. Table S63. Proportions by demographic category for South Africa 218](#_Toc173834698)

[108. Table S64. Proportions by demographic category for Spain 220](#_Toc173834699)

[109. Table S65. Proportions by demographic category for Sweden 222](#_Toc173834700)

[110. Table S66. Proportions by demographic category for Tanzania 224](#_Toc173834701)

[111. Table S67. Proportions by demographic category for Türkiye 226](#_Toc173834702)

[112. Table S68. Proportions by demographic category for United Kingdom 228](#_Toc173834703)

[113. Table S69. Proportions by demographic category for United States 230](#_Toc173834704)

[114. Table S70. Population weighted meta-analysis of proportion (prevalence) of daily cigarette smoking by demographic category 232](#_Toc173834705)

[115. Figures S1-S34. Forest plots of Mean for Demographic Categories 233](#_Toc173834706)

[116. Figures S35-S68. Forest plots of Intensity for Demographic Categories 250](#_Toc173834707)

[117. Figures S69-S102. Forest plots of Proportion for Demographic Categories 267](#_Toc173834708)

# Table S1a. Nationally representative descriptive statistics for Argentina

| **Characteristic** | **N = 6,724**^1^ |
| --- | --- |
| **Age group** |  |
| 18-24 | 1,108 (16%) |
| 25-34 | 1,527 (23%) |
| 35-44 | 1,279 (19%) |
| 45-54 | 1,074 (16%) |
| 55-64 | 871 (13%) |
| 65-74 | 590 (8.8%) |
| 75-84 | 247 (3.7%) |
| 85 or older | 29 (0.4%) |
| (Missing) | 0 (0%) |
| **Gender** |  |
| Male | 3,143 (47%) |
| Female | 3,542 (53%) |
| Other | 21 (0.3%) |
| (Missing) | 18 (0.3%) |
| **Marital status** |  |
| Married | 1,565 (23%) |
| Separated | 455 (6.8%) |
| Divorced | 321 (4.8%) |
| Widowed | 401 (6.0%) |
| Single, never married | 2,381 (35%) |
| Domestic Partner | 1,514 (23%) |
| (Missing) | 88 (1.3%) |
| **Employment** |  |
| Employed for an employer | 2,440 (36%) |
| Self-employed | 1,748 (26%) |
| Retired | 773 (11%) |
| Student | 354 (5.3%) |
| Homemaker | 639 (9.5%) |
| Unemployed and looking for a job | 569 (8.5%) |
| None of these/Other | 179 (2.7%) |
| (Missing) | 22 (0.3%) |
| **Religious service attendance** |  |
| More than 1/week | 532 (7.9%) |
| 1/week | 773 (12%) |
| 1-3/month | 461 (6.8%) |
| A few times a year | 1,949 (29%) |
| Never | 2,982 (44%) |
| (Missing) | 27 (0.4%) |
| **Education** |  |
| Up to 8 years | 2,263 (34%) |
| 9-15 years | 3,823 (57%) |
| 16+ years | 635 (9.4%) |
| (Missing) | 3 (<0.1%) |
| **Immigration** |  |
| Born in this country | 6,346 (94%) |
| Born in another country | 348 (5.2%) |
| (Missing) | 29 (0.4%) |
| **Religious affiliation** |  |
| Christianity | 4,992 (74%) |
| Islam | 9 (0.1%) |
| Hinduism | 6 (<0.1%) |
| Buddhism | 35 (0.5%) |
| Judaism | 40 (0.6%) |
| Sikhism | 0 (<0.1%) |
| Baha'i | 0 (0%) |
| Jainism | 0 (0%) |
| Shinto | 0 (0%) |
| Taoism | 2 (<0.1%) |
| Confucianism | 0 (<0.1%) |
| Primal, Animist, or Folk religion | 19 (0.3%) |
| Spiritism | 0 (0%) |
| Umbanda, Candomble, and other African-derived religions | 0 (0%) |
| Chinese folk/traditional religion | 0 (0%) |
| Some other religion | 156 (2.3%) |
| No religion/Atheist/Agnostic | 1,352 (20%) |
| (Missing) | 111 (1.7%) |
| **Race/Ethnicity** |  |
| Asian | 43 (0.6%) |
| Black | 95 (1.4%) |
| Indigenous | 129 (1.9%) |
| Mestizo(a) | 1,801 (27%) |
| Mullato(a) | 75 (1.1%) |
| Other | 104 (1.5%) |
| White | 3,406 (51%) |
| (Missing) | 1,070 (16%) |
| ^1^n (%) | |

# Table S1b. Means by demographic category for Argentina

| Variable | Category | Mean | 95% CI | SE | Global p-value |
| --- | --- | --- | --- | --- | --- |
| Age group | 18-24 | 2.68 | (2.05, 3.30) | 0.32 | < .001 |
|  | 25-34 | 4.17 | (3.53, 4.82) | 0.33 |  |
|  | 35-44 | 5.01 | (4.25, 5.78) | 0.39 |  |
|  | 45-54 | 4.06 | (3.45, 4.67) | 0.31 |  |
|  | 55-64 | 4.08 | (3.29, 4.87) | 0.40 |  |
|  | 65-74 | 3.66 | (2.71, 4.61) | 0.48 |  |
|  | 75-84 | 1.08 | (0.25, 1.90) | 0.42 |  |
|  | 85 or older | 1.09 | (0.00, 2.78) | 0.81 |  |
| Gender | Male | 4.55 | (4.08, 5.02) | 0.24 | < .001 |
|  | Female | 3.30 | (2.96, 3.64) | 0.17 |  |
|  | Other | 2.35 | (0.00, 5.77) | 1.61 |  |
| Marital status | Married | 2.88 | (2.38, 3.37) | 0.25 | < .001 |
|  | Separated | 5.50 | (4.11, 6.90) | 0.71 |  |
|  | Divorced | 5.19 | (3.80, 6.58) | 0.71 |  |
|  | Widowed | 3.18 | (2.14, 4.22) | 0.53 |  |
|  | Never | 3.92 | (3.43, 4.41) | 0.25 |  |
|  | Domestic Partner | 4.29 | (3.66, 4.93) | 0.32 |  |
| Employment | Employed for an employer | 4.22 | (3.73, 4.72) | 0.25 | < .001 |
|  | Self-employed | 4.07 | (3.45, 4.68) | 0.31 |  |
|  | Retired | 2.84 | (2.15, 3.52) | 0.35 |  |
|  | Student | 1.62 | (1.06, 2.17) | 0.28 |  |
|  | Homemaker | 3.58 | (2.79, 4.37) | 0.40 |  |
|  | Unemployed and looking for a job | 4.65 | (3.50, 5.79) | 0.58 |  |
|  | None of these/Other | 5.14 | (3.05, 7.23) | 1.06 |  |
| Religious service attendance | More than 1/week | 2.10 | (1.24, 2.95) | 0.44 | < .001 |
|  | 1/week | 2.43 | (1.85, 3.00) | 0.29 |  |
|  | 1-3/month | 3.96 | (2.86, 5.06) | 0.56 |  |
|  | A few times a year | 3.74 | (3.19, 4.29) | 0.28 |  |
|  | Never | 4.67 | (4.21, 5.12) | 0.23 |  |
| Education | Up to 8 years | 4.78 | (4.10, 5.45) | 0.35 | < .001 |
|  | 9-15 years | 3.68 | (3.39, 3.97) | 0.15 |  |
|  | 16+ years | 1.93 | (1.41, 2.44) | 0.26 |  |
| Immigration status | Born in this country | 3.97 | (3.67, 4.27) | 0.15 | < .001 |
|  | Born in another country | 2.37 | (1.47, 3.26) | 0.45 |  |
| Religious affiliation | Christianity | 3.73 | (3.39, 4.06) | 0.17 | < .001 |
|  | Islam | 0.58 | (0.00, 1.79) | 0.53 |  |
|  | Hinduism | 3.29 | * | * |  |
|  | Buddhism | 3.69 | (1.47, 5.91) | 1.06 |  |
|  | Judaism | 2.28 | (0.51, 4.05) | 0.86 |  |
|  | Sikhism | 0.00 | * | * |  |
|  | Taoism | 9.21 | * | * |  |
|  | Confucianism | 0.00 | * | * |  |
|  | Primal, Animist, or Folk religion | 5.94 | (0.00, 16.01) | 4.12 |  |
|  | Some other religion | 5.48 | (3.29, 7.68) | 1.11 |  |
|  | No religion/Atheist/Agnostic | 4.31 | (3.67, 4.95) | 0.33 |  |
| Race/Ethnicity | Asian | 4.48 | (0.58, 8.38) | 1.81 | 0.761 |
|  | Black | 4.70 | (2.14, 7.26) | 1.27 |  |
|  | Indigenous | 4.45 | (2.07, 6.83) | 1.18 |  |
|  | Mestizo(a) | 3.75 | (3.27, 4.23) | 0.24 |  |
|  | Mullato(a) | 4.05 | (0.33, 7.76) | 1.85 |  |
|  | White | 3.85 | (3.45, 4.24) | 0.20 |  |
|  | Other | 5.55 | (2.47, 8.63) | 1.55 |  |

# Table S2a. Nationally representative descriptive statistics for Australia

| **Characteristic** | **N = 3,844**^1^ |
| --- | --- |
| **Age group** |  |
| 18-24 | 345 (9.0%) |
| 25-34 | 586 (15%) |
| 35-44 | 681 (18%) |
| 45-54 | 650 (17%) |
| 55-64 | 652 (17%) |
| 65-74 | 522 (14%) |
| 75-84 | 359 (9.3%) |
| 85 or older | 48 (1.3%) |
| (Missing) | 2 (<0.1%) |
| **Gender** |  |
| Male | 1,861 (48%) |
| Female | 1,941 (50%) |
| Other | 36 (0.9%) |
| (Missing) | 6 (0.2%) |
| **Marital status** |  |
| Married | 1,797 (47%) |
| Separated | 158 (4.1%) |
| Divorced | 332 (8.6%) |
| Widowed | 215 (5.6%) |
| Single, never married | 855 (22%) |
| Domestic Partner | 450 (12%) |
| (Missing) | 38 (1.0%) |
| **Employment** |  |
| Employed for an employer | 1,881 (49%) |
| Self-employed | 380 (9.9%) |
| Retired | 912 (24%) |
| Student | 190 (5.0%) |
| Homemaker | 137 (3.6%) |
| Unemployed and looking for a job | 134 (3.5%) |
| None of these/Other | 206 (5.4%) |
| (Missing) | 4 (0.1%) |
| **Religious service attendance** |  |
| More than 1/week | 162 (4.2%) |
| 1/week | 299 (7.8%) |
| 1-3/month | 135 (3.5%) |
| A few times a year | 656 (17%) |
| Never | 2,584 (67%) |
| (Missing) | 7 (0.2%) |
| **Education** |  |
| Up to 8 years | 70 (1.8%) |
| 9-15 years | 2,434 (63%) |
| 16+ years | 1,330 (35%) |
| (Missing) | 10 (0.3%) |
| **Immigration** |  |
| Born in this country | 2,953 (77%) |
| Born in another country | 885 (23%) |
| (Missing) | 6 (0.2%) |
| **Religious affiliation** |  |
| Christianity | 1,592 (41%) |
| Islam | 45 (1.2%) |
| Hinduism | 31 (0.8%) |
| Buddhism | 36 (0.9%) |
| Judaism | 26 (0.7%) |
| Sikhism | 8 (0.2%) |
| Baha'i | 7 (0.2%) |
| Jainism | 0 (0%) |
| Shinto | 0 (0%) |
| Taoism | 5 (0.1%) |
| Confucianism | 0 (0%) |
| Primal, Animist, or Folk religion | 23 (0.6%) |
| Spiritism | 0 (0%) |
| Umbanda, Candomble, and other African-derived religions | 0 (0%) |
| Chinese folk/traditional religion | 0 (0%) |
| Some other religion | 39 (1.0%) |
| No religion/Atheist/Agnostic | 2,020 (53%) |
| (Missing) | 15 (0.4%) |
| **Race/Ethnicity** |  |
| Aboriginal | 53 (1.4%) |
| Australian | 1,946 (51%) |
| Australian British/European | 1,047 (27%) |
| Chinese | 75 (1.9%) |
| Indian | 58 (1.5%) |
| Japanese | 1 (<0.1%) |
| Malay | 11 (0.3%) |
| New Zealander | 91 (2.4%) |
| Other | 163 (4.2%) |
| Other European | 357 (9.3%) |
| Russian | 7 (0.2%) |
| Samoan | 4 (0.1%) |
| Sinhalese | 1 (<0.1%) |
| Spanish | 2 (<0.1%) |
| Sri Lankan Moor | 1 (<0.1%) |
| Sri Lankan Tamil | 7 (0.2%) |
| Vietnamese | 7 (0.2%) |
| (Missing) | 14 (0.4%) |
| ^1^n (%) | |

# Table S2b. Means by demographic category for Australia

| Variable | Category | Mean | 95% CI | SE | Global p-value |
| --- | --- | --- | --- | --- | --- |
| Age group | 18-24 | 0.21 | (0.06, 0.36) | 0.08 | < .001 |
|  | 25-34 | 1.05 | (0.59, 1.50) | 0.23 |  |
|  | 35-44 | 1.99 | (1.30, 2.68) | 0.35 |  |
|  | 45-54 | 1.97 | (1.40, 2.55) | 0.29 |  |
|  | 55-64 | 1.63 | (1.20, 2.06) | 0.22 |  |
|  | 65-74 | 0.85 | (0.50, 1.21) | 0.18 |  |
|  | 75-84 | 0.52 | (0.03, 1.01) | 0.25 |  |
|  | 85 or older | 0.77 | (0.00, 1.89) | 0.53 |  |
| Gender | Male | 1.59 | (1.26, 1.93) | 0.17 | 0.024 |
|  | Female | 1.06 | (0.83, 1.29) | 0.12 |  |
|  | Other | 0.78 | (0.00, 1.86) | 0.53 |  |
| Marital status | Married | 0.79 | (0.57, 1.00) | 0.11 | < .001 |
|  | Separated | 3.26 | (1.74, 4.77) | 0.76 |  |
|  | Divorced | 2.02 | (1.21, 2.82) | 0.41 |  |
|  | Widowed | 1.21 | (0.48, 1.95) | 0.37 |  |
|  | Never | 1.56 | (1.08, 2.04) | 0.25 |  |
|  | Domestic Partner | 1.81 | (1.10, 2.52) | 0.36 |  |
| Employment | Employed for an employer | 1.27 | (1.00, 1.55) | 0.14 | < .001 |
|  | Self-employed | 0.70 | (0.32, 1.09) | 0.20 |  |
|  | Retired | 1.06 | (0.70, 1.42) | 0.18 |  |
|  | Student | 0.30 | (0.00, 0.63) | 0.17 |  |
|  | Homemaker | 2.52 | (0.82, 4.23) | 0.86 |  |
|  | Unemployed and looking for a job | 3.69 | (1.86, 5.52) | 0.93 |  |
|  | None of these/Other | 2.55 | (1.28, 3.83) | 0.65 |  |
| Religious service attendance | More than 1/week | 0.17 | (0.00, 0.44) | 0.13 | < .001 |
|  | 1/week | 0.57 | (0.04, 1.11) | 0.27 |  |
|  | 1-3/month | 0.54 | (0.00, 1.17) | 0.32 |  |
|  | A few times a year | 1.08 | (0.64, 1.52) | 0.22 |  |
|  | Never | 1.58 | (1.31, 1.84) | 0.14 |  |
| Education | Up to 8 years | 3.71 | (0.90, 6.53) | 1.41 | < .001 |
|  | 9-15 years | 1.67 | (1.38, 1.96) | 0.15 |  |
|  | 16+ years | 0.54 | (0.38, 0.69) | 0.08 |  |
| Immigration status | Born in this country | 1.41 | (1.18, 1.64) | 0.12 | 0.078 |
|  | Born in another country | 1.01 | (0.62, 1.40) | 0.20 |  |
| Religious affiliation | Christianity | 1.07 | (0.80, 1.33) | 0.14 | < .001 |
|  | Islam | 1.25 | (0.00, 3.37) | 1.04 |  |
|  | Hinduism | 0.00 | * | * |  |
|  | Buddhism | 2.54 | (0.00, 5.70) | 1.52 |  |
|  | Judaism | 0.44 | (0.00, 1.15) | 0.33 |  |
|  | Sikhism | 0.00 | * | * |  |
|  | Baha'i | 0.00 | * | * |  |
|  | Taoism | 1.36 | * | * |  |
|  | Primal, Animist, or Folk religion | 0.31 | (0.00, 0.98) | 0.30 |  |
|  | Some other religion | 2.26 | (0.00, 5.23) | 1.44 |  |
|  | No religion/Atheist/Agnostic | 1.53 | (1.23, 1.83) | 0.15 |  |
| Race/Ethnicity | Aboriginal | 3.39 | (0.26, 6.52) | 1.54 | < .001 |
|  | Australian | 1.37 | (1.09, 1.64) | 0.14 |  |
|  | Australian British/European | 1.13 | (0.81, 1.46) | 0.17 |  |
|  | Chinese | 0.20 | (0.00, 0.44) | 0.12 |  |
|  | Indian | 0.03 | (0.00, 0.11) | 0.04 |  |
|  | Japanese | 0.00 | * | * |  |
|  | Malay | 0.00 | * | * |  |
|  | Sinhalese | 0.00 | * | * |  |
|  | Spanish | 1.01 | * | * |  |
|  | Sri Lankan Moor | 0.00 | * | * |  |
|  | Sri Lankan Tamil | 0.00 | * | * |  |
|  | Vietnamese | 1.48 | * | * |  |
|  | Russian | 0.45 | * | * |  |
|  | Samoan | 26.11 | * | * |  |
|  | New Zealander | 2.41 | (0.27, 4.56) | 1.07 |  |
|  | Other European | 1.03 | (0.39, 1.68) | 0.33 |  |
|  | Other | 1.69 | (0.52, 2.86) | 0.59 |  |

# Table S3a. Nationally representative descriptive statistics for Brazil

| **Characteristic** | **N = 13,204**^1^ |
| --- | --- |
| **Age group** |  |
| 18-24 | 1,986 (15%) |
| 25-34 | 2,916 (22%) |
| 35-44 | 2,840 (22%) |
| 45-54 | 2,271 (17%) |
| 55-64 | 1,805 (14%) |
| 65-74 | 1,076 (8.1%) |
| 75-84 | 267 (2.0%) |
| 85 or older | 44 (0.3%) |
| (Missing) | 0 (0%) |
| **Gender** |  |
| Male | 6,320 (48%) |
| Female | 6,820 (52%) |
| Other | 35 (0.3%) |
| (Missing) | 30 (0.2%) |
| **Marital status** |  |
| Married | 4,646 (35%) |
| Separated | 594 (4.5%) |
| Divorced | 865 (6.5%) |
| Widowed | 408 (3.1%) |
| Single, never married | 4,347 (33%) |
| Domestic Partner | 2,081 (16%) |
| (Missing) | 263 (2.0%) |
| **Employment** |  |
| Employed for an employer | 3,756 (28%) |
| Self-employed | 2,918 (22%) |
| Retired | 1,536 (12%) |
| Student | 624 (4.7%) |
| Homemaker | 1,305 (9.9%) |
| Unemployed and looking for a job | 2,419 (18%) |
| None of these/Other | 448 (3.4%) |
| (Missing) | 199 (1.5%) |
| **Religious service attendance** |  |
| More than 1/week | 2,386 (18%) |
| 1/week | 2,272 (17%) |
| 1-3/month | 1,398 (11%) |
| A few times a year | 3,978 (30%) |
| Never | 3,110 (24%) |
| (Missing) | 61 (0.5%) |
| **Education** |  |
| Up to 8 years | 3,139 (24%) |
| 9-15 years | 7,665 (58%) |
| 16+ years | 2,390 (18%) |
| (Missing) | 10 (<0.1%) |
| **Immigration** |  |
| Born in this country | 12,688 (96%) |
| Born in another country | 153 (1.2%) |
| (Missing) | 363 (2.7%) |
| **Religious affiliation** |  |
| Christianity | 9,911 (75%) |
| Islam | 6 (<0.1%) |
| Hinduism | 1 (<0.1%) |
| Buddhism | 37 (0.3%) |
| Judaism | 31 (0.2%) |
| Sikhism | 0 (0%) |
| Baha'i | 2 (<0.1%) |
| Jainism | 2 (<0.1%) |
| Shinto | 1 (<0.1%) |
| Taoism | 1 (<0.1%) |
| Confucianism | 6 (<0.1%) |
| Primal, Animist, or Folk religion | 15 (0.1%) |
| Spiritism | 696 (5.3%) |
| Umbanda, Candomble, and other African-derived religions | 525 (4.0%) |
| Chinese folk/traditional religion | 0 (0%) |
| Some other religion | 144 (1.1%) |
| No religion/Atheist/Agnostic | 1,712 (13%) |
| (Missing) | 113 (0.9%) |
| **Race/Ethnicity** |  |
| Amarela | 238 (1.8%) |
| Branca | 5,169 (39%) |
| Indígena | 131 (1.0%) |
| Other | 61 (0.5%) |
| Parda | 5,125 (39%) |
| Preta | 1,615 (12%) |
| (Missing) | 865 (6.6%) |
| ^1^n (%) | |

# Table S3b. Means by demographic category for Brazil

| Variable | Category | Mean | 95% CI | SE | Global p-value |
| --- | --- | --- | --- | --- | --- |
| Age group | 18-24 | 1.06 | (0.85, 1.28) | 0.11 | < .001 |
|  | 25-34 | 2.32 | (2.04, 2.60) | 0.14 |  |
|  | 35-44 | 2.69 | (2.42, 2.96) | 0.14 |  |
|  | 45-54 | 2.82 | (2.43, 3.20) | 0.20 |  |
|  | 55-64 | 3.16 | (2.68, 3.64) | 0.24 |  |
|  | 65-74 | 2.68 | (1.92, 3.44) | 0.39 |  |
|  | 75-84 | 0.24 | (0.00, 0.53) | 0.14 |  |
|  | 85 or older | 1.32 | (0.00, 3.11) | 0.88 |  |
| Gender | Male | 2.94 | (2.71, 3.17) | 0.12 | < .001 |
|  | Female | 1.89 | (1.71, 2.08) | 0.09 |  |
|  | Other | 1.76 | (0.39, 3.13) | 0.67 |  |
| Marital status | Married | 1.73 | (1.51, 1.95) | 0.11 | < .001 |
|  | Separated | 4.22 | (3.23, 5.22) | 0.51 |  |
|  | Divorced | 3.52 | (2.83, 4.21) | 0.35 |  |
|  | Widowed | 2.17 | (1.21, 3.14) | 0.49 |  |
|  | Never | 2.45 | (2.20, 2.71) | 0.13 |  |
|  | Domestic Partner | 2.81 | (2.47, 3.14) | 0.17 |  |
| Employment | Employed for an employer | 2.01 | (1.79, 2.22) | 0.11 | < .001 |
|  | Self-employed | 2.91 | (2.60, 3.22) | 0.16 |  |
|  | Retired | 2.27 | (1.73, 2.81) | 0.28 |  |
|  | Student | 0.58 | (0.28, 0.87) | 0.15 |  |
|  | Homemaker | 2.50 | (1.96, 3.03) | 0.27 |  |
|  | Unemployed and looking for a job | 2.91 | (2.55, 3.27) | 0.19 |  |
|  | None of these/Other | 2.20 | (1.53, 2.86) | 0.34 |  |
| Religious service attendance | More than 1/week | 1.29 | (1.00, 1.58) | 0.15 | < .001 |
|  | 1/week | 2.12 | (1.75, 2.49) | 0.19 |  |
|  | 1-3/month | 2.40 | (2.01, 2.78) | 0.20 |  |
|  | A few times a year | 2.70 | (2.45, 2.96) | 0.13 |  |
|  | Never | 3.05 | (2.71, 3.39) | 0.17 |  |
| Education | Up to 8 years | 3.29 | (2.92, 3.66) | 0.19 | < .001 |
|  | 9-15 years | 2.31 | (2.14, 2.49) | 0.09 |  |
|  | 16+ years | 1.48 | (1.19, 1.77) | 0.15 |  |
| Immigration status | Born in this country | 2.40 | (2.26, 2.55) | 0.07 | 0.183 |
|  | Born in another country | 1.75 | (0.77, 2.73) | 0.50 |  |
| Religious affiliation | Christianity | 1.95 | (1.79, 2.10) | 0.08 | < .001 |
|  | Islam | 7.93 | * | * |  |
|  | Hinduism | 10.67 | * | * |  |
|  | Buddhism | 3.48 | (0.34, 6.61) | 1.50 |  |
|  | Judaism | 3.48 | (0.57, 6.39) | 1.34 |  |
|  | Baha'i | 0.67 | * | * |  |
|  | Jainism | 6.51 | * | * |  |
|  | Shinto | 21.90 | * | * |  |
|  | Taoism | 0.32 | * | * |  |
|  | Confucianism | 0.00 | * | * |  |
|  | Primal, Animist, or Folk religion | 0.40 | * | * |  |
|  | Spiritism | 4.27 | (3.35, 5.20) | 0.47 |  |
|  | Umbanda, Candomble, and other African-derived religions | 5.37 | (4.41, 6.33) | 0.49 |  |
|  | Some other religion | 4.77 | (2.83, 6.71) | 0.98 |  |
|  | No religion/Atheist/Agnostic | 3.07 | (2.64, 3.50) | 0.22 |  |
| Race/Ethnicity | Branca | 2.64 | (2.40, 2.88) | 0.12 | 0.014 |
|  | Preta | 2.06 | (1.73, 2.38) | 0.17 |  |
|  | Parda | 2.20 | (1.98, 2.42) | 0.11 |  |
|  | Amarela | 2.81 | (1.73, 3.88) | 0.55 |  |
|  | Indígena | 3.64 | (1.18, 6.10) | 1.25 |  |
|  | Other | 3.38 | (1.48, 5.28) | 0.95 |  |

# Table S4a. Nationally representative descriptive statistics for Egypt

| **Characteristic** | **N = 4,729**^1^ |
| --- | --- |
| **Age group** |  |
| 18-24 | 960 (20%) |
| 25-34 | 1,296 (27%) |
| 35-44 | 1,016 (21%) |
| 45-54 | 706 (15%) |
| 55-64 | 579 (12%) |
| 65-74 | 156 (3.3%) |
| 75-84 | 15 (0.3%) |
| 85 or older | 2 (<0.1%) |
| (Missing) | 0 (0%) |
| **Gender** |  |
| Male | 2,394 (51%) |
| Female | 2,334 (49%) |
| Other | 0 (0%) |
| (Missing) | 0 (<0.1%) |
| **Marital status** |  |
| Married | 3,387 (72%) |
| Separated | 39 (0.8%) |
| Divorced | 101 (2.1%) |
| Widowed | 238 (5.0%) |
| Single, never married | 947 (20%) |
| Domestic Partner | 0 (0%) |
| (Missing) | 17 (0.4%) |
| **Employment** |  |
| Employed for an employer | 1,267 (27%) |
| Self-employed | 892 (19%) |
| Retired | 253 (5.4%) |
| Student | 297 (6.3%) |
| Homemaker | 1,772 (37%) |
| Unemployed and looking for a job | 224 (4.7%) |
| None of these/Other | 21 (0.4%) |
| (Missing) | 3 (<0.1%) |
| **Religious service attendance** |  |
| More than 1/week | 839 (18%) |
| 1/week | 960 (20%) |
| 1-3/month | 368 (7.8%) |
| A few times a year | 458 (9.7%) |
| Never | 2,091 (44%) |
| (Missing) | 12 (0.3%) |
| **Education** |  |
| Up to 8 years | 2,486 (53%) |
| 9-15 years | 1,599 (34%) |
| 16+ years | 643 (14%) |
| (Missing) | 1 (<0.1%) |
| **Immigration** |  |
| Born in this country | 4,713 (100%) |
| Born in another country | 16 (0.3%) |
| (Missing) | 1 (<0.1%) |
| **Religious affiliation** |  |
| Christianity | 120 (2.5%) |
| Islam | 4,607 (97%) |
| Hinduism | 0 (0%) |
| Buddhism | 0 (0%) |
| Judaism | 0 (0%) |
| Sikhism | 0 (0%) |
| Baha'i | 0 (0%) |
| Jainism | 0 (0%) |
| Shinto | 0 (0%) |
| Taoism | 0 (<0.1%) |
| Confucianism | 0 (0%) |
| Primal, Animist, or Folk religion | 0 (0%) |
| Spiritism | 0 (0%) |
| Umbanda, Candomble, and other African-derived religions | 0 (0%) |
| Chinese folk/traditional religion | 0 (0%) |
| Some other religion | 0 (0%) |
| No religion/Atheist/Agnostic | 0 (0%) |
| (Missing) | 1 (<0.1%) |
| **Race/Ethnicity** |  |
| Arab | 4,585 (97%) |
| Bedouin Arab | 4 (<0.1%) |
| Greek | 1 (<0.1%) |
| Nubian | 27 (0.6%) |
| Turkish | 9 (0.2%) |
| (Missing) | 102 (2.2%) |
| ^1^n (%) | |

# Table S4b. Means by demographic category for Egypt

| Variable | Category | Mean | 95% CI | SE | Global p-value |
| --- | --- | --- | --- | --- | --- |
| Age group | 18-24 | 2.83 | (2.12, 3.54) | 0.36 | < .001 |
|  | 25-34 | 3.73 | (3.14, 4.32) | 0.30 |  |
|  | 35-44 | 3.08 | (2.61, 3.55) | 0.24 |  |
|  | 45-54 | 3.01 | (2.32, 3.70) | 0.35 |  |
|  | 55-64 | 4.37 | (3.48, 5.27) | 0.45 |  |
|  | 65-74 | 2.64 | (1.37, 3.92) | 0.64 |  |
|  | 75-84 | 0.63 | (0.00, 2.24) | 0.63 |  |
|  | 85 or older | 0.00 | * | * |  |
| Gender | Male | 6.53 | (5.99, 7.07) | 0.28 | < .001 |
|  | Female | 0.05 | (0.01, 0.09) | 0.02 |  |
| Marital status | Married | 3.16 | (2.81, 3.52) | 0.18 | < .001 |
|  | Separated | 2.42 | (0.32, 4.52) | 1.03 |  |
|  | Divorced | 3.64 | (1.19, 6.08) | 1.23 |  |
|  | Widowed | 1.22 | (0.53, 1.92) | 0.35 |  |
|  | Never | 4.47 | (3.73, 5.21) | 0.38 |  |
| Employment | Employed for an employer | 5.91 | (5.21, 6.62) | 0.36 | < .001 |
|  | Self-employed | 5.83 | (5.00, 6.66) | 0.42 |  |
|  | Retired | 3.79 | (2.55, 5.03) | 0.63 |  |
|  | Student | 1.87 | (1.08, 2.65) | 0.40 |  |
|  | Homemaker | 0.09 | (0.03, 0.15) | 0.03 |  |
|  | Unemployed and looking for a job | 5.85 | (3.97, 7.73) | 0.95 |  |
|  | None of these/Other | 3.60 | (0.00, 7.69) | 1.86 |  |
| Religious service attendance | More than 1/week | 3.76 | (3.08, 4.43) | 0.34 | < .001 |
|  | 1/week | 4.14 | (3.54, 4.73) | 0.30 |  |
|  | 1-3/month | 3.55 | (2.74, 4.35) | 0.41 |  |
|  | A few times a year | 4.29 | (3.29, 5.29) | 0.51 |  |
|  | Never | 2.55 | (2.13, 2.96) | 0.21 |  |
| Education | Up to 8 years | 3.64 | (3.19, 4.09) | 0.23 | < .001 |
|  | 9-15 years | 3.34 | (2.89, 3.78) | 0.23 |  |
|  | 16+ years | 2.12 | (1.42, 2.82) | 0.36 |  |
| Immigration status | Born in this country | 3.33 | (3.01, 3.65) | 0.16 | 0.469 |
|  | Born in another country | 3.06 | (0.00, 9.44) | 2.54 |  |
| Religious affiliation | Christianity | 2.69 | (0.53, 4.84) | 1.09 | < .001 |
|  | Islam | 3.35 | (3.03, 3.67) | 0.16 |  |
|  | Taoism | 0.00 | * | * |  |
| Race/Ethnicity | Arab | 3.33 | (3.01, 3.65) | 0.16 | < .001 |
|  | Turkish | 0.38 | (0.00, 1.53) | 0.33 |  |
|  | Greek | 0.00 | * | * |  |
|  | Bedouin Arab | 2.51 | * | * |  |
|  | Nubian | 4.45 | (3.28, 5.61) | 0.56 |  |

# Table S5a. Nationally representative descriptive statistics for Germany

| **Characteristic** | **N = 9,506**^1^ |
| --- | --- |
| **Age group** |  |
| 18-24 | 829 (8.7%) |
| 25-34 | 1,464 (15%) |
| 35-44 | 1,446 (15%) |
| 45-54 | 1,590 (17%) |
| 55-64 | 1,717 (18%) |
| 65-74 | 1,960 (21%) |
| 75-84 | 453 (4.8%) |
| 85 or older | 47 (0.5%) |
| (Missing) | 0 (0%) |
| **Gender** |  |
| Male | 4,641 (49%) |
| Female | 4,843 (51%) |
| Other | 11 (0.1%) |
| (Missing) | 11 (0.1%) |
| **Marital status** |  |
| Married | 4,784 (50%) |
| Separated | 219 (2.3%) |
| Divorced | 767 (8.1%) |
| Widowed | 409 (4.3%) |
| Single, never married | 2,627 (28%) |
| Domestic Partner | 619 (6.5%) |
| (Missing) | 81 (0.9%) |
| **Employment** |  |
| Employed for an employer | 4,950 (52%) |
| Self-employed | 712 (7.5%) |
| Retired | 2,480 (26%) |
| Student | 605 (6.4%) |
| Homemaker | 251 (2.6%) |
| Unemployed and looking for a job | 288 (3.0%) |
| None of these/Other | 204 (2.1%) |
| (Missing) | 14 (0.2%) |
| **Religious service attendance** |  |
| More than 1/week | 285 (3.0%) |
| 1/week | 424 (4.5%) |
| 1-3/month | 550 (5.8%) |
| A few times a year | 2,362 (25%) |
| Never | 5,876 (62%) |
| (Missing) | 9 (<0.1%) |
| **Education** |  |
| Up to 8 years | 235 (2.5%) |
| 9-15 years | 6,094 (64%) |
| 16+ years | 3,164 (33%) |
| (Missing) | 13 (0.1%) |
| **Immigration** |  |
| Born in this country | 8,722 (92%) |
| Born in another country | 744 (7.8%) |
| (Missing) | 40 (0.4%) |
| **Religious affiliation** |  |
| Christianity | 5,052 (53%) |
| Islam | 351 (3.7%) |
| Hinduism | 12 (0.1%) |
| Buddhism | 51 (0.5%) |
| Judaism | 19 (0.2%) |
| Sikhism | 5 (<0.1%) |
| Baha'i | 3 (<0.1%) |
| Jainism | 0 (0%) |
| Shinto | 2 (<0.1%) |
| Taoism | 0 (<0.1%) |
| Confucianism | 4 (<0.1%) |
| Primal, Animist, or Folk religion | 34 (0.4%) |
| Spiritism | 0 (0%) |
| Umbanda, Candomble, and other African-derived religions | 0 (0%) |
| Chinese folk/traditional religion | 0 (0%) |
| Some other religion | 60 (0.6%) |
| No religion/Atheist/Agnostic | 3,815 (40%) |
| (Missing) | 99 (1.0%) |
| ^1^n (%) | |

# Table S5b. Means by demographic category for Germany

| Variable | Category | Mean | 95% CI | SE | Global p-value |
| --- | --- | --- | --- | --- | --- |
| Age group | 18-24 | 2.06 | (1.53, 2.58) | 0.27 | < .001 |
|  | 25-34 | 2.99 | (2.58, 3.39) | 0.21 |  |
|  | 35-44 | 3.63 | (3.16, 4.09) | 0.24 |  |
|  | 45-54 | 4.65 | (4.09, 5.20) | 0.28 |  |
|  | 55-64 | 4.82 | (4.26, 5.39) | 0.29 |  |
|  | 65-74 | 3.48 | (3.00, 3.96) | 0.25 |  |
|  | 75-84 | 1.99 | (1.41, 2.57) | 0.29 |  |
|  | 85 or older | 0.48 | (0.00, 1.35) | 0.43 |  |
| Gender | Male | 3.91 | (3.61, 4.21) | 0.15 | 0.018 |
|  | Female | 3.41 | (3.13, 3.69) | 0.14 |  |
|  | Other | 1.90 | (0.00, 4.79) | 1.16 |  |
| Marital status | Married | 3.63 | (3.32, 3.93) | 0.15 | 0.007 |
|  | Separated | 4.17 | (2.85, 5.49) | 0.67 |  |
|  | Divorced | 4.81 | (4.14, 5.49) | 0.34 |  |
|  | Widowed | 3.46 | (2.55, 4.38) | 0.46 |  |
|  | Never | 3.48 | (3.11, 3.85) | 0.19 |  |
|  | Domestic Partner | 3.11 | (2.47, 3.76) | 0.33 |  |
| Employment | Employed for an employer | 3.98 | (3.68, 4.29) | 0.16 | < .001 |
|  | Self-employed | 3.70 | (2.92, 4.48) | 0.40 |  |
|  | Retired | 3.15 | (2.80, 3.50) | 0.18 |  |
|  | Student | 1.14 | (0.75, 1.52) | 0.20 |  |
|  | Homemaker | 5.07 | (3.66, 6.49) | 0.72 |  |
|  | Unemployed and looking for a job | 6.09 | (4.88, 7.31) | 0.62 |  |
|  | None of these/Other | 3.92 | (2.78, 5.07) | 0.58 |  |
| Religious service attendance | More than 1/week | 3.33 | (2.27, 4.39) | 0.54 | 0.001 |
|  | 1/week | 3.06 | (2.27, 3.84) | 0.40 |  |
|  | 1-3/month | 4.73 | (3.48, 5.98) | 0.63 |  |
|  | A few times a year | 3.05 | (2.69, 3.41) | 0.18 |  |
|  | Never | 3.85 | (3.59, 4.11) | 0.13 |  |
| Education | Up to 8 years | 4.27 | (3.09, 5.44) | 0.60 | < .001 |
|  | 9-15 years | 4.33 | (4.06, 4.60) | 0.14 |  |
|  | 16+ years | 2.31 | (2.02, 2.59) | 0.14 |  |
| Immigration status | Born in this country | 3.72 | (3.50, 3.93) | 0.11 | 0.010 |
|  | Born in another country | 2.89 | (2.30, 3.48) | 0.30 |  |
| Religious affiliation | Christianity | 3.64 | (3.38, 3.89) | 0.13 | < .001 |
|  | Islam | 3.38 | (2.20, 4.55) | 0.60 |  |
|  | Hinduism | 4.01 | * | * |  |
|  | Buddhism | 4.09 | (0.93, 7.25) | 1.56 |  |
|  | Judaism | 2.53 | (0.00, 6.17) | 1.38 |  |
|  | Sikhism | 2.79 | * | * |  |
|  | Baha'i | 3.71 | * | * |  |
|  | Shinto | 0.00 | * | * |  |
|  | Taoism | 25.00 | * | * |  |
|  | Confucianism | 0.00 | * | * |  |
|  | Primal, Animist, or Folk religion | 2.48 | (0.00, 4.97) | 1.17 |  |
|  | Some other religion | 6.06 | (2.21, 9.92) | 1.91 |  |
|  | No religion/Atheist/Agnostic | 3.68 | (3.33, 4.02) | 0.18 |  |

# Table S6a. Nationally representative descriptive statistics for Hong Kong

| **Characteristic** | **N = 3,012**^1^ |
| --- | --- |
| **Age group** |  |
| 18-24 | 217 (7.2%) |
| 25-34 | 464 (15%) |
| 35-44 | 542 (18%) |
| 45-54 | 611 (20%) |
| 55-64 | 644 (21%) |
| 65-74 | 492 (16%) |
| 75-84 | 28 (0.9%) |
| 85 or older | 15 (0.5%) |
| (Missing) | 0 (0%) |
| **Gender** |  |
| Male | 1,390 (46%) |
| Female | 1,620 (54%) |
| Other | 2 (<0.1%) |
| (Missing) | 0 (0%) |
| **Marital status** |  |
| Married | 2,080 (69%) |
| Separated | 21 (0.7%) |
| Divorced | 105 (3.5%) |
| Widowed | 45 (1.5%) |
| Single, never married | 723 (24%) |
| Domestic Partner | 37 (1.2%) |
| (Missing) | 1 (<0.1%) |
| **Employment** |  |
| Employed for an employer | 2,056 (68%) |
| Self-employed | 245 (8.1%) |
| Retired | 423 (14%) |
| Student | 55 (1.8%) |
| Homemaker | 114 (3.8%) |
| Unemployed and looking for a job | 62 (2.0%) |
| None of these/Other | 39 (1.3%) |
| (Missing) | 18 (0.6%) |
| **Religious service attendance** |  |
| More than 1/week | 237 (7.9%) |
| 1/week | 567 (19%) |
| 1-3/month | 332 (11%) |
| A few times a year | 543 (18%) |
| Never | 1,332 (44%) |
| (Missing) | 1 (<0.1%) |
| **Education** |  |
| Up to 8 years | 433 (14%) |
| 9-15 years | 2,031 (67%) |
| 16+ years | 547 (18%) |
| (Missing) | 0 (0%) |
| **Immigration** |  |
| Born in this country | 2,637 (88%) |
| Born in another country | 321 (11%) |
| (Missing) | 53 (1.8%) |
| **Religious affiliation** |  |
| Christianity | 757 (25%) |
| Islam | 86 (2.8%) |
| Hinduism | 20 (0.7%) |
| Buddhism | 349 (12%) |
| Judaism | 10 (0.3%) |
| Sikhism | 2 (<0.1%) |
| Baha'i | 3 (<0.1%) |
| Jainism | 1 (<0.1%) |
| Shinto | 19 (0.6%) |
| Taoism | 97 (3.2%) |
| Confucianism | 11 (0.4%) |
| Primal, Animist, or Folk religion | 27 (0.9%) |
| Spiritism | 0 (0%) |
| Umbanda, Candomble, and other African-derived religions | 0 (0%) |
| Chinese folk/traditional religion | 106 (3.5%) |
| Some other religion | 4 (0.1%) |
| No religion/Atheist/Agnostic | 1,518 (50%) |
| (Missing) | 5 (0.2%) |
| **Race/Ethnicity** |  |
| Chinese (Cantonese) | 1,930 (64%) |
| Chinese (Chaoshan) | 201 (6.7%) |
| Chinese (Fujianese) | 117 (3.9%) |
| Chinese (Hakka) | 121 (4.0%) |
| Chinese (Other ethnicity) | 264 (8.8%) |
| Chinese (Shanghainese) | 89 (2.9%) |
| East Asian (Korean, Japanese) | 10 (0.3%) |
| Other | 4 (0.1%) |
| South Asian (Indian, Nepalese, Pakistani) | 17 (0.6%) |
| Southeast Asian (Filipino, Indonesian, Thailand) | 46 (1.5%) |
| Taiwanese | 14 (0.4%) |
| White | 15 (0.5%) |
| (Missing) | 184 (6.1%) |
| ^1^n (%) | |

# Table S6b. Means by demographic category for Hong Kong

| Variable | Category | Mean | 95% CI | SE | Global p-value |
| --- | --- | --- | --- | --- | --- |
| Age group | 18-24 | 2.08 | (1.63, 2.53) | 0.23 | < .001 |
|  | 25-34 | 1.93 | (1.37, 2.48) | 0.28 |  |
|  | 35-44 | 2.03 | (1.62, 2.44) | 0.21 |  |
|  | 45-54 | 3.36 | (2.68, 4.05) | 0.35 |  |
|  | 55-64 | 1.96 | (1.68, 2.24) | 0.14 |  |
|  | 65-74 | 0.59 | (0.09, 1.08) | 0.25 |  |
|  | 75-84 | 0.00 | * | * |  |
|  | 85 or older | 0.00 | * | * |  |
| Gender | Male | 2.37 | (2.06, 2.68) | 0.16 | < .001 |
|  | Female | 1.70 | (1.38, 2.01) | 0.16 |  |
|  | Other | 0.00 | * | * |  |
| Marital status | Married | 2.25 | (1.98, 2.52) | 0.14 | < .001 |
|  | Separated | 1.32 | (0.00, 4.24) | 1.17 |  |
|  | Divorced | 2.45 | (0.61, 4.29) | 0.93 |  |
|  | Widowed | 0.70 | (0.00, 1.86) | 0.57 |  |
|  | Never | 1.38 | (1.02, 1.75) | 0.19 |  |
|  | Domestic Partner | 1.21 | (0.08, 2.33) | 0.55 |  |
| Employment | Employed for an employer | 2.27 | (2.03, 2.52) | 0.13 | < .001 |
|  | Self-employed | 2.69 | (1.89, 3.48) | 0.40 |  |
|  | Retired | 0.81 | (0.16, 1.46) | 0.33 |  |
|  | Student | 0.50 | (0.00, 1.07) | 0.28 |  |
|  | Homemaker | 1.16 | (0.46, 1.86) | 0.35 |  |
|  | Unemployed and looking for a job | 2.33 | (0.00, 4.86) | 1.26 |  |
|  | None of these/Other | 0.71 | (0.00, 4.78) | 1.65 |  |
| Religious service attendance | More than 1/week | 3.25 | (2.61, 3.89) | 0.33 | < .001 |
|  | 1/week | 2.82 | (2.09, 3.55) | 0.37 |  |
|  | 1-3/month | 3.37 | (2.50, 4.24) | 0.44 |  |
|  | A few times a year | 1.40 | (1.09, 1.72) | 0.16 |  |
|  | Never | 1.35 | (1.10, 1.60) | 0.13 |  |
| Education | Up to 8 years | 3.67 | (2.52, 4.82) | 0.58 | < .001 |
|  | 9-15 years | 1.98 | (1.79, 2.18) | 0.10 |  |
|  | 16+ years | 0.79 | (0.58, 0.99) | 0.10 |  |
| Immigration status | Born in this country | 2.11 | (1.88, 2.35) | 0.12 | 0.001 |
|  | Born in another country | 1.12 | (0.36, 1.88) | 0.38 |  |
| Religious affiliation | Christianity | 2.66 | (2.03, 3.29) | 0.32 | < .001 |
|  | Islam | 3.11 | (0.86, 5.36) | 1.09 |  |
|  | Hinduism | 2.74 | (1.53, 3.94) | 0.39 |  |
|  | Buddhism | 2.51 | (2.02, 3.00) | 0.25 |  |
|  | Judaism | 3.52 | * | * |  |
|  | Sikhism | 5.29 | * | * |  |
|  | Baha'i | 5.29 | * | * |  |
|  | Jainism | 3.00 | * | * |  |
|  | Shinto | 0.53 | (0.00, 2.05) | 0.35 |  |
|  | Taoism | 1.60 | (0.85, 2.35) | 0.38 |  |
|  | Confucianism | 3.38 | * | * |  |
|  | Primal, Animist, or Folk religion | 2.16 | (0.49, 3.83) | 0.74 |  |
|  | Chinese folk/traditional religion | 2.84 | (1.75, 3.93) | 0.55 |  |
|  | Some other religion | 1.76 | * | * |  |
|  | No religion/Atheist/Agnostic | 1.45 | (1.22, 1.67) | 0.11 |  |
| Race/Ethnicity | White | 0.98 | (0.00, 10.49) | 0.96 | < .001 |
|  | Other | 0.00 | * | * |  |
|  | Chinese (Cantonese) | 2.07 | (1.78, 2.35) | 0.15 |  |
|  | Chinese (Chaoshan) | 2.84 | (2.17, 3.50) | 0.34 |  |
|  | Chinese (Fujianese) | 3.04 | (2.12, 3.96) | 0.46 |  |
|  | Chinese (Hakka) | 1.51 | (1.02, 2.01) | 0.25 |  |
|  | Chinese (Shanghainese) | 1.43 | (0.73, 2.14) | 0.35 |  |
|  | Chinese (Other ethnicity) | 1.10 | (0.67, 1.53) | 0.22 |  |
|  | East Asian (Korean, Japanese) | 2.56 | * | * |  |
|  | Southeast Asian (Filipino, Indonesian, Thailand) | 0.20 | (0.00, 0.46) | 0.13 |  |
|  | South Asian (Indian, Nepalese, Pakistani) | 4.67 | (0.00, 16.92) | 3.67 |  |
|  | Taiwanese | 1.81 | * | * |  |

# Table S7a. Nationally representative descriptive statistics for India

| **Characteristic** | **N = 12,765**^1^ |
| --- | --- |
| **Age group** |  |
| 18-24 | 2,543 (20%) |
| 25-34 | 3,260 (26%) |
| 35-44 | 2,699 (21%) |
| 45-54 | 1,893 (15%) |
| 55-64 | 1,524 (12%) |
| 65-74 | 676 (5.3%) |
| 75-84 | 148 (1.2%) |
| 85 or older | 23 (0.2%) |
| (Missing) | 0 (0%) |
| **Gender** |  |
| Male | 6,473 (51%) |
| Female | 6,292 (49%) |
| Other | 0 (0%) |
| (Missing) | 0 (0%) |
| **Marital status** |  |
| Married | 9,848 (77%) |
| Separated | 45 (0.4%) |
| Divorced | 25 (0.2%) |
| Widowed | 445 (3.5%) |
| Single, never married | 2,065 (16%) |
| Domestic Partner | 269 (2.1%) |
| (Missing) | 69 (0.5%) |
| **Employment** |  |
| Employed for an employer | 2,660 (21%) |
| Self-employed | 3,401 (27%) |
| Retired | 286 (2.2%) |
| Student | 532 (4.2%) |
| Homemaker | 4,221 (33%) |
| Unemployed and looking for a job | 902 (7.1%) |
| None of these/Other | 715 (5.6%) |
| (Missing) | 48 (0.4%) |
| **Religious service attendance** |  |
| More than 1/week | 2,875 (23%) |
| 1/week | 3,166 (25%) |
| 1-3/month | 2,740 (21%) |
| A few times a year | 2,090 (16%) |
| Never | 1,823 (14%) |
| (Missing) | 71 (0.6%) |
| **Education** |  |
| Up to 8 years | 11,422 (89%) |
| 9-15 years | 1,194 (9.4%) |
| 16+ years | 145 (1.1%) |
| (Missing) | 4 (<0.1%) |
| **Immigration** |  |
| Born in this country | 12,629 (99%) |
| Born in another country | 110 (0.9%) |
| (Missing) | 26 (0.2%) |
| **Religious affiliation** |  |
| Christianity | 306 (2.4%) |
| Islam | 1,555 (12%) |
| Hinduism | 10,362 (81%) |
| Buddhism | 230 (1.8%) |
| Judaism | 0 (0%) |
| Sikhism | 127 (1.0%) |
| Baha'i | 0 (0%) |
| Jainism | 10 (<0.1%) |
| Shinto | 1 (<0.1%) |
| Taoism | 0 (0%) |
| Confucianism | 0 (0%) |
| Primal, Animist, or Folk religion | 30 (0.2%) |
| Spiritism | 0 (0%) |
| Umbanda, Candomble, and other African-derived religions | 0 (0%) |
| Chinese folk/traditional religion | 0 (0%) |
| Some other religion | 67 (0.5%) |
| No religion/Atheist/Agnostic | 13 (0.1%) |
| (Missing) | 62 (0.5%) |
| **Race/Ethnicity** |  |
| General | 3,538 (28%) |
| Other backward caste | 4,177 (33%) |
| Schedule caste | 3,599 (28%) |
| Schedule tribe | 1,185 (9.3%) |
| (Missing) | 267 (2.1%) |
| ^1^n (%) | |

# Table S7b. Means by demographic category for India

| Variable | Category | Mean | 95% CI | SE | Global p-value |
| --- | --- | --- | --- | --- | --- |
| Age group | 18-24 | 0.38 | (0.14, 0.63) | 0.13 | < .001 |
|  | 25-34 | 0.58 | (0.34, 0.82) | 0.12 |  |
|  | 35-44 | 0.56 | (0.42, 0.71) | 0.07 |  |
|  | 45-54 | 1.08 | (0.78, 1.39) | 0.16 |  |
|  | 55-64 | 0.90 | (0.62, 1.19) | 0.15 |  |
|  | 65-74 | 0.50 | (0.23, 0.76) | 0.13 |  |
|  | 75-84 | 0.95 | (0.06, 1.83) | 0.45 |  |
|  | 85 or older | 0.72 | (0.00, 7.42) | 2.31 |  |
| Gender | Male | 1.18 | (0.98, 1.37) | 0.10 | < .001 |
|  | Female | 0.11 | (0.04, 0.19) | 0.04 |  |
| Marital status | Married | 0.63 | (0.53, 0.72) | 0.05 | < .001 |
|  | Separated | 0.98 | (0.00, 2.31) | 0.66 |  |
|  | Divorced | 0.06 | (0.00, 0.19) | 0.06 |  |
|  | Widowed | 0.75 | (0.12, 1.38) | 0.32 |  |
|  | Never | 0.77 | (0.35, 1.19) | 0.21 |  |
|  | Domestic Partner | 0.51 | (0.11, 0.90) | 0.20 |  |
| Employment | Employed for an employer | 0.72 | (0.55, 0.89) | 0.09 | < .001 |
|  | Self-employed | 1.01 | (0.75, 1.26) | 0.13 |  |
|  | Retired | 1.24 | (0.54, 1.94) | 0.35 |  |
|  | Student | 0.36 | (0.00, 0.78) | 0.20 |  |
|  | Homemaker | 0.18 | (0.07, 0.29) | 0.06 |  |
|  | Unemployed and looking for a job | 0.96 | (0.27, 1.66) | 0.35 |  |
|  | None of these/Other | 1.06 | (0.64, 1.48) | 0.21 |  |
| Religious service attendance | More than 1/week | 0.71 | (0.48, 0.94) | 0.12 | 0.404 |
|  | 1/week | 0.67 | (0.44, 0.90) | 0.12 |  |
|  | 1-3/month | 0.51 | (0.37, 0.65) | 0.07 |  |
|  | A few times a year | 0.70 | (0.49, 0.92) | 0.11 |  |
|  | Never | 0.68 | (0.33, 1.03) | 0.18 |  |
| Education | Up to 8 years | 0.67 | (0.56, 0.79) | 0.06 | < .001 |
|  | 9-15 years | 0.52 | (0.31, 0.73) | 0.11 |  |
|  | 16+ years | 0.16 | (0.00, 0.33) | 0.08 |  |
| Immigration status | Born in this country | 0.64 | (0.53, 0.74) | 0.05 | 0.079 |
|  | Born in another country | 2.24 | (0.43, 4.06) | 0.92 |  |
| Religious affiliation | Christianity | 0.69 | (0.00, 1.46) | 0.39 | < .001 |
|  | Islam | 1.01 | (0.50, 1.51) | 0.26 |  |
|  | Hinduism | 0.60 | (0.51, 0.69) | 0.05 |  |
|  | Buddhism | 0.10 | (0.02, 0.19) | 0.04 |  |
|  | Sikhism | 0.20 | (0.00, 0.41) | 0.11 |  |
|  | Jainism | 0.12 | * | * |  |
|  | Shinto | 0.00 | * | * |  |
|  | Primal, Animist, or Folk religion | 0.13 | (0.00, 0.32) | 0.09 |  |
|  | Some other religion | 0.01 | (0.00, 0.03) | 0.01 |  |
|  | No religion/Atheist/Agnostic | 20.03 | (0.00, 108.45) | 17.07 |  |
| Race/Ethnicity | General | 0.79 | (0.53, 1.04) | 0.13 | 0.283 |
|  | Other backward caste | 0.60 | (0.42, 0.79) | 0.09 |  |
|  | Schedule caste | 0.64 | (0.50, 0.78) | 0.07 |  |
|  | Schedule tribe | 0.44 | (0.20, 0.69) | 0.13 |  |

# Table S8a. Nationally representative descriptive statistics for Indonesia

| **Characteristic** | **N = 6,992**^1^ |
| --- | --- |
| **Age group** |  |
| 18-24 | 1,216 (17%) |
| 25-34 | 1,707 (24%) |
| 35-44 | 1,613 (23%) |
| 45-54 | 1,301 (19%) |
| 55-64 | 910 (13%) |
| 65-74 | 218 (3.1%) |
| 75-84 | 18 (0.3%) |
| 85 or older | 9 (0.1%) |
| (Missing) | 0 (0%) |
| **Gender** |  |
| Male | 3,461 (50%) |
| Female | 3,513 (50%) |
| Other | 7 (<0.1%) |
| (Missing) | 11 (0.2%) |
| **Marital status** |  |
| Married | 4,846 (69%) |
| Separated | 81 (1.2%) |
| Divorced | 196 (2.8%) |
| Widowed | 425 (6.1%) |
| Single, never married | 1,381 (20%) |
| Domestic Partner | 18 (0.3%) |
| (Missing) | 45 (0.6%) |
| **Employment** |  |
| Employed for an employer | 1,323 (19%) |
| Self-employed | 2,187 (31%) |
| Retired | 78 (1.1%) |
| Student | 272 (3.9%) |
| Homemaker | 2,138 (31%) |
| Unemployed and looking for a job | 529 (7.6%) |
| None of these/Other | 448 (6.4%) |
| (Missing) | 18 (0.3%) |
| **Religious service attendance** |  |
| More than 1/week | 2,667 (38%) |
| 1/week | 2,529 (36%) |
| 1-3/month | 786 (11%) |
| A few times a year | 659 (9.4%) |
| Never | 332 (4.8%) |
| (Missing) | 18 (0.3%) |
| **Education** |  |
| Up to 8 years | 3,079 (44%) |
| 9-15 years | 3,491 (50%) |
| 16+ years | 419 (6.0%) |
| (Missing) | 2 (<0.1%) |
| **Immigration** |  |
| Born in this country | 6,958 (100%) |
| Born in another country | 34 (0.5%) |
| (Missing) | 0 (0%) |
| **Religious affiliation** |  |
| Christianity | 504 (7.2%) |
| Islam | 6,406 (92%) |
| Hinduism | 73 (1.0%) |
| Buddhism | 3 (<0.1%) |
| Judaism | 0 (0%) |
| Sikhism | 0 (0%) |
| Baha'i | 0 (0%) |
| Jainism | 0 (0%) |
| Shinto | 0 (0%) |
| Taoism | 1 (<0.1%) |
| Confucianism | 0 (0%) |
| Primal, Animist, or Folk religion | 0 (0%) |
| Spiritism | 0 (0%) |
| Umbanda, Candomble, and other African-derived religions | 0 (0%) |
| Chinese folk/traditional religion | 0 (0%) |
| Some other religion | 1 (<0.1%) |
| No religion/Atheist/Agnostic | 0 (0%) |
| (Missing) | 4 (<0.1%) |
| **Race/Ethnicity** |  |
| Bali | 69 (1.0%) |
| Banjar/Melayu Banjar | 320 (4.6%) |
| Batak | 165 (2.4%) |
| Betawi | 251 (3.6%) |
| Bugis | 243 (3.5%) |
| Jawa | 2,846 (41%) |
| Madura | 262 (3.7%) |
| Makasar | 91 (1.3%) |
| Minangkabau | 273 (3.9%) |
| Other | 1,262 (18%) |
| Sunda/Parahyangan | 1,172 (17%) |
| (Missing) | 38 (0.5%) |
| ^1^n (%) | |

# Table S8b. Means by demographic category for Indonesia

| Variable | Category | Mean | 95% CI | SE | Global p-value |
| --- | --- | --- | --- | --- | --- |
| Age group | 18-24 | 2.87 | (2.41, 3.33) | 0.23 | < .001 |
|  | 25-34 | 4.03 | (3.61, 4.45) | 0.22 |  |
|  | 35-44 | 4.32 | (3.81, 4.83) | 0.26 |  |
|  | 45-54 | 3.94 | (3.49, 4.38) | 0.23 |  |
|  | 55-64 | 3.93 | (3.22, 4.63) | 0.36 |  |
|  | 65-74 | 3.02 | (1.79, 4.25) | 0.62 |  |
|  | 75-84 | 0.00 | * | * |  |
|  | 85 or older | 5.67 | * | * |  |
| Gender | Male | 7.35 | (6.98, 7.72) | 0.19 | < .001 |
|  | Female | 0.36 | (0.27, 0.45) | 0.05 |  |
|  | Other | 0.73 | (0.00, 8.79) | 1.02 |  |
| Marital status | Married | 3.88 | (3.62, 4.15) | 0.14 | < .001 |
|  | Separated | 4.67 | (2.87, 6.46) | 0.90 |  |
|  | Divorced | 2.99 | (2.02, 3.95) | 0.49 |  |
|  | Widowed | 2.44 | (1.68, 3.21) | 0.39 |  |
|  | Never | 4.14 | (3.63, 4.65) | 0.26 |  |
|  | Domestic Partner | 1.71 | (0.00, 4.03) | 1.04 |  |
| Employment | Employed for an employer | 5.48 | (4.91, 6.05) | 0.29 | < .001 |
|  | Self-employed | 5.53 | (5.11, 5.94) | 0.21 |  |
|  | Retired | 3.44 | (1.89, 4.99) | 0.77 |  |
|  | Student | 1.89 | (1.24, 2.53) | 0.33 |  |
|  | Homemaker | 0.43 | (0.29, 0.57) | 0.07 |  |
|  | Unemployed and looking for a job | 4.94 | (4.16, 5.72) | 0.40 |  |
|  | None of these/Other | 6.74 | (5.81, 7.67) | 0.47 |  |
| Religious service attendance | More than 1/week | 3.98 | (3.63, 4.34) | 0.18 | 0.114 |
|  | 1/week | 3.97 | (3.60, 4.35) | 0.19 |  |
|  | 1-3/month | 3.30 | (2.70, 3.89) | 0.30 |  |
|  | A few times a year | 3.26 | (2.61, 3.91) | 0.33 |  |
|  | Never | 3.79 | (2.68, 4.90) | 0.56 |  |
| Education | Up to 8 years | 4.06 | (3.67, 4.46) | 0.20 | < .001 |
|  | 9-15 years | 3.76 | (3.48, 4.04) | 0.14 |  |
|  | 16+ years | 2.63 | (2.09, 3.16) | 0.27 |  |
| Immigration status | Born in this country | 3.83 | (3.60, 4.07) | 0.12 | < .001 |
|  | Born in another country | 2.21 | (0.00, 4.79) | 1.09 |  |
| Religious affiliation | Christianity | 3.35 | (2.28, 4.43) | 0.55 | < .001 |
|  | Islam | 3.89 | (3.65, 4.13) | 0.12 |  |
|  | Hinduism | 1.64 | (0.00, 3.54) | 0.95 |  |
|  | Buddhism | 3.75 | * | * |  |
|  | Taoism | 0.00 | * | * |  |
|  | Some other religion | 0.00 | * | * |  |
| Race/Ethnicity | Banjar/Melayu Banjar | 3.99 | (2.60, 5.38) | 0.70 | 0.002 |
|  | Betawi | 5.14 | (3.92, 6.35) | 0.62 |  |
|  | Bugis | 3.38 | (2.46, 4.31) | 0.47 |  |
|  | Jawa | 3.53 | (3.17, 3.90) | 0.19 |  |
|  | Madura | 3.91 | (2.68, 5.14) | 0.62 |  |
|  | Minangkabau | 4.57 | (3.67, 5.47) | 0.45 |  |
|  | Sunda/Parahyangan | 4.62 | (3.99, 5.25) | 0.32 |  |
|  | Bali | 1.96 | (0.00, 4.05) | 1.04 |  |
|  | Batak | 5.12 | (2.51, 7.74) | 1.32 |  |
|  | Makasar | 3.68 | (2.34, 5.03) | 0.67 |  |
|  | Other | 3.29 | (2.83, 3.75) | 0.23 |  |

# Table S9a. Nationally representative descriptive statistics for Israel

| **Characteristic** | **N = 3,669**^1^ |
| --- | --- |
| **Age group** |  |
| 18-24 | 553 (15%) |
| 25-34 | 712 (19%) |
| 35-44 | 666 (18%) |
| 45-54 | 592 (16%) |
| 55-64 | 504 (14%) |
| 65-74 | 427 (12%) |
| 75-84 | 202 (5.5%) |
| 85 or older | 12 (0.3%) |
| (Missing) | 0 (0%) |
| **Gender** |  |
| Male | 1,791 (49%) |
| Female | 1,872 (51%) |
| Other | 0 (<0.1%) |
| (Missing) | 6 (0.2%) |
| **Marital status** |  |
| Married | 2,056 (56%) |
| Separated | 48 (1.3%) |
| Divorced | 258 (7.0%) |
| Widowed | 212 (5.8%) |
| Single, never married | 834 (23%) |
| Domestic Partner | 193 (5.3%) |
| (Missing) | 69 (1.9%) |
| **Employment** |  |
| Employed for an employer | 1,793 (49%) |
| Self-employed | 424 (12%) |
| Retired | 576 (16%) |
| Student | 388 (11%) |
| Homemaker | 211 (5.7%) |
| Unemployed and looking for a job | 148 (4.0%) |
| None of these/Other | 118 (3.2%) |
| (Missing) | 10 (0.3%) |
| **Religious service attendance** |  |
| More than 1/week | 649 (18%) |
| 1/week | 495 (14%) |
| 1-3/month | 374 (10%) |
| A few times a year | 1,014 (28%) |
| Never | 1,122 (31%) |
| (Missing) | 14 (0.4%) |
| **Education** |  |
| Up to 8 years | 224 (6.1%) |
| 9-15 years | 1,517 (41%) |
| 16+ years | 1,926 (52%) |
| (Missing) | 2 (<0.1%) |
| **Immigration** |  |
| Born in this country | 2,796 (76%) |
| Born in another country | 868 (24%) |
| (Missing) | 5 (0.1%) |
| **Religious affiliation** |  |
| Christianity | 39 (1.1%) |
| Islam | 656 (18%) |
| Hinduism | 0 (0%) |
| Buddhism | 0 (0%) |
| Judaism | 2,897 (79%) |
| Sikhism | 0 (0%) |
| Baha'i | 2 (<0.1%) |
| Jainism | 0 (0%) |
| Shinto | 0 (0%) |
| Taoism | 1 (<0.1%) |
| Confucianism | 0 (0%) |
| Primal, Animist, or Folk religion | 1 (<0.1%) |
| Spiritism | 0 (0%) |
| Umbanda, Candomble, and other African-derived religions | 0 (0%) |
| Chinese folk/traditional religion | 0 (0%) |
| Some other religion | 5 (0.1%) |
| No religion/Atheist/Agnostic | 64 (1.7%) |
| (Missing) | 4 (0.1%) |
| **Race/Ethnicity** |  |
| Arab | 674 (18%) |
| Jewish | 2,926 (80%) |
| Other | 39 (1.1%) |
| (Missing) | 30 (0.8%) |
| ^1^n (%) | |

# Table S9b. Means by demographic category for Israel

| Variable | Category | Mean | 95% CI | SE | Global p-value |
| --- | --- | --- | --- | --- | --- |
| Age group | 18-24 | 1.86 | (1.30, 2.43) | 0.29 | < .001 |
|  | 25-34 | 3.43 | (2.37, 4.48) | 0.54 |  |
|  | 35-44 | 4.16 | (3.44, 4.88) | 0.37 |  |
|  | 45-54 | 3.39 | (2.80, 3.98) | 0.30 |  |
|  | 55-64 | 3.67 | (2.89, 4.45) | 0.40 |  |
|  | 65-74 | 2.30 | (1.51, 3.09) | 0.40 |  |
|  | 75-84 | 0.99 | (0.16, 1.82) | 0.42 |  |
|  | 85 or older | 1.44 | (0.00, 3.94) | 0.73 |  |
| Gender | Male | 4.98 | (4.30, 5.66) | 0.35 | < .001 |
|  | Female | 1.26 | (1.07, 1.45) | 0.10 |  |
|  | Other | 3.00 | * | * |  |
| Marital status | Married | 3.15 | (2.74, 3.56) | 0.21 | < .001 |
|  | Separated | 4.32 | (1.99, 6.65) | 1.15 |  |
|  | Divorced | 5.07 | (3.75, 6.40) | 0.67 |  |
|  | Widowed | 2.70 | (0.09, 5.31) | 1.32 |  |
|  | Never | 2.47 | (1.94, 3.00) | 0.27 |  |
|  | Domestic Partner | 2.41 | (1.53, 3.29) | 0.45 |  |
| Employment | Employed for an employer | 3.16 | (2.71, 3.61) | 0.23 | < .001 |
|  | Self-employed | 5.25 | (3.54, 6.96) | 0.87 |  |
|  | Retired | 2.13 | (1.39, 2.86) | 0.37 |  |
|  | Student | 2.43 | (1.67, 3.19) | 0.39 |  |
|  | Homemaker | 1.09 | (0.36, 1.82) | 0.37 |  |
|  | Unemployed and looking for a job | 3.83 | (2.03, 5.62) | 0.91 |  |
|  | None of these/Other | 3.42 | (1.70, 5.13) | 0.87 |  |
| Religious service attendance | More than 1/week | 2.83 | (2.24, 3.43) | 0.30 | 0.030 |
|  | 1/week | 2.27 | (1.68, 2.86) | 0.30 |  |
|  | 1-3/month | 2.58 | (1.75, 3.40) | 0.42 |  |
|  | A few times a year | 3.03 | (2.49, 3.58) | 0.28 |  |
|  | Never | 3.79 | (2.98, 4.60) | 0.41 |  |
| Education | Up to 8 years | 4.69 | (2.55, 6.84) | 1.09 | < .001 |
|  | 9-15 years | 3.73 | (3.12, 4.35) | 0.31 |  |
|  | 16+ years | 2.38 | (2.04, 2.71) | 0.17 |  |
| Immigration status | Born in this country | 3.20 | (2.76, 3.64) | 0.23 | 0.108 |
|  | Born in another country | 2.69 | (2.20, 3.17) | 0.25 |  |
| Religious affiliation | Christianity | 6.82 | (2.65, 11.00) | 2.04 | < .001 |
|  | Islam | 4.87 | (3.49, 6.26) | 0.70 |  |
|  | Judaism | 2.55 | (2.26, 2.84) | 0.15 |  |
|  | Baha'i | 0.00 | * | * |  |
|  | Taoism | 0.00 | * | * |  |
|  | Primal, Animist, or Folk religion | 0.00 | * | * |  |
|  | Some other religion | 7.60 | (0.00, 19.14) | 5.81 |  |
|  | No religion/Atheist/Agnostic | 6.10 | (3.52, 8.69) | 1.29 |  |
| Race/Ethnicity | Arab | 5.07 | (3.69, 6.45) | 0.70 | 0.001 |
|  | Jewish | 2.58 | (2.27, 2.88) | 0.15 |  |
|  | Other | 5.56 | (2.01, 9.12) | 1.76 |  |

# Table S10a. Nationally representative descriptive statistics for Japan

| **Characteristic** | **N = 20,543**^1^ |
| --- | --- |
| **Age group** |  |
| 18-24 | 1,589 (7.7%) |
| 25-34 | 2,425 (12%) |
| 35-44 | 3,075 (15%) |
| 45-54 | 3,595 (17%) |
| 55-64 | 2,965 (14%) |
| 65-74 | 5,300 (26%) |
| 75-84 | 1,472 (7.2%) |
| 85 or older | 120 (0.6%) |
| (Missing) | 0 (0%) |
| **Gender** |  |
| Male | 9,847 (48%) |
| Female | 10,602 (52%) |
| Other | 28 (0.1%) |
| (Missing) | 66 (0.3%) |
| **Marital status** |  |
| Married | 11,837 (58%) |
| Separated | 190 (0.9%) |
| Divorced | 2,126 (10%) |
| Widowed | 1,179 (5.7%) |
| Single, never married | 5,004 (24%) |
| Domestic Partner | 144 (0.7%) |
| (Missing) | 64 (0.3%) |
| **Employment** |  |
| Employed for an employer | 10,853 (53%) |
| Self-employed | 1,748 (8.5%) |
| Retired | 2,535 (12%) |
| Student | 491 (2.4%) |
| Homemaker | 1,276 (6.2%) |
| Unemployed and looking for a job | 622 (3.0%) |
| None of these/Other | 2,983 (15%) |
| (Missing) | 36 (0.2%) |
| **Religious service attendance** |  |
| More than 1/week | 316 (1.5%) |
| 1/week | 348 (1.7%) |
| 1-3/month | 862 (4.2%) |
| A few times a year | 3,112 (15%) |
| Never | 15,788 (77%) |
| (Missing) | 117 (0.6%) |
| **Education** |  |
| Up to 8 years | 567 (2.8%) |
| 9-15 years | 14,893 (72%) |
| 16+ years | 5,083 (25%) |
| (Missing) | 0 (0%) |
| **Immigration** |  |
| Born in this country | 19,548 (95%) |
| Born in another country | 158 (0.8%) |
| (Missing) | 837 (4.1%) |
| **Religious affiliation** |  |
| Christianity | 381 (1.9%) |
| Islam | 10 (<0.1%) |
| Hinduism | 5 (<0.1%) |
| Buddhism | 6,709 (33%) |
| Judaism | 10 (<0.1%) |
| Sikhism | 6 (<0.1%) |
| Baha'i | 2 (<0.1%) |
| Jainism | 11 (<0.1%) |
| Shinto | 469 (2.3%) |
| Taoism | 7 (<0.1%) |
| Confucianism | 17 (<0.1%) |
| Primal, Animist, or Folk religion | 19 (<0.1%) |
| Spiritism | 0 (0%) |
| Umbanda, Candomble, and other African-derived religions | 0 (0%) |
| Chinese folk/traditional religion | 0 (0%) |
| Some other religion | 46 (0.2%) |
| No religion/Atheist/Agnostic | 12,497 (61%) |
| (Missing) | 355 (1.7%) |
| ^1^n (%) | |

# Table S10b. Means by demographic category for Japan

| Variable | Category | Mean | 95% CI | SE | Global p-value |
| --- | --- | --- | --- | --- | --- |
| Age group | 18-24 | 0.88 | (0.64, 1.13) | 0.13 | < .001 |
|  | 25-34 | 2.29 | (2.01, 2.58) | 0.14 |  |
|  | 35-44 | 3.87 | (3.54, 4.20) | 0.17 |  |
|  | 45-54 | 4.26 | (3.97, 4.56) | 0.15 |  |
|  | 55-64 | 4.48 | (4.15, 4.80) | 0.17 |  |
|  | 65-74 | 2.37 | (2.18, 2.56) | 0.10 |  |
|  | 75-84 | 1.25 | (0.99, 1.52) | 0.14 |  |
|  | 85 or older | 0.57 | (0.00, 1.46) | 0.43 |  |
| Gender | Male | 4.47 | (4.29, 4.65) | 0.09 | < .001 |
|  | Female | 1.67 | (1.55, 1.79) | 0.06 |  |
|  | Other | 1.00 | (0.00, 2.11) | 0.53 |  |
| Marital status | Married | 3.11 | (2.97, 3.26) | 0.07 | < .001 |
|  | Separated | 3.58 | (2.27, 4.89) | 0.66 |  |
|  | Divorced | 4.42 | (3.97, 4.87) | 0.23 |  |
|  | Widowed | 1.41 | (1.06, 1.76) | 0.18 |  |
|  | Never | 2.51 | (2.33, 2.68) | 0.09 |  |
|  | Domestic Partner | 4.50 | (2.99, 6.01) | 0.76 |  |
| Employment | Employed for an employer | 3.57 | (3.40, 3.74) | 0.08 | < .001 |
|  | Self-employed | 4.54 | (4.08, 5.00) | 0.23 |  |
|  | Retired | 2.07 | (1.83, 2.30) | 0.12 |  |
|  | Student | 0.50 | (0.01, 0.98) | 0.25 |  |
|  | Homemaker | 1.33 | (1.06, 1.60) | 0.14 |  |
|  | Unemployed and looking for a job | 3.05 | (2.61, 3.49) | 0.22 |  |
|  | None of these/Other | 2.04 | (1.82, 2.26) | 0.11 |  |
| Religious service attendance | More than 1/week | 3.31 | (2.45, 4.18) | 0.44 | 0.010 |
|  | 1/week | 3.30 | (2.45, 4.14) | 0.43 |  |
|  | 1-3/month | 2.90 | (2.40, 3.40) | 0.25 |  |
|  | A few times a year | 3.49 | (3.19, 3.79) | 0.15 |  |
|  | Never | 2.92 | (2.80, 3.04) | 0.06 |  |
| Education | Up to 8 years | 4.34 | (3.56, 5.13) | 0.40 | < .001 |
|  | 9-15 years | 3.36 | (3.22, 3.49) | 0.07 |  |
|  | 16+ years | 1.87 | (1.69, 2.05) | 0.09 |  |
| Immigration status | Born in this country | 3.02 | (2.92, 3.13) | 0.06 | 0.010 |
|  | Born in another country | 2.04 | (1.14, 2.95) | 0.45 |  |
| Religious affiliation | Christianity | 1.94 | (1.35, 2.54) | 0.30 | < .001 |
|  | Islam | 2.66 | * | * |  |
|  | Hinduism | 6.16 | * | * |  |
|  | Buddhism | 3.20 | (3.00, 3.39) | 0.10 |  |
|  | Judaism | 3.89 | * | * |  |
|  | Sikhism | 4.48 | * | * |  |
|  | Baha'i | 10.10 | * | * |  |
|  | Jainism | 3.37 | (1.19, 5.56) | 1.07 |  |
|  | Shinto | 3.08 | (2.35, 3.82) | 0.37 |  |
|  | Taoism | 0.00 | * | * |  |
|  | Confucianism | 2.89 | (0.00, 23.09) | 1.89 |  |
|  | Primal, Animist, or Folk religion | 9.53 | (0.00, 24.98) | 4.73 |  |
|  | Some other religion | 2.32 | (0.00, 4.87) | 1.20 |  |
|  | No religion/Atheist/Agnostic | 2.94 | (2.80, 3.08) | 0.07 |  |

# Table S11a. Nationally representative descriptive statistics for Kenya

| **Characteristic** | **N = 11,389**^1^ |
| --- | --- |
| **Age group** |  |
| 18-24 | 2,868 (25%) |
| 25-34 | 3,335 (29%) |
| 35-44 | 2,182 (19%) |
| 45-54 | 1,378 (12%) |
| 55-64 | 872 (7.7%) |
| 65-74 | 600 (5.3%) |
| 75-84 | 122 (1.1%) |
| 85 or older | 27 (0.2%) |
| (Missing) | 5 (<0.1%) |
| **Gender** |  |
| Male | 5,567 (49%) |
| Female | 5,813 (51%) |
| Other | 2 (<0.1%) |
| (Missing) | 7 (<0.1%) |
| **Marital status** |  |
| Married | 6,626 (58%) |
| Separated | 467 (4.1%) |
| Divorced | 111 (1.0%) |
| Widowed | 464 (4.1%) |
| Single, never married | 3,531 (31%) |
| Domestic Partner | 146 (1.3%) |
| (Missing) | 43 (0.4%) |
| **Employment** |  |
| Employed for an employer | 1,467 (13%) |
| Self-employed | 3,630 (32%) |
| Retired | 319 (2.8%) |
| Student | 1,136 (10.0%) |
| Homemaker | 1,537 (13%) |
| Unemployed and looking for a job | 3,153 (28%) |
| None of these/Other | 138 (1.2%) |
| (Missing) | 9 (<0.1%) |
| **Religious service attendance** |  |
| More than 1/week | 2,774 (24%) |
| 1/week | 6,063 (53%) |
| 1-3/month | 1,219 (11%) |
| A few times a year | 855 (7.5%) |
| Never | 465 (4.1%) |
| (Missing) | 13 (0.1%) |
| **Education** |  |
| Up to 8 years | 4,485 (39%) |
| 9-15 years | 6,115 (54%) |
| 16+ years | 783 (6.9%) |
| (Missing) | 6 (<0.1%) |
| **Immigration** |  |
| Born in this country | 11,270 (99%) |
| Born in another country | 117 (1.0%) |
| (Missing) | 2 (<0.1%) |
| **Religious affiliation** |  |
| Christianity | 10,334 (91%) |
| Islam | 918 (8.1%) |
| Hinduism | 0 (0%) |
| Buddhism | 1 (<0.1%) |
| Judaism | 3 (<0.1%) |
| Sikhism | 0 (0%) |
| Baha'i | 1 (<0.1%) |
| Jainism | 1 (<0.1%) |
| Shinto | 0 (0%) |
| Taoism | 0 (0%) |
| Confucianism | 3 (<0.1%) |
| Primal, Animist, or Folk religion | 7 (<0.1%) |
| Spiritism | 0 (0%) |
| Umbanda, Candomble, and other African-derived religions | 0 (0%) |
| Chinese folk/traditional religion | 0 (0%) |
| Some other religion | 5 (<0.1%) |
| No religion/Atheist/Agnostic | 108 (0.9%) |
| (Missing) | 9 (<0.1%) |
| **Race/Ethnicity** |  |
| Embu | 197 (1.7%) |
| Kalenjin | 1,377 (12%) |
| Kamba | 1,299 (11%) |
| Kenyan Somali/Somali | 396 (3.5%) |
| Kikuyu | 2,119 (19%) |
| Kisii | 789 (6.9%) |
| Luhya | 1,943 (17%) |
| Luo | 1,120 (9.8%) |
| Maasai | 237 (2.1%) |
| Meru | 630 (5.5%) |
| Miji Kenda tribes | 708 (6.2%) |
| Other | 548 (4.8%) |
| (Missing) | 27 (0.2%) |
| ^1^n (%) | |

# Table S11b. Means by demographic category for Kenya

| Variable | Category | Mean | 95% CI | SE | Global p-value |
| --- | --- | --- | --- | --- | --- |
| Age group | 18-24 | 0.12 | (0.08, 0.17) | 0.02 | < .001 |
|  | 25-34 | 0.21 | (0.14, 0.28) | 0.04 |  |
|  | 35-44 | 0.34 | (0.23, 0.44) | 0.05 |  |
|  | 45-54 | 0.50 | (0.32, 0.67) | 0.09 |  |
|  | 55-64 | 0.50 | (0.25, 0.75) | 0.13 |  |
|  | 65-74 | 0.42 | (0.21, 0.64) | 0.11 |  |
|  | 75-84 | 0.08 | (0.00, 0.18) | 0.05 |  |
|  | 85 or older | 0.00 | * | * |  |
| Gender | Male | 0.51 | (0.43, 0.59) | 0.04 | < .001 |
|  | Female | 0.06 | (0.02, 0.09) | 0.02 |  |
|  | Other | 0.00 | * | * |  |
| Marital status | Married | 0.30 | (0.23, 0.36) | 0.03 | < .001 |
|  | Separated | 0.56 | (0.33, 0.79) | 0.12 |  |
|  | Divorced | 0.71 | (0.25, 1.16) | 0.23 |  |
|  | Widowed | 0.30 | (0.08, 0.53) | 0.12 |  |
|  | Never | 0.17 | (0.11, 0.22) | 0.03 |  |
|  | Domestic Partner | 0.90 | (0.02, 1.78) | 0.44 |  |
| Employment | Employed for an employer | 0.38 | (0.21, 0.54) | 0.08 | < .001 |
|  | Self-employed | 0.37 | (0.28, 0.45) | 0.04 |  |
|  | Retired | 0.44 | (0.16, 0.73) | 0.14 |  |
|  | Student | 0.06 | (0.03, 0.09) | 0.02 |  |
|  | Homemaker | 0.10 | (0.01, 0.19) | 0.05 |  |
|  | Unemployed and looking for a job | 0.24 | (0.17, 0.31) | 0.03 |  |
|  | None of these/Other | 1.37 | (0.39, 2.35) | 0.50 |  |
| Religious service attendance | More than 1/week | 0.11 | (0.07, 0.16) | 0.02 | < .001 |
|  | 1/week | 0.19 | (0.14, 0.23) | 0.02 |  |
|  | 1-3/month | 0.44 | (0.26, 0.62) | 0.09 |  |
|  | A few times a year | 0.76 | (0.48, 1.03) | 0.14 |  |
|  | Never | 1.20 | (0.75, 1.64) | 0.23 |  |
| Education | Up to 8 years | 0.33 | (0.25, 0.40) | 0.04 | 0.136 |
|  | 9-15 years | 0.24 | (0.18, 0.30) | 0.03 |  |
|  | 16+ years | 0.33 | (0.14, 0.51) | 0.09 |  |
| Immigration status | Born in this country | 0.28 | (0.24, 0.33) | 0.02 | 0.138 |
|  | Born in another country | 0.13 | (0.00, 0.33) | 0.10 |  |
| Religious affiliation | Christianity | 0.25 | (0.21, 0.29) | 0.02 | * |
|  | Islam | 0.50 | (0.29, 0.70) | 0.11 |  |
|  | Buddhism | 0.00 | * | * |  |
|  | Judaism | 0.00 | * | * |  |
|  | Baha'i | 0.00 | * | * |  |
|  | Jainism | 0.00 | * | * |  |
|  | Confucianism | 0.00 | * | * |  |
|  | Primal, Animist, or Folk religion | 1.83 | (0.00, 4.79) | 1.48 |  |
|  | Some other religion | 0.00 | * | * |  |
|  | No religion/Atheist/Agnostic | 1.38 | (0.41, 2.36) | 0.49 |  |
| Race/Ethnicity | Luhya | 0.18 | (0.09, 0.27) | 0.05 | < .001 |
|  | Luo | 0.13 | (0.06, 0.21) | 0.04 |  |
|  | Kalenjin | 0.10 | (0.05, 0.15) | 0.03 |  |
|  | Kamba | 0.52 | (0.33, 0.71) | 0.10 |  |
|  | Kikuyu | 0.37 | (0.27, 0.46) | 0.05 |  |
|  | Kisii | 0.20 | (0.00, 0.40) | 0.11 |  |
|  | Maasai | 0.07 | (0.00, 0.15) | 0.04 |  |
|  | Meru | 0.59 | (0.35, 0.84) | 0.12 |  |
|  | Kenyan Somali/Somali | 0.59 | (0.18, 1.00) | 0.21 |  |
|  | Miji Kenda tribes | 0.26 | (0.12, 0.40) | 0.07 |  |
|  | Embu | 0.20 | (0.00, 0.48) | 0.14 |  |
|  | Other | 0.18 | (0.07, 0.30) | 0.06 |  |

# Table S12a. Nationally representative descriptive statistics for Mexico

| **Characteristic** | **N = 5,776**^1^ |
| --- | --- |
| **Age group** |  |
| 18-24 | 986 (17%) |
| 25-34 | 1,293 (22%) |
| 35-44 | 1,158 (20%) |
| 45-54 | 989 (17%) |
| 55-64 | 697 (12%) |
| 65-74 | 498 (8.6%) |
| 75-84 | 131 (2.3%) |
| 85 or older | 24 (0.4%) |
| (Missing) | 0 (0%) |
| **Gender** |  |
| Male | 2,755 (48%) |
| Female | 2,997 (52%) |
| Other | 3 (<0.1%) |
| (Missing) | 21 (0.4%) |
| **Marital status** |  |
| Married | 2,089 (36%) |
| Separated | 403 (7.0%) |
| Divorced | 230 (4.0%) |
| Widowed | 347 (6.0%) |
| Single, never married | 1,432 (25%) |
| Domestic Partner | 1,109 (19%) |
| (Missing) | 166 (2.9%) |
| **Employment** |  |
| Employed for an employer | 1,921 (33%) |
| Self-employed | 1,091 (19%) |
| Retired | 386 (6.7%) |
| Student | 247 (4.3%) |
| Homemaker | 1,257 (22%) |
| Unemployed and looking for a job | 564 (9.8%) |
| None of these/Other | 169 (2.9%) |
| (Missing) | 141 (2.4%) |
| **Religious service attendance** |  |
| More than 1/week | 609 (11%) |
| 1/week | 1,261 (22%) |
| 1-3/month | 676 (12%) |
| A few times a year | 2,054 (36%) |
| Never | 1,134 (20%) |
| (Missing) | 43 (0.7%) |
| **Education** |  |
| Up to 8 years | 1,291 (22%) |
| 9-15 years | 3,180 (55%) |
| 16+ years | 1,304 (23%) |
| (Missing) | 1 (<0.1%) |
| **Immigration** |  |
| Born in this country | 5,517 (96%) |
| Born in another country | 108 (1.9%) |
| (Missing) | 151 (2.6%) |
| **Religious affiliation** |  |
| Christianity | 4,844 (84%) |
| Islam | 2 (<0.1%) |
| Hinduism | 3 (<0.1%) |
| Buddhism | 6 (0.1%) |
| Judaism | 7 (0.1%) |
| Sikhism | 0 (0%) |
| Baha'i | 1 (<0.1%) |
| Jainism | 1 (<0.1%) |
| Shinto | 2 (<0.1%) |
| Taoism | 4 (<0.1%) |
| Confucianism | 1 (<0.1%) |
| Primal, Animist, or Folk religion | 20 (0.3%) |
| Spiritism | 0 (0%) |
| Umbanda, Candomble, and other African-derived religions | 0 (0%) |
| Chinese folk/traditional religion | 0 (0%) |
| Some other religion | 41 (0.7%) |
| No religion/Atheist/Agnostic | 770 (13%) |
| (Missing) | 75 (1.3%) |
| **Race/Ethnicity** |  |
| Black | 108 (1.9%) |
| Indigenous | 594 (10%) |
| Mestizo | 2,762 (48%) |
| Mulatto | 63 (1.1%) |
| Other | 339 (5.9%) |
| White | 1,116 (19%) |
| (Missing) | 794 (14%) |
| ^1^n (%) | |

# Table S12b. Means by demographic category for Mexico

| Variable | Category | Mean | 95% CI | SE | Global p-value |
| --- | --- | --- | --- | --- | --- |
| Age group | 18-24 | 1.15 | (0.61, 1.69) | 0.28 | 0.895 |
|  | 25-34 | 1.26 | (1.01, 1.52) | 0.13 |  |
|  | 35-44 | 1.35 | (0.98, 1.72) | 0.19 |  |
|  | 45-54 | 1.08 | (0.72, 1.45) | 0.19 |  |
|  | 55-64 | 1.17 | (0.87, 1.47) | 0.15 |  |
|  | 65-74 | 1.52 | (0.94, 2.11) | 0.30 |  |
|  | 75-84 | 1.24 | (0.28, 2.20) | 0.48 |  |
|  | 85 or older | 0.80 | (0.00, 2.10) | 0.60 |  |
| Gender | Male | 1.95 | (1.64, 2.27) | 0.16 | < .001 |
|  | Female | 0.58 | (0.48, 0.68) | 0.05 |  |
|  | Other | 2.91 | * | * |  |
| Marital status | Married | 0.96 | (0.77, 1.15) | 0.09 | 0.017 |
|  | Separated | 2.23 | (1.34, 3.13) | 0.46 |  |
|  | Divorced | 2.15 | (0.87, 3.43) | 0.65 |  |
|  | Widowed | 1.26 | (0.74, 1.77) | 0.26 |  |
|  | Never | 1.37 | (0.94, 1.80) | 0.22 |  |
|  | Domestic Partner | 1.04 | (0.86, 1.23) | 0.10 |  |
| Employment | Employed for an employer | 1.42 | (1.17, 1.67) | 0.13 | < .001 |
|  | Self-employed | 1.52 | (1.13, 1.91) | 0.20 |  |
|  | Retired | 1.51 | (0.85, 2.16) | 0.33 |  |
|  | Student | 0.60 | (0.33, 0.88) | 0.14 |  |
|  | Homemaker | 0.48 | (0.32, 0.64) | 0.08 |  |
|  | Unemployed and looking for a job | 1.89 | (0.92, 2.87) | 0.50 |  |
|  | None of these/Other | 1.20 | (0.45, 1.95) | 0.36 |  |
| Religious service attendance | More than 1/week | 0.50 | (0.31, 0.68) | 0.09 | < .001 |
|  | 1/week | 0.82 | (0.59, 1.05) | 0.12 |  |
|  | 1-3/month | 1.00 | (0.67, 1.34) | 0.17 |  |
|  | A few times a year | 1.47 | (1.20, 1.74) | 0.14 |  |
|  | Never | 1.83 | (1.26, 2.39) | 0.29 |  |
| Education | Up to 8 years | 1.47 | (0.97, 1.96) | 0.25 | 0.041 |
|  | 9-15 years | 1.26 | (1.06, 1.45) | 0.10 |  |
|  | 16+ years | 0.97 | (0.77, 1.16) | 0.10 |  |
| Immigration status | Born in this country | 1.20 | (1.04, 1.36) | 0.08 | 0.015 |
|  | Born in another country | 3.35 | (1.60, 5.10) | 0.88 |  |
| Religious affiliation | Christianity | 1.12 | (0.97, 1.26) | 0.07 | < .001 |
|  | Islam | 0.00 | * | * |  |
|  | Hinduism | 1.65 | * | * |  |
|  | Buddhism | 1.19 | * | * |  |
|  | Judaism | 1.49 | * | * |  |
|  | Baha'i | 0.00 | * | * |  |
|  | Jainism | 18.00 | * | * |  |
|  | Shinto | 3.33 | * | * |  |
|  | Taoism | 3.75 | * | * |  |
|  | Confucianism | 1.00 | * | * |  |
|  | Primal, Animist, or Folk religion | 1.86 | (0.00, 5.49) | 1.39 |  |
|  | Some other religion | 2.16 | (0.73, 3.59) | 0.69 |  |
|  | No religion/Atheist/Agnostic | 1.92 | (1.13, 2.70) | 0.40 |  |
| Race/Ethnicity | Black | 1.32 | (0.50, 2.14) | 0.40 | 0.514 |
|  | Indigenous | 1.08 | (0.29, 1.87) | 0.40 |  |
|  | White | 1.47 | (1.14, 1.81) | 0.17 |  |
|  | Mestizo | 1.15 | (0.97, 1.33) | 0.09 |  |
|  | Mulatto | 1.46 | (0.76, 2.16) | 0.35 |  |
|  | Other | 1.41 | (0.75, 2.07) | 0.34 |  |

# Table S13a. Nationally representative descriptive statistics for Nigeria

| **Characteristic** | **N = 6,827**^1^ |
| --- | --- |
| **Age group** |  |
| 18-24 | 1,533 (22%) |
| 25-34 | 2,145 (31%) |
| 35-44 | 1,552 (23%) |
| 45-54 | 873 (13%) |
| 55-64 | 419 (6.1%) |
| 65-74 | 224 (3.3%) |
| 75-84 | 63 (0.9%) |
| 85 or older | 19 (0.3%) |
| (Missing) | 0 (0%) |
| **Gender** |  |
| Male | 3,371 (49%) |
| Female | 3,456 (51%) |
| Other | 0 (<0.1%) |
| (Missing) | 0 (0%) |
| **Marital status** |  |
| Married | 4,065 (60%) |
| Separated | 117 (1.7%) |
| Divorced | 71 (1.0%) |
| Widowed | 231 (3.4%) |
| Single, never married | 2,289 (34%) |
| Domestic Partner | 12 (0.2%) |
| (Missing) | 42 (0.6%) |
| **Employment** |  |
| Employed for an employer | 699 (10%) |
| Self-employed | 3,898 (57%) |
| Retired | 178 (2.6%) |
| Student | 650 (9.5%) |
| Homemaker | 499 (7.3%) |
| Unemployed and looking for a job | 684 (10%) |
| None of these/Other | 211 (3.1%) |
| (Missing) | 8 (0.1%) |
| **Religious service attendance** |  |
| More than 1/week | 4,049 (59%) |
| 1/week | 1,895 (28%) |
| 1-3/month | 531 (7.8%) |
| A few times a year | 254 (3.7%) |
| Never | 77 (1.1%) |
| (Missing) | 20 (0.3%) |
| **Education** |  |
| Up to 8 years | 2,575 (38%) |
| 9-15 years | 4,120 (60%) |
| 16+ years | 130 (1.9%) |
| (Missing) | 2 (<0.1%) |
| **Immigration** |  |
| Born in this country | 6,779 (99%) |
| Born in another country | 47 (0.7%) |
| (Missing) | 1 (<0.1%) |
| **Religious affiliation** |  |
| Christianity | 3,476 (51%) |
| Islam | 3,302 (48%) |
| Hinduism | 0 (0%) |
| Buddhism | 0 (0%) |
| Judaism | 0 (0%) |
| Sikhism | 0 (0%) |
| Baha'i | 0 (0%) |
| Jainism | 0 (0%) |
| Shinto | 1 (<0.1%) |
| Taoism | 0 (0%) |
| Confucianism | 0 (<0.1%) |
| Primal, Animist, or Folk religion | 24 (0.3%) |
| Spiritism | 0 (0%) |
| Umbanda, Candomble, and other African-derived religions | 0 (0%) |
| Chinese folk/traditional religion | 0 (0%) |
| Some other religion | 1 (<0.1%) |
| No religion/Atheist/Agnostic | 15 (0.2%) |
| (Missing) | 9 (0.1%) |
| **Race/Ethnicity** |  |
| Edo | 116 (1.7%) |
| Efik | 48 (0.7%) |
| Fulani | 266 (3.9%) |
| Hausa | 2,342 (34%) |
| Ibibio | 180 (2.6%) |
| Idoma | 61 (0.9%) |
| Igala | 77 (1.1%) |
| Igbo (Ibo) | 1,111 (16%) |
| Ijaw | 110 (1.6%) |
| Kanuri | 31 (0.5%) |
| Other | 1,014 (15%) |
| Tiv | 198 (2.9%) |
| Urhobo | 38 (0.6%) |
| Yoruba | 1,230 (18%) |
| (Missing) | 4 (<0.1%) |
| ^1^n (%) | |

# Table S13b. Means by demographic category for Nigeria

| Variable | Category | Mean | 95% CI | SE | Global p-value |
| --- | --- | --- | --- | --- | --- |
| Age group | 18-24 | 0.14 | (0.06, 0.22) | 0.04 | 0.040 |
|  | 25-34 | 0.24 | (0.14, 0.33) | 0.05 |  |
|  | 35-44 | 0.47 | (0.25, 0.69) | 0.11 |  |
|  | 45-54 | 0.24 | (0.05, 0.43) | 0.10 |  |
|  | 55-64 | 0.11 | (0.00, 0.23) | 0.06 |  |
|  | 65-74 | 0.07 | (0.00, 0.20) | 0.07 |  |
|  | 75-84 | 0.25 | (0.00, 0.60) | 0.18 |  |
|  | 85 or older | 0.46 | (0.00, 1.54) | 0.47 |  |
| Gender | Male | 0.43 | (0.32, 0.55) | 0.06 | < .001 |
|  | Female | 0.08 | (0.02, 0.15) | 0.03 |  |
|  | Other | 0.00 | * | * |  |
| Marital status | Married | 0.24 | (0.15, 0.32) | 0.04 | 0.520 |
|  | Separated | 0.65 | (0.07, 1.24) | 0.29 |  |
|  | Divorced | 1.44 | (0.00, 3.54) | 1.05 |  |
|  | Widowed | 0.33 | (0.00, 0.89) | 0.28 |  |
|  | Never | 0.22 | (0.14, 0.31) | 0.04 |  |
|  | Domestic Partner | 0.48 | (0.00, 1.31) | 0.30 |  |
| Employment | Employed for an employer | 0.25 | (0.13, 0.37) | 0.06 | 0.004 |
|  | Self-employed | 0.30 | (0.19, 0.40) | 0.05 |  |
|  | Retired | 0.22 | (0.00, 0.43) | 0.11 |  |
|  | Student | 0.10 | (0.05, 0.14) | 0.02 |  |
|  | Homemaker | 0.32 | (0.05, 0.59) | 0.14 |  |
|  | Unemployed and looking for a job | 0.14 | (0.07, 0.21) | 0.04 |  |
|  | None of these/Other | 0.28 | (0.03, 0.54) | 0.13 |  |
| Religious service attendance | More than 1/week | 0.22 | (0.14, 0.29) | 0.04 | 0.425 |
|  | 1/week | 0.27 | (0.15, 0.38) | 0.06 |  |
|  | 1-3/month | 0.31 | (0.09, 0.53) | 0.11 |  |
|  | A few times a year | 0.60 | (0.00, 1.22) | 0.32 |  |
|  | Never | 0.56 | (0.02, 1.10) | 0.27 |  |
| Education | Up to 8 years | 0.25 | (0.13, 0.37) | 0.06 | 0.377 |
|  | 9-15 years | 0.26 | (0.18, 0.35) | 0.04 |  |
|  | 16+ years | 0.16 | (0.04, 0.28) | 0.06 |  |
| Immigration status | Born in this country | 0.26 | (0.19, 0.32) | 0.03 | 0.854 |
|  | Born in another country | 0.32 | (0.00, 0.97) | 0.32 |  |
| Religious affiliation | Christianity | 0.19 | (0.13, 0.26) | 0.03 | < .001 |
|  | Islam | 0.32 | (0.21, 0.44) | 0.06 |  |
|  | Shinto | 0.00 | * | * |  |
|  | Confucianism | 0.00 | * | * |  |
|  | Primal, Animist, or Folk religion | 0.86 | (0.10, 1.62) | 0.35 |  |
|  | Some other religion | 0.00 | * | * |  |
|  | No religion/Atheist/Agnostic | 0.00 | * | * |  |
| Race/Ethnicity | Hausa | 0.33 | (0.19, 0.48) | 0.07 | < .001 |
|  | Yoruba | 0.23 | (0.06, 0.39) | 0.08 |  |
|  | Igbo (Ibo) | 0.21 | (0.08, 0.35) | 0.07 |  |
|  | Edo | 0.18 | (0.03, 0.32) | 0.07 |  |
|  | Urhobo | 0.00 | * | * |  |
|  | Fulani | 0.13 | (0.02, 0.24) | 0.05 |  |
|  | Kanuri | 1.55 | (0.01, 3.09) | 0.72 |  |
|  | Tiv | 0.25 | (0.11, 0.39) | 0.07 |  |
|  | Efik | 0.16 | (0.00, 0.39) | 0.11 |  |
|  | Ijaw | 0.19 | (0.00, 0.50) | 0.16 |  |
|  | Igala | 0.51 | (0.00, 1.29) | 0.39 |  |
|  | Ibibio | 0.09 | (0.00, 0.22) | 0.07 |  |
|  | Idoma | 0.26 | (0.00, 0.51) | 0.13 |  |
|  | Other | 0.20 | (0.09, 0.31) | 0.06 |  |

# Table S14a. Nationally representative descriptive statistics for Philippines

| **Characteristic** | **N = 5,292**^1^ |
| --- | --- |
| **Age group** |  |
| 18-24 | 1,073 (20%) |
| 25-34 | 1,322 (25%) |
| 35-44 | 1,058 (20%) |
| 45-54 | 813 (15%) |
| 55-64 | 641 (12%) |
| 65-74 | 331 (6.3%) |
| 75-84 | 51 (1.0%) |
| 85 or older | 4 (<0.1%) |
| (Missing) | 0 (0%) |
| **Gender** |  |
| Male | 2,625 (50%) |
| Female | 2,643 (50%) |
| Other | 13 (0.2%) |
| (Missing) | 11 (0.2%) |
| **Marital status** |  |
| Married | 2,385 (45%) |
| Separated | 249 (4.7%) |
| Divorced | 9 (0.2%) |
| Widowed | 274 (5.2%) |
| Single, never married | 1,206 (23%) |
| Domestic Partner | 1,152 (22%) |
| (Missing) | 16 (0.3%) |
| **Employment** |  |
| Employed for an employer | 1,350 (26%) |
| Self-employed | 1,379 (26%) |
| Retired | 158 (3.0%) |
| Student | 585 (11%) |
| Homemaker | 1,049 (20%) |
| Unemployed and looking for a job | 658 (12%) |
| None of these/Other | 113 (2.1%) |
| (Missing) | 0 (0%) |
| **Religious service attendance** |  |
| More than 1/week | 844 (16%) |
| 1/week | 1,929 (36%) |
| 1-3/month | 1,374 (26%) |
| A few times a year | 929 (18%) |
| Never | 210 (4.0%) |
| (Missing) | 6 (0.1%) |
| **Education** |  |
| Up to 8 years | 1,188 (22%) |
| 9-15 years | 3,722 (70%) |
| 16+ years | 381 (7.2%) |
| (Missing) | 1 (<0.1%) |
| **Immigration** |  |
| Born in this country | 5,284 (100%) |
| Born in another country | 8 (0.1%) |
| (Missing) | 0 (0%) |
| **Religious affiliation** |  |
| Christianity | 4,914 (93%) |
| Islam | 297 (5.6%) |
| Hinduism | 0 (0%) |
| Buddhism | 4 (<0.1%) |
| Judaism | 4 (<0.1%) |
| Sikhism | 0 (0%) |
| Baha'i | 1 (<0.1%) |
| Jainism | 0 (0%) |
| Shinto | 0 (0%) |
| Taoism | 0 (0%) |
| Confucianism | 0 (0%) |
| Primal, Animist, or Folk religion | 5 (<0.1%) |
| Spiritism | 0 (0%) |
| Umbanda, Candomble, and other African-derived religions | 0 (0%) |
| Chinese folk/traditional religion | 0 (0%) |
| Some other religion | 35 (0.7%) |
| No religion/Atheist/Agnostic | 23 (0.4%) |
| (Missing) | 9 (0.2%) |
| **Race/Ethnicity** |  |
| Aeta | 1 (<0.1%) |
| Badjao | 2 (<0.1%) |
| Bicolano/Bikolano | 300 (5.7%) |
| Cebuano | 656 (12%) |
| Chinese-Filipino | 3 (<0.1%) |
| Igorot | 42 (0.8%) |
| Ilocano/Ilokano | 429 (8.1%) |
| Ilonggo/Hiligaynon | 428 (8.1%) |
| Kapampangan | 107 (2.0%) |
| Maguindanaoan | 84 (1.6%) |
| Mangyan | 2 (<0.1%) |
| Maranao | 39 (0.7%) |
| Masbateno | 54 (1.0%) |
| Other | 244 (4.6%) |
| Pangasinense | 107 (2.0%) |
| Tagalog | 1,691 (32%) |
| Tausug | 94 (1.8%) |
| Visayan/Bisaya | 739 (14%) |
| Waray | 216 (4.1%) |
| Zamboangueno | 51 (1.0%) |
| (Missing) | 3 (<0.1%) |
| ^1^n (%) | |

# Table S14b. Means by demographic category for Philippines

| Variable | Category | Mean | 95% CI | SE | Global p-value |
| --- | --- | --- | --- | --- | --- |
| Age group | 18-24 | 1.05 | (0.66, 1.44) | 0.20 | < .001 |
|  | 25-34 | 1.79 | (1.45, 2.13) | 0.17 |  |
|  | 35-44 | 2.28 | (1.89, 2.66) | 0.20 |  |
|  | 45-54 | 1.96 | (1.60, 2.32) | 0.18 |  |
|  | 55-64 | 1.79 | (1.26, 2.32) | 0.27 |  |
|  | 65-74 | 1.33 | (0.64, 2.03) | 0.35 |  |
|  | 75-84 | 1.61 | (0.00, 3.31) | 0.84 |  |
|  | 85 or older | 0.00 | * | * |  |
| Gender | Male | 3.19 | (2.86, 3.52) | 0.17 | < .001 |
|  | Female | 0.29 | (0.24, 0.35) | 0.03 |  |
|  | Other | 0.28 | (0.00, 0.93) | 0.28 |  |
| Marital status | Married | 1.91 | (1.62, 2.19) | 0.14 | < .001 |
|  | Separated | 1.84 | (1.21, 2.47) | 0.32 |  |
|  | Divorced | 1.49 | (0.00, 6.88) | 1.17 |  |
|  | Widowed | 1.20 | (0.65, 1.76) | 0.28 |  |
|  | Never | 1.09 | (0.81, 1.37) | 0.14 |  |
|  | Domestic Partner | 2.16 | (1.76, 2.56) | 0.20 |  |
| Employment | Employed for an employer | 2.57 | (2.23, 2.91) | 0.17 | < .001 |
|  | Self-employed | 2.46 | (2.04, 2.87) | 0.21 |  |
|  | Retired | 1.85 | (0.69, 3.01) | 0.59 |  |
|  | Student | 0.65 | (0.10, 1.21) | 0.28 |  |
|  | Homemaker | 0.42 | (0.28, 0.56) | 0.07 |  |
|  | Unemployed and looking for a job | 1.64 | (1.26, 2.02) | 0.19 |  |
|  | None of these/Other | 1.11 | (0.53, 1.70) | 0.29 |  |
| Religious service attendance | More than 1/week | 1.65 | (1.18, 2.13) | 0.24 | < .001 |
|  | 1/week | 1.30 | (1.06, 1.54) | 0.12 |  |
|  | 1-3/month | 1.74 | (1.46, 2.02) | 0.14 |  |
|  | A few times a year | 2.51 | (2.03, 2.98) | 0.24 |  |
|  | Never | 2.56 | (1.68, 3.44) | 0.45 |  |
| Education | Up to 8 years | 2.26 | (1.86, 2.67) | 0.21 | < .001 |
|  | 9-15 years | 1.63 | (1.42, 1.84) | 0.11 |  |
|  | 16+ years | 1.09 | (0.70, 1.47) | 0.19 |  |
| Immigration status | Born in this country | 1.73 | (1.56, 1.90) | 0.09 | 0.443 |
|  | Born in another country | 3.15 | (0.00, 8.50) | 1.87 |  |
| Religious affiliation | Christianity | 1.67 | (1.49, 1.85) | 0.09 | < .001 |
|  | Islam | 2.51 | (1.40, 3.62) | 0.56 |  |
|  | Buddhism | 9.75 | * | * |  |
|  | Judaism | 0.83 | * | * |  |
|  | Baha'i | 0.00 | * | * |  |
|  | Primal, Animist, or Folk religion | 0.65 | (0.00, 2.02) | 0.70 |  |
|  | Some other religion | 2.49 | (0.80, 4.18) | 0.82 |  |
|  | No religion/Atheist/Agnostic | 2.65 | (0.00, 5.38) | 1.25 |  |
| Race/Ethnicity | Tagalog | 1.91 | (1.59, 2.22) | 0.16 | < .001 |
|  | Cebuano | 1.69 | (1.24, 2.14) | 0.23 |  |
|  | Ilocano/Ilokano | 1.66 | (1.06, 2.27) | 0.31 |  |
|  | Visayan/Bisaya | 1.07 | (0.67, 1.46) | 0.20 |  |
|  | Ilonggo/Hiligaynon | 1.77 | (1.22, 2.31) | 0.28 |  |
|  | Bicolano/Bikolano | 1.67 | (1.05, 2.30) | 0.32 |  |
|  | Waray | 1.46 | (0.76, 2.16) | 0.35 |  |
|  | Tausug | 3.00 | (0.00, 6.36) | 1.69 |  |
|  | Maranao | 1.94 | (0.72, 3.16) | 0.58 |  |
|  | Maguindanaoan | 2.04 | (1.13, 2.96) | 0.46 |  |
|  | Chinese-Filipino | 0.00 | * | * |  |
|  | Kapampangan | 2.25 | (0.76, 3.74) | 0.75 |  |
|  | Pangasinense | 1.90 | (1.04, 2.75) | 0.43 |  |
|  | Zamboangueno | 3.22 | (1.09, 5.36) | 1.04 |  |
|  | Masbateno | 3.72 | (0.45, 6.99) | 1.60 |  |
|  | Aeta | 0.00 | * | * |  |
|  | Igorot | 1.50 | (0.54, 2.46) | 0.46 |  |
|  | Mangyan | 0.00 | * | * |  |
|  | Badjao | 0.00 | * | * |  |
|  | Other | 1.45 | (0.84, 2.06) | 0.31 |  |

# Table S15a. Nationally representative descriptive statistics for Poland

| **Characteristic** | **N = 10,389**^1^ |
| --- | --- |
| **Age group** |  |
| 18-24 | 955 (9.2%) |
| 25-34 | 1,822 (18%) |
| 35-44 | 2,139 (21%) |
| 45-54 | 1,722 (17%) |
| 55-64 | 1,678 (16%) |
| 65-74 | 1,672 (16%) |
| 75-84 | 352 (3.4%) |
| 85 or older | 47 (0.5%) |
| (Missing) | 1 (<0.1%) |
| **Gender** |  |
| Male | 4,974 (48%) |
| Female | 5,387 (52%) |
| Other | 3 (<0.1%) |
| (Missing) | 26 (0.2%) |
| **Marital status** |  |
| Married | 6,065 (58%) |
| Separated | 111 (1.1%) |
| Divorced | 529 (5.1%) |
| Widowed | 990 (9.5%) |
| Single, never married | 1,811 (17%) |
| Domestic Partner | 504 (4.8%) |
| (Missing) | 379 (3.6%) |
| **Employment** |  |
| Employed for an employer | 5,837 (56%) |
| Self-employed | 686 (6.6%) |
| Retired | 2,434 (23%) |
| Student | 515 (5.0%) |
| Homemaker | 338 (3.3%) |
| Unemployed and looking for a job | 284 (2.7%) |
| None of these/Other | 169 (1.6%) |
| (Missing) | 126 (1.2%) |
| **Religious service attendance** |  |
| More than 1/week | 305 (2.9%) |
| 1/week | 3,263 (31%) |
| 1-3/month | 2,081 (20%) |
| A few times a year | 3,064 (29%) |
| Never | 1,597 (15%) |
| (Missing) | 78 (0.8%) |
| **Education** |  |
| Up to 8 years | 1,238 (12%) |
| 9-15 years | 6,130 (59%) |
| 16+ years | 3,020 (29%) |
| (Missing) | 1 (<0.1%) |
| **Immigration** |  |
| Born in this country | 10,258 (99%) |
| Born in another country | 108 (1.0%) |
| (Missing) | 23 (0.2%) |
| **Religious affiliation** |  |
| Christianity | 9,378 (90%) |
| Islam | 2 (<0.1%) |
| Hinduism | 0 (0%) |
| Buddhism | 2 (<0.1%) |
| Judaism | 0 (0%) |
| Sikhism | 1 (<0.1%) |
| Baha'i | 0 (0%) |
| Jainism | 3 (<0.1%) |
| Shinto | 1 (<0.1%) |
| Taoism | 0 (0%) |
| Confucianism | 0 (0%) |
| Primal, Animist, or Folk religion | 11 (0.1%) |
| Spiritism | 0 (0%) |
| Umbanda, Candomble, and other African-derived religions | 0 (0%) |
| Chinese folk/traditional religion | 0 (0%) |
| Some other religion | 0 (0%) |
| No religion/Atheist/Agnostic | 942 (9.1%) |
| (Missing) | 50 (0.5%) |
| **Race/Ethnicity** |  |
| Belarussian | 2 (<0.1%) |
| German | 4 (<0.1%) |
| Kashubians | 3 (<0.1%) |
| Other | 4 (<0.1%) |
| Polish | 10,309 (99%) |
| Silesia | 14 (0.1%) |
| Ukrainian | 38 (0.4%) |
| (Missing) | 14 (0.1%) |
| ^1^n (%) | |

# Table S15b. Means by demographic category for Poland

| Variable | Category | Mean | 95% CI | SE | Global p-value |
| --- | --- | --- | --- | --- | --- |
| Age group | 18-24 | 2.52 | (1.97, 3.06) | 0.28 | < .001 |
|  | 25-34 | 3.72 | (3.35, 4.09) | 0.19 |  |
|  | 35-44 | 4.07 | (3.64, 4.51) | 0.22 |  |
|  | 45-54 | 4.29 | (3.82, 4.75) | 0.24 |  |
|  | 55-64 | 5.06 | (4.17, 5.95) | 0.45 |  |
|  | 65-74 | 2.45 | (1.96, 2.94) | 0.25 |  |
|  | 75-84 | 1.37 | (0.38, 2.37) | 0.50 |  |
|  | 85 or older | 1.59 | (0.00, 3.49) | 0.94 |  |
| Gender | Male | 5.24 | (4.83, 5.64) | 0.21 | < .001 |
|  | Female | 2.28 | (1.97, 2.59) | 0.16 |  |
|  | Other | 6.14 | * | * |  |
| Marital status | Married | 3.35 | (3.07, 3.62) | 0.14 | < .001 |
|  | Separated | 7.20 | (4.08, 10.32) | 1.57 |  |
|  | Divorced | 6.33 | (5.14, 7.52) | 0.60 |  |
|  | Widowed | 3.61 | (2.23, 5.00) | 0.71 |  |
|  | Never | 3.71 | (3.28, 4.15) | 0.22 |  |
|  | Domestic Partner | 4.56 | (3.76, 5.36) | 0.41 |  |
| Employment | Employed for an employer | 3.95 | (3.66, 4.24) | 0.15 | < .001 |
|  | Self-employed | 5.23 | (4.52, 5.95) | 0.36 |  |
|  | Retired | 2.64 | (2.00, 3.29) | 0.33 |  |
|  | Student | 1.56 | (1.12, 2.01) | 0.23 |  |
|  | Homemaker | 3.41 | (2.09, 4.72) | 0.67 |  |
|  | Unemployed and looking for a job | 7.17 | (4.96, 9.39) | 1.13 |  |
|  | None of these/Other | 5.16 | (3.17, 7.15) | 1.01 |  |
| Religious service attendance | More than 1/week | 1.62 | (0.45, 2.79) | 0.59 | < .001 |
|  | 1/week | 2.31 | (1.82, 2.80) | 0.25 |  |
|  | 1-3/month | 3.73 | (3.26, 4.21) | 0.24 |  |
|  | A few times a year | 4.60 | (4.21, 4.99) | 0.20 |  |
|  | Never | 5.15 | (4.51, 5.78) | 0.32 |  |
| Education | Up to 8 years | 4.92 | (3.49, 6.36) | 0.73 | < .001 |
|  | 9-15 years | 3.92 | (3.65, 4.20) | 0.14 |  |
|  | 16+ years | 2.74 | (2.47, 3.01) | 0.14 |  |
| Immigration status | Born in this country | 3.67 | (3.41, 3.93) | 0.13 | 0.008 |
|  | Born in another country | 6.03 | (4.22, 7.84) | 0.91 |  |
| Religious affiliation | Christianity | 3.61 | (3.33, 3.88) | 0.14 | < .001 |
|  | Islam | 0.00 | * | * |  |
|  | Buddhism | 0.00 | * | * |  |
|  | Sikhism | 12.00 | * | * |  |
|  | Jainism | 0.00 | * | * |  |
|  | Shinto | 20.00 | * | * |  |
|  | Primal, Animist, or Folk religion | 1.49 | (0.00, 6.65) | 0.82 |  |
|  | No religion/Atheist/Agnostic | 4.62 | (3.95, 5.30) | 0.34 |  |
| Race/Ethnicity | Polish | 3.68 | (3.41, 3.94) | 0.13 | < .001 |
|  | German | 0.00 | * | * |  |
|  | Belarussian | 7.05 | * | * |  |
|  | Ukrainian | 9.14 | (3.50, 14.77) | 2.75 |  |
|  | Silesia | 5.62 | (2.27, 8.97) | 1.32 |  |
|  | Kashubians | 3.15 | * | * |  |
|  | Other | 1.17 | (0.00, 5.64) | 1.73 |  |

# Table S16a. Nationally representative descriptive statistics for South Africa

| **Characteristic** | **N = 2,651**^1^ |
| --- | --- |
| **Age group** |  |
| 18-24 | 461 (17%) |
| 25-34 | 709 (27%) |
| 35-44 | 611 (23%) |
| 45-54 | 381 (14%) |
| 55-64 | 261 (9.9%) |
| 65-74 | 158 (6.0%) |
| 75-84 | 58 (2.2%) |
| 85 or older | 2 (<0.1%) |
| (Missing) | 9 (0.3%) |
| **Gender** |  |
| Male | 1,288 (49%) |
| Female | 1,356 (51%) |
| Other | 2 (<0.1%) |
| (Missing) | 4 (0.2%) |
| **Marital status** |  |
| Married | 539 (20%) |
| Separated | 76 (2.9%) |
| Divorced | 51 (1.9%) |
| Widowed | 133 (5.0%) |
| Single, never married | 1,561 (59%) |
| Domestic Partner | 264 (10.0%) |
| (Missing) | 28 (1.0%) |
| **Employment** |  |
| Employed for an employer | 569 (21%) |
| Self-employed | 412 (16%) |
| Retired | 243 (9.2%) |
| Student | 204 (7.7%) |
| Homemaker | 137 (5.2%) |
| Unemployed and looking for a job | 1,008 (38%) |
| None of these/Other | 74 (2.8%) |
| (Missing) | 3 (0.1%) |
| **Religious service attendance** |  |
| More than 1/week | 414 (16%) |
| 1/week | 891 (34%) |
| 1-3/month | 574 (22%) |
| A few times a year | 431 (16%) |
| Never | 334 (13%) |
| (Missing) | 7 (0.3%) |
| **Education** |  |
| Up to 8 years | 668 (25%) |
| 9-15 years | 1,796 (68%) |
| 16+ years | 183 (6.9%) |
| (Missing) | 4 (0.2%) |
| **Immigration** |  |
| Born in this country | 2,511 (95%) |
| Born in another country | 139 (5.2%) |
| (Missing) | 1 (<0.1%) |
| **Religious affiliation** |  |
| Christianity | 2,163 (82%) |
| Islam | 62 (2.3%) |
| Hinduism | 1 (<0.1%) |
| Buddhism | 12 (0.5%) |
| Judaism | 0 (0%) |
| Sikhism | 0 (0%) |
| Baha'i | 0 (0%) |
| Jainism | 2 (<0.1%) |
| Shinto | 2 (<0.1%) |
| Taoism | 1 (<0.1%) |
| Confucianism | 0 (0%) |
| Primal, Animist, or Folk religion | 127 (4.8%) |
| Spiritism | 0 (0%) |
| Umbanda, Candomble, and other African-derived religions | 0 (0%) |
| Chinese folk/traditional religion | 0 (0%) |
| Some other religion | 5 (0.2%) |
| No religion/Atheist/Agnostic | 253 (9.6%) |
| (Missing) | 23 (0.9%) |
| **Race/Ethnicity** |  |
| Asian/Indian | 6 (0.2%) |
| Black | 2,381 (90%) |
| Colored | 252 (9.5%) |
| Other | 1 (<0.1%) |
| White | 8 (0.3%) |
| (Missing) | 3 (0.1%) |
| ^1^n (%) | |

# Table S16b. Means by demographic category for South Africa

| Variable | Category | Mean | 95% CI | SE | Global p-value |
| --- | --- | --- | --- | --- | --- |
| Age group | 18-24 | 1.09 | (0.71, 1.47) | 0.19 | < .001 |
|  | 25-34 | 1.56 | (1.27, 1.85) | 0.15 |  |
|  | 35-44 | 2.39 | (1.40, 3.39) | 0.50 |  |
|  | 45-54 | 1.98 | (1.27, 2.69) | 0.36 |  |
|  | 55-64 | 1.99 | (0.83, 3.15) | 0.59 |  |
|  | 65-74 | 0.57 | (0.00, 1.38) | 0.39 |  |
|  | 75-84 | 0.92 | (0.05, 1.79) | 0.43 |  |
|  | 85 or older | 0.00 | * | * |  |
| Gender | Male | 2.52 | (2.12, 2.93) | 0.21 | < .001 |
|  | Female | 0.91 | (0.45, 1.37) | 0.23 |  |
|  | Other | 4.02 | * | * |  |
| Marital status | Married | 1.87 | (1.31, 2.43) | 0.28 | < .001 |
|  | Separated | 3.32 | (0.98, 5.66) | 1.17 |  |
|  | Divorced | 2.27 | (0.74, 3.80) | 0.76 |  |
|  | Widowed | 0.70 | (0.00, 1.72) | 0.48 |  |
|  | Never | 1.40 | (1.14, 1.66) | 0.13 |  |
|  | Domestic Partner | 3.07 | (0.95, 5.19) | 1.07 |  |
| Employment | Employed for an employer | 1.97 | (1.43, 2.50) | 0.27 | 0.002 |
|  | Self-employed | 2.08 | (1.40, 2.76) | 0.35 |  |
|  | Retired | 1.05 | (0.39, 1.71) | 0.33 |  |
|  | Student | 0.97 | (0.37, 1.57) | 0.31 |  |
|  | Homemaker | 1.04 | (0.32, 1.76) | 0.37 |  |
|  | Unemployed and looking for a job | 1.76 | (1.14, 2.39) | 0.32 |  |
|  | None of these/Other | 1.99 | (0.00, 4.54) | 1.28 |  |
| Religious service attendance | More than 1/week | 0.97 | (0.53, 1.42) | 0.23 | < .001 |
|  | 1/week | 1.63 | (0.75, 2.52) | 0.45 |  |
|  | 1-3/month | 1.37 | (1.00, 1.73) | 0.19 |  |
|  | A few times a year | 2.07 | (1.57, 2.56) | 0.25 |  |
|  | Never | 2.87 | (2.06, 3.68) | 0.41 |  |
| Education | Up to 8 years | 1.75 | (0.54, 2.96) | 0.62 | 0.839 |
|  | 9-15 years | 1.70 | (1.41, 1.98) | 0.15 |  |
|  | 16+ years | 1.50 | (0.83, 2.17) | 0.34 |  |
| Immigration status | Born in this country | 1.73 | (1.36, 2.11) | 0.19 | 0.116 |
|  | Born in another country | 1.06 | (0.30, 1.82) | 0.38 |  |
| Religious affiliation | Christianity | 1.61 | (1.19, 2.02) | 0.21 | < .001 |
|  | Islam | 4.41 | (2.67, 6.15) | 0.87 |  |
|  | Hinduism | 0.00 | * | * |  |
|  | Buddhism | 1.56 | (0.00, 14.63) | 1.11 |  |
|  | Jainism | 5.00 | * | * |  |
|  | Shinto | 0.00 | * | * |  |
|  | Taoism | 0.00 | * | * |  |
|  | Primal, Animist, or Folk religion | 1.23 | (0.60, 1.85) | 0.32 |  |
|  | Some other religion | 6.85 | * | * |  |
|  | No religion/Atheist/Agnostic | 1.98 | (1.41, 2.55) | 0.29 |  |
| Race/Ethnicity | Black | 1.30 | (1.11, 1.50) | 0.10 | < .001 |
|  | White | 2.56 | (0.00, 16.39) | 1.88 |  |
|  | Asian/Indian | 0.00 | * | * |  |
|  | Colored | 5.44 | (2.74, 8.14) | 1.37 |  |
|  | Other | 0.00 | * | * |  |

# Table S17a. Nationally representative descriptive statistics for Spain

| **Characteristic** | **N = 6,290**^1^ |
| --- | --- |
| **Age group** |  |
| 18-24 | 594 (9.4%) |
| 25-34 | 949 (15%) |
| 35-44 | 1,278 (20%) |
| 45-54 | 1,354 (22%) |
| 55-64 | 1,096 (17%) |
| 65-74 | 855 (14%) |
| 75-84 | 162 (2.6%) |
| 85 or older | 3 (<0.1%) |
| (Missing) | 0 (0%) |
| **Gender** |  |
| Male | 3,142 (50%) |
| Female | 3,119 (50%) |
| Other | 6 (0.1%) |
| (Missing) | 22 (0.4%) |
| **Marital status** |  |
| Married | 2,947 (47%) |
| Separated | 237 (3.8%) |
| Divorced | 518 (8.2%) |
| Widowed | 189 (3.0%) |
| Single, never married | 1,742 (28%) |
| Domestic Partner | 589 (9.4%) |
| (Missing) | 67 (1.1%) |
| **Employment** |  |
| Employed for an employer | 2,862 (45%) |
| Self-employed | 576 (9.2%) |
| Retired | 1,278 (20%) |
| Student | 448 (7.1%) |
| Homemaker | 345 (5.5%) |
| Unemployed and looking for a job | 646 (10%) |
| None of these/Other | 123 (2.0%) |
| (Missing) | 11 (0.2%) |
| **Religious service attendance** |  |
| More than 1/week | 317 (5.0%) |
| 1/week | 662 (11%) |
| 1-3/month | 437 (6.9%) |
| A few times a year | 1,972 (31%) |
| Never | 2,875 (46%) |
| (Missing) | 27 (0.4%) |
| **Education** |  |
| Up to 8 years | 802 (13%) |
| 9-15 years | 4,145 (66%) |
| 16+ years | 1,341 (21%) |
| (Missing) | 2 (<0.1%) |
| **Immigration** |  |
| Born in this country | 5,479 (87%) |
| Born in another country | 788 (13%) |
| (Missing) | 23 (0.4%) |
| **Religious affiliation** |  |
| Christianity | 4,074 (65%) |
| Islam | 135 (2.1%) |
| Hinduism | 7 (0.1%) |
| Buddhism | 36 (0.6%) |
| Judaism | 4 (<0.1%) |
| Sikhism | 3 (<0.1%) |
| Baha'i | 2 (<0.1%) |
| Jainism | 1 (<0.1%) |
| Shinto | 0 (0%) |
| Taoism | 5 (<0.1%) |
| Confucianism | 3 (<0.1%) |
| Primal, Animist, or Folk religion | 7 (0.1%) |
| Spiritism | 0 (0%) |
| Umbanda, Candomble, and other African-derived religions | 0 (0%) |
| Chinese folk/traditional religion | 0 (0%) |
| Some other religion | 27 (0.4%) |
| No religion/Atheist/Agnostic | 1,932 (31%) |
| (Missing) | 55 (0.9%) |
| ^1^n (%) | |

# Table S17b. Means by demographic category for Spain

| Variable | Category | Mean | 95% CI | SE | Global p-value |
| --- | --- | --- | --- | --- | --- |
| Age group | 18-24 | 1.92 | (1.50, 2.33) | 0.21 | < .001 |
|  | 25-34 | 2.98 | (2.52, 3.43) | 0.23 |  |
|  | 35-44 | 4.28 | (3.81, 4.75) | 0.24 |  |
|  | 45-54 | 4.21 | (3.76, 4.65) | 0.23 |  |
|  | 55-64 | 4.13 | (3.52, 4.75) | 0.31 |  |
|  | 65-74 | 3.16 | (2.29, 4.03) | 0.44 |  |
|  | 75-84 | 1.83 | (0.85, 2.82) | 0.50 |  |
|  | 85 or older | 6.17 | * | * |  |
| Gender | Male | 4.19 | (3.82, 4.56) | 0.19 | < .001 |
|  | Female | 3.01 | (2.75, 3.28) | 0.13 |  |
|  | Other | 5.15 | (0.00, 15.20) | 1.94 |  |
| Marital status | Married | 3.44 | (3.11, 3.77) | 0.17 | 0.148 |
|  | Separated | 3.97 | (3.02, 4.91) | 0.48 |  |
|  | Divorced | 4.58 | (3.71, 5.46) | 0.44 |  |
|  | Widowed | 3.80 | (2.44, 5.16) | 0.69 |  |
|  | Never | 3.41 | (3.01, 3.82) | 0.21 |  |
|  | Domestic Partner | 3.92 | (3.12, 4.73) | 0.41 |  |
| Employment | Employed for an employer | 3.73 | (3.44, 4.03) | 0.15 | < .001 |
|  | Self-employed | 4.31 | (3.59, 5.03) | 0.37 |  |
|  | Retired | 3.35 | (2.69, 4.01) | 0.34 |  |
|  | Student | 1.91 | (1.31, 2.50) | 0.30 |  |
|  | Homemaker | 3.03 | (2.11, 3.94) | 0.46 |  |
|  | Unemployed and looking for a job | 4.24 | (3.60, 4.88) | 0.33 |  |
|  | None of these/Other | 4.55 | (2.56, 6.54) | 1.00 |  |
| Religious service attendance | More than 1/week | 2.00 | (1.38, 2.63) | 0.32 | < .001 |
|  | 1/week | 3.62 | (2.94, 4.31) | 0.35 |  |
|  | 1-3/month | 4.25 | (3.14, 5.35) | 0.56 |  |
|  | A few times a year | 3.47 | (3.09, 3.85) | 0.19 |  |
|  | Never | 3.77 | (3.43, 4.11) | 0.17 |  |
| Education | Up to 8 years | 4.12 | (3.31, 4.93) | 0.41 | 0.001 |
|  | 9-15 years | 3.74 | (3.47, 4.01) | 0.14 |  |
|  | 16+ years | 2.87 | (2.43, 3.32) | 0.23 |  |
| Immigration status | Born in this country | 3.79 | (3.54, 4.04) | 0.13 | < .001 |
|  | Born in another country | 2.33 | (1.88, 2.78) | 0.23 |  |
| Religious affiliation | Christianity | 3.69 | (3.41, 3.98) | 0.15 | < .001 |
|  | Islam | 2.49 | (1.46, 3.51) | 0.52 |  |
|  | Hinduism | 5.22 | * | * |  |
|  | Buddhism | 2.31 | (0.91, 3.71) | 0.67 |  |
|  | Judaism | 6.41 | * | * |  |
|  | Sikhism | 14.47 | * | * |  |
|  | Baha'i | 6.00 | * | * |  |
|  | Jainism | 0.00 | * | * |  |
|  | Taoism | 2.71 | * | * |  |
|  | Confucianism | 17.40 | * | * |  |
|  | Primal, Animist, or Folk religion | 3.93 | * | * |  |
|  | Some other religion | 5.80 | (2.13, 9.46) | 1.67 |  |
|  | No religion/Atheist/Agnostic | 3.44 | (3.05, 3.84) | 0.20 |  |

# Table S18a. Nationally representative descriptive statistics for Sweden

| **Characteristic** | **N = 15,068**^1^ |
| --- | --- |
| **Age group** |  |
| 18-24 | 1,515 (10%) |
| 25-34 | 2,636 (17%) |
| 35-44 | 2,358 (16%) |
| 45-54 | 2,406 (16%) |
| 55-64 | 2,282 (15%) |
| 65-74 | 2,146 (14%) |
| 75-84 | 1,605 (11%) |
| 85 or older | 122 (0.8%) |
| (Missing) | 0 (0%) |
| **Gender** |  |
| Male | 7,536 (50%) |
| Female | 7,493 (50%) |
| Other | 27 (0.2%) |
| (Missing) | 12 (<0.1%) |
| **Marital status** |  |
| Married | 6,408 (43%) |
| Separated | 426 (2.8%) |
| Divorced | 801 (5.3%) |
| Widowed | 433 (2.9%) |
| Single, never married | 3,854 (26%) |
| Domestic Partner | 3,073 (20%) |
| (Missing) | 72 (0.5%) |
| **Employment** |  |
| Employed for an employer | 7,907 (52%) |
| Self-employed | 1,243 (8.3%) |
| Retired | 3,832 (25%) |
| Student | 1,332 (8.8%) |
| Homemaker | 75 (0.5%) |
| Unemployed and looking for a job | 324 (2.2%) |
| None of these/Other | 337 (2.2%) |
| (Missing) | 18 (0.1%) |
| **Religious service attendance** |  |
| More than 1/week | 236 (1.6%) |
| 1/week | 434 (2.9%) |
| 1-3/month | 486 (3.2%) |
| A few times a year | 3,950 (26%) |
| Never | 9,918 (66%) |
| (Missing) | 45 (0.3%) |
| **Education** |  |
| Up to 8 years | 252 (1.7%) |
| 9-15 years | 10,790 (72%) |
| 16+ years | 4,026 (27%) |
| (Missing) | 0 (0%) |
| **Immigration** |  |
| Born in this country | 13,922 (92%) |
| Born in another country | 1,052 (7.0%) |
| (Missing) | 94 (0.6%) |
| **Religious affiliation** |  |
| Christianity | 8,346 (55%) |
| Islam | 470 (3.1%) |
| Hinduism | 22 (0.1%) |
| Buddhism | 110 (0.7%) |
| Judaism | 54 (0.4%) |
| Sikhism | 4 (<0.1%) |
| Baha'i | 6 (<0.1%) |
| Jainism | 0 (0%) |
| Shinto | 0 (<0.1%) |
| Taoism | 4 (<0.1%) |
| Confucianism | 0 (0%) |
| Primal, Animist, or Folk religion | 83 (0.5%) |
| Spiritism | 0 (0%) |
| Umbanda, Candomble, and other African-derived religions | 0 (0%) |
| Chinese folk/traditional religion | 0 (0%) |
| Some other religion | 198 (1.3%) |
| No religion/Atheist/Agnostic | 5,697 (38%) |
| (Missing) | 74 (0.5%) |
| ^1^n (%) | |

# Table S18b. Means by demographic category for Sweden

| Variable | Category | Mean | 95% CI | SE | Global p-value |
| --- | --- | --- | --- | --- | --- |
| Age group | 18-24 | 0.51 | (0.36, 0.67) | 0.08 | < .001 |
|  | 25-34 | 0.81 | (0.66, 0.96) | 0.08 |  |
|  | 35-44 | 0.92 | (0.74, 1.10) | 0.09 |  |
|  | 45-54 | 0.96 | (0.78, 1.13) | 0.09 |  |
|  | 55-64 | 1.05 | (0.88, 1.22) | 0.09 |  |
|  | 65-74 | 1.02 | (0.80, 1.24) | 0.11 |  |
|  | 75-84 | 0.46 | (0.32, 0.60) | 0.07 |  |
|  | 85 or older | 0.10 | (0.00, 0.26) | 0.08 |  |
| Gender | Male | 0.74 | (0.65, 0.84) | 0.05 | 0.009 |
|  | Female | 0.94 | (0.85, 1.04) | 0.05 |  |
|  | Other | 1.13 | (0.00, 2.73) | 0.77 |  |
| Marital status | Married | 0.61 | (0.51, 0.70) | 0.05 | < .001 |
|  | Separated | 1.35 | (0.80, 1.89) | 0.28 |  |
|  | Divorced | 1.76 | (1.34, 2.17) | 0.21 |  |
|  | Widowed | 0.64 | (0.40, 0.89) | 0.13 |  |
|  | Never | 0.96 | (0.82, 1.11) | 0.07 |  |
|  | Domestic Partner | 0.91 | (0.78, 1.05) | 0.07 |  |
| Employment | Employed for an employer | 0.79 | (0.71, 0.87) | 0.04 | < .001 |
|  | Self-employed | 1.05 | (0.76, 1.35) | 0.15 |  |
|  | Retired | 0.83 | (0.69, 0.97) | 0.07 |  |
|  | Student | 0.31 | (0.22, 0.41) | 0.05 |  |
|  | Homemaker | 2.61 | (0.49, 4.72) | 1.06 |  |
|  | Unemployed and looking for a job | 1.99 | (1.46, 2.51) | 0.27 |  |
|  | None of these/Other | 2.12 | (1.35, 2.89) | 0.39 |  |
| Religious service attendance | More than 1/week | 0.56 | (0.20, 0.92) | 0.18 | 0.226 |
|  | 1/week | 0.84 | (0.52, 1.15) | 0.16 |  |
|  | 1-3/month | 0.68 | (0.42, 0.95) | 0.13 |  |
|  | A few times a year | 0.79 | (0.65, 0.93) | 0.07 |  |
|  | Never | 0.88 | (0.80, 0.96) | 0.04 |  |
| Education | Up to 8 years | 0.93 | (0.29, 1.58) | 0.32 | < .001 |
|  | 9-15 years | 0.99 | (0.90, 1.07) | 0.04 |  |
|  | 16+ years | 0.45 | (0.36, 0.54) | 0.05 |  |
| Immigration status | Born in this country | 0.80 | (0.74, 0.87) | 0.03 | < .001 |
|  | Born in another country | 1.37 | (1.03, 1.70) | 0.17 |  |
| Religious affiliation | Christianity | 0.78 | (0.69, 0.87) | 0.05 | < .001 |
|  | Islam | 2.20 | (1.46, 2.94) | 0.38 |  |
|  | Hinduism | 0.59 | (0.00, 2.15) | 0.62 |  |
|  | Buddhism | 1.01 | (0.16, 1.86) | 0.43 |  |
|  | Judaism | 0.85 | (0.00, 2.01) | 0.57 |  |
|  | Sikhism | 0.00 | * | * |  |
|  | Baha'i | 2.90 | * | * |  |
|  | Shinto | 2.00 | * | * |  |
|  | Taoism | 3.51 | * | * |  |
|  | Primal, Animist, or Folk religion | 1.80 | (0.00, 3.81) | 1.01 |  |
|  | Some other religion | 0.89 | (0.38, 1.41) | 0.26 |  |
|  | No religion/Atheist/Agnostic | 0.81 | (0.72, 0.90) | 0.05 |  |

# Table S19a. Nationally representative descriptive statistics for Tanzania

| **Characteristic** | **N = 9,075**^1^ |
| --- | --- |
| **Age group** |  |
| 18-24 | 2,284 (25%) |
| 25-34 | 2,515 (28%) |
| 35-44 | 1,719 (19%) |
| 45-54 | 1,206 (13%) |
| 55-64 | 700 (7.7%) |
| 65-74 | 460 (5.1%) |
| 75-84 | 153 (1.7%) |
| 85 or older | 35 (0.4%) |
| (Missing) | 2 (<0.1%) |
| **Gender** |  |
| Male | 4,299 (47%) |
| Female | 4,776 (53%) |
| Other | 0 (0%) |
| (Missing) | 0 (0%) |
| **Marital status** |  |
| Married | 5,577 (61%) |
| Separated | 404 (4.5%) |
| Divorced | 103 (1.1%) |
| Widowed | 450 (5.0%) |
| Single, never married | 2,260 (25%) |
| Domestic Partner | 275 (3.0%) |
| (Missing) | 7 (<0.1%) |
| **Employment** |  |
| Employed for an employer | 513 (5.6%) |
| Self-employed | 4,625 (51%) |
| Retired | 139 (1.5%) |
| Student | 319 (3.5%) |
| Homemaker | 1,796 (20%) |
| Unemployed and looking for a job | 1,491 (16%) |
| None of these/Other | 186 (2.1%) |
| (Missing) | 6 (<0.1%) |
| **Religious service attendance** |  |
| More than 1/week | 2,622 (29%) |
| 1/week | 4,268 (47%) |
| 1-3/month | 1,082 (12%) |
| A few times a year | 814 (9.0%) |
| Never | 288 (3.2%) |
| (Missing) | 1 (<0.1%) |
| **Education** |  |
| Up to 8 years | 6,699 (74%) |
| 9-15 years | 2,252 (25%) |
| 16+ years | 122 (1.3%) |
| (Missing) | 2 (<0.1%) |
| **Immigration** |  |
| Born in this country | 9,048 (100%) |
| Born in another country | 25 (0.3%) |
| (Missing) | 1 (<0.1%) |
| **Religious affiliation** |  |
| Christianity | 5,647 (62%) |
| Islam | 3,189 (35%) |
| Hinduism | 0 (0%) |
| Buddhism | 0 (0%) |
| Judaism | 0 (0%) |
| Sikhism | 0 (0%) |
| Baha'i | 0 (0%) |
| Jainism | 0 (0%) |
| Shinto | 0 (0%) |
| Taoism | 1 (<0.1%) |
| Confucianism | 0 (0%) |
| Primal, Animist, or Folk religion | 12 (0.1%) |
| Spiritism | 0 (0%) |
| Umbanda, Candomble, and other African-derived religions | 0 (0%) |
| Chinese folk/traditional religion | 0 (0%) |
| Some other religion | 0 (0%) |
| No religion/Atheist/Agnostic | 216 (2.4%) |
| (Missing) | 10 (0.1%) |
| **Race/Ethnicity** |  |
| African | 9,060 (100%) |
| Arab | 11 (0.1%) |
| Indian | 3 (<0.1%) |
| (Missing) | 2 (<0.1%) |
| ^1^n (%) | |

# Table S19b. Means by demographic category for Tanzania

| Variable | Category | Mean | 95% CI | SE | Global p-value |
| --- | --- | --- | --- | --- | --- |
| Age group | 18-24 | 0.08 | (0.04, 0.13) | 0.02 | < .001 |
|  | 25-34 | 0.21 | (0.14, 0.28) | 0.04 |  |
|  | 35-44 | 0.31 | (0.21, 0.41) | 0.05 |  |
|  | 45-54 | 0.31 | (0.21, 0.40) | 0.05 |  |
|  | 55-64 | 0.52 | (0.14, 0.91) | 0.20 |  |
|  | 65-74 | 0.21 | (0.06, 0.37) | 0.08 |  |
|  | 75-84 | 0.27 | (0.00, 0.69) | 0.21 |  |
|  | 85 or older | 0.00 | * | * |  |
| Gender | Male | 0.44 | (0.37, 0.52) | 0.04 | < .001 |
|  | Female | 0.05 | (0.00, 0.10) | 0.03 |  |
| Marital status | Married | 0.26 | (0.19, 0.33) | 0.04 | < .001 |
|  | Separated | 0.51 | (0.26, 0.76) | 0.12 |  |
|  | Divorced | 0.31 | (0.00, 0.66) | 0.18 |  |
|  | Widowed | 0.07 | (0.01, 0.12) | 0.03 |  |
|  | Never | 0.17 | (0.10, 0.24) | 0.04 |  |
|  | Domestic Partner | 0.17 | (0.05, 0.30) | 0.06 |  |
| Employment | Employed for an employer | 0.18 | (0.08, 0.28) | 0.05 | < .001 |
|  | Self-employed | 0.35 | (0.26, 0.43) | 0.04 |  |
|  | Retired | 0.21 | (0.04, 0.38) | 0.08 |  |
|  | Student | 0.01 | (0.00, 0.02) | 0.01 |  |
|  | Homemaker | 0.02 | (0.00, 0.05) | 0.01 |  |
|  | Unemployed and looking for a job | 0.21 | (0.12, 0.30) | 0.05 |  |
|  | None of these/Other | 0.34 | (0.08, 0.59) | 0.13 |  |
| Religious service attendance | More than 1/week | 0.24 | (0.13, 0.36) | 0.06 | 0.009 |
|  | 1/week | 0.17 | (0.12, 0.21) | 0.02 |  |
|  | 1-3/month | 0.29 | (0.18, 0.40) | 0.06 |  |
|  | A few times a year | 0.36 | (0.21, 0.51) | 0.08 |  |
|  | Never | 0.57 | (0.23, 0.92) | 0.17 |  |
| Education | Up to 8 years | 0.26 | (0.20, 0.32) | 0.03 | 0.012 |
|  | 9-15 years | 0.17 | (0.12, 0.23) | 0.03 |  |
|  | 16+ years | 0.09 | (0.00, 0.21) | 0.06 |  |
| Immigration status | Born in this country | 0.24 | (0.19, 0.28) | 0.02 | 0.018 |
|  | Born in another country | 0.07 | (0.00, 0.20) | 0.07 |  |
| Religious affiliation | Christianity | 0.16 | (0.12, 0.20) | 0.02 | < .001 |
|  | Islam | 0.38 | (0.26, 0.49) | 0.06 |  |
|  | Taoism | 0.00 | * | * |  |
|  | Primal, Animist, or Folk religion | 0.00 | * | * |  |
|  | No religion/Atheist/Agnostic | 0.14 | (0.05, 0.24) | 0.05 |  |
| Race/Ethnicity | Indian | 0.00 | * | * | 0.477 |
|  | Arab | 0.00 | * | * |  |
|  | African | 0.24 | (0.19, 0.28) | 0.02 |  |

# Table S20a. Nationally representative descriptive statistics for Türkiye

| **Characteristic** | **N = 1,473**^1^ |
| --- | --- |
| **Age group** |  |
| 18-24 | 222 (15%) |
| 25-34 | 312 (21%) |
| 35-44 | 318 (22%) |
| 45-54 | 262 (18%) |
| 55-64 | 200 (14%) |
| 65-74 | 120 (8.2%) |
| 75-84 | 35 (2.4%) |
| 85 or older | 3 (0.2%) |
| (Missing) | 0 (0%) |
| **Gender** |  |
| Male | 754 (51%) |
| Female | 719 (49%) |
| Other | 0 (0%) |
| (Missing) | 0 (0%) |
| **Marital status** |  |
| Married | 936 (64%) |
| Separated | 13 (0.9%) |
| Divorced | 64 (4.3%) |
| Widowed | 64 (4.3%) |
| Single, never married | 379 (26%) |
| Domestic Partner | 0 (0%) |
| (Missing) | 17 (1.1%) |
| **Employment** |  |
| Employed for an employer | 413 (28%) |
| Self-employed | 255 (17%) |
| Retired | 205 (14%) |
| Student | 107 (7.3%) |
| Homemaker | 347 (24%) |
| Unemployed and looking for a job | 87 (5.9%) |
| None of these/Other | 59 (4.0%) |
| (Missing) | 0 (0%) |
| **Religious service attendance** |  |
| More than 1/week | 493 (33%) |
| 1/week | 271 (18%) |
| 1-3/month | 174 (12%) |
| A few times a year | 255 (17%) |
| Never | 274 (19%) |
| (Missing) | 6 (0.4%) |
| **Education** |  |
| Up to 8 years | 436 (30%) |
| 9-15 years | 711 (48%) |
| 16+ years | 326 (22%) |
| (Missing) | 0 (0%) |
| **Immigration** |  |
| Born in this country | 1,415 (96%) |
| Born in another country | 58 (4.0%) |
| (Missing) | 0 (0%) |
| **Religious affiliation** |  |
| Christianity | 2 (0.1%) |
| Islam | 1,381 (94%) |
| Hinduism | 0 (0%) |
| Buddhism | 1 (<0.1%) |
| Judaism | 1 (<0.1%) |
| Sikhism | 1 (<0.1%) |
| Baha'i | 0 (0%) |
| Jainism | 0 (0%) |
| Shinto | 0 (0%) |
| Taoism | 0 (0%) |
| Confucianism | 0 (0%) |
| Primal, Animist, or Folk religion | 1 (<0.1%) |
| Spiritism | 0 (0%) |
| Umbanda, Candomble, and other African-derived religions | 0 (0%) |
| Chinese folk/traditional religion | 0 (0%) |
| Some other religion | 1 (<0.1%) |
| No religion/Atheist/Agnostic | 66 (4.5%) |
| (Missing) | 19 (1.3%) |
| **Race/Ethnicity** |  |
| Albanian | 8 (0.5%) |
| Arab | 51 (3.5%) |
| Armenian | 1 (<0.1%) |
| Azeri | 9 (0.6%) |
| Bosnian | 5 (0.3%) |
| Circassian | 19 (1.3%) |
| Georgian | 4 (0.3%) |
| Greek | 1 (<0.1%) |
| Kurdish/Zaza | 252 (17%) |
| Laz | 25 (1.7%) |
| Other | 58 (3.9%) |
| Turkish | 1,030 (70%) |
| Uyghur | 1 (<0.1%) |
| (Missing) | 9 (0.6%) |
| ^1^n (%) | |

# Table S20b. Means by demographic category for Türkiye

| Variable | Category | Mean | 95% CI | SE | Global p-value |
| --- | --- | --- | --- | --- | --- |
| Age group | 18-24 | 8.73 | (7.01, 10.46) | 0.87 | < .001 |
|  | 25-34 | 10.81 | (9.14, 12.48) | 0.85 |  |
|  | 35-44 | 10.17 | (8.72, 11.62) | 0.74 |  |
|  | 45-54 | 11.17 | (9.13, 13.20) | 1.03 |  |
|  | 55-64 | 9.46 | (7.12, 11.80) | 1.18 |  |
|  | 65-74 | 7.36 | (3.61, 11.11) | 1.89 |  |
|  | 75-84 | 2.91 | (0.22, 5.60) | 1.30 |  |
|  | 85 or older | 20.00 | * | * |  |
| Gender | Male | 13.60 | (12.37, 14.83) | 0.63 | < .001 |
|  | Female | 5.79 | (4.94, 6.64) | 0.43 |  |
| Marital status | Married | 8.77 | (7.83, 9.71) | 0.48 | < .001 |
|  | Separated | 7.85 | (1.11, 14.59) | 2.78 |  |
|  | Divorced | 17.74 | (12.66, 22.83) | 2.54 |  |
|  | Widowed | 6.00 | (1.24, 10.77) | 2.38 |  |
|  | Never | 11.67 | (10.30, 13.04) | 0.70 |  |
| Employment | Employed for an employer | 10.79 | (9.48, 12.11) | 0.67 | < .001 |
|  | Self-employed | 15.21 | (13.11, 17.32) | 1.07 |  |
|  | Retired | 8.70 | (6.32, 11.09) | 1.21 |  |
|  | Student | 5.47 | (3.93, 7.02) | 0.78 |  |
|  | Homemaker | 5.05 | (3.65, 6.45) | 0.71 |  |
|  | Unemployed and looking for a job | 12.94 | (9.92, 15.96) | 1.52 |  |
|  | None of these/Other | 14.11 | (8.40, 19.83) | 2.84 |  |
| Religious service attendance | More than 1/week | 8.57 | (7.13, 10.01) | 0.73 | 0.051 |
|  | 1/week | 9.67 | (7.94, 11.40) | 0.88 |  |
|  | 1-3/month | 8.77 | (6.61, 10.93) | 1.09 |  |
|  | A few times a year | 12.07 | (10.06, 14.08) | 1.02 |  |
|  | Never | 10.61 | (8.93, 12.29) | 0.85 |  |
| Education | Up to 8 years | 9.70 | (7.91, 11.50) | 0.91 | 0.638 |
|  | 9-15 years | 10.06 | (9.01, 11.12) | 0.54 |  |
|  | 16+ years | 9.29 | (8.05, 10.53) | 0.63 |  |
| Immigration status | Born in this country | 9.74 | (8.95, 10.54) | 0.41 | 0.690 |
|  | Born in another country | 10.85 | (5.36, 16.34) | 2.74 |  |
| Religious affiliation | Christianity | 4.05 | * | * | < .001 |
|  | Islam | 9.59 | (8.77, 10.41) | 0.42 |  |
|  | Buddhism | 0.00 | * | * |  |
|  | Judaism | 20.00 | * | * |  |
|  | Sikhism | 0.00 | * | * |  |
|  | Primal, Animist, or Folk religion | 16.04 | * | * |  |
|  | Some other religion | 25.81 | * | * |  |
|  | No religion/Atheist/Agnostic | 13.65 | (10.52, 16.78) | 1.56 |  |
| Race/Ethnicity | Arab | 7.74 | (3.49, 12.00) | 2.10 | < .001 |
|  | Turkish | 9.25 | (8.34, 10.15) | 0.46 |  |
|  | Greek | 12.84 | * | * |  |
|  | Kurdish/Zaza | 12.02 | (9.77, 14.28) | 1.14 |  |
|  | Laz | 7.13 | (1.98, 12.28) | 2.31 |  |
|  | Circassian | 13.79 | (5.01, 22.57) | 3.19 |  |
|  | Bosnian | 6.10 | * | * |  |
|  | Armenian | 0.00 | * | * |  |
|  | Georgian | 9.69 | * | * |  |
|  | Uyghur | 4.00 | * | * |  |
|  | Albanian | 25.08 | * | * |  |
|  | Azeri | 9.83 | * | * |  |
|  | Other | 9.97 | (5.00, 14.95) | 2.47 |  |

# Table S21a. Nationally representative descriptive statistics for United Kingdom

| **Characteristic** | **N = 5,368**^1^ |
| --- | --- |
| **Age group** |  |
| 18-24 | 490 (9.1%) |
| 25-34 | 911 (17%) |
| 35-44 | 901 (17%) |
| 45-54 | 901 (17%) |
| 55-64 | 864 (16%) |
| 65-74 | 838 (16%) |
| 75-84 | 417 (7.8%) |
| 85 or older | 46 (0.9%) |
| (Missing) | 1 (<0.1%) |
| **Gender** |  |
| Male | 2,557 (48%) |
| Female | 2,789 (52%) |
| Other | 14 (0.3%) |
| (Missing) | 9 (0.2%) |
| **Marital status** |  |
| Married | 2,510 (47%) |
| Separated | 114 (2.1%) |
| Divorced | 435 (8.1%) |
| Widowed | 294 (5.5%) |
| Single, never married | 1,456 (27%) |
| Domestic Partner | 512 (9.5%) |
| (Missing) | 48 (0.9%) |
| **Employment** |  |
| Employed for an employer | 2,798 (52%) |
| Self-employed | 469 (8.7%) |
| Retired | 1,262 (24%) |
| Student | 229 (4.3%) |
| Homemaker | 184 (3.4%) |
| Unemployed and looking for a job | 215 (4.0%) |
| None of these/Other | 201 (3.7%) |
| (Missing) | 11 (0.2%) |
| **Religious service attendance** |  |
| More than 1/week | 291 (5.4%) |
| 1/week | 499 (9.3%) |
| 1-3/month | 293 (5.5%) |
| A few times a year | 1,165 (22%) |
| Never | 3,110 (58%) |
| (Missing) | 10 (0.2%) |
| **Education** |  |
| Up to 8 years | 1,314 (24%) |
| 9-15 years | 2,072 (39%) |
| 16+ years | 1,974 (37%) |
| (Missing) | 8 (0.2%) |
| **Immigration** |  |
| Born in this country | 4,659 (87%) |
| Born in another country | 682 (13%) |
| (Missing) | 27 (0.5%) |
| **Religious affiliation** |  |
| Christianity | 2,750 (51%) |
| Islam | 218 (4.1%) |
| Hinduism | 61 (1.1%) |
| Buddhism | 30 (0.6%) |
| Judaism | 44 (0.8%) |
| Sikhism | 29 (0.5%) |
| Baha'i | 6 (0.1%) |
| Jainism | 4 (<0.1%) |
| Shinto | 0 (0%) |
| Taoism | 4 (<0.1%) |
| Confucianism | 2 (<0.1%) |
| Primal, Animist, or Folk religion | 36 (0.7%) |
| Spiritism | 0 (0%) |
| Umbanda, Candomble, and other African-derived religions | 0 (0%) |
| Chinese folk/traditional religion | 0 (0%) |
| Some other religion | 61 (1.1%) |
| No religion/Atheist/Agnostic | 2,099 (39%) |
| (Missing) | 25 (0.5%) |
| **Race/Ethnicity** |  |
| Asian | 426 (7.9%) |
| Black | 152 (2.8%) |
| Other | 96 (1.8%) |
| White | 4,647 (87%) |
| (Missing) | 47 (0.9%) |
| ^1^n (%) | |

# Table S21b. Means by demographic category for United Kingdom

| Variable | Category | Mean | 95% CI | SE | Global p-value |
| --- | --- | --- | --- | --- | --- |
| Age group | 18-24 | 0.46 | (0.17, 0.74) | 0.15 | < .001 |
|  | 25-34 | 1.67 | (1.33, 2.02) | 0.18 |  |
|  | 35-44 | 2.29 | (1.73, 2.85) | 0.29 |  |
|  | 45-54 | 2.04 | (1.52, 2.55) | 0.26 |  |
|  | 55-64 | 2.23 | (1.72, 2.74) | 0.26 |  |
|  | 65-74 | 1.87 | (1.38, 2.36) | 0.25 |  |
|  | 75-84 | 0.71 | (0.23, 1.18) | 0.24 |  |
|  | 85 or older | 0.26 | (0.00, 0.66) | 0.20 |  |
| Gender | Male | 1.97 | (1.69, 2.24) | 0.14 | 0.114 |
|  | Female | 1.57 | (1.31, 1.82) | 0.13 |  |
|  | Other | 1.79 | (0.00, 4.54) | 1.21 |  |
| Marital status | Married | 1.55 | (1.32, 1.79) | 0.12 | 0.006 |
|  | Separated | 1.01 | (0.30, 1.73) | 0.35 |  |
|  | Divorced | 2.45 | (1.67, 3.23) | 0.40 |  |
|  | Widowed | 2.37 | (1.30, 3.45) | 0.55 |  |
|  | Never | 1.57 | (1.23, 1.92) | 0.18 |  |
|  | Domestic Partner | 2.53 | (1.72, 3.35) | 0.42 |  |
| Employment | Employed for an employer | 1.74 | (1.49, 1.98) | 0.12 | < .001 |
|  | Self-employed | 1.87 | (1.26, 2.49) | 0.31 |  |
|  | Retired | 1.33 | (1.01, 1.66) | 0.17 |  |
|  | Student | 0.50 | (0.09, 0.91) | 0.21 |  |
|  | Homemaker | 2.96 | (1.17, 4.75) | 0.91 |  |
|  | Unemployed and looking for a job | 2.54 | (1.44, 3.63) | 0.56 |  |
|  | None of these/Other | 4.00 | (2.40, 5.60) | 0.81 |  |
| Religious service attendance | More than 1/week | 1.44 | (0.89, 2.00) | 0.28 | < .001 |
|  | 1/week | 2.23 | (1.58, 2.89) | 0.33 |  |
|  | 1-3/month | 1.79 | (1.16, 2.41) | 0.32 |  |
|  | A few times a year | 1.12 | (0.85, 1.39) | 0.14 |  |
|  | Never | 1.95 | (1.68, 2.23) | 0.14 |  |
| Education | Up to 8 years | 2.00 | (1.43, 2.57) | 0.29 | < .001 |
|  | 9-15 years | 1.96 | (1.70, 2.23) | 0.14 |  |
|  | 16+ years | 1.38 | (1.20, 1.57) | 0.10 |  |
| Immigration status | Born in this country | 1.84 | (1.64, 2.05) | 0.11 | 0.002 |
|  | Born in another country | 1.19 | (0.82, 1.55) | 0.19 |  |
| Religious affiliation | Christianity | 1.77 | (1.52, 2.03) | 0.13 | < .001 |
|  | Islam | 0.84 | (0.36, 1.32) | 0.24 |  |
|  | Hinduism | 0.49 | (0.07, 0.91) | 0.21 |  |
|  | Buddhism | 2.74 | (0.82, 4.66) | 0.90 |  |
|  | Judaism | 0.58 | (0.00, 1.53) | 0.46 |  |
|  | Sikhism | 0.63 | (0.00, 1.79) | 0.54 |  |
|  | Baha'i | 24.27 | * | * |  |
|  | Jainism | 5.00 | * | * |  |
|  | Taoism | 0.12 | * | * |  |
|  | Confucianism | 0.00 | * | * |  |
|  | Primal, Animist, or Folk religion | 0.93 | (0.00, 1.92) | 0.48 |  |
|  | Some other religion | 2.75 | (0.00, 5.91) | 1.57 |  |
|  | No religion/Atheist/Agnostic | 1.82 | (1.53, 2.11) | 0.15 |  |
| Race/Ethnicity | Asian | 0.90 | (0.51, 1.30) | 0.20 | < .001 |
|  | Black | 0.89 | (0.42, 1.37) | 0.24 |  |
|  | White | 1.88 | (1.67, 2.09) | 0.11 |  |
|  | Other | 1.29 | (0.29, 2.29) | 0.50 |  |

# Table S22a. Nationally representative descriptive statistics for United States

| **Characteristic** | **N = 38,312**^1^ |
| --- | --- |
| **Age group** |  |
| 18-24 | 2,682 (7.0%) |
| 25-34 | 7,802 (20%) |
| 35-44 | 5,850 (15%) |
| 45-54 | 5,957 (16%) |
| 55-64 | 7,518 (20%) |
| 65-74 | 5,459 (14%) |
| 75-84 | 2,364 (6.2%) |
| 85 or older | 679 (1.8%) |
| (Missing) | 0 (0%) |
| **Gender** |  |
| Male | 18,222 (48%) |
| Female | 19,562 (51%) |
| Other | 392 (1.0%) |
| (Missing) | 136 (0.4%) |
| **Marital status** |  |
| Married | 20,360 (53%) |
| Separated | 727 (1.9%) |
| Divorced | 3,636 (9.5%) |
| Widowed | 1,978 (5.2%) |
| Single, never married | 9,431 (25%) |
| Domestic Partner | 1,971 (5.1%) |
| (Missing) | 207 (0.5%) |
| **Employment** |  |
| Employed for an employer | 19,502 (51%) |
| Self-employed | 3,445 (9.0%) |
| Retired | 9,016 (24%) |
| Student | 1,145 (3.0%) |
| Homemaker | 2,049 (5.3%) |
| Unemployed and looking for a job | 1,777 (4.6%) |
| None of these/Other | 1,292 (3.4%) |
| (Missing) | 87 (0.2%) |
| **Religious service attendance** |  |
| More than 1/week | 2,633 (6.9%) |
| 1/week | 5,887 (15%) |
| 1-3/month | 2,819 (7.4%) |
| A few times a year | 8,870 (23%) |
| Never | 17,975 (47%) |
| (Missing) | 128 (0.3%) |
| **Education** |  |
| Up to 8 years | 210 (0.5%) |
| 9-15 years | 25,322 (66%) |
| 16+ years | 12,705 (33%) |
| (Missing) | 75 (0.2%) |
| **Immigration** |  |
| Born in this country | 34,865 (91%) |
| Born in another country | 3,020 (7.9%) |
| (Missing) | 427 (1.1%) |
| **Religious affiliation** |  |
| Christianity | 22,954 (60%) |
| Islam | 205 (0.5%) |
| Hinduism | 167 (0.4%) |
| Buddhism | 336 (0.9%) |
| Judaism | 638 (1.7%) |
| Sikhism | 24 (<0.1%) |
| Baha'i | 13 (<0.1%) |
| Jainism | 18 (<0.1%) |
| Shinto | 12 (<0.1%) |
| Taoism | 93 (0.2%) |
| Confucianism | 8 (<0.1%) |
| Primal, Animist, or Folk religion | 240 (0.6%) |
| Spiritism | 0 (0%) |
| Umbanda, Candomble, and other African-derived religions | 0 (0%) |
| Chinese folk/traditional religion | 0 (0%) |
| Some other religion | 1,267 (3.3%) |
| No religion/Atheist/Agnostic | 11,870 (31%) |
| (Missing) | 467 (1.2%) |
| **Race/Ethnicity** |  |
| Asian | 2,466 (6.4%) |
| Black | 4,501 (12%) |
| Hispanic | 6,724 (18%) |
| Other | 997 (2.6%) |
| White | 23,605 (62%) |
| (Missing) | 20 (<0.1%) |
| ^1^n (%) | |

# Table S22b. Means by demographic category for United States

| Variable | Category | Mean | 95% CI | SE | Global p-value |
| --- | --- | --- | --- | --- | --- |
| Age group | 18-24 | 0.41 | (0.05, 0.78) | 0.18 | < .001 |
|  | 25-34 | 1.25 | (0.77, 1.73) | 0.24 |  |
|  | 35-44 | 1.37 | (1.11, 1.63) | 0.13 |  |
|  | 45-54 | 1.40 | (1.17, 1.64) | 0.12 |  |
|  | 55-64 | 1.64 | (1.46, 1.83) | 0.10 |  |
|  | 65-74 | 1.29 | (1.12, 1.46) | 0.09 |  |
|  | 75-84 | 0.53 | (0.41, 0.66) | 0.06 |  |
|  | 85 or older | 0.14 | (0.00, 0.33) | 0.09 |  |
| Gender | Male | 1.32 | (1.15, 1.49) | 0.08 | 0.001 |
|  | Female | 1.21 | (1.02, 1.39) | 0.09 |  |
|  | Other | 0.47 | (0.02, 0.92) | 0.23 |  |
| Marital status | Married | 0.97 | (0.86, 1.09) | 0.06 | < .001 |
|  | Separated | 2.44 | (1.28, 3.60) | 0.59 |  |
|  | Divorced | 1.74 | (1.50, 1.99) | 0.13 |  |
|  | Widowed | 1.43 | (1.11, 1.74) | 0.16 |  |
|  | Never | 1.29 | (0.92, 1.66) | 0.19 |  |
|  | Domestic Partner | 2.45 | (1.69, 3.20) | 0.38 |  |
| Employment | Employed for an employer | 1.14 | (0.96, 1.33) | 0.09 | < .001 |
|  | Self-employed | 1.63 | (1.13, 2.13) | 0.26 |  |
|  | Retired | 1.09 | (0.98, 1.20) | 0.06 |  |
|  | Student | 0.30 | (0.08, 0.51) | 0.11 |  |
|  | Homemaker | 1.57 | (1.00, 2.13) | 0.29 |  |
|  | Unemployed and looking for a job | 1.53 | (0.87, 2.18) | 0.34 |  |
|  | None of these/Other | 3.01 | (1.92, 4.11) | 0.56 |  |
| Religious service attendance | More than 1/week | 0.39 | (0.25, 0.53) | 0.07 | < .001 |
|  | 1/week | 0.49 | (0.37, 0.61) | 0.06 |  |
|  | 1-3/month | 0.92 | (0.61, 1.23) | 0.16 |  |
|  | A few times a year | 1.38 | (1.14, 1.62) | 0.12 |  |
|  | Never | 1.62 | (1.40, 1.84) | 0.11 |  |
| Education | Up to 8 years | 3.84 | (0.00, 9.88) | 3.06 | < .001 |
|  | 9-15 years | 1.68 | (1.50, 1.86) | 0.09 |  |
|  | 16+ years | 0.35 | (0.32, 0.39) | 0.02 |  |
| Immigration status | Born in this country | 1.34 | (1.20, 1.47) | 0.07 | < .001 |
|  | Born in another country | 0.26 | (0.17, 0.36) | 0.05 |  |
| Religious affiliation | Christianity | 1.16 | (1.02, 1.31) | 0.07 | < .001 |
|  | Islam | 1.42 | (0.00, 3.61) | 1.11 |  |
|  | Hinduism | 0.10 | (0.00, 0.23) | 0.06 |  |
|  | Buddhism | 0.83 | (0.24, 1.41) | 0.30 |  |
|  | Judaism | 0.55 | (0.32, 0.78) | 0.12 |  |
|  | Sikhism | 0.00 | * | * |  |
|  | Baha'i | 1.51 | (0.00, 9.77) | 1.12 |  |
|  | Jainism | 0.00 | * | * |  |
|  | Shinto | 0.00 | * | * |  |
|  | Taoism | 4.69 | (0.22, 9.16) | 2.24 |  |
|  | Confucianism | 0.00 | * | * |  |
|  | Primal, Animist, or Folk religion | 1.59 | (0.38, 2.80) | 0.62 |  |
|  | Some other religion | 2.60 | (0.54, 4.65) | 1.01 |  |
|  | No religion/Atheist/Agnostic | 1.31 | (1.09, 1.53) | 0.11 |  |
| Race/Ethnicity | Asian | 0.32 | (0.07, 0.57) | 0.13 | < .001 |
|  | Black | 1.17 | (0.84, 1.50) | 0.17 |  |
|  | White | 1.39 | (1.28, 1.51) | 0.06 |  |
|  | Other | 1.94 | (1.10, 2.78) | 0.43 |  |
|  | Hispanic | 1.05 | (0.55, 1.54) | 0.25 |  |

# **Table S23. Population weighted meta-analysis of daily cigarette consumption per capita (mean) by demographic category**

| Variable | Category | Est | 95% CI | SE |
| --- | --- | --- | --- | --- |
| Age group |  |  |  |  |
|  | 18-24 | 1.10 | (0.98,1.23) | 0.064 |
|  | 25-34 | 1.74 | (1.61,1.86) | 0.064 |
|  | 35-44 | 1.95 | (1.86,2.05) | 0.048 |
|  | 45-54 | 2.16 | (2.01,2.31) | 0.075 |
|  | 55-64 | 2.14 | (1.99,2.29) | 0.077 |
|  | 65-74 | 1.51 | (1.32,1.69) | 0.095 |
|  | 75-84 | 0.93 | (0.54,1.33) | 0.202 |
|  | 85 or older | 0.67 | (-1.63,2.97) | 1.173 |
| Gender |  |  |  |  |
|  | Male | 2.70 | (2.61,2.80) | 0.049 |
|  | Female | 0.87 | (0.82,0.92) | 0.024 |
|  | Other | 1.18 | (0.64,1.72) | 0.273 |
| Marital status |  |  |  |  |
|  | Married | 1.64 | (1.58,1.70) | 0.030 |
|  | Separated | 2.35 | (1.78,2.93) | 0.295 |
|  | Divorced | 2.10 | (1.88,2.32) | 0.112 |
|  | Widowed | 1.46 | (1.16,1.76) | 0.153 |
|  | Domestic partner | 1.59 | (1.32,1.86) | 0.137 |
|  | Single, never married | 1.88 | (1.70,2.06) | 0.093 |
| Employment status |  |  |  |  |
|  | Employed for an employer | 2.07 | (1.97,2.16) | 0.049 |
|  | Self-employed | 2.48 | (2.34,2.62) | 0.071 |
|  | Retired | 1.84 | (1.52,2.15) | 0.160 |
|  | Student | 0.76 | (0.58,0.93) | 0.090 |
|  | Homemaker | 1.07 | (0.96,1.18) | 0.058 |
|  | Unemployed and looking for a job | 2.39 | (2.08,2.70) | 0.159 |
|  | None of these/other | 2.54 | (2.25,2.83) | 0.148 |
| Education |  |  |  |  |
|  | Up to 8 years | 2.32 | (1.67,2.96) | 0.331 |
|  | 9-15 years | 1.81 | (1.72,1.91) | 0.048 |
|  | 16+ years | 1.10 | (1.01,1.19) | 0.046 |
| Religious service attendance |  |  |  |  |
|  | >1/week | 1.50 | (1.38,1.62) | 0.062 |
|  | 1/week | 1.65 | (1.53,1.77) | 0.062 |
|  | 1-3/month | 1.65 | (1.53,1.77) | 0.059 |
|  | A few times a year | 1.92 | (1.80,2.05) | 0.064 |
|  | Never | 2.00 | (1.83,2.18) | 0.091 |
| Immigration status |  |  |  |  |
|  | Born in this country | 1.79 | (1.74,1.85) | 0.028 |
|  | Born in another country | 2.17 | (1.40,2.94) | 0.393 |

# Table S24a. Descriptive statistics for Argentina conditional on smokers

| **Characteristic** | **N = 2,293**^1^ |
| --- | --- |
| **Age group** |  |
| 18-24 | 343 (15%) |
| 25-34 | 603 (26%) |
| 35-44 | 521 (23%) |
| 45-54 | 356 (16%) |
| 55-64 | 267 (12%) |
| 65-74 | 172 (7.5%) |
| 75-84 | 28 (1.2%) |
| 85 or older | 3 (0.1%) |
| (Missing) | 0 (0%) |
| **Gender** |  |
| Male | 1,142 (50%) |
| Female | 1,134 (49%) |
| Other | 5 (0.2%) |
| (Missing) | 13 (0.6%) |
| **Marital status** |  |
| Married | 392 (17%) |
| Separated | 203 (8.9%) |
| Divorced | 121 (5.3%) |
| Widowed | 110 (4.8%) |
| Single, never married | 869 (38%) |
| Domestic Partner | 560 (24%) |
| (Missing) | 38 (1.7%) |
| **Employment** |  |
| Employed for an employer | 920 (40%) |
| Self-employed | 567 (25%) |
| Retired | 189 (8.2%) |
| Student | 89 (3.9%) |
| Homemaker | 235 (10%) |
| Unemployed and looking for a job | 226 (9.8%) |
| None of these/Other | 64 (2.8%) |
| (Missing) | 4 (0.2%) |
| **Religious service attendance** |  |
| More than 1/week | 107 (4.7%) |
| 1/week | 220 (9.6%) |
| 1-3/month | 179 (7.8%) |
| A few times a year | 635 (28%) |
| Never | 1,140 (50%) |
| (Missing) | 13 (0.6%) |
| **Education** |  |
| Up to 8 years | 869 (38%) |
| 9-15 years | 1,281 (56%) |
| 16+ years | 143 (6.2%) |
| (Missing) | 1 (<0.1%) |
| **Immigration** |  |
| Born in this country | 2,205 (96%) |
| Born in another country | 86 (3.7%) |
| (Missing) | 3 (0.1%) |
| **Religious affiliation** |  |
| Christianity | 1,622 (71%) |
| Islam | 1 (<0.1%) |
| Hinduism | 6 (0.3%) |
| Buddhism | 18 (0.8%) |
| Judaism | 15 (0.7%) |
| Sikhism | 0 (0%) |
| Baha'i | 0 (0%) |
| Jainism | 0 (0%) |
| Shinto | 0 (0%) |
| Taoism | 1 (<0.1%) |
| Confucianism | 0 (0%) |
| Primal, Animist, or Folk religion | 6 (0.3%) |
| Spiritism | 0 (0%) |
| Umbanda, Candomble, and other African-derived religions | 0 (0%) |
| Chinese folk/traditional religion | 0 (0%) |
| Some other religion | 65 (2.8%) |
| No religion/Atheist/Agnostic | 525 (23%) |
| (Missing) | 34 (1.5%) |
| **Race/Ethnicity** |  |
| Asian | 17 (0.7%) |
| Black | 40 (1.7%) |
| Indigenous | 58 (2.5%) |
| Mestizo(a) | 610 (27%) |
| Mullato(a) | 18 (0.8%) |
| Other | 42 (1.8%) |
| White | 1,088 (47%) |
| (Missing) | 420 (18%) |
| ^1^n (%) | |

# Table S24b. Intensity by demographic category for Argentina conditional on smokers

| Variable | Category | Intensity | 95% CI | SE | Global p-value |
| --- | --- | --- | --- | --- | --- |
| Age group | 18-24 | 9.05 | (7.46, 10.64) | 0.81 | < .001 |
|  | 25-34 | 10.93 | (9.63, 12.23) | 0.66 |  |
|  | 35-44 | 12.53 | (11.09, 13.96) | 0.73 |  |
|  | 45-54 | 12.63 | (11.35, 13.91) | 0.65 |  |
|  | 55-64 | 13.70 | (11.98, 15.43) | 0.87 |  |
|  | 65-74 | 13.24 | (11.21, 15.27) | 1.03 |  |
|  | 75-84 | 10.44 | (5.73, 15.15) | 2.18 |  |
|  | 85 or older | 9.31 | * | * |  |
| Gender | Male | 12.73 | (11.77, 13.70) | 0.49 | 0.007 |
|  | Female | 10.77 | (9.99, 11.56) | 0.40 |  |
|  | Other | 10.79 | * | * |  |
| Marital status | Married | 11.97 | (10.64, 13.29) | 0.68 | 0.263 |
|  | Separated | 12.44 | (9.94, 14.95) | 1.27 |  |
|  | Divorced | 14.18 | (11.60, 16.77) | 1.30 |  |
|  | Widowed | 12.28 | (9.83, 14.73) | 1.23 |  |
|  | Never | 10.99 | (9.97, 12.00) | 0.52 |  |
|  | Domestic Partner | 11.99 | (10.73, 13.25) | 0.64 |  |
| Employment | Employed for an employer | 11.48 | (10.52, 12.44) | 0.49 | < .001 |
|  | Self-employed | 12.86 | (11.49, 14.24) | 0.70 |  |
|  | Retired | 12.57 | (10.81, 14.33) | 0.89 |  |
|  | Student | 6.70 | (4.98, 8.42) | 0.86 |  |
|  | Homemaker | 10.30 | (8.70, 11.90) | 0.81 |  |
|  | Unemployed and looking for a job | 12.09 | (9.64, 14.54) | 1.24 |  |
|  | None of these/Other | 15.24 | (11.25, 19.22) | 1.98 |  |
| Religious service attendance | More than 1/week | 11.46 | (8.25, 14.66) | 1.61 | 0.003 |
|  | 1/week | 9.21 | (7.73, 10.68) | 0.75 |  |
|  | 1-3/month | 10.33 | (8.20, 12.46) | 1.08 |  |
|  | A few times a year | 11.81 | (10.52, 13.11) | 0.66 |  |
|  | Never | 12.46 | (11.60, 13.32) | 0.44 |  |
| Education | Up to 8 years | 12.70 | (11.40, 14.00) | 0.66 | 0.004 |
|  | 9-15 years | 11.44 | (10.80, 12.08) | 0.33 |  |
|  | 16+ years | 8.89 | (7.02, 10.76) | 0.94 |  |
| Immigration status | Born in this country | 11.82 | (11.18, 12.47) | 0.33 | 0.205 |
|  | Born in another country | 10.27 | (7.89, 12.66) | 1.20 |  |
| Religious affiliation | Christianity | 11.93 | (11.16, 12.70) | 0.39 | < .001 |
|  | Islam | 5.29 | * | * |  |
|  | Hinduism | 3.62 | * | * |  |
|  | Buddhism | 6.94 | (3.95, 9.92) | 1.26 |  |
|  | Judaism | 6.07 | (0.37, 11.77) | 2.13 |  |
|  | Taoism | 17.55 | * | * |  |
|  | Primal, Animist, or Folk religion | 18.89 | (10.49, 27.28) | 4.26 |  |
|  | Some other religion | 13.32 | (9.31, 17.34) | 2.00 |  |
|  | No religion/Atheist/Agnostic | 11.42 | (10.24, 12.59) | 0.60 |  |
| Race/Ethnicity | Asian | 10.98 | (3.80, 18.16) | 2.96 | 0.062 |
|  | Black | 12.98 | (7.96, 17.99) | 2.42 |  |
|  | Indigenous | 9.63 | (5.56, 13.70) | 2.02 |  |
|  | Mestizo(a) | 10.65 | (9.68, 11.61) | 0.49 |  |
|  | Mullato(a) | 15.51 | (6.13, 24.89) | 4.22 |  |
|  | White | 12.36 | (11.48, 13.24) | 0.45 |  |
|  | Other | 13.81 | (8.49, 19.14) | 2.63 |  |

# Table S25a. Descriptive statistics for Australia conditional on smokers

| **Characteristic** | **N = 483**^1^ |
| --- | --- |
| **Age group** |  |
| 18-24 | 20 (4.2%) |
| 25-34 | 77 (16%) |
| 35-44 | 106 (22%) |
| 45-54 | 108 (22%) |
| 55-64 | 103 (21%) |
| 65-74 | 39 (8.1%) |
| 75-84 | 23 (4.8%) |
| 85 or older | 7 (1.4%) |
| (Missing) | 0 (0%) |
| **Gender** |  |
| Male | 266 (55%) |
| Female | 209 (43%) |
| Other | 6 (1.2%) |
| (Missing) | 2 (0.4%) |
| **Marital status** |  |
| Married | 144 (30%) |
| Separated | 39 (8.0%) |
| Divorced | 58 (12%) |
| Widowed | 25 (5.1%) |
| Single, never married | 128 (27%) |
| Domestic Partner | 82 (17%) |
| (Missing) | 7 (1.6%) |
| **Employment** |  |
| Employed for an employer | 235 (49%) |
| Self-employed | 43 (8.9%) |
| Retired | 84 (17%) |
| Student | 11 (2.3%) |
| Homemaker | 28 (5.9%) |
| Unemployed and looking for a job | 37 (7.7%) |
| None of these/Other | 41 (8.5%) |
| (Missing) | 3 (0.5%) |
| **Religious service attendance** |  |
| More than 1/week | 7 (1.4%) |
| 1/week | 19 (4.0%) |
| 1-3/month | 14 (3.0%) |
| A few times a year | 70 (15%) |
| Never | 371 (77%) |
| (Missing) | 1 (0.3%) |
| **Education** |  |
| Up to 8 years | 22 (4.5%) |
| 9-15 years | 370 (77%) |
| 16+ years | 90 (19%) |
| (Missing) | 0 (0%) |
| **Immigration** |  |
| Born in this country | 395 (82%) |
| Born in another country | 88 (18%) |
| (Missing) | 0 (0%) |
| **Religious affiliation** |  |
| Christianity | 163 (34%) |
| Islam | 5 (1.1%) |
| Hinduism | 0 (<0.1%) |
| Buddhism | 6 (1.2%) |
| Judaism | 1 (0.2%) |
| Sikhism | 0 (0%) |
| Baha'i | 0 (0%) |
| Jainism | 0 (0%) |
| Shinto | 0 (0%) |
| Taoism | 3 (0.6%) |
| Confucianism | 0 (0%) |
| Primal, Animist, or Folk religion | 2 (0.5%) |
| Spiritism | 0 (0%) |
| Umbanda, Candomble, and other African-derived religions | 0 (0%) |
| Chinese folk/traditional religion | 0 (0%) |
| Some other religion | 6 (1.2%) |
| No religion/Atheist/Agnostic | 297 (61%) |
| (Missing) | 0 (<0.1%) |
| **Race/Ethnicity** |  |
| Aboriginal | 11 (2.3%) |
| Australian | 248 (51%) |
| Australian British/European | 132 (27%) |
| Chinese | 4 (0.9%) |
| Indian | 0 (<0.1%) |
| Japanese | 0 (0%) |
| Malay | 0 (0%) |
| New Zealander | 18 (3.7%) |
| Other | 25 (5.3%) |
| Other European | 32 (6.6%) |
| Russian | 3 (0.6%) |
| Samoan | 4 (0.9%) |
| Sinhalese | 0 (0%) |
| Spanish | 0 (<0.1%) |
| Sri Lankan Moor | 0 (0%) |
| Sri Lankan Tamil | 0 (0%) |
| Vietnamese | 1 (0.2%) |
| (Missing) | 3 (0.7%) |
| ^1^n (%) | |

# Table S25b. Intensity by demographic category for Australia conditional on smokers

| Variable | Category | Intensity | 95% CI | SE | Global p-value |
| --- | --- | --- | --- | --- | --- |
| Age group | 18-24 | 3.53 | (1.73, 5.33) | 0.81 | < .001 |
|  | 25-34 | 8.15 | (5.92, 10.38) | 1.11 |  |
|  | 35-44 | 13.16 | (10.31, 16.02) | 1.44 |  |
|  | 45-54 | 12.70 | (10.61, 14.80) | 1.05 |  |
|  | 55-64 | 11.38 | (9.73, 13.04) | 0.83 |  |
|  | 65-74 | 12.51 | (9.36, 15.67) | 1.53 |  |
|  | 75-84 | 13.92 | (1.92, 25.93) | 3.93 |  |
|  | 85 or older | 11.80 | * | * |  |
| Gender | Male | 11.81 | (10.23, 13.39) | 0.80 | < .001 |
|  | Female | 10.98 | (9.58, 12.38) | 0.71 |  |
|  | Other | 4.78 | (0.00, 15.49) | 1.56 |  |
| Marital status | Married | 10.97 | (9.30, 12.64) | 0.84 | 0.334 |
|  | Separated | 14.16 | (10.93, 17.39) | 1.58 |  |
|  | Divorced | 12.52 | (9.28, 15.76) | 1.61 |  |
|  | Widowed | 13.71 | (8.44, 18.97) | 2.39 |  |
|  | Never | 10.53 | (8.20, 12.86) | 1.18 |  |
|  | Domestic Partner | 10.72 | (7.99, 13.44) | 1.36 |  |
| Employment | Employed for an employer | 10.66 | (9.23, 12.09) | 0.72 | < .001 |
|  | Self-employed | 7.20 | (4.17, 10.23) | 1.47 |  |
|  | Retired | 14.30 | (11.83, 16.77) | 1.23 |  |
|  | Student | 5.21 | (0.00, 11.08) | 1.58 |  |
|  | Homemaker | 13.79 | (8.18, 19.40) | 2.62 |  |
|  | Unemployed and looking for a job | 13.35 | (9.24, 17.45) | 2.00 |  |
|  | None of these/Other | 12.71 | (8.23, 17.18) | 2.19 |  |
| Religious service attendance | More than 1/week | 5.98 | * | * | 0.233 |
|  | 1/week | 12.24 | (6.92, 17.55) | 2.20 |  |
|  | 1-3/month | 9.13 | (0.00, 41.25) | 4.05 |  |
|  | A few times a year | 11.11 | (8.26, 13.96) | 1.42 |  |
|  | Never | 11.49 | (10.27, 12.71) | 0.62 |  |
| Education | Up to 8 years | 11.99 | (5.58, 18.39) | 3.03 | 0.009 |
|  | 9-15 years | 11.99 | (10.72, 13.26) | 0.64 |  |
|  | 16+ years | 8.64 | (6.88, 10.40) | 0.88 |  |
| Immigration status | Born in this country | 11.30 | (10.17, 12.43) | 0.57 | 0.754 |
|  | Born in another country | 11.70 | (8.57, 14.83) | 1.56 |  |
| Religious affiliation | Christianity | 12.34 | (10.58, 14.11) | 0.89 | < .001 |
|  | Islam | 11.08 | (0.00, 23.20) | 6.00 |  |
|  | Buddhism | 17.71 | (12.98, 22.44) | 2.26 |  |
|  | Judaism | 14.15 | * | * |  |
|  | Taoism | 2.01 | * | * |  |
|  | Primal, Animist, or Folk religion | 3.00 | * | * |  |
|  | Some other religion | 15.56 | (8.32, 22.79) | 3.18 |  |
|  | No religion/Atheist/Agnostic | 10.87 | (9.48, 12.25) | 0.70 |  |
| Race/Ethnicity | Aboriginal | 16.55 | * | * | < .001 |
|  | Australian | 11.60 | (10.22, 12.98) | 0.70 |  |
|  | Australian British/European | 9.66 | (7.86, 11.46) | 0.91 |  |
|  | Chinese | 3.45 | * | * |  |
|  | Indian | 6.00 | * | * |  |
|  | Spanish | 5.00 | * | * |  |
|  | Vietnamese | 10.00 | * | * |  |
|  | Russian | 10.36 | * | * |  |
|  | Samoan | 25.88 | * | * |  |
|  | New Zealander | 12.87 | (0.00, 27.44) | 3.43 |  |
|  | Other European | 13.63 | (7.57, 19.69) | 2.81 |  |
|  | Other | 10.90 | (4.93, 16.86) | 2.71 |  |

# Table S26a. Descriptive statistics for Brazil conditional on smokers

| **Characteristic** | **N = 2,785**^1^ |
| --- | --- |
| **Age group** |  |
| 18-24 | 258 (9.3%) |
| 25-34 | 619 (22%) |
| 35-44 | 684 (25%) |
| 45-54 | 525 (19%) |
| 55-64 | 462 (17%) |
| 65-74 | 218 (7.8%) |
| 75-84 | 12 (0.4%) |
| 85 or older | 6 (0.2%) |
| (Missing) | 0 (0%) |
| **Gender** |  |
| Male | 1,549 (56%) |
| Female | 1,216 (44%) |
| Other | 10 (0.4%) |
| (Missing) | 10 (0.4%) |
| **Marital status** |  |
| Married | 697 (25%) |
| Separated | 186 (6.7%) |
| Divorced | 256 (9.2%) |
| Widowed | 83 (3.0%) |
| Single, never married | 960 (34%) |
| Domestic Partner | 533 (19%) |
| (Missing) | 71 (2.5%) |
| **Employment** |  |
| Employed for an employer | 727 (26%) |
| Self-employed | 710 (25%) |
| Retired | 276 (9.9%) |
| Student | 60 (2.2%) |
| Homemaker | 259 (9.3%) |
| Unemployed and looking for a job | 607 (22%) |
| None of these/Other | 95 (3.4%) |
| (Missing) | 52 (1.9%) |
| **Religious service attendance** |  |
| More than 1/week | 314 (11%) |
| 1/week | 444 (16%) |
| 1-3/month | 335 (12%) |
| A few times a year | 934 (34%) |
| Never | 748 (27%) |
| (Missing) | 10 (0.4%) |
| **Education** |  |
| Up to 8 years | 846 (30%) |
| 9-15 years | 1,618 (58%) |
| 16+ years | 319 (11%) |
| (Missing) | 2 (<0.1%) |
| **Immigration** |  |
| Born in this country | 2,669 (96%) |
| Born in another country | 35 (1.3%) |
| (Missing) | 82 (2.9%) |
| **Religious affiliation** |  |
| Christianity | 1,754 (63%) |
| Islam | 2 (<0.1%) |
| Hinduism | 1 (<0.1%) |
| Buddhism | 11 (0.4%) |
| Judaism | 11 (0.4%) |
| Sikhism | 0 (0%) |
| Baha'i | 1 (<0.1%) |
| Jainism | 2 (<0.1%) |
| Shinto | 0 (<0.1%) |
| Taoism | 0 (<0.1%) |
| Confucianism | 0 (0%) |
| Primal, Animist, or Folk religion | 1 (<0.1%) |
| Spiritism | 222 (8.0%) |
| Umbanda, Candomble, and other African-derived religions | 242 (8.7%) |
| Chinese folk/traditional religion | 0 (0%) |
| Some other religion | 49 (1.7%) |
| No religion/Atheist/Agnostic | 468 (17%) |
| (Missing) | 22 (0.8%) |
| **Race/Ethnicity** |  |
| Amarela | 58 (2.1%) |
| Branca | 1,105 (40%) |
| Indígena | 36 (1.3%) |
| Other | 17 (0.6%) |
| Parda | 1,018 (37%) |
| Preta | 339 (12%) |
| (Missing) | 213 (7.6%) |
| ^1^n (%) | |

# Table S26b. Intensity by demographic category for Brazil conditional on smokers

| Variable | Category | Intensity | 95% CI | SE | Global p-value |
| --- | --- | --- | --- | --- | --- |
| Age group | 18-24 | 9.70 | (8.19, 11.22) | 0.77 | < .001 |
|  | 25-34 | 11.77 | (10.84, 12.71) | 0.48 |  |
|  | 35-44 | 11.79 | (11.09, 12.49) | 0.36 |  |
|  | 45-54 | 13.06 | (11.93, 14.19) | 0.57 |  |
|  | 55-64 | 13.01 | (11.69, 14.33) | 0.67 |  |
|  | 65-74 | 13.97 | (11.39, 16.56) | 1.31 |  |
|  | 75-84 | 6.08 | (0.00, 18.11) | 1.59 |  |
|  | 85 or older | 9.43 | (4.13, 14.73) | 1.72 |  |
| Gender | Male | 12.77 | (12.14, 13.39) | 0.32 | 0.002 |
|  | Female | 11.51 | (10.76, 12.25) | 0.38 |  |
|  | Other | 7.44 | (1.43, 13.45) | 1.98 |  |
| Marital status | Married | 12.74 | (11.74, 13.73) | 0.51 | 0.189 |
|  | Separated | 13.93 | (11.79, 16.07) | 1.08 |  |
|  | Divorced | 12.74 | (11.03, 14.44) | 0.86 |  |
|  | Widowed | 10.84 | (7.35, 14.33) | 1.75 |  |
|  | Never | 11.76 | (10.96, 12.56) | 0.41 |  |
|  | Domestic Partner | 11.69 | (10.82, 12.55) | 0.44 |  |
| Employment | Employed for an employer | 11.01 | (10.29, 11.73) | 0.36 | 0.001 |
|  | Self-employed | 12.64 | (11.72, 13.56) | 0.47 |  |
|  | Retired | 13.31 | (11.25, 15.37) | 1.05 |  |
|  | Student | 8.27 | (5.20, 11.35) | 1.51 |  |
|  | Homemaker | 13.66 | (11.66, 15.66) | 1.01 |  |
|  | Unemployed and looking for a job | 12.46 | (11.48, 13.45) | 0.50 |  |
|  | None of these/Other | 11.11 | (9.01, 13.20) | 1.05 |  |
| Religious service attendance | More than 1/week | 11.21 | (9.35, 13.07) | 0.94 | 0.012 |
|  | 1/week | 11.70 | (10.39, 13.02) | 0.67 |  |
|  | 1-3/month | 10.98 | (9.64, 12.33) | 0.68 |  |
|  | A few times a year | 12.28 | (11.55, 13.00) | 0.37 |  |
|  | Never | 13.33 | (12.43, 14.22) | 0.46 |  |
| Education | Up to 8 years | 12.81 | (11.82, 13.80) | 0.51 | 0.249 |
|  | 9-15 years | 11.87 | (11.27, 12.47) | 0.30 |  |
|  | 16+ years | 12.24 | (10.83, 13.66) | 0.72 |  |
| Immigration status | Born in this country | 12.27 | (11.79, 12.74) | 0.24 | < .001 |
|  | Born in another country | 7.82 | (5.25, 10.39) | 1.26 |  |
| Religious affiliation | Christianity | 12.09 | (11.50, 12.67) | 0.30 | < .001 |
|  | Islam | 29.58 | * | * |  |
|  | Hinduism | 20.00 | * | * |  |
|  | Buddhism | 11.43 | (8.12, 14.73) | 1.53 |  |
|  | Judaism | 9.91 | * | * |  |
|  | Baha'i | 1.36 | * | * |  |
|  | Jainism | 6.51 | * | * |  |
|  | Shinto | 33.00 | * | * |  |
|  | Taoism | 2.00 | * | * |  |
|  | Primal, Animist, or Folk religion | 5.74 | * | * |  |
|  | Spiritism | 13.89 | (11.83, 15.95) | 1.05 |  |
|  | Umbanda, Candomble, and other African-derived religions | 12.00 | (10.04, 13.96) | 0.98 |  |
|  | Some other religion | 15.02 | (11.12, 18.91) | 1.90 |  |
|  | No religion/Atheist/Agnostic | 11.68 | (10.61, 12.75) | 0.54 |  |
| Race/Ethnicity | Branca | 13.17 | (12.46, 13.88) | 0.36 | < .001 |
|  | Preta | 10.15 | (9.14, 11.15) | 0.51 |  |
|  | Parda | 11.80 | (11.00, 12.60) | 0.41 |  |
|  | Amarela | 11.99 | (8.80, 15.18) | 1.58 |  |
|  | Indígena | 13.94 | (7.23, 20.65) | 3.28 |  |
|  | Other | 13.45 | (9.58, 17.33) | 1.65 |  |

# Table S27a. Descriptive statistics for Egypt conditional on smokers

| **Characteristic** | **N = 1,085**^1^ |
| --- | --- |
| **Age group** |  |
| 18-24 | 193 (18%) |
| 25-34 | 329 (30%) |
| 35-44 | 226 (21%) |
| 45-54 | 155 (14%) |
| 55-64 | 152 (14%) |
| 65-74 | 30 (2.7%) |
| 75-84 | 1 (<0.1%) |
| 85 or older | 0 (0%) |
| (Missing) | 0 (0%) |
| **Gender** |  |
| Male | 1,055 (97%) |
| Female | 30 (2.8%) |
| Other | 0 (0%) |
| (Missing) | 0 (0%) |
| **Marital status** |  |
| Married | 761 (70%) |
| Separated | 5 (0.5%) |
| Divorced | 13 (1.2%) |
| Widowed | 22 (2.0%) |
| Single, never married | 275 (25%) |
| Domestic Partner | 0 (0%) |
| (Missing) | 8 (0.8%) |
| **Employment** |  |
| Employed for an employer | 485 (45%) |
| Self-employed | 369 (34%) |
| Retired | 68 (6.3%) |
| Student | 46 (4.3%) |
| Homemaker | 28 (2.6%) |
| Unemployed and looking for a job | 82 (7.6%) |
| None of these/Other | 6 (0.6%) |
| (Missing) | 0 (0%) |
| **Religious service attendance** |  |
| More than 1/week | 232 (21%) |
| 1/week | 273 (25%) |
| 1-3/month | 101 (9.3%) |
| A few times a year | 114 (11%) |
| Never | 363 (33%) |
| (Missing) | 1 (<0.1%) |
| **Education** |  |
| Up to 8 years | 613 (57%) |
| 9-15 years | 362 (33%) |
| 16+ years | 110 (10%) |
| (Missing) | 0 (0%) |
| **Immigration** |  |
| Born in this country | 1,081 (100%) |
| Born in another country | 4 (0.3%) |
| (Missing) | 0 (0%) |
| **Religious affiliation** |  |
| Christianity | 17 (1.6%) |
| Islam | 1,067 (98%) |
| Hinduism | 0 (0%) |
| Buddhism | 0 (0%) |
| Judaism | 0 (0%) |
| Sikhism | 0 (0%) |
| Baha'i | 0 (0%) |
| Jainism | 0 (0%) |
| Shinto | 0 (0%) |
| Taoism | 0 (0%) |
| Confucianism | 0 (0%) |
| Primal, Animist, or Folk religion | 0 (0%) |
| Spiritism | 0 (0%) |
| Umbanda, Candomble, and other African-derived religions | 0 (0%) |
| Chinese folk/traditional religion | 0 (0%) |
| Some other religion | 0 (0%) |
| No religion/Atheist/Agnostic | 0 (0%) |
| (Missing) | 1 (<0.1%) |
| **Race/Ethnicity** |  |
| Arab | 1,063 (98%) |
| Bedouin Arab | 1 (<0.1%) |
| Greek | 0 (0%) |
| Nubian | 9 (0.8%) |
| Turkish | 2 (0.2%) |
| (Missing) | 10 (0.9%) |
| ^1^n (%) | |

# Table S27b. Intensity by demographic category for Egypt conditional on smokers

| Variable | Category | Intensity | 95% CI | SE | Global p-value |
| --- | --- | --- | --- | --- | --- |
| Age group | 18-24 | 14.30 | (12.14, 16.45) | 1.09 | < .001 |
|  | 25-34 | 15.11 | (13.62, 16.60) | 0.76 |  |
|  | 35-44 | 14.09 | (12.99, 15.19) | 0.56 |  |
|  | 45-54 | 14.25 | (12.67, 15.82) | 0.79 |  |
|  | 55-64 | 16.99 | (14.79, 19.20) | 1.11 |  |
|  | 65-74 | 14.50 | (10.20, 18.79) | 2.05 |  |
|  | 75-84 | 10.00 | * | * |  |
| Gender | Male | 14.95 | (14.19, 15.71) | 0.39 | 0.012 |
|  | Female | 9.00 | (3.74, 14.26) | 2.34 |  |
| Marital status | Married | 14.37 | (13.48, 15.26) | 0.46 | 0.123 |
|  | Separated | 18.60 | * | * |  |
|  | Divorced | 27.81 | (11.70, 43.93) | 6.61 |  |
|  | Widowed | 13.96 | (10.87, 17.04) | 1.42 |  |
|  | Never | 15.63 | (14.21, 17.06) | 0.72 |  |
| Employment | Employed for an employer | 15.65 | (14.47, 16.83) | 0.60 | 0.033 |
|  | Self-employed | 14.23 | (12.88, 15.58) | 0.68 |  |
|  | Retired | 14.78 | (12.02, 17.55) | 1.38 |  |
|  | Student | 12.50 | (10.34, 14.67) | 1.07 |  |
|  | Homemaker | 9.55 | (3.71, 15.39) | 2.49 |  |
|  | Unemployed and looking for a job | 15.94 | (12.85, 19.02) | 1.55 |  |
|  | None of these/Other | 12.07 | * | * |  |
| Religious service attendance | More than 1/week | 13.95 | (12.32, 15.59) | 0.83 | 0.115 |
|  | 1/week | 14.81 | (13.66, 15.96) | 0.58 |  |
|  | 1-3/month | 13.36 | (10.94, 15.78) | 1.22 |  |
|  | A few times a year | 17.55 | (15.21, 19.89) | 1.18 |  |
|  | Never | 15.10 | (13.70, 16.50) | 0.71 |  |
| Education | Up to 8 years | 14.91 | (13.89, 15.92) | 0.52 | 0.417 |
|  | 9-15 years | 15.17 | (14.11, 16.23) | 0.54 |  |
|  | 16+ years | 13.68 | (11.49, 15.86) | 1.10 |  |
| Immigration status | Born in this country | 14.87 | (14.12, 15.63) | 0.39 | 0.131 |
|  | Born in another country | 16.21 | * | * |  |
| Religious affiliation | Christianity | 18.76 | (15.04, 22.49) | 1.72 | 0.030 |
|  | Islam | 14.81 | (14.05, 15.58) | 0.39 |  |
| Race/Ethnicity | Arab | 14.91 | (14.15, 15.67) | 0.39 | < .001 |
|  | Turkish | 2.00 | * | * |  |
|  | Bedouin Arab | 10.00 | * | * |  |
|  | Nubian | 13.98 | (9.90, 18.07) | 1.26 |  |

# Table S28a. Descriptive statistics for Germany conditional on smokers

| **Characteristic** | **N = 2,590**^1^ |
| --- | --- |
| **Age group** |  |
| 18-24 | 171 (6.6%) |
| 25-34 | 391 (15%) |
| 35-44 | 390 (15%) |
| 45-54 | 532 (21%) |
| 55-64 | 535 (21%) |
| 65-74 | 480 (19%) |
| 75-84 | 88 (3.4%) |
| 85 or older | 4 (0.2%) |
| (Missing) | 0 (0%) |
| **Gender** |  |
| Male | 1,299 (50%) |
| Female | 1,287 (50%) |
| Other | 3 (0.1%) |
| (Missing) | 1 (<0.1%) |
| **Marital status** |  |
| Married | 1,289 (50%) |
| Separated | 71 (2.7%) |
| Divorced | 248 (9.6%) |
| Widowed | 98 (3.8%) |
| Single, never married | 686 (26%) |
| Domestic Partner | 176 (6.8%) |
| (Missing) | 22 (0.9%) |
| **Employment** |  |
| Employed for an employer | 1,424 (55%) |
| Self-employed | 208 (8.0%) |
| Retired | 595 (23%) |
| Student | 92 (3.5%) |
| Homemaker | 87 (3.3%) |
| Unemployed and looking for a job | 121 (4.7%) |
| None of these/Other | 57 (2.2%) |
| (Missing) | 7 (0.3%) |
| **Religious service attendance** |  |
| More than 1/week | 70 (2.7%) |
| 1/week | 130 (5.0%) |
| 1-3/month | 181 (7.0%) |
| A few times a year | 569 (22%) |
| Never | 1,637 (63%) |
| (Missing) | 4 (0.2%) |
| **Education** |  |
| Up to 8 years | 78 (3.0%) |
| 9-15 years | 1,874 (72%) |
| 16+ years | 631 (24%) |
| (Missing) | 7 (0.3%) |
| **Immigration** |  |
| Born in this country | 2,395 (92%) |
| Born in another country | 183 (7.1%) |
| (Missing) | 13 (0.5%) |
| **Religious affiliation** |  |
| Christianity | 1,371 (53%) |
| Islam | 114 (4.4%) |
| Hinduism | 6 (0.2%) |
| Buddhism | 17 (0.6%) |
| Judaism | 4 (0.1%) |
| Sikhism | 2 (<0.1%) |
| Baha'i | 3 (0.1%) |
| Jainism | 0 (0%) |
| Shinto | 0 (0%) |
| Taoism | 0 (<0.1%) |
| Confucianism | 0 (0%) |
| Primal, Animist, or Folk religion | 8 (0.3%) |
| Spiritism | 0 (0%) |
| Umbanda, Candomble, and other African-derived religions | 0 (0%) |
| Chinese folk/traditional religion | 0 (0%) |
| Some other religion | 21 (0.8%) |
| No religion/Atheist/Agnostic | 1,018 (39%) |
| (Missing) | 29 (1.1%) |
| ^1^n (%) | |

# Table S28b. Intensity by demographic category for Germany conditional on smokers

| Variable | Category | Intensity | 95% CI | SE | Global p-value |
| --- | --- | --- | --- | --- | --- |
| Age group | 18-24 | 10.16 | (8.50, 11.82) | 0.84 | < .001 |
|  | 25-34 | 11.32 | (10.32, 12.31) | 0.51 |  |
|  | 35-44 | 13.58 | (12.48, 14.67) | 0.56 |  |
|  | 45-54 | 14.28 | (13.21, 15.34) | 0.54 |  |
|  | 55-64 | 15.91 | (14.78, 17.04) | 0.58 |  |
|  | 65-74 | 14.91 | (13.63, 16.18) | 0.65 |  |
|  | 75-84 | 10.44 | (8.79, 12.08) | 0.83 |  |
|  | 85 or older | 5.61 | * | * |  |
| Gender | Male | 14.34 | (13.65, 15.03) | 0.35 | < .001 |
|  | Female | 13.17 | (12.51, 13.82) | 0.34 |  |
|  | Other | 6.49 | * | * |  |
| Marital status | Married | 13.90 | (13.18, 14.62) | 0.37 | < .001 |
|  | Separated | 12.92 | (10.21, 15.63) | 1.36 |  |
|  | Divorced | 15.24 | (14.02, 16.47) | 0.62 |  |
|  | Widowed | 15.54 | (13.37, 17.72) | 1.09 |  |
|  | Never | 13.42 | (12.49, 14.35) | 0.47 |  |
|  | Domestic Partner | 11.26 | (9.80, 12.72) | 0.74 |  |
| Employment | Employed for an employer | 14.03 | (13.37, 14.70) | 0.34 | < .001 |
|  | Self-employed | 13.17 | (11.33, 15.00) | 0.93 |  |
|  | Retired | 13.75 | (12.80, 14.70) | 0.48 |  |
|  | Student | 7.92 | (6.21, 9.63) | 0.86 |  |
|  | Homemaker | 15.05 | (12.36, 17.74) | 1.35 |  |
|  | Unemployed and looking for a job | 14.70 | (12.90, 16.51) | 0.91 |  |
|  | None of these/Other | 14.02 | (11.77, 16.27) | 1.12 |  |
| Religious service attendance | More than 1/week | 13.89 | (10.92, 16.87) | 1.49 | 0.001 |
|  | 1/week | 10.26 | (8.49, 12.04) | 0.90 |  |
|  | 1-3/month | 14.82 | (12.01, 17.63) | 1.42 |  |
|  | A few times a year | 13.22 | (12.31, 14.12) | 0.46 |  |
|  | Never | 14.08 | (13.50, 14.65) | 0.29 |  |
| Education | Up to 8 years | 13.34 | (10.72, 15.96) | 1.31 | < .001 |
|  | 9-15 years | 14.37 | (13.81, 14.93) | 0.28 |  |
|  | 16+ years | 11.93 | (10.96, 12.89) | 0.49 |  |
| Immigration status | Born in this country | 13.89 | (13.39, 14.39) | 0.25 | 0.015 |
|  | Born in another country | 11.88 | (10.33, 13.43) | 0.78 |  |
| Religious affiliation | Christianity | 13.88 | (13.31, 14.45) | 0.29 | < .001 |
|  | Islam | 10.39 | (7.65, 13.12) | 1.38 |  |
|  | Hinduism | 7.90 | * | * |  |
|  | Buddhism | 13.83 | (3.77, 23.89) | 3.27 |  |
|  | Judaism | 13.47 | * | * |  |
|  | Sikhism | 8.93 | * | * |  |
|  | Baha'i | 4.70 | * | * |  |
|  | Taoism | 23.46 | * | * |  |
|  | Primal, Animist, or Folk religion | 10.70 | * | * |  |
|  | Some other religion | 17.96 | (12.47, 23.46) | 2.34 |  |
|  | No religion/Atheist/Agnostic | 13.93 | (13.09, 14.78) | 0.43 |  |

# Table S29a. Descriptive statistics for Hong Kong conditional on smokers

| **Characteristic** | **N = 956**^1^ |
| --- | --- |
| **Age group** |  |
| 18-24 | 91 (9.5%) |
| 25-34 | 167 (17%) |
| 35-44 | 161 (17%) |
| 45-54 | 257 (27%) |
| 55-64 | 237 (25%) |
| 65-74 | 43 (4.5%) |
| 75-84 | 0 (0%) |
| 85 or older | 0 (0%) |
| (Missing) | 0 (0%) |
| **Gender** |  |
| Male | 528 (55%) |
| Female | 428 (45%) |
| Other | 0 (0%) |
| (Missing) | 0 (0%) |
| **Marital status** |  |
| Married | 780 (82%) |
| Separated | 7 (0.8%) |
| Divorced | 31 (3.3%) |
| Widowed | 2 (0.2%) |
| Single, never married | 130 (14%) |
| Domestic Partner | 6 (0.7%) |
| (Missing) | 0 (0%) |
| **Employment** |  |
| Employed for an employer | 750 (78%) |
| Self-employed | 105 (11%) |
| Retired | 42 (4.4%) |
| Student | 6 (0.7%) |
| Homemaker | 28 (2.9%) |
| Unemployed and looking for a job | 13 (1.4%) |
| None of these/Other | 8 (0.8%) |
| (Missing) | 4 (0.4%) |
| **Religious service attendance** |  |
| More than 1/week | 154 (16%) |
| 1/week | 237 (25%) |
| 1-3/month | 179 (19%) |
| A few times a year | 136 (14%) |
| Never | 249 (26%) |
| (Missing) | 0 (0%) |
| **Education** |  |
| Up to 8 years | 185 (19%) |
| 9-15 years | 699 (73%) |
| 16+ years | 72 (7.6%) |
| (Missing) | 0 (0%) |
| **Immigration** |  |
| Born in this country | 894 (94%) |
| Born in another country | 49 (5.2%) |
| (Missing) | 13 (1.3%) |
| **Religious affiliation** |  |
| Christianity | 271 (28%) |
| Islam | 42 (4.4%) |
| Hinduism | 16 (1.7%) |
| Buddhism | 177 (18%) |
| Judaism | 10 (1.0%) |
| Sikhism | 2 (0.2%) |
| Baha'i | 3 (0.3%) |
| Jainism | 1 (<0.1%) |
| Shinto | 2 (0.2%) |
| Taoism | 28 (3.0%) |
| Confucianism | 6 (0.6%) |
| Primal, Animist, or Folk religion | 7 (0.8%) |
| Spiritism | 0 (0%) |
| Umbanda, Candomble, and other African-derived religions | 0 (0%) |
| Chinese folk/traditional religion | 44 (4.6%) |
| Some other religion | 2 (0.2%) |
| No religion/Atheist/Agnostic | 343 (36%) |
| (Missing) | 3 (0.3%) |
| **Race/Ethnicity** |  |
| Chinese (Cantonese) | 573 (60%) |
| Chinese (Chaoshan) | 87 (9.1%) |
| Chinese (Fujianese) | 63 (6.6%) |
| Chinese (Hakka) | 37 (3.9%) |
| Chinese (Other ethnicity) | 52 (5.4%) |
| Chinese (Shanghainese) | 30 (3.2%) |
| East Asian (Korean, Japanese) | 8 (0.8%) |
| Other | 0 (0%) |
| South Asian (Indian, Nepalese, Pakistani) | 5 (0.6%) |
| Southeast Asian (Filipino, Indonesian, Thailand) | 2 (0.2%) |
| Taiwanese | 5 (0.5%) |
| White | 3 (0.3%) |
| (Missing) | 90 (9.4%) |
| ^1^n (%) | |

# Table S29b. Intensity by demographic category for Hong Kong conditional on smokers

| Variable | Category | Intensity | 95% CI | SE | Global p-value |
| --- | --- | --- | --- | --- | --- |
| Age group | 18-24 | 4.95 | (4.17, 5.73) | 0.39 | < .001 |
|  | 25-34 | 5.59 | (4.41, 6.77) | 0.59 |  |
|  | 35-44 | 6.91 | (5.89, 7.93) | 0.52 |  |
|  | 45-54 | 8.08 | (7.00, 9.16) | 0.55 |  |
|  | 55-64 | 5.41 | (4.93, 5.89) | 0.24 |  |
|  | 65-74 | 6.74 | (2.46, 11.02) | 2.11 |  |
| Gender | Male | 6.30 | (5.71, 6.89) | 0.30 | 0.531 |
|  | Female | 6.60 | (5.81, 7.39) | 0.40 |  |
| Marital status | Married | 6.10 | (5.58, 6.62) | 0.26 | < .001 |
|  | Separated | 5.19 | * | * |  |
|  | Divorced | 8.30 | (3.55, 13.05) | 2.29 |  |
|  | Widowed | 15.00 | * | * |  |
|  | Never | 7.85 | (6.51, 9.20) | 0.68 |  |
|  | Domestic Partner | 7.44 | * | * |  |
| Employment | Employed for an employer | 6.31 | (5.84, 6.79) | 0.24 | 0.123 |
|  | Self-employed | 6.34 | (4.96, 7.71) | 0.69 |  |
|  | Retired | 8.23 | (3.66, 12.81) | 2.25 |  |
|  | Student | 4.27 | * | * |  |
|  | Homemaker | 5.39 | (2.80, 7.98) | 1.14 |  |
|  | Unemployed and looking for a job | 10.81 | (3.28, 18.34) | 2.81 |  |
|  | None of these/Other | 5.10 | * | * |  |
| Religious service attendance | More than 1/week | 5.04 | (4.27, 5.81) | 0.39 | < .001 |
|  | 1/week | 6.98 | (5.69, 8.27) | 0.65 |  |
|  | 1-3/month | 6.24 | (5.04, 7.45) | 0.61 |  |
|  | A few times a year | 5.59 | (4.74, 6.44) | 0.43 |  |
|  | Never | 7.41 | (6.61, 8.20) | 0.40 |  |
| Education | Up to 8 years | 8.89 | (7.11, 10.67) | 0.90 | 0.003 |
|  | 9-15 years | 5.83 | (5.43, 6.23) | 0.20 |  |
|  | 16+ years | 6.09 | (5.13, 7.06) | 0.48 |  |
| Immigration status | Born in this country | 6.36 | (5.88, 6.84) | 0.25 | 0.271 |
|  | Born in another country | 7.86 | (4.79, 10.93) | 1.49 |  |
| Religious affiliation | Christianity | 7.49 | (6.29, 8.69) | 0.61 | < .001 |
|  | Islam | 7.01 | (3.43, 10.58) | 1.65 |  |
|  | Hinduism | 3.37 | * | * |  |
|  | Buddhism | 5.06 | (4.39, 5.73) | 0.34 |  |
|  | Judaism | 3.73 | * | * |  |
|  | Sikhism | 5.29 | * | * |  |
|  | Baha'i | 5.29 | * | * |  |
|  | Jainism | 3.00 | * | * |  |
|  | Shinto | 5.04 | * | * |  |
|  | Taoism | 5.52 | (3.27, 7.77) | 1.02 |  |
|  | Confucianism | 7.10 | * | * |  |
|  | Primal, Animist, or Folk religion | 7.73 | * | * |  |
|  | Chinese folk/traditional religion | 6.85 | (5.04, 8.67) | 0.88 |  |
|  | Some other religion | 3.00 | * | * |  |
|  | No religion/Atheist/Agnostic | 6.49 | (5.86, 7.11) | 0.32 |  |
| Race/Ethnicity | White | 3.61 | * | * | < .001 |
|  | Chinese (Cantonese) | 6.87 | (6.19, 7.54) | 0.34 |  |
|  | Chinese (Chaoshan) | 6.34 | (5.24, 7.43) | 0.54 |  |
|  | Chinese (Fujianese) | 5.65 | (4.38, 6.91) | 0.63 |  |
|  | Chinese (Hakka) | 4.69 | (3.81, 5.57) | 0.43 |  |
|  | Chinese (Shanghainese) | 4.10 | (2.76, 5.44) | 0.64 |  |
|  | Chinese (Other ethnicity) | 5.43 | (3.98, 6.88) | 0.72 |  |
|  | East Asian (Korean, Japanese) | 5.55 | * | * |  |
|  | Southeast Asian (Filipino, Indonesian, Thailand) | 4.87 | * | * |  |
|  | South Asian (Indian, Nepalese, Pakistani) | 12.50 | * | * |  |
|  | Taiwanese | 5.22 | * | * |  |

# Table S30a. Descriptive statistics for India conditional on smokers

| **Characteristic** | **N = 1,484**^1^ |
| --- | --- |
| **Age group** |  |
| 18-24 | 231 (16%) |
| 25-34 | 345 (23%) |
| 35-44 | 338 (23%) |
| 45-54 | 273 (18%) |
| 55-64 | 200 (13%) |
| 65-74 | 77 (5.2%) |
| 75-84 | 16 (1.1%) |
| 85 or older | 3 (0.2%) |
| (Missing) | 0 (0%) |
| **Gender** |  |
| Male | 1,126 (76%) |
| Female | 358 (24%) |
| Other | 0 (0%) |
| (Missing) | 0 (0%) |
| **Marital status** |  |
| Married | 1,128 (76%) |
| Separated | 5 (0.3%) |
| Divorced | 3 (0.2%) |
| Widowed | 28 (1.9%) |
| Single, never married | 229 (15%) |
| Domestic Partner | 62 (4.2%) |
| (Missing) | 28 (1.9%) |
| **Employment** |  |
| Employed for an employer | 404 (27%) |
| Self-employed | 472 (32%) |
| Retired | 53 (3.6%) |
| Student | 49 (3.3%) |
| Homemaker | 269 (18%) |
| Unemployed and looking for a job | 129 (8.7%) |
| None of these/Other | 102 (6.9%) |
| (Missing) | 5 (0.3%) |
| **Religious service attendance** |  |
| More than 1/week | 350 (24%) |
| 1/week | 368 (25%) |
| 1-3/month | 346 (23%) |
| A few times a year | 219 (15%) |
| Never | 190 (13%) |
| (Missing) | 11 (0.7%) |
| **Education** |  |
| Up to 8 years | 1,329 (90%) |
| 9-15 years | 142 (9.6%) |
| 16+ years | 13 (0.9%) |
| (Missing) | 0 (0%) |
| **Immigration** |  |
| Born in this country | 1,458 (98%) |
| Born in another country | 23 (1.6%) |
| (Missing) | 3 (0.2%) |
| **Religious affiliation** |  |
| Christianity | 34 (2.3%) |
| Islam | 185 (12%) |
| Hinduism | 1,231 (83%) |
| Buddhism | 9 (0.6%) |
| Judaism | 0 (0%) |
| Sikhism | 8 (0.5%) |
| Baha'i | 0 (0%) |
| Jainism | 1 (<0.1%) |
| Shinto | 0 (0%) |
| Taoism | 0 (0%) |
| Confucianism | 0 (0%) |
| Primal, Animist, or Folk religion | 3 (0.2%) |
| Spiritism | 0 (0%) |
| Umbanda, Candomble, and other African-derived religions | 0 (0%) |
| Chinese folk/traditional religion | 0 (0%) |
| Some other religion | 0 (<0.1%) |
| No religion/Atheist/Agnostic | 3 (0.2%) |
| (Missing) | 10 (0.7%) |
| **Race/Ethnicity** |  |
| General | 405 (27%) |
| Other backward caste | 478 (32%) |
| Schedule caste | 460 (31%) |
| Schedule tribe | 118 (8.0%) |
| (Missing) | 23 (1.6%) |
| ^1^n (%) | |

# Table S30b. Intensity by demographic category for India conditional on smokers

| Variable | Category | Intensity | 95% CI | SE | Global p-value |
| --- | --- | --- | --- | --- | --- |
| Age group | 18-24 | 6.31 | (2.48, 10.15) | 1.94 | < .001 |
|  | 25-34 | 8.02 | (4.97, 11.07) | 1.55 |  |
|  | 35-44 | 7.16 | (5.47, 8.86) | 0.85 |  |
|  | 45-54 | 10.58 | (8.07, 13.09) | 1.27 |  |
|  | 55-64 | 9.88 | (7.51, 12.26) | 1.20 |  |
|  | 65-74 | 6.52 | (4.18, 8.86) | 1.16 |  |
|  | 75-84 | 11.65 | (0.10, 23.21) | 3.45 |  |
|  | 85 or older | 2.50 | * | * |  |
| Gender | Male | 8.13 | (6.96, 9.30) | 0.60 | 0.504 |
|  | Female | 10.47 | (3.58, 17.35) | 3.44 |  |
| Marital status | Married | 7.93 | (6.94, 8.93) | 0.51 | < .001 |
|  | Separated | 9.72 | (0.00, 30.39) | 2.54 |  |
|  | Divorced | 3.00 | * | * |  |
|  | Widowed | 17.17 | (4.58, 29.76) | 5.69 |  |
|  | Never | 9.58 | (4.70, 14.46) | 2.47 |  |
|  | Domestic Partner | 4.68 | (1.68, 7.68) | 1.45 |  |
| Employment | Employed for an employer | 6.01 | (4.73, 7.28) | 0.64 | 0.014 |
|  | Self-employed | 9.56 | (7.45, 11.67) | 1.07 |  |
|  | Retired | 8.55 | (5.56, 11.54) | 1.46 |  |
|  | Student | 5.71 | (1.09, 10.32) | 2.18 |  |
|  | Homemaker | 10.29 | (4.17, 16.41) | 3.06 |  |
|  | Unemployed and looking for a job | 9.44 | (3.09, 15.80) | 3.19 |  |
|  | None of these/Other | 9.19 | (6.22, 12.17) | 1.47 |  |
| Religious service attendance | More than 1/week | 9.53 | (6.88, 12.18) | 1.34 | 0.364 |
|  | 1/week | 8.02 | (5.46, 10.57) | 1.30 |  |
|  | 1-3/month | 6.69 | (4.90, 8.49) | 0.91 |  |
|  | A few times a year | 8.64 | (6.31, 10.97) | 1.18 |  |
|  | Never | 8.84 | (4.64, 13.04) | 2.12 |  |
| Education | Up to 8 years | 8.44 | (7.19, 9.69) | 0.64 | 0.175 |
|  | 9-15 years | 6.83 | (4.42, 9.23) | 1.21 |  |
|  | 16+ years | 5.51 | * | * |  |
| Immigration status | Born in this country | 8.19 | (7.02, 9.37) | 0.60 | 0.325 |
|  | Born in another country | 12.32 | (2.27, 22.37) | 4.62 |  |
| Religious affiliation | Christianity | 11.27 | (0.00, 25.46) | 5.97 | < .001 |
|  | Islam | 10.56 | (5.74, 15.39) | 2.44 |  |
|  | Hinduism | 7.62 | (6.69, 8.55) | 0.47 |  |
|  | Buddhism | 4.35 | * | * |  |
|  | Sikhism | 2.82 | * | * |  |
|  | Jainism | 2.00 | * | * |  |
|  | Primal, Animist, or Folk religion | 1.35 | * | * |  |
|  | Some other religion | 2.00 | * | * |  |
|  | No religion/Atheist/Agnostic | 97.00 | * | * |  |
| Race/Ethnicity | General | 9.55 | (6.76, 12.33) | 1.41 | 0.546 |
|  | Other backward caste | 7.41 | (5.43, 9.40) | 1.01 |  |
|  | Schedule caste | 7.77 | (6.54, 9.01) | 0.63 |  |
|  | Schedule tribe | 9.26 | (4.21, 14.31) | 2.51 |  |

# Table S31a. Descriptive statistics for Indonesia conditional on smokers

| **Characteristic** | **N = 2,944**^1^ |
| --- | --- |
| **Age group** |  |
| 18-24 | 477 (16%) |
| 25-34 | 740 (25%) |
| 35-44 | 709 (24%) |
| 45-54 | 564 (19%) |
| 55-64 | 372 (13%) |
| 65-74 | 77 (2.6%) |
| 75-84 | 0 (0%) |
| 85 or older | 6 (0.2%) |
| (Missing) | 0 (0%) |
| **Gender** |  |
| Male | 2,496 (85%) |
| Female | 441 (15%) |
| Other | 4 (0.1%) |
| (Missing) | 3 (0.1%) |
| **Marital status** |  |
| Married | 2,031 (69%) |
| Separated | 37 (1.3%) |
| Divorced | 70 (2.4%) |
| Widowed | 115 (3.9%) |
| Single, never married | 655 (22%) |
| Domestic Partner | 9 (0.3%) |
| (Missing) | 27 (0.9%) |
| **Employment** |  |
| Employed for an employer | 742 (25%) |
| Self-employed | 1,225 (42%) |
| Retired | 30 (1.0%) |
| Student | 81 (2.8%) |
| Homemaker | 286 (9.7%) |
| Unemployed and looking for a job | 290 (9.9%) |
| None of these/Other | 280 (9.5%) |
| (Missing) | 10 (0.3%) |
| **Religious service attendance** |  |
| More than 1/week | 1,192 (40%) |
| 1/week | 1,074 (36%) |
| 1-3/month | 284 (9.7%) |
| A few times a year | 249 (8.4%) |
| Never | 139 (4.7%) |
| (Missing) | 6 (0.2%) |
| **Education** |  |
| Up to 8 years | 1,320 (45%) |
| 9-15 years | 1,499 (51%) |
| 16+ years | 125 (4.2%) |
| (Missing) | 1 (<0.1%) |
| **Immigration** |  |
| Born in this country | 2,932 (100%) |
| Born in another country | 12 (0.4%) |
| (Missing) | 0 (0%) |
| **Religious affiliation** |  |
| Christianity | 180 (6.1%) |
| Islam | 2,745 (93%) |
| Hinduism | 16 (0.5%) |
| Buddhism | 2 (<0.1%) |
| Judaism | 0 (0%) |
| Sikhism | 0 (0%) |
| Baha'i | 0 (0%) |
| Jainism | 0 (0%) |
| Shinto | 0 (0%) |
| Taoism | 0 (0%) |
| Confucianism | 0 (0%) |
| Primal, Animist, or Folk religion | 0 (0%) |
| Spiritism | 0 (0%) |
| Umbanda, Candomble, and other African-derived religions | 0 (0%) |
| Chinese folk/traditional religion | 0 (0%) |
| Some other religion | 0 (0%) |
| No religion/Atheist/Agnostic | 0 (0%) |
| (Missing) | 1 (<0.1%) |
| **Race/Ethnicity** |  |
| Bali | 15 (0.5%) |
| Banjar/Melayu Banjar | 126 (4.3%) |
| Batak | 71 (2.4%) |
| Betawi | 110 (3.7%) |
| Bugis | 98 (3.3%) |
| Jawa | 1,164 (40%) |
| Madura | 116 (3.9%) |
| Makasar | 36 (1.2%) |
| Minangkabau | 117 (4.0%) |
| Other | 484 (16%) |
| Sunda/Parahyangan | 593 (20%) |
| (Missing) | 15 (0.5%) |
| ^1^n (%) | |

# Table S31b. Intensity by demographic category for Indonesia conditional on smokers

| Variable | Category | Intensity | 95% CI | SE | Global p-value |
| --- | --- | --- | --- | --- | --- |
| Age group | 18-24 | 8.85 | (7.89, 9.81) | 0.49 | 0.018 |
|  | 25-34 | 10.51 | (9.84, 11.18) | 0.34 |  |
|  | 35-44 | 10.89 | (10.04, 11.74) | 0.43 |  |
|  | 45-54 | 9.82 | (9.10, 10.53) | 0.36 |  |
|  | 55-64 | 10.18 | (8.98, 11.38) | 0.61 |  |
|  | 65-74 | 9.72 | (7.28, 12.15) | 1.19 |  |
|  | 85 or older | 8.50 | * | * |  |
| Gender | Male | 10.38 | (9.97, 10.79) | 0.21 | < .001 |
|  | Female | 7.10 | (5.88, 8.32) | 0.62 |  |
|  | Other | 6.00 | * | * |  |
| Marital status | Married | 10.28 | (9.84, 10.72) | 0.23 | 0.028 |
|  | Separated | 11.32 | (9.38, 13.27) | 0.95 |  |
|  | Divorced | 9.52 | (7.92, 11.12) | 0.79 |  |
|  | Widowed | 10.65 | (8.81, 12.48) | 0.92 |  |
|  | Never | 9.72 | (8.83, 10.61) | 0.45 |  |
|  | Domestic Partner | 4.93 | * | * |  |
| Employment | Employed for an employer | 10.28 | (9.52, 11.05) | 0.39 | < .001 |
|  | Self-employed | 10.45 | (9.88, 11.01) | 0.29 |  |
|  | Retired | 9.86 | (7.28, 12.44) | 1.23 |  |
|  | Student | 7.63 | (6.00, 9.27) | 0.81 |  |
|  | Homemaker | 7.32 | (5.70, 8.95) | 0.82 |  |
|  | Unemployed and looking for a job | 9.62 | (8.54, 10.71) | 0.55 |  |
|  | None of these/Other | 11.04 | (9.87, 12.21) | 0.59 |  |
| Religious service attendance | More than 1/week | 9.97 | (9.44, 10.49) | 0.27 | 0.810 |
|  | 1/week | 10.12 | (9.45, 10.80) | 0.34 |  |
|  | 1-3/month | 10.59 | (9.35, 11.84) | 0.63 |  |
|  | A few times a year | 10.35 | (8.99, 11.71) | 0.69 |  |
|  | Never | 10.81 | (8.59, 13.03) | 1.12 |  |
| Education | Up to 8 years | 10.35 | (9.69, 11.02) | 0.34 | 0.324 |
|  | 9-15 years | 9.93 | (9.48, 10.37) | 0.23 |  |
|  | 16+ years | 10.66 | (9.22, 12.10) | 0.73 |  |
| Immigration status | Born in this country | 10.17 | (9.76, 10.57) | 0.21 | 0.004 |
|  | Born in another country | 6.51 | (3.15, 9.86) | 1.34 |  |
| Religious affiliation | Christianity | 10.55 | (7.87, 13.23) | 1.36 | < .001 |
|  | Islam | 10.13 | (9.74, 10.52) | 0.20 |  |
|  | Hinduism | 10.25 | (5.91, 14.58) | 1.75 |  |
|  | Buddhism | 5.00 | * | * |  |
| Race/Ethnicity | Banjar/Melayu Banjar | 10.86 | (7.83, 13.88) | 1.52 | 0.040 |
|  | Betawi | 12.94 | (10.28, 15.60) | 1.33 |  |
|  | Bugis | 9.70 | (8.01, 11.39) | 0.84 |  |
|  | Jawa | 9.95 | (9.38, 10.53) | 0.29 |  |
|  | Madura | 11.11 | (9.13, 13.10) | 0.98 |  |
|  | Minangkabau | 11.31 | (10.22, 12.39) | 0.55 |  |
|  | Sunda/Parahyangan | 9.78 | (8.88, 10.68) | 0.46 |  |
|  | Bali | 9.86 | (0.61, 19.11) | 1.64 |  |
|  | Batak | 12.97 | (8.20, 17.75) | 2.38 |  |
|  | Makasar | 10.96 | (7.83, 14.10) | 1.46 |  |
|  | Other | 9.39 | (8.56, 10.22) | 0.42 |  |

# Table S32a. Descriptive statistics for Israel conditional on smokers

| **Characteristic** | **N = 882**^1^ |
| --- | --- |
| **Age group** |  |
| 18-24 | 99 (11%) |
| 25-34 | 186 (21%) |
| 35-44 | 209 (24%) |
| 45-54 | 160 (18%) |
| 55-64 | 134 (15%) |
| 65-74 | 74 (8.3%) |
| 75-84 | 18 (2.0%) |
| 85 or older | 3 (0.3%) |
| (Missing) | 0 (0%) |
| **Gender** |  |
| Male | 623 (71%) |
| Female | 255 (29%) |
| Other | 0 (<0.1%) |
| (Missing) | 4 (0.5%) |
| **Marital status** |  |
| Married | 503 (57%) |
| Separated | 17 (1.9%) |
| Divorced | 88 (10%) |
| Widowed | 24 (2.7%) |
| Single, never married | 181 (21%) |
| Domestic Partner | 52 (5.9%) |
| (Missing) | 16 (1.8%) |
| **Employment** |  |
| Employed for an employer | 466 (53%) |
| Self-employed | 153 (17%) |
| Retired | 91 (10%) |
| Student | 80 (9.0%) |
| Homemaker | 18 (2.1%) |
| Unemployed and looking for a job | 42 (4.8%) |
| None of these/Other | 32 (3.6%) |
| (Missing) | 1 (<0.1%) |
| **Religious service attendance** |  |
| More than 1/week | 142 (16%) |
| 1/week | 99 (11%) |
| 1-3/month | 82 (9.3%) |
| A few times a year | 257 (29%) |
| Never | 298 (34%) |
| (Missing) | 5 (0.6%) |
| **Education** |  |
| Up to 8 years | 57 (6.5%) |
| 9-15 years | 392 (44%) |
| 16+ years | 432 (49%) |
| (Missing) | 1 (0.1%) |
| **Immigration** |  |
| Born in this country | 673 (76%) |
| Born in another country | 209 (24%) |
| (Missing) | 1 (<0.1%) |
| **Religious affiliation** |  |
| Christianity | 17 (1.9%) |
| Islam | 172 (19%) |
| Hinduism | 0 (0%) |
| Buddhism | 0 (0%) |
| Judaism | 669 (76%) |
| Sikhism | 0 (0%) |
| Baha'i | 0 (0%) |
| Jainism | 0 (0%) |
| Shinto | 0 (0%) |
| Taoism | 0 (0%) |
| Confucianism | 0 (0%) |
| Primal, Animist, or Folk religion | 0 (0%) |
| Spiritism | 0 (0%) |
| Umbanda, Candomble, and other African-derived religions | 0 (0%) |
| Chinese folk/traditional religion | 0 (0%) |
| Some other religion | 2 (0.2%) |
| No religion/Atheist/Agnostic | 22 (2.5%) |
| (Missing) | 1 (<0.1%) |
| **Race/Ethnicity** |  |
| Arab | 182 (21%) |
| Jewish | 679 (77%) |
| Other | 13 (1.5%) |
| (Missing) | 9 (1.0%) |
| ^1^n (%) | |

# Table S32b. Intensity by demographic category for Israel conditional on smokers

| Variable | Category | Intensity | 95% CI | SE | Global p-value |
| --- | --- | --- | --- | --- | --- |
| Age group | 18-24 | 10.69 | (8.84, 12.54) | 0.93 | < .001 |
|  | 25-34 | 13.35 | (10.29, 16.41) | 1.55 |  |
|  | 35-44 | 13.57 | (11.92, 15.21) | 0.83 |  |
|  | 45-54 | 12.85 | (11.35, 14.34) | 0.76 |  |
|  | 55-64 | 14.09 | (12.24, 15.94) | 0.93 |  |
|  | 65-74 | 13.62 | (11.08, 16.15) | 1.27 |  |
|  | 75-84 | 12.31 | (5.37, 19.25) | 2.85 |  |
|  | 85 or older | 6.28 | * | * |  |
| Gender | Male | 14.55 | (13.18, 15.92) | 0.70 | < .001 |
|  | Female | 9.57 | (8.54, 10.60) | 0.52 |  |
|  | Other | 3.00 | * | * |  |
| Marital status | Married | 13.30 | (12.07, 14.52) | 0.62 | < .001 |
|  | Separated | 12.40 | (7.79, 17.00) | 2.02 |  |
|  | Divorced | 14.92 | (12.93, 16.91) | 1.00 |  |
|  | Widowed | 23.24 | (4.37, 42.11) | 8.94 |  |
|  | Never | 11.56 | (10.10, 13.02) | 0.74 |  |
|  | Domestic Partner | 8.89 | (6.93, 10.86) | 0.97 |  |
| Employment | Employed for an employer | 12.49 | (11.23, 13.74) | 0.64 | 0.482 |
|  | Self-employed | 14.92 | (10.90, 18.94) | 2.03 |  |
|  | Retired | 13.75 | (11.19, 16.31) | 1.29 |  |
|  | Student | 11.84 | (9.61, 14.08) | 1.12 |  |
|  | Homemaker | 12.62 | (8.43, 16.82) | 1.85 |  |
|  | Unemployed and looking for a job | 14.94 | (11.64, 18.23) | 1.61 |  |
|  | None of these/Other | 12.92 | (9.18, 16.66) | 1.80 |  |
| Religious service attendance | More than 1/week | 13.19 | (12.08, 14.31) | 0.56 | 0.168 |
|  | 1/week | 11.53 | (9.18, 13.88) | 1.18 |  |
|  | 1-3/month | 11.99 | (9.84, 14.15) | 1.08 |  |
|  | A few times a year | 12.33 | (10.80, 13.86) | 0.77 |  |
|  | Never | 14.56 | (12.28, 16.84) | 1.16 |  |
| Education | Up to 8 years | 18.58 | (15.33, 21.83) | 1.62 | < .001 |
|  | 9-15 years | 14.80 | (13.00, 16.61) | 0.92 |  |
|  | 16+ years | 10.84 | (9.79, 11.88) | 0.53 |  |
| Immigration status | Born in this country | 13.60 | (12.24, 14.95) | 0.69 | 0.017 |
|  | Born in another country | 11.49 | (10.19, 12.79) | 0.66 |  |
| Religious affiliation | Christianity | 16.00 | (7.40, 24.59) | 3.81 | < .001 |
|  | Islam | 19.27 | (15.62, 22.92) | 1.85 |  |
|  | Judaism | 11.30 | (10.38, 12.22) | 0.47 |  |
|  | Some other religion | 20.00 | * | * |  |
|  | No religion/Atheist/Agnostic | 17.61 | (12.06, 23.17) | 2.60 |  |
| Race/Ethnicity | Arab | 19.26 | (15.75, 22.76) | 1.78 | < .001 |
|  | Jewish | 11.33 | (10.40, 12.26) | 0.47 |  |
|  | Other | 19.77 | (14.81, 24.74) | 1.99 |  |

# Table S33a. Descriptive statistics for Japan conditional on smokers

| **Characteristic** | **N = 4,766**^1^ |
| --- | --- |
| **Age group** |  |
| 18-24 | 199 (4.2%) |
| 25-34 | 496 (10%) |
| 35-44 | 883 (19%) |
| 45-54 | 1,067 (22%) |
| 55-64 | 892 (19%) |
| 65-74 | 1,044 (22%) |
| 75-84 | 175 (3.7%) |
| 85 or older | 10 (0.2%) |
| (Missing) | 0 (0%) |
| **Gender** |  |
| Male | 3,170 (67%) |
| Female | 1,582 (33%) |
| Other | 3 (<0.1%) |
| (Missing) | 11 (0.2%) |
| **Marital status** |  |
| Married | 2,832 (59%) |
| Separated | 73 (1.5%) |
| Divorced | 671 (14%) |
| Widowed | 159 (3.3%) |
| Single, never married | 962 (20%) |
| Domestic Partner | 53 (1.1%) |
| (Missing) | 15 (0.3%) |
| **Employment** |  |
| Employed for an employer | 2,927 (61%) |
| Self-employed | 554 (12%) |
| Retired | 463 (9.7%) |
| Student | 30 (0.6%) |
| Homemaker | 142 (3.0%) |
| Unemployed and looking for a job | 149 (3.1%) |
| None of these/Other | 499 (10%) |
| (Missing) | 2 (<0.1%) |
| **Religious service attendance** |  |
| More than 1/week | 87 (1.8%) |
| 1/week | 124 (2.6%) |
| 1-3/month | 246 (5.2%) |
| A few times a year | 843 (18%) |
| Never | 3,445 (72%) |
| (Missing) | 21 (0.4%) |
| **Education** |  |
| Up to 8 years | 169 (3.5%) |
| 9-15 years | 3,719 (78%) |
| 16+ years | 879 (18%) |
| (Missing) | 0 (0%) |
| **Immigration** |  |
| Born in this country | 4,505 (95%) |
| Born in another country | 46 (1.0%) |
| (Missing) | 215 (4.5%) |
| **Religious affiliation** |  |
| Christianity | 76 (1.6%) |
| Islam | 6 (0.1%) |
| Hinduism | 5 (<0.1%) |
| Buddhism | 1,612 (34%) |
| Judaism | 9 (0.2%) |
| Sikhism | 6 (0.1%) |
| Baha'i | 2 (<0.1%) |
| Jainism | 9 (0.2%) |
| Shinto | 108 (2.3%) |
| Taoism | 0 (0%) |
| Confucianism | 5 (0.1%) |
| Primal, Animist, or Folk religion | 9 (0.2%) |
| Spiritism | 0 (0%) |
| Umbanda, Candomble, and other African-derived religions | 0 (0%) |
| Chinese folk/traditional religion | 0 (0%) |
| Some other religion | 9 (0.2%) |
| No religion/Atheist/Agnostic | 2,833 (59%) |
| (Missing) | 79 (1.7%) |
| ^1^n (%) | |

# Table S33b. Intensity by demographic category for Japan conditional on smokers

| Variable | Category | Intensity | 95% CI | SE | Global p-value |
| --- | --- | --- | --- | --- | --- |
| Age group | 18-24 | 7.63 | (6.01, 9.25) | 0.82 | < .001 |
|  | 25-34 | 11.39 | (10.49, 12.30) | 0.46 |  |
|  | 35-44 | 13.65 | (13.05, 14.25) | 0.30 |  |
|  | 45-54 | 14.63 | (14.09, 15.18) | 0.28 |  |
|  | 55-64 | 15.30 | (14.70, 15.90) | 0.31 |  |
|  | 65-74 | 13.18 | (12.58, 13.78) | 0.31 |  |
|  | 75-84 | 12.51 | (11.05, 13.96) | 0.74 |  |
|  | 85 or older | 9.21 | (0.00, 19.70) | 2.03 |  |
| Gender | Male | 14.21 | (13.87, 14.55) | 0.17 | < .001 |
|  | Female | 12.19 | (11.72, 12.66) | 0.24 |  |
|  | Other | 10.52 | * | * |  |
| Marital status | Married | 13.54 | (13.19, 13.88) | 0.17 | 0.009 |
|  | Separated | 9.77 | (7.06, 12.48) | 1.36 |  |
|  | Divorced | 14.49 | (13.68, 15.30) | 0.41 |  |
|  | Widowed | 12.43 | (10.64, 14.22) | 0.91 |  |
|  | Never | 13.49 | (12.87, 14.12) | 0.32 |  |
|  | Domestic Partner | 12.75 | (10.81, 14.69) | 0.96 |  |
| Employment | Employed for an employer | 13.54 | (13.19, 13.89) | 0.18 | 0.008 |
|  | Self-employed | 14.67 | (13.83, 15.52) | 0.43 |  |
|  | Retired | 12.29 | (11.50, 13.08) | 0.40 |  |
|  | Student | 11.53 | (0.61, 22.44) | 5.02 |  |
|  | Homemaker | 13.31 | (11.72, 14.89) | 0.79 |  |
|  | Unemployed and looking for a job | 13.54 | (12.33, 14.74) | 0.61 |  |
|  | None of these/Other | 13.80 | (12.95, 14.66) | 0.44 |  |
| Religious service attendance | More than 1/week | 12.56 | (10.46, 14.66) | 1.05 | < .001 |
|  | 1/week | 9.54 | (7.93, 11.14) | 0.81 |  |
|  | 1-3/month | 10.38 | (9.14, 11.63) | 0.63 |  |
|  | A few times a year | 13.34 | (12.68, 14.00) | 0.34 |  |
|  | Never | 14.02 | (13.70, 14.34) | 0.16 |  |
| Education | Up to 8 years | 15.12 | (13.45, 16.80) | 0.85 | < .001 |
|  | 9-15 years | 13.99 | (13.69, 14.30) | 0.16 |  |
|  | 16+ years | 11.42 | (10.75, 12.10) | 0.34 |  |
| Immigration status | Born in this country | 13.62 | (13.34, 13.90) | 0.14 | < .001 |
|  | Born in another country | 7.75 | (5.58, 9.93) | 1.07 |  |
| Religious affiliation | Christianity | 10.35 | (8.30, 12.40) | 1.02 | < .001 |
|  | Islam | 4.50 | * | * |  |
|  | Hinduism | 7.00 | * | * |  |
|  | Buddhism | 13.91 | (13.43, 14.38) | 0.24 |  |
|  | Judaism | 4.10 | * | * |  |
|  | Sikhism | 4.14 | * | * |  |
|  | Baha'i | 12.00 | * | * |  |
|  | Jainism | 4.34 | * | * |  |
|  | Shinto | 13.74 | (11.85, 15.63) | 0.95 |  |
|  | Confucianism | 8.03 | * | * |  |
|  | Primal, Animist, or Folk religion | 24.59 | * | * |  |
|  | Some other religion | 10.89 | * | * |  |
|  | No religion/Atheist/Agnostic | 13.55 | (13.20, 13.90) | 0.18 |  |

# Table S34a. Descriptive statistics for Kenya conditional on smokers

| **Characteristic** | **N = 713**^1^ |
| --- | --- |
| **Age group** |  |
| 18-24 | 128 (18%) |
| 25-34 | 183 (26%) |
| 35-44 | 152 (21%) |
| 45-54 | 120 (17%) |
| 55-64 | 71 (9.9%) |
| 65-74 | 56 (7.8%) |
| 75-84 | 3 (0.5%) |
| 85 or older | 0 (0%) |
| (Missing) | 0 (0%) |
| **Gender** |  |
| Male | 616 (86%) |
| Female | 94 (13%) |
| Other | 0 (0%) |
| (Missing) | 3 (0.4%) |
| **Marital status** |  |
| Married | 394 (55%) |
| Separated | 58 (8.2%) |
| Divorced | 16 (2.2%) |
| Widowed | 23 (3.2%) |
| Single, never married | 193 (27%) |
| Domestic Partner | 23 (3.3%) |
| (Missing) | 6 (0.8%) |
| **Employment** |  |
| Employed for an employer | 116 (16%) |
| Self-employed | 289 (41%) |
| Retired | 34 (4.7%) |
| Student | 50 (7.0%) |
| Homemaker | 19 (2.7%) |
| Unemployed and looking for a job | 183 (26%) |
| None of these/Other | 23 (3.2%) |
| (Missing) | 0 (0%) |
| **Religious service attendance** |  |
| More than 1/week | 104 (15%) |
| 1/week | 285 (40%) |
| 1-3/month | 108 (15%) |
| A few times a year | 123 (17%) |
| Never | 92 (13%) |
| (Missing) | 0 (0%) |
| **Education** |  |
| Up to 8 years | 305 (43%) |
| 9-15 years | 352 (49%) |
| 16+ years | 57 (7.9%) |
| (Missing) | 0 (0%) |
| **Immigration** |  |
| Born in this country | 707 (99%) |
| Born in another country | 6 (0.9%) |
| (Missing) | 0 (0%) |
| **Religious affiliation** |  |
| Christianity | 600 (84%) |
| Islam | 89 (13%) |
| Hinduism | 0 (0%) |
| Buddhism | 0 (0%) |
| Judaism | 0 (0%) |
| Sikhism | 0 (0%) |
| Baha'i | 0 (0%) |
| Jainism | 0 (0%) |
| Shinto | 0 (0%) |
| Taoism | 0 (0%) |
| Confucianism | 0 (0%) |
| Primal, Animist, or Folk religion | 3 (0.4%) |
| Spiritism | 0 (0%) |
| Umbanda, Candomble, and other African-derived religions | 0 (0%) |
| Chinese folk/traditional religion | 0 (0%) |
| Some other religion | 0 (0%) |
| No religion/Atheist/Agnostic | 20 (2.9%) |
| (Missing) | 0 (0%) |
| **Race/Ethnicity** |  |
| Embu | 9 (1.3%) |
| Kalenjin | 61 (8.6%) |
| Kamba | 121 (17%) |
| Kenyan Somali/Somali | 35 (5.0%) |
| Kikuyu | 198 (28%) |
| Kisii | 23 (3.2%) |
| Luhya | 69 (9.6%) |
| Luo | 46 (6.4%) |
| Maasai | 7 (1.0%) |
| Meru | 65 (9.2%) |
| Miji Kenda tribes | 47 (6.6%) |
| Other | 29 (4.1%) |
| (Missing) | 2 (0.3%) |
| ^1^n (%) | |

# Table S34b. Intensity by demographic category for Kenya conditional on smokers

| Variable | Category | Intensity | 95% CI | SE | Global p-value |
| --- | --- | --- | --- | --- | --- |
| Age group | 18-24 | 4.42 | (3.27, 5.58) | 0.58 | 0.023 |
|  | 25-34 | 4.81 | (3.53, 6.10) | 0.65 |  |
|  | 35-44 | 5.35 | (4.08, 6.62) | 0.64 |  |
|  | 45-54 | 6.19 | (4.65, 7.72) | 0.77 |  |
|  | 55-64 | 6.18 | (4.20, 8.16) | 0.99 |  |
|  | 65-74 | 4.56 | (3.16, 5.95) | 0.70 |  |
|  | 75-84 | 2.79 | * | * |  |
| Gender | Male | 5.14 | (4.61, 5.67) | 0.27 | 0.452 |
|  | Female | 6.51 | (2.89, 10.14) | 1.80 |  |
| Marital status | Married | 5.56 | (4.75, 6.37) | 0.41 | 0.088 |
|  | Separated | 4.95 | (3.49, 6.41) | 0.73 |  |
|  | Divorced | 5.70 | (3.86, 7.55) | 0.74 |  |
|  | Widowed | 6.75 | (3.32, 10.19) | 1.58 |  |
|  | Never | 4.12 | (3.36, 4.87) | 0.38 |  |
|  | Domestic Partner | 7.26 | (0.00, 15.42) | 3.60 |  |
| Employment | Employed for an employer | 5.69 | (3.70, 7.69) | 1.00 | 0.012 |
|  | Self-employed | 5.20 | (4.30, 6.10) | 0.46 |  |
|  | Retired | 4.24 | (2.22, 6.26) | 0.98 |  |
|  | Student | 3.35 | (2.23, 4.47) | 0.50 |  |
|  | Homemaker | 8.94 | (1.72, 16.16) | 3.10 |  |
|  | Unemployed and looking for a job | 4.67 | (3.85, 5.50) | 0.42 |  |
|  | None of these/Other | 8.77 | (3.97, 13.56) | 2.21 |  |
| Religious service attendance | More than 1/week | 4.48 | (3.25, 5.71) | 0.61 | 0.314 |
|  | 1/week | 4.76 | (3.82, 5.70) | 0.48 |  |
|  | 1-3/month | 5.89 | (4.22, 7.56) | 0.84 |  |
|  | A few times a year | 5.51 | (4.01, 7.01) | 0.76 |  |
|  | Never | 6.23 | (4.65, 7.81) | 0.79 |  |
| Education | Up to 8 years | 4.98 | (4.29, 5.67) | 0.35 | 0.613 |
|  | 9-15 years | 5.55 | (4.60, 6.50) | 0.48 |  |
|  | 16+ years | 5.34 | (2.87, 7.82) | 1.23 |  |
| Immigration status | Born in this country | 5.28 | (4.70, 5.86) | 0.30 | < .001 |
|  | Born in another country | 2.89 | (2.30, 3.48) | 0.13 |  |
| Religious affiliation | Christianity | 5.12 | (4.46, 5.78) | 0.34 | 0.406 |
|  | Islam | 5.60 | (4.08, 7.13) | 0.76 |  |
|  | Primal, Animist, or Folk religion | 4.47 | * | * |  |
|  | No religion/Atheist/Agnostic | 7.37 | (4.52, 10.22) | 1.33 |  |
| Race/Ethnicity | Luhya | 5.65 | (3.38, 7.91) | 1.13 | < .001 |
|  | Luo | 4.46 | (2.76, 6.16) | 0.81 |  |
|  | Kalenjin | 3.49 | (2.47, 4.52) | 0.49 |  |
|  | Kamba | 6.34 | (4.60, 8.08) | 0.88 |  |
|  | Kikuyu | 4.58 | (3.76, 5.40) | 0.41 |  |
|  | Kisii | 7.44 | (0.00, 16.54) | 3.81 |  |
|  | Maasai | 2.79 | * | * |  |
|  | Meru | 5.90 | (4.31, 7.48) | 0.79 |  |
|  | Kenyan Somali/Somali | 7.76 | (5.10, 10.42) | 1.25 |  |
|  | Miji Kenda tribes | 4.15 | (3.18, 5.12) | 0.47 |  |
|  | Embu | 5.86 | * | * |  |
|  | Other | 3.96 | (1.84, 6.08) | 0.96 |  |

# Table S35a. Descriptive statistics for Mexico conditional on smokers

| **Characteristic** | **N = 1,438**^1^ |
| --- | --- |
| **Age group** |  |
| 18-24 | 261 (18%) |
| 25-34 | 381 (26%) |
| 35-44 | 324 (23%) |
| 45-54 | 198 (14%) |
| 55-64 | 150 (10%) |
| 65-74 | 101 (7.0%) |
| 75-84 | 18 (1.3%) |
| 85 or older | 6 (0.4%) |
| (Missing) | 0 (0%) |
| **Gender** |  |
| Male | 953 (66%) |
| Female | 480 (33%) |
| Other | 1 (<0.1%) |
| (Missing) | 4 (0.3%) |
| **Marital status** |  |
| Married | 420 (29%) |
| Separated | 130 (9.0%) |
| Divorced | 70 (4.9%) |
| Widowed | 83 (5.8%) |
| Single, never married | 403 (28%) |
| Domestic Partner | 280 (19%) |
| (Missing) | 52 (3.6%) |
| **Employment** |  |
| Employed for an employer | 591 (41%) |
| Self-employed | 304 (21%) |
| Retired | 85 (5.9%) |
| Student | 50 (3.5%) |
| Homemaker | 154 (11%) |
| Unemployed and looking for a job | 157 (11%) |
| None of these/Other | 52 (3.6%) |
| (Missing) | 45 (3.2%) |
| **Religious service attendance** |  |
| More than 1/week | 83 (5.7%) |
| 1/week | 229 (16%) |
| 1-3/month | 160 (11%) |
| A few times a year | 617 (43%) |
| Never | 342 (24%) |
| (Missing) | 7 (0.5%) |
| **Education** |  |
| Up to 8 years | 307 (21%) |
| 9-15 years | 832 (58%) |
| 16+ years | 299 (21%) |
| (Missing) | 0 (0%) |
| **Immigration** |  |
| Born in this country | 1,352 (94%) |
| Born in another country | 39 (2.7%) |
| (Missing) | 47 (3.3%) |
| **Religious affiliation** |  |
| Christianity | 1,146 (80%) |
| Islam | 0 (0%) |
| Hinduism | 1 (<0.1%) |
| Buddhism | 1 (<0.1%) |
| Judaism | 3 (0.2%) |
| Sikhism | 0 (0%) |
| Baha'i | 0 (0%) |
| Jainism | 1 (<0.1%) |
| Shinto | 1 (<0.1%) |
| Taoism | 4 (0.3%) |
| Confucianism | 1 (<0.1%) |
| Primal, Animist, or Folk religion | 6 (0.4%) |
| Spiritism | 0 (0%) |
| Umbanda, Candomble, and other African-derived religions | 0 (0%) |
| Chinese folk/traditional religion | 0 (0%) |
| Some other religion | 17 (1.2%) |
| No religion/Atheist/Agnostic | 232 (16%) |
| (Missing) | 25 (1.7%) |
| **Race/Ethnicity** |  |
| Black | 28 (1.9%) |
| Indigenous | 109 (7.5%) |
| Mestizo | 722 (50%) |
| Mulatto | 27 (1.9%) |
| Other | 73 (5.1%) |
| White | 294 (20%) |
| (Missing) | 186 (13%) |
| ^1^n (%) | |

# Table S35b. Intensity by demographic category for Mexico conditional on smokers

| Variable | Category | Intensity | 95% CI | SE | Global p-value |
| --- | --- | --- | --- | --- | --- |
| Age group | 18-24 | 4.65 | (2.60, 6.71) | 1.04 | < .001 |
|  | 25-34 | 4.47 | (3.72, 5.21) | 0.38 |  |
|  | 35-44 | 5.20 | (4.05, 6.35) | 0.58 |  |
|  | 45-54 | 6.00 | (4.32, 7.67) | 0.85 |  |
|  | 55-64 | 5.75 | (4.54, 6.96) | 0.61 |  |
|  | 65-74 | 8.07 | (6.13, 10.02) | 0.98 |  |
|  | 75-84 | 9.05 | (3.99, 14.11) | 2.18 |  |
|  | 85 or older | 3.60 | (2.65, 4.55) | 0.46 |  |
| Gender | Male | 5.89 | (5.08, 6.70) | 0.41 | < .001 |
|  | Female | 4.06 | (3.58, 4.54) | 0.24 |  |
|  | Other | 7.83 | (0.00, 22.50) | 3.41 |  |
| Marital status | Married | 5.10 | (4.32, 5.87) | 0.39 | 0.060 |
|  | Separated | 7.66 | (5.17, 10.15) | 1.26 |  |
|  | Divorced | 7.14 | (3.34, 10.93) | 1.90 |  |
|  | Widowed | 5.52 | (4.19, 6.85) | 0.67 |  |
|  | Never | 5.11 | (3.69, 6.52) | 0.72 |  |
|  | Domestic Partner | 4.40 | (3.83, 4.97) | 0.29 |  |
| Employment | Employed for an employer | 4.83 | (4.11, 5.56) | 0.37 | < .001 |
|  | Self-employed | 5.78 | (4.62, 6.94) | 0.59 |  |
|  | Retired | 7.24 | (5.14, 9.34) | 1.05 |  |
|  | Student | 2.85 | (2.12, 3.58) | 0.36 |  |
|  | Homemaker | 4.42 | (3.43, 5.41) | 0.50 |  |
|  | Unemployed and looking for a job | 7.25 | (3.80, 10.70) | 1.75 |  |
|  | None of these/Other | 4.08 | (2.62, 5.54) | 0.71 |  |
| Religious service attendance | More than 1/week | 4.15 | (3.22, 5.08) | 0.47 | 0.093 |
|  | 1/week | 4.91 | (3.87, 5.94) | 0.52 |  |
|  | 1-3/month | 4.30 | (3.26, 5.33) | 0.52 |  |
|  | A few times a year | 5.22 | (4.42, 6.02) | 0.41 |  |
|  | Never | 6.54 | (4.75, 8.32) | 0.91 |  |
| Education | Up to 8 years | 6.46 | (4.49, 8.44) | 1.00 | 0.197 |
|  | 9-15 years | 5.11 | (4.49, 5.74) | 0.32 |  |
|  | 16+ years | 4.66 | (3.93, 5.38) | 0.37 |  |
| Immigration status | Born in this country | 5.19 | (4.61, 5.77) | 0.30 | 0.016 |
|  | Born in another country | 9.38 | (5.91, 12.86) | 1.71 |  |
| Religious affiliation | Christianity | 5.04 | (4.54, 5.53) | 0.25 | < .001 |
|  | Hinduism | 3.64 | * | * |  |
|  | Buddhism | 7.47 | * | * |  |
|  | Judaism | 3.27 | * | * |  |
|  | Jainism | 16.87 | * | * |  |
|  | Shinto | 5.00 | * | * |  |
|  | Taoism | 4.00 | * | * |  |
|  | Confucianism | 1.00 | * | * |  |
|  | Primal, Animist, or Folk religion | 6.11 | * | * |  |
|  | Some other religion | 5.04 | (2.03, 8.06) | 1.07 |  |
|  | No religion/Atheist/Agnostic | 6.68 | (4.18, 9.18) | 1.27 |  |
| Race/Ethnicity | Black | 4.77 | (2.79, 6.74) | 0.93 | 0.182 |
|  | Indigenous | 5.82 | (1.67, 9.97) | 2.09 |  |
|  | White | 6.00 | (4.94, 7.07) | 0.54 |  |
|  | Mestizo | 4.81 | (4.22, 5.41) | 0.30 |  |
|  | Mulatto | 6.25 | (0.00, 14.43) | 3.63 |  |
|  | Other | 6.45 | (3.99, 8.91) | 1.23 |  |

# Table S36a. Descriptive statistics for Nigeria conditional on smokers

| **Characteristic** | **N = 368**^1^ |
| --- | --- |
| **Age group** |  |
| 18-24 | 60 (16%) |
| 25-34 | 110 (30%) |
| 35-44 | 116 (32%) |
| 45-54 | 50 (14%) |
| 55-64 | 21 (5.8%) |
| 65-74 | 1 (0.4%) |
| 75-84 | 7 (1.8%) |
| 85 or older | 2 (0.5%) |
| (Missing) | 0 (0%) |
| **Gender** |  |
| Male | 310 (84%) |
| Female | 58 (16%) |
| Other | 0 (0%) |
| (Missing) | 0 (0%) |
| **Marital status** |  |
| Married | 220 (60%) |
| Separated | 13 (3.5%) |
| Divorced | 6 (1.6%) |
| Widowed | 5 (1.4%) |
| Single, never married | 118 (32%) |
| Domestic Partner | 3 (0.7%) |
| (Missing) | 3 (0.8%) |
| **Employment** |  |
| Employed for an employer | 46 (12%) |
| Self-employed | 201 (55%) |
| Retired | 15 (4.0%) |
| Student | 29 (7.8%) |
| Homemaker | 34 (9.3%) |
| Unemployed and looking for a job | 28 (7.7%) |
| None of these/Other | 15 (4.1%) |
| (Missing) | 0 (<0.1%) |
| **Religious service attendance** |  |
| More than 1/week | 203 (55%) |
| 1/week | 111 (30%) |
| 1-3/month | 28 (7.5%) |
| A few times a year | 16 (4.5%) |
| Never | 8 (2.1%) |
| (Missing) | 3 (0.8%) |
| **Education** |  |
| Up to 8 years | 152 (41%) |
| 9-15 years | 211 (57%) |
| 16+ years | 5 (1.5%) |
| (Missing) | 0 (0%) |
| **Immigration** |  |
| Born in this country | 367 (100%) |
| Born in another country | 1 (0.3%) |
| (Missing) | 0 (0%) |
| **Religious affiliation** |  |
| Christianity | 164 (45%) |
| Islam | 198 (54%) |
| Hinduism | 0 (0%) |
| Buddhism | 0 (0%) |
| Judaism | 0 (0%) |
| Sikhism | 0 (0%) |
| Baha'i | 0 (0%) |
| Jainism | 0 (0%) |
| Shinto | 0 (0%) |
| Taoism | 0 (0%) |
| Confucianism | 0 (0%) |
| Primal, Animist, or Folk religion | 6 (1.6%) |
| Spiritism | 0 (0%) |
| Umbanda, Candomble, and other African-derived religions | 0 (0%) |
| Chinese folk/traditional religion | 0 (0%) |
| Some other religion | 0 (0%) |
| No religion/Atheist/Agnostic | 0 (0%) |
| (Missing) | 0 (<0.1%) |
| **Race/Ethnicity** |  |
| Edo | 8 (2.1%) |
| Efik | 2 (0.6%) |
| Fulani | 15 (4.1%) |
| Hausa | 150 (41%) |
| Ibibio | 8 (2.1%) |
| Idoma | 5 (1.4%) |
| Igala | 3 (0.9%) |
| Igbo (Ibo) | 49 (13%) |
| Ijaw | 2 (0.5%) |
| Kanuri | 5 (1.4%) |
| Other | 53 (14%) |
| Tiv | 16 (4.2%) |
| Urhobo | 0 (0.1%) |
| Yoruba | 53 (14%) |
| (Missing) | 0 (0%) |
| ^1^n (%) | |

# Table S36b. Intensity by demographic category for Nigeria conditional on smokers

| Variable | Category | Intensity | 95% CI | SE | Global p-value |
| --- | --- | --- | --- | --- | --- |
| Age group | 18-24 | 4.75 | (2.34, 7.16) | 1.19 | < .001 |
|  | 25-34 | 6.07 | (4.05, 8.09) | 1.01 |  |
|  | 35-44 | 6.88 | (4.58, 9.19) | 1.16 |  |
|  | 45-54 | 5.04 | (0.73, 9.35) | 2.08 |  |
|  | 55-64 | 2.77 | (1.78, 3.76) | 0.42 |  |
|  | 65-74 | 10.00 | * | * |  |
|  | 75-84 | 2.34 | (0.00, 5.10) | 0.32 |  |
|  | 85 or older | 5.00 | * | * |  |
| Gender | Male | 5.39 | (4.28, 6.50) | 0.56 | 0.302 |
|  | Female | 8.91 | (1.93, 15.89) | 3.41 |  |
| Marital status | Married | 5.04 | (3.76, 6.32) | 0.65 | < .001 |
|  | Separated | 6.75 | (0.00, 14.78) | 2.82 |  |
|  | Divorced | 23.74 | * | * |  |
|  | Widowed | 15.17 | * | * |  |
|  | Never | 5.69 | (3.88, 7.50) | 0.91 |  |
|  | Domestic Partner | 2.11 | * | * |  |
| Employment | Employed for an employer | 5.08 | (3.24, 6.92) | 0.89 | 0.004 |
|  | Self-employed | 6.75 | (4.69, 8.80) | 1.04 |  |
|  | Retired | 2.65 | (1.65, 3.65) | 0.40 |  |
|  | Student | 3.39 | (2.13, 4.65) | 0.55 |  |
|  | Homemaker | 5.96 | (0.98, 10.94) | 2.36 |  |
|  | Unemployed and looking for a job | 3.91 | (2.65, 5.17) | 0.59 |  |
|  | None of these/Other | 4.29 | (2.50, 6.08) | 0.68 |  |
| Religious service attendance | More than 1/week | 5.25 | (3.75, 6.74) | 0.75 | 0.826 |
|  | 1/week | 5.86 | (3.45, 8.27) | 1.21 |  |
|  | 1-3/month | 5.94 | (1.78, 10.09) | 1.99 |  |
|  | A few times a year | 10.80 | (0.00, 22.26) | 4.88 |  |
|  | Never | 5.72 | (0.00, 27.60) | 2.73 |  |
| Education | Up to 8 years | 4.51 | (3.13, 5.88) | 0.70 | 0.159 |
|  | 9-15 years | 6.88 | (4.83, 8.93) | 1.04 |  |
|  | 16+ years | 5.42 | * | * |  |
| Immigration status | Born in this country | 5.72 | (4.47, 6.98) | 0.64 | < .001 |
|  | Born in another country | 20.00 | * | * |  |
| Religious affiliation | Christianity | 5.10 | (3.55, 6.66) | 0.79 | 0.238 |
|  | Islam | 6.34 | (4.47, 8.21) | 0.94 |  |
|  | Primal, Animist, or Folk religion | 3.44 | (0.00, 13.14) | 1.50 |  |
| Race/Ethnicity | Hausa | 6.15 | (4.17, 8.13) | 1.00 | < .001 |
|  | Yoruba | 7.75 | (1.82, 13.68) | 2.82 |  |
|  | Igbo (Ibo) | 5.44 | (2.01, 8.86) | 1.67 |  |
|  | Edo | 3.02 | * | * |  |
|  | Fulani | 2.57 | * | * |  |
|  | Kanuri | 9.48 | * | * |  |
|  | Tiv | 3.86 | * | * |  |
|  | Efik | 4.83 | * | * |  |
|  | Ijaw | 10.84 | * | * |  |
|  | Igala | 14.86 | * | * |  |
|  | Ibibio | 6.47 | * | * |  |
|  | Idoma | 3.61 | * | * |  |
|  | Other | 4.29 | (2.95, 5.63) | 0.66 |  |

# Table S37a. Descriptive statistics for Philippines conditional on smokers

| **Characteristic** | **N = 1,214**^1^ |
| --- | --- |
| **Age group** |  |
| 18-24 | 195 (16%) |
| 25-34 | 357 (29%) |
| 35-44 | 284 (23%) |
| 45-54 | 186 (15%) |
| 55-64 | 128 (11%) |
| 65-74 | 54 (4.4%) |
| 75-84 | 9 (0.7%) |
| 85 or older | 0 (0%) |
| (Missing) | 0 (0%) |
| **Gender** |  |
| Male | 1,011 (83%) |
| Female | 198 (16%) |
| Other | 1 (<0.1%) |
| (Missing) | 4 (0.3%) |
| **Marital status** |  |
| Married | 546 (45%) |
| Separated | 67 (5.5%) |
| Divorced | 2 (0.2%) |
| Widowed | 40 (3.3%) |
| Single, never married | 233 (19%) |
| Domestic Partner | 322 (27%) |
| (Missing) | 5 (0.4%) |
| **Employment** |  |
| Employed for an employer | 452 (37%) |
| Self-employed | 390 (32%) |
| Retired | 29 (2.4%) |
| Student | 64 (5.3%) |
| Homemaker | 96 (7.9%) |
| Unemployed and looking for a job | 162 (13%) |
| None of these/Other | 21 (1.7%) |
| (Missing) | 0 (0%) |
| **Religious service attendance** |  |
| More than 1/week | 178 (15%) |
| 1/week | 370 (31%) |
| 1-3/month | 336 (28%) |
| A few times a year | 262 (22%) |
| Never | 64 (5.3%) |
| (Missing) | 4 (0.3%) |
| **Education** |  |
| Up to 8 years | 330 (27%) |
| 9-15 years | 820 (68%) |
| 16+ years | 64 (5.3%) |
| (Missing) | 0 (0%) |
| **Immigration** |  |
| Born in this country | 1,211 (100%) |
| Born in another country | 3 (0.2%) |
| (Missing) | 0 (0%) |
| **Religious affiliation** |  |
| Christianity | 1,115 (92%) |
| Islam | 74 (6.1%) |
| Hinduism | 0 (0%) |
| Buddhism | 2 (0.2%) |
| Judaism | 3 (0.3%) |
| Sikhism | 0 (0%) |
| Baha'i | 0 (0%) |
| Jainism | 0 (0%) |
| Shinto | 0 (0%) |
| Taoism | 0 (0%) |
| Confucianism | 0 (0%) |
| Primal, Animist, or Folk religion | 1 (<0.1%) |
| Spiritism | 0 (0%) |
| Umbanda, Candomble, and other African-derived religions | 0 (0%) |
| Chinese folk/traditional religion | 0 (0%) |
| Some other religion | 9 (0.7%) |
| No religion/Atheist/Agnostic | 9 (0.8%) |
| (Missing) | 0 (<0.1%) |
| **Race/Ethnicity** |  |
| Aeta | 0 (0%) |
| Badjao | 0 (0%) |
| Bicolano/Bikolano | 70 (5.8%) |
| Cebuano | 153 (13%) |
| Chinese-Filipino | 0 (0%) |
| Igorot | 9 (0.8%) |
| Ilocano/Ilokano | 97 (8.0%) |
| Ilonggo/Hiligaynon | 109 (9.0%) |
| Kapampangan | 31 (2.6%) |
| Maguindanaoan | 14 (1.2%) |
| Mangyan | 0 (0%) |
| Maranao | 10 (0.8%) |
| Masbateno | 17 (1.4%) |
| Other | 43 (3.6%) |
| Pangasinense | 29 (2.4%) |
| Tagalog | 436 (36%) |
| Tausug | 25 (2.1%) |
| Visayan/Bisaya | 110 (9.1%) |
| Waray | 42 (3.5%) |
| Zamboangueno | 16 (1.3%) |
| (Missing) | 0 (0%) |
| ^1^n (%) | |

# Table S37b. Intensity by demographic category for Philippines conditional on smokers

| Variable | Category | Intensity | 95% CI | SE | Global p-value |
| --- | --- | --- | --- | --- | --- |
| Age group | 18-24 | 6.81 | (4.81, 8.81) | 1.01 | 0.019 |
|  | 25-34 | 7.04 | (6.13, 7.95) | 0.46 |  |
|  | 35-44 | 8.78 | (7.92, 9.65) | 0.44 |  |
|  | 45-54 | 8.98 | (7.82, 10.14) | 0.59 |  |
|  | 55-64 | 9.05 | (7.43, 10.68) | 0.82 |  |
|  | 65-74 | 8.18 | (5.30, 11.05) | 1.43 |  |
|  | 75-84 | 10.10 | * | * |  |
| Gender | Male | 8.48 | (7.86, 9.11) | 0.32 | < .001 |
|  | Female | 5.07 | (4.47, 5.66) | 0.30 |  |
|  | Other | 4.00 | * | * |  |
| Marital status | Married | 8.66 | (7.89, 9.43) | 0.39 | 0.023 |
|  | Separated | 7.51 | (5.81, 9.21) | 0.85 |  |
|  | Divorced | 6.39 | * | * |  |
|  | Widowed | 8.98 | (6.68, 11.28) | 1.12 |  |
|  | Never | 6.24 | (5.07, 7.40) | 0.59 |  |
|  | Domestic Partner | 8.17 | (7.05, 9.30) | 0.57 |  |
| Employment | Employed for an employer | 7.88 | (7.18, 8.58) | 0.36 | 0.002 |
|  | Self-employed | 8.91 | (7.85, 9.96) | 0.54 |  |
|  | Retired | 9.96 | (6.41, 13.52) | 1.70 |  |
|  | Student | 8.41 | (2.40, 14.41) | 2.96 |  |
|  | Homemaker | 5.32 | (3.94, 6.70) | 0.69 |  |
|  | Unemployed and looking for a job | 7.36 | (6.35, 8.38) | 0.51 |  |
|  | None of these/Other | 6.30 | (3.68, 8.91) | 1.18 |  |
| Religious service attendance | More than 1/week | 8.52 | (6.46, 10.59) | 1.05 | 0.038 |
|  | 1/week | 7.13 | (6.23, 8.04) | 0.46 |  |
|  | 1-3/month | 7.51 | (6.78, 8.25) | 0.37 |  |
|  | A few times a year | 9.50 | (8.25, 10.74) | 0.63 |  |
|  | Never | 8.44 | (6.28, 10.61) | 1.08 |  |
| Education | Up to 8 years | 8.48 | (7.54, 9.43) | 0.48 | 0.344 |
|  | 9-15 years | 7.92 | (7.21, 8.63) | 0.36 |  |
|  | 16+ years | 6.87 | (4.85, 8.88) | 1.01 |  |
| Immigration status | Born in this country | 8.02 | (7.48, 8.55) | 0.27 | 0.470 |
|  | Born in another country | 9.91 | * | * |  |
| Religious affiliation | Christianity | 7.86 | (7.35, 8.36) | 0.26 | < .001 |
|  | Islam | 10.16 | (6.12, 14.20) | 2.02 |  |
|  | Buddhism | 20.00 | * | * |  |
|  | Judaism | 1.00 | * | * |  |
|  | Primal, Animist, or Folk religion | 5.00 | * | * |  |
|  | Some other religion | 10.46 | * | * |  |
|  | No religion/Atheist/Agnostic | 7.44 | * | * |  |
| Race/Ethnicity | Tagalog | 8.06 | (7.21, 8.91) | 0.43 | 0.599 |
|  | Cebuano | 7.71 | (6.77, 8.65) | 0.48 |  |
|  | Ilocano/Ilokano | 7.76 | (6.04, 9.48) | 0.86 |  |
|  | Visayan/Bisaya | 7.58 | (5.84, 9.33) | 0.88 |  |
|  | Ilonggo/Hiligaynon | 7.41 | (5.35, 9.46) | 1.02 |  |
|  | Bicolano/Bikolano | 7.26 | (5.78, 8.74) | 0.74 |  |
|  | Waray | 8.09 | (6.49, 9.69) | 0.77 |  |
|  | Tausug | 11.40 | (0.00, 24.14) | 5.36 |  |
|  | Maranao | 7.92 | * | * |  |
|  | Maguindanaoan | 12.24 | (6.76, 17.72) | 1.95 |  |
|  | Kapampangan | 7.90 | (3.03, 12.77) | 2.24 |  |
|  | Pangasinense | 6.80 | (4.67, 8.94) | 0.95 |  |
|  | Zamboangueno | 10.55 | * | * |  |
|  | Masbateno | 11.70 | * | * |  |
|  | Igorot | 7.02 | * | * |  |
|  | Other | 8.33 | (6.00, 10.67) | 1.13 |  |

# Table S38a. Descriptive statistics for Poland conditional on smokers

| **Characteristic** | **N = 3,230**^1^ |
| --- | --- |
| **Age group** |  |
| 18-24 | 265 (8.2%) |
| 25-34 | 619 (19%) |
| 35-44 | 731 (23%) |
| 45-54 | 636 (20%) |
| 55-64 | 587 (18%) |
| 65-74 | 344 (11%) |
| 75-84 | 41 (1.3%) |
| 85 or older | 6 (0.2%) |
| (Missing) | 0 (0%) |
| **Gender** |  |
| Male | 2,024 (63%) |
| Female | 1,197 (37%) |
| Other | 3 (<0.1%) |
| (Missing) | 6 (0.2%) |
| **Marital status** |  |
| Married | 1,787 (55%) |
| Separated | 51 (1.6%) |
| Divorced | 245 (7.6%) |
| Widowed | 243 (7.5%) |
| Single, never married | 626 (19%) |
| Domestic Partner | 210 (6.5%) |
| (Missing) | 67 (2.1%) |
| **Employment** |  |
| Employed for an employer | 2,002 (62%) |
| Self-employed | 285 (8.8%) |
| Retired | 499 (15%) |
| Student | 102 (3.1%) |
| Homemaker | 107 (3.3%) |
| Unemployed and looking for a job | 145 (4.5%) |
| None of these/Other | 80 (2.5%) |
| (Missing) | 9 (0.3%) |
| **Religious service attendance** |  |
| More than 1/week | 37 (1.1%) |
| 1/week | 661 (20%) |
| 1-3/month | 697 (22%) |
| A few times a year | 1,163 (36%) |
| Never | 649 (20%) |
| (Missing) | 22 (0.7%) |
| **Education** |  |
| Up to 8 years | 443 (14%) |
| 9-15 years | 2,004 (62%) |
| 16+ years | 782 (24%) |
| (Missing) | 1 (<0.1%) |
| **Immigration** |  |
| Born in this country | 3,158 (98%) |
| Born in another country | 59 (1.8%) |
| (Missing) | 13 (0.4%) |
| **Religious affiliation** |  |
| Christianity | 2,842 (88%) |
| Islam | 0 (0%) |
| Hinduism | 0 (0%) |
| Buddhism | 0 (0%) |
| Judaism | 0 (0%) |
| Sikhism | 1 (<0.1%) |
| Baha'i | 0 (0%) |
| Jainism | 0 (0%) |
| Shinto | 1 (<0.1%) |
| Taoism | 0 (0%) |
| Confucianism | 0 (0%) |
| Primal, Animist, or Folk religion | 4 (0.1%) |
| Spiritism | 0 (0%) |
| Umbanda, Candomble, and other African-derived religions | 0 (0%) |
| Chinese folk/traditional religion | 0 (0%) |
| Some other religion | 0 (0%) |
| No religion/Atheist/Agnostic | 366 (11%) |
| (Missing) | 17 (0.5%) |
| **Race/Ethnicity** |  |
| Belarussian | 2 (<0.1%) |
| German | 0 (0%) |
| Kashubians | 1 (<0.1%) |
| Other | 1 (<0.1%) |
| Polish | 3,191 (99%) |
| Silesia | 5 (0.2%) |
| Ukrainian | 26 (0.8%) |
| (Missing) | 3 (0.1%) |
| ^1^n (%) | |

# Table S38b. Intensity by demographic category for Poland conditional on smokers

| Variable | Category | Intensity | 95% CI | SE | Global p-value |
| --- | --- | --- | --- | --- | --- |
| Age group | 18-24 | 9.23 | (8.12, 10.34) | 0.56 | < .001 |
|  | 25-34 | 11.25 | (10.59, 11.91) | 0.34 |  |
|  | 35-44 | 12.15 | (11.49, 12.80) | 0.33 |  |
|  | 45-54 | 12.02 | (11.15, 12.89) | 0.44 |  |
|  | 55-64 | 14.62 | (12.59, 16.65) | 1.03 |  |
|  | 65-74 | 12.07 | (10.62, 13.52) | 0.74 |  |
|  | 75-84 | 11.99 | (7.66, 16.32) | 2.12 |  |
|  | 85 or older | 11.87 | (6.24, 17.51) | 1.33 |  |
| Gender | Male | 13.05 | (12.46, 13.63) | 0.30 | < .001 |
|  | Female | 10.63 | (9.53, 11.73) | 0.56 |  |
|  | Other | 6.14 | * | * |  |
| Marital status | Married | 11.81 | (11.30, 12.32) | 0.26 | < .001 |
|  | Separated | 16.09 | (10.15, 22.03) | 2.95 |  |
|  | Divorced | 14.01 | (12.73, 15.30) | 0.65 |  |
|  | Widowed | 15.10 | (10.50, 19.70) | 2.33 |  |
|  | Never | 11.13 | (10.30, 11.96) | 0.42 |  |
|  | Domestic Partner | 11.42 | (10.13, 12.71) | 0.65 |  |
| Employment | Employed for an employer | 11.86 | (11.31, 12.40) | 0.28 | < .001 |
|  | Self-employed | 12.81 | (11.72, 13.90) | 0.55 |  |
|  | Retired | 13.22 | (10.77, 15.67) | 1.25 |  |
|  | Student | 8.01 | (7.09, 8.92) | 0.46 |  |
|  | Homemaker | 11.61 | (9.67, 13.54) | 0.97 |  |
|  | Unemployed and looking for a job | 14.30 | (12.91, 15.70) | 0.70 |  |
|  | None of these/Other | 12.64 | (9.99, 15.28) | 1.32 |  |
| Religious service attendance | More than 1/week | 13.33 | (9.86, 16.79) | 1.70 | 0.039 |
|  | 1/week | 11.74 | (9.88, 13.59) | 0.95 |  |
|  | 1-3/month | 11.32 | (10.58, 12.06) | 0.38 |  |
|  | A few times a year | 12.41 | (11.75, 13.07) | 0.34 |  |
|  | Never | 12.95 | (11.86, 14.04) | 0.56 |  |
| Education | Up to 8 years | 14.00 | (10.99, 17.01) | 1.53 | < .001 |
|  | 9-15 years | 12.29 | (11.77, 12.80) | 0.26 |  |
|  | 16+ years | 10.77 | (10.15, 11.40) | 0.32 |  |
| Immigration status | Born in this country | 12.17 | (11.58, 12.77) | 0.30 | 0.358 |
|  | Born in another country | 11.32 | (9.43, 13.20) | 0.94 |  |
| Religious affiliation | Christianity | 12.17 | (11.52, 12.81) | 0.33 | < .001 |
|  | Sikhism | 12.00 | * | * |  |
|  | Shinto | 20.00 | * | * |  |
|  | Primal, Animist, or Folk religion | 4.51 | (0.00, 14.14) | 1.09 |  |
|  | No religion/Atheist/Agnostic | 12.13 | (11.14, 13.12) | 0.50 |  |
| Race/Ethnicity | Polish | 12.14 | (11.55, 12.73) | 0.30 | < .001 |
|  | Belarussian | 11.23 | * | * |  |
|  | Ukrainian | 13.27 | (7.76, 18.77) | 2.62 |  |
|  | Silesia | 15.54 | * | * |  |
|  | Kashubians | 10.00 | * | * |  |
|  | Other | 16.67 | * | * |  |

# Table S39a. Descriptive statistics for South Africa conditional on smokers

| **Characteristic** | **N = 669**^1^ |
| --- | --- |
| **Age group** |  |
| 18-24 | 104 (16%) |
| 25-34 | 196 (29%) |
| 35-44 | 178 (27%) |
| 45-54 | 94 (14%) |
| 55-64 | 61 (9.2%) |
| 65-74 | 17 (2.6%) |
| 75-84 | 15 (2.2%) |
| 85 or older | 0 (0%) |
| (Missing) | 2 (0.3%) |
| **Gender** |  |
| Male | 497 (74%) |
| Female | 167 (25%) |
| Other | 2 (0.2%) |
| (Missing) | 3 (0.4%) |
| **Marital status** |  |
| Married | 141 (21%) |
| Separated | 37 (5.6%) |
| Divorced | 18 (2.6%) |
| Widowed | 14 (2.1%) |
| Single, never married | 355 (53%) |
| Domestic Partner | 84 (13%) |
| (Missing) | 20 (3.0%) |
| **Employment** |  |
| Employed for an employer | 160 (24%) |
| Self-employed | 119 (18%) |
| Retired | 47 (7.1%) |
| Student | 50 (7.5%) |
| Homemaker | 22 (3.3%) |
| Unemployed and looking for a job | 255 (38%) |
| None of these/Other | 14 (2.1%) |
| (Missing) | 1 (0.2%) |
| **Religious service attendance** |  |
| More than 1/week | 69 (10%) |
| 1/week | 186 (28%) |
| 1-3/month | 147 (22%) |
| A few times a year | 143 (21%) |
| Never | 122 (18%) |
| (Missing) | 2 (0.4%) |
| **Education** |  |
| Up to 8 years | 113 (17%) |
| 9-15 years | 511 (76%) |
| 16+ years | 45 (6.7%) |
| (Missing) | 0 (<0.1%) |
| **Immigration** |  |
| Born in this country | 648 (97%) |
| Born in another country | 20 (3.1%) |
| (Missing) | 0 (0%) |
| **Religious affiliation** |  |
| Christianity | 504 (75%) |
| Islam | 32 (4.7%) |
| Hinduism | 0 (0%) |
| Buddhism | 3 (0.4%) |
| Judaism | 0 (0%) |
| Sikhism | 0 (0%) |
| Baha'i | 0 (0%) |
| Jainism | 2 (0.3%) |
| Shinto | 0 (0%) |
| Taoism | 0 (0%) |
| Confucianism | 0 (0%) |
| Primal, Animist, or Folk religion | 27 (4.0%) |
| Spiritism | 0 (0%) |
| Umbanda, Candomble, and other African-derived religions | 0 (0%) |
| Chinese folk/traditional religion | 0 (0%) |
| Some other religion | 5 (0.7%) |
| No religion/Atheist/Agnostic | 90 (13%) |
| (Missing) | 7 (1.0%) |
| **Race/Ethnicity** |  |
| Asian/Indian | 0 (0%) |
| Black | 532 (80%) |
| Colored | 132 (20%) |
| Other | 0 (0%) |
| White | 4 (0.6%) |
| (Missing) | 0 (0%) |
| ^1^n (%) | |

# Table S39b. Intensity by demographic category for South Africa conditional on smokers

| Variable | Category | Intensity | 95% CI | SE | Global p-value |
| --- | --- | --- | --- | --- | --- |
| Age group | 18-24 | 5.59 | (4.63, 6.54) | 0.48 | < .001 |
|  | 25-34 | 5.84 | (5.11, 6.56) | 0.37 |  |
|  | 35-44 | 8.44 | (5.40, 11.49) | 1.54 |  |
|  | 45-54 | 8.01 | (6.31, 9.71) | 0.86 |  |
|  | 55-64 | 8.65 | (5.94, 11.35) | 1.35 |  |
|  | 65-74 | 5.43 | (2.04, 8.81) | 1.13 |  |
|  | 75-84 | 3.64 | (2.43, 4.85) | 0.48 |  |
| Gender | Male | 6.75 | (6.08, 7.42) | 0.34 | 0.053 |
|  | Female | 7.98 | (4.54, 11.41) | 1.74 |  |
|  | Other | 4.20 | (0.00, 11.90) | 1.02 |  |
| Marital status | Married | 6.99 | (5.57, 8.41) | 0.72 | 0.684 |
|  | Separated | 6.76 | (2.83, 10.69) | 1.92 |  |
|  | Divorced | 7.18 | (3.05, 11.30) | 1.75 |  |
|  | Widowed | 5.27 | (1.76, 8.77) | 1.42 |  |
|  | Never | 6.48 | (5.81, 7.16) | 0.34 |  |
|  | Domestic Partner | 9.71 | (3.73, 15.70) | 3.00 |  |
| Employment | Employed for an employer | 7.31 | (6.47, 8.14) | 0.42 | 0.111 |
|  | Self-employed | 7.38 | (5.88, 8.87) | 0.76 |  |
|  | Retired | 5.62 | (3.66, 7.59) | 0.94 |  |
|  | Student | 5.46 | (3.71, 7.21) | 0.84 |  |
|  | Homemaker | 6.41 | (3.71, 9.11) | 1.25 |  |
|  | Unemployed and looking for a job | 7.09 | (4.86, 9.32) | 1.13 |  |
|  | None of these/Other | 10.30 | (0.53, 20.07) | 3.83 |  |
| Religious service attendance | More than 1/week | 6.73 | (4.81, 8.64) | 0.91 | 0.035 |
|  | 1/week | 8.03 | (4.44, 11.62) | 1.82 |  |
|  | 1-3/month | 5.55 | (4.65, 6.45) | 0.46 |  |
|  | A few times a year | 6.47 | (5.53, 7.42) | 0.48 |  |
|  | Never | 8.13 | (6.68, 9.57) | 0.73 |  |
| Education | Up to 8 years | 10.37 | (5.28, 15.46) | 2.57 | 0.289 |
|  | 9-15 years | 6.31 | (5.74, 6.87) | 0.29 |  |
|  | 16+ years | 6.55 | (4.53, 8.56) | 0.99 |  |
| Immigration status | Born in this country | 7.03 | (5.89, 8.17) | 0.58 | 0.647 |
|  | Born in another country | 7.47 | (5.33, 9.62) | 1.00 |  |
| Religious affiliation | Christianity | 7.26 | (5.85, 8.67) | 0.72 | 0.003 |
|  | Islam | 8.58 | (5.83, 11.33) | 1.32 |  |
|  | Buddhism | 6.56 | * | * |  |
|  | Jainism | 5.00 | * | * |  |
|  | Primal, Animist, or Folk religion | 5.67 | (3.49, 7.86) | 1.04 |  |
|  | Some other religion | 8.55 | (3.60, 13.50) | 2.47 |  |
|  | No religion/Atheist/Agnostic | 5.71 | (4.47, 6.94) | 0.62 |  |
| Race/Ethnicity | Black | 6.12 | (5.57, 6.67) | 0.28 | 0.109 |
|  | White | 4.55 | * | * |  |
|  | Colored | 10.81 | (6.06, 15.55) | 2.40 |  |

# Table S40a. Descriptive statistics for Spain conditional on smokers

| **Characteristic** | **N = 2,082**^1^ |
| --- | --- |
| **Age group** |  |
| 18-24 | 165 (7.9%) |
| 25-34 | 323 (16%) |
| 35-44 | 491 (24%) |
| 45-54 | 481 (23%) |
| 55-64 | 358 (17%) |
| 65-74 | 230 (11%) |
| 75-84 | 32 (1.6%) |
| 85 or older | 2 (<0.1%) |
| (Missing) | 0 (0%) |
| **Gender** |  |
| Male | 1,128 (54%) |
| Female | 946 (45%) |
| Other | 3 (0.2%) |
| (Missing) | 4 (0.2%) |
| **Marital status** |  |
| Married | 909 (44%) |
| Separated | 99 (4.8%) |
| Divorced | 198 (9.5%) |
| Widowed | 64 (3.1%) |
| Single, never married | 588 (28%) |
| Domestic Partner | 204 (9.8%) |
| (Missing) | 20 (0.9%) |
| **Employment** |  |
| Employed for an employer | 991 (48%) |
| Self-employed | 210 (10%) |
| Retired | 383 (18%) |
| Student | 107 (5.1%) |
| Homemaker | 107 (5.1%) |
| Unemployed and looking for a job | 244 (12%) |
| None of these/Other | 38 (1.8%) |
| (Missing) | 3 (0.2%) |
| **Religious service attendance** |  |
| More than 1/week | 83 (4.0%) |
| 1/week | 260 (12%) |
| 1-3/month | 159 (7.7%) |
| A few times a year | 634 (30%) |
| Never | 940 (45%) |
| (Missing) | 5 (0.3%) |
| **Education** |  |
| Up to 8 years | 283 (14%) |
| 9-15 years | 1,416 (68%) |
| 16+ years | 383 (18%) |
| (Missing) | 0 (0%) |
| **Immigration** |  |
| Born in this country | 1,867 (90%) |
| Born in another country | 207 (9.9%) |
| (Missing) | 8 (0.4%) |
| **Religious affiliation** |  |
| Christianity | 1,356 (65%) |
| Islam | 45 (2.1%) |
| Hinduism | 3 (0.1%) |
| Buddhism | 13 (0.6%) |
| Judaism | 4 (0.2%) |
| Sikhism | 3 (0.1%) |
| Baha'i | 1 (<0.1%) |
| Jainism | 0 (0%) |
| Shinto | 0 (0%) |
| Taoism | 1 (<0.1%) |
| Confucianism | 3 (0.1%) |
| Primal, Animist, or Folk religion | 5 (0.2%) |
| Spiritism | 0 (0%) |
| Umbanda, Candomble, and other African-derived religions | 0 (0%) |
| Chinese folk/traditional religion | 0 (0%) |
| Some other religion | 13 (0.6%) |
| No religion/Atheist/Agnostic | 621 (30%) |
| (Missing) | 15 (0.7%) |
| ^1^n (%) | |

# Table S40b. Intensity by demographic category for Spain conditional on smokers

| Variable | Category | Intensity | 95% CI | SE | Global p-value |
| --- | --- | --- | --- | --- | --- |
| Age group | 18-24 | 6.94 | (6.07, 7.81) | 0.44 | < .001 |
|  | 25-34 | 8.92 | (8.04, 9.81) | 0.45 |  |
|  | 35-44 | 11.27 | (10.41, 12.12) | 0.44 |  |
|  | 45-54 | 12.11 | (11.35, 12.86) | 0.38 |  |
|  | 55-64 | 13.16 | (12.11, 14.21) | 0.54 |  |
|  | 65-74 | 12.27 | (10.11, 14.42) | 1.09 |  |
|  | 75-84 | 9.58 | (7.54, 11.62) | 0.98 |  |
|  | 85 or older | 9.25 | * | * |  |
| Gender | Male | 11.94 | (11.29, 12.60) | 0.33 | < .001 |
|  | Female | 10.22 | (9.67, 10.77) | 0.28 |  |
|  | Other | 10.24 | * | * |  |
| Marital status | Married | 11.53 | (10.88, 12.18) | 0.33 | 0.041 |
|  | Separated | 9.87 | (8.44, 11.29) | 0.72 |  |
|  | Divorced | 12.39 | (10.73, 14.06) | 0.84 |  |
|  | Widowed | 11.55 | (9.39, 13.71) | 1.08 |  |
|  | Never | 10.28 | (9.48, 11.08) | 0.41 |  |
|  | Domestic Partner | 11.35 | (9.71, 13.00) | 0.83 |  |
| Employment | Employed for an employer | 10.93 | (10.41, 11.44) | 0.26 | 0.001 |
|  | Self-employed | 12.50 | (11.23, 13.78) | 0.64 |  |
|  | Retired | 11.60 | (10.12, 13.08) | 0.75 |  |
|  | Student | 8.09 | (6.29, 9.89) | 0.91 |  |
|  | Homemaker | 10.15 | (8.14, 12.16) | 1.01 |  |
|  | Unemployed and looking for a job | 11.45 | (10.39, 12.51) | 0.54 |  |
|  | None of these/Other | 15.02 | (10.69, 19.35) | 2.11 |  |
| Religious service attendance | More than 1/week | 7.97 | (6.50, 9.44) | 0.74 | < .001 |
|  | 1/week | 9.40 | (8.21, 10.58) | 0.60 |  |
|  | 1-3/month | 11.83 | (9.60, 14.05) | 1.13 |  |
|  | A few times a year | 11.28 | (10.55, 12.01) | 0.37 |  |
|  | Never | 11.73 | (11.08, 12.37) | 0.33 |  |
| Education | Up to 8 years | 12.23 | (10.71, 13.75) | 0.77 | 0.076 |
|  | 9-15 years | 11.20 | (10.70, 11.70) | 0.26 |  |
|  | 16+ years | 10.23 | (9.22, 11.25) | 0.52 |  |
| Immigration status | Born in this country | 11.36 | (10.90, 11.82) | 0.24 | 0.002 |
|  | Born in another country | 9.28 | (8.01, 10.55) | 0.64 |  |
| Religious affiliation | Christianity | 11.40 | (10.84, 11.96) | 0.28 | < .001 |
|  | Islam | 8.21 | (6.06, 10.36) | 1.05 |  |
|  | Hinduism | 11.75 | * | * |  |
|  | Buddhism | 6.30 | * | * |  |
|  | Judaism | 6.82 | * | * |  |
|  | Sikhism | 14.47 | * | * |  |
|  | Baha'i | 15.00 | * | * |  |
|  | Taoism | 20.00 | * | * |  |
|  | Confucianism | 15.82 | * | * |  |
|  | Primal, Animist, or Folk religion | 6.06 | * | * |  |
|  | Some other religion | 12.29 | * | * |  |
|  | No religion/Atheist/Agnostic | 10.93 | (10.16, 11.69) | 0.39 |  |

# Table S41a. Descriptive statistics for Sweden conditional on smokers

| **Characteristic** | **N = 1,858**^1^ |
| --- | --- |
| **Age group** |  |
| 18-24 | 161 (8.7%) |
| 25-34 | 322 (17%) |
| 35-44 | 274 (15%) |
| 45-54 | 268 (14%) |
| 55-64 | 320 (17%) |
| 65-74 | 302 (16%) |
| 75-84 | 203 (11%) |
| 85 or older | 8 (0.5%) |
| (Missing) | 0 (0%) |
| **Gender** |  |
| Male | 858 (46%) |
| Female | 992 (53%) |
| Other | 4 (0.2%) |
| (Missing) | 3 (0.1%) |
| **Marital status** |  |
| Married | 668 (36%) |
| Separated | 73 (3.9%) |
| Divorced | 142 (7.6%) |
| Widowed | 65 (3.5%) |
| Single, never married | 504 (27%) |
| Domestic Partner | 394 (21%) |
| (Missing) | 12 (0.7%) |
| **Employment** |  |
| Employed for an employer | 901 (49%) |
| Self-employed | 181 (9.7%) |
| Retired | 507 (27%) |
| Student | 108 (5.8%) |
| Homemaker | 17 (0.9%) |
| Unemployed and looking for a job | 77 (4.1%) |
| None of these/Other | 63 (3.4%) |
| (Missing) | 2 (0.1%) |
| **Religious service attendance** |  |
| More than 1/week | 24 (1.3%) |
| 1/week | 69 (3.7%) |
| 1-3/month | 63 (3.4%) |
| A few times a year | 470 (25%) |
| Never | 1,225 (66%) |
| (Missing) | 8 (0.5%) |
| **Education** |  |
| Up to 8 years | 51 (2.7%) |
| 9-15 years | 1,510 (81%) |
| 16+ years | 297 (16%) |
| (Missing) | 0 (0%) |
| **Immigration** |  |
| Born in this country | 1,659 (89%) |
| Born in another country | 178 (9.6%) |
| (Missing) | 21 (1.1%) |
| **Religious affiliation** |  |
| Christianity | 1,001 (54%) |
| Islam | 117 (6.3%) |
| Hinduism | 4 (0.2%) |
| Buddhism | 18 (1.0%) |
| Judaism | 8 (0.4%) |
| Sikhism | 0 (0%) |
| Baha'i | 3 (0.2%) |
| Jainism | 0 (0%) |
| Shinto | 0 (<0.1%) |
| Taoism | 3 (0.1%) |
| Confucianism | 0 (0%) |
| Primal, Animist, or Folk religion | 14 (0.7%) |
| Spiritism | 0 (0%) |
| Umbanda, Candomble, and other African-derived religions | 0 (0%) |
| Chinese folk/traditional religion | 0 (0%) |
| Some other religion | 37 (2.0%) |
| No religion/Atheist/Agnostic | 644 (35%) |
| (Missing) | 9 (0.5%) |
| ^1^n (%) | |

# Table S41b. Intensity by demographic category for Sweden conditional on smokers

| Variable | Category | Intensity | 95% CI | SE | Global p-value |
| --- | --- | --- | --- | --- | --- |
| Age group | 18-24 | 5.67 | (4.39, 6.95) | 0.65 | < .001 |
|  | 25-34 | 7.27 | (6.35, 8.20) | 0.47 |  |
|  | 35-44 | 9.00 | (7.87, 10.14) | 0.58 |  |
|  | 45-54 | 9.85 | (8.92, 10.79) | 0.47 |  |
|  | 55-64 | 9.80 | (8.79, 10.80) | 0.51 |  |
|  | 65-74 | 10.59 | (9.23, 11.95) | 0.69 |  |
|  | 75-84 | 7.78 | (6.20, 9.36) | 0.78 |  |
|  | 85 or older | 10.78 | * | * |  |
| Gender | Male | 8.28 | (7.59, 8.97) | 0.35 | 0.099 |
|  | Female | 9.21 | (8.67, 9.76) | 0.28 |  |
|  | Other | 8.00 | * | * |  |
| Marital status | Married | 8.60 | (7.85, 9.34) | 0.38 | 0.016 |
|  | Separated | 9.60 | (6.98, 12.22) | 1.30 |  |
|  | Divorced | 11.31 | (9.69, 12.93) | 0.82 |  |
|  | Widowed | 7.75 | (6.27, 9.22) | 0.71 |  |
|  | Never | 8.41 | (7.55, 9.27) | 0.44 |  |
|  | Domestic Partner | 8.51 | (7.75, 9.27) | 0.39 |  |
| Employment | Employed for an employer | 8.29 | (7.76, 8.82) | 0.27 | < .001 |
|  | Self-employed | 8.65 | (7.05, 10.25) | 0.81 |  |
|  | Retired | 10.20 | (9.21, 11.20) | 0.51 |  |
|  | Student | 4.80 | (3.85, 5.76) | 0.48 |  |
|  | Homemaker | 12.77 | (3.80, 21.75) | 3.64 |  |
|  | Unemployed and looking for a job | 8.67 | (7.20, 10.14) | 0.74 |  |
|  | None of these/Other | 12.48 | (9.76, 15.20) | 1.34 |  |
| Religious service attendance | More than 1/week | 5.61 | (3.14, 8.08) | 1.16 | < .001 |
|  | 1/week | 6.45 | (5.29, 7.61) | 0.58 |  |
|  | 1-3/month | 6.31 | (4.67, 7.95) | 0.81 |  |
|  | A few times a year | 8.94 | (7.87, 10.00) | 0.54 |  |
|  | Never | 9.06 | (8.55, 9.56) | 0.26 |  |
| Education | Up to 8 years | 8.11 | (3.63, 12.60) | 2.16 | 0.470 |
|  | 9-15 years | 8.89 | (8.41, 9.37) | 0.24 |  |
|  | 16+ years | 8.20 | (7.12, 9.28) | 0.55 |  |
| Immigration status | Born in this country | 8.76 | (8.30, 9.22) | 0.23 | 0.743 |
|  | Born in another country | 8.89 | (7.43, 10.36) | 0.74 |  |
| Religious affiliation | Christianity | 9.04 | (8.39, 9.69) | 0.33 | < .001 |
|  | Islam | 9.26 | (7.17, 11.34) | 1.05 |  |
|  | Hinduism | 10.00 | * | * |  |
|  | Buddhism | 8.19 | (2.12, 14.26) | 1.07 |  |
|  | Judaism | 5.90 | * | * |  |
|  | Baha'i | 5.17 | * | * |  |
|  | Shinto | 2.00 | * | * |  |
|  | Taoism | 5.00 | * | * |  |
|  | Primal, Animist, or Folk religion | 12.29 | * | * |  |
|  | Some other religion | 5.28 | (3.26, 7.31) | 0.97 |  |
|  | No religion/Atheist/Agnostic | 8.54 | (7.95, 9.14) | 0.30 |  |

# Table S42a. Descriptive statistics for Tanzania conditional on smokers

| **Characteristic** | **N = 557**^1^ |
| --- | --- |
| **Age group** |  |
| 18-24 | 96 (17%) |
| 25-34 | 152 (27%) |
| 35-44 | 123 (22%) |
| 45-54 | 91 (16%) |
| 55-64 | 55 (9.9%) |
| 65-74 | 36 (6.5%) |
| 75-84 | 4 (0.7%) |
| 85 or older | 0 (0%) |
| (Missing) | 0 (0%) |
| **Gender** |  |
| Male | 432 (78%) |
| Female | 125 (22%) |
| Other | 0 (0%) |
| (Missing) | 0 (0%) |
| **Marital status** |  |
| Married | 362 (65%) |
| Separated | 38 (6.7%) |
| Divorced | 6 (1.1%) |
| Widowed | 18 (3.2%) |
| Single, never married | 118 (21%) |
| Domestic Partner | 15 (2.8%) |
| (Missing) | 0 (0%) |
| **Employment** |  |
| Employed for an employer | 33 (5.9%) |
| Self-employed | 351 (63%) |
| Retired | 7 (1.3%) |
| Student | 10 (1.8%) |
| Homemaker | 46 (8.3%) |
| Unemployed and looking for a job | 86 (16%) |
| None of these/Other | 23 (4.2%) |
| (Missing) | 0 (0%) |
| **Religious service attendance** |  |
| More than 1/week | 181 (32%) |
| 1/week | 211 (38%) |
| 1-3/month | 68 (12%) |
| A few times a year | 66 (12%) |
| Never | 31 (5.5%) |
| (Missing) | 0 (0%) |
| **Education** |  |
| Up to 8 years | 443 (80%) |
| 9-15 years | 104 (19%) |
| 16+ years | 10 (1.8%) |
| (Missing) | 0 (0%) |
| **Immigration** |  |
| Born in this country | 555 (100%) |
| Born in another country | 1 (<0.1%) |
| (Missing) | 1 (0.2%) |
| **Religious affiliation** |  |
| Christianity | 275 (49%) |
| Islam | 266 (48%) |
| Hinduism | 0 (0%) |
| Buddhism | 0 (0%) |
| Judaism | 0 (0%) |
| Sikhism | 0 (0%) |
| Baha'i | 0 (0%) |
| Jainism | 0 (0%) |
| Shinto | 0 (0%) |
| Taoism | 0 (0%) |
| Confucianism | 0 (0%) |
| Primal, Animist, or Folk religion | 0 (0%) |
| Spiritism | 0 (0%) |
| Umbanda, Candomble, and other African-derived religions | 0 (0%) |
| Chinese folk/traditional religion | 0 (0%) |
| Some other religion | 0 (0%) |
| No religion/Atheist/Agnostic | 15 (2.7%) |
| (Missing) | 1 (0.2%) |
| **Race/Ethnicity** |  |
| African | 557 (100%) |
| Arab | 0 (0%) |
| Indian | 0 (0%) |
| (Missing) | 0 (0%) |
| ^1^n (%) | |

# Table S42b. Intensity by demographic category for Tanzania conditional on smokers

| Variable | Category | Intensity | 95% CI | SE | Global p-value |
| --- | --- | --- | --- | --- | --- |
| Age group | 18-24 | 4.61 | (2.88, 6.35) | 0.85 | 0.677 |
|  | 25-34 | 5.43 | (4.41, 6.45) | 0.51 |  |
|  | 35-44 | 5.85 | (4.59, 7.10) | 0.63 |  |
|  | 45-54 | 5.16 | (4.31, 6.02) | 0.43 |  |
|  | 55-64 | 7.75 | (2.47, 13.03) | 2.60 |  |
|  | 65-74 | 5.06 | (3.13, 6.98) | 0.86 |  |
|  | 75-84 | 10.76 | * | * |  |
| Gender | Male | 5.40 | (4.87, 5.92) | 0.27 | 0.316 |
|  | Female | 11.24 | (0.00, 23.78) | 5.91 |  |
| Marital status | Married | 5.66 | (4.54, 6.77) | 0.57 | 0.005 |
|  | Separated | 6.86 | (4.89, 8.84) | 0.95 |  |
|  | Divorced | 6.96 | (0.00, 23.89) | 2.39 |  |
|  | Widowed | 4.46 | * | * |  |
|  | Never | 5.87 | (3.92, 7.82) | 0.97 |  |
|  | Domestic Partner | 3.67 | (2.60, 4.73) | 0.41 |  |
| Employment | Employed for an employer | 5.09 | (2.45, 7.73) | 1.16 | < .001 |
|  | Self-employed | 6.09 | (5.00, 7.18) | 0.55 |  |
|  | Retired | 5.22 | (0.00, 12.69) | 0.63 |  |
|  | Student | 1.16 | * | * |  |
|  | Homemaker | 5.14 | * | * |  |
|  | Unemployed and looking for a job | 5.15 | (3.72, 6.58) | 0.71 |  |
|  | None of these/Other | 3.62 | (2.33, 4.91) | 0.54 |  |
| Religious service attendance | More than 1/week | 7.26 | (4.20, 10.32) | 1.54 | 0.339 |
|  | 1/week | 4.91 | (4.17, 5.65) | 0.37 |  |
|  | 1-3/month | 5.12 | (4.23, 6.01) | 0.45 |  |
|  | A few times a year | 5.77 | (4.30, 7.23) | 0.73 |  |
|  | Never | 6.50 | (3.75, 9.26) | 1.31 |  |
| Education | Up to 8 years | 5.59 | (4.62, 6.55) | 0.49 | 0.047 |
|  | 9-15 years | 6.50 | (5.00, 8.00) | 0.75 |  |
|  | 16+ years | 3.72 | * | * |  |
| Immigration status | Born in this country | 5.72 | (4.90, 6.55) | 0.42 | < .001 |
|  | Born in another country | 3.00 | * | * |  |
| Religious affiliation | Christianity | 4.97 | (4.33, 5.61) | 0.32 | < .001 |
|  | Islam | 6.66 | (5.09, 8.23) | 0.80 |  |
|  | No religion/Atheist/Agnostic | 2.73 | (1.66, 3.81) | 0.44 |  |
| Race/Ethnicity | African | 5.72 | (4.89, 6.54) | 0.42 | * |

# Table S43a. Descriptive statistics for Türkiye conditional on smokers

| **Characteristic** | **N = 789**^1^ |
| --- | --- |
| **Age group** |  |
| 18-24 | 116 (15%) |
| 25-34 | 181 (23%) |
| 35-44 | 176 (22%) |
| 45-54 | 151 (19%) |
| 55-64 | 106 (13%) |
| 65-74 | 48 (6.0%) |
| 75-84 | 8 (1.1%) |
| 85 or older | 3 (0.4%) |
| (Missing) | 0 (0%) |
| **Gender** |  |
| Male | 478 (61%) |
| Female | 311 (39%) |
| Other | 0 (0%) |
| (Missing) | 0 (0%) |
| **Marital status** |  |
| Married | 475 (60%) |
| Separated | 7 (0.9%) |
| Divorced | 46 (5.8%) |
| Widowed | 20 (2.5%) |
| Single, never married | 233 (29%) |
| Domestic Partner | 0 (0%) |
| (Missing) | 9 (1.2%) |
| **Employment** |  |
| Employed for an employer | 251 (32%) |
| Self-employed | 179 (23%) |
| Retired | 97 (12%) |
| Student | 44 (5.6%) |
| Homemaker | 127 (16%) |
| Unemployed and looking for a job | 59 (7.5%) |
| None of these/Other | 32 (4.1%) |
| (Missing) | 0 (0%) |
| **Religious service attendance** |  |
| More than 1/week | 231 (29%) |
| 1/week | 153 (19%) |
| 1-3/month | 93 (12%) |
| A few times a year | 159 (20%) |
| Never | 150 (19%) |
| (Missing) | 2 (0.3%) |
| **Education** |  |
| Up to 8 years | 219 (28%) |
| 9-15 years | 401 (51%) |
| 16+ years | 170 (22%) |
| (Missing) | 0 (0%) |
| **Immigration** |  |
| Born in this country | 761 (96%) |
| Born in another country | 28 (3.6%) |
| (Missing) | 0 (0%) |
| **Religious affiliation** |  |
| Christianity | 1 (0.2%) |
| Islam | 733 (93%) |
| Hinduism | 0 (0%) |
| Buddhism | 0 (0%) |
| Judaism | 1 (0.1%) |
| Sikhism | 0 (0%) |
| Baha'i | 0 (0%) |
| Jainism | 0 (0%) |
| Shinto | 0 (0%) |
| Taoism | 0 (0%) |
| Confucianism | 0 (0%) |
| Primal, Animist, or Folk religion | 1 (<0.1%) |
| Spiritism | 0 (0%) |
| Umbanda, Candomble, and other African-derived religions | 0 (0%) |
| Chinese folk/traditional religion | 0 (0%) |
| Some other religion | 1 (0.1%) |
| No religion/Atheist/Agnostic | 44 (5.5%) |
| (Missing) | 8 (1.1%) |
| **Race/Ethnicity** |  |
| Albanian | 7 (0.8%) |
| Arab | 19 (2.4%) |
| Armenian | 0 (0%) |
| Azeri | 8 (1.0%) |
| Bosnian | 3 (0.4%) |
| Circassian | 16 (2.0%) |
| Georgian | 4 (0.5%) |
| Greek | 1 (0.1%) |
| Kurdish/Zaza | 146 (18%) |
| Laz | 11 (1.4%) |
| Other | 30 (3.7%) |
| Turkish | 540 (68%) |
| Uyghur | 1 (0.2%) |
| (Missing) | 3 (0.4%) |
| ^1^n (%) | |

# Table S43b. Intensity by demographic category for Türkiye conditional on smokers

| Variable | Category | Intensity | 95% CI | SE | Global p-value |
| --- | --- | --- | --- | --- | --- |
| Age group | 18-24 | 16.92 | (14.50, 19.34) | 1.22 | < .001 |
|  | 25-34 | 18.67 | (16.61, 20.73) | 1.04 |  |
|  | 35-44 | 18.70 | (16.80, 20.61) | 0.96 |  |
|  | 45-54 | 19.49 | (16.69, 22.29) | 1.41 |  |
|  | 55-64 | 17.87 | (14.78, 20.97) | 1.56 |  |
|  | 65-74 | 18.61 | (11.48, 25.75) | 3.52 |  |
|  | 75-84 | 12.02 | * | * |  |
|  | 85 or older | 20.00 | * | * |  |
| Gender | Male | 21.60 | (20.15, 23.04) | 0.74 | < .001 |
|  | Female | 13.48 | (12.14, 14.81) | 0.68 |  |
| Marital status | Married | 17.36 | (15.95, 18.76) | 0.71 | 0.032 |
|  | Separated | 15.49 | * | * |  |
|  | Divorced | 25.01 | (19.36, 30.65) | 2.79 |  |
|  | Widowed | 19.60 | (9.71, 29.49) | 4.55 |  |
|  | Never | 19.22 | (17.53, 20.92) | 0.86 |  |
| Employment | Employed for an employer | 17.86 | (16.19, 19.54) | 0.85 | < .001 |
|  | Self-employed | 21.90 | (19.58, 24.21) | 1.17 |  |
|  | Retired | 18.59 | (15.13, 22.06) | 1.74 |  |
|  | Student | 13.55 | (11.02, 16.09) | 1.25 |  |
|  | Homemaker | 13.87 | (11.16, 16.58) | 1.37 |  |
|  | Unemployed and looking for a job | 19.16 | (15.67, 22.64) | 1.73 |  |
|  | None of these/Other | 25.60 | (18.69, 32.51) | 3.34 |  |
| Religious service attendance | More than 1/week | 18.49 | (16.31, 20.67) | 1.11 | 0.336 |
|  | 1/week | 17.32 | (14.97, 19.67) | 1.19 |  |
|  | 1-3/month | 16.31 | (13.37, 19.26) | 1.48 |  |
|  | A few times a year | 19.60 | (17.18, 22.01) | 1.22 |  |
|  | Never | 19.39 | (17.19, 21.58) | 1.11 |  |
| Education | Up to 8 years | 19.35 | (16.67, 22.02) | 1.36 | 0.664 |
|  | 9-15 years | 17.98 | (16.60, 19.37) | 0.70 |  |
|  | 16+ years | 18.16 | (16.52, 19.79) | 0.83 |  |
| Immigration status | Born in this country | 18.25 | (17.17, 19.33) | 0.55 | 0.289 |
|  | Born in another country | 22.45 | (14.36, 30.55) | 3.92 |  |
| Religious affiliation | Christianity | 7.08 | * | * | < .001 |
|  | Islam | 18.24 | (17.10, 19.37) | 0.58 |  |
|  | Judaism | 20.00 | * | * |  |
|  | Primal, Animist, or Folk religion | 40.00 | * | * |  |
|  | Some other religion | 25.13 | * | * |  |
|  | No religion/Atheist/Agnostic | 20.83 | (17.69, 23.98) | 1.55 |  |
| Race/Ethnicity | Arab | 19.75 | (11.78, 27.72) | 3.28 | < .001 |
|  | Turkish | 17.82 | (16.53, 19.10) | 0.65 |  |
|  | Greek | 12.84 | * | * |  |
|  | Kurdish/Zaza | 20.97 | (18.32, 23.61) | 1.34 |  |
|  | Laz | 16.02 | * | * |  |
|  | Circassian | 16.83 | (1.20, 32.45) | 3.67 |  |
|  | Bosnian | 9.27 | * | * |  |
|  | Georgian | 11.04 | * | * |  |
|  | Uyghur | 4.00 | * | * |  |
|  | Albanian | 28.45 | * | * |  |
|  | Azeri | 11.12 | * | * |  |
|  | Other | 19.60 | (12.78, 26.42) | 3.21 |  |

# Table S44a. Descriptive statistics for United Kingdom conditional on smokers

| **Characteristic** | **N = 928**^1^ |
| --- | --- |
| **Age group** |  |
| 18-24 | 38 (4.0%) |
| 25-34 | 220 (24%) |
| 35-44 | 190 (20%) |
| 45-54 | 157 (17%) |
| 55-64 | 148 (16%) |
| 65-74 | 133 (14%) |
| 75-84 | 39 (4.2%) |
| 85 or older | 1 (0.1%) |
| (Missing) | 0 (0%) |
| **Gender** |  |
| Male | 525 (57%) |
| Female | 398 (43%) |
| Other | 1 (0.1%) |
| (Missing) | 3 (0.4%) |
| **Marital status** |  |
| Married | 432 (47%) |
| Separated | 13 (1.4%) |
| Divorced | 85 (9.2%) |
| Widowed | 43 (4.6%) |
| Single, never married | 231 (25%) |
| Domestic Partner | 111 (12%) |
| (Missing) | 11 (1.2%) |
| **Employment** |  |
| Employed for an employer | 527 (57%) |
| Self-employed | 97 (10%) |
| Retired | 161 (17%) |
| Student | 13 (1.4%) |
| Homemaker | 37 (4.0%) |
| Unemployed and looking for a job | 40 (4.3%) |
| None of these/Other | 47 (5.1%) |
| (Missing) | 5 (0.5%) |
| **Religious service attendance** |  |
| More than 1/week | 66 (7.1%) |
| 1/week | 146 (16%) |
| 1-3/month | 61 (6.6%) |
| A few times a year | 132 (14%) |
| Never | 522 (56%) |
| (Missing) | 1 (<0.1%) |
| **Education** |  |
| Up to 8 years | 214 (23%) |
| 9-15 years | 378 (41%) |
| 16+ years | 334 (36%) |
| (Missing) | 1 (<0.1%) |
| **Immigration** |  |
| Born in this country | 815 (88%) |
| Born in another country | 109 (12%) |
| (Missing) | 4 (0.5%) |
| **Religious affiliation** |  |
| Christianity | 488 (53%) |
| Islam | 34 (3.7%) |
| Hinduism | 6 (0.6%) |
| Buddhism | 12 (1.3%) |
| Judaism | 2 (0.2%) |
| Sikhism | 3 (0.4%) |
| Baha'i | 5 (0.5%) |
| Jainism | 4 (0.5%) |
| Shinto | 0 (0%) |
| Taoism | 1 (<0.1%) |
| Confucianism | 0 (0%) |
| Primal, Animist, or Folk religion | 12 (1.3%) |
| Spiritism | 0 (0%) |
| Umbanda, Candomble, and other African-derived religions | 0 (0%) |
| Chinese folk/traditional religion | 0 (0%) |
| Some other religion | 14 (1.5%) |
| No religion/Atheist/Agnostic | 342 (37%) |
| (Missing) | 5 (0.5%) |
| **Race/Ethnicity** |  |
| Asian | 61 (6.6%) |
| Black | 25 (2.7%) |
| Other | 22 (2.3%) |
| White | 814 (88%) |
| (Missing) | 6 (0.7%) |
| ^1^n (%) | |

# Table S44b. Intensity by demographic category for United Kingdom conditional on smokers

| Variable | Category | Intensity | 95% CI | SE | Global p-value |
| --- | --- | --- | --- | --- | --- |
| Age group | 18-24 | 6.09 | (3.53, 8.65) | 1.25 | < .001 |
|  | 25-34 | 7.07 | (6.16, 7.99) | 0.46 |  |
|  | 35-44 | 10.99 | (9.24, 12.75) | 0.89 |  |
|  | 45-54 | 11.70 | (9.94, 13.46) | 0.89 |  |
|  | 55-64 | 13.78 | (12.21, 15.35) | 0.79 |  |
|  | 65-74 | 13.53 | (11.74, 15.32) | 0.90 |  |
|  | 75-84 | 11.25 | (6.63, 15.86) | 2.17 |  |
|  | 85 or older | 11.46 | * | * |  |
| Gender | Male | 10.03 | (9.10, 10.96) | 0.47 | < .001 |
|  | Female | 11.67 | (10.64, 12.70) | 0.52 |  |
|  | Other | 18.04 | (8.95, 27.13) | 1.99 |  |
| Marital status | Married | 9.67 | (8.80, 10.54) | 0.44 | < .001 |
|  | Separated | 9.54 | (3.23, 15.85) | 2.27 |  |
|  | Divorced | 13.20 | (11.16, 15.24) | 1.02 |  |
|  | Widowed | 16.48 | (12.58, 20.38) | 1.92 |  |
|  | Never | 10.09 | (8.70, 11.49) | 0.71 |  |
|  | Domestic Partner | 12.23 | (9.79, 14.67) | 1.23 |  |
| Employment | Employed for an employer | 9.45 | (8.61, 10.28) | 0.42 | < .001 |
|  | Self-employed | 9.36 | (7.50, 11.22) | 0.94 |  |
|  | Retired | 12.70 | (11.10, 14.30) | 0.81 |  |
|  | Student | 9.11 | (1.04, 17.19) | 2.80 |  |
|  | Homemaker | 14.51 | (8.96, 20.06) | 2.71 |  |
|  | Unemployed and looking for a job | 13.77 | (11.00, 16.54) | 1.35 |  |
|  | None of these/Other | 17.08 | (13.53, 20.63) | 1.75 |  |
| Religious service attendance | More than 1/week | 6.68 | (5.08, 8.29) | 0.80 | < .001 |
|  | 1/week | 8.06 | (6.42, 9.70) | 0.83 |  |
|  | 1-3/month | 8.61 | (6.95, 10.28) | 0.83 |  |
|  | A few times a year | 10.95 | (9.32, 12.58) | 0.82 |  |
|  | Never | 12.22 | (11.24, 13.20) | 0.50 |  |
| Education | Up to 8 years | 13.61 | (11.56, 15.66) | 1.04 | < .001 |
|  | 9-15 years | 11.30 | (10.29, 12.30) | 0.51 |  |
|  | 16+ years | 8.40 | (7.69, 9.12) | 0.36 |  |
| Immigration status | Born in this country | 11.07 | (10.32, 11.82) | 0.38 | < .001 |
|  | Born in another country | 8.17 | (6.67, 9.67) | 0.76 |  |
| Religious affiliation | Christianity | 10.70 | (9.78, 11.62) | 0.47 | < .001 |
|  | Islam | 5.37 | (3.08, 7.67) | 1.09 |  |
|  | Hinduism | 4.92 | * | * |  |
|  | Buddhism | 7.05 | * | * |  |
|  | Judaism | 11.48 | * | * |  |
|  | Sikhism | 5.17 | * | * |  |
|  | Baha'i | 27.60 | * | * |  |
|  | Jainism | 5.00 | * | * |  |
|  | Taoism | 2.02 | * | * |  |
|  | Primal, Animist, or Folk religion | 4.43 | * | * |  |
|  | Some other religion | 12.80 | * | * |  |
|  | No religion/Atheist/Agnostic | 11.54 | (10.51, 12.58) | 0.53 |  |
| Race/Ethnicity | Asian | 6.65 | (4.67, 8.63) | 0.99 | < .001 |
|  | Black | 5.62 | (3.09, 8.15) | 1.20 |  |
|  | White | 11.33 | (10.59, 12.07) | 0.38 |  |
|  | Other | 6.31 | (2.98, 9.64) | 1.55 |  |

# Table S45a. Descriptive statistics for United States conditional on smokers

| **Characteristic** | **N = 4,176**^1^ |
| --- | --- |
| **Age group** |  |
| 18-24 | 175 (4.2%) |
| 25-34 | 895 (21%) |
| 35-44 | 771 (18%) |
| 45-54 | 667 (16%) |
| 55-64 | 989 (24%) |
| 65-74 | 525 (13%) |
| 75-84 | 124 (3.0%) |
| 85 or older | 30 (0.7%) |
| (Missing) | 0 (0%) |
| **Gender** |  |
| Male | 1,977 (47%) |
| Female | 2,153 (52%) |
| Other | 33 (0.8%) |
| (Missing) | 13 (0.3%) |
| **Marital status** |  |
| Married | 1,657 (40%) |
| Separated | 172 (4.1%) |
| Divorced | 540 (13%) |
| Widowed | 218 (5.2%) |
| Single, never married | 1,196 (29%) |
| Domestic Partner | 379 (9.1%) |
| (Missing) | 14 (0.3%) |
| **Employment** |  |
| Employed for an employer | 1,972 (47%) |
| Self-employed | 475 (11%) |
| Retired | 814 (20%) |
| Student | 85 (2.0%) |
| Homemaker | 241 (5.8%) |
| Unemployed and looking for a job | 277 (6.6%) |
| None of these/Other | 304 (7.3%) |
| (Missing) | 8 (0.2%) |
| **Religious service attendance** |  |
| More than 1/week | 156 (3.7%) |
| 1/week | 348 (8.3%) |
| 1-3/month | 270 (6.5%) |
| A few times a year | 1,004 (24%) |
| Never | 2,379 (57%) |
| (Missing) | 18 (0.4%) |
| **Education** |  |
| Up to 8 years | 36 (0.9%) |
| 9-15 years | 3,623 (87%) |
| 16+ years | 508 (12%) |
| (Missing) | 8 (0.2%) |
| **Immigration** |  |
| Born in this country | 3,975 (95%) |
| Born in another country | 107 (2.6%) |
| (Missing) | 94 (2.3%) |
| **Religious affiliation** |  |
| Christianity | 2,374 (57%) |
| Islam | 22 (0.5%) |
| Hinduism | 5 (0.1%) |
| Buddhism | 35 (0.8%) |
| Judaism | 31 (0.7%) |
| Sikhism | 0 (0%) |
| Baha'i | 3 (<0.1%) |
| Jainism | 0 (0%) |
| Shinto | 0 (0%) |
| Taoism | 52 (1.2%) |
| Confucianism | 0 (0%) |
| Primal, Animist, or Folk religion | 31 (0.7%) |
| Spiritism | 0 (0%) |
| Umbanda, Candomble, and other African-derived religions | 0 (0%) |
| Chinese folk/traditional religion | 0 (0%) |
| Some other religion | 234 (5.6%) |
| No religion/Atheist/Agnostic | 1,295 (31%) |
| (Missing) | 93 (2.2%) |
| **Race/Ethnicity** |  |
| Asian | 111 (2.7%) |
| Black | 655 (16%) |
| Hispanic | 757 (18%) |
| Other | 150 (3.6%) |
| White | 2,502 (60%) |
| (Missing) | 2 (<0.1%) |
| ^1^n (%) | |

# Table S45b. Intensity by demographic category for United States conditional on smokers

| Variable | Category | Intensity | 95% CI | SE | Global p-value |
| --- | --- | --- | --- | --- | --- |
| Age group | 18-24 | 6.74 | (3.17, 10.31) | 1.81 | < .001 |
|  | 25-34 | 10.94 | (8.47, 13.42) | 1.26 |  |
|  | 35-44 | 10.57 | (9.21, 11.93) | 0.69 |  |
|  | 45-54 | 13.13 | (11.80, 14.46) | 0.68 |  |
|  | 55-64 | 13.29 | (12.35, 14.24) | 0.48 |  |
|  | 65-74 | 14.34 | (13.09, 15.60) | 0.64 |  |
|  | 75-84 | 12.27 | (10.98, 13.56) | 0.65 |  |
|  | 85 or older | 13.41 | * | * |  |
| Gender | Male | 12.71 | (11.74, 13.69) | 0.50 | 0.003 |
|  | Female | 11.54 | (10.50, 12.59) | 0.53 |  |
|  | Other | 5.55 | (0.90, 10.20) | 2.27 |  |
| Marital status | Married | 12.82 | (11.85, 13.79) | 0.49 | 0.097 |
|  | Separated | 10.34 | (6.26, 14.42) | 2.07 |  |
|  | Divorced | 12.58 | (11.20, 13.96) | 0.70 |  |
|  | Widowed | 13.63 | (12.31, 14.94) | 0.67 |  |
|  | Never | 10.47 | (8.64, 12.30) | 0.93 |  |
|  | Domestic Partner | 13.02 | (11.40, 14.64) | 0.82 |  |
| Employment | Employed for an employer | 11.78 | (10.61, 12.95) | 0.60 | < .001 |
|  | Self-employed | 12.17 | (9.69, 14.65) | 1.26 |  |
|  | Retired | 13.45 | (12.36, 14.55) | 0.56 |  |
|  | Student | 4.01 | (1.34, 6.67) | 1.34 |  |
|  | Homemaker | 13.83 | (12.08, 15.59) | 0.89 |  |
|  | Unemployed and looking for a job | 9.94 | (7.78, 12.11) | 1.10 |  |
|  | None of these/Other | 12.99 | (11.41, 14.57) | 0.80 |  |
| Religious service attendance | More than 1/week | 7.46 | (5.47, 9.46) | 1.01 | < .001 |
|  | 1/week | 9.73 | (8.27, 11.19) | 0.74 |  |
|  | 1-3/month | 10.10 | (8.15, 12.05) | 0.99 |  |
|  | A few times a year | 12.82 | (11.44, 14.21) | 0.70 |  |
|  | Never | 12.51 | (11.52, 13.50) | 0.50 |  |
| Education | Up to 8 years | 24.58 | (5.52, 43.65) | 9.29 | < .001 |
|  | 9-15 years | 12.26 | (11.49, 13.02) | 0.39 |  |
|  | 16+ years | 9.64 | (8.99, 10.28) | 0.33 |  |
| Immigration status | Born in this country | 12.15 | (11.42, 12.88) | 0.37 | < .001 |
|  | Born in another country | 8.41 | (6.60, 10.22) | 0.91 |  |
| Religious affiliation | Christianity | 11.87 | (10.95, 12.79) | 0.46 | < .001 |
|  | Islam | 13.34 | (9.57, 17.11) | 1.68 |  |
|  | Hinduism | 3.91 | * | * |  |
|  | Buddhism | 8.89 | (2.53, 15.26) | 3.05 |  |
|  | Judaism | 12.63 | (9.78, 15.47) | 1.34 |  |
|  | Baha'i | 5.56 | * | * |  |
|  | Taoism | 8.47 | (3.48, 13.45) | 2.46 |  |
|  | Primal, Animist, or Folk religion | 12.75 | (6.91, 18.59) | 2.77 |  |
|  | Some other religion | 12.80 | (6.99, 18.61) | 2.80 |  |
|  | No religion/Atheist/Agnostic | 12.40 | (11.06, 13.75) | 0.68 |  |
| Race/Ethnicity | Asian | 7.57 | (2.26, 12.89) | 2.68 | < .001 |
|  | Black | 8.51 | (7.14, 9.89) | 0.70 |  |
|  | White | 13.83 | (13.15, 14.52) | 0.35 |  |
|  | Other | 13.18 | (11.09, 15.27) | 1.06 |  |
|  | Hispanic | 9.64 | (6.86, 12.42) | 1.42 |  |

# Table S46. Population weighted meta-analysis of daily cigarette consumption per smoker (intensity) by demographic category

| Variable | Category | Est | 95% CI | SE |
| --- | --- | --- | --- | --- |
| Age group |  |  |  |  |
|  | 18-24 | 7.29 | (5.72,8.86) | 0.801 |
|  | 25-34 | 9.18 | (7.94,10.42) | 0.633 |
|  | 35-44 | 9.35 | (8.65,10.05) | 0.358 |
|  | 45-54 | 11.03 | (10.01,12.06) | 0.524 |
|  | 55-64 | 10.93 | (9.97,11.89) | 0.489 |
|  | 65-74 | 9.80 | (8.78,10.82) | 0.520 |
|  | 75-84 | 10.45 | (7.14,13.77) | 1.690 |
|  | 85 or older | 8.21 | (6.42,10.00) | 0.913 |
| Gender |  |  |  |  |
|  | Male | 10.03 | (9.55,10.51) | 0.247 |
|  | Female | 10.05 | (7.35,12.76) | 1.378 |
|  | Other | 7.32 | (4.97,9.68) | 1.200 |
| Marital status |  |  |  |  |
|  | Married | 9.71 | (9.29,10.13) | 0.216 |
|  | Separated | 10.04 | (7.88,12.21) | 1.105 |
|  | Divorced | 12.97 | (12.15,13.80) | 0.421 |
|  | Widowed | 14.20 | (9.48,18.92) | 2.409 |
|  | Domestic partner | 7.84 | (6.44,9.23) | 0.713 |
|  | Single, never married | 9.97 | (8.03,11.90) | 0.988 |
| Employment status |  |  |  |  |
|  | Employed for an employer | 8.71 | (8.18,9.25) | 0.271 |
|  | Self-employed | 10.56 | (9.67,11.45) | 0.454 |
|  | Retired | 10.11 | (8.93,11.29) | 0.602 |
|  | Student | 6.54 | (4.71,8.37) | 0.934 |
|  | Homemaker | 10.50 | (8.05,12.94) | 1.246 |
|  | Unemployed and looking for a job | 10.17 | (7.67,12.67) | 1.276 |
|  | None of these/other | 10.55 | (9.32,11.78) | 0.628 |
| Education |  |  |  |  |
|  | Up to 8 years | 11.52 | (9.51,13.54) | 1.028 |
|  | 9-15 years | 9.30 | (8.35,10.25) | 0.484 |
|  | 16+ years | 10.21 | (9.85,10.57) | 0.184 |
| Religious service attendance |  |  |  |  |
|  | >1/week | 9.47 | (8.37,10.56) | 0.560 |
|  | 1/week | 8.94 | (7.90,9.98) | 0.530 |
|  | 1-3/month | 8.56 | (7.77,9.34) | 0.400 |
|  | A few times a year | 10.38 | (9.34,11.42) | 0.529 |
|  | Never | 10.42 | (8.73,12.10) | 0.859 |
| Immigration status |  |  |  |  |
|  | Born in this country | 9.79 | (9.31,10.27) | 0.245 |
|  | Born in another country | 10.50 | (6.44,14.56) | 2.071 |

# Table S47. Random effects meta-analysis of proportion (prevalence) of daily cigarette smoking by demographic category.

|  | | | | | Prediction Interval | |  | | |
| --- | --- | --- | --- | --- | --- | --- | --- | --- | --- |
| Variable | Category | Proportion | 95% CI of Proportion | SE Analogue (CI Width/4) | LL | UL | Heterogeneity (τ) | I^2 | Global p-value |
| Age group |  |  |  |  |  |  |  |  | <.001** |
|  | 18-24 | 0.14 | (0.09,0.20) | 0.03 | 0.02 | 0.51 | 0.12 | 97.0 |  |
|  | 25-34 | 0.20 | (0.14,0.27) | 0.03 | 0.04 | 0.57 | 0.15 | 96.6 |  |
|  | 35-44 | 0.21 | (0.16,0.28) | 0.03 | 0.06 | 0.53 | 0.14 | 95.7 |  |
|  | 45-54 | 0.21 | (0.16,0.27) | 0.03 | 0.05 | 0.56 | 0.13 | 95.8 |  |
|  | 55-64 | 0.20 | (0.15,0.26) | 0.03 | 0.04 | 0.51 | 0.13 | 95.4 |  |
|  | 65-74 | 0.14 | (0.10,0.19) | 0.02 | 0.01 | 0.39 | 0.11 | 96.2 |  |
|  | 75-84 | 0.06 | (0.03,0.11) | 0.02 | 0.00 | 0.25 | 0.10 | 98.6 |  |
|  | 85 or older | 0.02 | (0.00,0.12) | 0.03 | 0.00 | 1.00 | 0.10 | 99.6 |  |
| Gender |  |  |  |  |  |  |  |  | <.001** |
|  | Male | 0.26 | (0.19,0.34) | 0.04 | 0.08 | 0.70 | 0.18 | 96.8 |  |
|  | Female | 0.08 | (0.05,0.15) | 0.02 | 0.00 | 0.42 | 0.11 | 98.3 |  |
|  | Other | 0.18 | (0.03,0.66) | 0.16 | 0.00 | 1.00 | 0.70 | 99.8 |  |
| Marital status |  |  |  |  |  |  |  |  | <.001** |
|  | Married | 0.17 | (0.12,0.23) | 0.03 | 0.05 | 0.49 | 0.12 | 96.0 |  |
|  | Separated | 0.25 | (0.20,0.32) | 0.03 | 0.08 | 0.49 | 0.15 | 95.7 |  |
|  | Divorced | 0.22 | (0.16,0.30) | 0.03 | 0.04 | 0.70 | 0.16 | 96.7 |  |
|  | Widowed | 0.12 | (0.08,0.16) | 0.02 | 0.02 | 0.33 | 0.09 | 95.9 |  |
|  | Domestic partner | 0.22 | (0.18,0.27) | 0.02 | 0.05 | 0.39 | 0.11 | 93.6 |  |
|  | Single, never married | 0.18 | (0.13,0.25) | 0.03 | 0.03 | 0.60 | 0.14 | 96.6 |  |
| Employment status |  |  |  |  |  |  |  |  | <.001** |
|  | Employed for an employer | 0.22 | (0.16,0.29) | 0.03 | 0.04 | 0.59 | 0.16 | 96.8 |  |
|  | Self-employed | 0.24 | (0.17,0.32) | 0.04 | 0.05 | 0.69 | 0.17 | 97.0 |  |
|  | Retired | 0.16 | (0.12,0.20) | 0.02 | 0.05 | 0.45 | 0.09 | 93.5 |  |
|  | Student | 0.10 | (0.06,0.14) | 0.02 | 0.01 | 0.40 | 0.09 | 96.8 |  |
|  | Homemaker | 0.10 | (0.06,0.17) | 0.03 | 0.01 | 0.36 | 0.13 | 98.1 |  |
|  | Unemployed and looking for a job | 0.23 | (0.17,0.32) | 0.04 | 0.04 | 0.67 | 0.18 | 97.2 |  |
|  | None of these/other | 0.23 | (0.18,0.29) | 0.03 | 0.07 | 0.60 | 0.12 | 94.6 |  |
| Education |  |  |  |  |  |  |  |  | <.001** |
|  | Up to 8 years | 0.22 | (0.16,0.28) | 0.03 | 0.05 | 0.49 | 0.14 | 96.0 |  |
|  | 9-15 years | 0.19 | (0.14,0.26) | 0.03 | 0.03 | 0.55 | 0.14 | 96.6 |  |
|  | 16+ years | 0.13 | (0.09,0.18) | 0.02 | 0.02 | 0.50 | 0.11 | 96.3 |  |
| Religious service attendance |  |  |  |  |  |  |  |  | <.001** |
|  | >1/week | 0.15 | (0.10,0.22) | 0.03 | 0.03 | 0.64 | 0.14 | 97.3 |  |
|  | 1/week | 0.18 | (0.12,0.25) | 0.03 | 0.04 | 0.55 | 0.15 | 97.1 |  |
|  | 1-3/month | 0.20 | (0.15,0.27) | 0.03 | 0.05 | 0.53 | 0.15 | 96.5 |  |
|  | A few times a year | 0.20 | (0.15,0.26) | 0.03 | 0.06 | 0.60 | 0.13 | 95.6 |  |
|  | Never | 0.22 | (0.17,0.27) | 0.02 | 0.08 | 0.53 | 0.12 | 94.1 |  |
| Immigration status |  |  |  |  |  |  |  |  | <.001** |
|  | Born in this country | 0.19 | (0.14,0.25) | 0.03 | 0.04 | 0.52 | 0.13 | 96.0 |  |
|  | Born in another country | 0.17 | (0.11,0.24) | 0.03 | 0.02 | 0.54 | 0.15 | 97.4 |  |

*Note.* *p < .05; **p < .007 (Bonferroni corrected threshold).

# Table S48. Proportions by demographic category for Argentina

| Variable | Category | Proportion | 95% CI | SE | Global p-value |
| --- | --- | --- | --- | --- | --- |
| Age group | 18-24 | 0.30 | (0.26, 0.34) | 0.02 | < .001 |
|  | 25-34 | 0.38 | (0.35, 0.42) | 0.02 |  |
|  | 35-44 | 0.40 | (0.36, 0.43) | 0.02 |  |
|  | 45-54 | 0.32 | (0.28, 0.36) | 0.02 |  |
|  | 55-64 | 0.30 | (0.25, 0.34) | 0.02 |  |
|  | 65-74 | 0.27 | (0.22, 0.33) | 0.03 |  |
|  | 75-84 | 0.10 | (0.04, 0.16) | 0.03 |  |
|  | 85 or older | 0.12 | (0.00, 0.32) | 0.10 |  |
| Gender | Male | 0.36 | (0.33, 0.38) | 0.01 | 0.007 |
|  | Female | 0.31 | (0.29, 0.33) | 0.01 |  |
|  | Other | 0.22 | (0.02, 0.43) | 0.10 |  |
| Marital status | Married | 0.24 | (0.21, 0.27) | 0.02 | < .001 |
|  | Separated | 0.44 | (0.38, 0.50) | 0.03 |  |
|  | Divorced | 0.36 | (0.29, 0.43) | 0.03 |  |
|  | Widowed | 0.25 | (0.19, 0.32) | 0.04 |  |
|  | Never | 0.36 | (0.33, 0.39) | 0.01 |  |
|  | Domestic Partner | 0.36 | (0.32, 0.39) | 0.02 |  |
| Employment | Employed for an employer | 0.37 | (0.34, 0.39) | 0.01 | < .001 |
|  | Self-employed | 0.31 | (0.28, 0.35) | 0.02 |  |
|  | Retired | 0.22 | (0.18, 0.27) | 0.02 |  |
|  | Student | 0.24 | (0.18, 0.30) | 0.03 |  |
|  | Homemaker | 0.35 | (0.30, 0.41) | 0.03 |  |
|  | Unemployed and looking for a job | 0.39 | (0.33, 0.44) | 0.03 |  |
|  | None of these/Other | 0.34 | (0.25, 0.43) | 0.05 |  |
| Religious service attendance | More than 1/week | 0.18 | (0.13, 0.23) | 0.03 | < .001 |
|  | 1/week | 0.26 | (0.21, 0.31) | 0.02 |  |
|  | 1-3/month | 0.38 | (0.32, 0.45) | 0.03 |  |
|  | A few times a year | 0.32 | (0.29, 0.34) | 0.02 |  |
|  | Never | 0.37 | (0.35, 0.40) | 0.01 |  |
| Education | Up to 8 years | 0.38 | (0.34, 0.41) | 0.02 | < .001 |
|  | 9-15 years | 0.32 | (0.30, 0.34) | 0.01 |  |
|  | 16+ years | 0.22 | (0.18, 0.25) | 0.02 |  |
| Immigration status | Born in this country | 0.34 | (0.32, 0.35) | 0.01 | 0.001 |
|  | Born in another country | 0.23 | (0.16, 0.29) | 0.03 |  |
| Religious affiliation | Christianity | 0.31 | (0.29, 0.33) | 0.01 | < .001 |
|  | Islam | 0.11 | (0.00, 0.32) | 0.09 |  |
|  | Hinduism | 0.91 | * | * |  |
|  | Buddhism | 0.49 | (0.28, 0.69) | 0.10 |  |
|  | Judaism | 0.38 | (0.14, 0.61) | 0.11 |  |
|  | Sikhism | 0.00 | * | * |  |
|  | Taoism | 0.50 | * | * |  |
|  | Confucianism | 0.00 | * | * |  |
|  | Primal, Animist, or Folk religion | 0.32 | (0.00, 0.76) | 0.17 |  |
|  | Some other religion | 0.42 | (0.30, 0.54) | 0.06 |  |
|  | No religion/Atheist/Agnostic | 0.38 | (0.34, 0.41) | 0.02 |  |
| Race/ethnicity | Asian | 0.37 | (0.16, 0.58) | 0.10 | 0.070 |
|  | Black | 0.42 | (0.25, 0.58) | 0.08 |  |
|  | Indigenous | 0.43 | (0.30, 0.57) | 0.07 |  |
|  | Mestizo(a) | 0.34 | (0.31, 0.37) | 0.02 |  |
|  | Mullato(a) | 0.25 | (0.08, 0.42) | 0.08 |  |
|  | White | 0.31 | (0.29, 0.33) | 0.01 |  |
|  | Other | 0.41 | (0.29, 0.53) | 0.06 |  |

# Table S49. Proportions by demographic category for Australia

| Variable | Category | Proportion | 95% CI | SE | Global p-value |
| --- | --- | --- | --- | --- | --- |
| Age group | 18-24 | 0.06 | (0.02, 0.10) | 0.02 | < .001 |
|  | 25-34 | 0.13 | (0.08, 0.17) | 0.02 |  |
|  | 35-44 | 0.15 | (0.11, 0.19) | 0.02 |  |
|  | 45-54 | 0.16 | (0.12, 0.19) | 0.02 |  |
|  | 55-64 | 0.14 | (0.11, 0.17) | 0.02 |  |
|  | 65-74 | 0.07 | (0.05, 0.09) | 0.01 |  |
|  | 75-84 | 0.04 | (0.01, 0.06) | 0.01 |  |
|  | 85 or older | 0.07 | (0.00, 0.15) | 0.04 |  |
| Gender | Male | 0.14 | (0.11, 0.16) | 0.01 | 0.018 |
|  | Female | 0.10 | (0.08, 0.11) | 0.01 |  |
|  | Other | 0.16 | (0.00, 0.34) | 0.09 |  |
| Marital status | Married | 0.07 | (0.06, 0.09) | 0.01 | < .001 |
|  | Separated | 0.24 | (0.14, 0.33) | 0.05 |  |
|  | Divorced | 0.16 | (0.11, 0.21) | 0.03 |  |
|  | Widowed | 0.09 | (0.05, 0.13) | 0.02 |  |
|  | Never | 0.15 | (0.12, 0.18) | 0.02 |  |
|  | Domestic Partner | 0.17 | (0.12, 0.22) | 0.03 |  |
| Employment | Employed for an employer | 0.12 | (0.10, 0.14) | 0.01 | < .001 |
|  | Self-employed | 0.11 | (0.06, 0.15) | 0.02 |  |
|  | Retired | 0.07 | (0.05, 0.10) | 0.01 |  |
|  | Student | 0.06 | (0.01, 0.11) | 0.03 |  |
|  | Homemaker | 0.18 | (0.08, 0.27) | 0.05 |  |
|  | Unemployed and looking for a job | 0.28 | (0.17, 0.39) | 0.06 |  |
|  | None of these/Other | 0.20 | (0.13, 0.28) | 0.04 |  |
| Religious service attendance | More than 1/week | 0.02 | (0.00, 0.06) | 0.02 | < .001 |
|  | 1/week | 0.04 | (0.01, 0.08) | 0.02 |  |
|  | 1-3/month | 0.06 | (0.00, 0.12) | 0.03 |  |
|  | A few times a year | 0.10 | (0.07, 0.12) | 0.01 |  |
|  | Never | 0.14 | (0.12, 0.16) | 0.01 |  |
| Education | Up to 8 years | 0.31 | (0.14, 0.48) | 0.09 | < .001 |
|  | 9-15 years | 0.14 | (0.12, 0.16) | 0.01 |  |
|  | 16+ years | 0.06 | (0.05, 0.07) | 0.01 |  |
| Immigration status | Born in this country | 0.12 | (0.11, 0.14) | 0.01 | 0.009 |
|  | Born in another country | 0.09 | (0.06, 0.11) | 0.01 |  |
| Religious affiliation | Christianity | 0.09 | (0.07, 0.10) | 0.01 | < .001 |
|  | Islam | 0.11 | (0.00, 0.26) | 0.07 |  |
|  | Hinduism | 0.00 | (0.00, 0.01) | 0.00 |  |
|  | Buddhism | 0.14 | (0.00, 0.29) | 0.07 |  |
|  | Judaism | 0.03 | (0.00, 0.08) | 0.02 |  |
|  | Sikhism | 0.00 | * | * |  |
|  | Baha'i | 0.00 | * | * |  |
|  | Taoism | 0.68 | * | * |  |
|  | Primal, Animist, or Folk religion | 0.10 | (0.00, 0.33) | 0.10 |  |
|  | Some other religion | 0.15 | (0.00, 0.31) | 0.08 |  |
|  | No religion/Atheist/Agnostic | 0.14 | (0.12, 0.16) | 0.01 |  |
| Race/ethnicity | Aboriginal | 0.20 | (0.03, 0.38) | 0.09 | < .001 |
|  | Australian | 0.12 | (0.10, 0.14) | 0.01 |  |
|  | Australian British/European | 0.12 | (0.09, 0.14) | 0.01 |  |
|  | Chinese | 0.06 | (0.00, 0.12) | 0.03 |  |
|  | Indian | 0.01 | (0.00, 0.02) | 0.01 |  |
|  | Japanese | 0.00 | * | * |  |
|  | Malay | 0.00 | * | * |  |
|  | Sinhalese | 0.00 | * | * |  |
|  | Spanish | 0.20 | * | * |  |
|  | Sri Lankan Moor | 0.00 | * | * |  |
|  | Sri Lankan Tamil | 0.00 | * | * |  |
|  | Vietnamese | 0.15 | * | * |  |
|  | Russian | 0.20 | * | * |  |
|  | Samoan | 1.00 | * | * |  |
|  | New Zealander | 0.18 | (0.06, 0.30) | 0.06 |  |
|  | Other European | 0.08 | (0.04, 0.11) | 0.02 |  |
|  | Other | 0.15 | (0.08, 0.23) | 0.04 |  |

# Table S50. Proportions by demographic category for Brazil

| Variable | Category | Proportion | 95% CI | SE | Global p-value |
| --- | --- | --- | --- | --- | --- |
| Age group | 18-24 | 0.11 | (0.10, 0.13) | 0.01 | < .001 |
|  | 25-34 | 0.20 | (0.18, 0.21) | 0.01 |  |
|  | 35-44 | 0.23 | (0.21, 0.25) | 0.01 |  |
|  | 45-54 | 0.21 | (0.19, 0.24) | 0.01 |  |
|  | 55-64 | 0.25 | (0.22, 0.28) | 0.01 |  |
|  | 65-74 | 0.19 | (0.15, 0.23) | 0.02 |  |
|  | 75-84 | 0.04 | (0.00, 0.08) | 0.02 |  |
|  | 85 or older | 0.14 | (0.00, 0.32) | 0.09 |  |
| Gender | Male | 0.23 | (0.22, 0.25) | 0.01 | < .001 |
|  | Female | 0.16 | (0.15, 0.18) | 0.01 |  |
|  | Other | 0.23 | (0.09, 0.38) | 0.07 |  |
| Marital status | Married | 0.14 | (0.12, 0.15) | 0.01 | < .001 |
|  | Separated | 0.30 | (0.25, 0.35) | 0.03 |  |
|  | Divorced | 0.28 | (0.24, 0.32) | 0.02 |  |
|  | Widowed | 0.19 | (0.14, 0.25) | 0.03 |  |
|  | Never | 0.21 | (0.19, 0.22) | 0.01 |  |
|  | Domestic Partner | 0.24 | (0.22, 0.26) | 0.01 |  |
| Employment | Employed for an employer | 0.18 | (0.17, 0.20) | 0.01 | < .001 |
|  | Self-employed | 0.23 | (0.21, 0.25) | 0.01 |  |
|  | Retired | 0.17 | (0.14, 0.20) | 0.02 |  |
|  | Student | 0.07 | (0.05, 0.09) | 0.01 |  |
|  | Homemaker | 0.18 | (0.15, 0.21) | 0.01 |  |
|  | Unemployed and looking for a job | 0.23 | (0.21, 0.26) | 0.01 |  |
|  | None of these/Other | 0.20 | (0.15, 0.25) | 0.03 |  |
| Religious service attendance | More than 1/week | 0.12 | (0.10, 0.13) | 0.01 | < .001 |
|  | 1/week | 0.18 | (0.16, 0.20) | 0.01 |  |
|  | 1-3/month | 0.22 | (0.19, 0.25) | 0.01 |  |
|  | A few times a year | 0.22 | (0.20, 0.24) | 0.01 |  |
|  | Never | 0.23 | (0.21, 0.25) | 0.01 |  |
| Education | Up to 8 years | 0.26 | (0.24, 0.28) | 0.01 | < .001 |
|  | 9-15 years | 0.19 | (0.18, 0.21) | 0.01 |  |
|  | 16+ years | 0.12 | (0.10, 0.14) | 0.01 |  |
| Immigration status | Born in this country | 0.20 | (0.19, 0.21) | 0.00 | 0.540 |
|  | Born in another country | 0.22 | (0.14, 0.31) | 0.04 |  |
| Religious affiliation | Christianity | 0.16 | (0.15, 0.17) | 0.01 | < .001 |
|  | Islam | 0.27 | * | * |  |
|  | Hinduism | 0.53 | * | * |  |
|  | Buddhism | 0.30 | (0.08, 0.53) | 0.11 |  |
|  | Judaism | 0.34 | (0.06, 0.61) | 0.13 |  |
|  | Baha'i | 0.49 | * | * |  |
|  | Jainism | 1.00 | * | * |  |
|  | Shinto | 0.66 | * | * |  |
|  | Taoism | 0.16 | * | * |  |
|  | Confucianism | 0.00 | * | * |  |
|  | Primal, Animist, or Folk religion | 0.07 | * | * |  |
|  | Spiritism | 0.31 | (0.26, 0.36) | 0.02 |  |
|  | Umbanda, Candomble, and other African-derived religions | 0.46 | (0.40, 0.51) | 0.03 |  |
|  | Some other religion | 0.32 | (0.21, 0.43) | 0.05 |  |
|  | No religion/Atheist/Agnostic | 0.26 | (0.23, 0.29) | 0.01 |  |
| Race/ethnicity | Branca | 0.20 | (0.19, 0.22) | 0.01 | 0.220 |
|  | Preta | 0.20 | (0.18, 0.23) | 0.01 |  |
|  | Parda | 0.19 | (0.17, 0.20) | 0.01 |  |
|  | Amarela | 0.23 | (0.17, 0.30) | 0.03 |  |
|  | Indígena | 0.26 | (0.17, 0.35) | 0.05 |  |
|  | Other | 0.26 | (0.13, 0.39) | 0.06 |  |

# Table S51. Proportions by demographic category for Egypt

| Variable | Category | Proportion | 95% CI | SE | Global p-value |
| --- | --- | --- | --- | --- | --- |
| Age group | 18-24 | 0.20 | (0.16, 0.23) | 0.02 | < .001 |
|  | 25-34 | 0.25 | (0.22, 0.28) | 0.02 |  |
|  | 35-44 | 0.22 | (0.19, 0.25) | 0.01 |  |
|  | 45-54 | 0.21 | (0.17, 0.25) | 0.02 |  |
|  | 55-64 | 0.26 | (0.22, 0.30) | 0.02 |  |
|  | 65-74 | 0.18 | (0.11, 0.25) | 0.04 |  |
|  | 75-84 | 0.06 | (0.00, 0.22) | 0.06 |  |
|  | 85 or older | 0.00 | * | * |  |
| Gender | Male | 0.44 | (0.41, 0.46) | 0.01 | < .001 |
|  | Female | 0.01 | (0.00, 0.01) | 0.00 |  |
| Marital status | Married | 0.22 | (0.20, 0.24) | 0.01 | < .001 |
|  | Separated | 0.13 | (0.02, 0.24) | 0.05 |  |
|  | Divorced | 0.13 | (0.06, 0.20) | 0.03 |  |
|  | Widowed | 0.08 | (0.04, 0.12) | 0.02 |  |
|  | Never | 0.29 | (0.24, 0.33) | 0.02 |  |
| Employment | Employed for an employer | 0.38 | (0.34, 0.41) | 0.02 | < .001 |
|  | Self-employed | 0.41 | (0.37, 0.45) | 0.02 |  |
|  | Retired | 0.26 | (0.19, 0.33) | 0.04 |  |
|  | Student | 0.15 | (0.09, 0.21) | 0.03 |  |
|  | Homemaker | 0.01 | (0.00, 0.02) | 0.00 |  |
|  | Unemployed and looking for a job | 0.37 | (0.29, 0.45) | 0.04 |  |
|  | None of these/Other | 0.30 | (0.02, 0.58) | 0.13 |  |
| Religious service attendance | More than 1/week | 0.27 | (0.23, 0.31) | 0.02 | < .001 |
|  | 1/week | 0.28 | (0.24, 0.32) | 0.02 |  |
|  | 1-3/month | 0.27 | (0.21, 0.32) | 0.03 |  |
|  | A few times a year | 0.24 | (0.20, 0.29) | 0.02 |  |
|  | Never | 0.17 | (0.15, 0.19) | 0.01 |  |
| Education | Up to 8 years | 0.24 | (0.22, 0.27) | 0.01 | 0.006 |
|  | 9-15 years | 0.22 | (0.20, 0.24) | 0.01 |  |
|  | 16+ years | 0.16 | (0.11, 0.20) | 0.02 |  |
| Immigration status | Born in this country | 0.22 | (0.21, 0.24) | 0.01 | 0.486 |
|  | Born in another country | 0.19 | (0.00, 0.51) | 0.13 |  |
| Religious affiliation | Christianity | 0.14 | (0.04, 0.24) | 0.05 | < .001 |
|  | Islam | 0.23 | (0.21, 0.24) | 0.01 |  |
|  | Taoism | 0.00 | * | * |  |
| Race/ethnicity | Arab | 0.22 | (0.21, 0.24) | 0.01 | 0.846 |
|  | Turkish | 0.18 | (0.00, 0.70) | 0.16 |  |
|  | Greek | 0.00 | * | * |  |
|  | Bedouin Arab | 0.25 | * | * |  |
|  | Nubian | 0.32 | (0.18, 0.47) | 0.07 |  |

# Table S52. Proportions by demographic category for Germany

| Variable | Category | Proportion | 95% CI | SE | Global p-value |
| --- | --- | --- | --- | --- | --- |
| Age group | 18-24 | 0.20 | (0.16, 0.24) | 0.02 | < .001 |
|  | 25-34 | 0.26 | (0.24, 0.29) | 0.01 |  |
|  | 35-44 | 0.27 | (0.24, 0.29) | 0.01 |  |
|  | 45-54 | 0.33 | (0.29, 0.36) | 0.02 |  |
|  | 55-64 | 0.30 | (0.28, 0.33) | 0.01 |  |
|  | 65-74 | 0.23 | (0.21, 0.26) | 0.01 |  |
|  | 75-84 | 0.19 | (0.14, 0.24) | 0.02 |  |
|  | 85 or older | 0.09 | (0.00, 0.21) | 0.06 |  |
| Gender | Male | 0.27 | (0.26, 0.29) | 0.01 | 0.449 |
|  | Female | 0.26 | (0.24, 0.27) | 0.01 |  |
|  | Other | 0.29 | (0.00, 0.77) | 0.19 |  |
| Marital status | Married | 0.26 | (0.24, 0.28) | 0.01 | 0.032 |
|  | Separated | 0.32 | (0.25, 0.40) | 0.04 |  |
|  | Divorced | 0.31 | (0.28, 0.35) | 0.02 |  |
|  | Widowed | 0.22 | (0.17, 0.28) | 0.03 |  |
|  | Never | 0.26 | (0.24, 0.28) | 0.01 |  |
|  | Domestic Partner | 0.28 | (0.23, 0.32) | 0.02 |  |
| Employment | Employed for an employer | 0.28 | (0.27, 0.30) | 0.01 | < .001 |
|  | Self-employed | 0.28 | (0.24, 0.33) | 0.02 |  |
|  | Retired | 0.23 | (0.21, 0.25) | 0.01 |  |
|  | Student | 0.15 | (0.11, 0.18) | 0.02 |  |
|  | Homemaker | 0.34 | (0.26, 0.41) | 0.04 |  |
|  | Unemployed and looking for a job | 0.42 | (0.35, 0.49) | 0.04 |  |
|  | None of these/Other | 0.28 | (0.20, 0.36) | 0.04 |  |
| Religious service attendance | More than 1/week | 0.24 | (0.18, 0.31) | 0.03 | 0.005 |
|  | 1/week | 0.30 | (0.24, 0.36) | 0.03 |  |
|  | 1-3/month | 0.32 | (0.27, 0.37) | 0.03 |  |
|  | A few times a year | 0.23 | (0.21, 0.26) | 0.01 |  |
|  | Never | 0.27 | (0.26, 0.29) | 0.01 |  |
| Education | Up to 8 years | 0.32 | (0.25, 0.39) | 0.04 | < .001 |
|  | 9-15 years | 0.30 | (0.29, 0.32) | 0.01 |  |
|  | 16+ years | 0.19 | (0.18, 0.21) | 0.01 |  |
| Immigration status | Born in this country | 0.27 | (0.26, 0.28) | 0.01 | 0.295 |
|  | Born in another country | 0.25 | (0.21, 0.29) | 0.02 |  |
| Religious affiliation | Christianity | 0.26 | (0.25, 0.28) | 0.01 | < .001 |
|  | Islam | 0.32 | (0.25, 0.39) | 0.04 |  |
|  | Hinduism | 0.51 | * | * |  |
|  | Buddhism | 0.31 | (0.15, 0.47) | 0.08 |  |
|  | Judaism | 0.19 | (0.00, 0.42) | 0.09 |  |
|  | Sikhism | 0.31 | * | * |  |
|  | Baha'i | 0.43 | * | * |  |
|  | Shinto | 0.00 | * | * |  |
|  | Taoism | 1.00 | * | * |  |
|  | Confucianism | 0.00 | * | * |  |
|  | Primal, Animist, or Folk religion | 0.21 | (0.02, 0.40) | 0.09 |  |
|  | Some other religion | 0.34 | (0.16, 0.52) | 0.09 |  |
|  | No religion/Atheist/Agnostic | 0.26 | (0.25, 0.28) | 0.01 |  |

# Table S53. Proportions by demographic category for Hong Kong

| Variable | Category | Proportion | 95% CI | SE | Global p-value |
| --- | --- | --- | --- | --- | --- |
| Age group | 18-24 | 0.42 | (0.36, 0.48) | 0.03 | < .001 |
|  | 25-34 | 0.35 | (0.30, 0.41) | 0.03 |  |
|  | 35-44 | 0.30 | (0.25, 0.34) | 0.02 |  |
|  | 45-54 | 0.42 | (0.37, 0.46) | 0.02 |  |
|  | 55-64 | 0.36 | (0.32, 0.40) | 0.02 |  |
|  | 65-74 | 0.09 | (0.03, 0.14) | 0.03 |  |
|  | 75-84 | 0.00 | * | * |  |
|  | 85 or older | 0.00 | * | * |  |
| Gender | Male | 0.38 | (0.34, 0.41) | 0.02 | < .001 |
|  | Female | 0.26 | (0.23, 0.29) | 0.02 |  |
|  | Other | 0.00 | * | * |  |
| Marital status | Married | 0.37 | (0.34, 0.40) | 0.01 | < .001 |
|  | Separated | 0.31 | (0.00, 0.84) | 0.23 |  |
|  | Divorced | 0.29 | (0.14, 0.45) | 0.08 |  |
|  | Widowed | 0.05 | (0.00, 0.12) | 0.04 |  |
|  | Never | 0.18 | (0.15, 0.21) | 0.02 |  |
|  | Domestic Partner | 0.16 | (0.03, 0.30) | 0.07 |  |
| Employment | Employed for an employer | 0.36 | (0.34, 0.39) | 0.01 | < .001 |
|  | Self-employed | 0.42 | (0.34, 0.50) | 0.04 |  |
|  | Retired | 0.10 | (0.05, 0.15) | 0.03 |  |
|  | Student | 0.12 | (0.04, 0.19) | 0.04 |  |
|  | Homemaker | 0.23 | (0.08, 0.38) | 0.07 |  |
|  | Unemployed and looking for a job | 0.21 | (0.04, 0.39) | 0.09 |  |
|  | None of these/Other | 0.18 | (0.00, 0.45) | 0.13 |  |
| Religious service attendance | More than 1/week | 0.65 | (0.57, 0.72) | 0.04 | < .001 |
|  | 1/week | 0.41 | (0.35, 0.47) | 0.03 |  |
|  | 1-3/month | 0.54 | (0.47, 0.62) | 0.04 |  |
|  | A few times a year | 0.25 | (0.21, 0.30) | 0.02 |  |
|  | Never | 0.18 | (0.16, 0.21) | 0.01 |  |
| Education | Up to 8 years | 0.42 | (0.33, 0.51) | 0.05 | < .001 |
|  | 9-15 years | 0.34 | (0.32, 0.36) | 0.01 |  |
|  | 16+ years | 0.13 | (0.10, 0.16) | 0.01 |  |
| Immigration status | Born in this country | 0.33 | (0.31, 0.36) | 0.01 | < .001 |
|  | Born in another country | 0.15 | (0.08, 0.22) | 0.04 |  |
| Religious affiliation | Christianity | 0.36 | (0.31, 0.40) | 0.02 | < .001 |
|  | Islam | 0.48 | (0.29, 0.66) | 0.09 |  |
|  | Hinduism | 0.81 | (0.47, 1.00) | 0.11 |  |
|  | Buddhism | 0.50 | (0.43, 0.57) | 0.03 |  |
|  | Judaism | 0.95 | * | * |  |
|  | Sikhism | 1.00 | * | * |  |
|  | Baha'i | 1.00 | * | * |  |
|  | Jainism | 1.00 | * | * |  |
|  | Shinto | 0.11 | (0.00, 0.39) | 0.07 |  |
|  | Taoism | 0.29 | (0.16, 0.43) | 0.07 |  |
|  | Confucianism | 0.50 | * | * |  |
|  | Primal, Animist, or Folk religion | 0.28 | (0.09, 0.47) | 0.08 |  |
|  | Chinese folk/traditional religion | 0.41 | (0.28, 0.55) | 0.07 |  |
|  | Some other religion | 0.59 | * | * |  |
|  | No religion/Atheist/Agnostic | 0.22 | (0.20, 0.25) | 0.01 |  |
| Race/ethnicity | White | 0.22 | (0.00, 0.80) | 0.12 | < .001 |
|  | Other | 0.00 | * | * |  |
|  | Chinese (Cantonese) | 0.30 | (0.28, 0.33) | 0.01 |  |
|  | Chinese (Chaoshan) | 0.45 | (0.36, 0.54) | 0.04 |  |
|  | Chinese (Fujianese) | 0.55 | (0.45, 0.65) | 0.05 |  |
|  | Chinese (Hakka) | 0.32 | (0.23, 0.41) | 0.05 |  |
|  | Chinese (Shanghainese) | 0.37 | (0.20, 0.54) | 0.08 |  |
|  | Chinese (Other ethnicity) | 0.20 | (0.14, 0.25) | 0.03 |  |
|  | East Asian (Korean, Japanese) | 0.62 | * | * |  |
|  | Southeast Asian (Filipino, Indonesian, Thailand) | 0.12 | (0.00, 0.33) | 0.10 |  |
|  | South Asian (Indian, Nepalese, Pakistani) | 0.31 | (0.00, 1.00) | 0.23 |  |
|  | Taiwanese | 0.33 | * | * |  |

# Table S54. Proportions by demographic category for India

| Variable | Category | Proportion | 95% CI | SE | Global p-value |
| --- | --- | --- | --- | --- | --- |
| Age group | 18-24 | 0.06 | (0.05, 0.07) | 0.01 | < .001 |
|  | 25-34 | 0.07 | (0.06, 0.08) | 0.01 |  |
|  | 35-44 | 0.08 | (0.07, 0.09) | 0.01 |  |
|  | 45-54 | 0.10 | (0.09, 0.12) | 0.01 |  |
|  | 55-64 | 0.09 | (0.07, 0.11) | 0.01 |  |
|  | 65-74 | 0.08 | (0.05, 0.11) | 0.01 |  |
|  | 75-84 | 0.08 | (0.02, 0.14) | 0.03 |  |
|  | 85 or older | 0.00 | * | * |  |
| Gender | Male | 0.15 | (0.13, 0.16) | 0.01 | < .001 |
|  | Female | 0.01 | (0.01, 0.01) | 0.00 |  |
| Marital status | Married | 0.08 | (0.07, 0.09) | 0.00 | 0.004 |
|  | Separated | 0.10 | (0.00, 0.24) | 0.07 |  |
|  | Divorced | 0.04 | (0.00, 0.23) | 0.07 |  |
|  | Widowed | 0.04 | (0.02, 0.07) | 0.01 |  |
|  | Never | 0.08 | (0.06, 0.09) | 0.01 |  |
|  | Domestic Partner | 0.11 | (0.06, 0.17) | 0.03 |  |
| Employment | Employed for an employer | 0.12 | (0.10, 0.14) | 0.01 | < .001 |
|  | Self-employed | 0.11 | (0.09, 0.12) | 0.01 |  |
|  | Retired | 0.15 | (0.09, 0.21) | 0.03 |  |
|  | Student | 0.05 | (0.03, 0.08) | 0.01 |  |
|  | Homemaker | 0.02 | (0.01, 0.02) | 0.00 |  |
|  | Unemployed and looking for a job | 0.10 | (0.08, 0.13) | 0.01 |  |
|  | None of these/Other | 0.12 | (0.09, 0.15) | 0.02 |  |
| Religious service attendance | More than 1/week | 0.07 | (0.06, 0.09) | 0.01 | 0.911 |
|  | 1/week | 0.08 | (0.07, 0.10) | 0.01 |  |
|  | 1-3/month | 0.08 | (0.07, 0.09) | 0.01 |  |
|  | A few times a year | 0.08 | (0.07, 0.10) | 0.01 |  |
|  | Never | 0.08 | (0.06, 0.09) | 0.01 |  |
| Education | Up to 8 years | 0.08 | (0.07, 0.09) | 0.00 | 0.002 |
|  | 9-15 years | 0.07 | (0.06, 0.09) | 0.01 |  |
|  | 16+ years | 0.03 | (0.00, 0.06) | 0.01 |  |
| Immigration status | Born in this country | 0.08 | (0.07, 0.08) | 0.00 | 0.005 |
|  | Born in another country | 0.20 | (0.11, 0.28) | 0.04 |  |
| Religious affiliation | Christianity | 0.06 | (0.03, 0.09) | 0.01 | < .001 |
|  | Islam | 0.10 | (0.07, 0.12) | 0.01 |  |
|  | Hinduism | 0.08 | (0.07, 0.09) | 0.00 |  |
|  | Buddhism | 0.03 | (0.00, 0.05) | 0.01 |  |
|  | Sikhism | 0.06 | (0.01, 0.11) | 0.03 |  |
|  | Jainism | 0.06 | * | * |  |
|  | Shinto | 0.00 | * | * |  |
|  | Primal, Animist, or Folk religion | 0.10 | (0.00, 0.26) | 0.08 |  |
|  | Some other religion | 0.00 | (0.00, 0.01) | 0.00 |  |
|  | No religion/Atheist/Agnostic | 0.21 | (0.00, 1.00) | 0.18 |  |
| Race/ethnicity | General | 0.08 | (0.07, 0.10) | 0.01 | 0.002 |
|  | Other backward caste | 0.08 | (0.07, 0.09) | 0.01 |  |
|  | Schedule caste | 0.08 | (0.07, 0.10) | 0.01 |  |
|  | Schedule tribe | 0.05 | (0.04, 0.07) | 0.01 |  |

# Table S55. Proportions by demographic category for Indonesia

| Variable | Category | Proportion | 95% CI | SE | Global p-value |
| --- | --- | --- | --- | --- | --- |
| Age group | 18-24 | 0.32 | (0.28, 0.35) | 0.02 | < .001 |
|  | 25-34 | 0.39 | (0.36, 0.42) | 0.02 |  |
|  | 35-44 | 0.39 | (0.36, 0.42) | 0.02 |  |
|  | 45-54 | 0.40 | (0.36, 0.44) | 0.02 |  |
|  | 55-64 | 0.39 | (0.34, 0.44) | 0.03 |  |
|  | 65-74 | 0.32 | (0.20, 0.43) | 0.06 |  |
|  | 75-84 | 0.00 | * | * |  |
|  | 85 or older | 0.67 | * | * |  |
| Gender | Male | 0.71 | (0.69, 0.73) | 0.01 | < .001 |
|  | Female | 0.05 | (0.04, 0.06) | 0.01 |  |
|  | Other | 0.07 | (0.00, 0.31) | 0.07 |  |
| Marital status | Married | 0.38 | (0.36, 0.40) | 0.01 | < .001 |
|  | Separated | 0.42 | (0.27, 0.57) | 0.08 |  |
|  | Divorced | 0.30 | (0.22, 0.39) | 0.04 |  |
|  | Widowed | 0.23 | (0.17, 0.29) | 0.03 |  |
|  | Never | 0.42 | (0.39, 0.46) | 0.02 |  |
|  | Domestic Partner | 0.34 | (0.00, 0.73) | 0.17 |  |
| Employment | Employed for an employer | 0.53 | (0.49, 0.57) | 0.02 | < .001 |
|  | Self-employed | 0.53 | (0.50, 0.56) | 0.02 |  |
|  | Retired | 0.35 | (0.22, 0.48) | 0.06 |  |
|  | Student | 0.25 | (0.18, 0.31) | 0.03 |  |
|  | Homemaker | 0.06 | (0.04, 0.07) | 0.01 |  |
|  | Unemployed and looking for a job | 0.50 | (0.44, 0.56) | 0.03 |  |
|  | None of these/Other | 0.61 | (0.55, 0.67) | 0.03 |  |
| Religious service attendance | More than 1/week | 0.40 | (0.37, 0.43) | 0.01 | < .001 |
|  | 1/week | 0.39 | (0.36, 0.42) | 0.01 |  |
|  | 1-3/month | 0.31 | (0.27, 0.36) | 0.02 |  |
|  | A few times a year | 0.31 | (0.27, 0.36) | 0.02 |  |
|  | Never | 0.35 | (0.27, 0.42) | 0.04 |  |
| Education | Up to 8 years | 0.39 | (0.36, 0.42) | 0.02 | < .001 |
|  | 9-15 years | 0.38 | (0.36, 0.40) | 0.01 |  |
|  | 16+ years | 0.25 | (0.20, 0.29) | 0.02 |  |
| Immigration status | Born in this country | 0.38 | (0.36, 0.39) | 0.01 | 0.189 |
|  | Born in another country | 0.29 | (0.11, 0.48) | 0.09 |  |
| Religious affiliation | Christianity | 0.32 | (0.25, 0.38) | 0.03 | < .001 |
|  | Islam | 0.38 | (0.36, 0.40) | 0.01 |  |
|  | Hinduism | 0.16 | (0.02, 0.31) | 0.07 |  |
|  | Buddhism | 0.75 | * | * |  |
|  | Taoism | 0.00 | * | * |  |
|  | Some other religion | 0.00 | * | * |  |
| Race/ethnicity | Banjar/Melayu Banjar | 0.36 | (0.28, 0.43) | 0.04 | 0.010 |
|  | Betawi | 0.39 | (0.31, 0.46) | 0.04 |  |
|  | Bugis | 0.34 | (0.28, 0.39) | 0.03 |  |
|  | Jawa | 0.35 | (0.33, 0.38) | 0.01 |  |
|  | Madura | 0.36 | (0.29, 0.43) | 0.03 |  |
|  | Minangkabau | 0.40 | (0.34, 0.46) | 0.03 |  |
|  | Sunda/Parahyangan | 0.47 | (0.42, 0.53) | 0.03 |  |
|  | Bali | 0.20 | (0.03, 0.37) | 0.08 |  |
|  | Batak | 0.39 | (0.30, 0.48) | 0.05 |  |
|  | Makasar | 0.35 | (0.24, 0.45) | 0.05 |  |
|  | Other | 0.35 | (0.31, 0.39) | 0.02 |  |

# Table S56. Proportions by demographic category for Israel

| Variable | Category | Proportion | 95% CI | SE | Global p-value |
| --- | --- | --- | --- | --- | --- |
| Age group | 18-24 | 0.17 | (0.13, 0.22) | 0.02 | < .001 |
|  | 25-34 | 0.26 | (0.21, 0.30) | 0.02 |  |
|  | 35-44 | 0.31 | (0.26, 0.35) | 0.02 |  |
|  | 45-54 | 0.26 | (0.23, 0.30) | 0.02 |  |
|  | 55-64 | 0.26 | (0.22, 0.30) | 0.02 |  |
|  | 65-74 | 0.17 | (0.12, 0.21) | 0.02 |  |
|  | 75-84 | 0.08 | (0.04, 0.13) | 0.02 |  |
|  | 85 or older | 0.23 | (0.00, 0.67) | 0.13 |  |
| Gender | Male | 0.34 | (0.31, 0.38) | 0.02 | < .001 |
|  | Female | 0.13 | (0.11, 0.15) | 0.01 |  |
|  | Other | 1.00 | * | * |  |
| Marital status | Married | 0.24 | (0.21, 0.26) | 0.01 | < .001 |
|  | Separated | 0.35 | (0.20, 0.49) | 0.07 |  |
|  | Divorced | 0.34 | (0.25, 0.42) | 0.04 |  |
|  | Widowed | 0.12 | (0.07, 0.17) | 0.02 |  |
|  | Never | 0.21 | (0.18, 0.25) | 0.02 |  |
|  | Domestic Partner | 0.27 | (0.19, 0.34) | 0.04 |  |
| Employment | Employed for an employer | 0.25 | (0.22, 0.28) | 0.02 | < .001 |
|  | Self-employed | 0.35 | (0.30, 0.40) | 0.03 |  |
|  | Retired | 0.15 | (0.11, 0.19) | 0.02 |  |
|  | Student | 0.20 | (0.15, 0.26) | 0.03 |  |
|  | Homemaker | 0.09 | (0.04, 0.13) | 0.02 |  |
|  | Unemployed and looking for a job | 0.25 | (0.14, 0.37) | 0.06 |  |
|  | None of these/Other | 0.27 | (0.16, 0.37) | 0.05 |  |
| Religious service attendance | More than 1/week | 0.22 | (0.17, 0.26) | 0.02 | 0.102 |
|  | 1/week | 0.19 | (0.14, 0.25) | 0.03 |  |
|  | 1-3/month | 0.21 | (0.16, 0.26) | 0.02 |  |
|  | A few times a year | 0.25 | (0.21, 0.28) | 0.02 |  |
|  | Never | 0.26 | (0.22, 0.30) | 0.02 |  |
| Education | Up to 8 years | 0.25 | (0.15, 0.34) | 0.05 | 0.181 |
|  | 9-15 years | 0.25 | (0.23, 0.28) | 0.01 |  |
|  | 16+ years | 0.22 | (0.19, 0.25) | 0.01 |  |
| Immigration status | Born in this country | 0.24 | (0.21, 0.26) | 0.01 | 0.856 |
|  | Born in another country | 0.23 | (0.19, 0.27) | 0.02 |  |
| Religious affiliation | Christianity | 0.42 | (0.28, 0.56) | 0.07 | 0.009 |
|  | Islam | 0.25 | (0.20, 0.30) | 0.03 |  |
|  | Judaism | 0.23 | (0.20, 0.25) | 0.01 |  |
|  | Baha'i | 0.00 | * | * |  |
|  | Taoism | 0.00 | * | * |  |
|  | Primal, Animist, or Folk religion | 0.00 | * | * |  |
|  | Some other religion | 0.38 | (0.00, 0.96) | 0.29 |  |
|  | No religion/Atheist/Agnostic | 0.35 | (0.21, 0.48) | 0.07 |  |
| Race/ethnicity | Arab | 0.26 | (0.21, 0.31) | 0.03 | 0.467 |
|  | Jewish | 0.23 | (0.20, 0.25) | 0.01 |  |
|  | Other | 0.29 | (0.12, 0.45) | 0.08 |  |

# Table S57. Proportions by demographic category for Japan

| Variable | Category | Proportion | 95% CI | SE | Global p-value |
| --- | --- | --- | --- | --- | --- |
| Age group | 18-24 | 0.12 | (0.09, 0.14) | 0.01 | < .001 |
|  | 25-34 | 0.20 | (0.18, 0.22) | 0.01 |  |
|  | 35-44 | 0.28 | (0.26, 0.30) | 0.01 |  |
|  | 45-54 | 0.29 | (0.27, 0.31) | 0.01 |  |
|  | 55-64 | 0.29 | (0.28, 0.31) | 0.01 |  |
|  | 65-74 | 0.18 | (0.17, 0.19) | 0.01 |  |
|  | 75-84 | 0.10 | (0.08, 0.12) | 0.01 |  |
|  | 85 or older | 0.06 | (0.00, 0.13) | 0.04 |  |
| Gender | Male | 0.31 | (0.30, 0.33) | 0.01 | < .001 |
|  | Female | 0.14 | (0.13, 0.15) | 0.00 |  |
|  | Other | 0.10 | (0.00, 0.21) | 0.05 |  |
| Marital status | Married | 0.23 | (0.22, 0.24) | 0.00 | < .001 |
|  | Separated | 0.38 | (0.29, 0.47) | 0.05 |  |
|  | Divorced | 0.30 | (0.28, 0.33) | 0.01 |  |
|  | Widowed | 0.12 | (0.09, 0.14) | 0.01 |  |
|  | Never | 0.19 | (0.18, 0.20) | 0.01 |  |
|  | Domestic Partner | 0.36 | (0.26, 0.46) | 0.05 |  |
| Employment | Employed for an employer | 0.26 | (0.25, 0.27) | 0.01 | < .001 |
|  | Self-employed | 0.31 | (0.28, 0.33) | 0.01 |  |
|  | Retired | 0.17 | (0.15, 0.18) | 0.01 |  |
|  | Student | 0.04 | (0.03, 0.06) | 0.01 |  |
|  | Homemaker | 0.10 | (0.08, 0.12) | 0.01 |  |
|  | Unemployed and looking for a job | 0.23 | (0.20, 0.26) | 0.02 |  |
|  | None of these/Other | 0.15 | (0.14, 0.16) | 0.01 |  |
| Religious service attendance | More than 1/week | 0.27 | (0.21, 0.32) | 0.03 | < .001 |
|  | 1/week | 0.34 | (0.28, 0.41) | 0.03 |  |
|  | 1-3/month | 0.28 | (0.24, 0.31) | 0.02 |  |
|  | A few times a year | 0.26 | (0.24, 0.28) | 0.01 |  |
|  | Never | 0.21 | (0.20, 0.22) | 0.00 |  |
| Education | Up to 8 years | 0.29 | (0.24, 0.33) | 0.02 | < .001 |
|  | 9-15 years | 0.24 | (0.23, 0.25) | 0.00 |  |
|  | 16+ years | 0.16 | (0.15, 0.18) | 0.01 |  |
| Immigration status | Born in this country | 0.22 | (0.22, 0.23) | 0.00 | 0.376 |
|  | Born in another country | 0.26 | (0.17, 0.35) | 0.04 |  |
| Religious affiliation | Christianity | 0.19 | (0.14, 0.24) | 0.03 | < .001 |
|  | Islam | 0.58 | * | * |  |
|  | Hinduism | 0.91 | * | * |  |
|  | Buddhism | 0.23 | (0.22, 0.24) | 0.01 |  |
|  | Judaism | 0.93 | * | * |  |
|  | Sikhism | 0.97 | * | * |  |
|  | Baha'i | 0.78 | * | * |  |
|  | Jainism | 0.78 | (0.37, 1.00) | 0.19 |  |
|  | Shinto | 0.23 | (0.18, 0.27) | 0.02 |  |
|  | Taoism | 0.00 | * | * |  |
|  | Confucianism | 0.31 | (0.00, 0.83) | 0.12 |  |
|  | Primal, Animist, or Folk religion | 0.44 | (0.00, 1.00) | 0.17 |  |
|  | Some other religion | 0.19 | (0.05, 0.34) | 0.07 |  |
|  | No religion/Atheist/Agnostic | 0.22 | (0.21, 0.23) | 0.00 |  |

# Table S58. Proportions by demographic category for Kenya

| Variable | Category | Proportion | 95% CI | SE | Global p-value |
| --- | --- | --- | --- | --- | --- |
| Age group | 18-24 | 0.03 | (0.02, 0.03) | 0.00 | < .001 |
|  | 25-34 | 0.04 | (0.04, 0.05) | 0.00 |  |
|  | 35-44 | 0.06 | (0.05, 0.08) | 0.01 |  |
|  | 45-54 | 0.08 | (0.06, 0.10) | 0.01 |  |
|  | 55-64 | 0.08 | (0.05, 0.11) | 0.01 |  |
|  | 65-74 | 0.09 | (0.05, 0.13) | 0.02 |  |
|  | 75-84 | 0.03 | (0.00, 0.06) | 0.02 |  |
|  | 85 or older | 0.00 | * | * |  |
| Gender | Male | 0.10 | (0.09, 0.11) | 0.01 | < .001 |
|  | Female | 0.01 | (0.01, 0.01) | 0.00 |  |
|  | Other | 0.00 | * | * |  |
| Marital status | Married | 0.05 | (0.05, 0.06) | 0.00 | < .001 |
|  | Separated | 0.11 | (0.08, 0.15) | 0.02 |  |
|  | Divorced | 0.12 | (0.05, 0.20) | 0.04 |  |
|  | Widowed | 0.04 | (0.01, 0.08) | 0.02 |  |
|  | Never | 0.04 | (0.03, 0.05) | 0.00 |  |
|  | Domestic Partner | 0.13 | (0.05, 0.21) | 0.04 |  |
| Employment | Employed for an employer | 0.07 | (0.05, 0.08) | 0.01 | < .001 |
|  | Self-employed | 0.07 | (0.06, 0.08) | 0.01 |  |
|  | Retired | 0.10 | (0.05, 0.15) | 0.03 |  |
|  | Student | 0.02 | (0.01, 0.02) | 0.00 |  |
|  | Homemaker | 0.01 | (0.00, 0.02) | 0.00 |  |
|  | Unemployed and looking for a job | 0.05 | (0.04, 0.06) | 0.01 |  |
|  | None of these/Other | 0.16 | (0.07, 0.24) | 0.04 |  |
| Religious service attendance | More than 1/week | 0.03 | (0.02, 0.03) | 0.00 | < .001 |
|  | 1/week | 0.04 | (0.03, 0.05) | 0.00 |  |
|  | 1-3/month | 0.07 | (0.06, 0.09) | 0.01 |  |
|  | A few times a year | 0.14 | (0.11, 0.17) | 0.02 |  |
|  | Never | 0.19 | (0.14, 0.25) | 0.03 |  |
| Education | Up to 8 years | 0.07 | (0.05, 0.08) | 0.01 | 0.002 |
|  | 9-15 years | 0.04 | (0.04, 0.05) | 0.00 |  |
|  | 16+ years | 0.06 | (0.04, 0.08) | 0.01 |  |
| Immigration status | Born in this country | 0.05 | (0.05, 0.06) | 0.00 | 0.834 |
|  | Born in another country | 0.05 | (0.00, 0.11) | 0.03 |  |
| Religious affiliation | Christianity | 0.05 | (0.04, 0.05) | 0.00 | < .001 |
|  | Islam | 0.09 | (0.06, 0.11) | 0.01 |  |
|  | Buddhism | 0.00 | * | * |  |
|  | Judaism | 0.00 | * | * |  |
|  | Baha'i | 0.00 | * | * |  |
|  | Jainism | 0.00 | * | * |  |
|  | Confucianism | 0.00 | * | * |  |
|  | Primal, Animist, or Folk religion | 0.41 | (0.07, 0.75) | 0.17 |  |
|  | Some other religion | 0.00 | * | * |  |
|  | No religion/Atheist/Agnostic | 0.19 | (0.10, 0.28) | 0.05 |  |
| Race/ethnicity | Luhya | 0.03 | (0.02, 0.04) | 0.01 | < .001 |
|  | Luo | 0.03 | (0.02, 0.04) | 0.01 |  |
|  | Kalenjin | 0.03 | (0.02, 0.04) | 0.01 |  |
|  | Kamba | 0.08 | (0.06, 0.10) | 0.01 |  |
|  | Kikuyu | 0.08 | (0.06, 0.10) | 0.01 |  |
|  | Kisii | 0.03 | (0.00, 0.05) | 0.01 |  |
|  | Maasai | 0.03 | (0.00, 0.05) | 0.01 |  |
|  | Meru | 0.10 | (0.08, 0.12) | 0.01 |  |
|  | Kenyan Somali/Somali | 0.08 | (0.03, 0.12) | 0.02 |  |
|  | Miji Kenda tribes | 0.06 | (0.03, 0.09) | 0.01 |  |
|  | Embu | 0.03 | (0.00, 0.08) | 0.02 |  |
|  | Other | 0.05 | (0.02, 0.07) | 0.01 |  |

# Table S59. Proportions by demographic category for Mexico

| Variable | Category | Proportion | 95% CI | SE | Global p-value |
| --- | --- | --- | --- | --- | --- |
| Age group | 18-24 | 0.25 | (0.21, 0.28) | 0.02 | < .001 |
|  | 25-34 | 0.28 | (0.25, 0.31) | 0.02 |  |
|  | 35-44 | 0.26 | (0.22, 0.29) | 0.02 |  |
|  | 45-54 | 0.18 | (0.15, 0.21) | 0.02 |  |
|  | 55-64 | 0.21 | (0.17, 0.25) | 0.02 |  |
|  | 65-74 | 0.19 | (0.13, 0.25) | 0.03 |  |
|  | 75-84 | 0.14 | (0.06, 0.22) | 0.04 |  |
|  | 85 or older | 0.22 | (0.00, 0.55) | 0.15 |  |
| Gender | Male | 0.33 | (0.31, 0.35) | 0.01 | < .001 |
|  | Female | 0.14 | (0.13, 0.16) | 0.01 |  |
|  | Other | 0.41 | * | * |  |
| Marital status | Married | 0.19 | (0.17, 0.21) | 0.01 | < .001 |
|  | Separated | 0.30 | (0.24, 0.35) | 0.03 |  |
|  | Divorced | 0.30 | (0.23, 0.37) | 0.04 |  |
|  | Widowed | 0.22 | (0.16, 0.29) | 0.03 |  |
|  | Never | 0.27 | (0.24, 0.30) | 0.02 |  |
|  | Domestic Partner | 0.24 | (0.21, 0.27) | 0.02 |  |
| Employment | Employed for an employer | 0.30 | (0.27, 0.32) | 0.01 | < .001 |
|  | Self-employed | 0.26 | (0.23, 0.30) | 0.02 |  |
|  | Retired | 0.21 | (0.15, 0.28) | 0.03 |  |
|  | Student | 0.19 | (0.12, 0.26) | 0.04 |  |
|  | Homemaker | 0.11 | (0.09, 0.13) | 0.01 |  |
|  | Unemployed and looking for a job | 0.26 | (0.22, 0.31) | 0.02 |  |
|  | None of these/Other | 0.28 | (0.18, 0.38) | 0.05 |  |
| Religious service attendance | More than 1/week | 0.12 | (0.09, 0.15) | 0.02 | < .001 |
|  | 1/week | 0.17 | (0.14, 0.20) | 0.01 |  |
|  | 1-3/month | 0.23 | (0.19, 0.27) | 0.02 |  |
|  | A few times a year | 0.28 | (0.25, 0.31) | 0.01 |  |
|  | Never | 0.28 | (0.25, 0.31) | 0.02 |  |
| Education | Up to 8 years | 0.23 | (0.20, 0.26) | 0.02 | 0.142 |
|  | 9-15 years | 0.24 | (0.22, 0.26) | 0.01 |  |
|  | 16+ years | 0.21 | (0.18, 0.24) | 0.01 |  |
| Immigration status | Born in this country | 0.23 | (0.22, 0.25) | 0.01 | 0.037 |
|  | Born in another country | 0.36 | (0.24, 0.48) | 0.06 |  |
| Religious affiliation | Christianity | 0.22 | (0.21, 0.24) | 0.01 | < .001 |
|  | Islam | 0.00 | * | * |  |
|  | Hinduism | 0.45 | * | * |  |
|  | Buddhism | 0.16 | * | * |  |
|  | Judaism | 0.30 | * | * |  |
|  | Baha'i | 0.00 | * | * |  |
|  | Jainism | 1.00 | * | * |  |
|  | Shinto | 0.67 | * | * |  |
|  | Taoism | 0.94 | * | * |  |
|  | Confucianism | 1.00 | * | * |  |
|  | Primal, Animist, or Folk religion | 0.30 | (0.00, 0.68) | 0.15 |  |
|  | Some other religion | 0.41 | (0.23, 0.60) | 0.09 |  |
|  | No religion/Atheist/Agnostic | 0.29 | (0.25, 0.33) | 0.02 |  |
| Race/ethnicity | Black | 0.24 | (0.15, 0.32) | 0.04 | 0.012 |
|  | Indigenous | 0.18 | (0.14, 0.22) | 0.02 |  |
|  | White | 0.25 | (0.21, 0.28) | 0.02 |  |
|  | Mestizo | 0.24 | (0.22, 0.26) | 0.01 |  |
|  | Mulatto | 0.40 | (0.26, 0.55) | 0.07 |  |
|  | Other | 0.22 | (0.17, 0.28) | 0.03 |  |

# Table S60. Proportions by demographic category for Nigeria

| Variable | Category | Proportion | 95% CI | SE | Global p-value |
| --- | --- | --- | --- | --- | --- |
| Age group | 18-24 | 0.03 | (0.02, 0.04) | 0.00 | < .001 |
|  | 25-34 | 0.04 | (0.03, 0.05) | 0.00 |  |
|  | 35-44 | 0.07 | (0.05, 0.09) | 0.01 |  |
|  | 45-54 | 0.05 | (0.03, 0.08) | 0.01 |  |
|  | 55-64 | 0.04 | (0.00, 0.08) | 0.02 |  |
|  | 65-74 | 0.01 | (0.00, 0.02) | 0.01 |  |
|  | 75-84 | 0.11 | (0.00, 0.26) | 0.08 |  |
|  | 85 or older | 0.09 | (0.00, 0.31) | 0.09 |  |
| Gender | Male | 0.08 | (0.07, 0.10) | 0.01 | < .001 |
|  | Female | 0.01 | (0.01, 0.01) | 0.00 |  |
|  | Other | 0.00 | * | * |  |
| Marital status | Married | 0.05 | (0.04, 0.06) | 0.01 | 0.322 |
|  | Separated | 0.10 | (0.01, 0.20) | 0.05 |  |
|  | Divorced | 0.07 | (0.01, 0.13) | 0.03 |  |
|  | Widowed | 0.02 | (0.00, 0.06) | 0.02 |  |
|  | Never | 0.04 | (0.03, 0.05) | 0.00 |  |
|  | Domestic Partner | 0.23 | (0.00, 0.64) | 0.15 |  |
| Employment | Employed for an employer | 0.05 | (0.03, 0.07) | 0.01 | 0.157 |
|  | Self-employed | 0.04 | (0.03, 0.05) | 0.01 |  |
|  | Retired | 0.08 | (0.00, 0.16) | 0.04 |  |
|  | Student | 0.03 | (0.02, 0.04) | 0.01 |  |
|  | Homemaker | 0.05 | (0.01, 0.10) | 0.02 |  |
|  | Unemployed and looking for a job | 0.04 | (0.02, 0.06) | 0.01 |  |
|  | None of these/Other | 0.07 | (0.01, 0.12) | 0.03 |  |
| Religious service attendance | More than 1/week | 0.04 | (0.03, 0.05) | 0.01 | 0.630 |
|  | 1/week | 0.05 | (0.03, 0.06) | 0.01 |  |
|  | 1-3/month | 0.05 | (0.03, 0.08) | 0.01 |  |
|  | A few times a year | 0.06 | (0.03, 0.09) | 0.02 |  |
|  | Never | 0.10 | (0.00, 0.21) | 0.05 |  |
| Education | Up to 8 years | 0.06 | (0.04, 0.07) | 0.01 | 0.200 |
|  | 9-15 years | 0.04 | (0.03, 0.04) | 0.00 |  |
|  | 16+ years | 0.03 | (0.01, 0.05) | 0.01 |  |
| Immigration status | Born in this country | 0.05 | (0.04, 0.05) | 0.00 | 0.083 |
|  | Born in another country | 0.02 | (0.00, 0.05) | 0.02 |  |
| Religious affiliation | Christianity | 0.04 | (0.03, 0.05) | 0.00 | 0.230 |
|  | Islam | 0.05 | (0.04, 0.07) | 0.01 |  |
|  | Shinto | 0.00 | * | * |  |
|  | Confucianism | 0.00 | * | * |  |
|  | Primal, Animist, or Folk religion | 0.25 | (0.00, 0.59) | 0.16 |  |
|  | Some other religion | 0.00 | * | * |  |
|  | No religion/Atheist/Agnostic | 0.00 | * | * |  |
| Race/ethnicity | Hausa | 0.05 | (0.04, 0.07) | 0.01 | < .001 |
|  | Yoruba | 0.03 | (0.02, 0.04) | 0.01 |  |
|  | Igbo (Ibo) | 0.04 | (0.03, 0.05) | 0.01 |  |
|  | Edo | 0.06 | (0.00, 0.12) | 0.03 |  |
|  | Urhobo | 0.00 | * | * |  |
|  | Fulani | 0.05 | (0.00, 0.10) | 0.02 |  |
|  | Kanuri | 0.16 | (0.01, 0.32) | 0.07 |  |
|  | Tiv | 0.06 | (0.03, 0.10) | 0.02 |  |
|  | Efik | 0.03 | (0.00, 0.07) | 0.02 |  |
|  | Ijaw | 0.02 | (0.00, 0.04) | 0.01 |  |
|  | Igala | 0.04 | (0.00, 0.08) | 0.02 |  |
|  | Ibibio | 0.01 | (0.00, 0.03) | 0.01 |  |
|  | Idoma | 0.07 | (0.00, 0.14) | 0.03 |  |
|  | Other | 0.05 | (0.02, 0.07) | 0.01 |  |

# Table S61. Proportions by demographic category for Philippines

| Variable | Category | Proportion | 95% CI | SE | Global p-value |
| --- | --- | --- | --- | --- | --- |
| Age group | 18-24 | 0.16 | (0.12, 0.19) | 0.02 | < .001 |
|  | 25-34 | 0.26 | (0.22, 0.29) | 0.02 |  |
|  | 35-44 | 0.26 | (0.23, 0.29) | 0.02 |  |
|  | 45-54 | 0.22 | (0.19, 0.25) | 0.02 |  |
|  | 55-64 | 0.20 | (0.15, 0.24) | 0.02 |  |
|  | 65-74 | 0.16 | (0.10, 0.22) | 0.03 |  |
|  | 75-84 | 0.15 | (0.05, 0.24) | 0.05 |  |
|  | 85 or older | 0.00 | * | * |  |
| Gender | Male | 0.38 | (0.35, 0.41) | 0.01 | < .001 |
|  | Female | 0.06 | (0.05, 0.07) | 0.00 |  |
|  | Other | 0.07 | (0.00, 0.23) | 0.07 |  |
| Marital status | Married | 0.22 | (0.20, 0.24) | 0.01 | < .001 |
|  | Separated | 0.25 | (0.18, 0.31) | 0.03 |  |
|  | Divorced | 0.23 | (0.00, 0.80) | 0.14 |  |
|  | Widowed | 0.13 | (0.08, 0.18) | 0.03 |  |
|  | Never | 0.18 | (0.14, 0.21) | 0.02 |  |
|  | Domestic Partner | 0.27 | (0.24, 0.30) | 0.02 |  |
| Employment | Employed for an employer | 0.33 | (0.29, 0.36) | 0.02 | < .001 |
|  | Self-employed | 0.28 | (0.24, 0.31) | 0.02 |  |
|  | Retired | 0.19 | (0.09, 0.28) | 0.05 |  |
|  | Student | 0.08 | (0.05, 0.11) | 0.02 |  |
|  | Homemaker | 0.08 | (0.06, 0.10) | 0.01 |  |
|  | Unemployed and looking for a job | 0.23 | (0.18, 0.27) | 0.02 |  |
|  | None of these/Other | 0.17 | (0.08, 0.27) | 0.05 |  |
| Religious service attendance | More than 1/week | 0.20 | (0.16, 0.23) | 0.02 | < .001 |
|  | 1/week | 0.18 | (0.16, 0.21) | 0.01 |  |
|  | 1-3/month | 0.24 | (0.21, 0.27) | 0.02 |  |
|  | A few times a year | 0.26 | (0.23, 0.30) | 0.02 |  |
|  | Never | 0.30 | (0.22, 0.38) | 0.04 |  |
| Education | Up to 8 years | 0.27 | (0.24, 0.31) | 0.02 | < .001 |
|  | 9-15 years | 0.21 | (0.19, 0.23) | 0.01 |  |
|  | 16+ years | 0.16 | (0.11, 0.21) | 0.02 |  |
| Immigration status | Born in this country | 0.22 | (0.20, 0.23) | 0.01 | 0.532 |
|  | Born in another country | 0.33 | (0.00, 0.89) | 0.19 |  |
| Religious affiliation | Christianity | 0.21 | (0.20, 0.23) | 0.01 | 0.008 |
|  | Islam | 0.25 | (0.20, 0.30) | 0.02 |  |
|  | Buddhism | 0.49 | * | * |  |
|  | Judaism | 0.83 | * | * |  |
|  | Baha'i | 0.00 | * | * |  |
|  | Primal, Animist, or Folk religion | 0.13 | (0.00, 0.40) | 0.14 |  |
|  | Some other religion | 0.24 | (0.11, 0.37) | 0.06 |  |
|  | No religion/Atheist/Agnostic | 0.36 | (0.05, 0.67) | 0.14 |  |
| Race/ethnicity | Tagalog | 0.24 | (0.21, 0.27) | 0.01 | < .001 |
|  | Cebuano | 0.22 | (0.17, 0.27) | 0.03 |  |
|  | Ilocano/Ilokano | 0.22 | (0.15, 0.28) | 0.03 |  |
|  | Visayan/Bisaya | 0.14 | (0.10, 0.18) | 0.02 |  |
|  | Ilonggo/Hiligaynon | 0.24 | (0.19, 0.29) | 0.02 |  |
|  | Bicolano/Bikolano | 0.23 | (0.16, 0.30) | 0.03 |  |
|  | Waray | 0.18 | (0.12, 0.24) | 0.03 |  |
|  | Tausug | 0.26 | (0.15, 0.38) | 0.06 |  |
|  | Maranao | 0.24 | (0.10, 0.39) | 0.07 |  |
|  | Maguindanaoan | 0.17 | (0.11, 0.22) | 0.03 |  |
|  | Chinese-Filipino | 0.00 | * | * |  |
|  | Kapampangan | 0.28 | (0.17, 0.40) | 0.06 |  |
|  | Pangasinense | 0.27 | (0.15, 0.39) | 0.06 |  |
|  | Zamboangueno | 0.31 | (0.20, 0.41) | 0.05 |  |
|  | Masbateno | 0.32 | (0.21, 0.42) | 0.05 |  |
|  | Aeta | 0.00 | * | * |  |
|  | Igorot | 0.21 | (0.11, 0.32) | 0.05 |  |
|  | Mangyan | 0.00 | * | * |  |
|  | Badjao | 0.00 | * | * |  |
|  | Other | 0.17 | (0.11, 0.23) | 0.03 |  |

# Table S62. Proportions by demographic category for Poland

| Variable | Category | Proportion | 95% CI | SE | Global p-value |
| --- | --- | --- | --- | --- | --- |
| Age group | 18-24 | 0.27 | (0.22, 0.32) | 0.03 | < .001 |
|  | 25-34 | 0.33 | (0.30, 0.36) | 0.01 |  |
|  | 35-44 | 0.34 | (0.30, 0.37) | 0.02 |  |
|  | 45-54 | 0.36 | (0.32, 0.39) | 0.02 |  |
|  | 55-64 | 0.35 | (0.31, 0.39) | 0.02 |  |
|  | 65-74 | 0.20 | (0.17, 0.24) | 0.02 |  |
|  | 75-84 | 0.11 | (0.05, 0.18) | 0.03 |  |
|  | 85 or older | 0.13 | (0.00, 0.30) | 0.08 |  |
| Gender | Male | 0.40 | (0.38, 0.43) | 0.01 | < .001 |
|  | Female | 0.21 | (0.19, 0.23) | 0.01 |  |
|  | Other | 1.00 | * | * |  |
| Marital status | Married | 0.28 | (0.26, 0.30) | 0.01 | < .001 |
|  | Separated | 0.45 | (0.34, 0.56) | 0.06 |  |
|  | Divorced | 0.45 | (0.39, 0.52) | 0.03 |  |
|  | Widowed | 0.24 | (0.19, 0.29) | 0.03 |  |
|  | Never | 0.33 | (0.30, 0.37) | 0.02 |  |
|  | Domestic Partner | 0.40 | (0.34, 0.45) | 0.03 |  |
| Employment | Employed for an employer | 0.33 | (0.31, 0.35) | 0.01 | < .001 |
|  | Self-employed | 0.41 | (0.37, 0.45) | 0.02 |  |
|  | Retired | 0.20 | (0.17, 0.23) | 0.02 |  |
|  | Student | 0.20 | (0.14, 0.25) | 0.03 |  |
|  | Homemaker | 0.29 | (0.20, 0.39) | 0.05 |  |
|  | Unemployed and looking for a job | 0.50 | (0.36, 0.64) | 0.07 |  |
|  | None of these/Other | 0.40 | (0.29, 0.51) | 0.06 |  |
| Religious service attendance | More than 1/week | 0.12 | (0.05, 0.19) | 0.03 | < .001 |
|  | 1/week | 0.20 | (0.17, 0.22) | 0.01 |  |
|  | 1-3/month | 0.33 | (0.29, 0.37) | 0.02 |  |
|  | A few times a year | 0.37 | (0.34, 0.40) | 0.01 |  |
|  | Never | 0.40 | (0.36, 0.43) | 0.02 |  |
| Education | Up to 8 years | 0.35 | (0.27, 0.44) | 0.04 | < .001 |
|  | 9-15 years | 0.32 | (0.30, 0.34) | 0.01 |  |
|  | 16+ years | 0.25 | (0.23, 0.28) | 0.01 |  |
| Immigration status | Born in this country | 0.30 | (0.28, 0.32) | 0.01 | < .001 |
|  | Born in another country | 0.54 | (0.41, 0.67) | 0.06 |  |
| Religious affiliation | Christianity | 0.30 | (0.28, 0.32) | 0.01 | < .001 |
|  | Islam | 0.00 | * | * |  |
|  | Buddhism | 0.00 | * | * |  |
|  | Sikhism | 1.00 | * | * |  |
|  | Jainism | 0.00 | * | * |  |
|  | Shinto | 1.00 | * | * |  |
|  | Primal, Animist, or Folk religion | 0.34 | (0.00, 1.00) | 0.15 |  |
|  | No religion/Atheist/Agnostic | 0.38 | (0.33, 0.43) | 0.03 |  |
| Race/ethnicity | Polish | 0.30 | (0.28, 0.32) | 0.01 | < .001 |
|  | German | 0.00 | * | * |  |
|  | Belarussian | 0.63 | * | * |  |
|  | Ukrainian | 0.69 | (0.51, 0.87) | 0.09 |  |
|  | Silesia | 0.36 | (0.08, 0.64) | 0.11 |  |
|  | Kashubians | 0.32 | * | * |  |
|  | Other | 0.13 | (0.00, 0.83) | 0.25 |  |

# Table S63. Proportions by demographic category for South Africa

| Variable | Category | Proportion | 95% CI | SE | Global p-value |
| --- | --- | --- | --- | --- | --- |
| Age group | 18-24 | 0.20 | (0.14, 0.25) | 0.03 | < .001 |
|  | 25-34 | 0.27 | (0.23, 0.30) | 0.02 |  |
|  | 35-44 | 0.29 | (0.24, 0.33) | 0.02 |  |
|  | 45-54 | 0.24 | (0.18, 0.31) | 0.03 |  |
|  | 55-64 | 0.23 | (0.14, 0.32) | 0.05 |  |
|  | 65-74 | 0.10 | (0.01, 0.19) | 0.05 |  |
|  | 75-84 | 0.25 | (0.03, 0.48) | 0.11 |  |
|  | 85 or older | 0.00 | * | * |  |
| Gender | Male | 0.38 | (0.34, 0.42) | 0.02 | < .001 |
|  | Female | 0.11 | (0.09, 0.14) | 0.01 |  |
|  | Other | 0.71 | * | * |  |
| Marital status | Married | 0.26 | (0.19, 0.34) | 0.04 | < .001 |
|  | Separated | 0.48 | (0.34, 0.63) | 0.07 |  |
|  | Divorced | 0.34 | (0.17, 0.52) | 0.09 |  |
|  | Widowed | 0.10 | (0.03, 0.17) | 0.04 |  |
|  | Never | 0.22 | (0.19, 0.25) | 0.02 |  |
|  | Domestic Partner | 0.32 | (0.25, 0.39) | 0.04 |  |
| Employment | Employed for an employer | 0.27 | (0.21, 0.33) | 0.03 | 0.199 |
|  | Self-employed | 0.28 | (0.22, 0.34) | 0.03 |  |
|  | Retired | 0.19 | (0.09, 0.28) | 0.05 |  |
|  | Student | 0.20 | (0.11, 0.29) | 0.05 |  |
|  | Homemaker | 0.16 | (0.07, 0.26) | 0.05 |  |
|  | Unemployed and looking for a job | 0.25 | (0.22, 0.28) | 0.02 |  |
|  | None of these/Other | 0.19 | (0.03, 0.36) | 0.08 |  |
| Religious service attendance | More than 1/week | 0.15 | (0.09, 0.20) | 0.03 | < .001 |
|  | 1/week | 0.20 | (0.16, 0.25) | 0.02 |  |
|  | 1-3/month | 0.24 | (0.19, 0.30) | 0.03 |  |
|  | A few times a year | 0.32 | (0.26, 0.38) | 0.03 |  |
|  | Never | 0.35 | (0.29, 0.42) | 0.03 |  |
| Education | Up to 8 years | 0.17 | (0.11, 0.22) | 0.03 | 0.008 |
|  | 9-15 years | 0.27 | (0.24, 0.30) | 0.02 |  |
|  | 16+ years | 0.23 | (0.16, 0.29) | 0.03 |  |
| Immigration status | Born in this country | 0.25 | (0.22, 0.28) | 0.02 | 0.030 |
|  | Born in another country | 0.14 | (0.05, 0.23) | 0.05 |  |
| Religious affiliation | Christianity | 0.22 | (0.19, 0.25) | 0.02 | < .001 |
|  | Islam | 0.51 | (0.29, 0.73) | 0.11 |  |
|  | Hinduism | 0.00 | * | * |  |
|  | Buddhism | 0.22 | (0.00, 1.00) | 0.15 |  |
|  | Jainism | 1.00 | * | * |  |
|  | Shinto | 0.00 | * | * |  |
|  | Taoism | 0.00 | * | * |  |
|  | Primal, Animist, or Folk religion | 0.22 | (0.14, 0.30) | 0.04 |  |
|  | Some other religion | 0.96 | * | * |  |
|  | No religion/Atheist/Agnostic | 0.34 | (0.27, 0.40) | 0.03 |  |
| Race/ethnicity | Black | 0.21 | (0.19, 0.24) | 0.01 | < .001 |
|  | White | 0.47 | (0.00, 1.00) | 0.09 |  |
|  | Asian/Indian | 0.00 | * | * |  |
|  | Colored | 0.51 | (0.34, 0.67) | 0.08 |  |
|  | Other | 0.00 | * | * |  |

# Table S64. Proportions by demographic category for Spain

| Variable | Category | Proportion | 95% CI | SE | Global p-value |
| --- | --- | --- | --- | --- | --- |
| Age group | 18-24 | 0.27 | (0.22, 0.32) | 0.02 | < .001 |
|  | 25-34 | 0.33 | (0.30, 0.37) | 0.02 |  |
|  | 35-44 | 0.38 | (0.35, 0.41) | 0.01 |  |
|  | 45-54 | 0.35 | (0.32, 0.38) | 0.01 |  |
|  | 55-64 | 0.32 | (0.28, 0.36) | 0.02 |  |
|  | 65-74 | 0.26 | (0.20, 0.31) | 0.03 |  |
|  | 75-84 | 0.20 | (0.10, 0.30) | 0.05 |  |
|  | 85 or older | 0.67 | * | * |  |
| Gender | Male | 0.35 | (0.33, 0.37) | 0.01 | < .001 |
|  | Female | 0.30 | (0.27, 0.32) | 0.01 |  |
|  | Other | 0.49 | (0.00, 1.00) | 0.18 |  |
| Marital status | Married | 0.30 | (0.28, 0.32) | 0.01 | 0.031 |
|  | Separated | 0.40 | (0.32, 0.49) | 0.04 |  |
|  | Divorced | 0.37 | (0.32, 0.43) | 0.03 |  |
|  | Widowed | 0.33 | (0.23, 0.44) | 0.05 |  |
|  | Never | 0.33 | (0.30, 0.36) | 0.01 |  |
|  | Domestic Partner | 0.34 | (0.30, 0.39) | 0.02 |  |
| Employment | Employed for an employer | 0.34 | (0.32, 0.36) | 0.01 | < .001 |
|  | Self-employed | 0.35 | (0.30, 0.39) | 0.02 |  |
|  | Retired | 0.29 | (0.25, 0.33) | 0.02 |  |
|  | Student | 0.23 | (0.18, 0.28) | 0.03 |  |
|  | Homemaker | 0.30 | (0.22, 0.37) | 0.04 |  |
|  | Unemployed and looking for a job | 0.37 | (0.33, 0.41) | 0.02 |  |
|  | None of these/Other | 0.30 | (0.22, 0.39) | 0.05 |  |
| Religious service attendance | More than 1/week | 0.25 | (0.19, 0.31) | 0.03 | 0.009 |
|  | 1/week | 0.39 | (0.33, 0.44) | 0.03 |  |
|  | 1-3/month | 0.36 | (0.30, 0.42) | 0.03 |  |
|  | A few times a year | 0.31 | (0.28, 0.34) | 0.01 |  |
|  | Never | 0.32 | (0.30, 0.34) | 0.01 |  |
| Education | Up to 8 years | 0.34 | (0.28, 0.39) | 0.03 | 0.010 |
|  | 9-15 years | 0.33 | (0.32, 0.35) | 0.01 |  |
|  | 16+ years | 0.28 | (0.25, 0.31) | 0.02 |  |
| Immigration status | Born in this country | 0.33 | (0.32, 0.35) | 0.01 | < .001 |
|  | Born in another country | 0.25 | (0.22, 0.29) | 0.02 |  |
| Religious affiliation | Christianity | 0.32 | (0.30, 0.34) | 0.01 | < .001 |
|  | Islam | 0.31 | (0.20, 0.41) | 0.05 |  |
|  | Hinduism | 0.44 | * | * |  |
|  | Buddhism | 0.37 | (0.12, 0.62) | 0.12 |  |
|  | Judaism | 1.00 | * | * |  |
|  | Sikhism | 1.00 | * | * |  |
|  | Baha'i | 0.40 | * | * |  |
|  | Jainism | 0.00 | * | * |  |
|  | Taoism | 0.16 | * | * |  |
|  | Confucianism | 1.00 | * | * |  |
|  | Primal, Animist, or Folk religion | 0.65 | * | * |  |
|  | Some other religion | 0.48 | (0.20, 0.77) | 0.13 |  |
|  | No religion/Atheist/Agnostic | 0.32 | (0.29, 0.34) | 0.01 |  |

# Table S65. Proportions by demographic category for Sweden

| Variable | Category | Proportion | 95% CI | SE | Global p-value |
| --- | --- | --- | --- | --- | --- |
| Age group | 18-24 | 0.09 | (0.07, 0.11) | 0.01 | < .001 |
|  | 25-34 | 0.11 | (0.10, 0.13) | 0.01 |  |
|  | 35-44 | 0.10 | (0.09, 0.12) | 0.01 |  |
|  | 45-54 | 0.10 | (0.08, 0.11) | 0.01 |  |
|  | 55-64 | 0.11 | (0.09, 0.12) | 0.01 |  |
|  | 65-74 | 0.10 | (0.08, 0.11) | 0.01 |  |
|  | 75-84 | 0.06 | (0.04, 0.07) | 0.01 |  |
|  | 85 or older | 0.01 | (0.00, 0.03) | 0.01 |  |
| Gender | Male | 0.09 | (0.08, 0.10) | 0.00 | 0.091 |
|  | Female | 0.10 | (0.09, 0.11) | 0.00 |  |
|  | Other | 0.16 | (0.00, 0.35) | 0.09 |  |
| Marital status | Married | 0.07 | (0.06, 0.08) | 0.00 | < .001 |
|  | Separated | 0.14 | (0.10, 0.18) | 0.02 |  |
|  | Divorced | 0.15 | (0.13, 0.18) | 0.01 |  |
|  | Widowed | 0.09 | (0.06, 0.12) | 0.02 |  |
|  | Never | 0.11 | (0.10, 0.13) | 0.01 |  |
|  | Domestic Partner | 0.11 | (0.10, 0.12) | 0.01 |  |
| Employment | Employed for an employer | 0.10 | (0.09, 0.10) | 0.00 | < .001 |
|  | Self-employed | 0.12 | (0.09, 0.15) | 0.01 |  |
|  | Retired | 0.08 | (0.07, 0.09) | 0.01 |  |
|  | Student | 0.06 | (0.05, 0.08) | 0.01 |  |
|  | Homemaker | 0.20 | (0.09, 0.32) | 0.06 |  |
|  | Unemployed and looking for a job | 0.23 | (0.18, 0.28) | 0.03 |  |
|  | None of these/Other | 0.17 | (0.12, 0.22) | 0.02 |  |
| Religious service attendance | More than 1/week | 0.10 | (0.04, 0.16) | 0.03 | 0.235 |
|  | 1/week | 0.13 | (0.09, 0.18) | 0.02 |  |
|  | 1-3/month | 0.11 | (0.07, 0.14) | 0.02 |  |
|  | A few times a year | 0.09 | (0.08, 0.10) | 0.01 |  |
|  | Never | 0.10 | (0.09, 0.10) | 0.00 |  |
| Education | Up to 8 years | 0.11 | (0.06, 0.16) | 0.02 | < .001 |
|  | 9-15 years | 0.11 | (0.10, 0.12) | 0.00 |  |
|  | 16+ years | 0.05 | (0.05, 0.06) | 0.00 |  |
| Immigration status | Born in this country | 0.09 | (0.09, 0.10) | 0.00 | < .001 |
|  | Born in another country | 0.15 | (0.12, 0.18) | 0.01 |  |
| Religious affiliation | Christianity | 0.09 | (0.08, 0.09) | 0.00 | < .001 |
|  | Islam | 0.24 | (0.18, 0.30) | 0.03 |  |
|  | Hinduism | 0.07 | (0.00, 0.35) | 0.09 |  |
|  | Buddhism | 0.12 | (0.03, 0.22) | 0.05 |  |
|  | Judaism | 0.14 | (0.00, 0.28) | 0.07 |  |
|  | Sikhism | 0.00 | * | * |  |
|  | Baha'i | 0.56 | * | * |  |
|  | Shinto | 1.00 | * | * |  |
|  | Taoism | 0.70 | * | * |  |
|  | Primal, Animist, or Folk religion | 0.14 | (0.02, 0.26) | 0.06 |  |
|  | Some other religion | 0.16 | (0.09, 0.24) | 0.04 |  |
|  | No religion/Atheist/Agnostic | 0.09 | (0.09, 0.10) | 0.00 |  |

# Table S66. Proportions by demographic category for Tanzania

| Variable | Category | Proportion | 95% CI | SE | Global p-value |
| --- | --- | --- | --- | --- | --- |
| Age group | 18-24 | 0.02 | (0.01, 0.03) | 0.01 | < .001 |
|  | 25-34 | 0.04 | (0.03, 0.05) | 0.01 |  |
|  | 35-44 | 0.05 | (0.04, 0.07) | 0.01 |  |
|  | 45-54 | 0.06 | (0.04, 0.08) | 0.01 |  |
|  | 55-64 | 0.07 | (0.04, 0.09) | 0.01 |  |
|  | 65-74 | 0.04 | (0.02, 0.07) | 0.01 |  |
|  | 75-84 | 0.02 | (0.00, 0.05) | 0.01 |  |
|  | 85 or older | 0.00 | * | * |  |
| Gender | Male | 0.08 | (0.07, 0.09) | 0.01 | < .001 |
|  | Female | 0.00 | (0.00, 0.01) | 0.00 |  |
| Marital status | Married | 0.05 | (0.04, 0.05) | 0.00 | < .001 |
|  | Separated | 0.08 | (0.05, 0.11) | 0.02 |  |
|  | Divorced | 0.04 | (0.00, 0.10) | 0.03 |  |
|  | Widowed | 0.02 | (0.00, 0.03) | 0.01 |  |
|  | Never | 0.03 | (0.02, 0.04) | 0.01 |  |
|  | Domestic Partner | 0.05 | (0.01, 0.08) | 0.02 |  |
| Employment | Employed for an employer | 0.04 | (0.02, 0.05) | 0.01 | < .001 |
|  | Self-employed | 0.06 | (0.05, 0.07) | 0.00 |  |
|  | Retired | 0.04 | (0.01, 0.07) | 0.01 |  |
|  | Student | 0.01 | (0.00, 0.02) | 0.01 |  |
|  | Homemaker | 0.00 | (0.00, 0.01) | 0.00 |  |
|  | Unemployed and looking for a job | 0.04 | (0.03, 0.05) | 0.01 |  |
|  | None of these/Other | 0.09 | (0.03, 0.16) | 0.03 |  |
| Religious service attendance | More than 1/week | 0.03 | (0.02, 0.04) | 0.00 | 0.002 |
|  | 1/week | 0.03 | (0.03, 0.04) | 0.00 |  |
|  | 1-3/month | 0.06 | (0.04, 0.08) | 0.01 |  |
|  | A few times a year | 0.06 | (0.04, 0.08) | 0.01 |  |
|  | Never | 0.09 | (0.05, 0.14) | 0.02 |  |
| Education | Up to 8 years | 0.05 | (0.04, 0.05) | 0.00 | < .001 |
|  | 9-15 years | 0.03 | (0.02, 0.03) | 0.00 |  |
|  | 16+ years | 0.02 | (0.00, 0.05) | 0.01 |  |
| Immigration status | Born in this country | 0.04 | (0.04, 0.05) | 0.00 | 0.389 |
|  | Born in another country | 0.02 | (0.00, 0.07) | 0.02 |  |
| Religious affiliation | Christianity | 0.03 | (0.03, 0.04) | 0.00 | 0.271 |
|  | Islam | 0.06 | (0.05, 0.07) | 0.01 |  |
|  | Taoism | 0.00 | * | * |  |
|  | Primal, Animist, or Folk religion | 0.00 | * | * |  |
|  | No religion/Atheist/Agnostic | 0.05 | (0.02, 0.09) | 0.02 |  |
| Race/ethnicity | Indian | 0.00 | * | * | 0.977 |
|  | Arab | 0.00 | * | * |  |
|  | African | 0.04 | (0.04, 0.05) | 0.00 |  |

# Table S67. Proportions by demographic category for Türkiye

| Variable | Category | Proportion | 95% CI | SE | Global p-value |
| --- | --- | --- | --- | --- | --- |
| Age group | 18-24 | 0.52 | (0.45, 0.59) | 0.03 | < .001 |
|  | 25-34 | 0.58 | (0.52, 0.64) | 0.03 |  |
|  | 35-44 | 0.55 | (0.49, 0.60) | 0.03 |  |
|  | 45-54 | 0.57 | (0.50, 0.64) | 0.04 |  |
|  | 55-64 | 0.53 | (0.43, 0.63) | 0.05 |  |
|  | 65-74 | 0.40 | (0.25, 0.54) | 0.07 |  |
|  | 75-84 | 0.24 | (0.01, 0.47) | 0.11 |  |
|  | 85 or older | 1.00 | * | * |  |
| Gender | Male | 0.63 | (0.59, 0.67) | 0.02 | < .001 |
|  | Female | 0.43 | (0.38, 0.48) | 0.02 |  |
| Marital status | Married | 0.50 | (0.46, 0.54) | 0.02 | < .001 |
|  | Separated | 0.51 | (0.12, 0.90) | 0.16 |  |
|  | Divorced | 0.71 | (0.58, 0.85) | 0.07 |  |
|  | Widowed | 0.31 | (0.13, 0.48) | 0.09 |  |
|  | Never | 0.61 | (0.57, 0.66) | 0.02 |  |
| Employment | Employed for an employer | 0.60 | (0.55, 0.65) | 0.02 | < .001 |
|  | Self-employed | 0.70 | (0.63, 0.76) | 0.03 |  |
|  | Retired | 0.47 | (0.37, 0.57) | 0.05 |  |
|  | Student | 0.41 | (0.33, 0.50) | 0.04 |  |
|  | Homemaker | 0.36 | (0.29, 0.43) | 0.04 |  |
|  | Unemployed and looking for a job | 0.68 | (0.57, 0.78) | 0.05 |  |
|  | None of these/Other | 0.55 | (0.40, 0.70) | 0.08 |  |
| Religious service attendance | More than 1/week | 0.46 | (0.41, 0.52) | 0.03 | 0.012 |
|  | 1/week | 0.56 | (0.49, 0.63) | 0.04 |  |
|  | 1-3/month | 0.54 | (0.44, 0.63) | 0.05 |  |
|  | A few times a year | 0.62 | (0.55, 0.69) | 0.03 |  |
|  | Never | 0.55 | (0.48, 0.62) | 0.04 |  |
| Education | Up to 8 years | 0.50 | (0.43, 0.57) | 0.04 | 0.216 |
|  | 9-15 years | 0.56 | (0.52, 0.60) | 0.02 |  |
|  | 16+ years | 0.51 | (0.47, 0.56) | 0.02 |  |
| Immigration status | Born in this country | 0.54 | (0.50, 0.57) | 0.02 | 0.541 |
|  | Born in another country | 0.48 | (0.32, 0.65) | 0.08 |  |
| Religious affiliation | Christianity | 0.55 | * | * | 0.040 |
|  | Islam | 0.53 | (0.49, 0.56) | 0.02 |  |
|  | Buddhism | 0.00 | * | * |  |
|  | Judaism | 1.00 | * | * |  |
|  | Sikhism | 0.00 | * | * |  |
|  | Primal, Animist, or Folk religion | 0.40 | * | * |  |
|  | Some other religion | 0.94 | * | * |  |
|  | No religion/Atheist/Agnostic | 0.66 | (0.55, 0.77) | 0.06 |  |
| Race/ethnicity | Arab | 0.39 | (0.22, 0.56) | 0.09 | < .001 |
|  | Turkish | 0.52 | (0.48, 0.56) | 0.02 |  |
|  | Greek | 1.00 | * | * |  |
|  | Kurdish/Zaza | 0.58 | (0.50, 0.65) | 0.04 |  |
|  | Laz | 0.44 | (0.19, 0.70) | 0.11 |  |
|  | Circassian | 0.82 | (0.58, 1.00) | 0.09 |  |
|  | Bosnian | 0.67 | * | * |  |
|  | Armenian | 0.00 | * | * |  |
|  | Georgian | 1.00 | * | * |  |
|  | Uyghur | 1.00 | * | * |  |
|  | Albanian | 0.88 | * | * |  |
|  | Azeri | 0.89 | * | * |  |
|  | Other | 0.51 | (0.33, 0.69) | 0.09 |  |

# Table S68. Proportions by demographic category for United Kingdom

| Variable | Category | Proportion | 95% CI | SE | Global p-value |
| --- | --- | --- | --- | --- | --- |
| Age group | 18-24 | 0.07 | (0.04, 0.11) | 0.02 | < .001 |
|  | 25-34 | 0.24 | (0.20, 0.27) | 0.02 |  |
|  | 35-44 | 0.21 | (0.17, 0.24) | 0.02 |  |
|  | 45-54 | 0.17 | (0.14, 0.21) | 0.02 |  |
|  | 55-64 | 0.16 | (0.13, 0.20) | 0.02 |  |
|  | 65-74 | 0.14 | (0.11, 0.17) | 0.02 |  |
|  | 75-84 | 0.06 | (0.03, 0.10) | 0.02 |  |
|  | 85 or older | 0.02 | (0.00, 0.05) | 0.02 |  |
| Gender | Male | 0.20 | (0.17, 0.22) | 0.01 | < .001 |
|  | Female | 0.13 | (0.12, 0.15) | 0.01 |  |
|  | Other | 0.10 | (0.00, 0.26) | 0.07 |  |
| Marital status | Married | 0.16 | (0.14, 0.18) | 0.01 | 0.111 |
|  | Separated | 0.10 | (0.05, 0.16) | 0.03 |  |
|  | Divorced | 0.18 | (0.14, 0.23) | 0.02 |  |
|  | Widowed | 0.14 | (0.09, 0.20) | 0.03 |  |
|  | Never | 0.16 | (0.13, 0.18) | 0.01 |  |
|  | Domestic Partner | 0.21 | (0.16, 0.26) | 0.03 |  |
| Employment | Employed for an employer | 0.18 | (0.16, 0.20) | 0.01 | < .001 |
|  | Self-employed | 0.20 | (0.15, 0.25) | 0.03 |  |
|  | Retired | 0.11 | (0.08, 0.13) | 0.01 |  |
|  | Student | 0.06 | (0.02, 0.09) | 0.02 |  |
|  | Homemaker | 0.20 | (0.11, 0.30) | 0.05 |  |
|  | Unemployed and looking for a job | 0.18 | (0.11, 0.26) | 0.04 |  |
|  | None of these/Other | 0.23 | (0.16, 0.30) | 0.04 |  |
| Religious service attendance | More than 1/week | 0.21 | (0.15, 0.28) | 0.03 | < .001 |
|  | 1/week | 0.28 | (0.22, 0.33) | 0.03 |  |
|  | 1-3/month | 0.21 | (0.15, 0.27) | 0.03 |  |
|  | A few times a year | 0.10 | (0.08, 0.12) | 0.01 |  |
|  | Never | 0.16 | (0.14, 0.18) | 0.01 |  |
| Education | Up to 8 years | 0.15 | (0.11, 0.19) | 0.02 | 0.469 |
|  | 9-15 years | 0.17 | (0.15, 0.19) | 0.01 |  |
|  | 16+ years | 0.16 | (0.15, 0.18) | 0.01 |  |
| Immigration status | Born in this country | 0.17 | (0.15, 0.18) | 0.01 | 0.281 |
|  | Born in another country | 0.14 | (0.11, 0.18) | 0.02 |  |
| Religious affiliation | Christianity | 0.17 | (0.15, 0.19) | 0.01 | < .001 |
|  | Islam | 0.15 | (0.08, 0.22) | 0.03 |  |
|  | Hinduism | 0.10 | (0.02, 0.17) | 0.04 |  |
|  | Buddhism | 0.39 | (0.13, 0.65) | 0.12 |  |
|  | Judaism | 0.05 | (0.00, 0.11) | 0.03 |  |
|  | Sikhism | 0.13 | (0.00, 0.33) | 0.09 |  |
|  | Baha'i | 0.88 | * | * |  |
|  | Jainism | 1.00 | * | * |  |
|  | Taoism | 0.12 | * | * |  |
|  | Confucianism | 0.00 | * | * |  |
|  | Primal, Animist, or Folk religion | 0.24 | (0.00, 0.51) | 0.12 |  |
|  | Some other religion | 0.21 | (0.08, 0.35) | 0.07 |  |
|  | No religion/Atheist/Agnostic | 0.16 | (0.14, 0.18) | 0.01 |  |
| Race/ethnicity | Asian | 0.14 | (0.09, 0.18) | 0.02 | 0.581 |
|  | Black | 0.16 | (0.08, 0.24) | 0.04 |  |
|  | White | 0.17 | (0.15, 0.18) | 0.01 |  |
|  | Other | 0.21 | (0.09, 0.33) | 0.06 |  |

# Table S69. Proportions by demographic category for United States

| Variable | Category | Proportion | 95% CI | SE | Global p-value |
| --- | --- | --- | --- | --- | --- |
| Age group | 18-24 | 0.06 | (0.02, 0.10) | 0.02 | < .001 |
|  | 25-34 | 0.11 | (0.08, 0.14) | 0.01 |  |
|  | 35-44 | 0.13 | (0.11, 0.15) | 0.01 |  |
|  | 45-54 | 0.11 | (0.09, 0.12) | 0.01 |  |
|  | 55-64 | 0.12 | (0.11, 0.14) | 0.01 |  |
|  | 65-74 | 0.09 | (0.08, 0.10) | 0.01 |  |
|  | 75-84 | 0.04 | (0.03, 0.05) | 0.00 |  |
|  | 85 or older | 0.01 | (0.00, 0.04) | 0.01 |  |
| Gender | Male | 0.10 | (0.09, 0.12) | 0.01 | 0.894 |
|  | Female | 0.10 | (0.09, 0.12) | 0.01 |  |
|  | Other | 0.08 | (0.00, 0.19) | 0.06 |  |
| Marital status | Married | 0.08 | (0.07, 0.08) | 0.00 | < .001 |
|  | Separated | 0.23 | (0.14, 0.33) | 0.05 |  |
|  | Divorced | 0.14 | (0.12, 0.16) | 0.01 |  |
|  | Widowed | 0.10 | (0.08, 0.12) | 0.01 |  |
|  | Never | 0.12 | (0.10, 0.15) | 0.01 |  |
|  | Domestic Partner | 0.19 | (0.13, 0.24) | 0.03 |  |
| Employment | Employed for an employer | 0.10 | (0.09, 0.11) | 0.01 | < .001 |
|  | Self-employed | 0.13 | (0.10, 0.17) | 0.02 |  |
|  | Retired | 0.08 | (0.07, 0.09) | 0.00 |  |
|  | Student | 0.07 | (0.02, 0.13) | 0.03 |  |
|  | Homemaker | 0.11 | (0.07, 0.15) | 0.02 |  |
|  | Unemployed and looking for a job | 0.15 | (0.10, 0.21) | 0.03 |  |
|  | None of these/Other | 0.23 | (0.16, 0.31) | 0.04 |  |
| Religious service attendance | More than 1/week | 0.05 | (0.03, 0.08) | 0.01 | < .001 |
|  | 1/week | 0.05 | (0.04, 0.06) | 0.01 |  |
|  | 1-3/month | 0.09 | (0.06, 0.13) | 0.02 |  |
|  | A few times a year | 0.11 | (0.09, 0.12) | 0.01 |  |
|  | Never | 0.13 | (0.12, 0.14) | 0.01 |  |
| Education | Up to 8 years | 0.16 | (0.00, 0.34) | 0.09 | < .001 |
|  | 9-15 years | 0.14 | (0.12, 0.15) | 0.01 |  |
|  | 16+ years | 0.04 | (0.03, 0.04) | 0.00 |  |
| Immigration status | Born in this country | 0.11 | (0.10, 0.12) | 0.00 | < .001 |
|  | Born in another country | 0.03 | (0.02, 0.04) | 0.01 |  |
| Religious affiliation | Christianity | 0.10 | (0.09, 0.11) | 0.00 | < .001 |
|  | Islam | 0.11 | (0.00, 0.25) | 0.07 |  |
|  | Hinduism | 0.02 | (0.00, 0.05) | 0.01 |  |
|  | Buddhism | 0.10 | (0.01, 0.18) | 0.04 |  |
|  | Judaism | 0.04 | (0.02, 0.06) | 0.01 |  |
|  | Sikhism | 0.00 | * | * |  |
|  | Baha'i | 0.27 | * | * |  |
|  | Jainism | 0.00 | * | * |  |
|  | Shinto | 0.00 | * | * |  |
|  | Taoism | 0.54 | (0.15, 0.92) | 0.19 |  |
|  | Confucianism | 0.00 | * | * |  |
|  | Primal, Animist, or Folk religion | 0.13 | (0.04, 0.22) | 0.05 |  |
|  | Some other religion | 0.20 | (0.11, 0.29) | 0.04 |  |
|  | No religion/Atheist/Agnostic | 0.11 | (0.09, 0.12) | 0.01 |  |
| Race/ethnicity | Asian | 0.04 | (0.02, 0.06) | 0.01 | < .001 |
|  | Black | 0.14 | (0.10, 0.17) | 0.02 |  |
|  | White | 0.10 | (0.09, 0.11) | 0.00 |  |
|  | Other | 0.15 | (0.10, 0.20) | 0.02 |  |
|  | Hispanic | 0.11 | (0.08, 0.14) | 0.02 |  |

# Table S70. Population weighted meta-analysis of proportion (prevalence) of daily cigarette smoking by demographic category

| Variable | Category | Proportion | 95% CI | SE Analogue |
| --- | --- | --- | --- | --- |
| Age group |  |  |  |  |
|  | 18-24 | 0.10 | (0.08,0.11) | 0.01 |
|  | 25-34 | 0.14 | (0.12,0.16) | 0.01 |
|  | 35-44 | 0.15 | (0.13,0.17) | 0.01 |
|  | 45-54 | 0.16 | (0.14,0.18) | 0.01 |
|  | 55-64 | 0.15 | (0.13,0.17) | 0.01 |
|  | 65-74 | 0.11 | (0.10,0.13) | 0.01 |
|  | 75-84 | 0.05 | (0.04,0.06) | 0.01 |
|  | 85 or older | 0.00 | (0.00,0.01) | 0.00 |
| Gender |  |  |  |  |
|  | Male | 0.22 | (0.19,0.24) | 0.01 |
|  | Female | 0.04 | (0.03,0.05) | 0.00 |
|  | Other | 0.10 | (0.08,0.12) | 0.01 |
| Marital status |  |  |  |  |
|  | Married | 0.13 | (0.11,0.14) | 0.01 |
|  | Separated | 0.19 | (0.17,0.21) | 0.01 |
|  | Divorced | 0.12 | (0.11,0.14) | 0.01 |
|  | Widowed | 0.09 | (0.07,0.10) | 0.01 |
|  | Domestic partner | 0.18 | (0.16,0.20) | 0.01 |
|  | Single, never married | 0.14 | (0.12,0.16) | 0.01 |
| Employment status |  |  |  |  |
|  | Employed for an employer | 0.18 | (0.16,0.20) | 0.01 |
|  | Self-employed | 0.18 | (0.16,0.20) | 0.01 |
|  | Retired | 0.16 | (0.14,0.18) | 0.01 |
|  | Student | 0.08 | (0.07,0.09) | 0.01 |
|  | Homemaker | 0.05 | (0.04,0.06) | 0.01 |
|  | Unemployed and looking for a job | 0.17 | (0.15,0.20) | 0.01 |
|  | None of these/other | 0.19 | (0.17,0.21) | 0.01 |
| Education |  |  |  |  |
|  | Up to 8 years | 0.15 | (0.13,0.17) | 0.01 |
|  | 9-15 years | 0.14 | (0.12,0.16) | 0.01 |
|  | 16+ years | 0.07 | (0.06,0.09) | 0.01 |
| Religious service attendance |  |  |  |  |
|  | >1/week | 0.11 | (0.10,0.13) | 0.01 |
|  | 1/week | 0.13 | (0.11,0.15) | 0.01 |
|  | 1-3/month | 0.14 | (0.12,0.16) | 0.01 |
|  | A few times a year | 0.14 | (0.13,0.16) | 0.01 |
|  | Never | 0.15 | (0.13,0.17) | 0.01 |
| Immigration status |  |  |  |  |
|  | Born in this country | 0.14 | (0.12,0.16) | 0.01 |
|  | Born in another country | 0.16 | (0.15,0.18) | 0.01 |

# Figures S1-S34. Forest plots of Mean for Demographic Categories


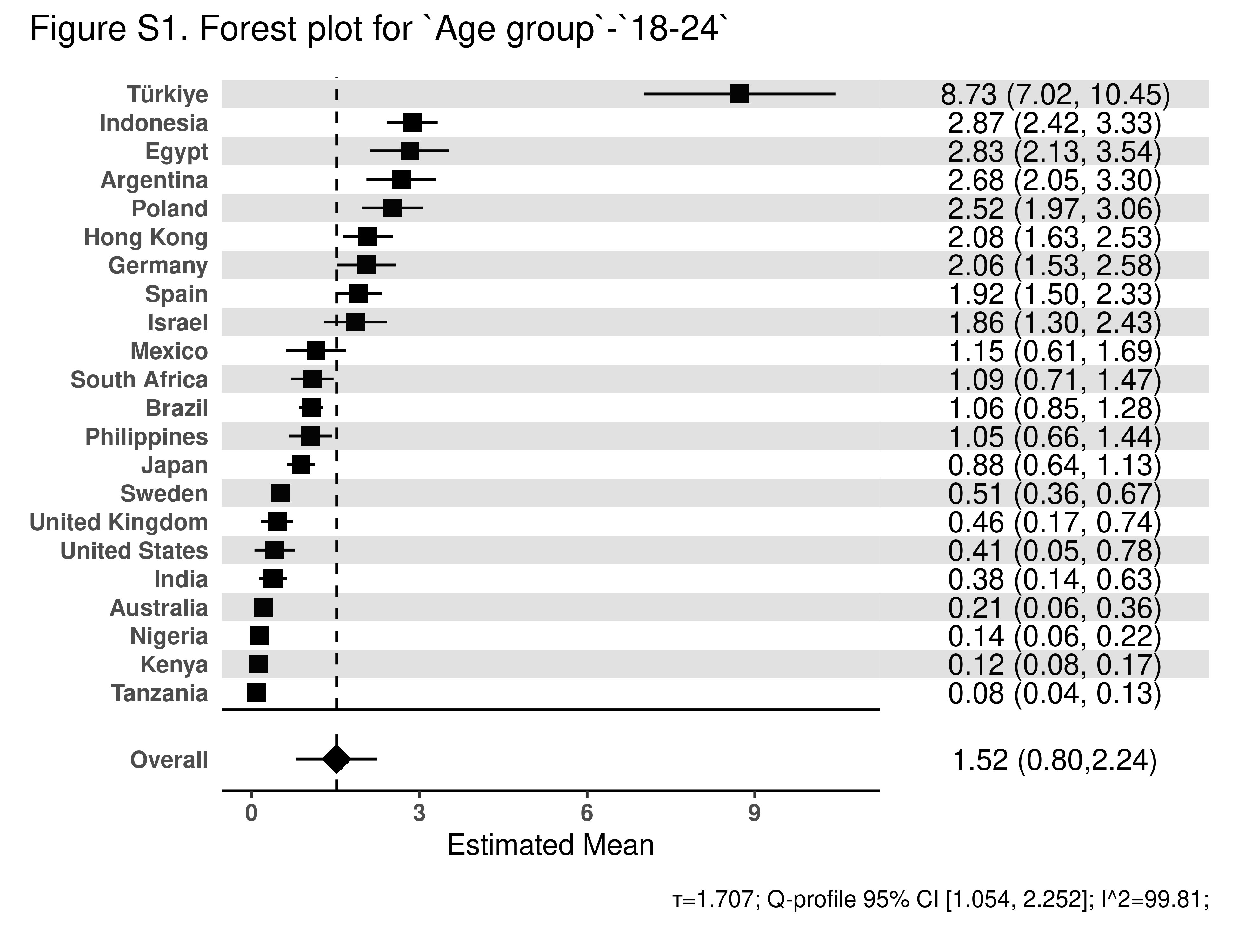

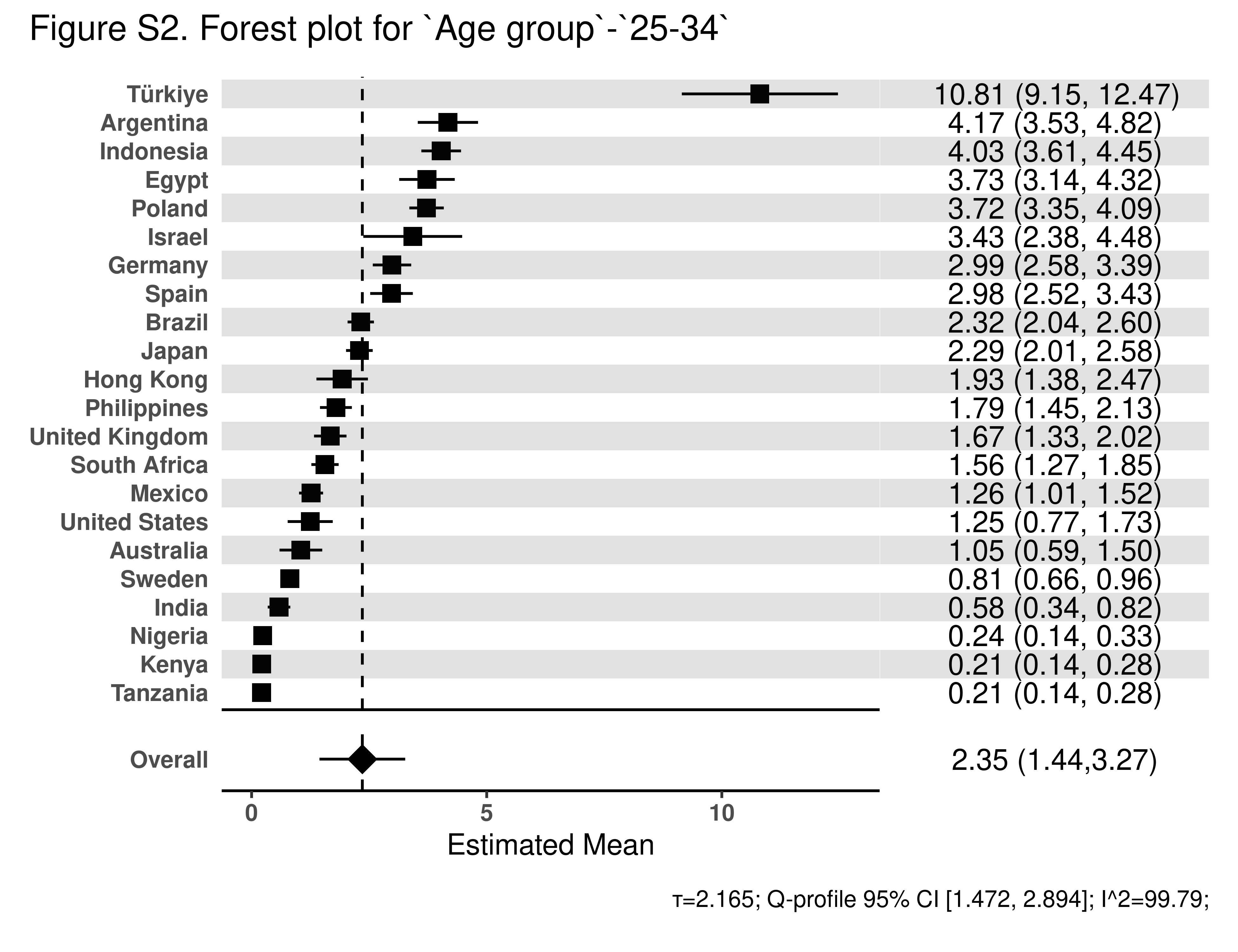

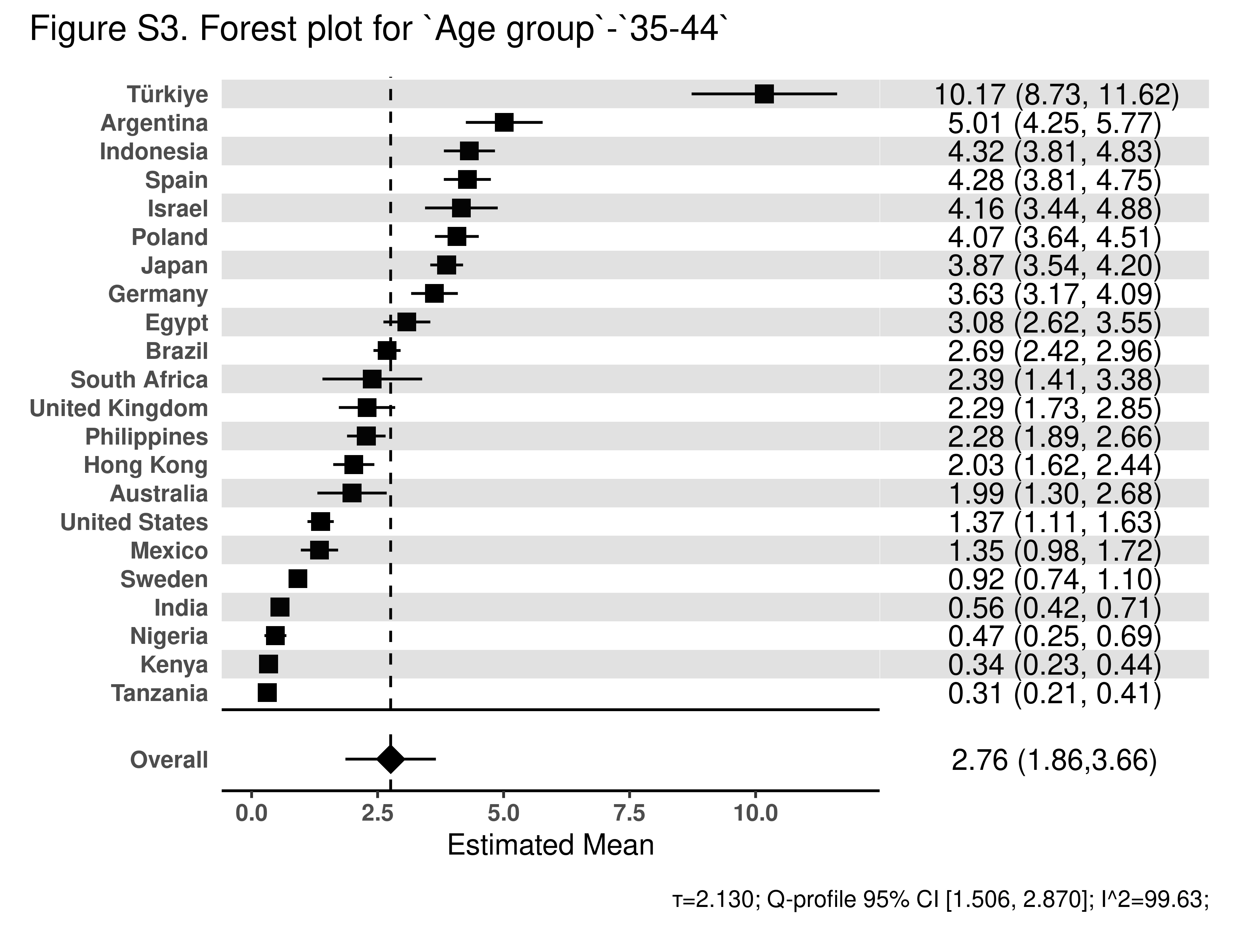

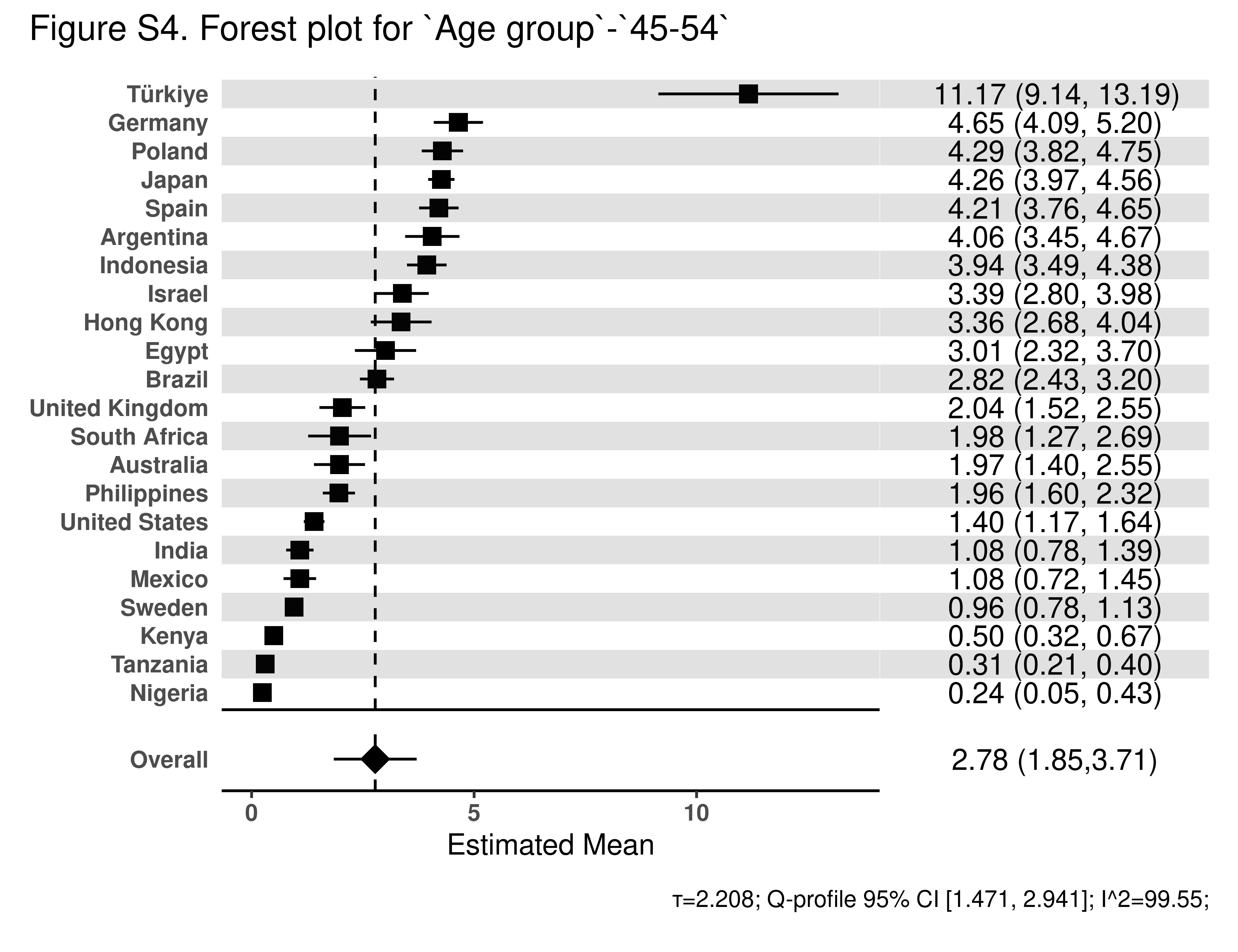

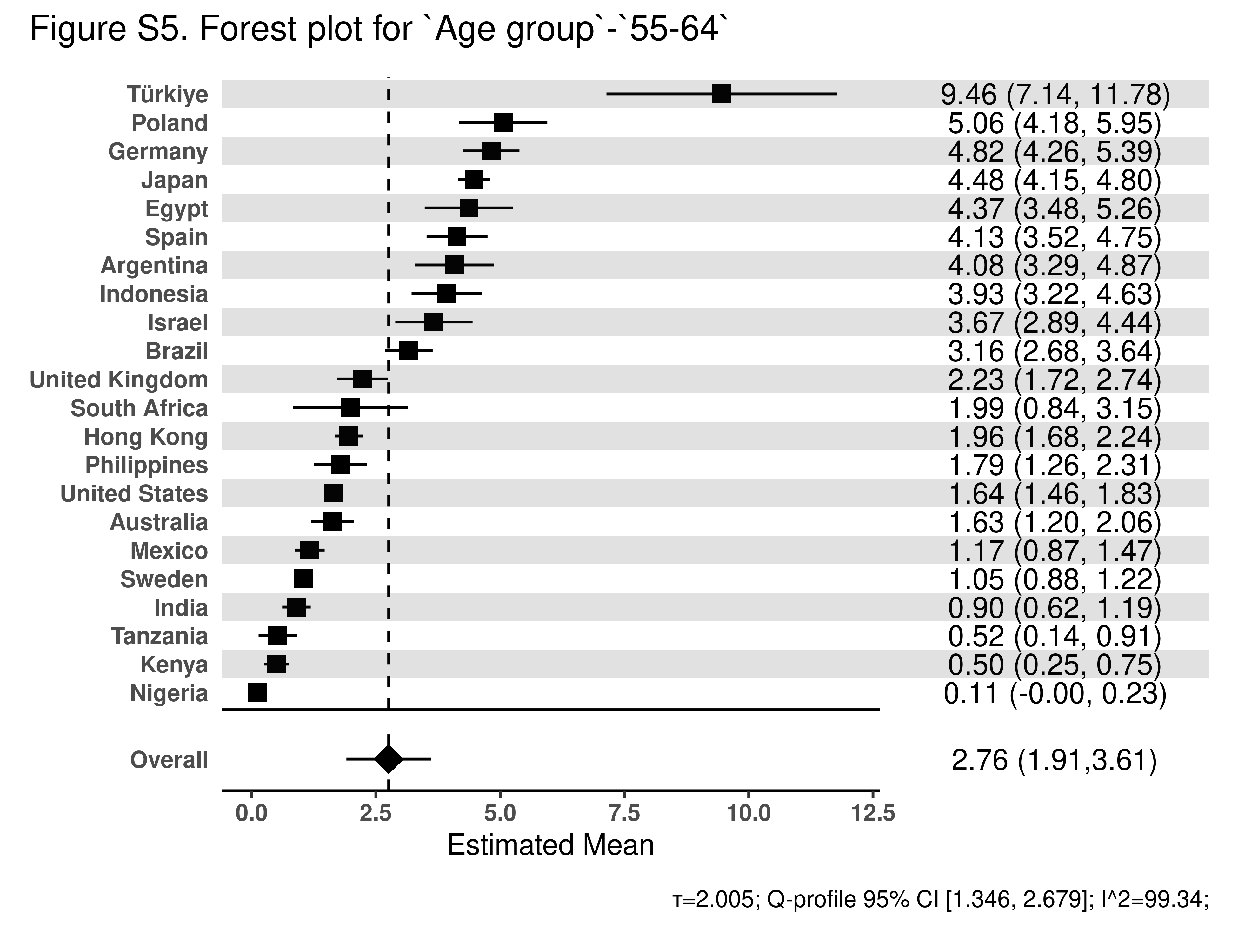

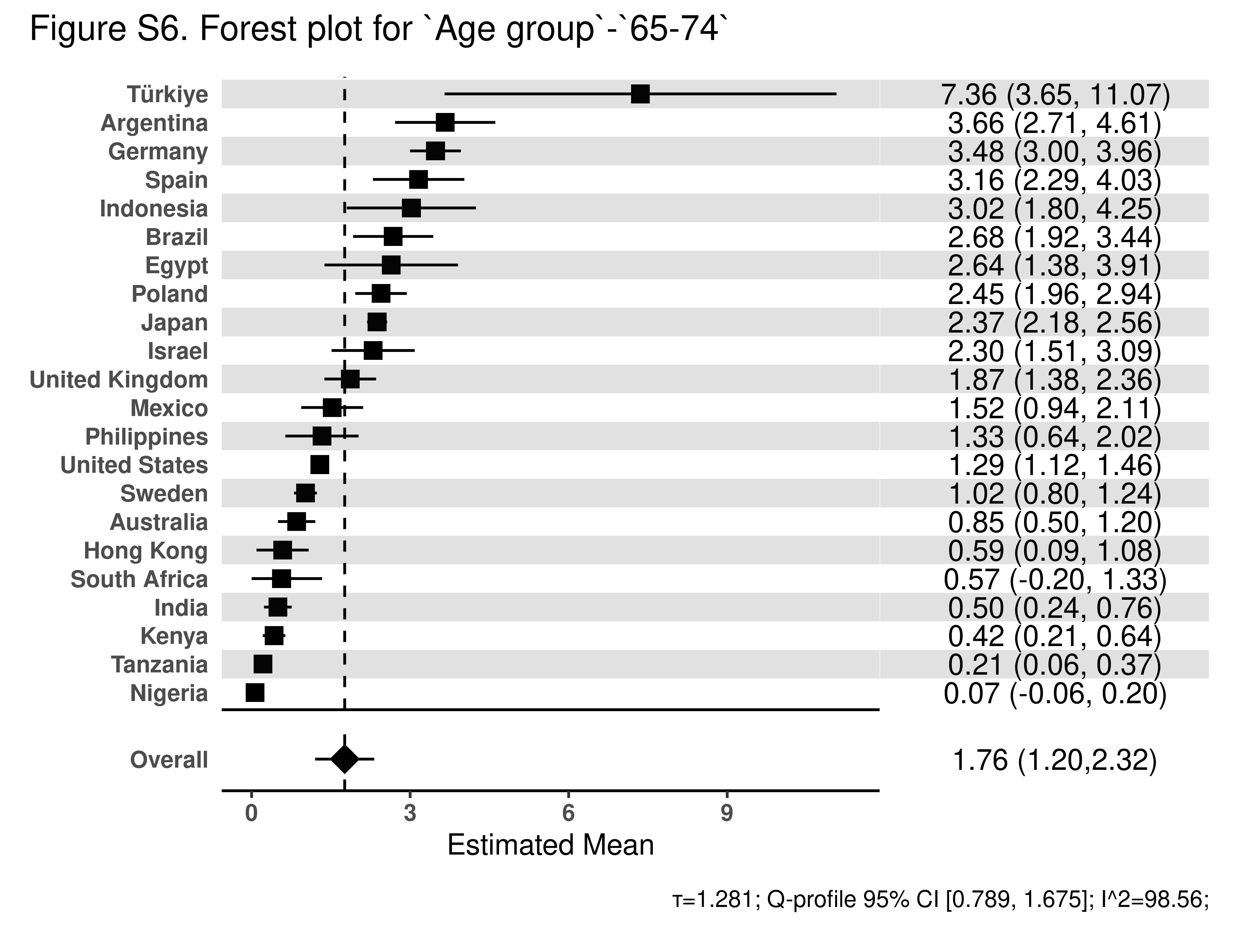

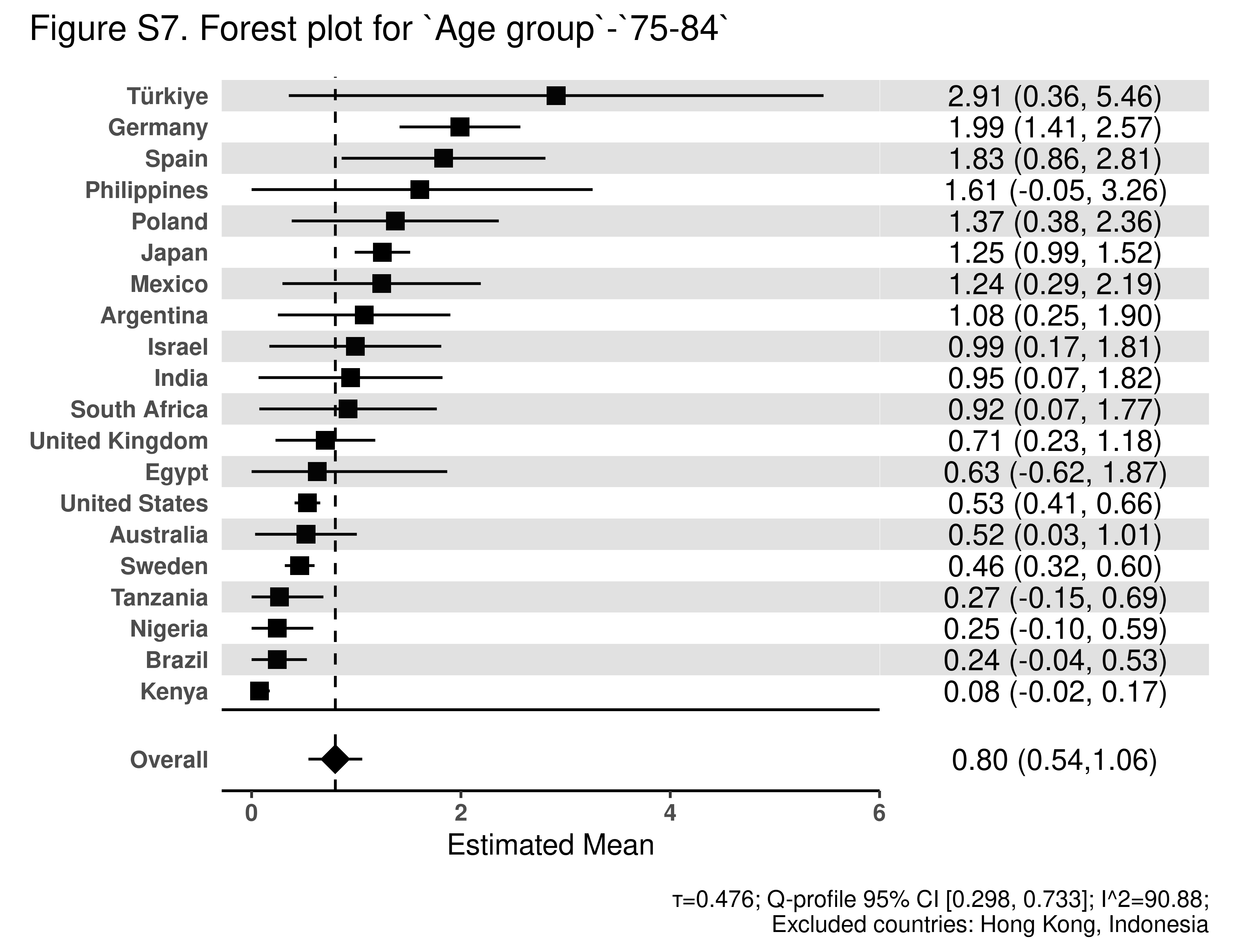

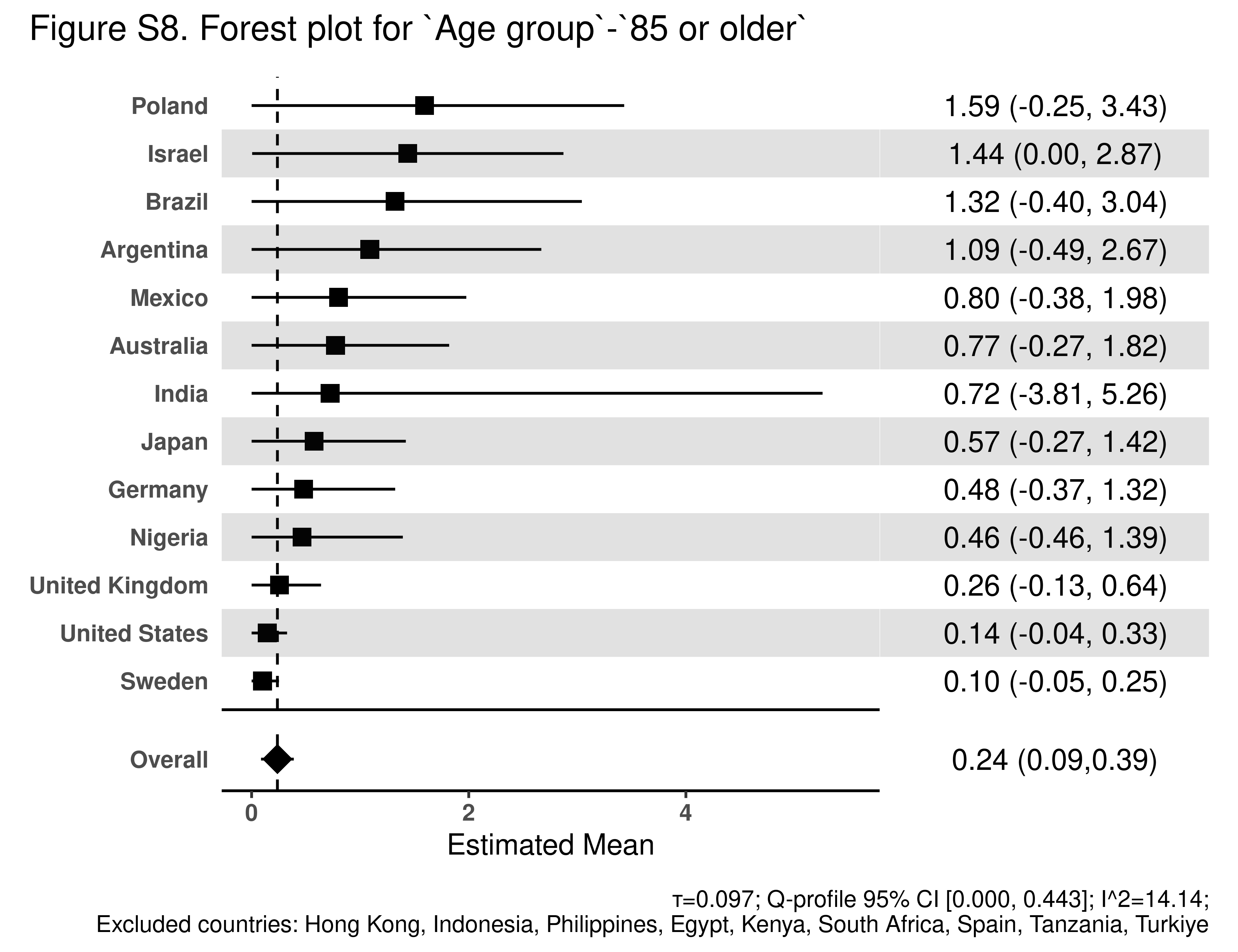

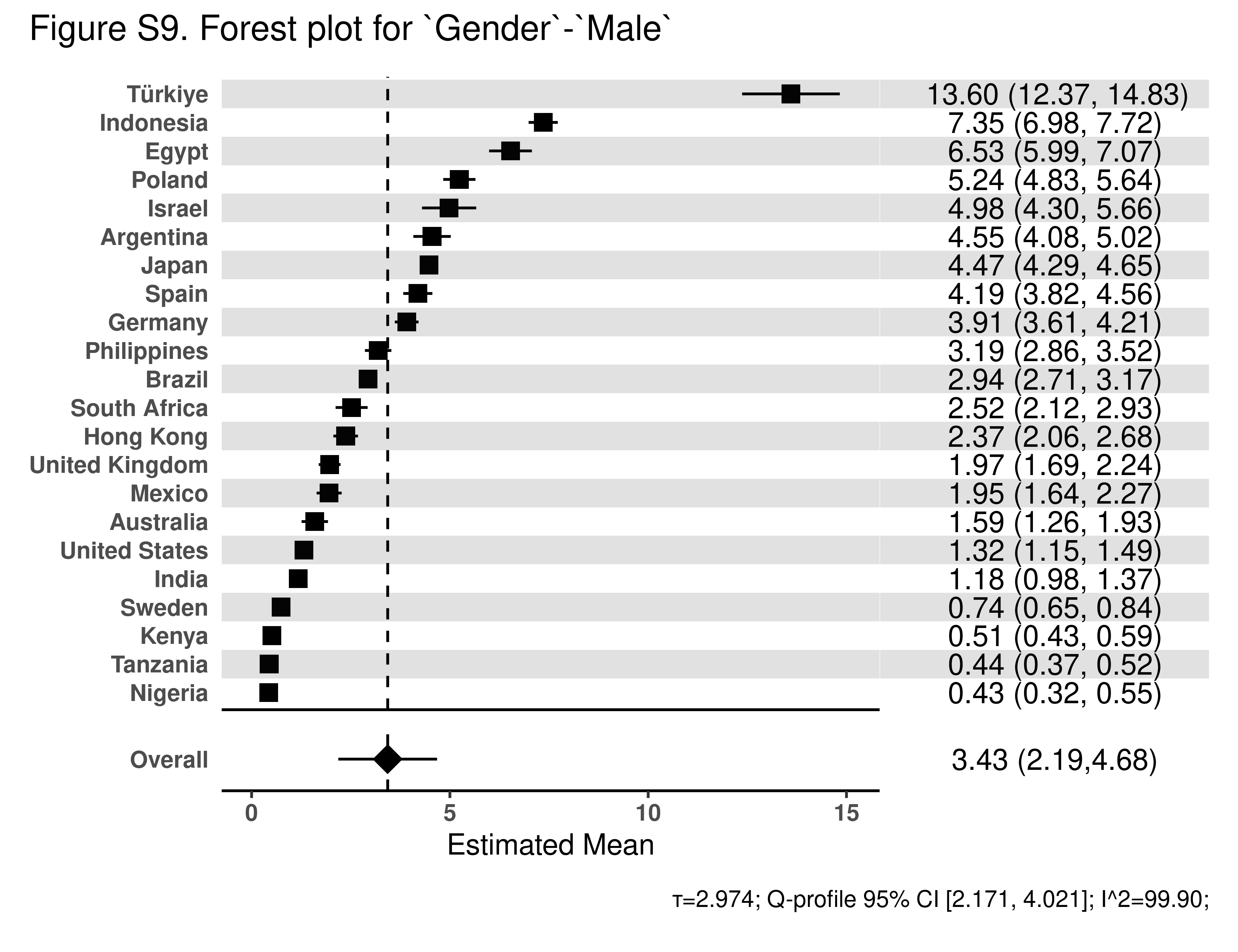

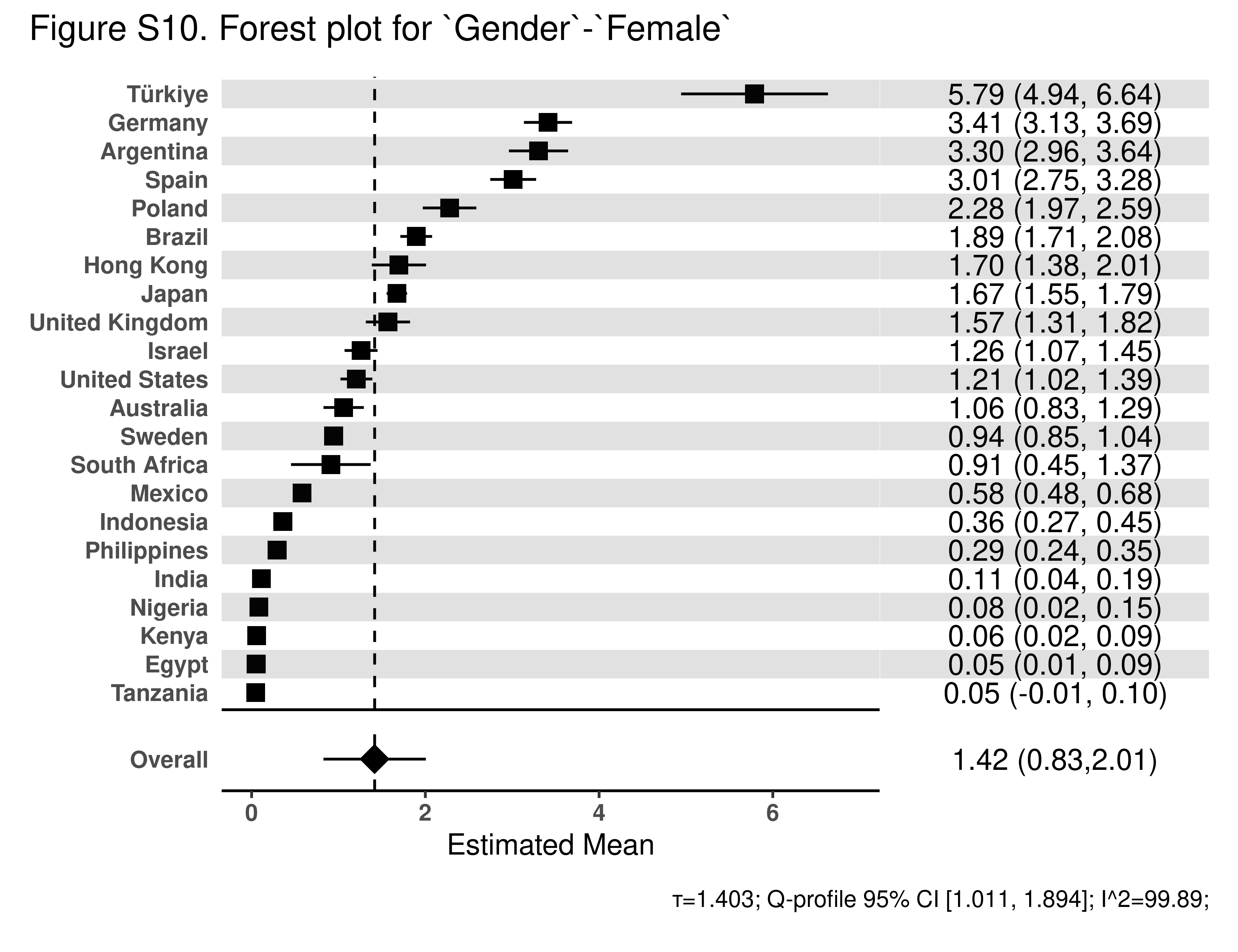

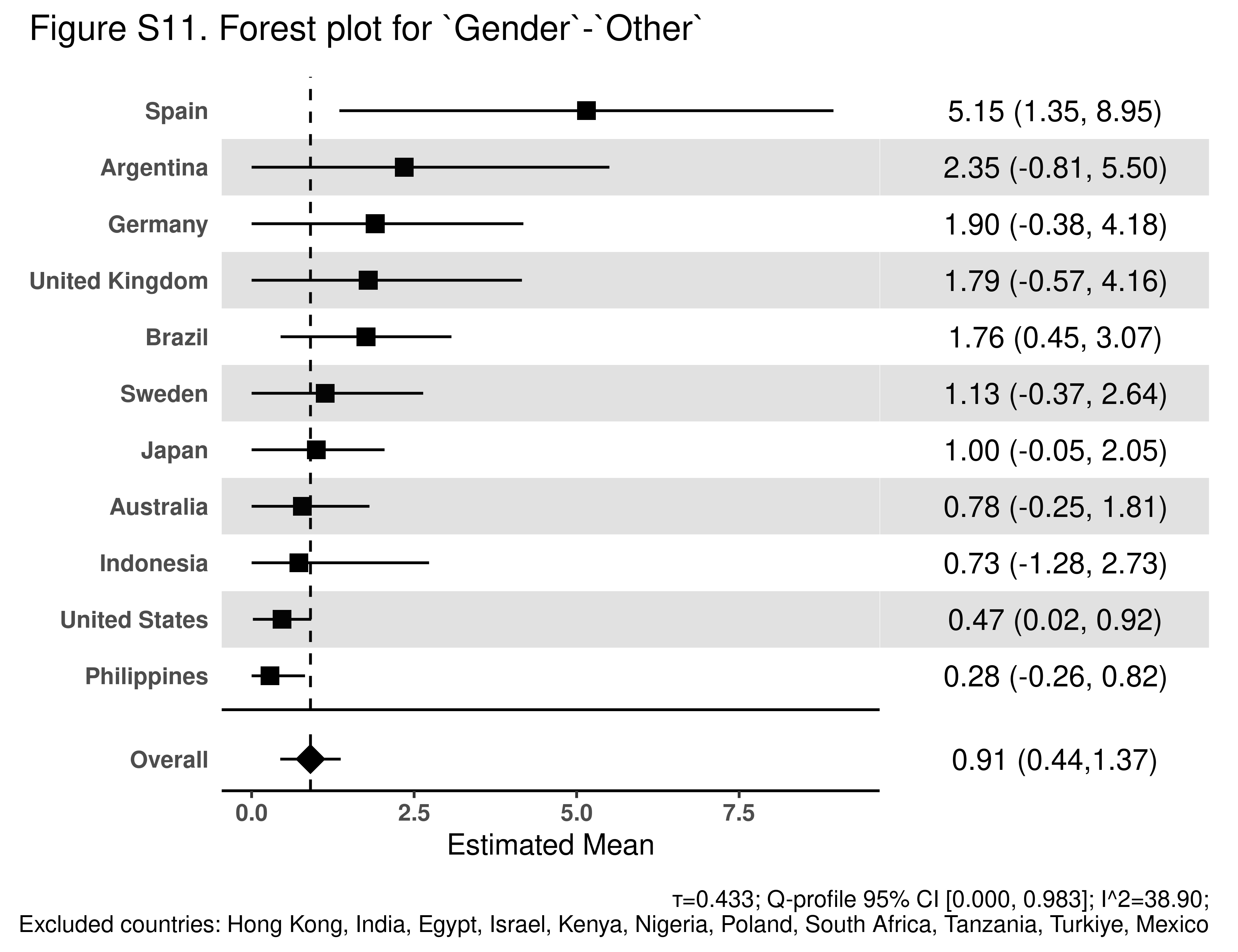

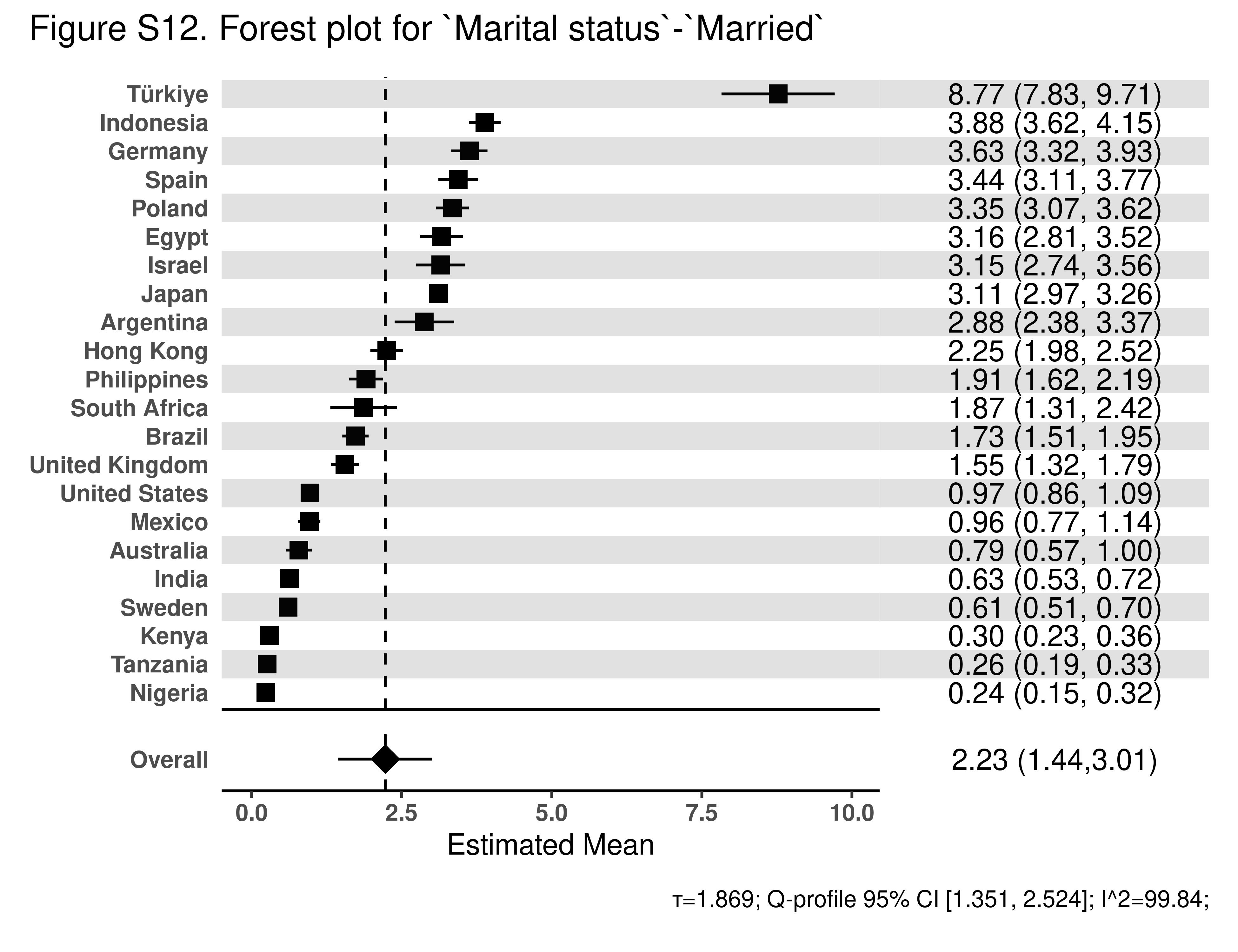

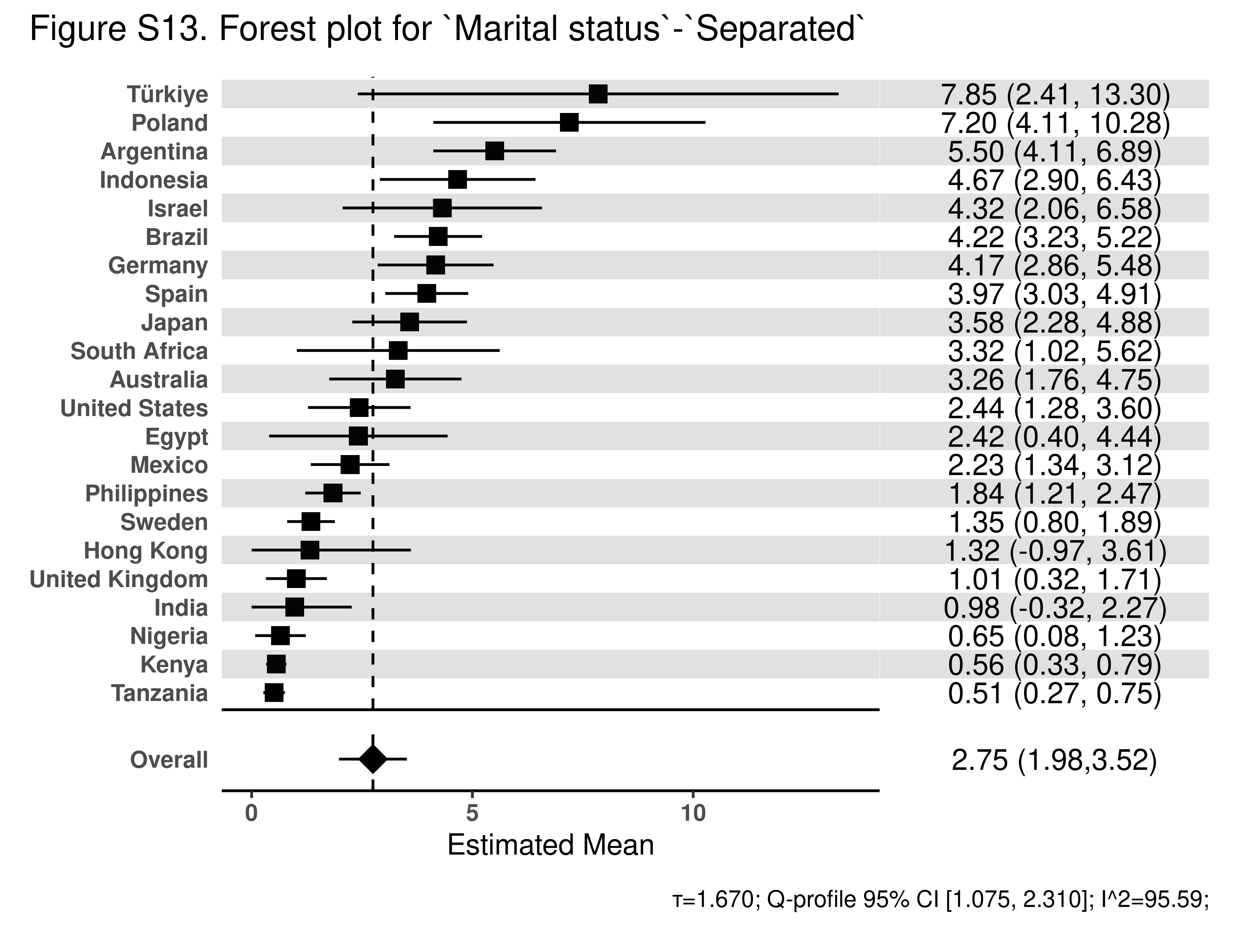

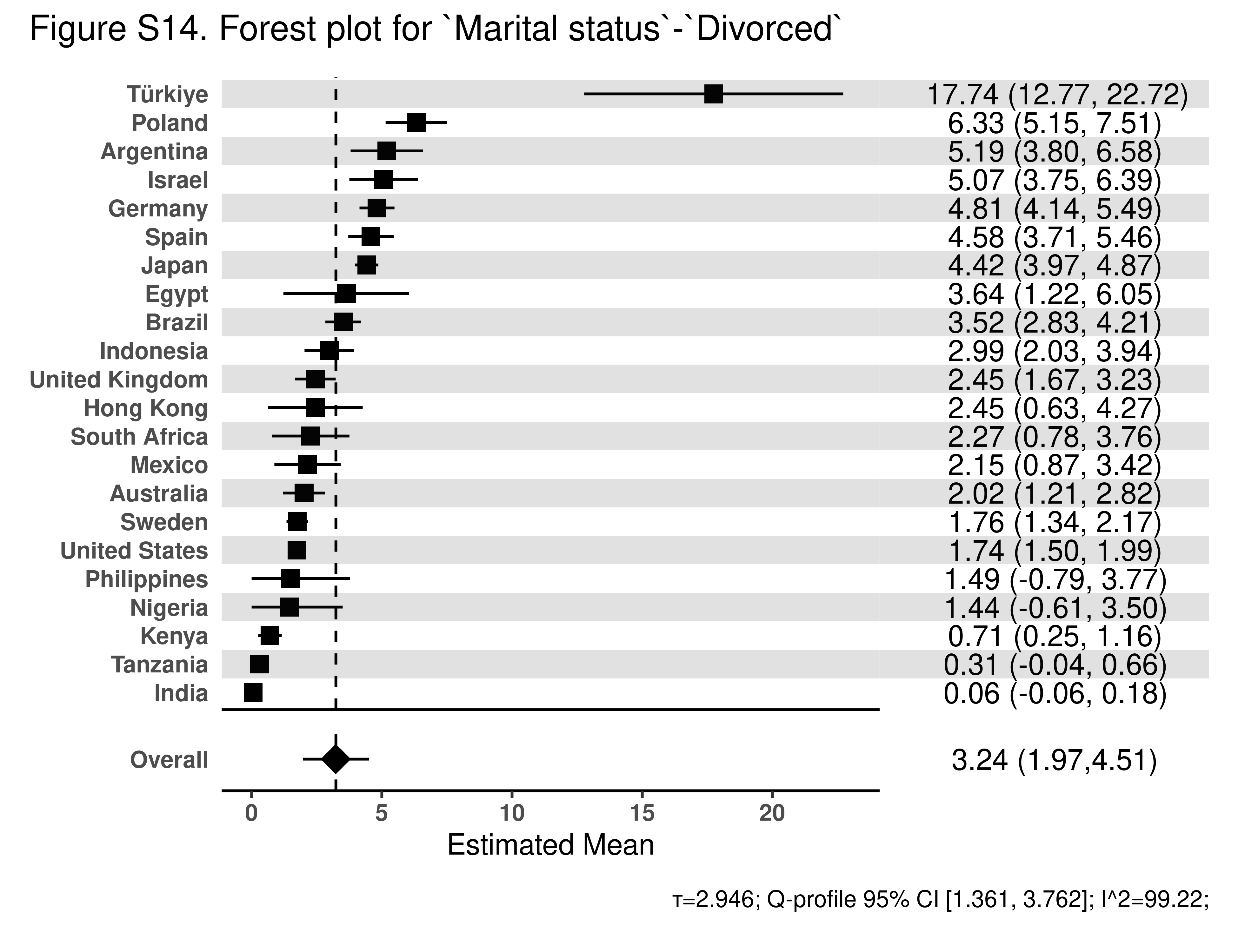

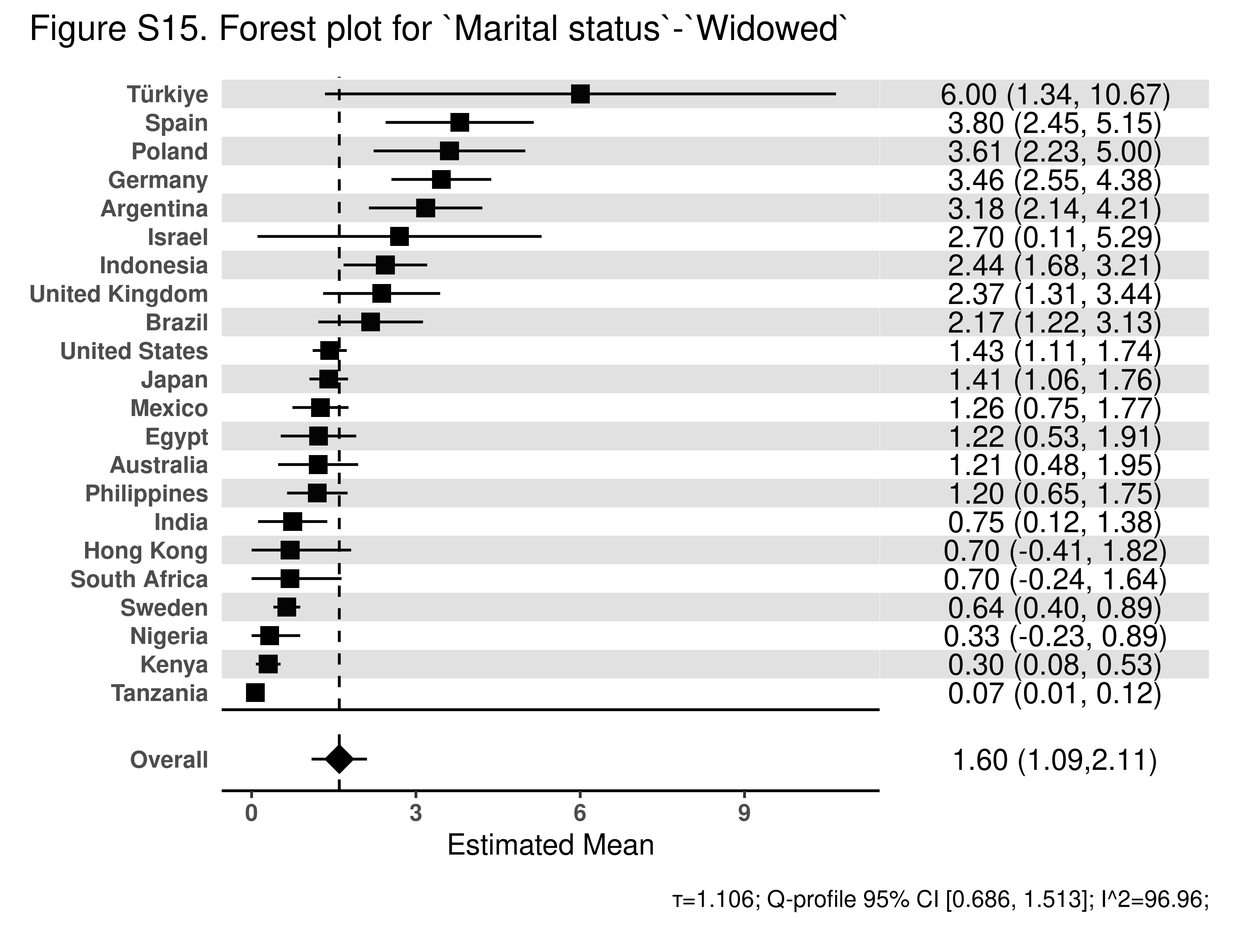

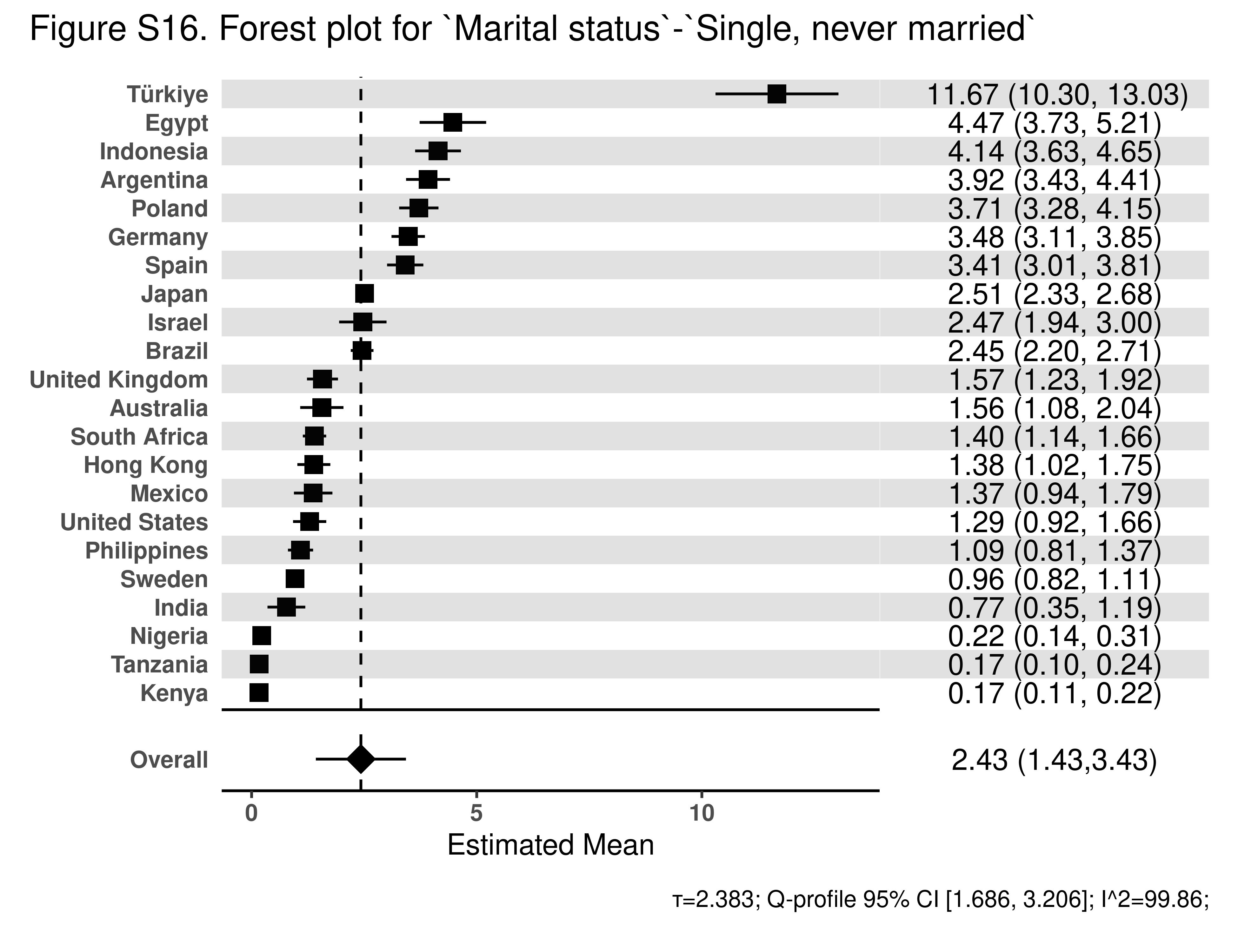

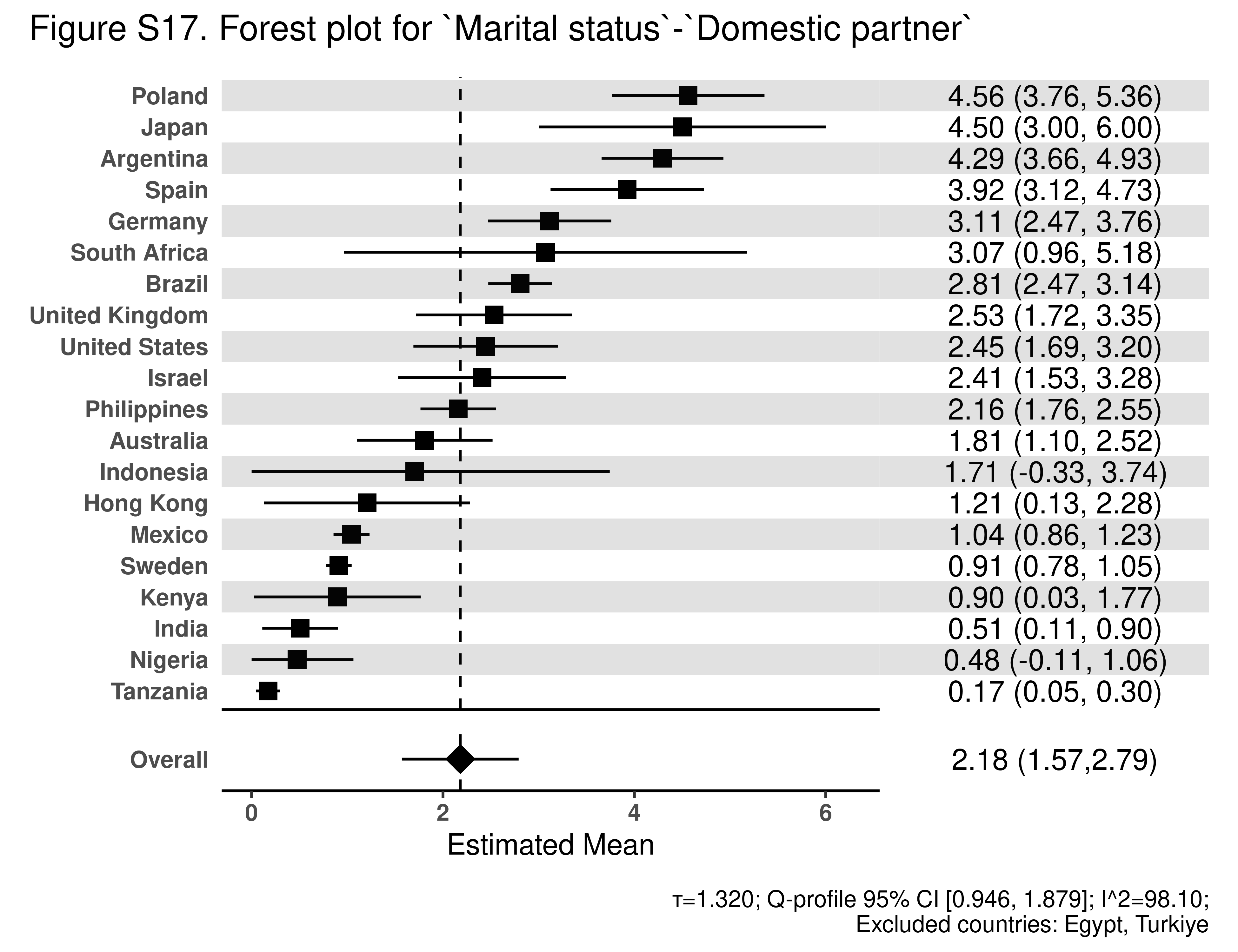

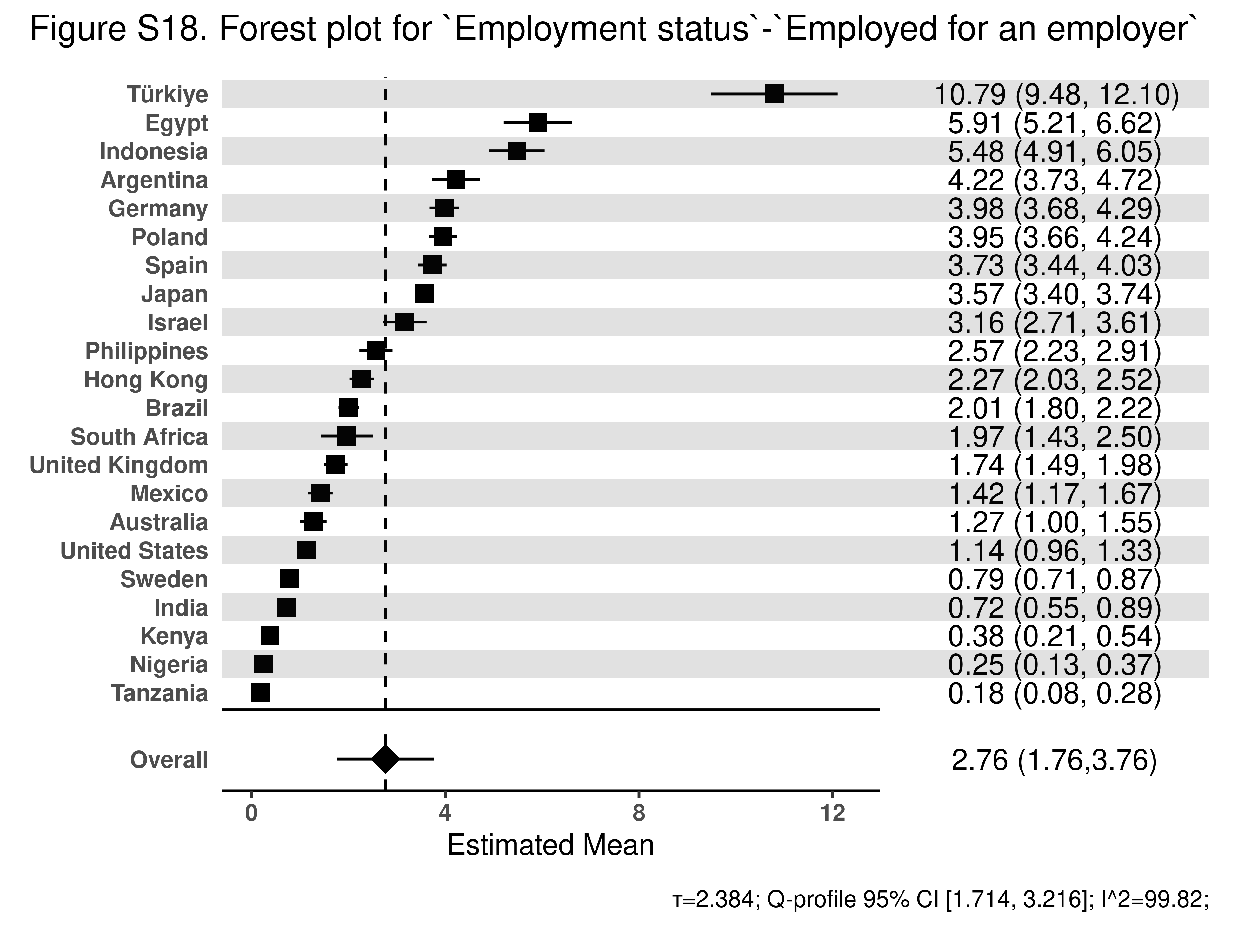

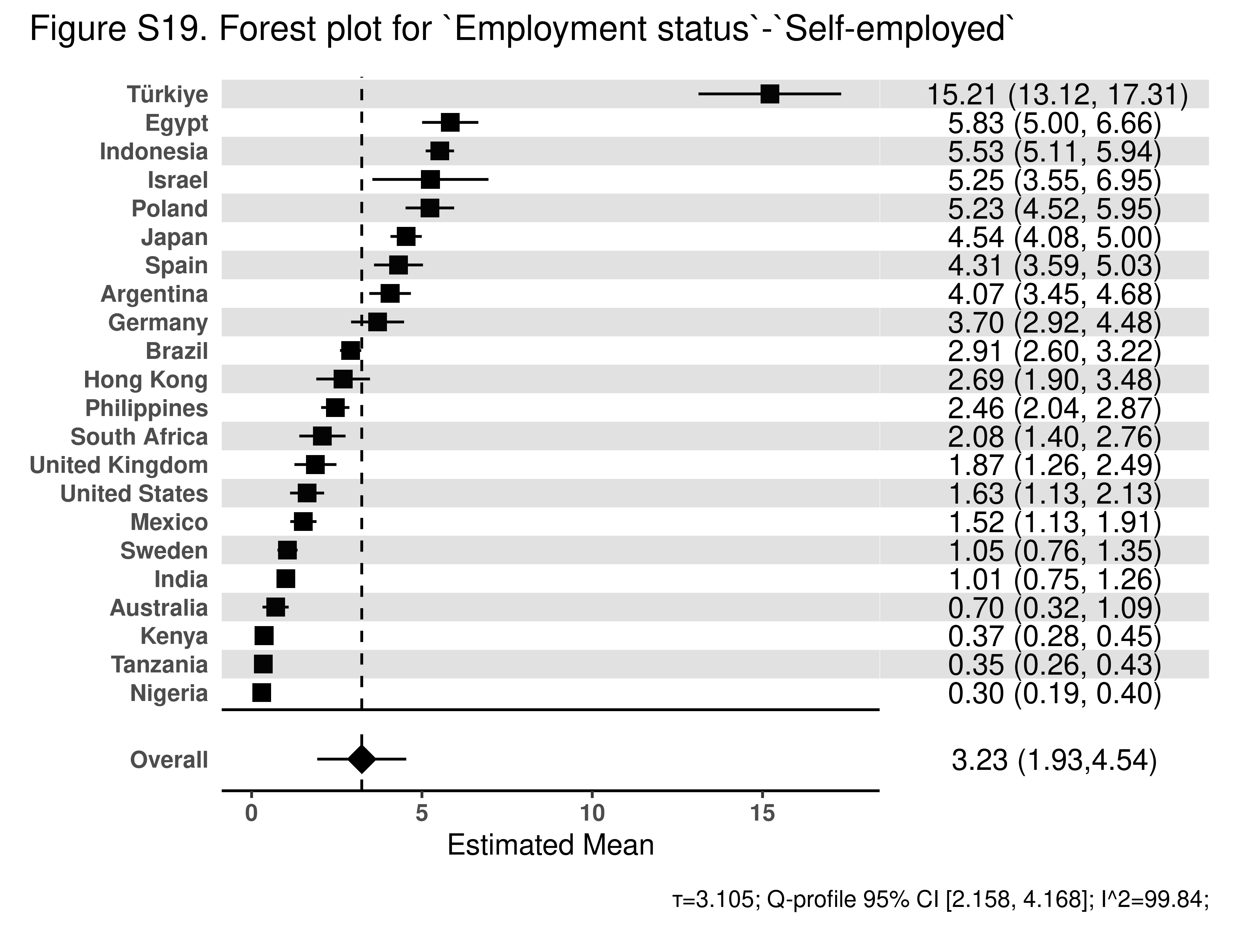

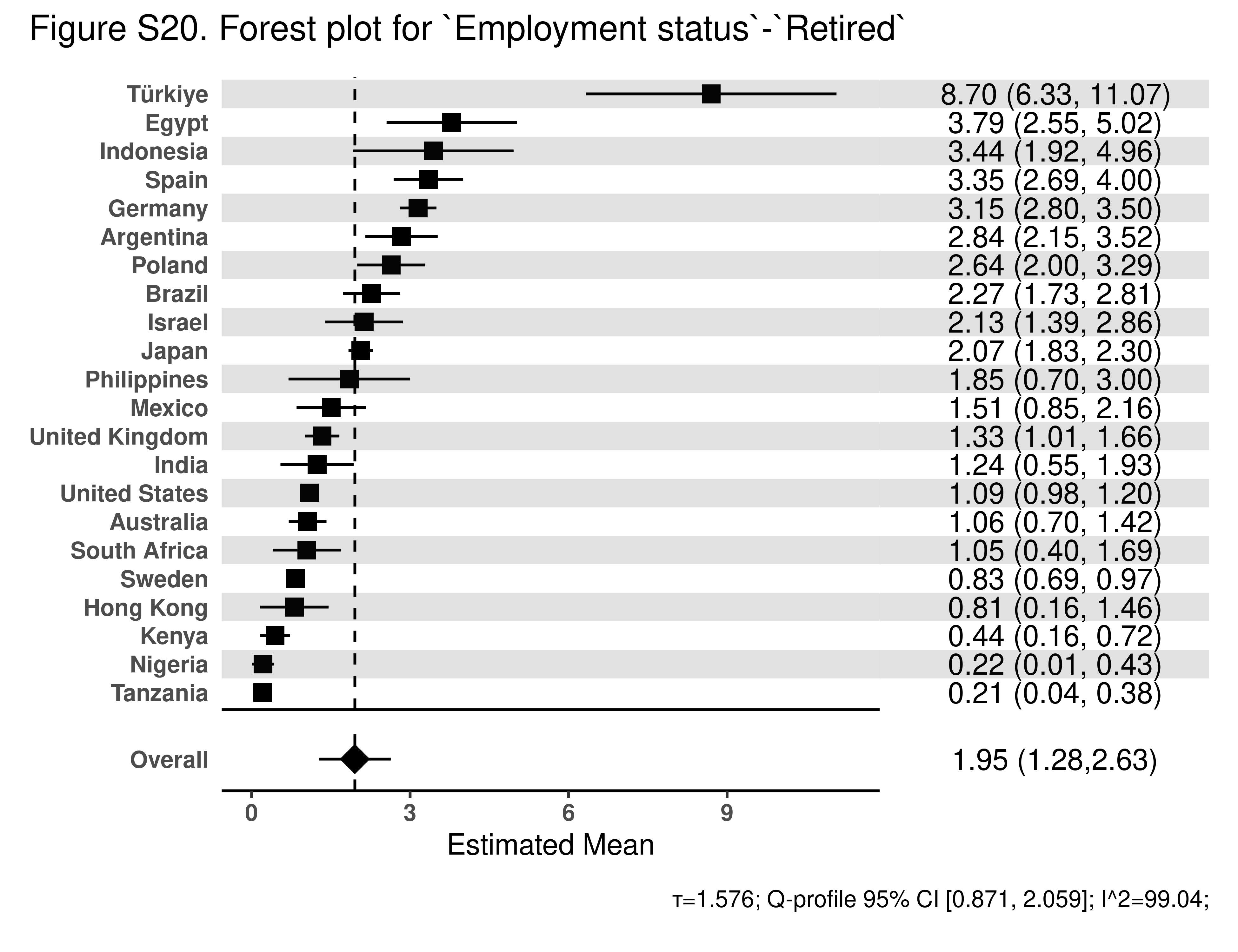

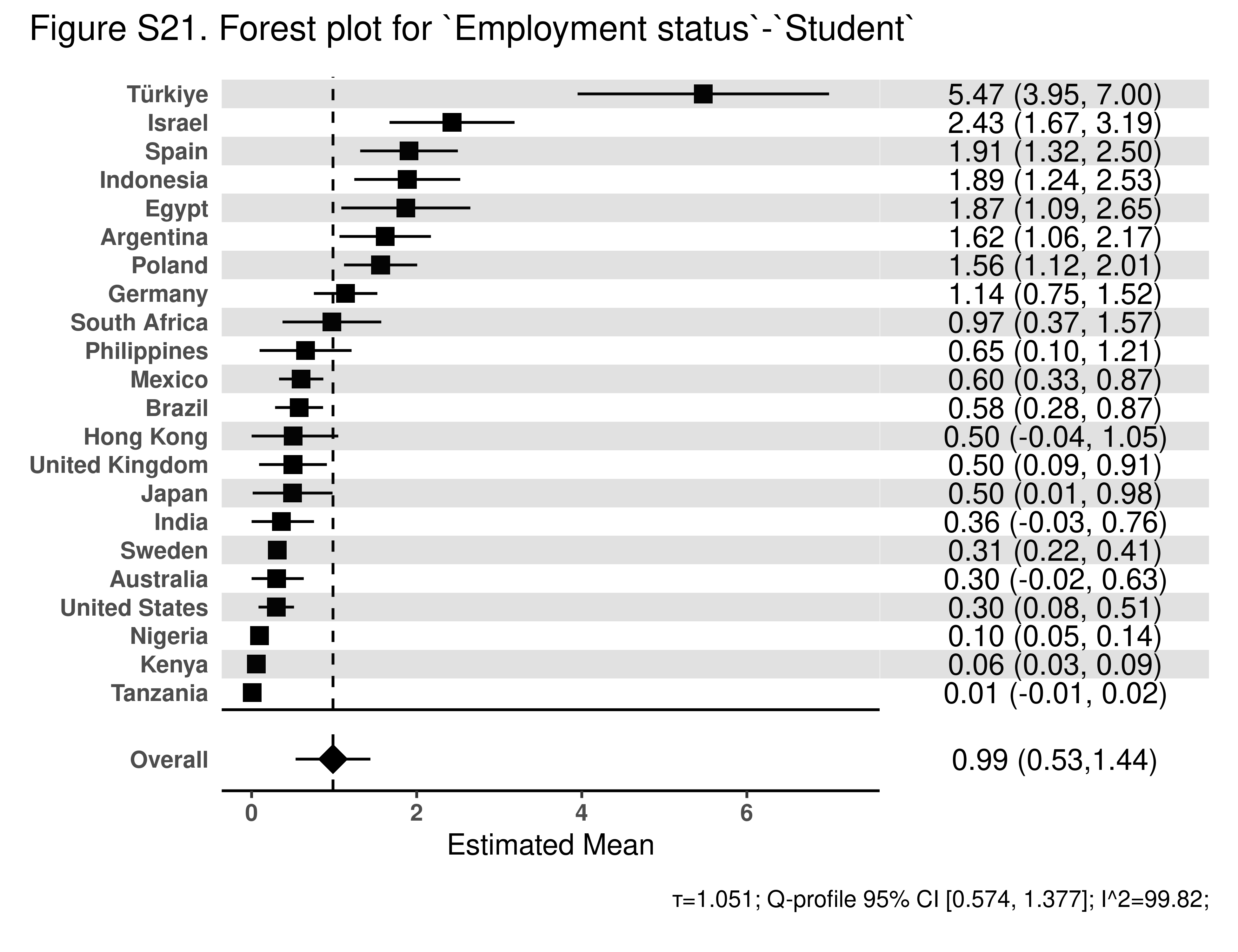

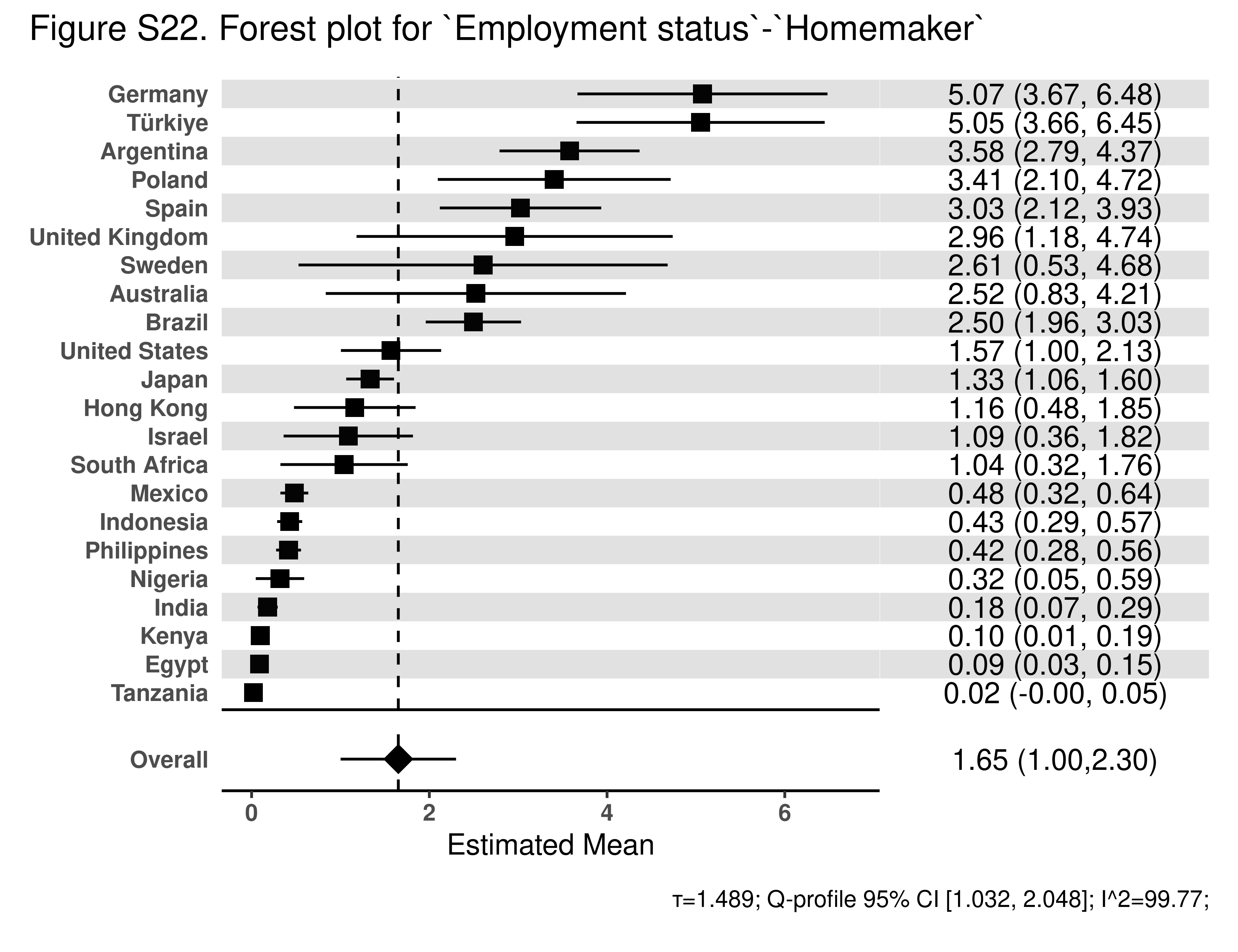

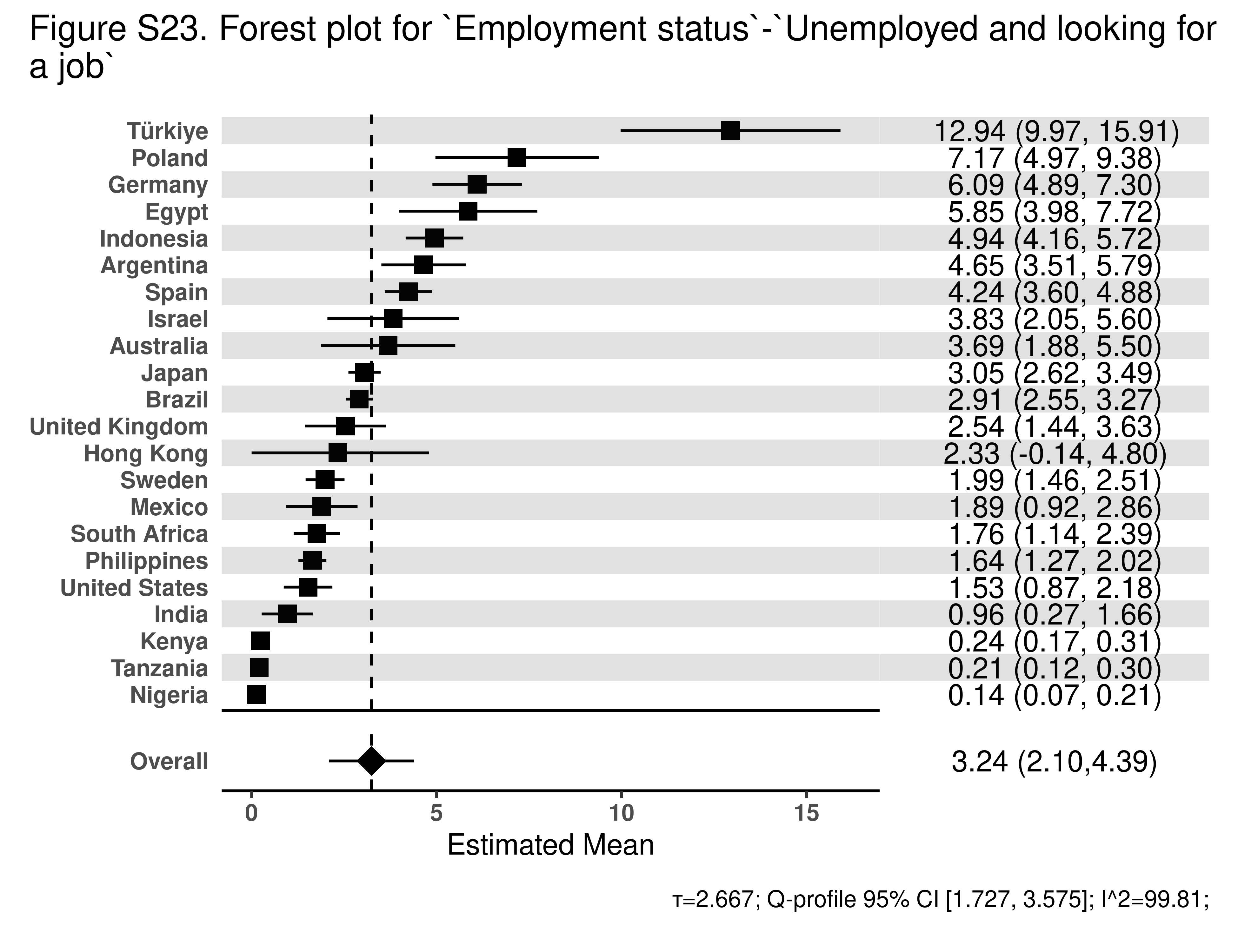

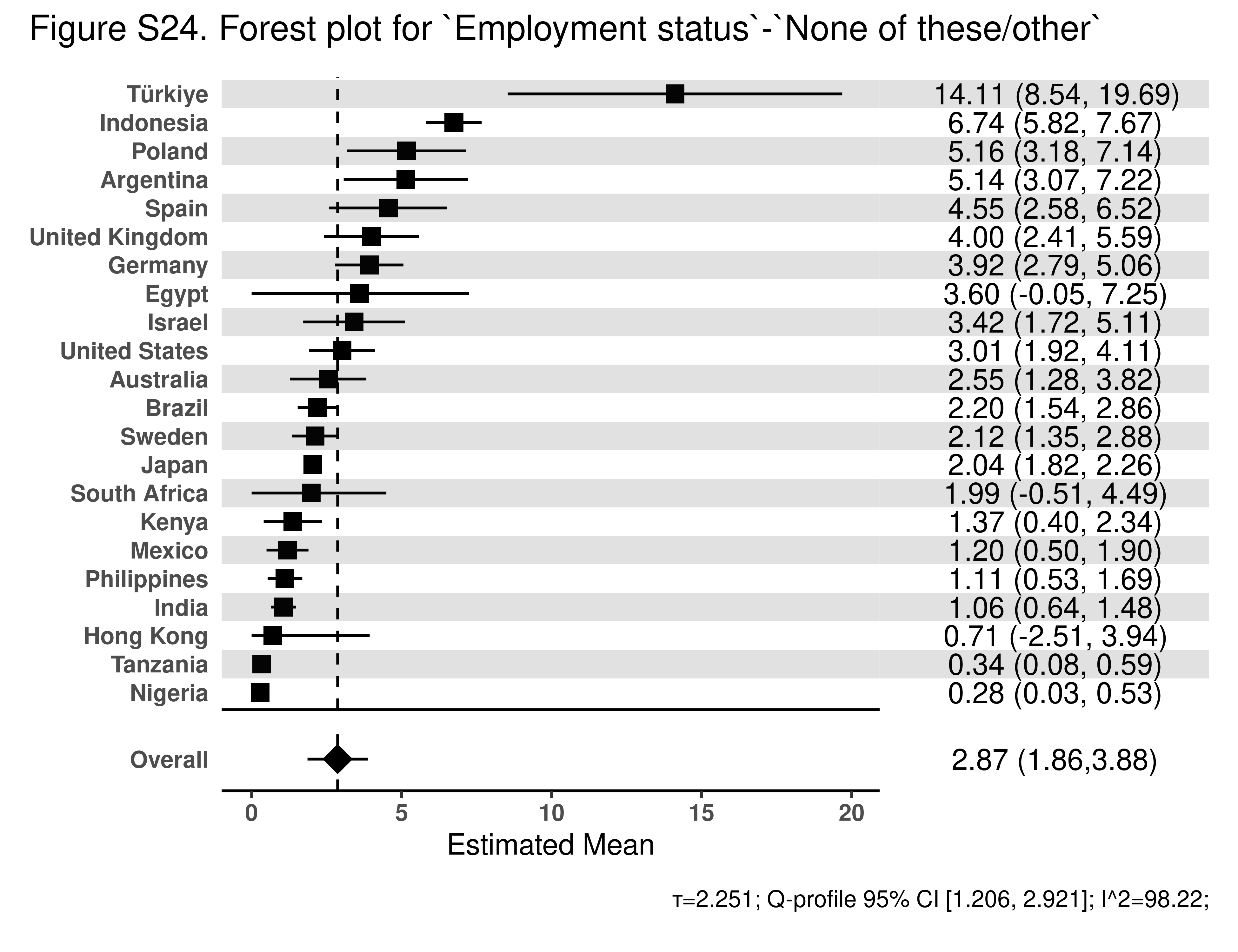



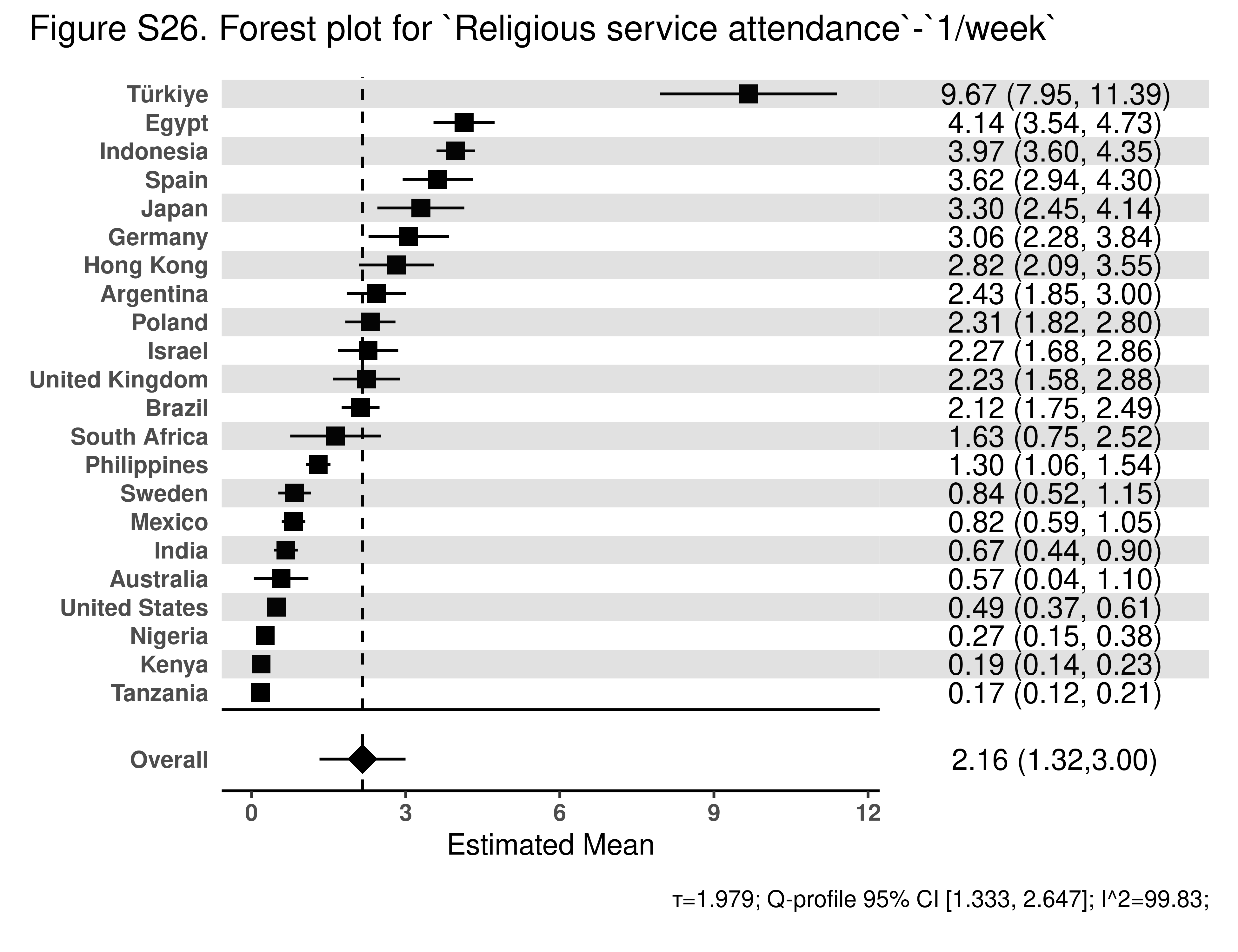

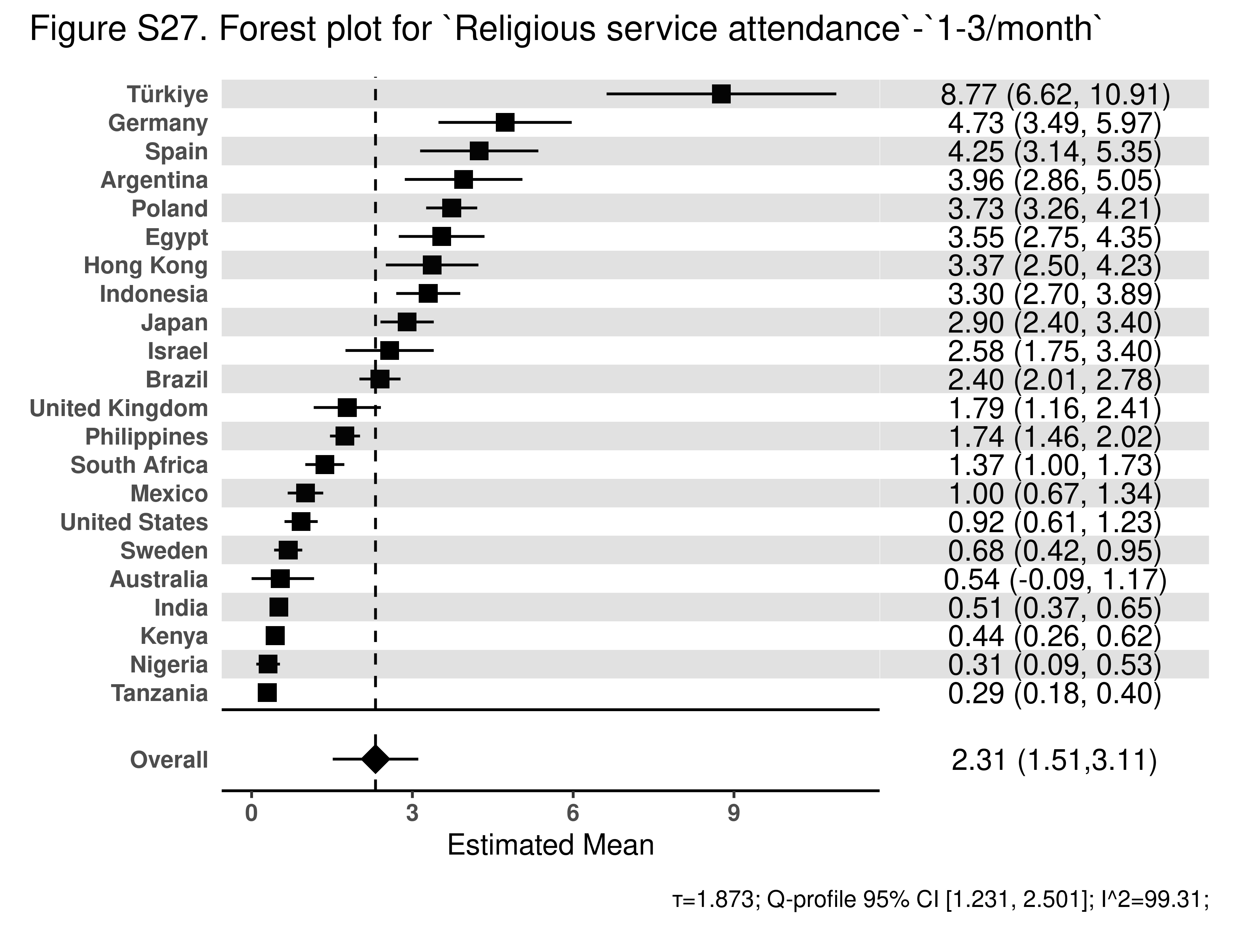

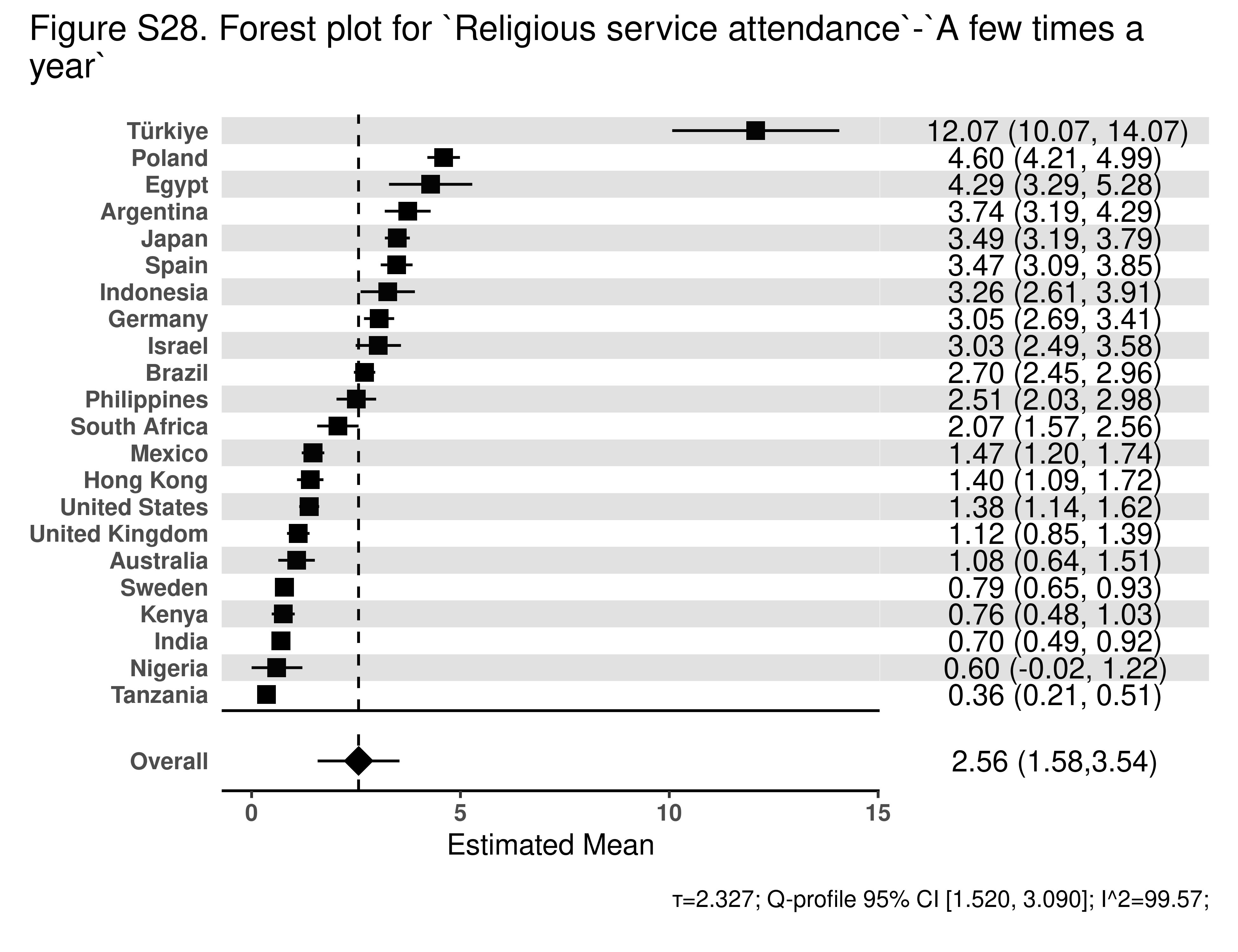

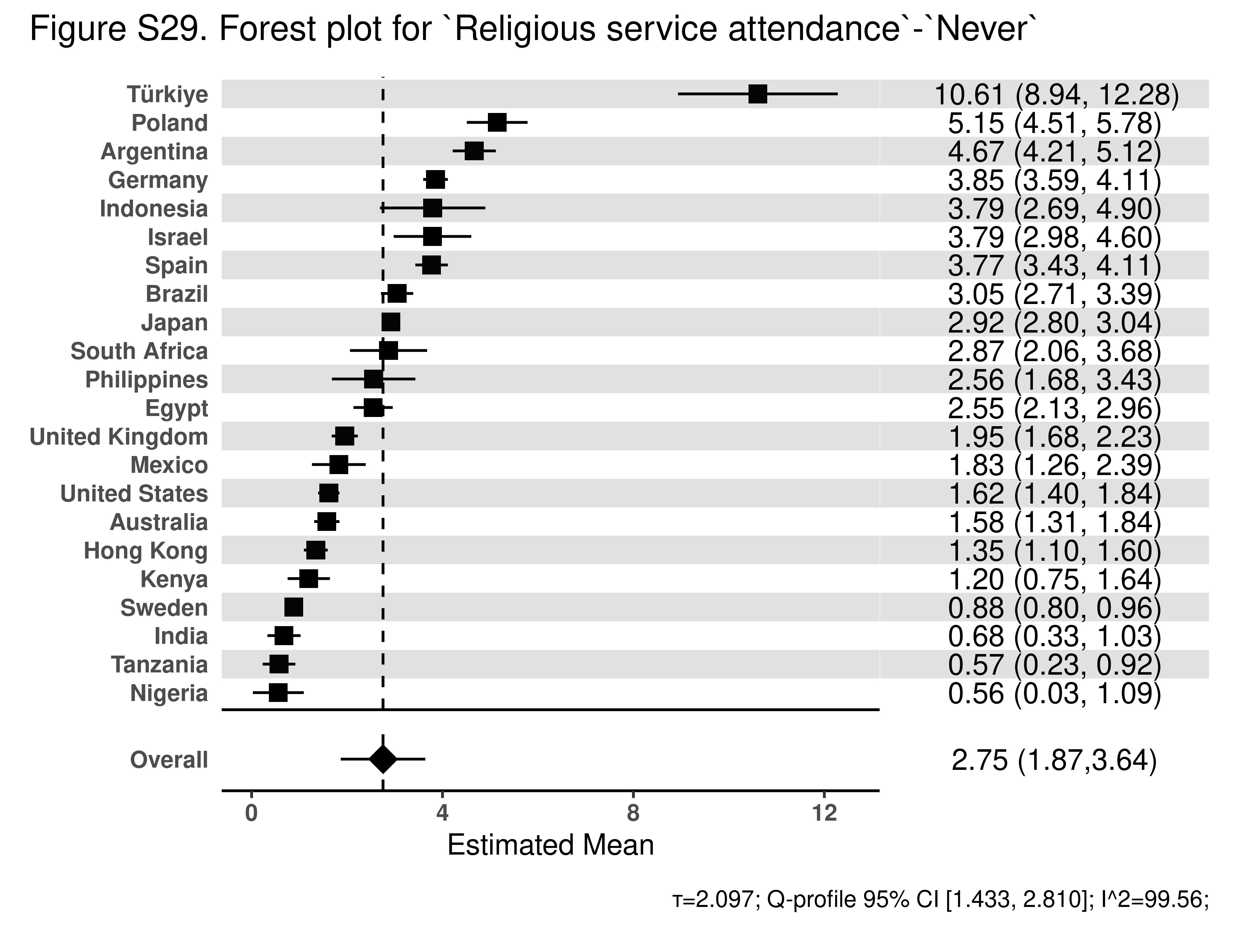

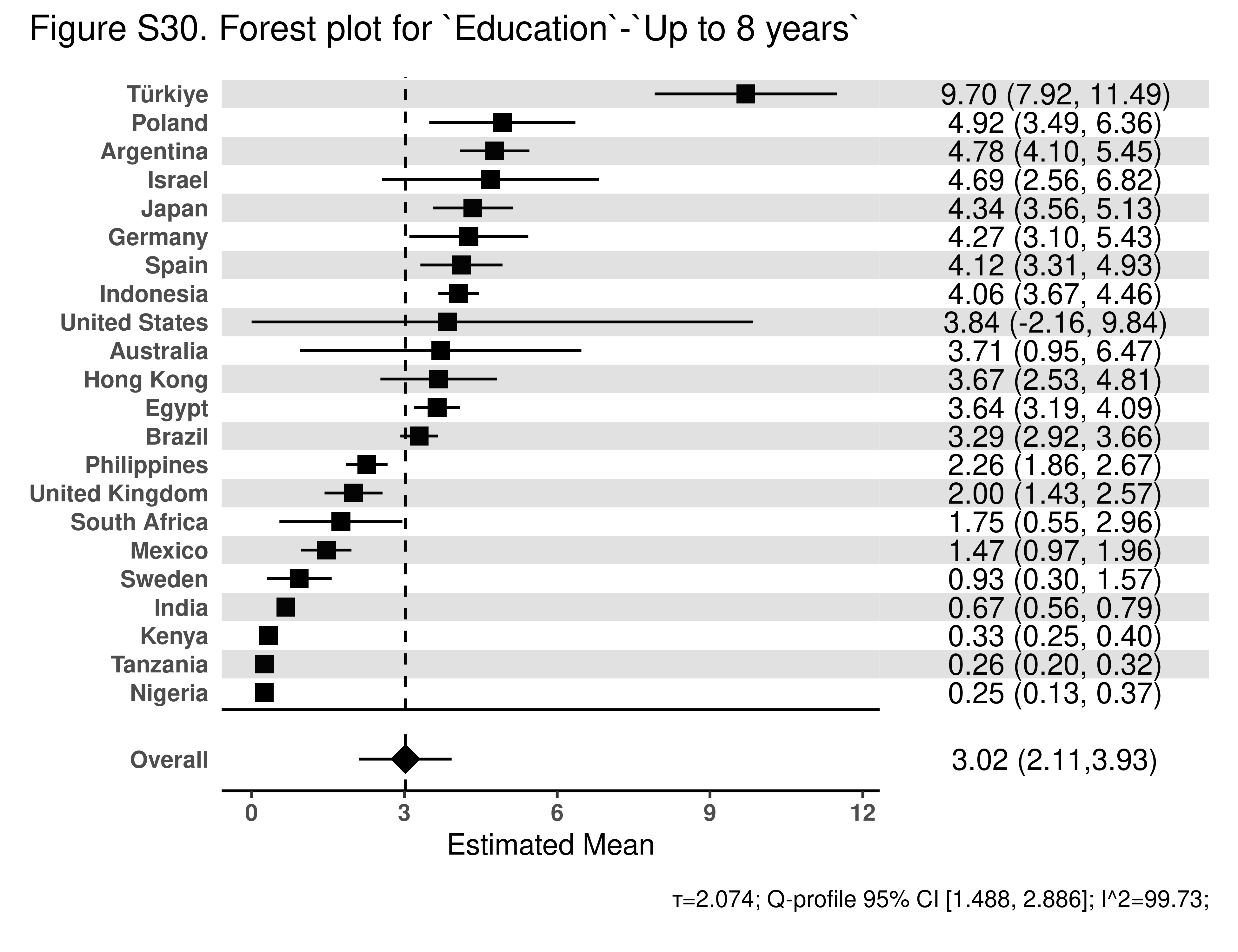

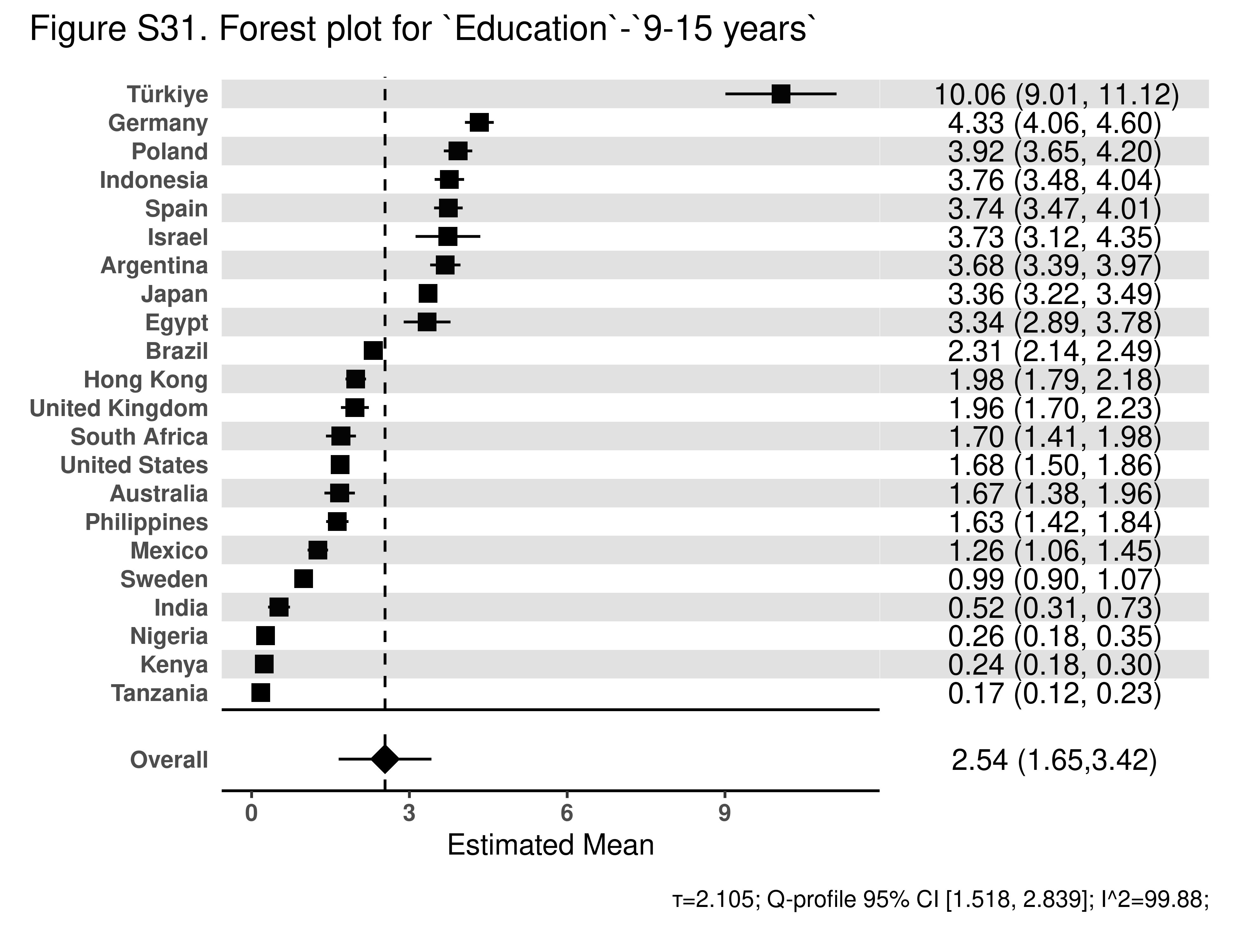

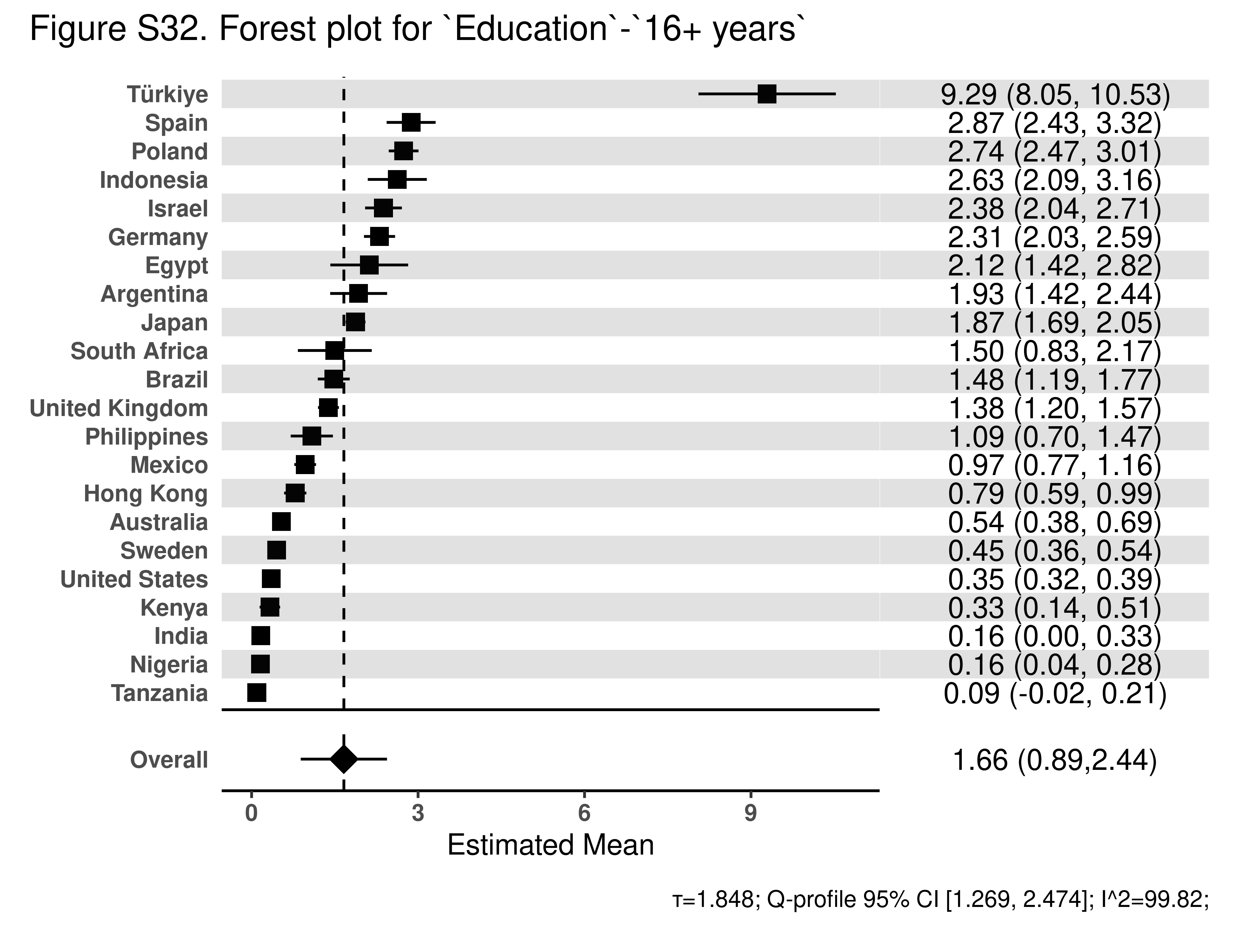

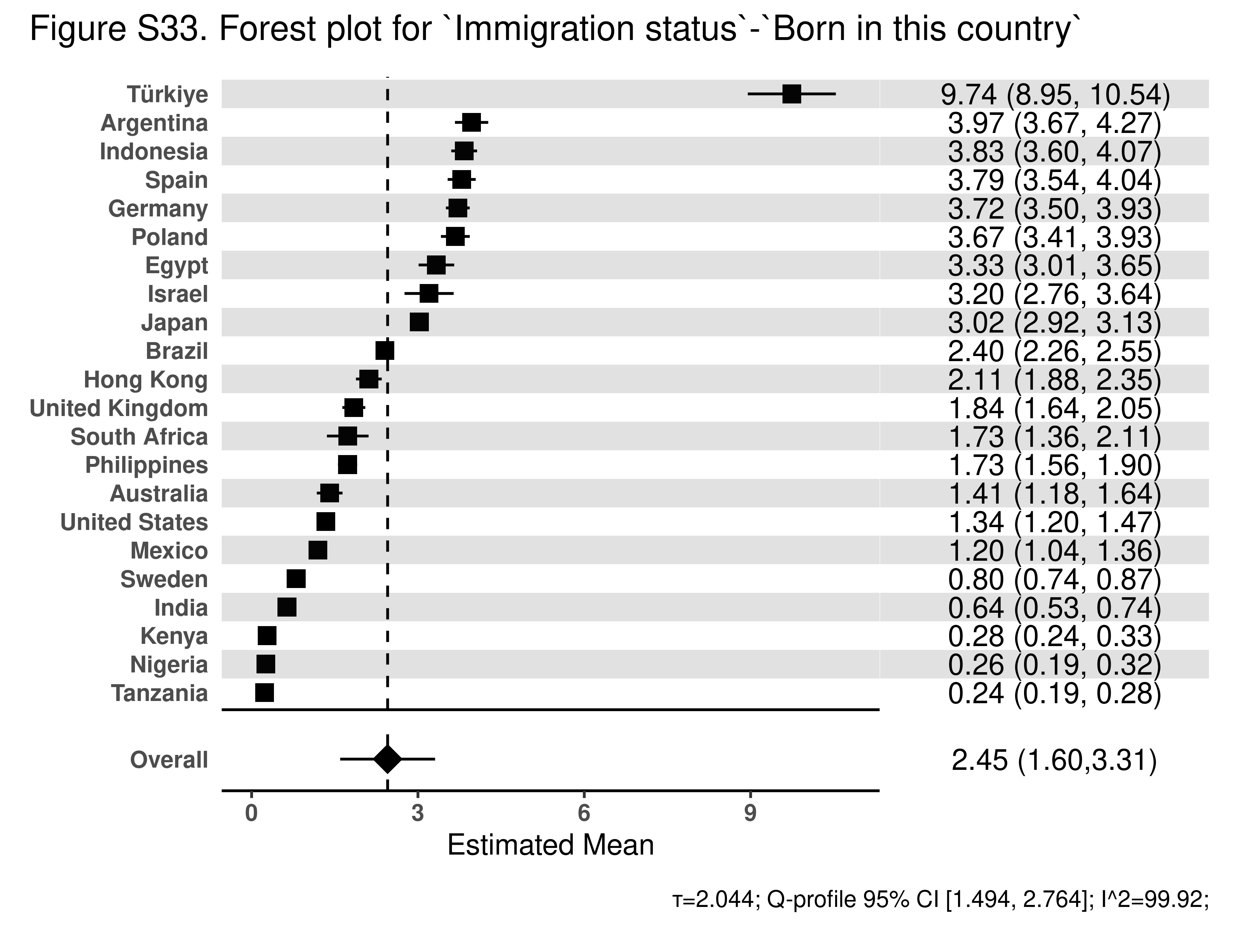

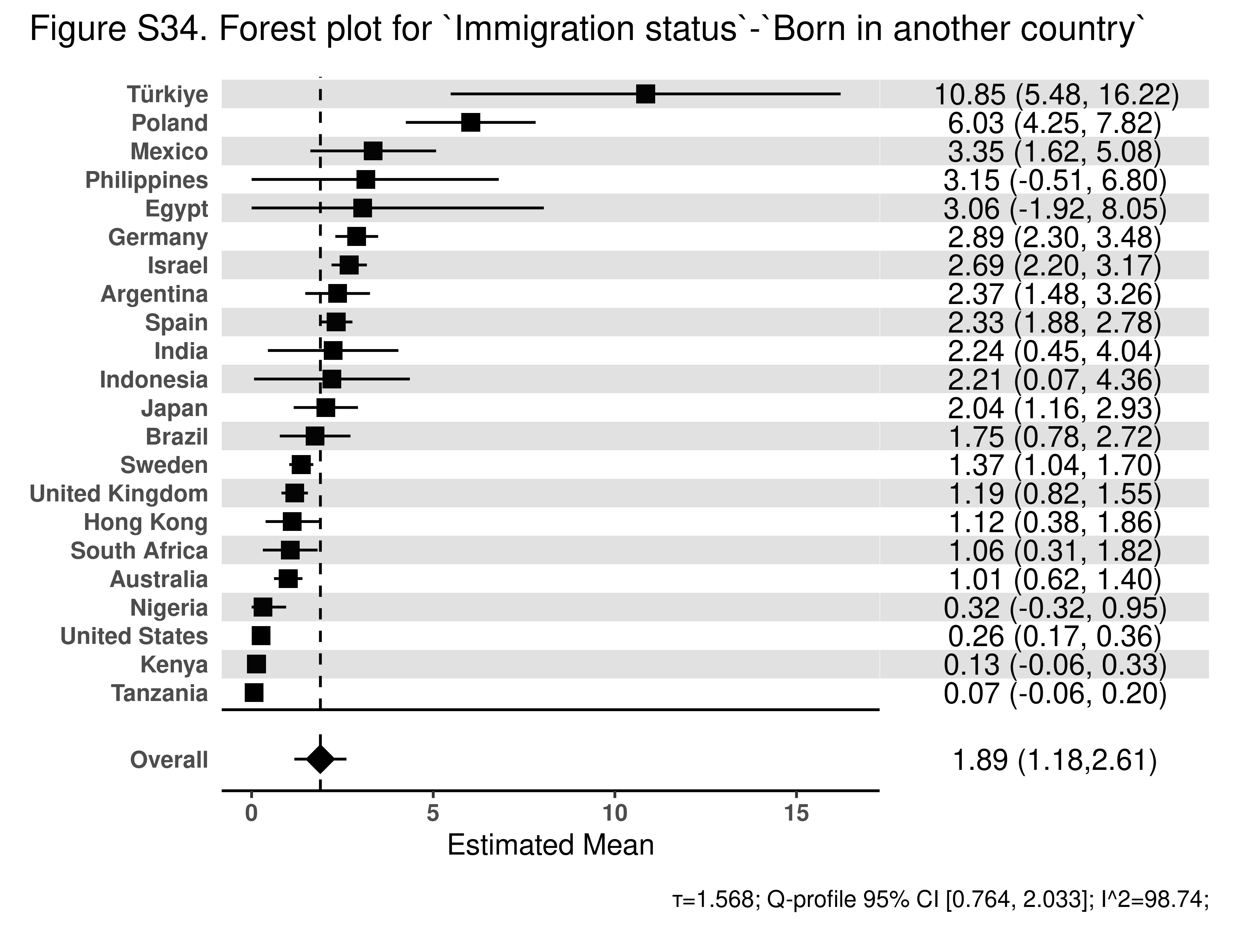


# Figures S35-S68. Forest plots of Intensity for Demographic Categories


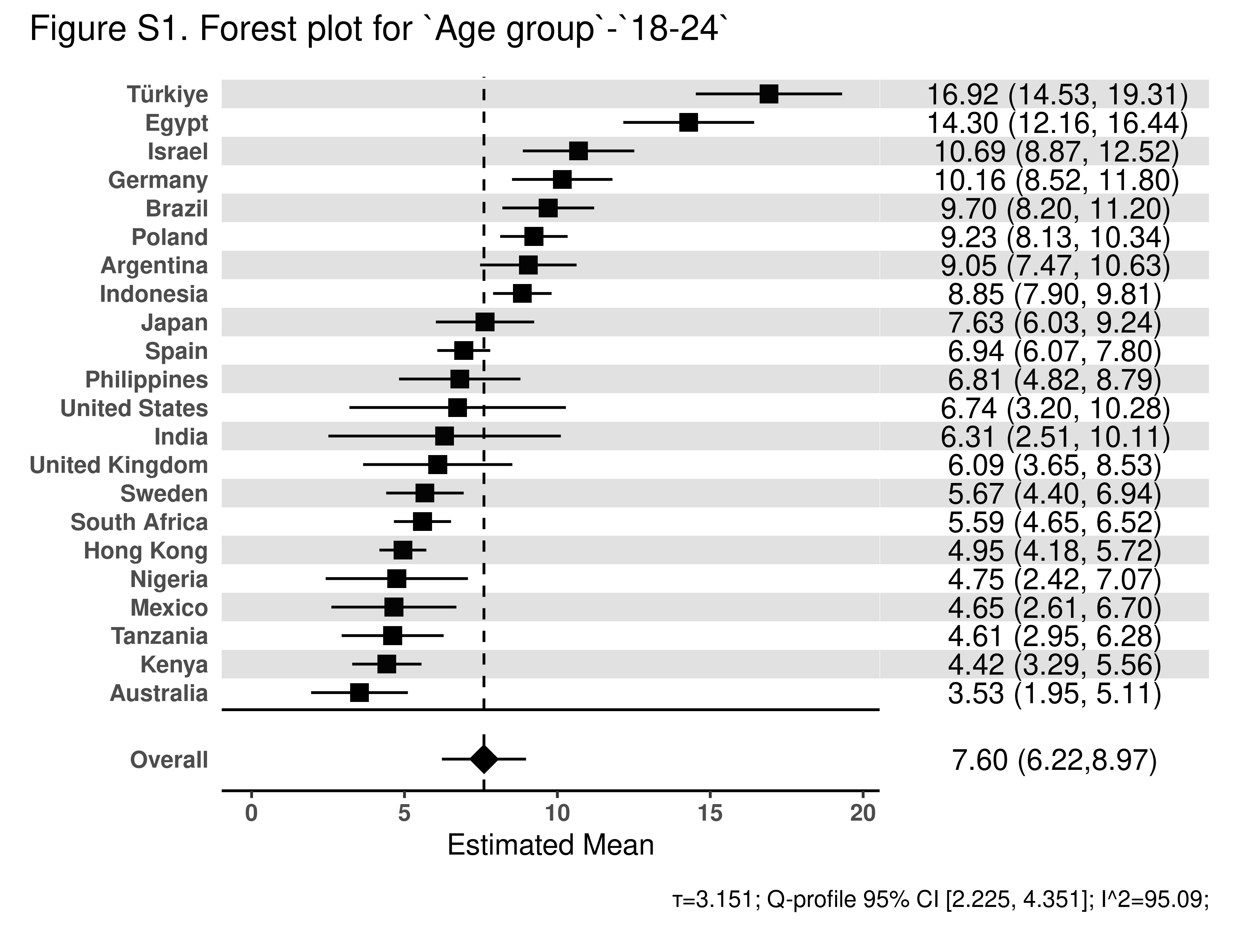


**Figure S36.**

Intensity

**Figure S35.**


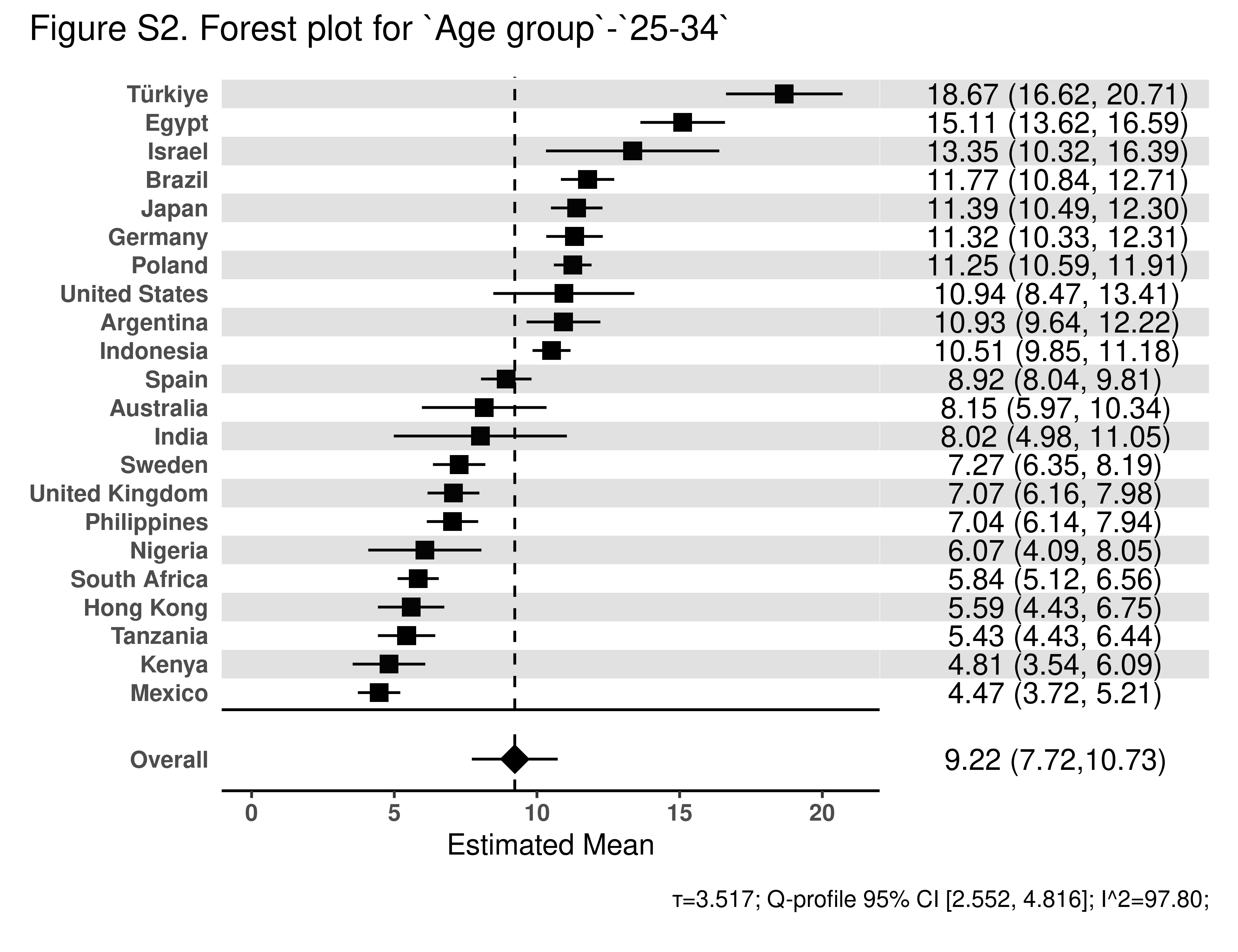


Intensity


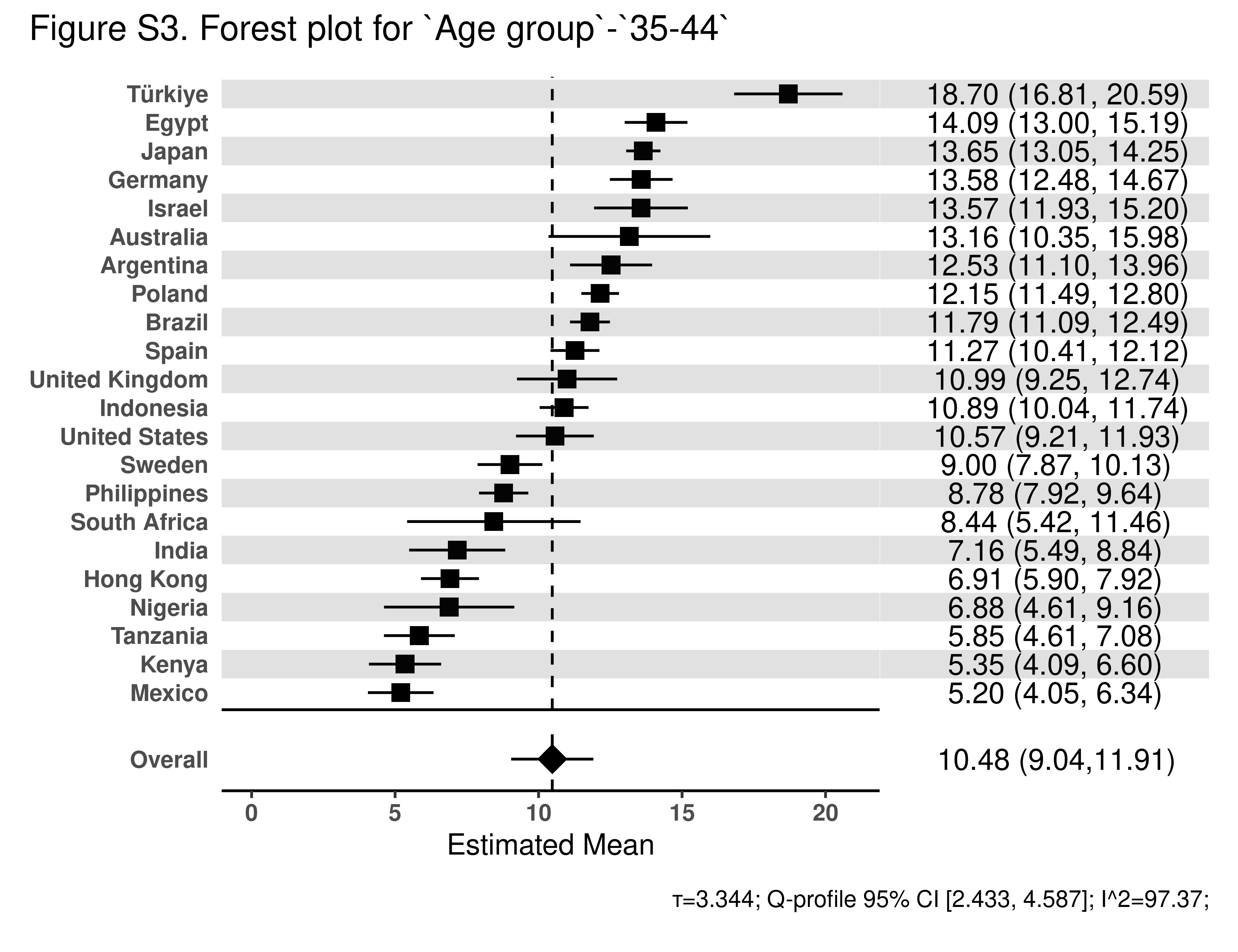

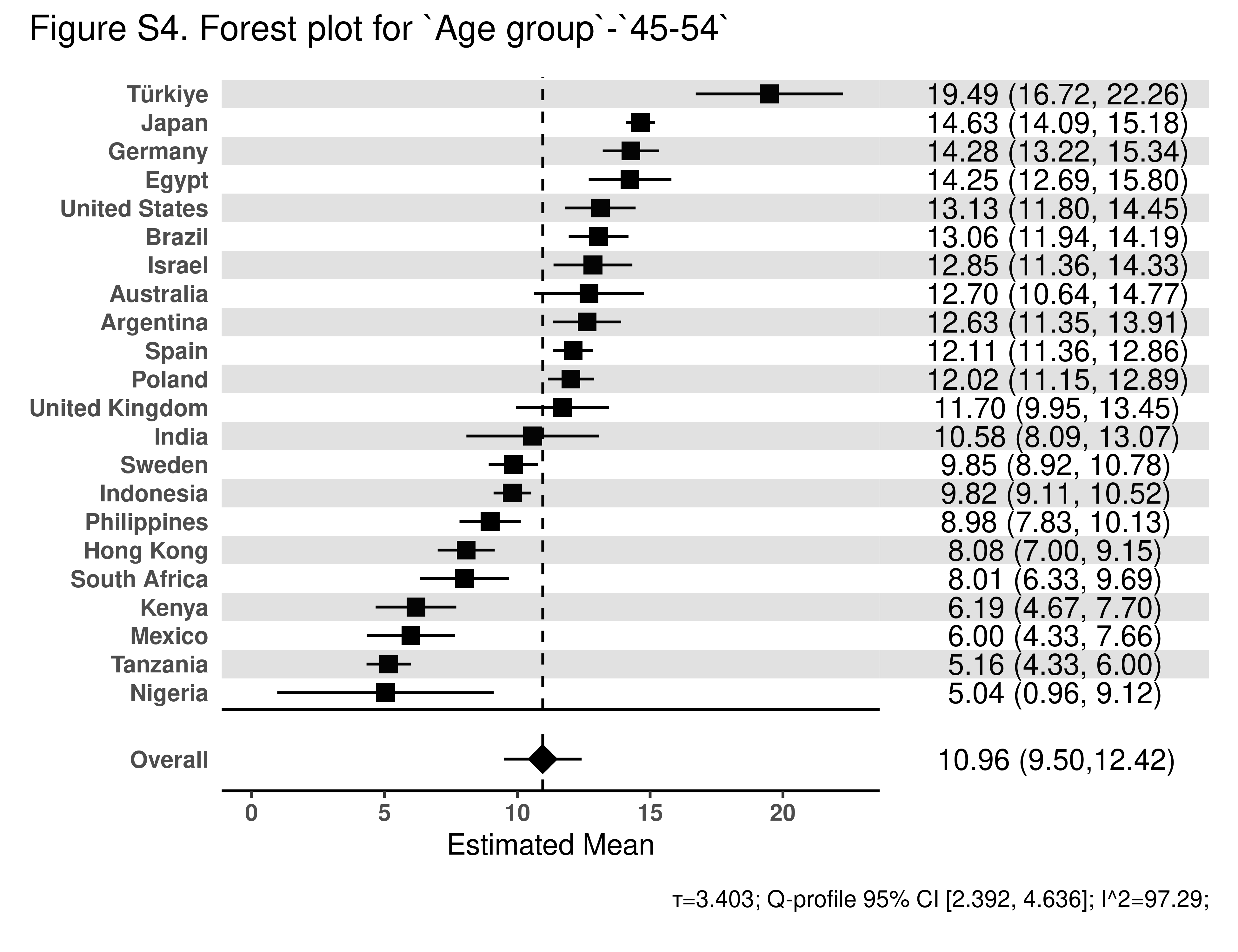

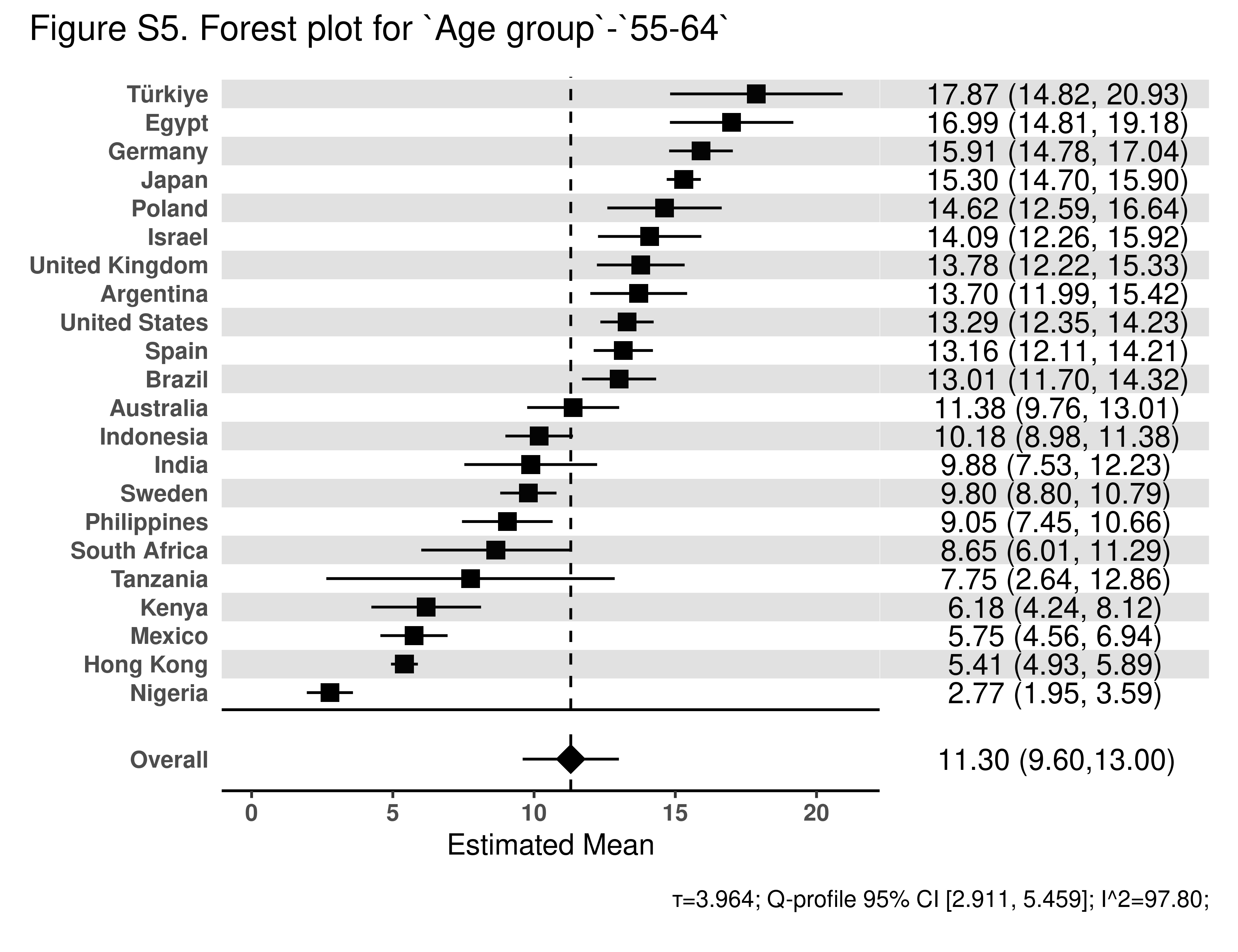

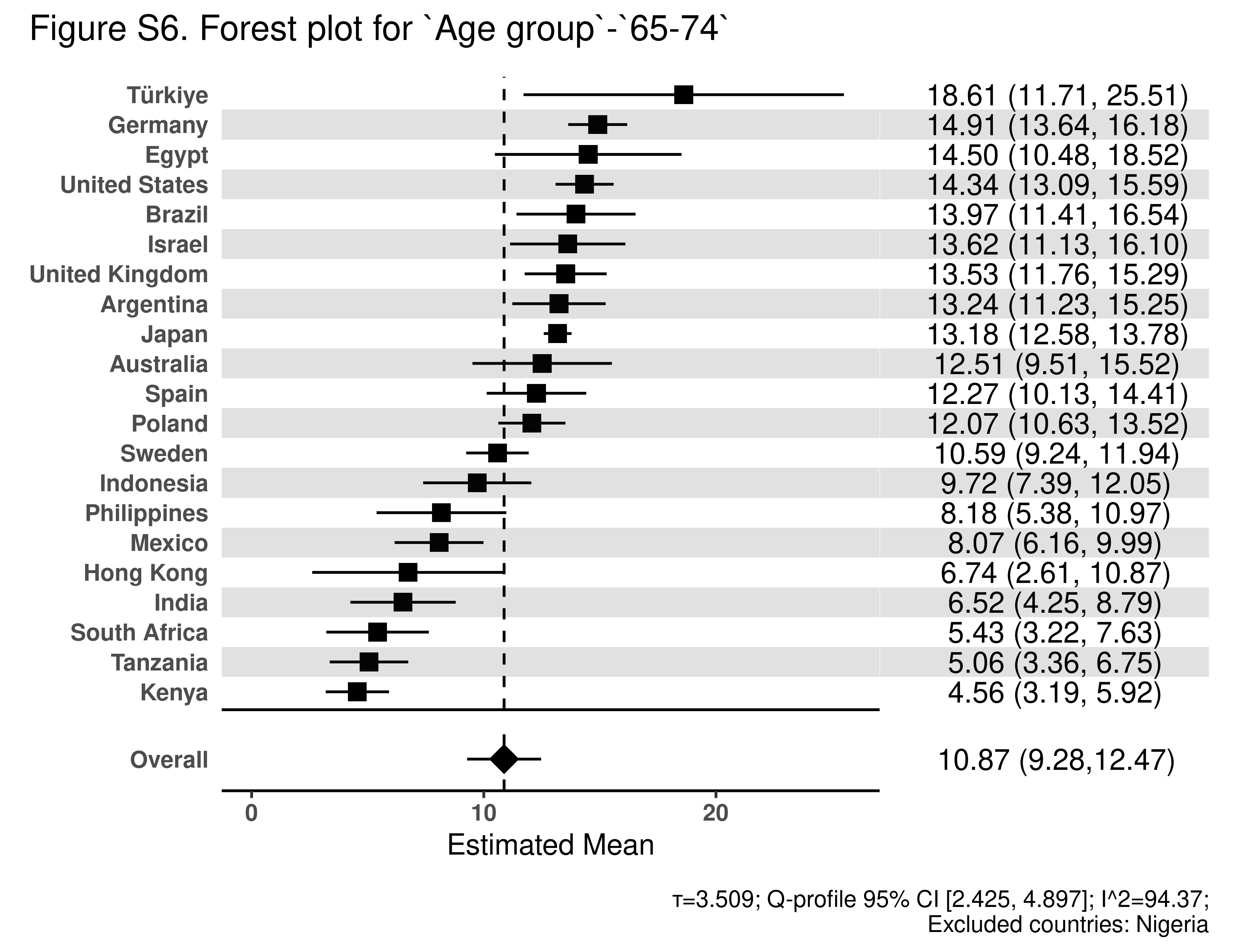

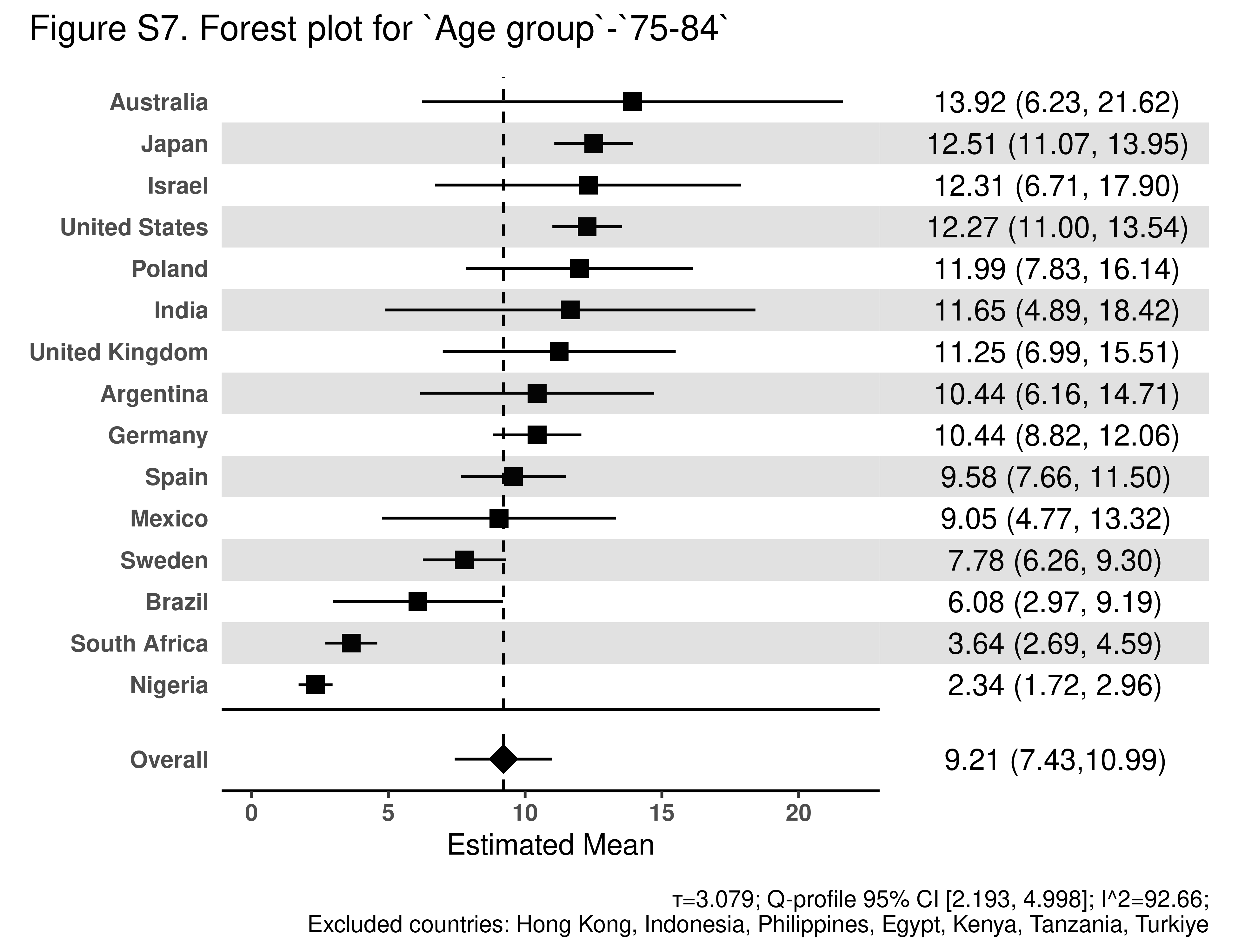

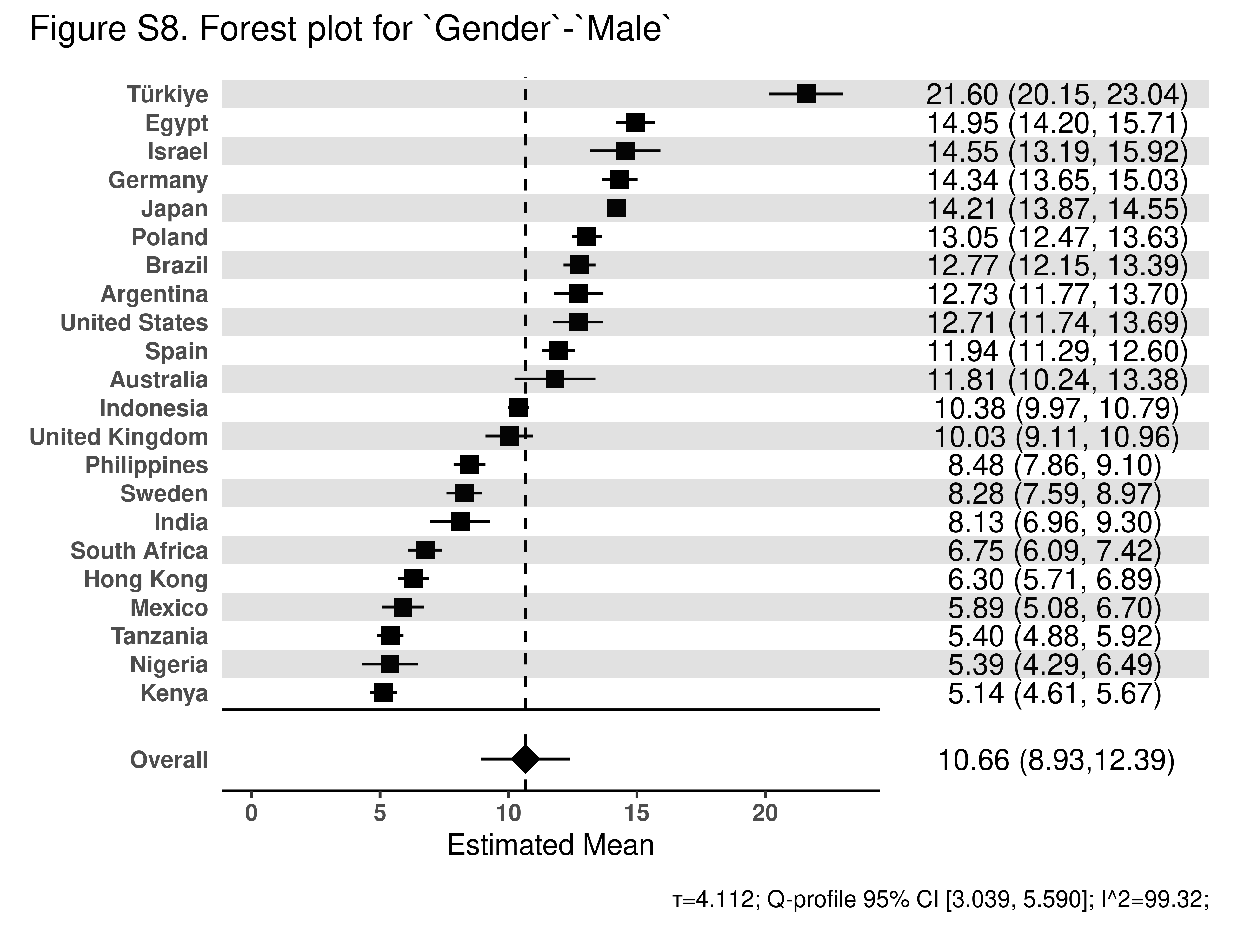

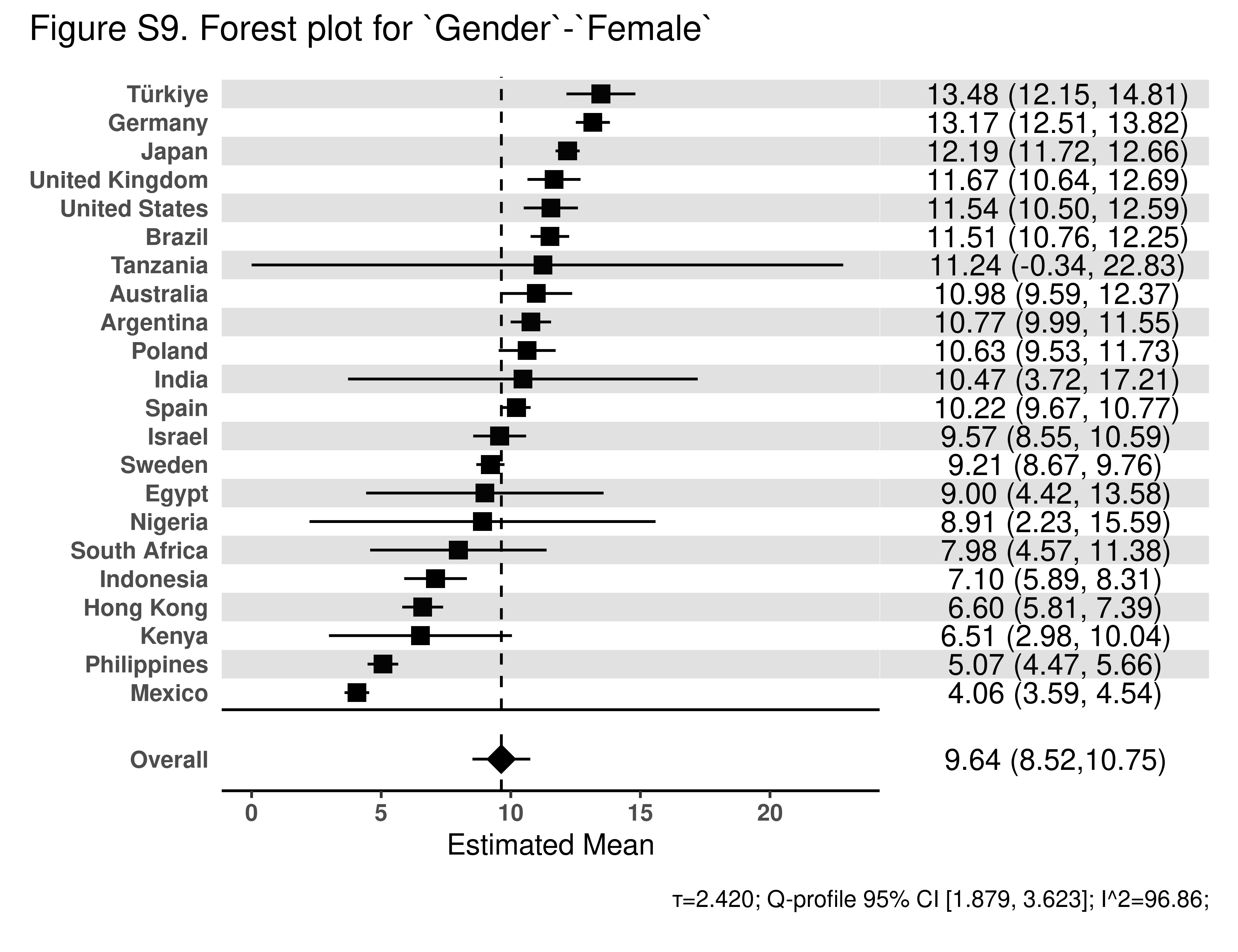

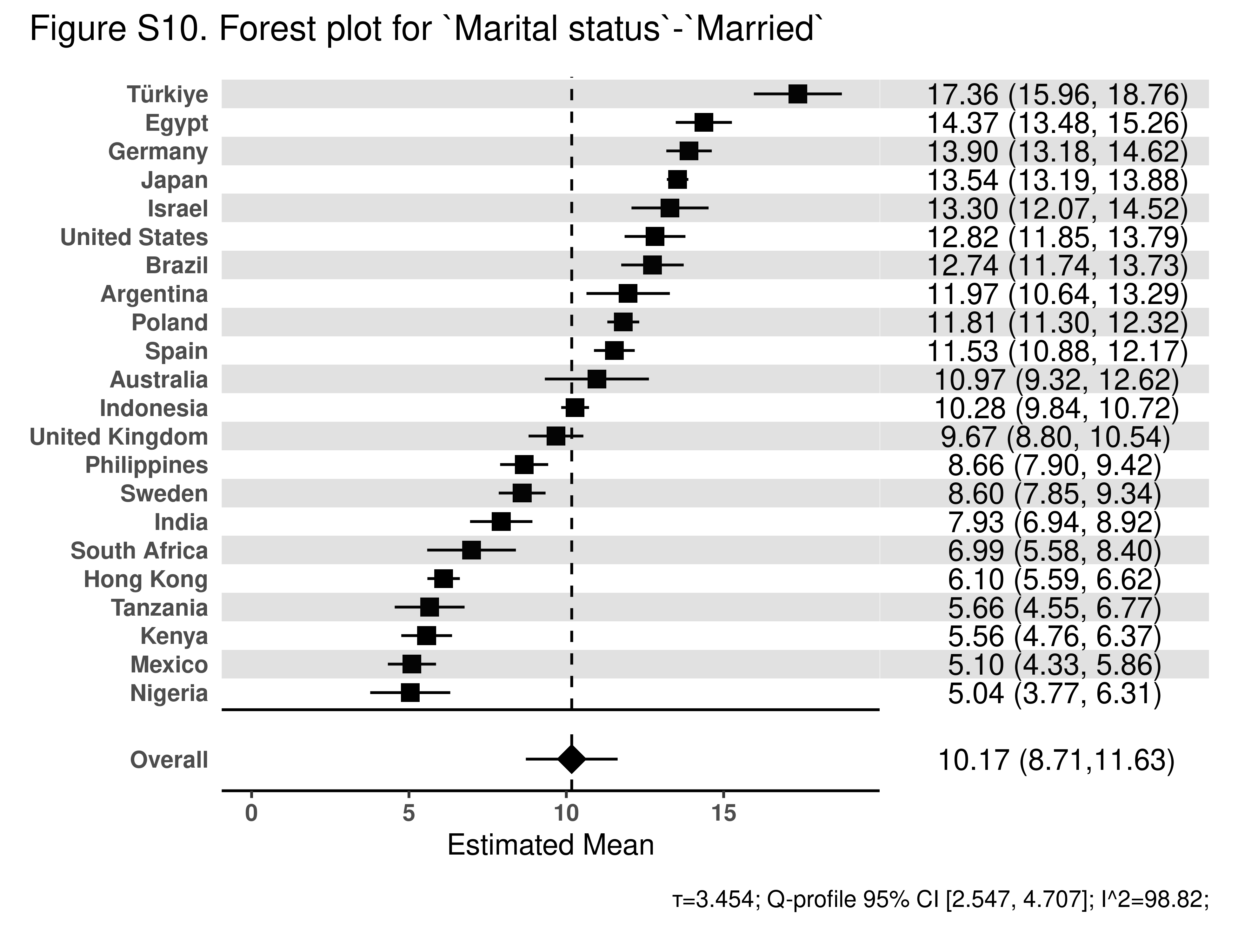

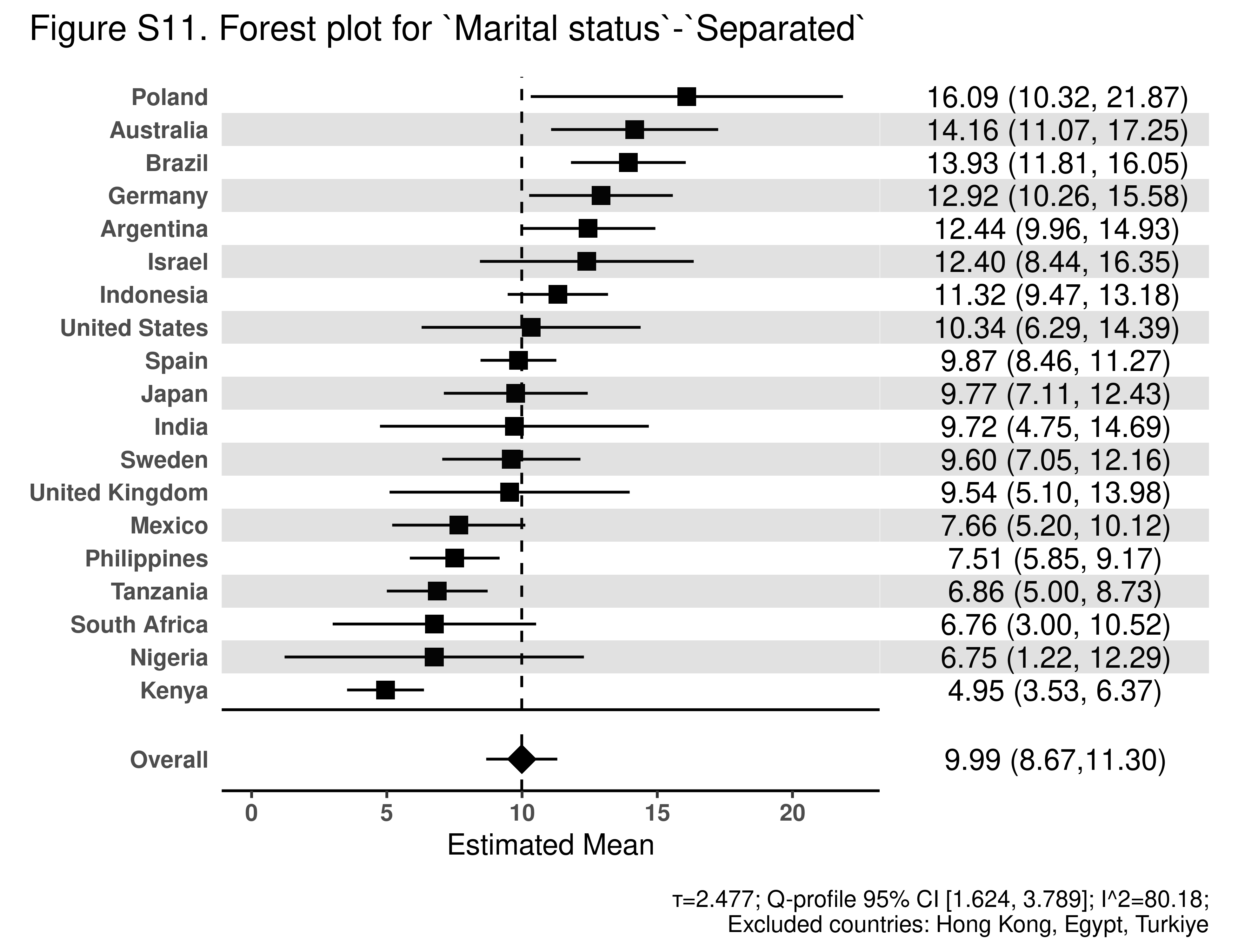

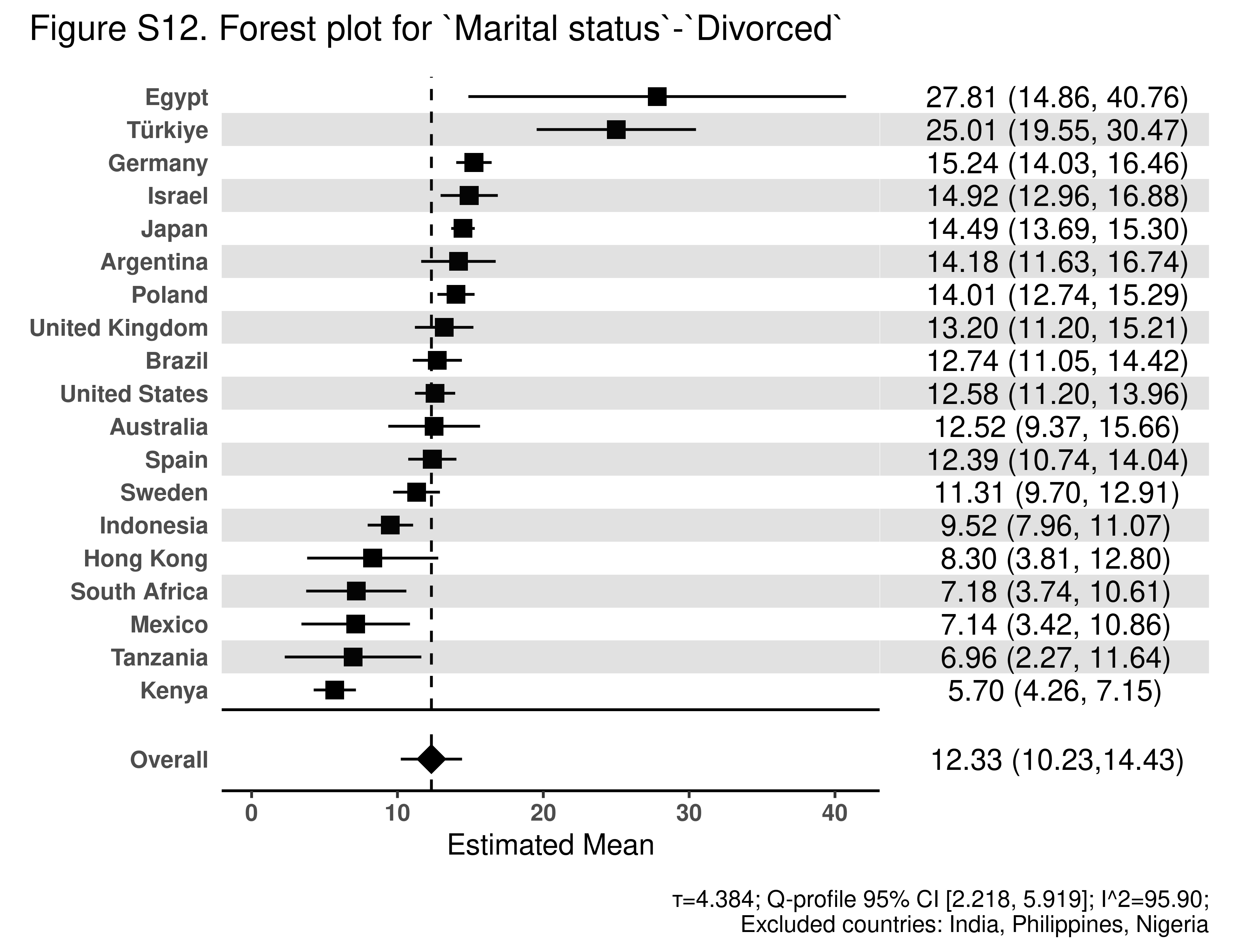

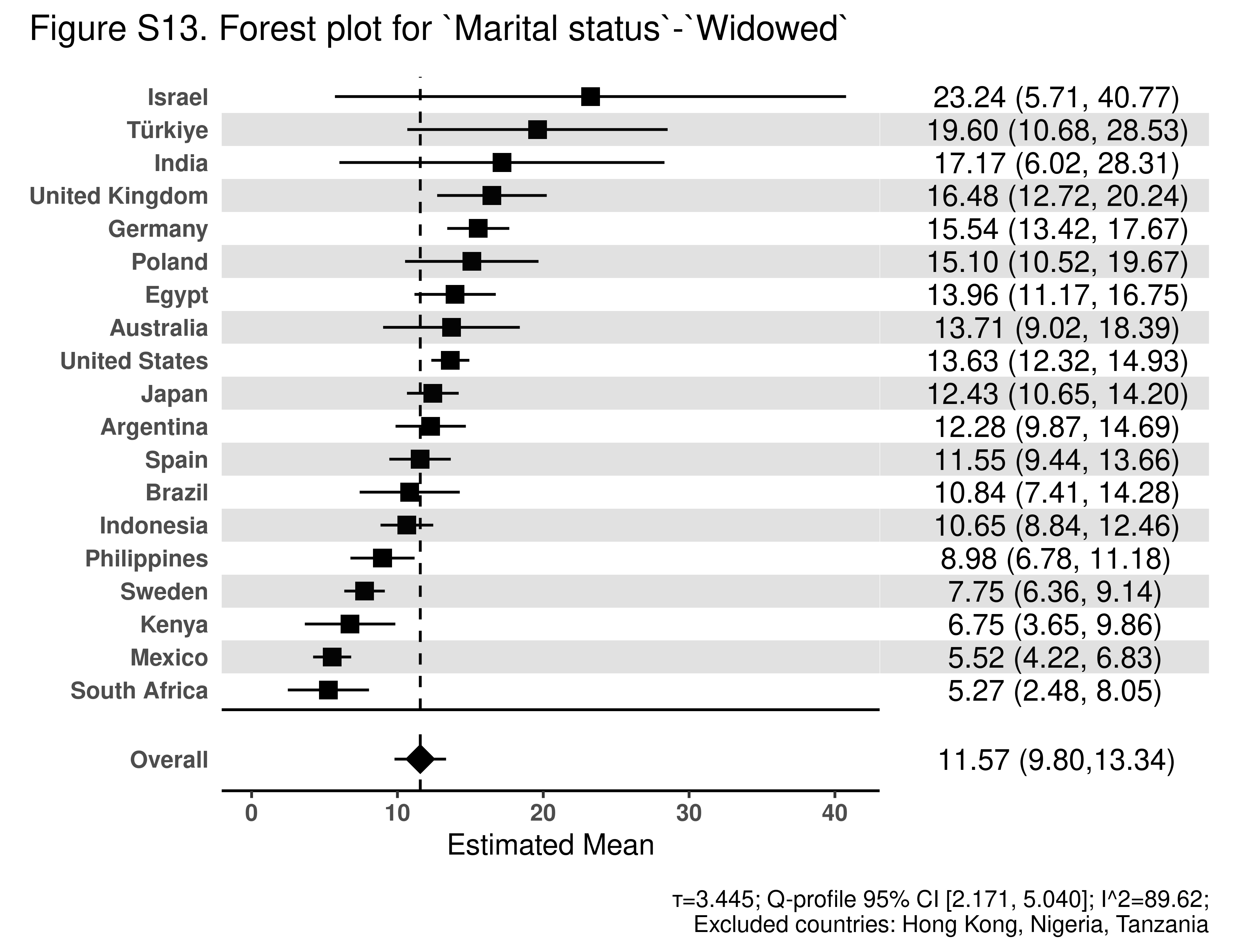

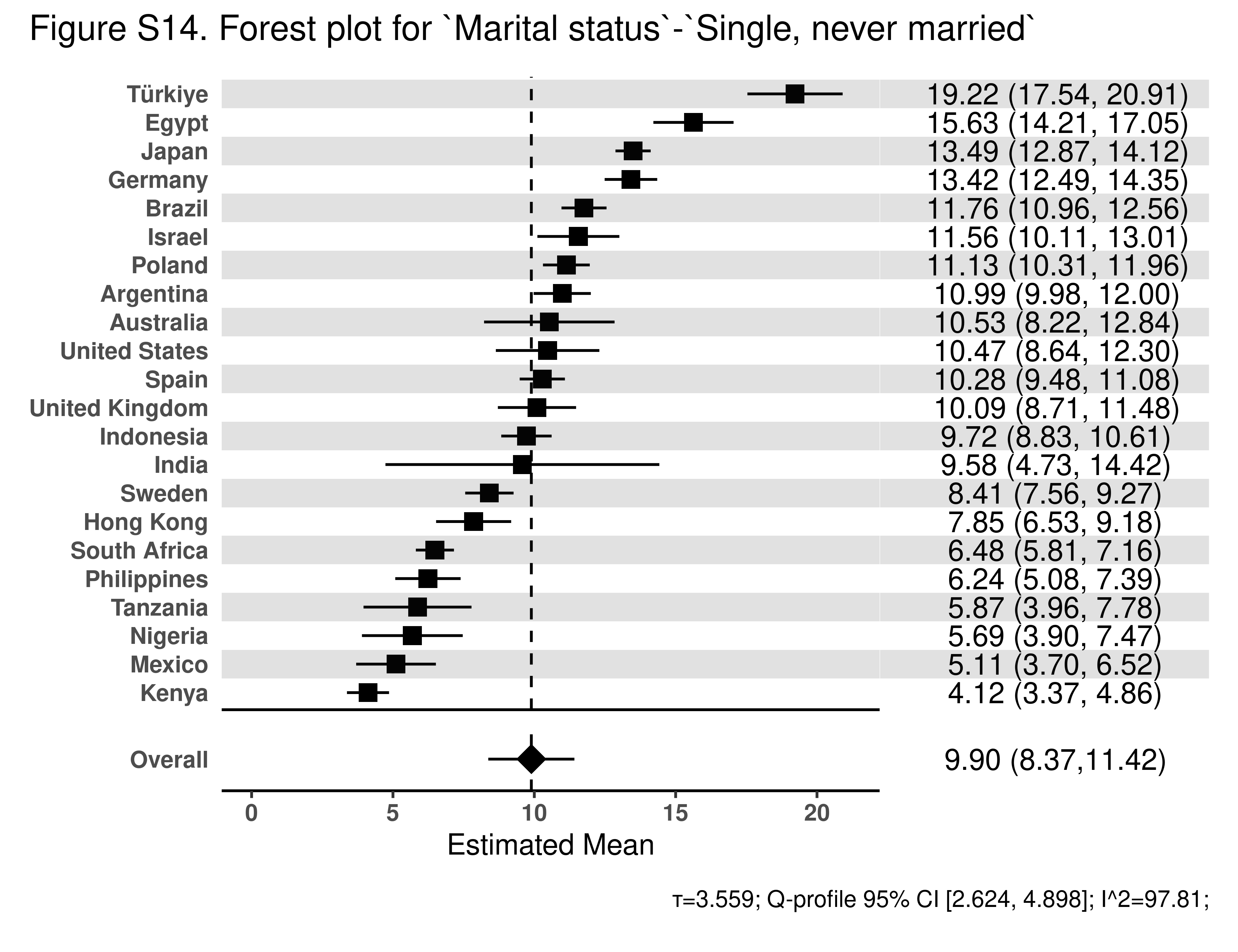

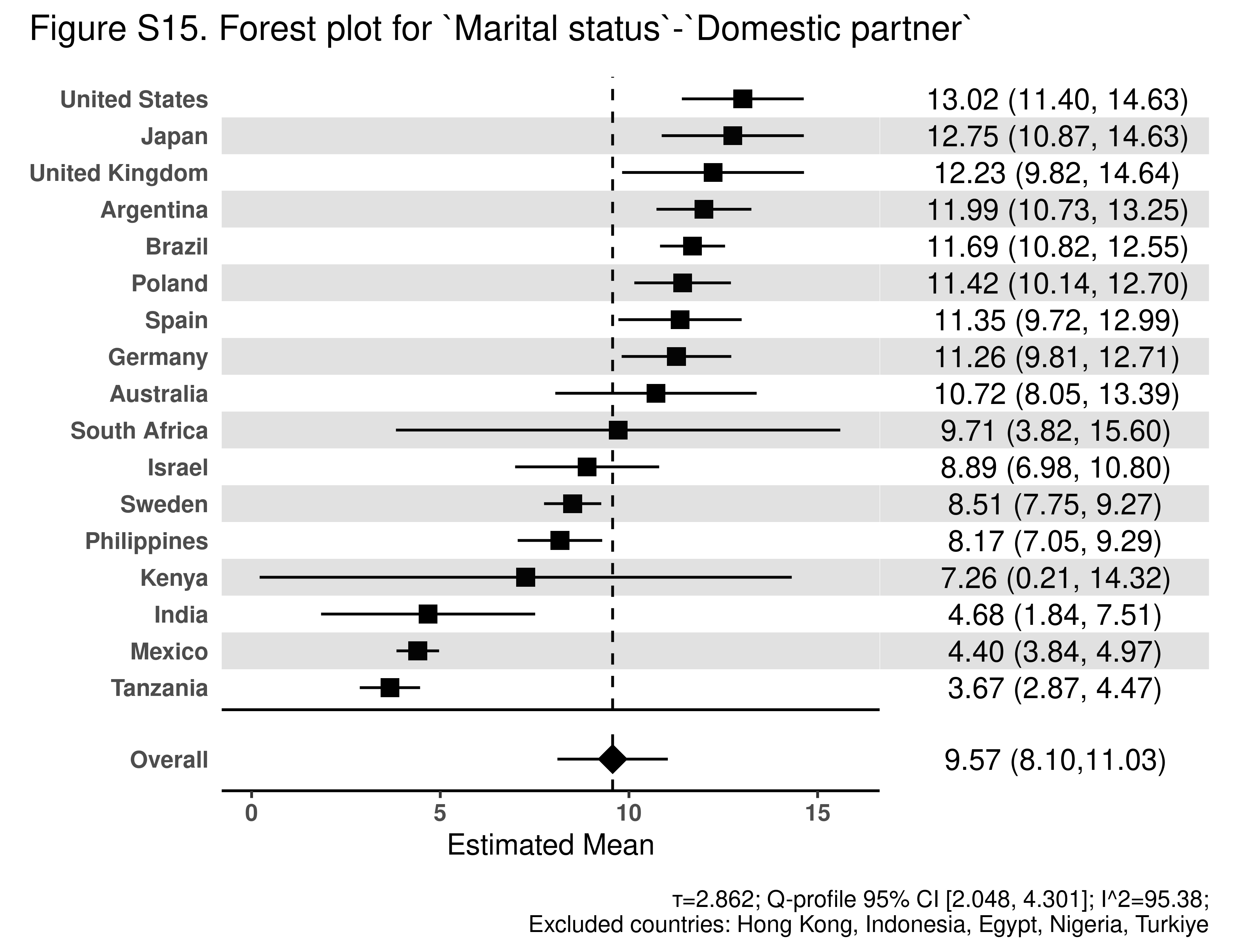

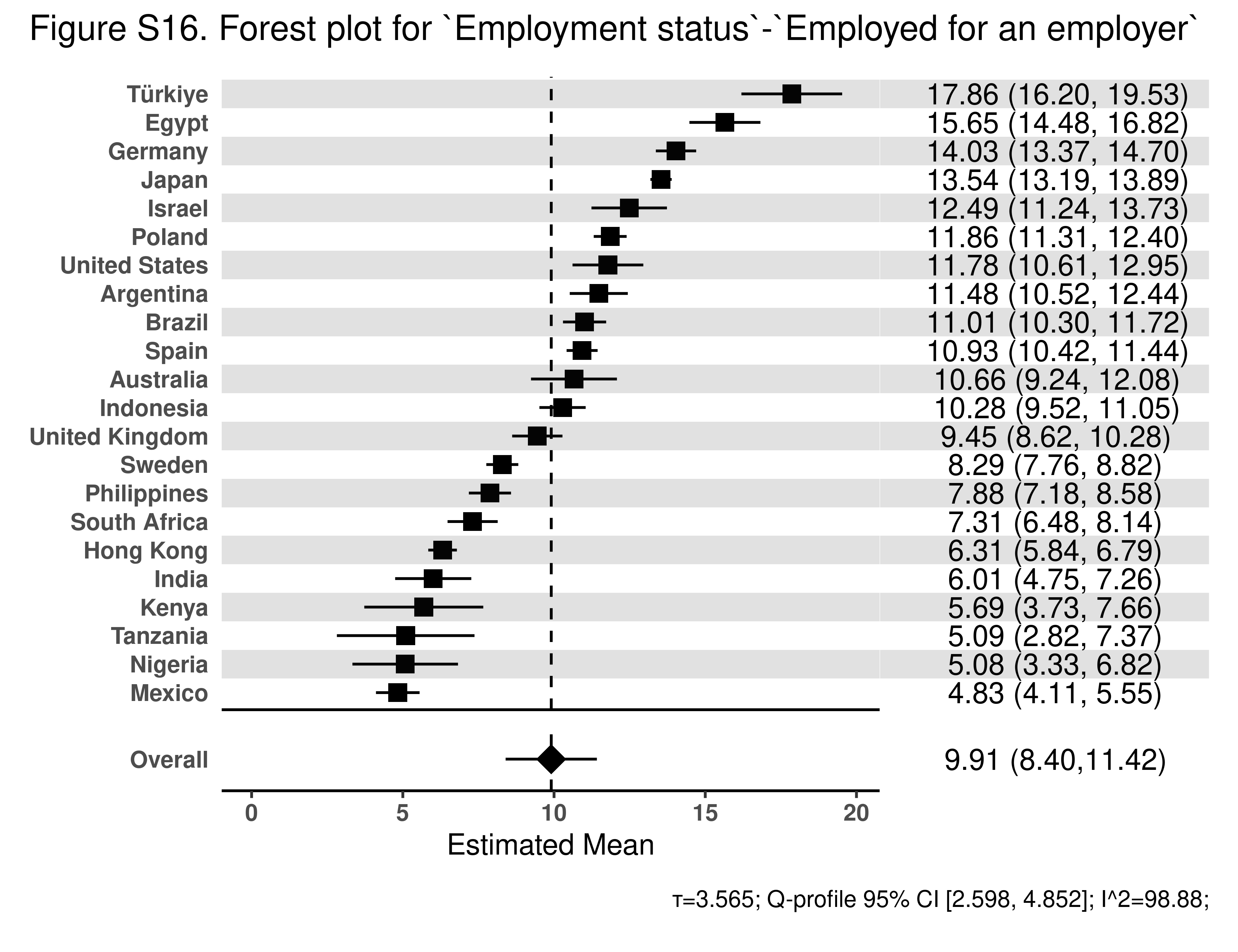

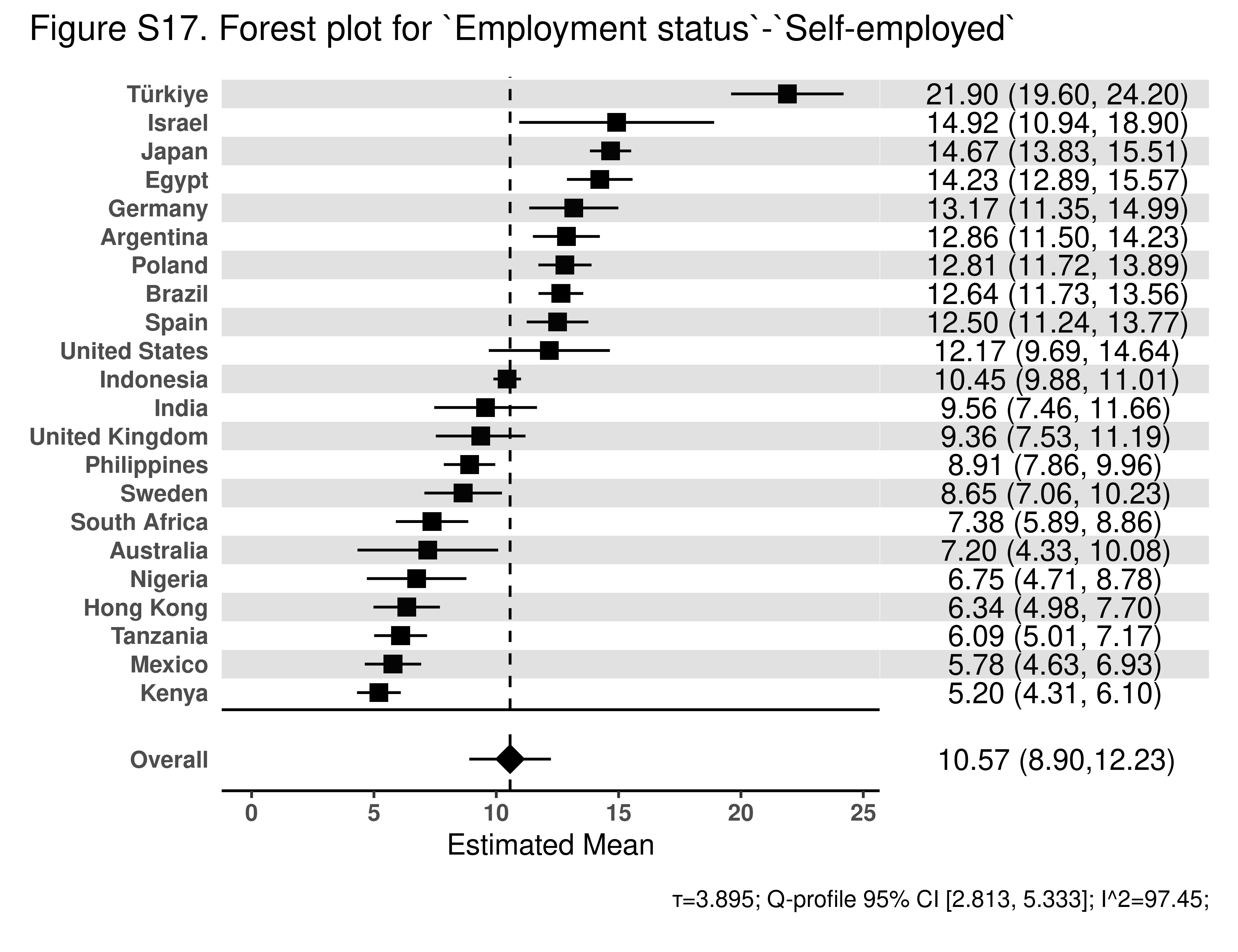

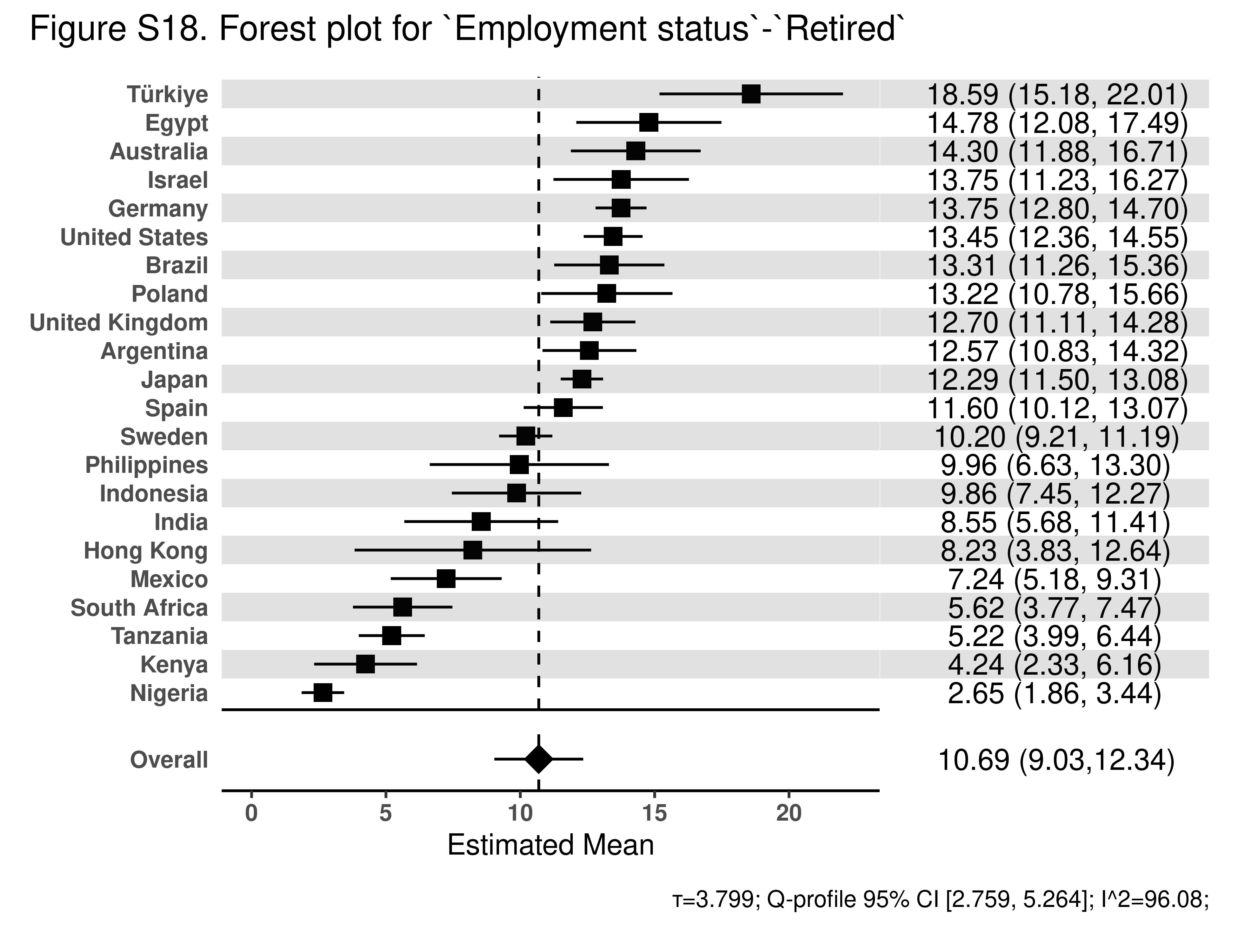

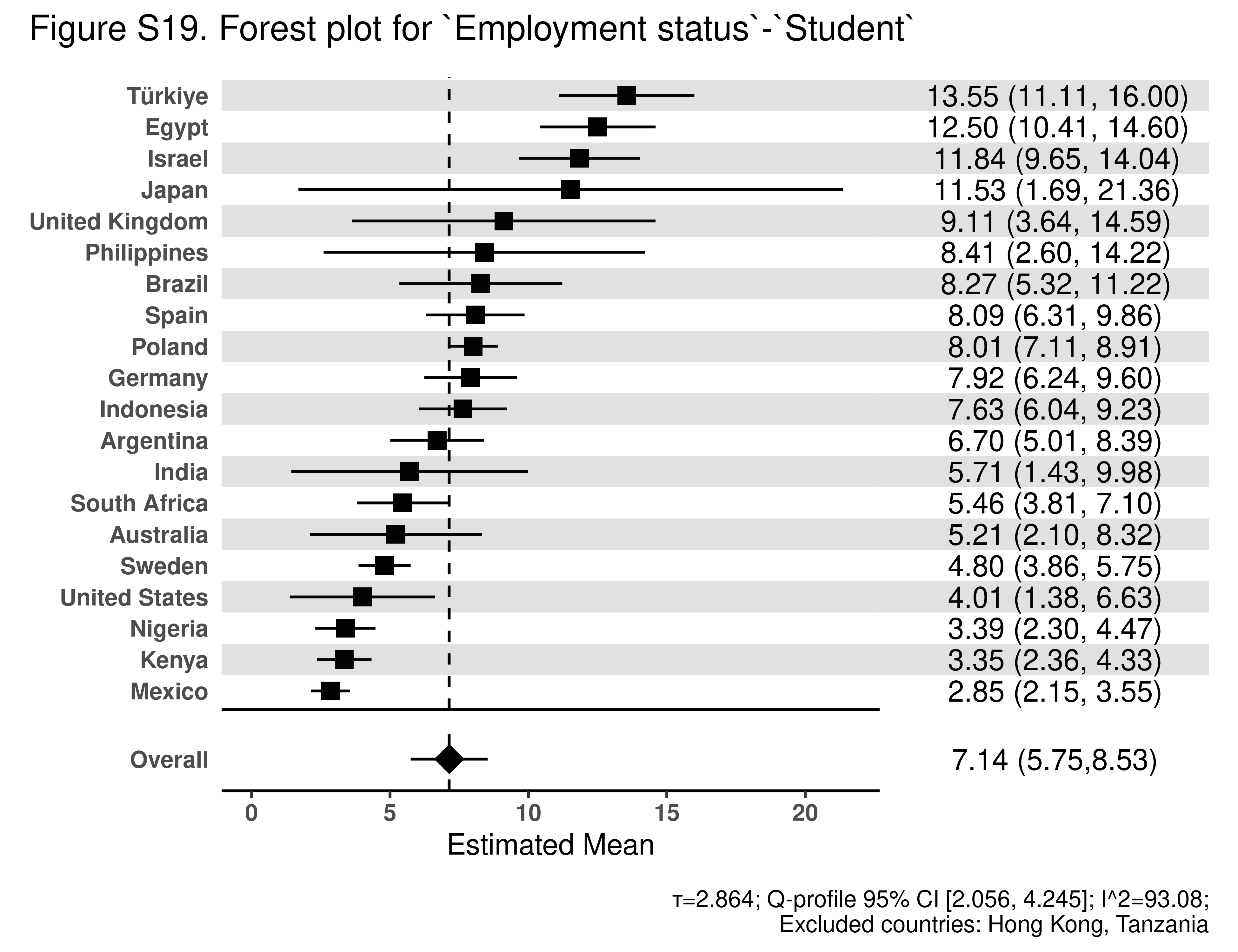

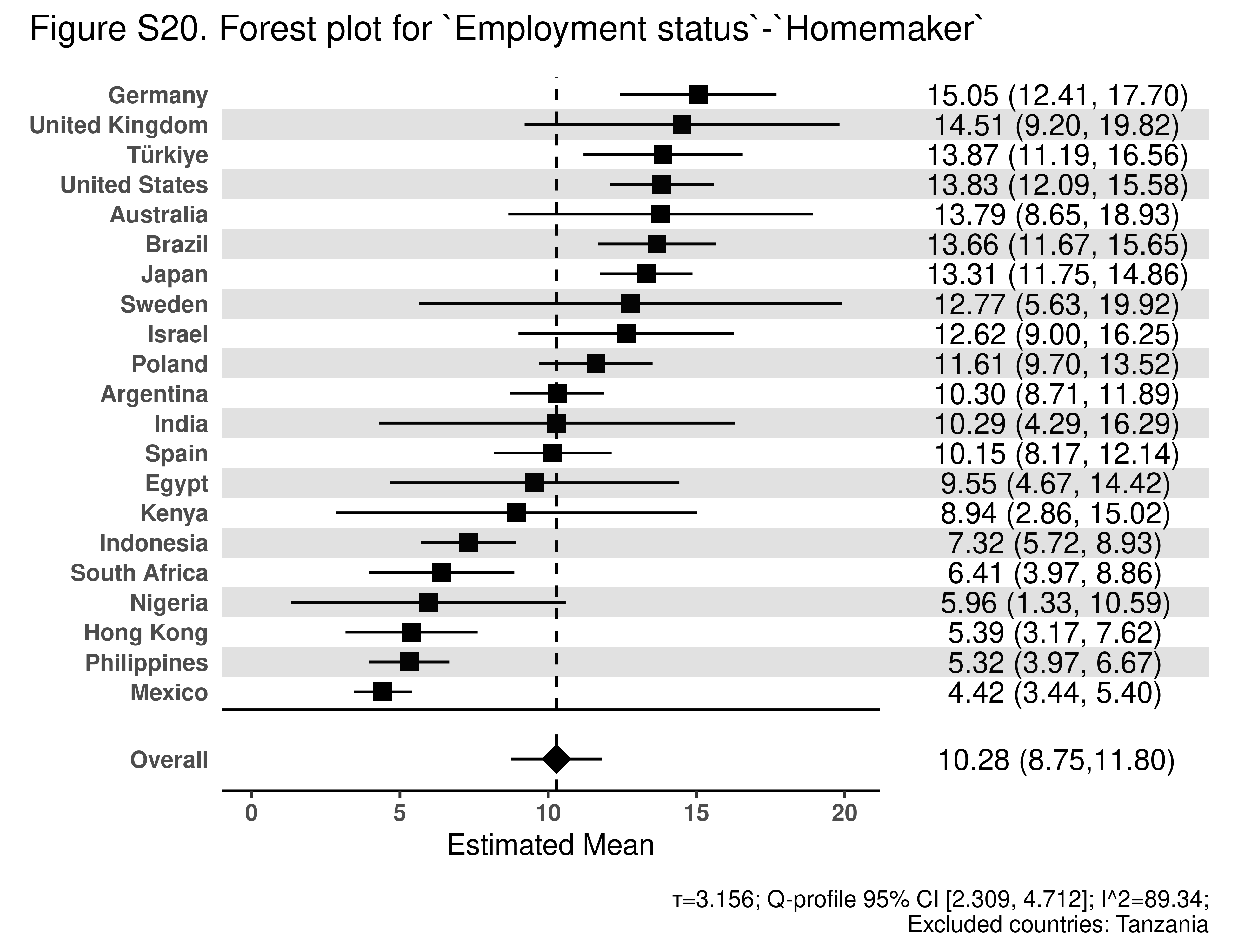

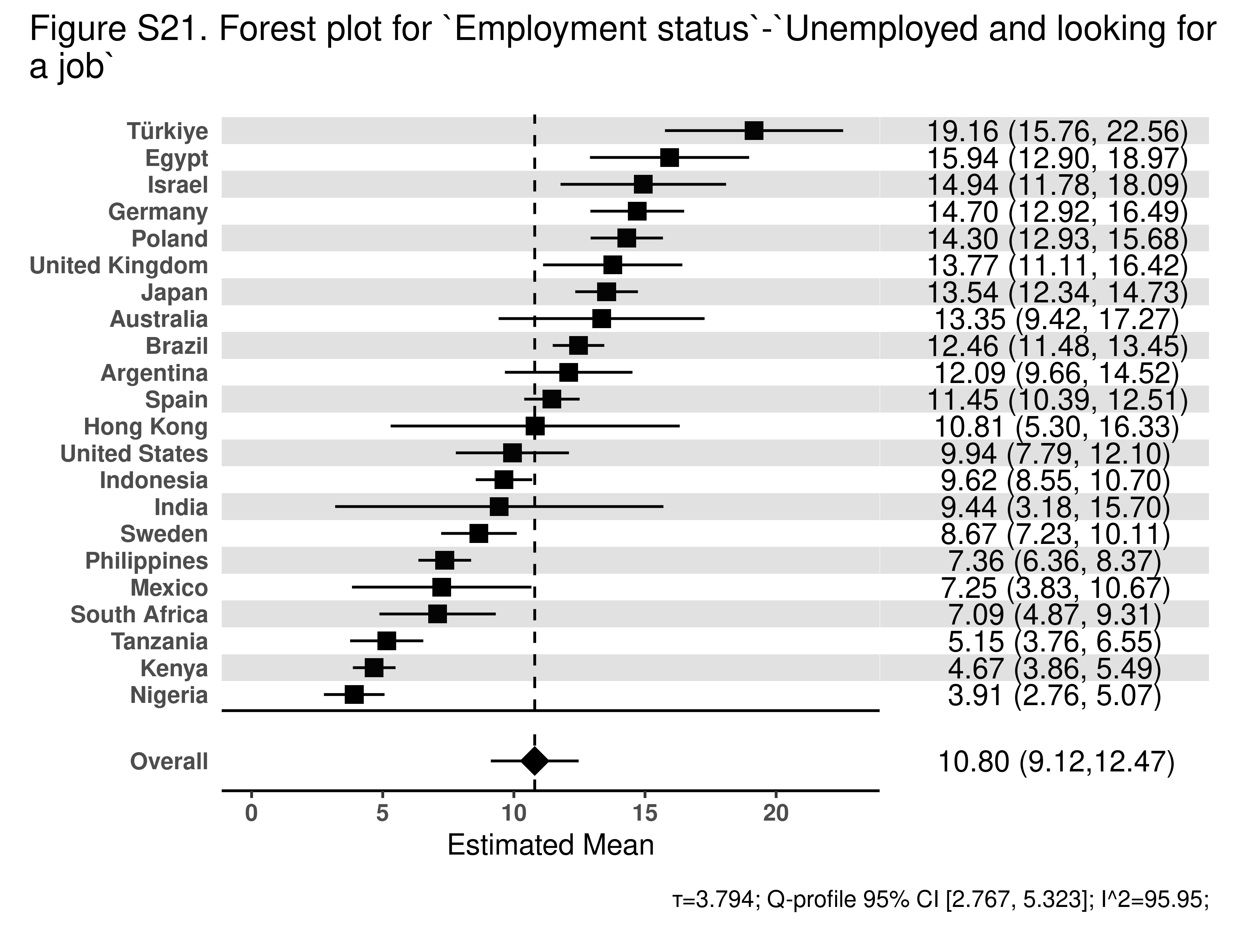

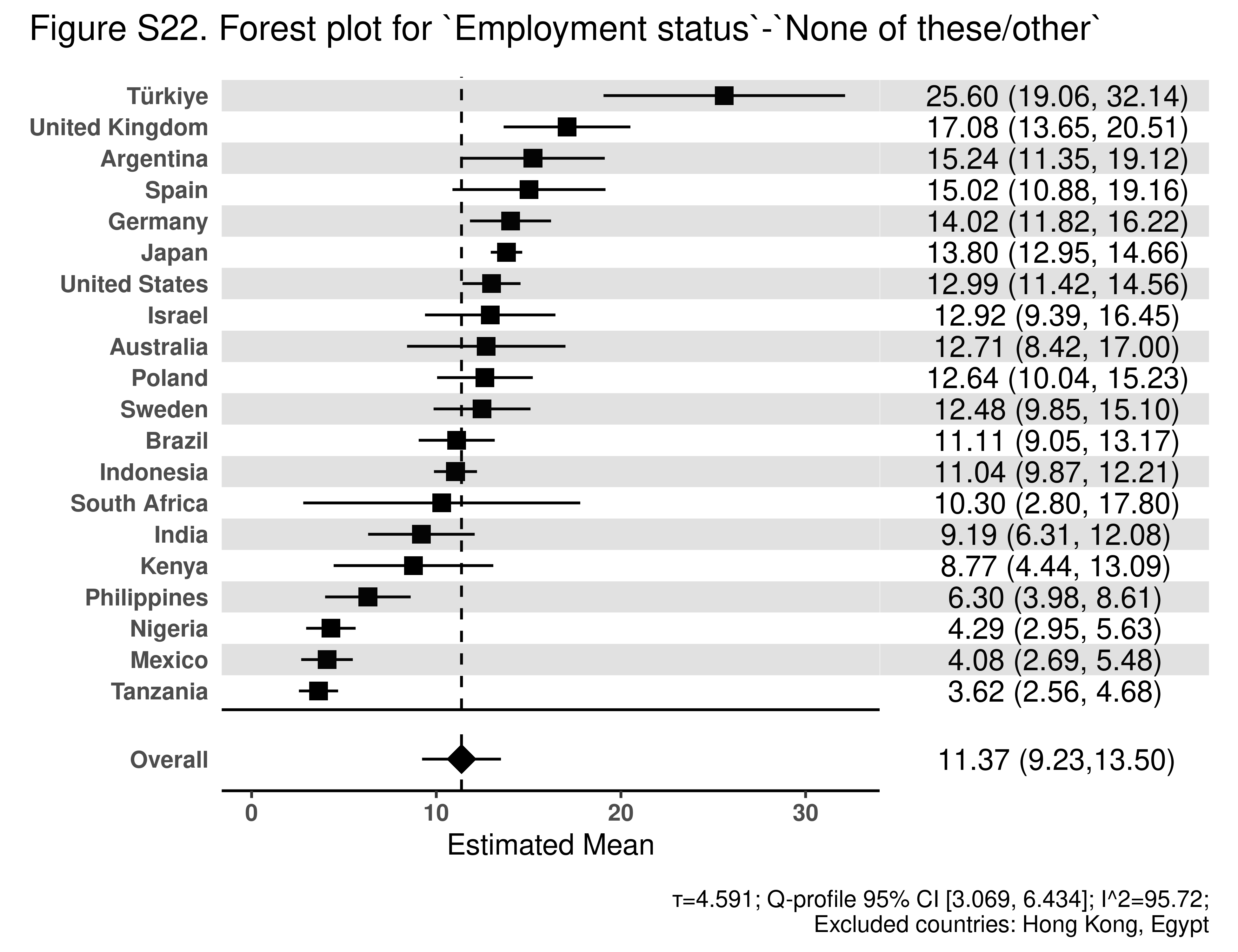

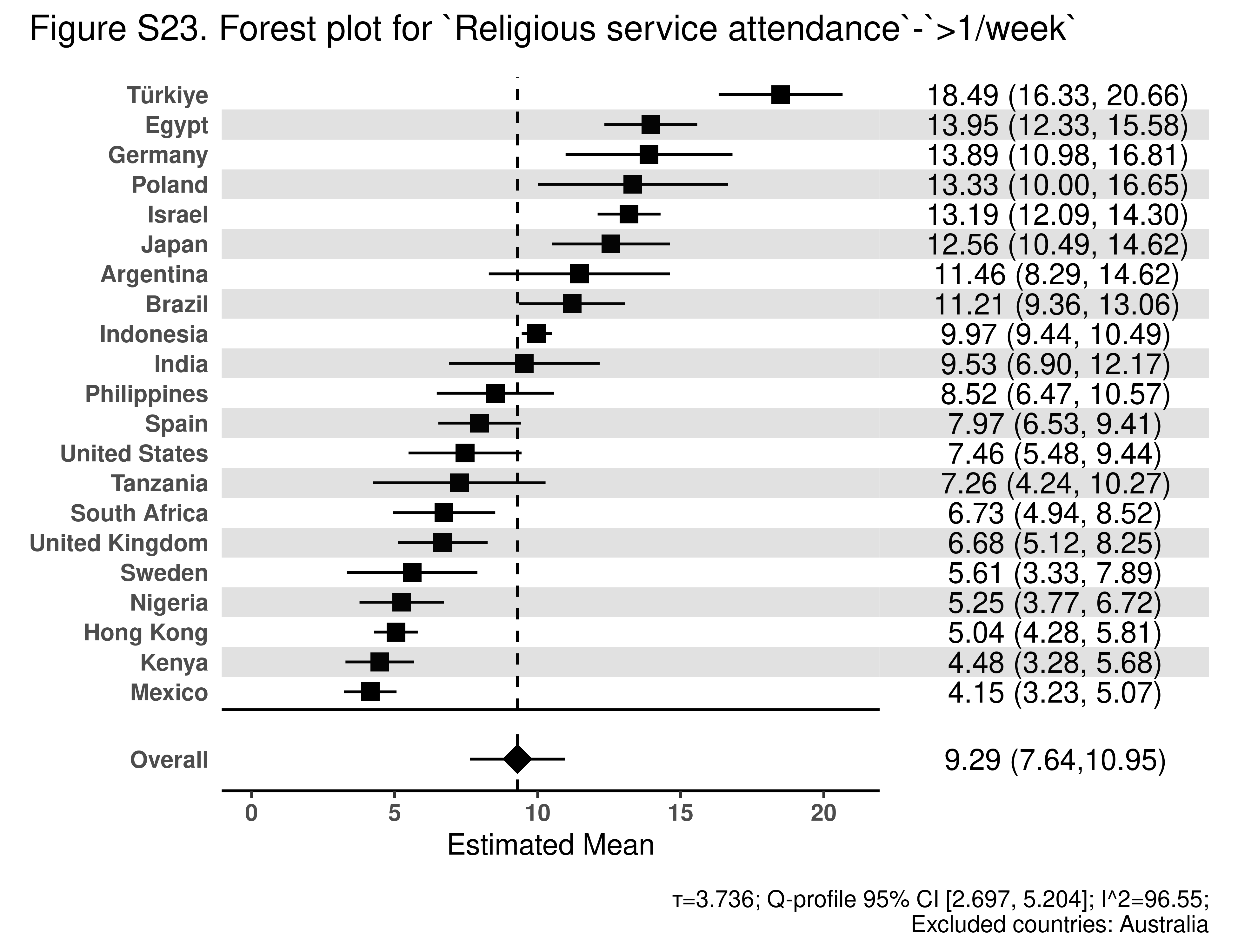

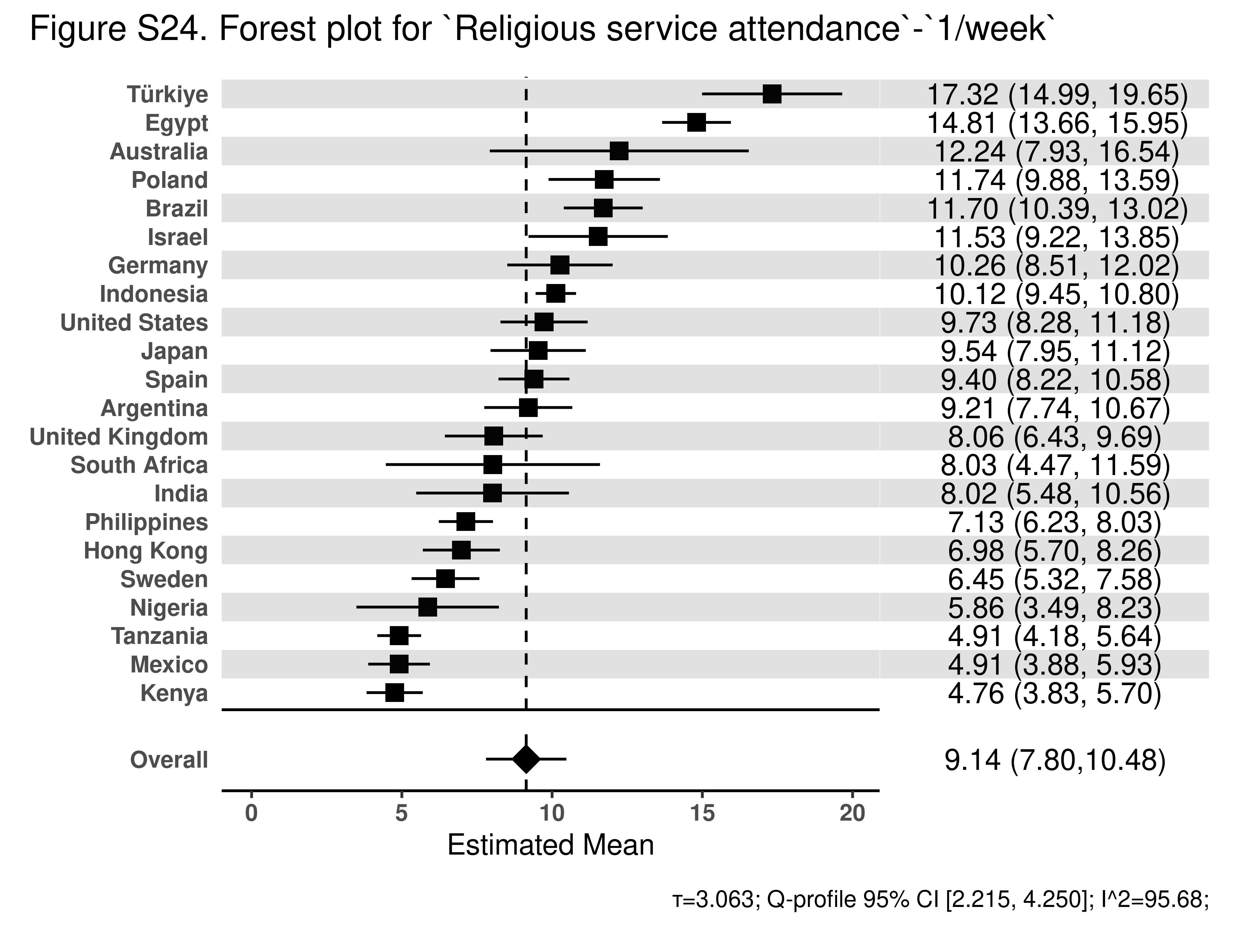

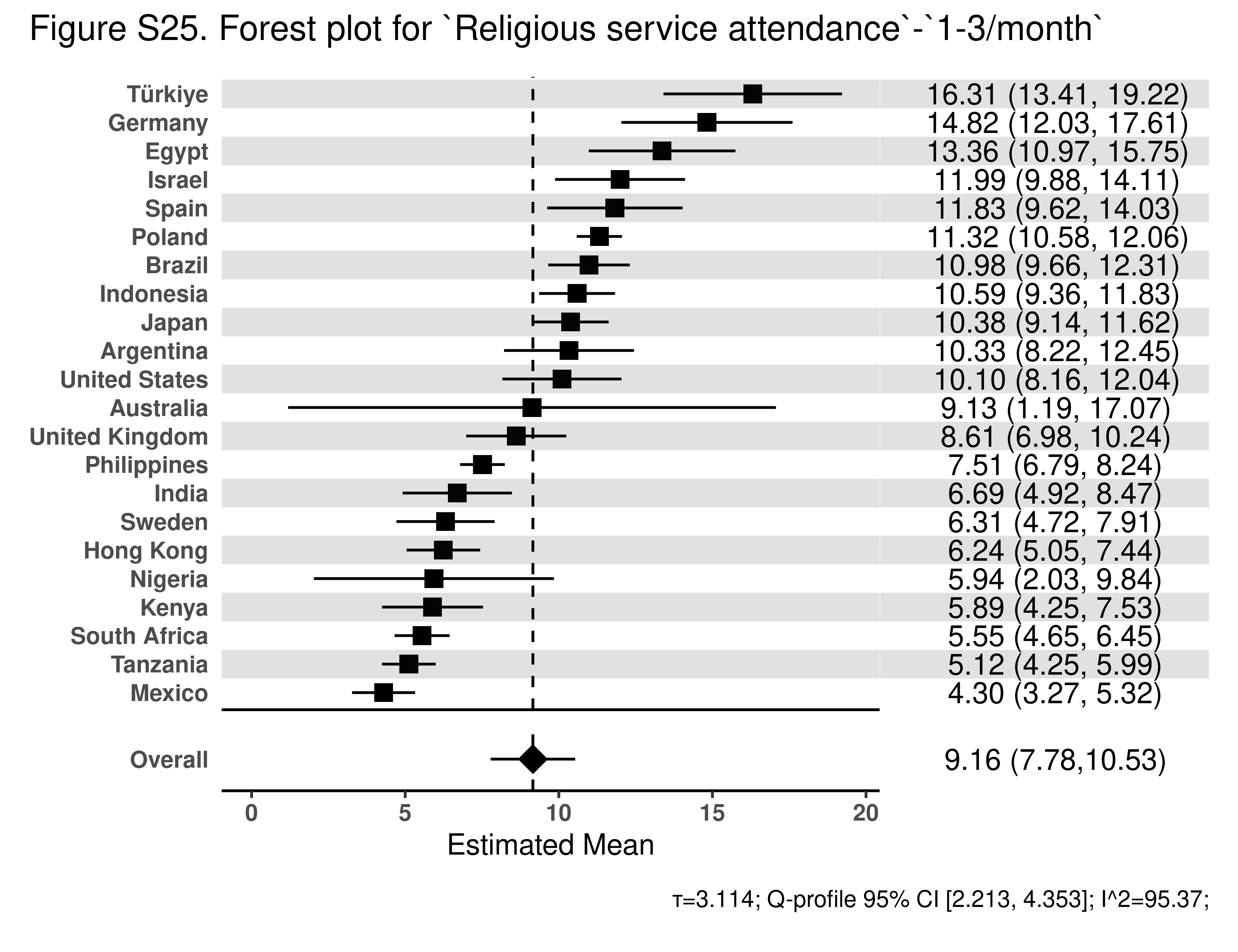

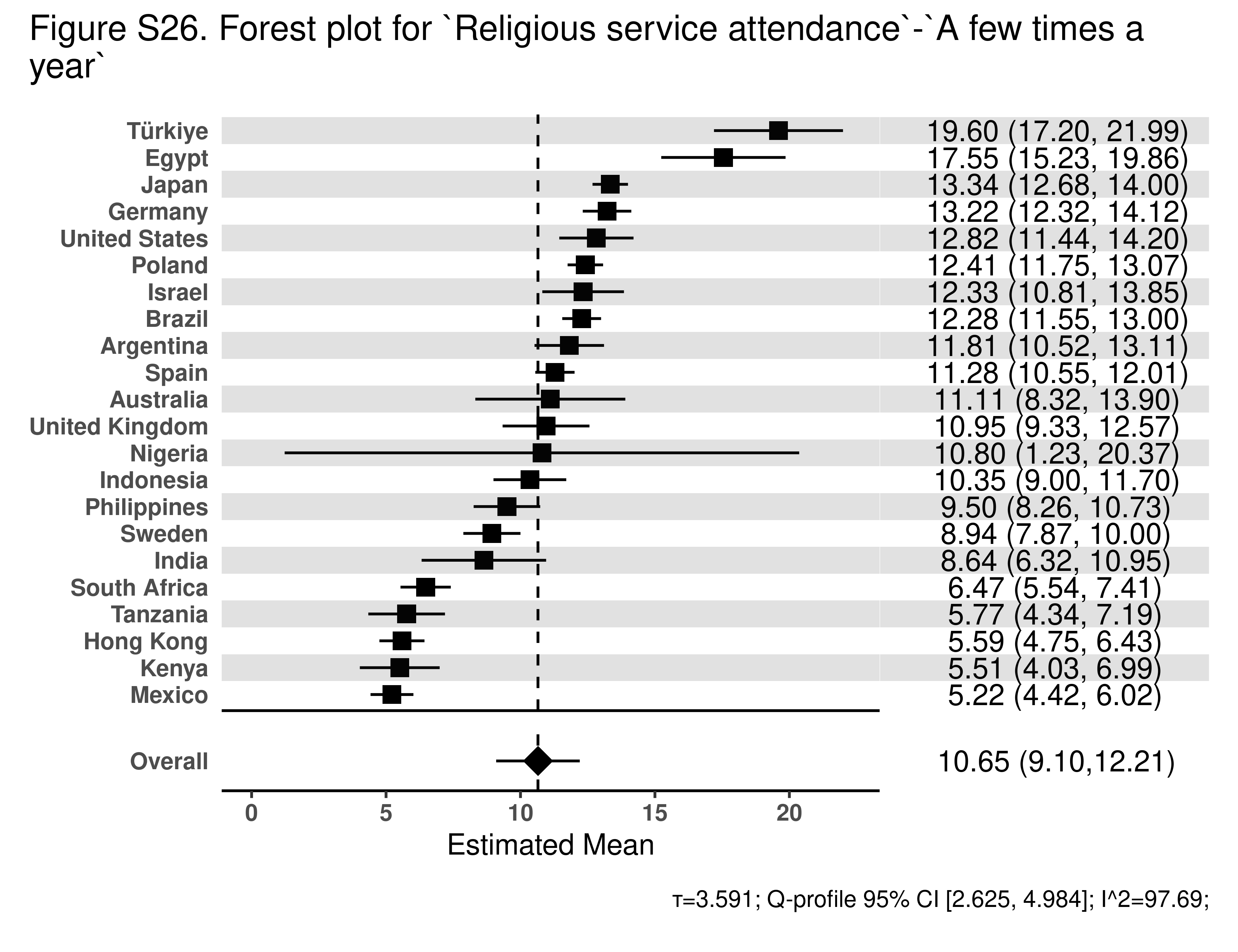

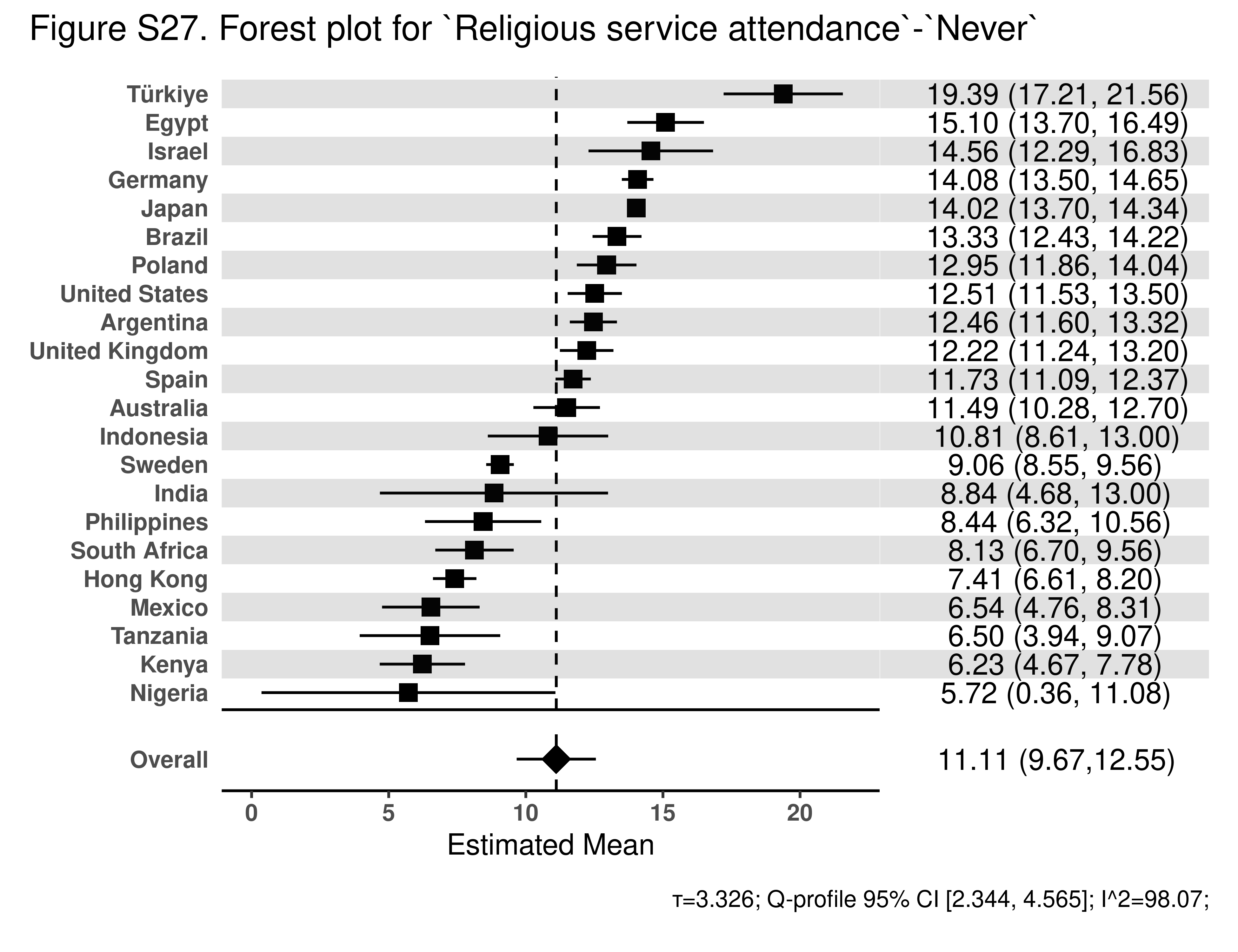

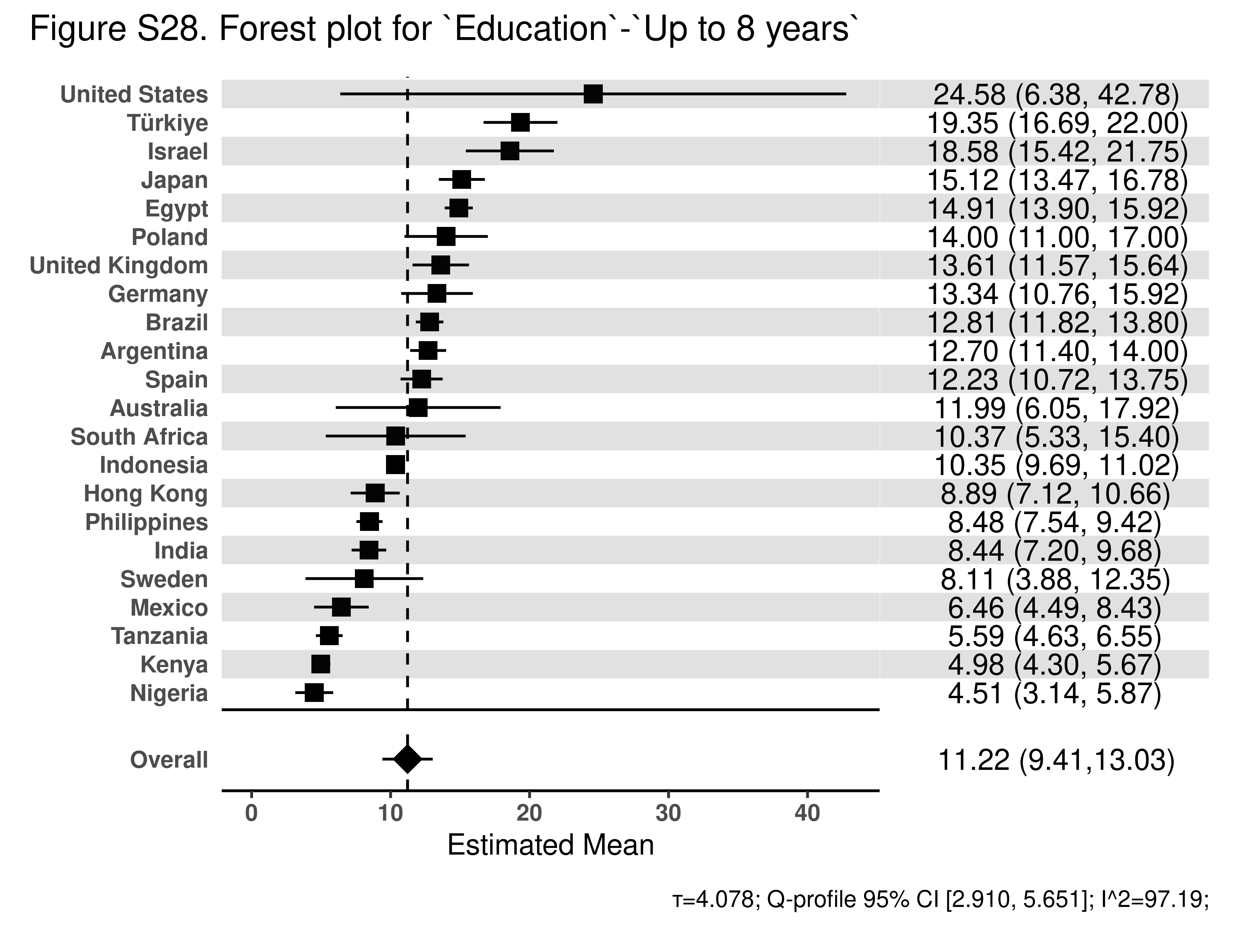

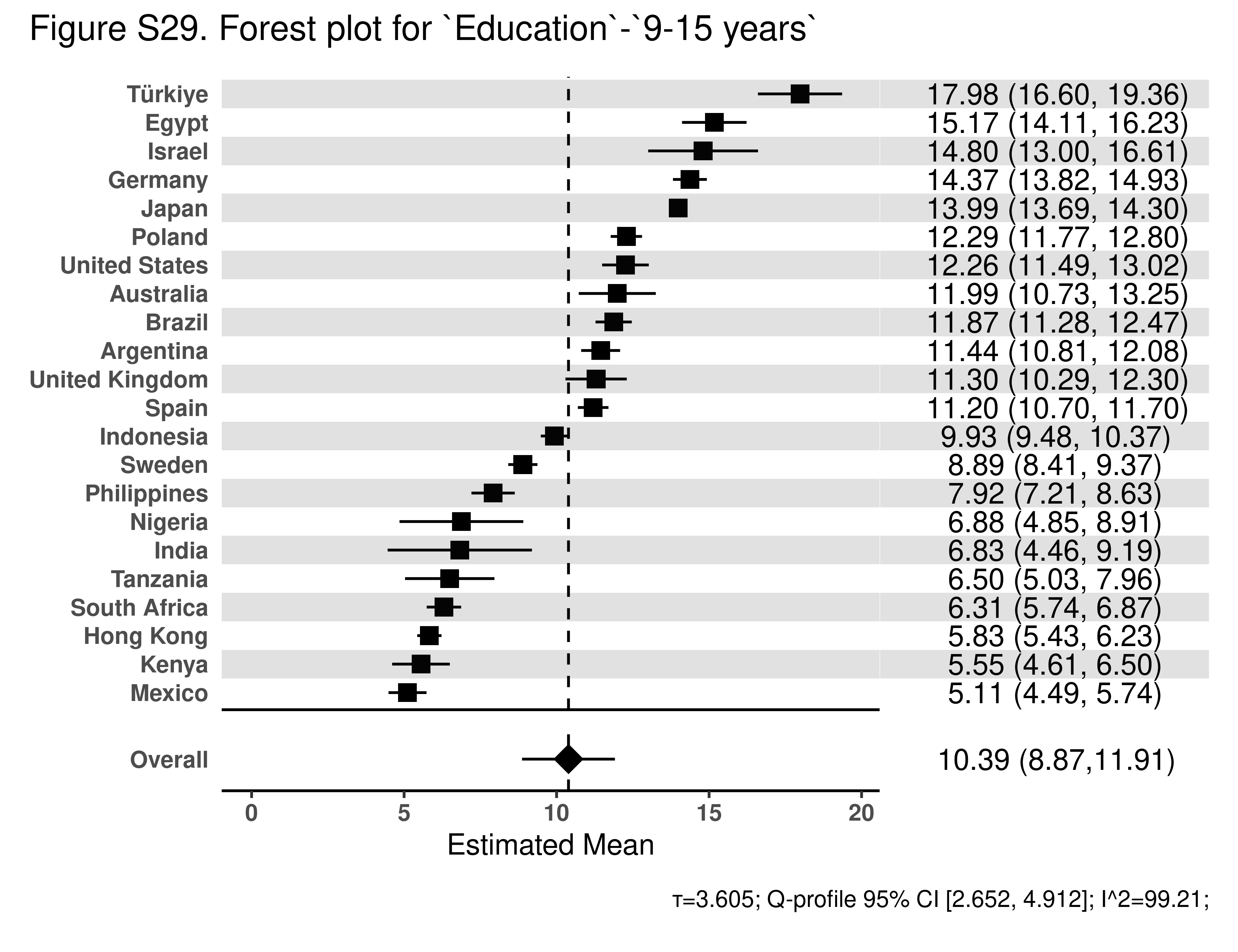

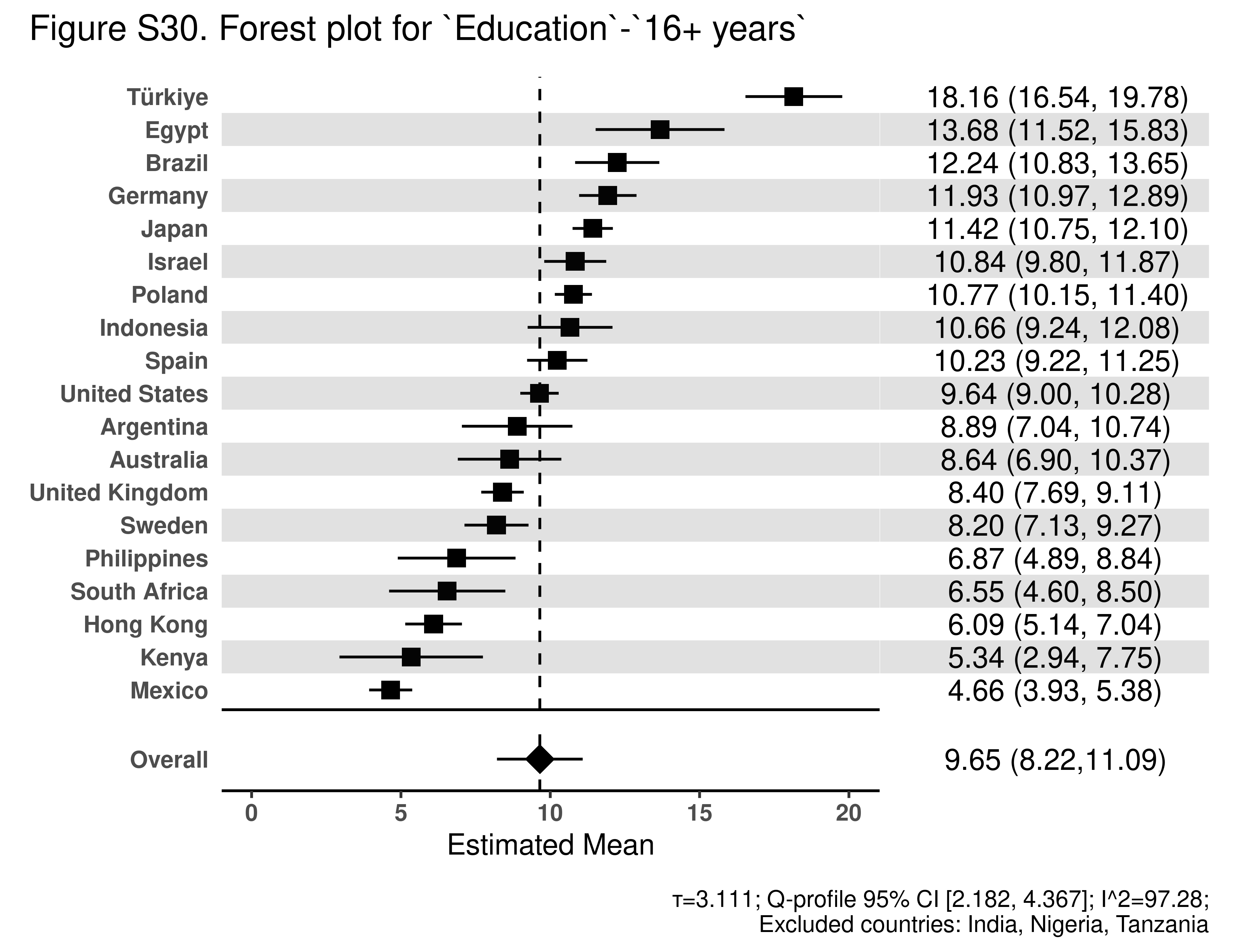

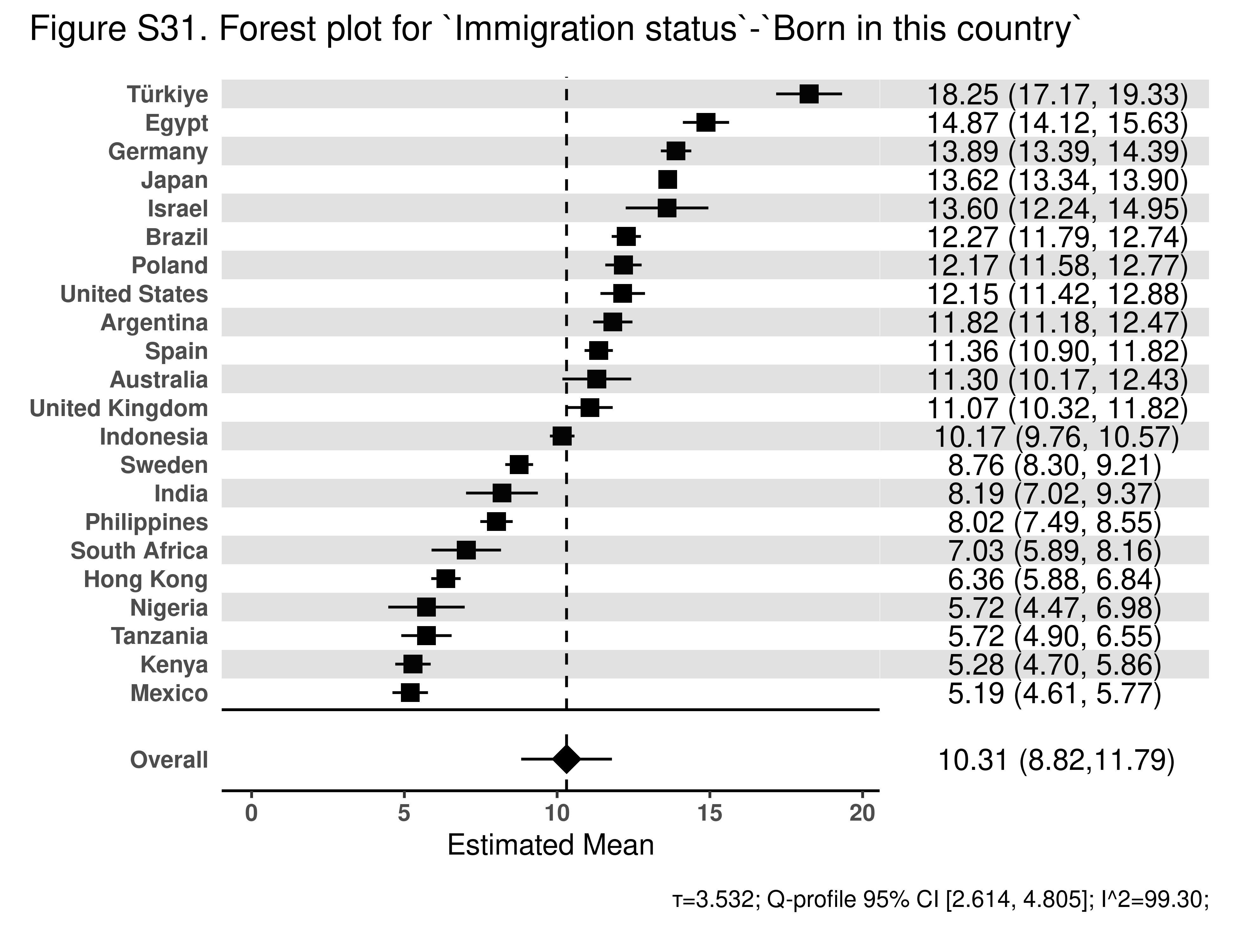

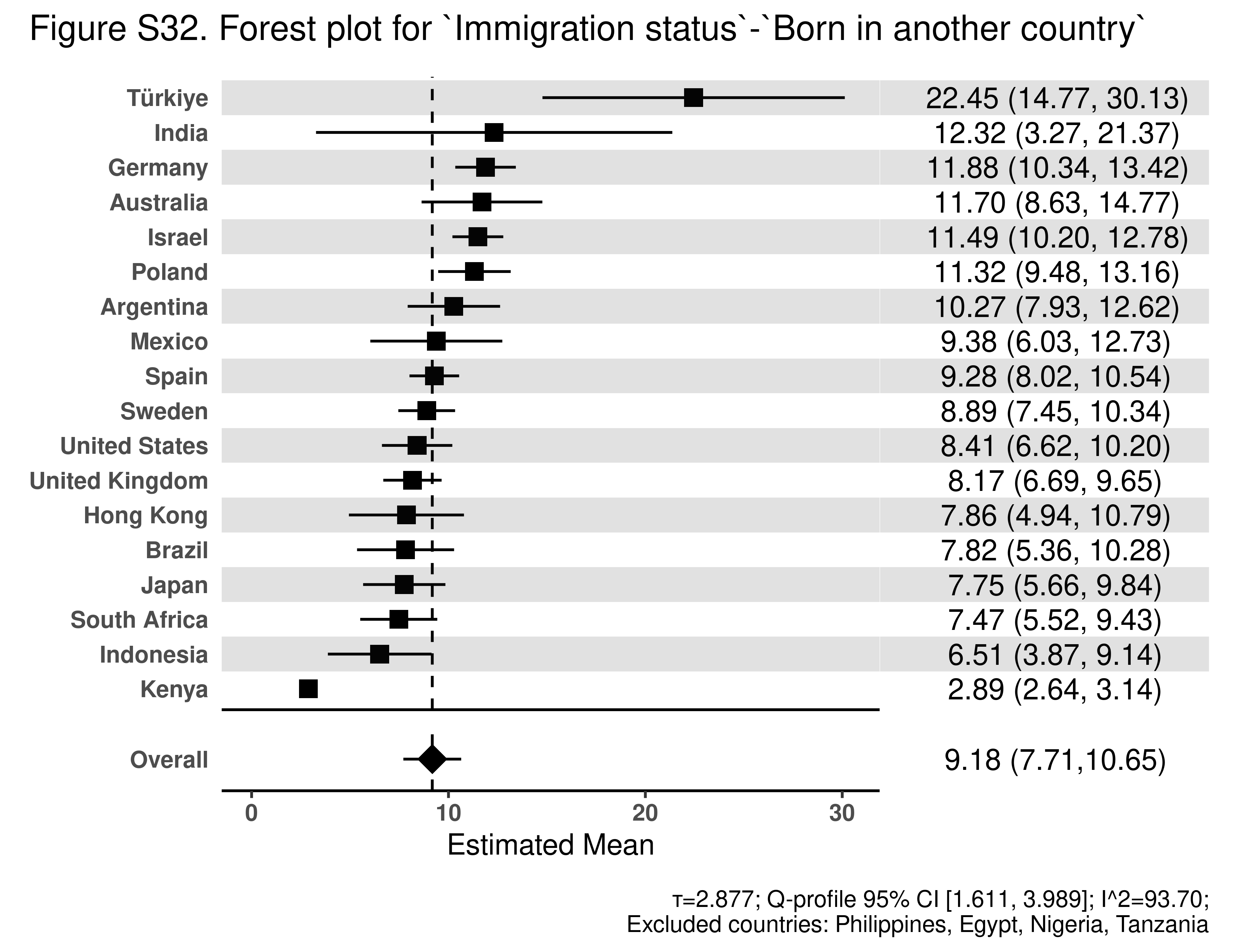

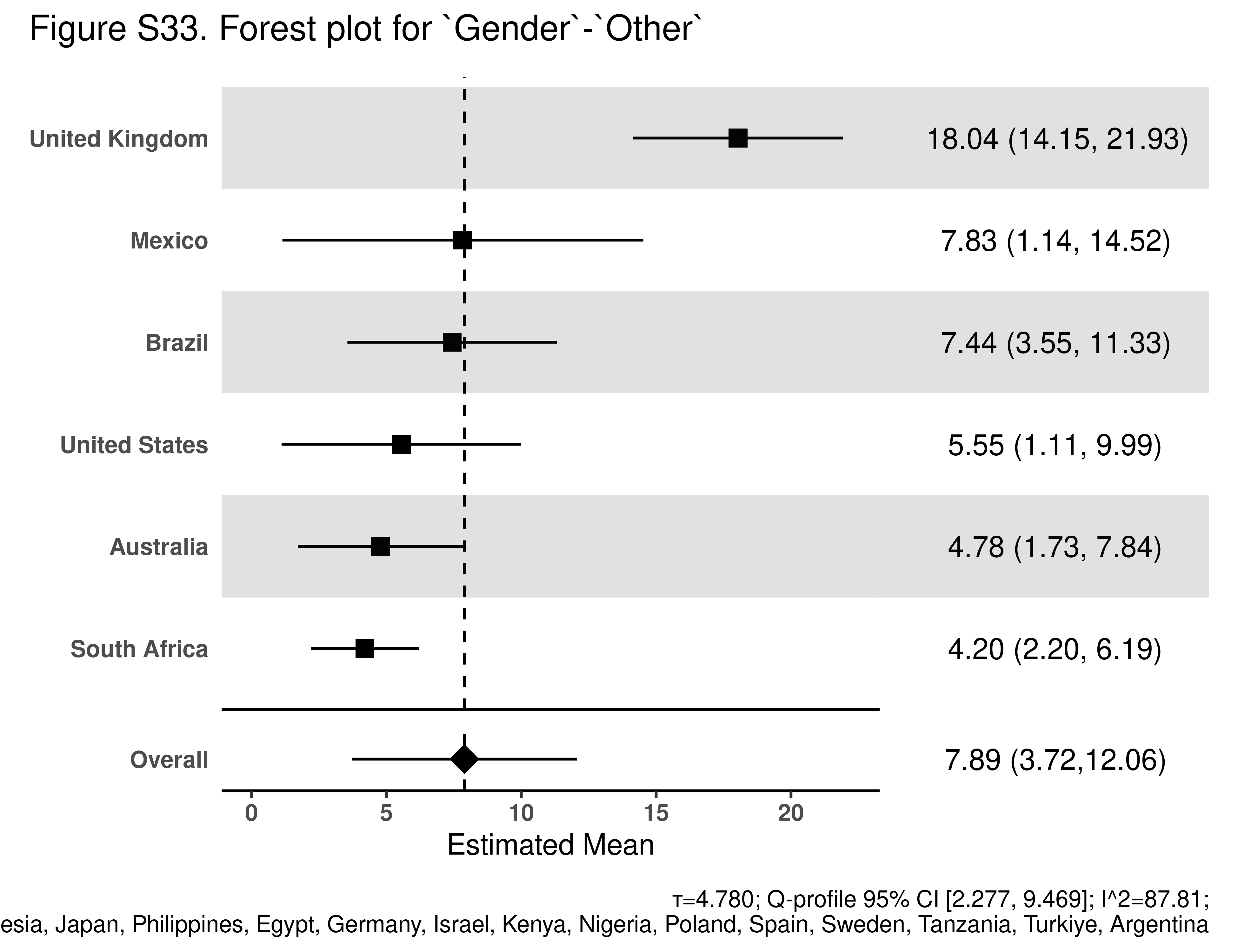

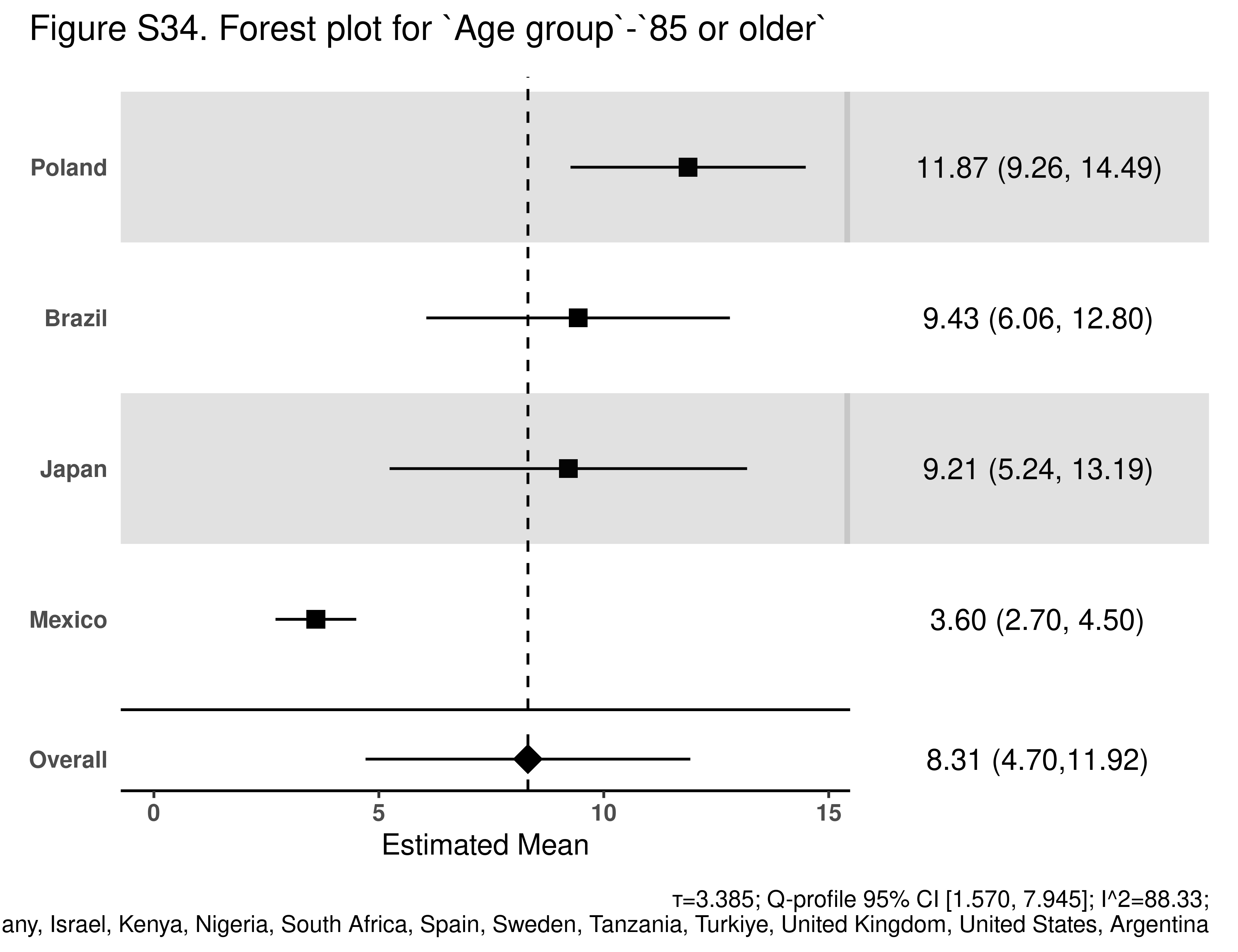


Intensity

**Figure S38.**

Intensity

**Figure S37.**

**Figure S39.**

Intensity

**Figure S40.**

Intensity

**Figure S41.**

Intensity

**Figure S42.**

Intensity

**Figure S43.**

Intensity

**Figure S44.**

Intensity

**Figure S46.**

Intensity

**Figure S45.**

Intensity

Intensity

**Figure S47.**

**Figure S48.**

Intensity

**Figure S50.**

Intensity

**Figure S49.**

Intensity

**Figure S52.**

Intensity

**Figure S51.**

Intensity

**Figure S54.**

Intensity

**Figure S53.**

Intensity

**Figure S56.**

Intensity

**Figure S55.**

Intensity

**Figure S58.**

Intensity

**Figure S57.**

Intensity

**Figure S60.**

Intensity

**Figure S59.**

Intensity

**Figure S62.**

Intensity

**Figure S61.**

Intensity

**Figure S64.**

Intensity

**Figure S63.**

Intensity

**Figure S66.**

Intensity

**Figure S65.**

Intensity

**Figure S68.**

Intensity

**Figure S67.**

Intensity

**Figure S48.**

Intensity

**Figure S46.**

# Figures S69-S102. Forest plots of Proportion for Demographic Categories


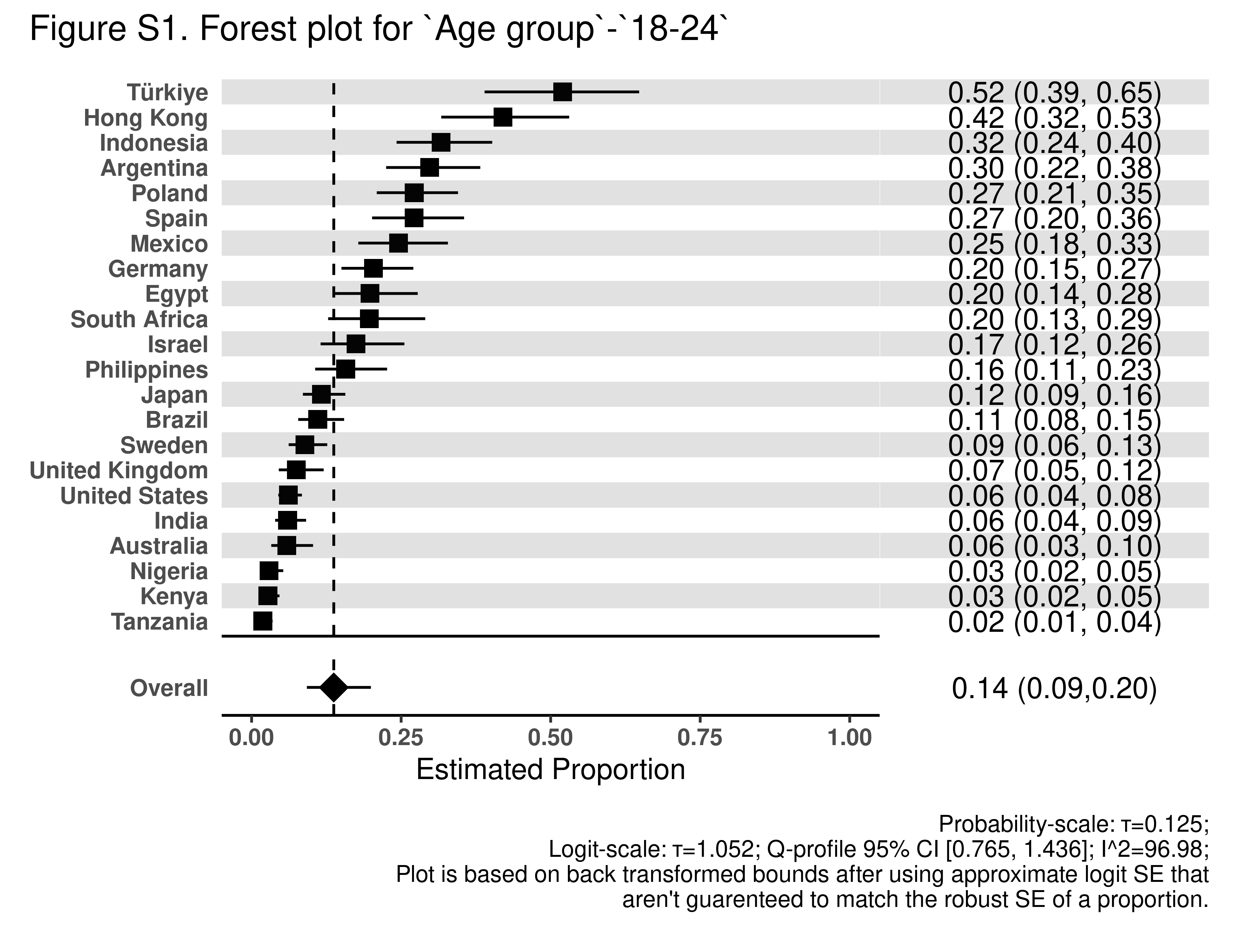

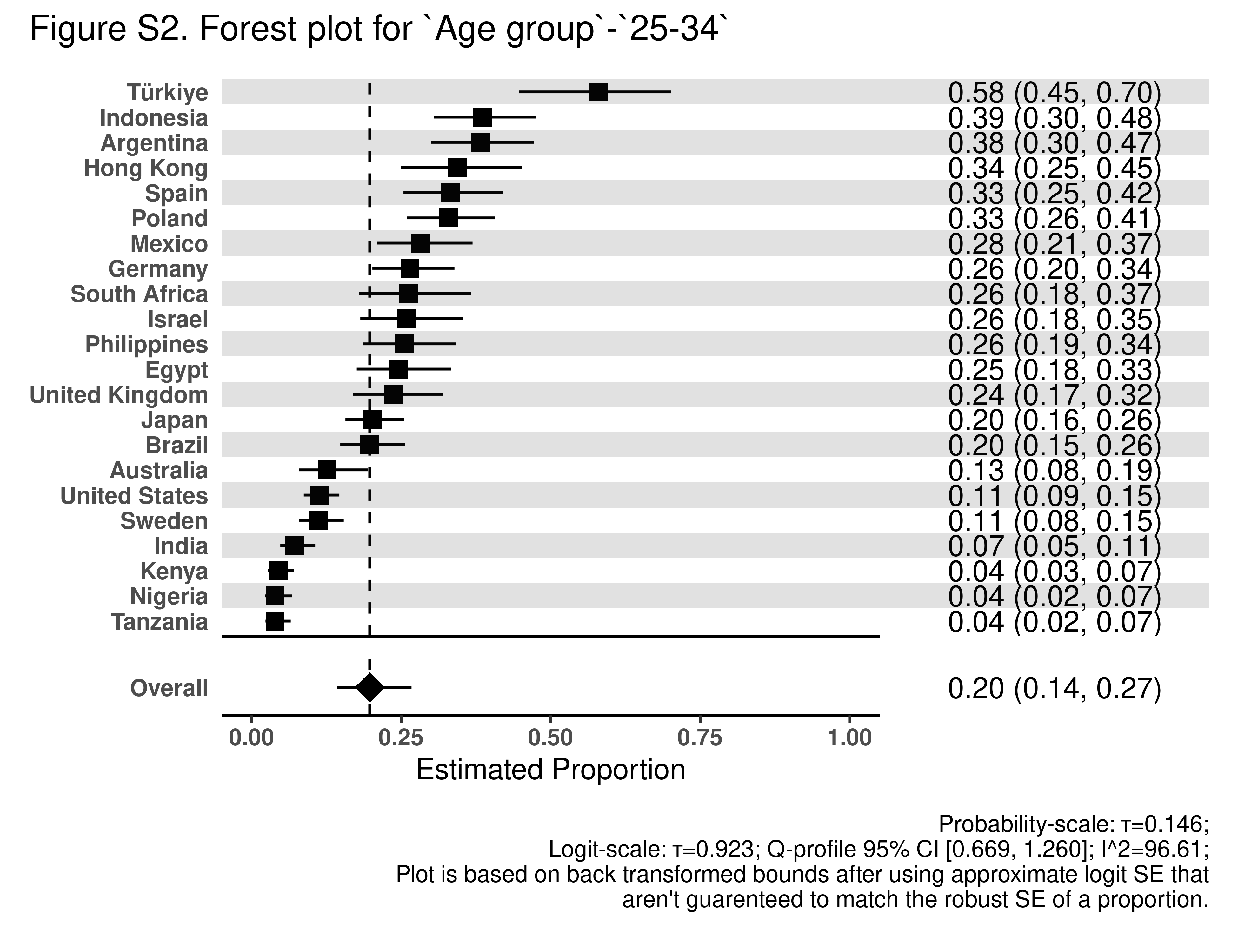

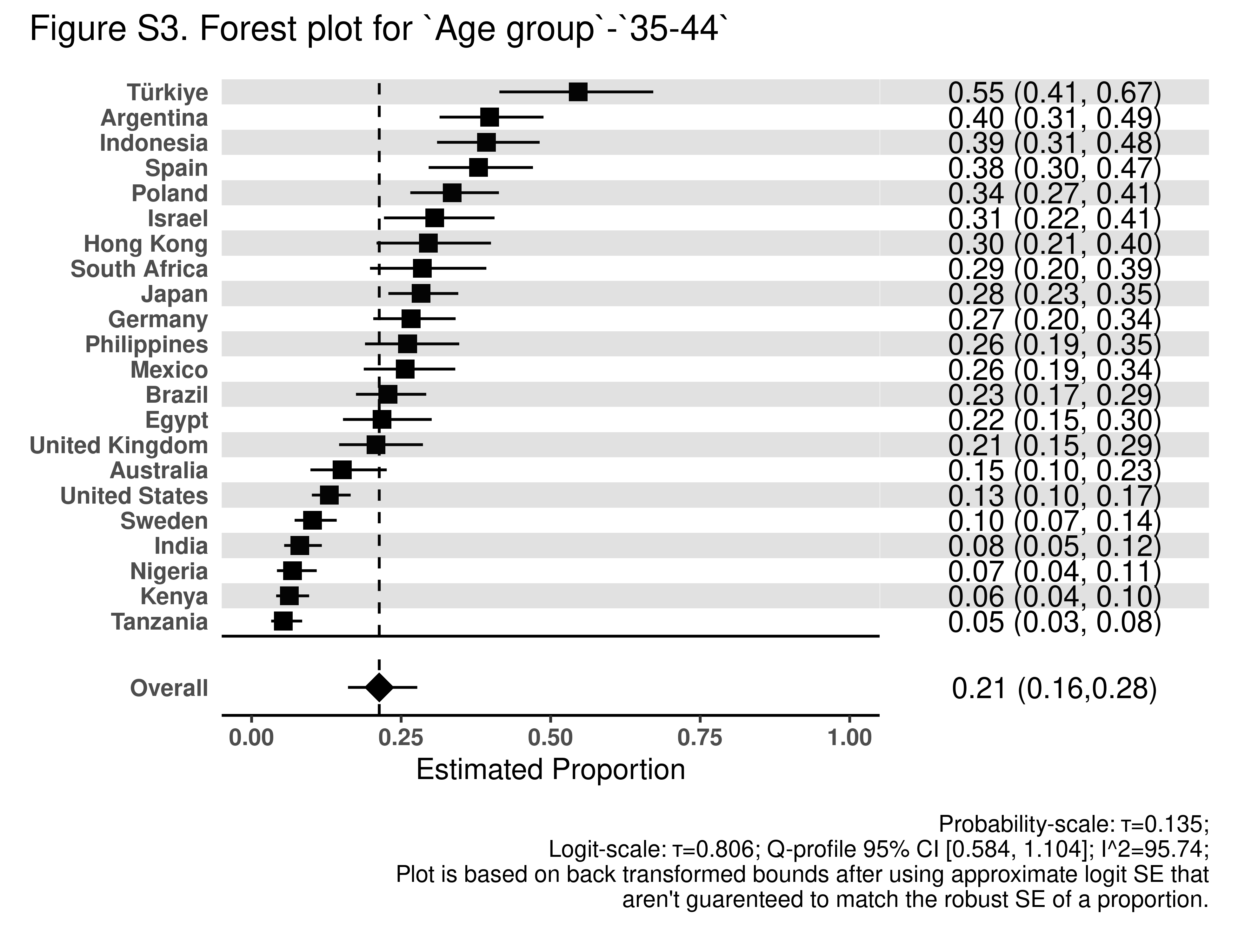

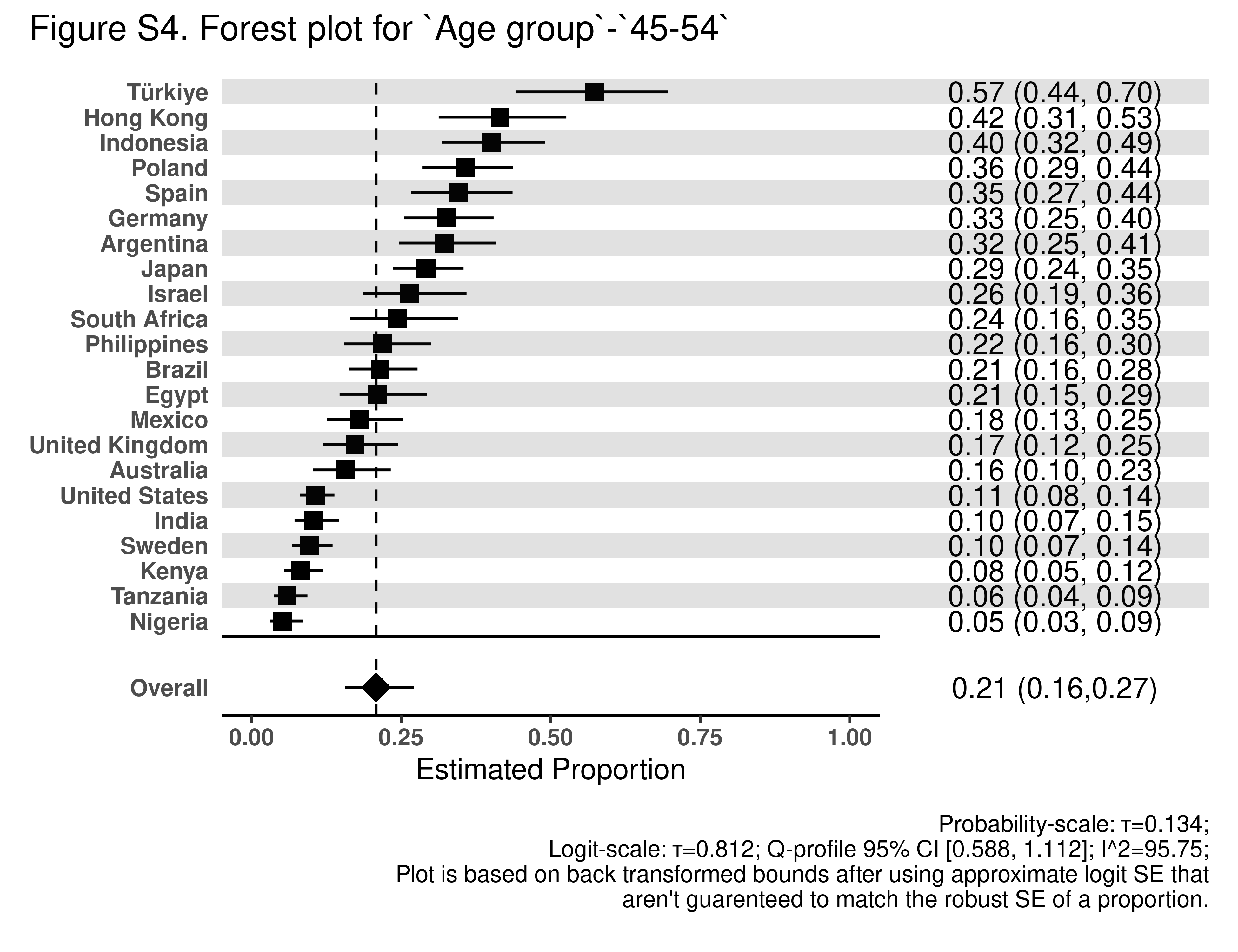

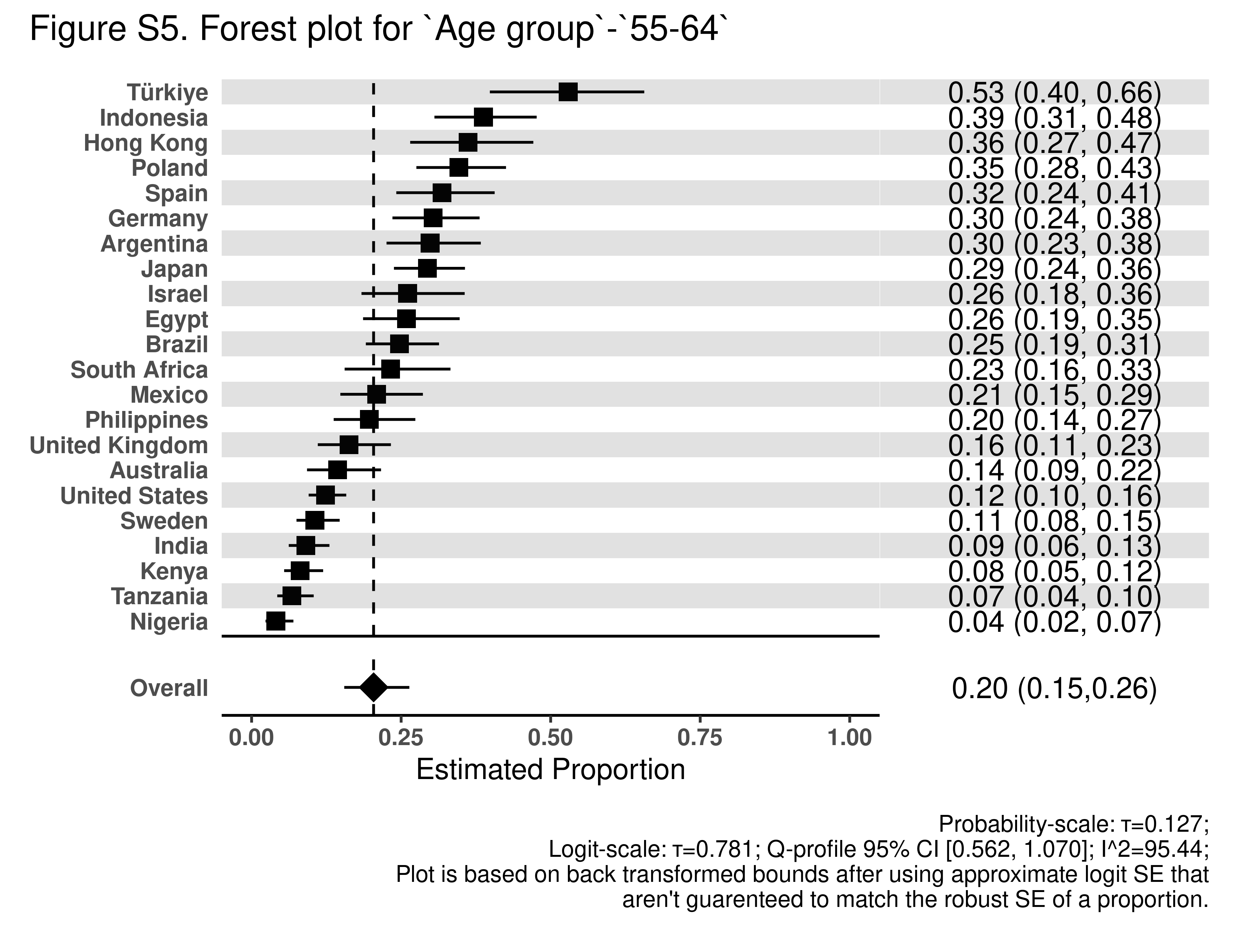

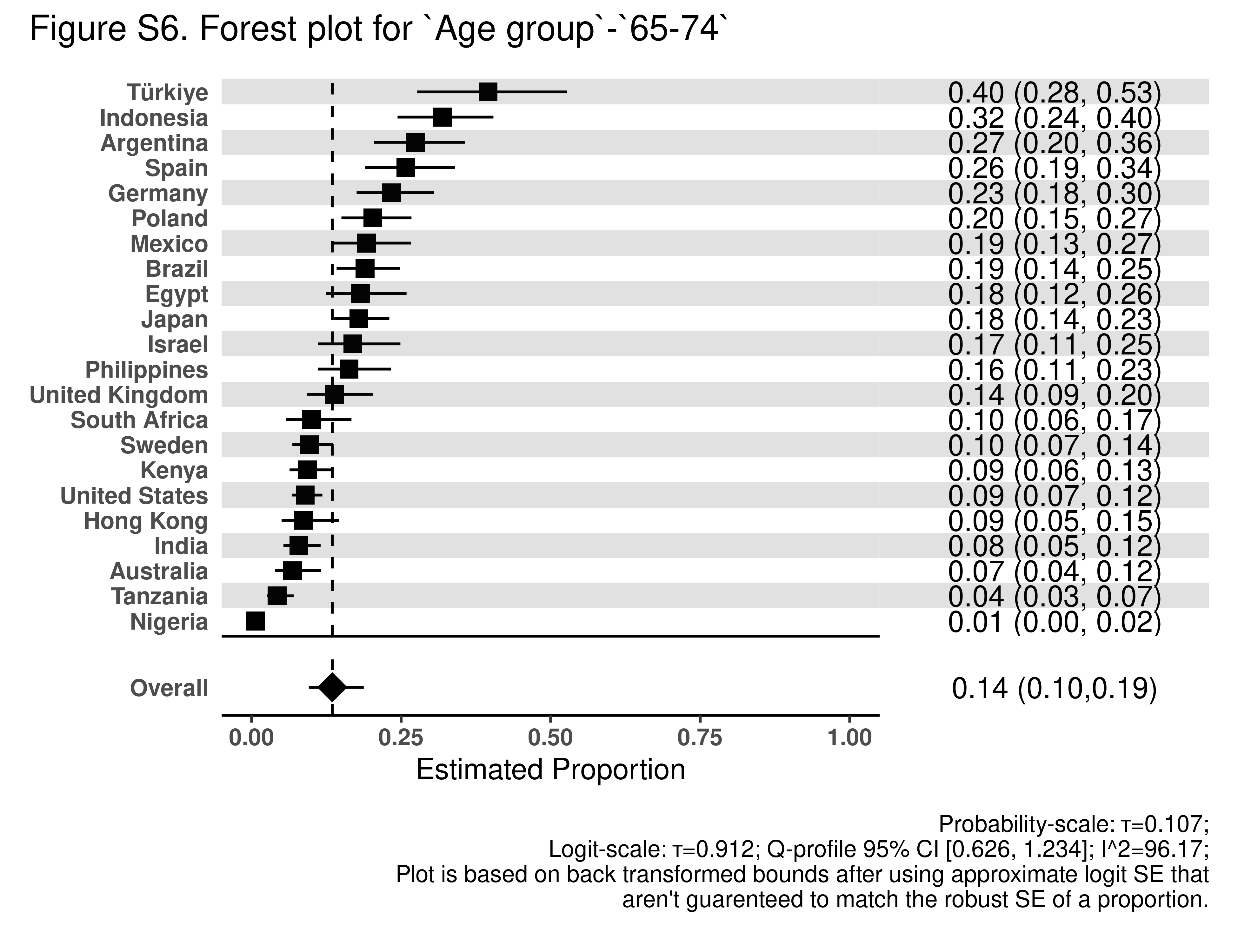

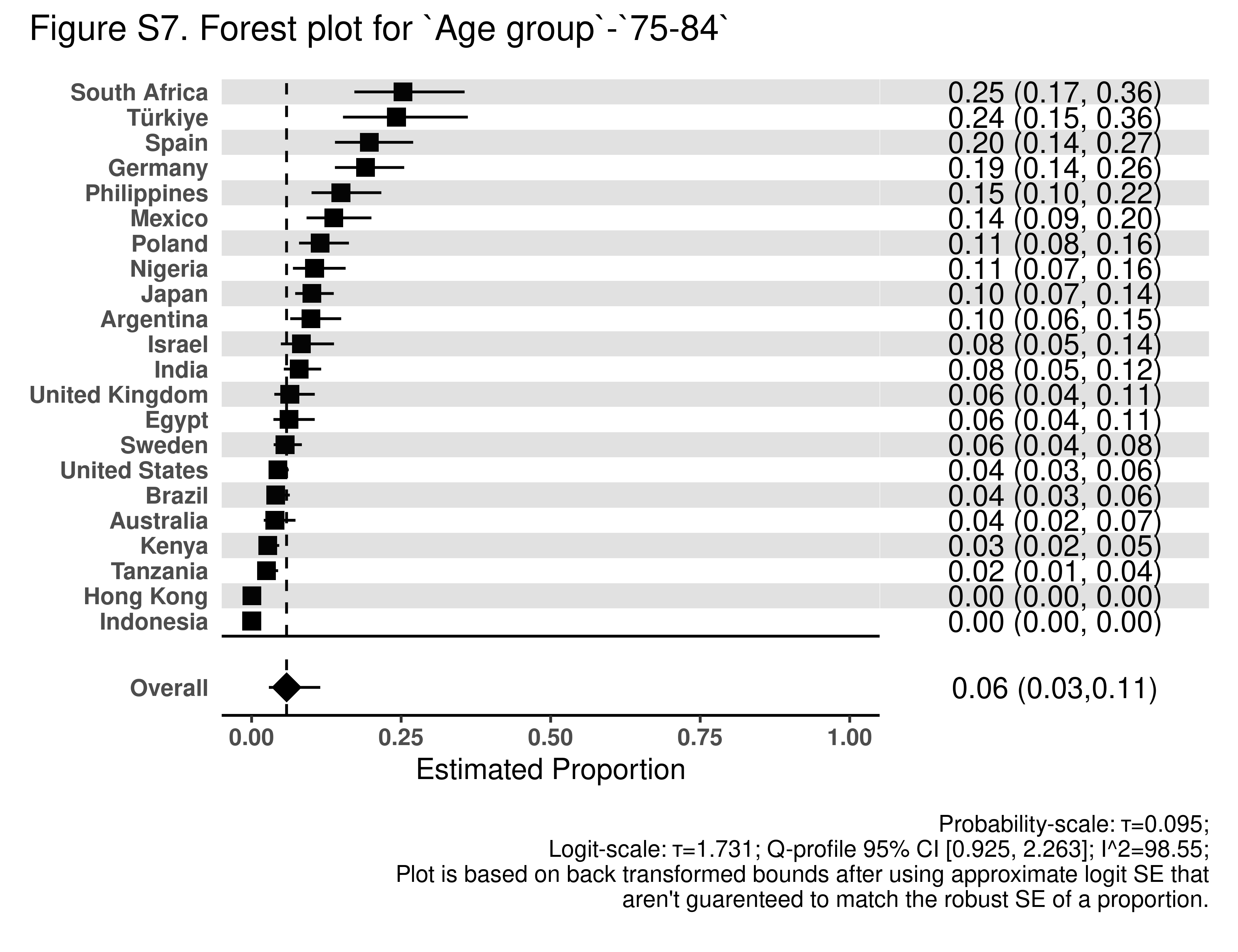

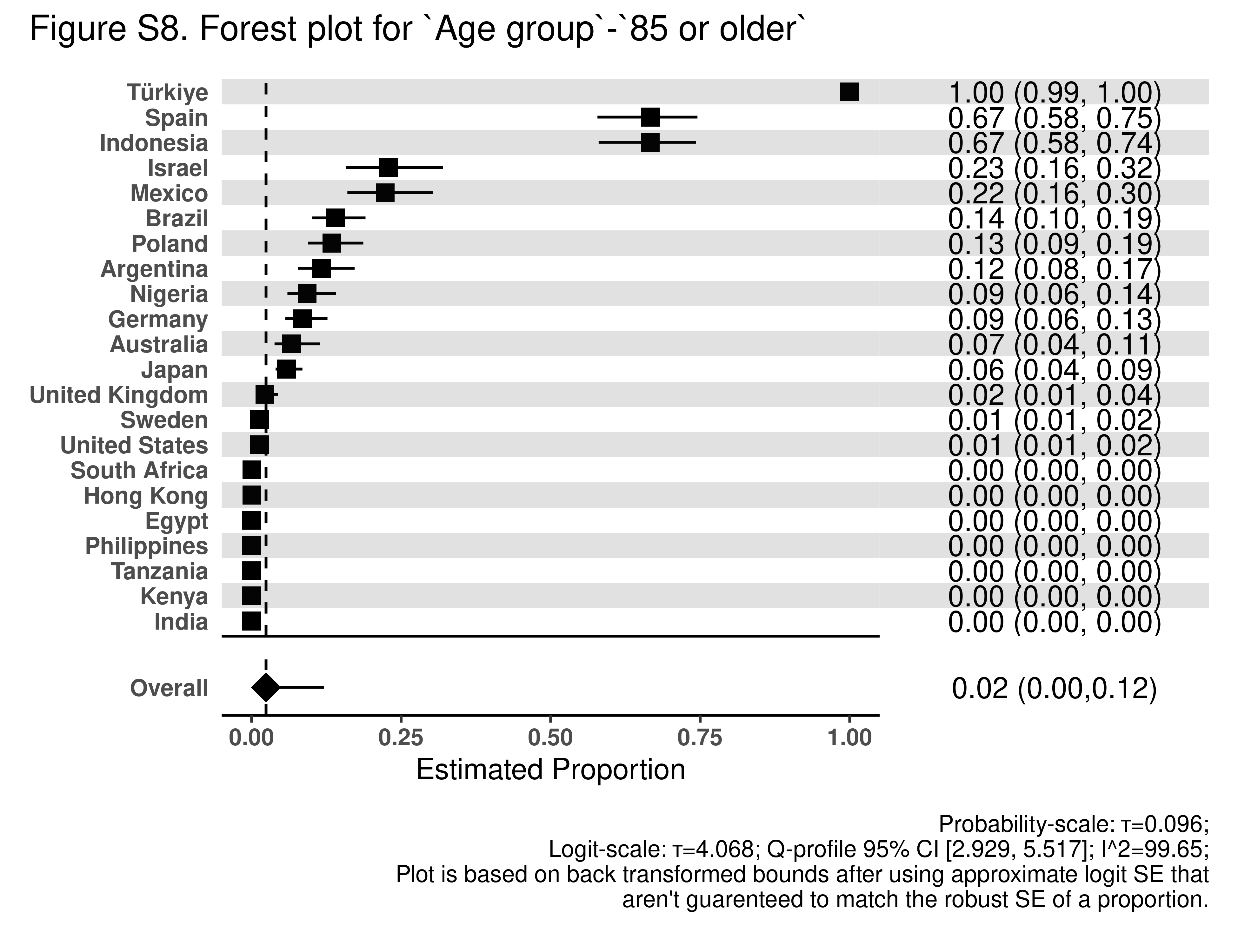

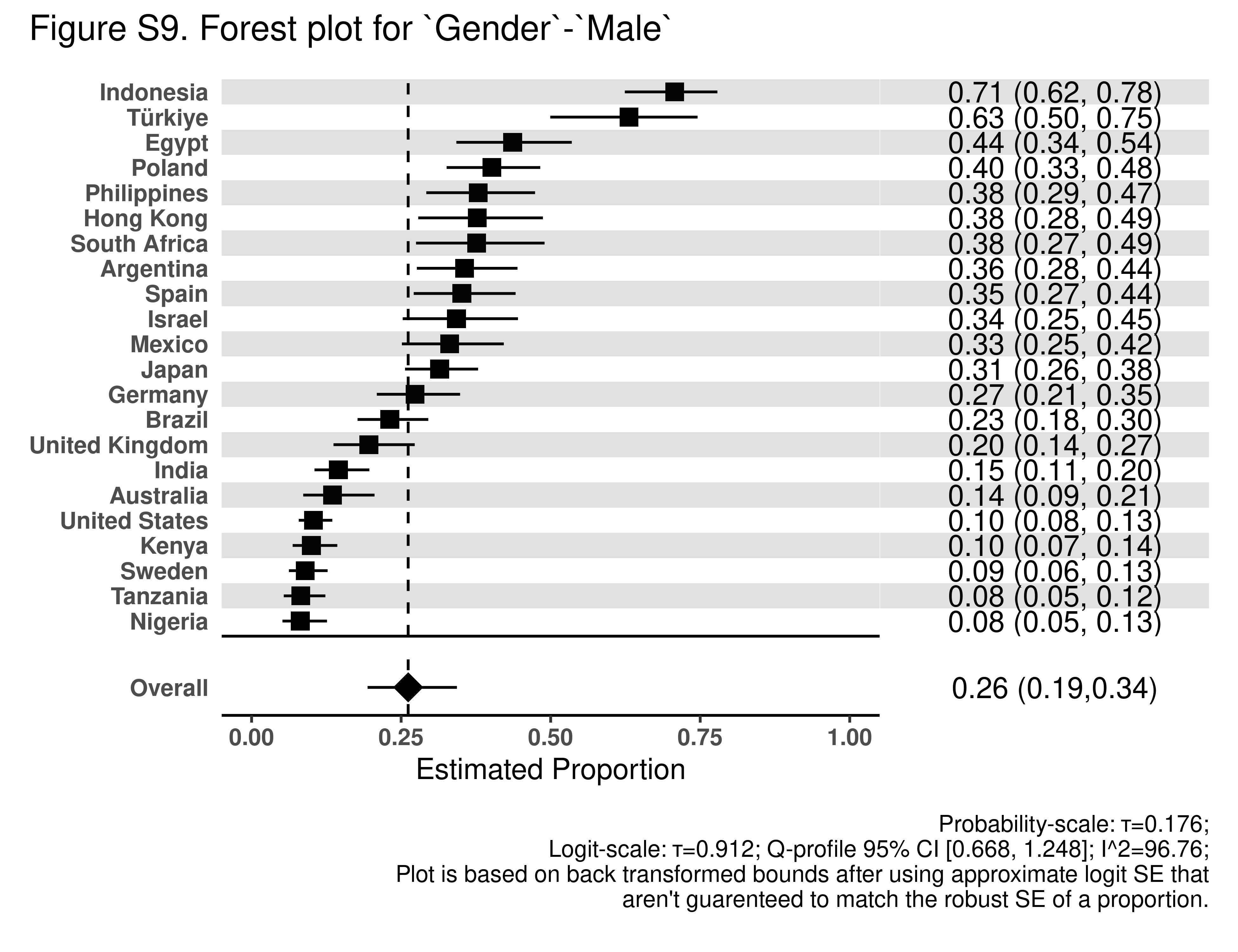

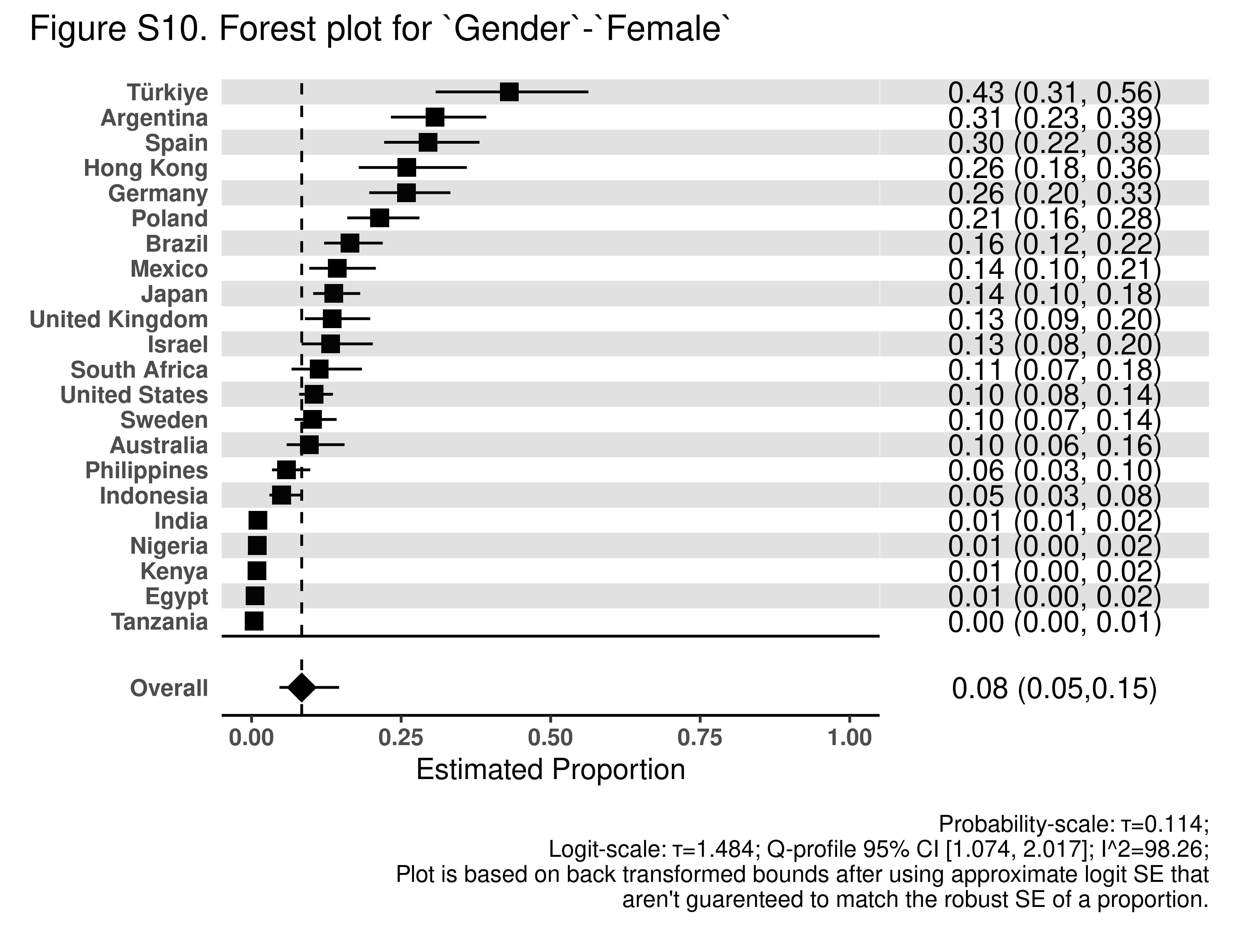

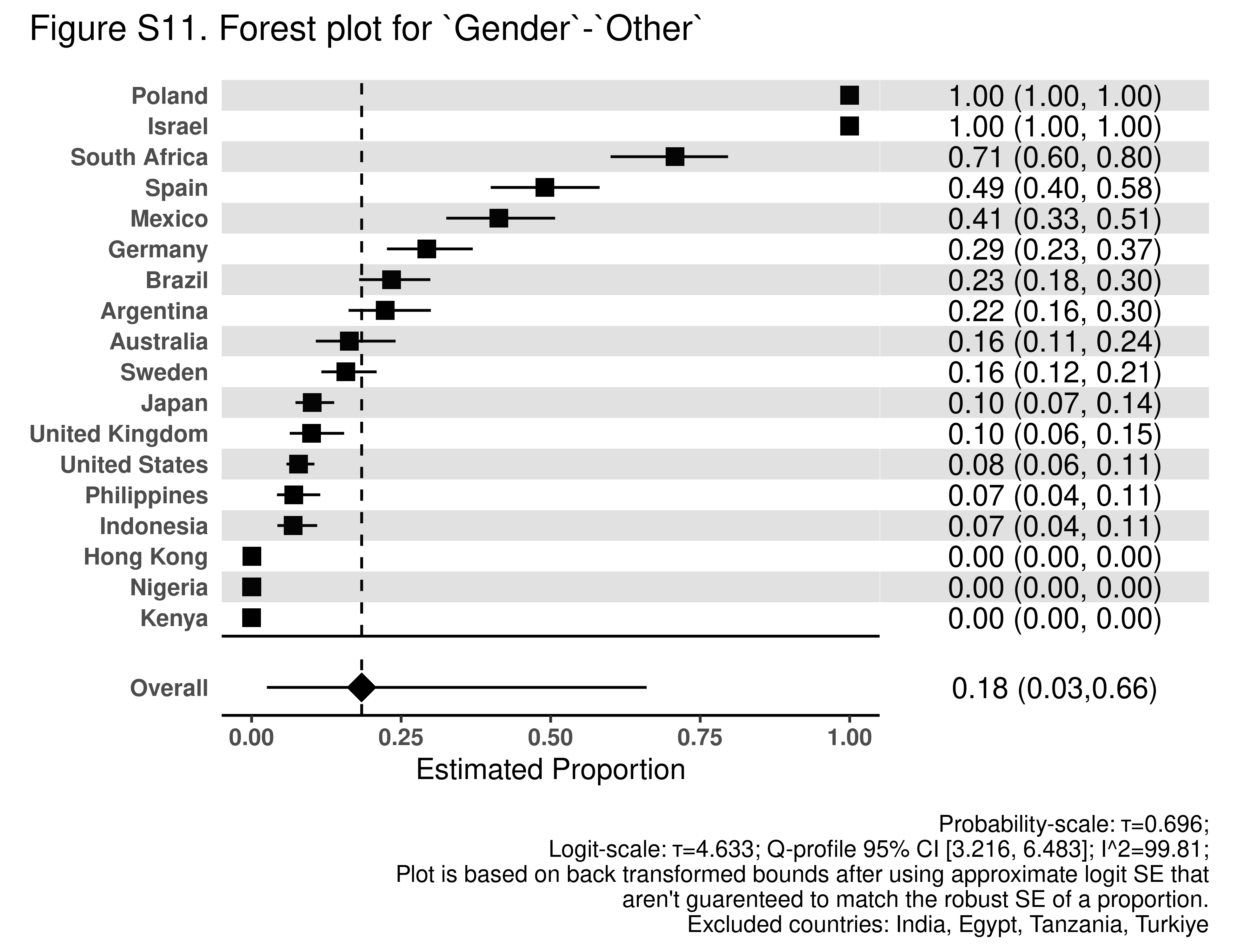

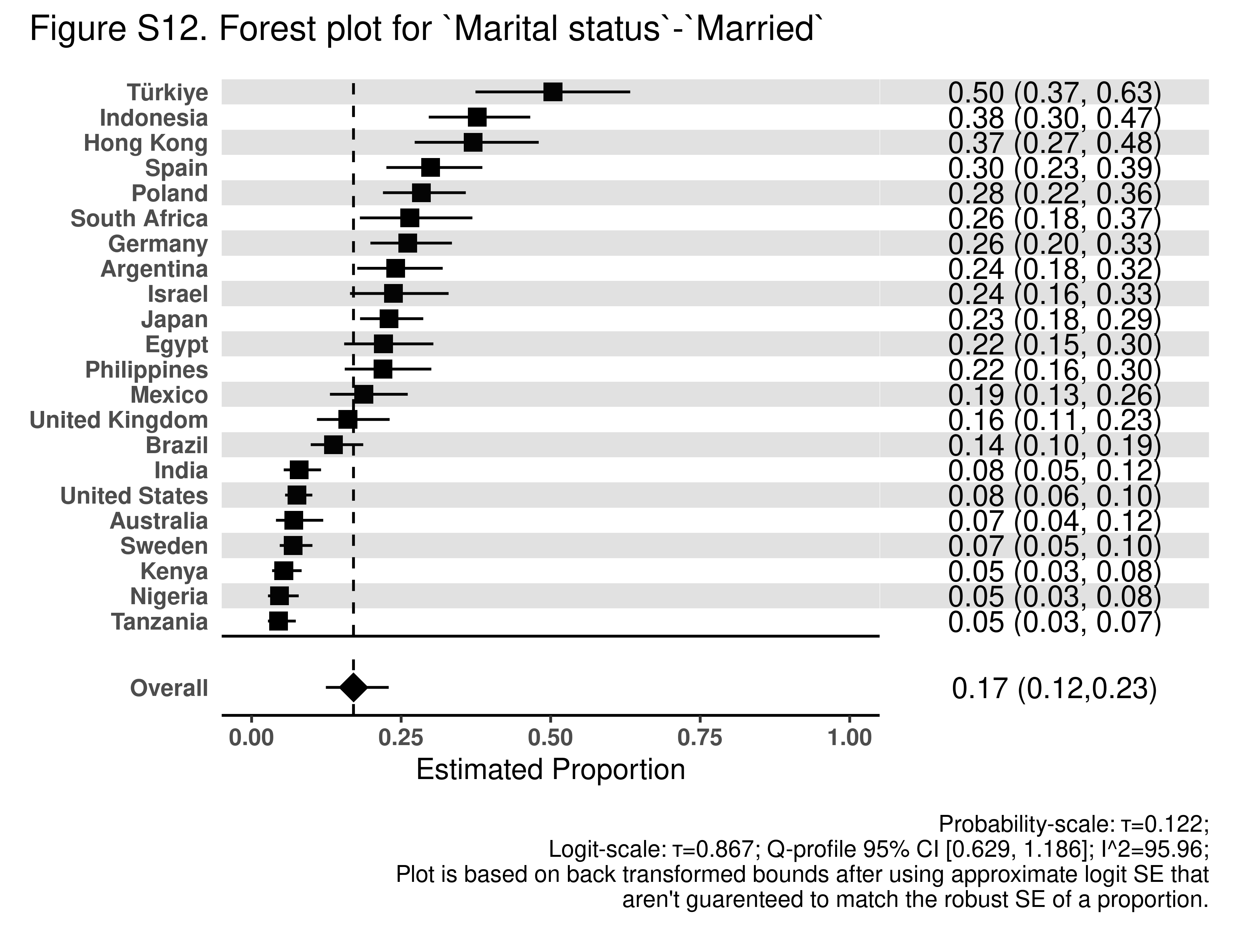

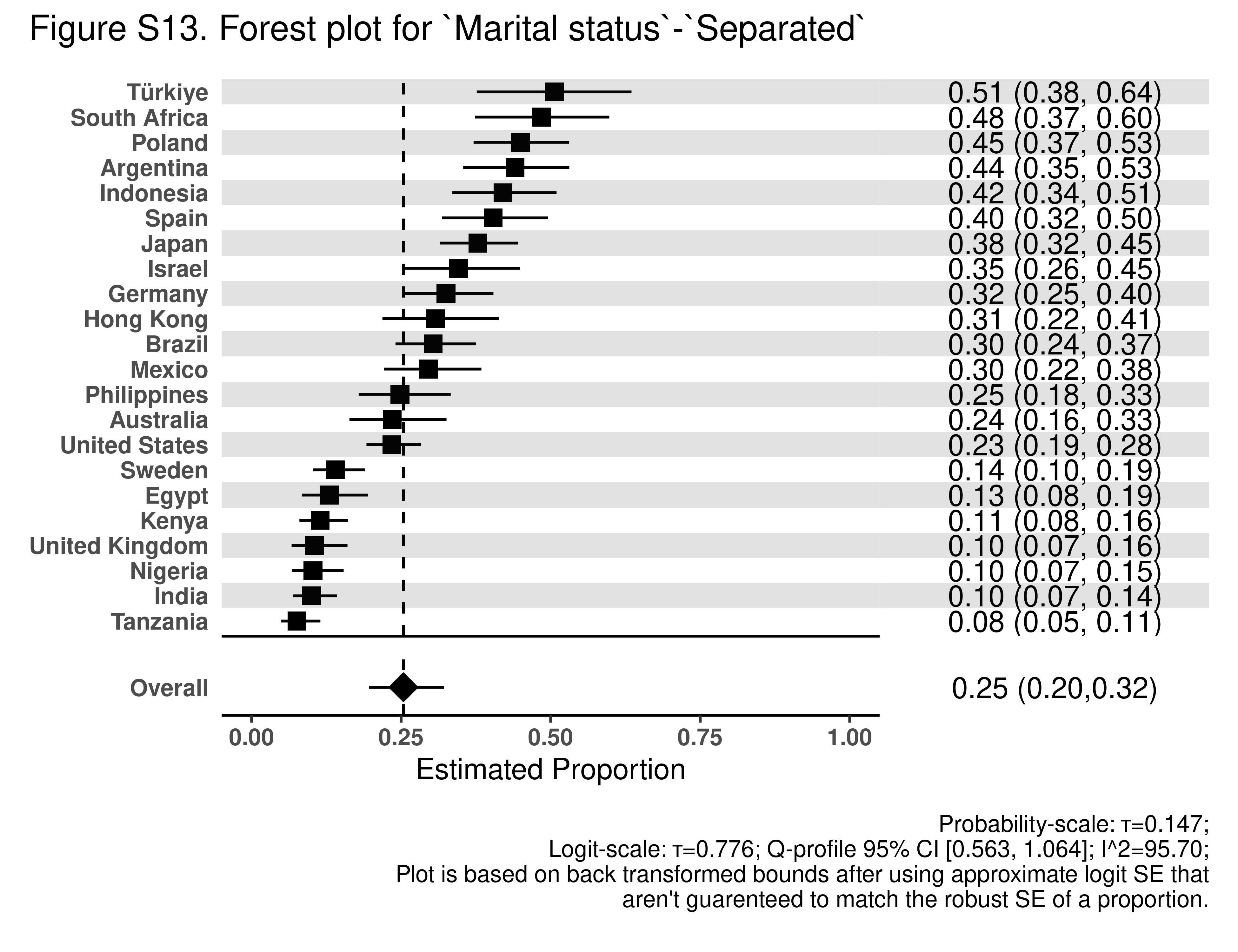

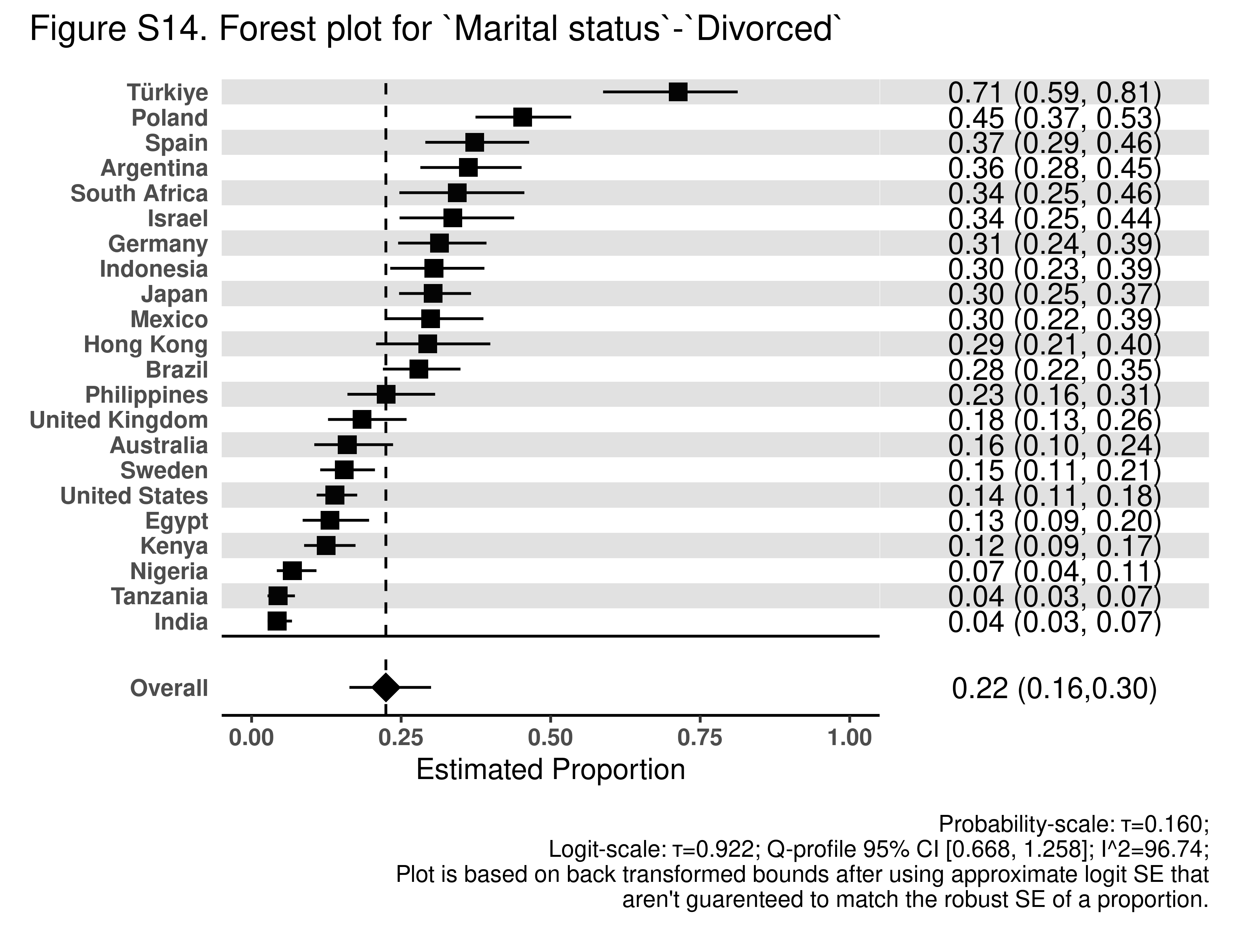

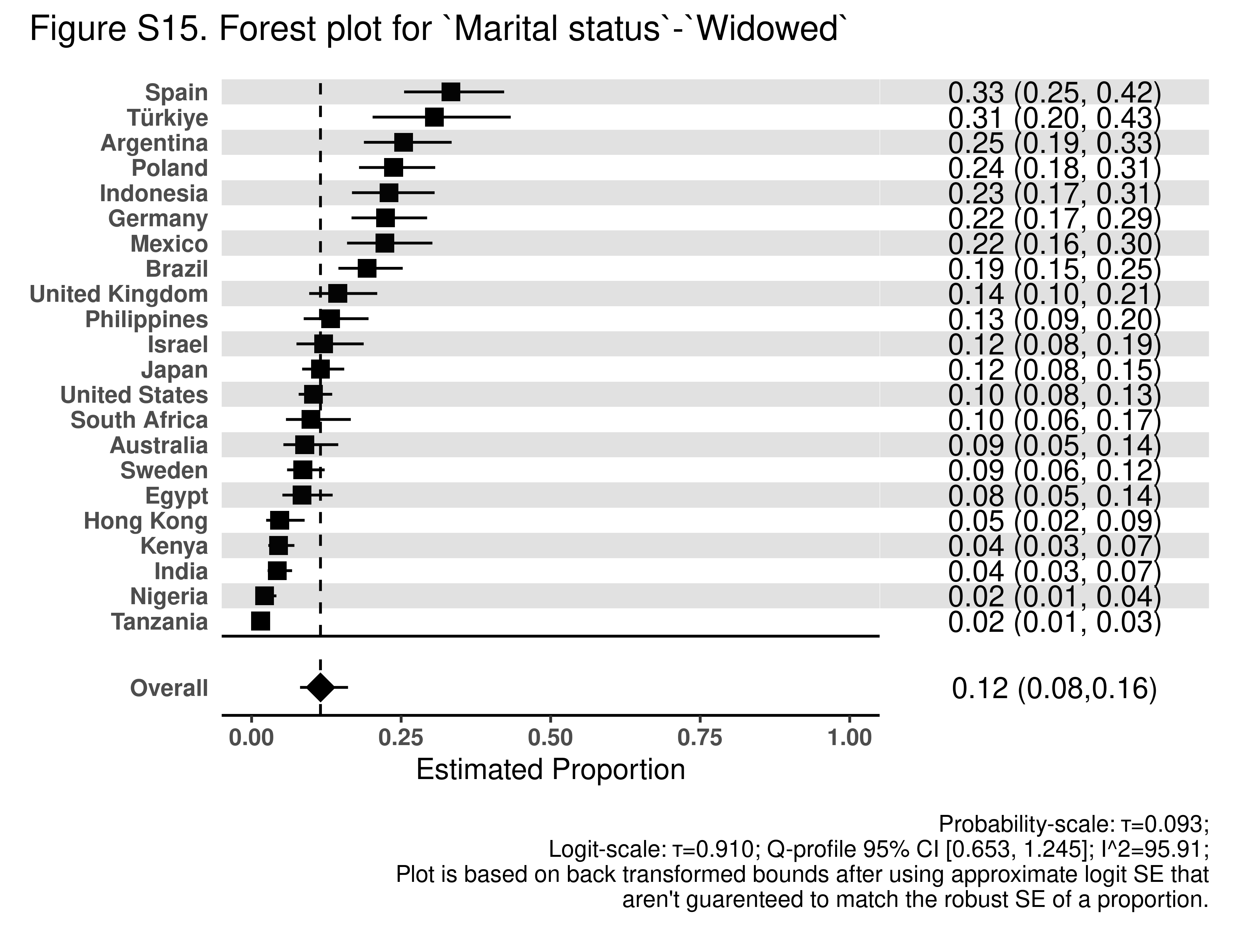

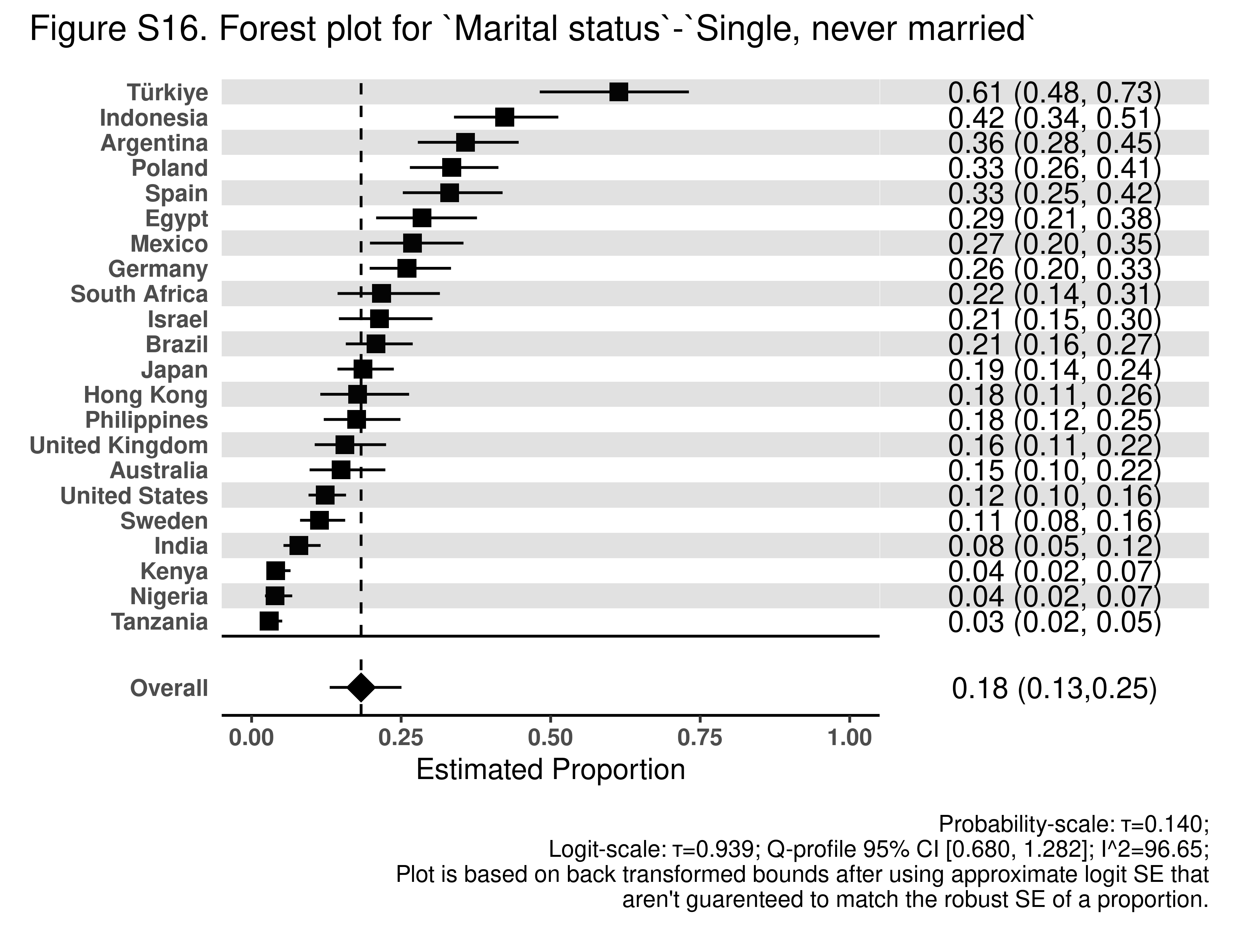

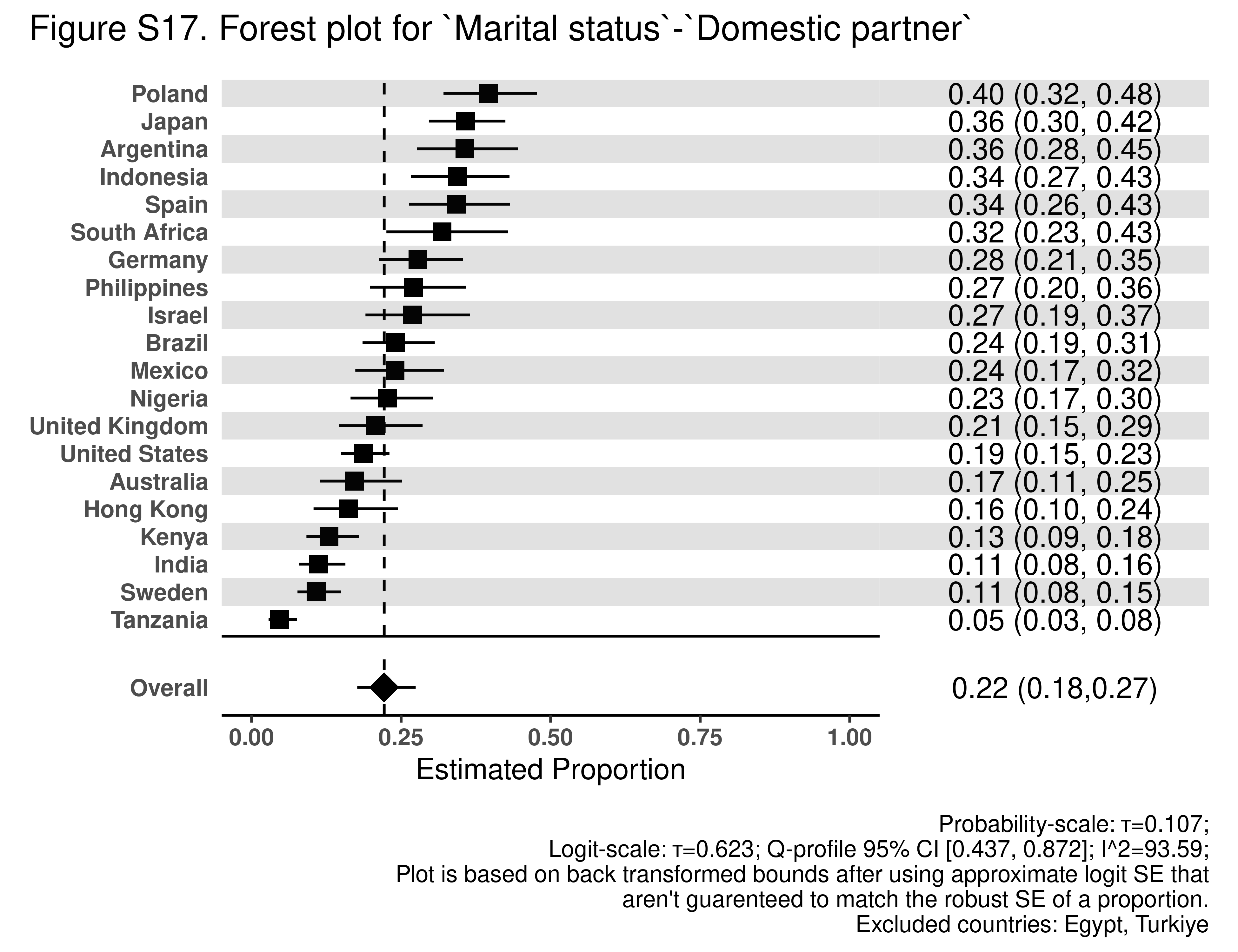

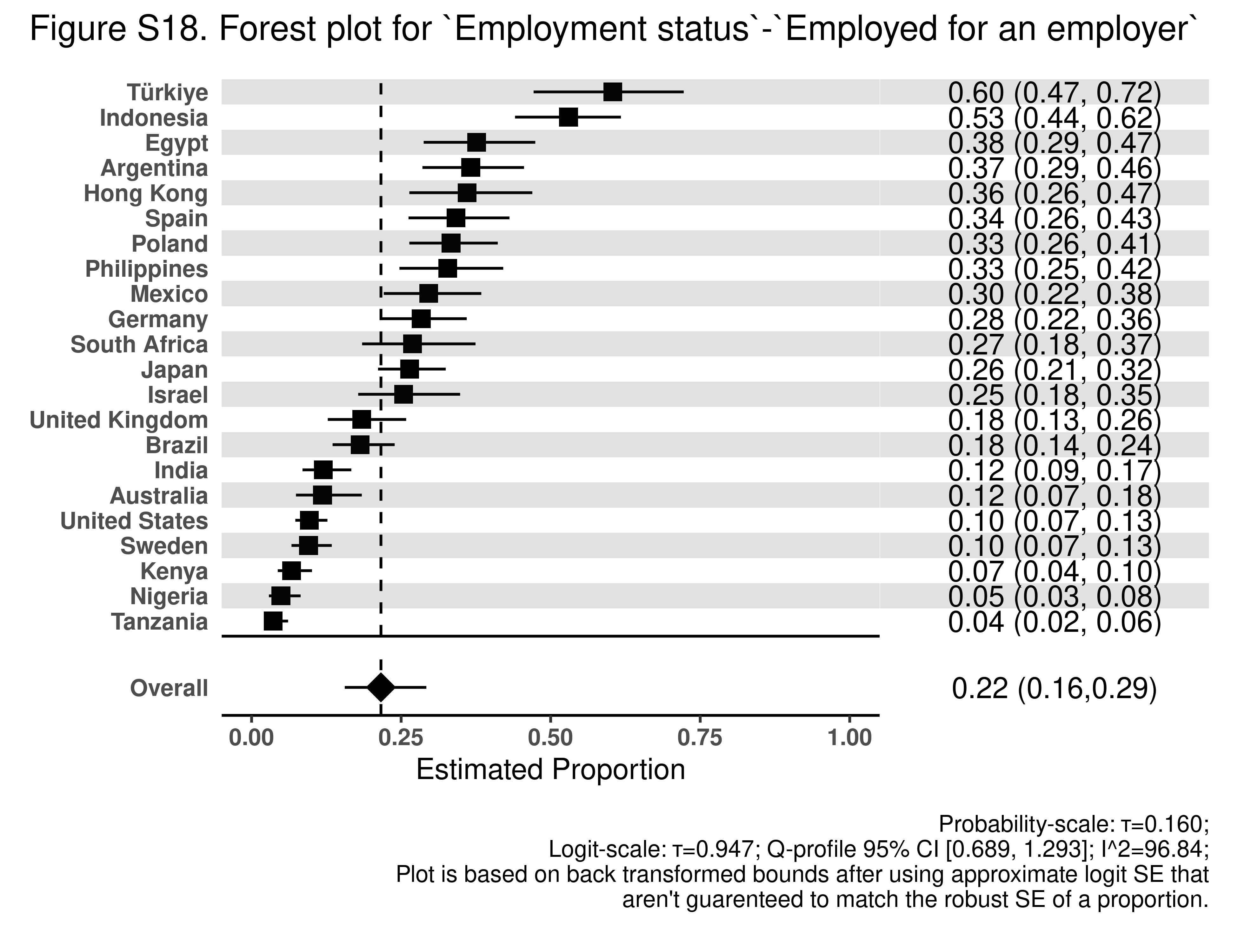

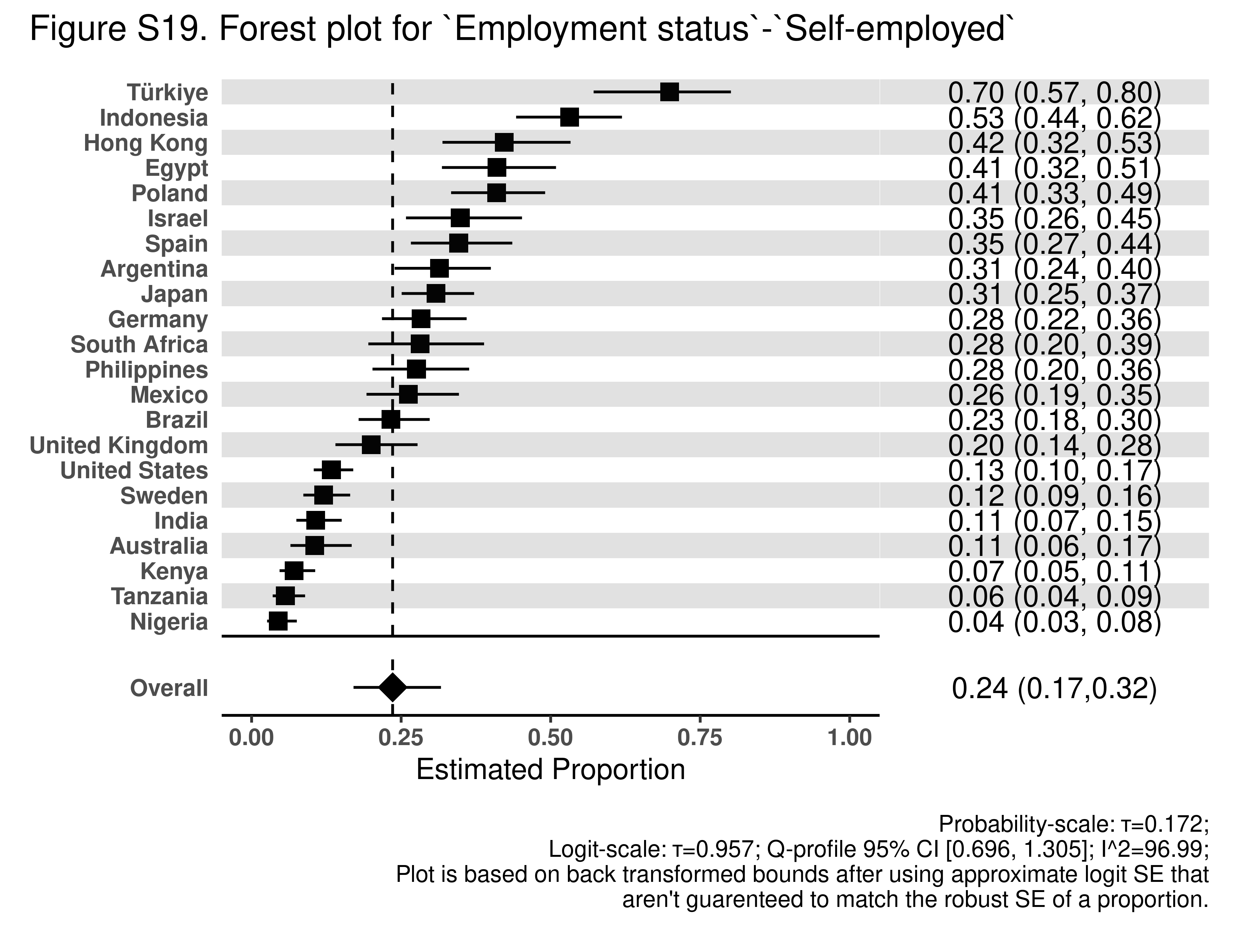

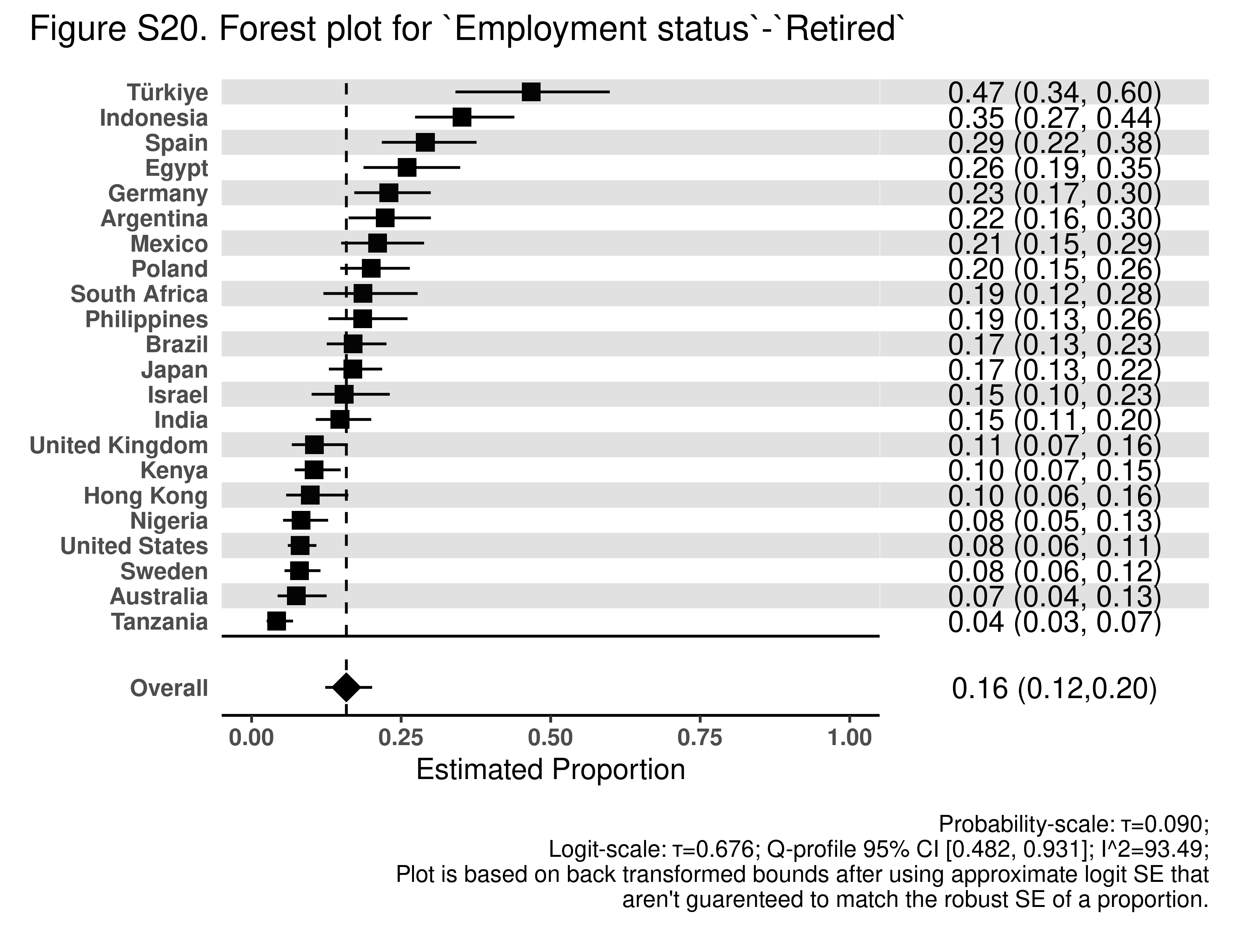

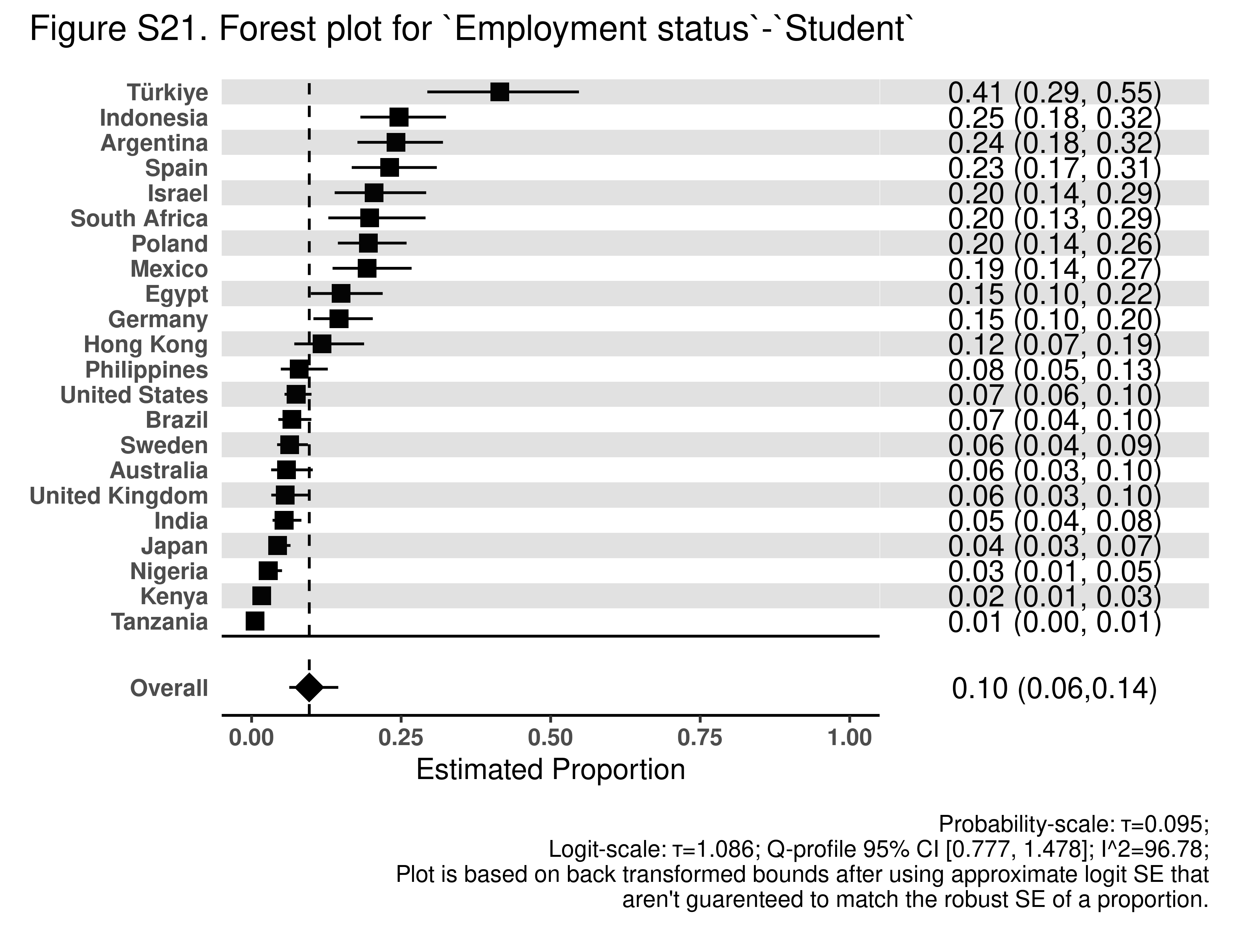

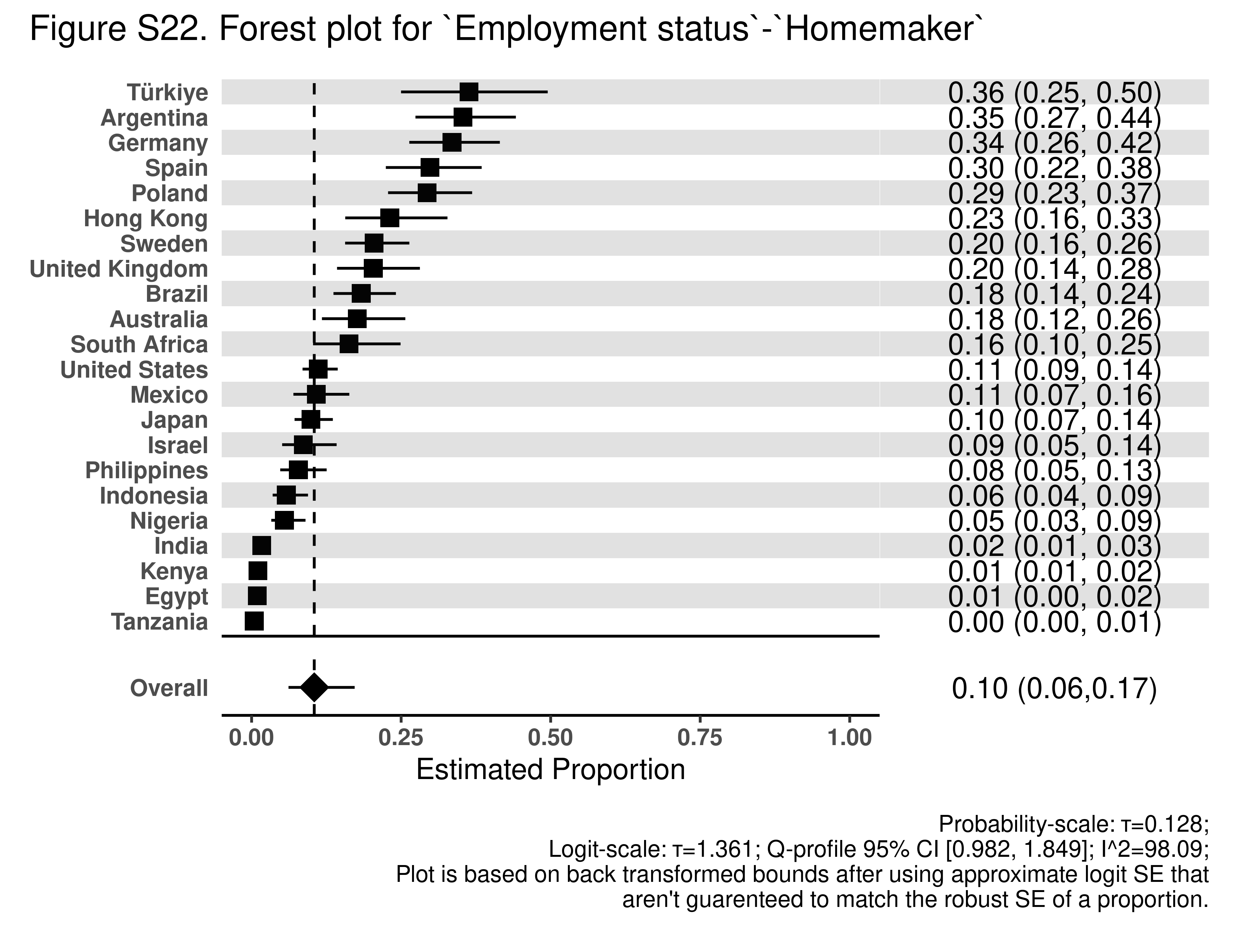

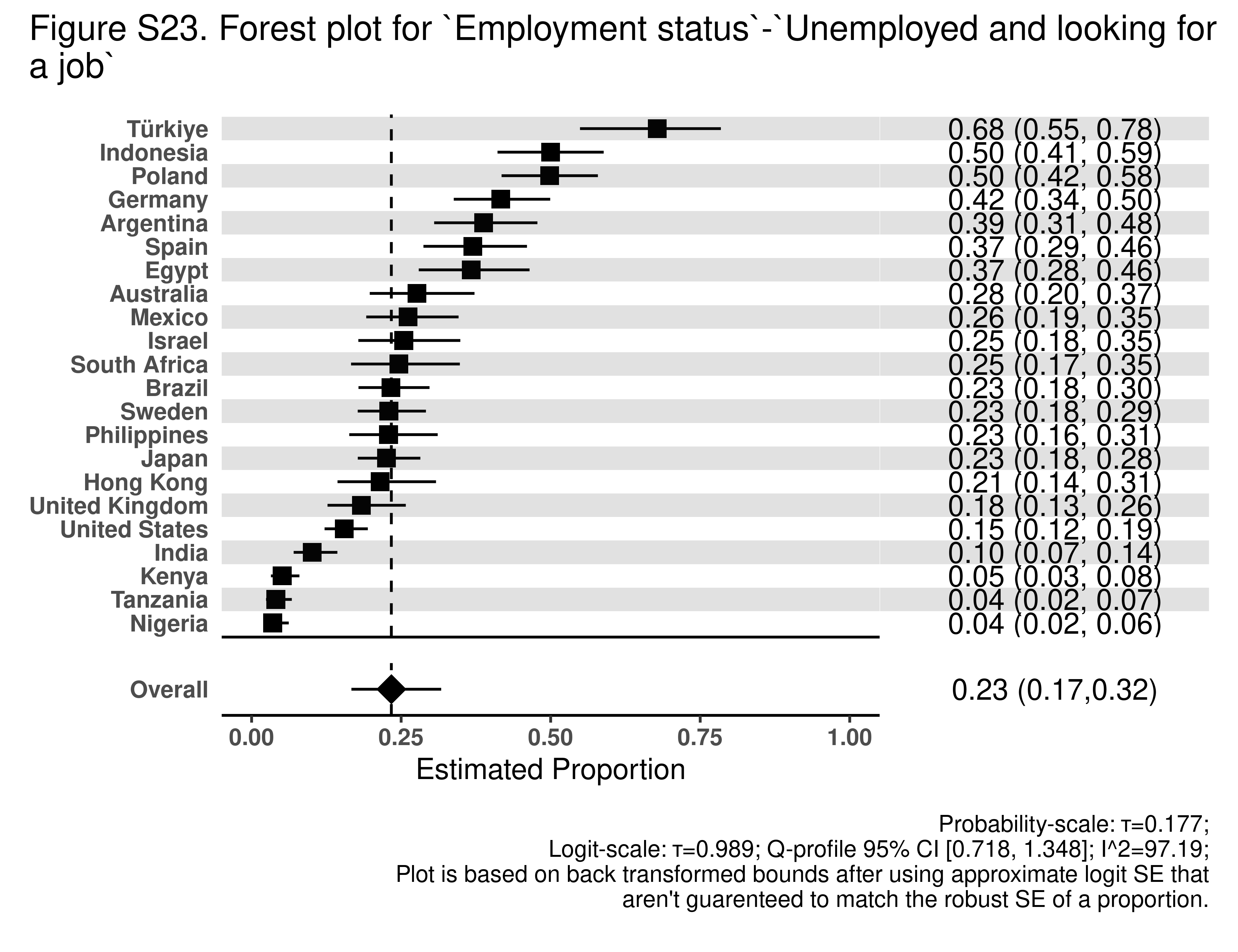

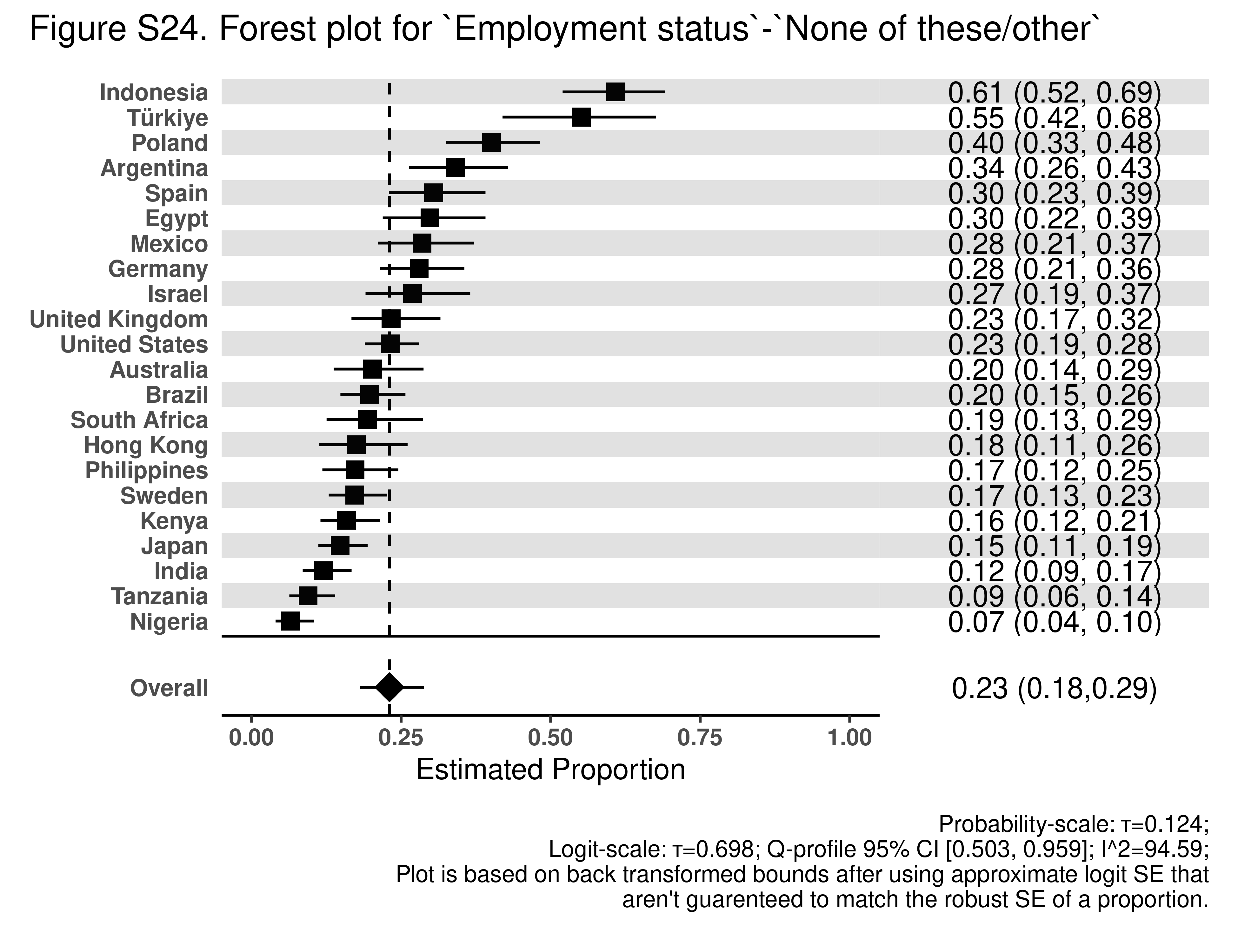

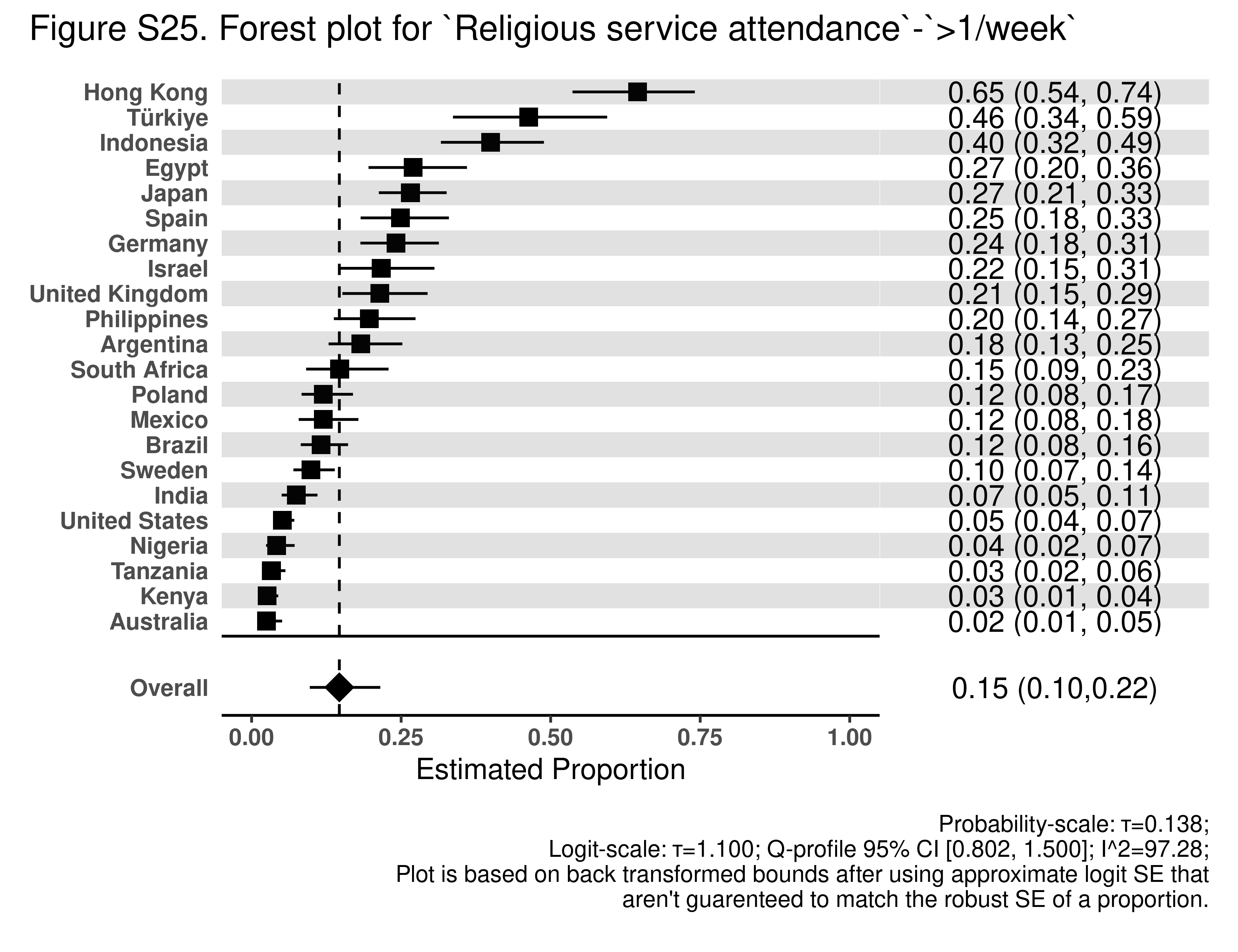

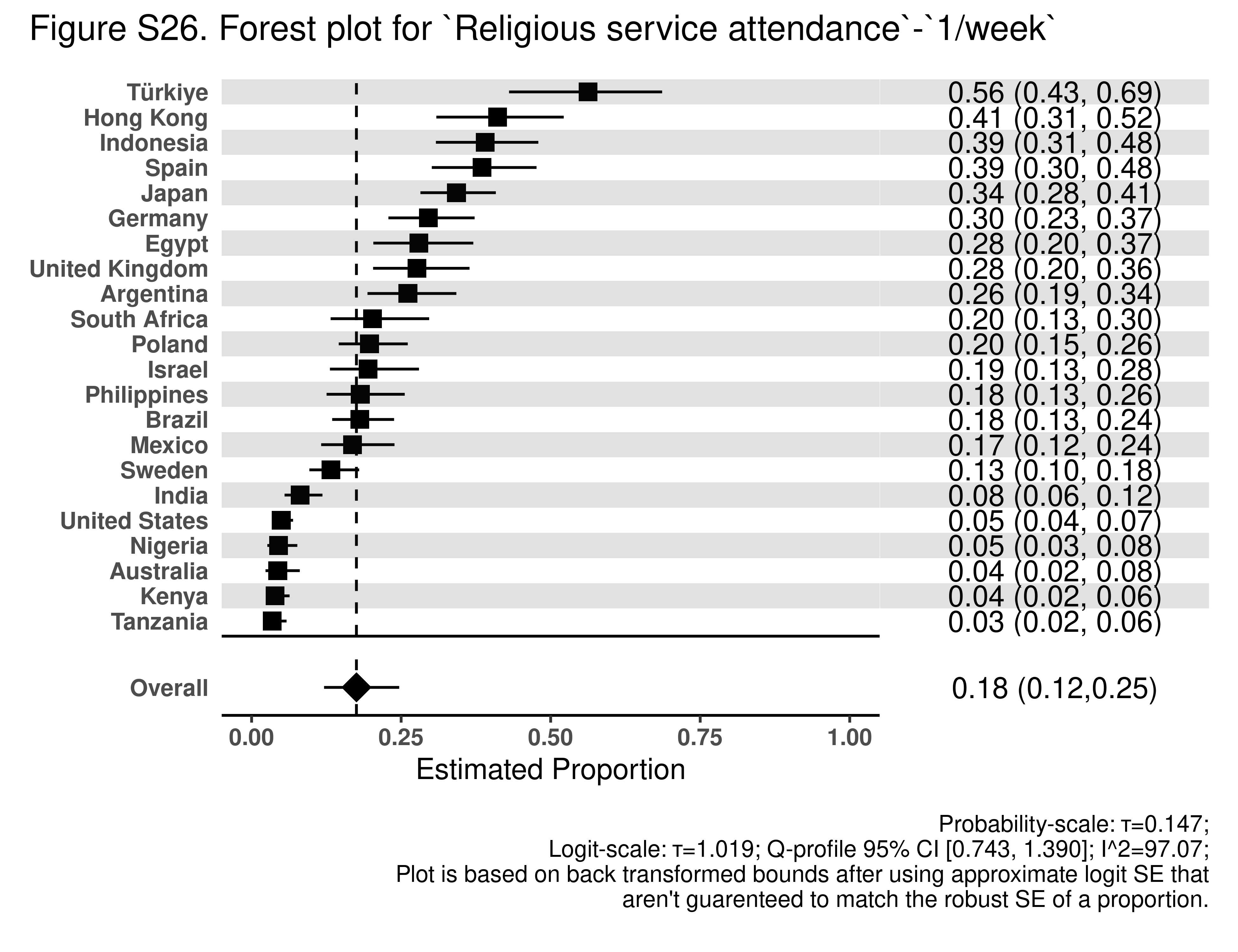

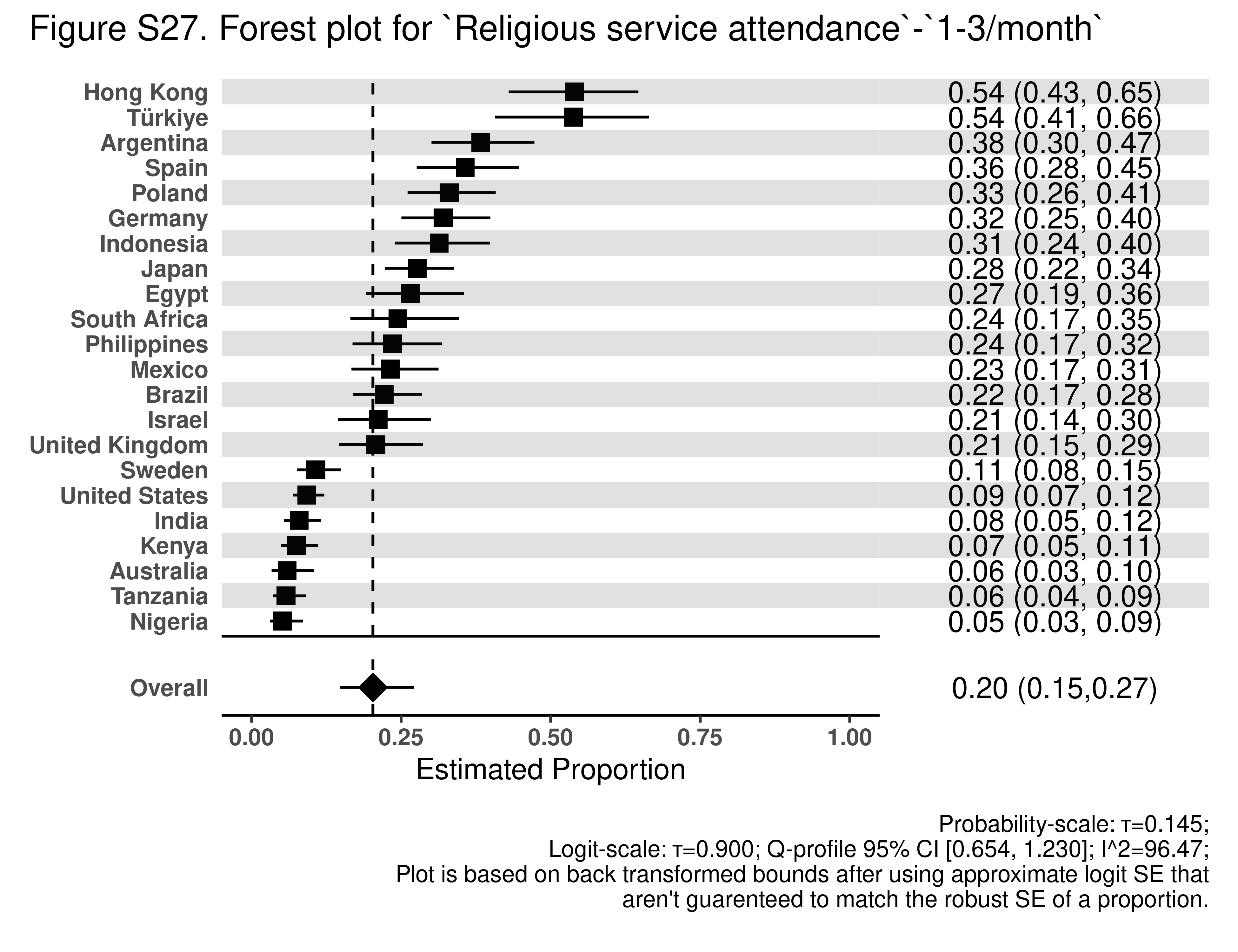

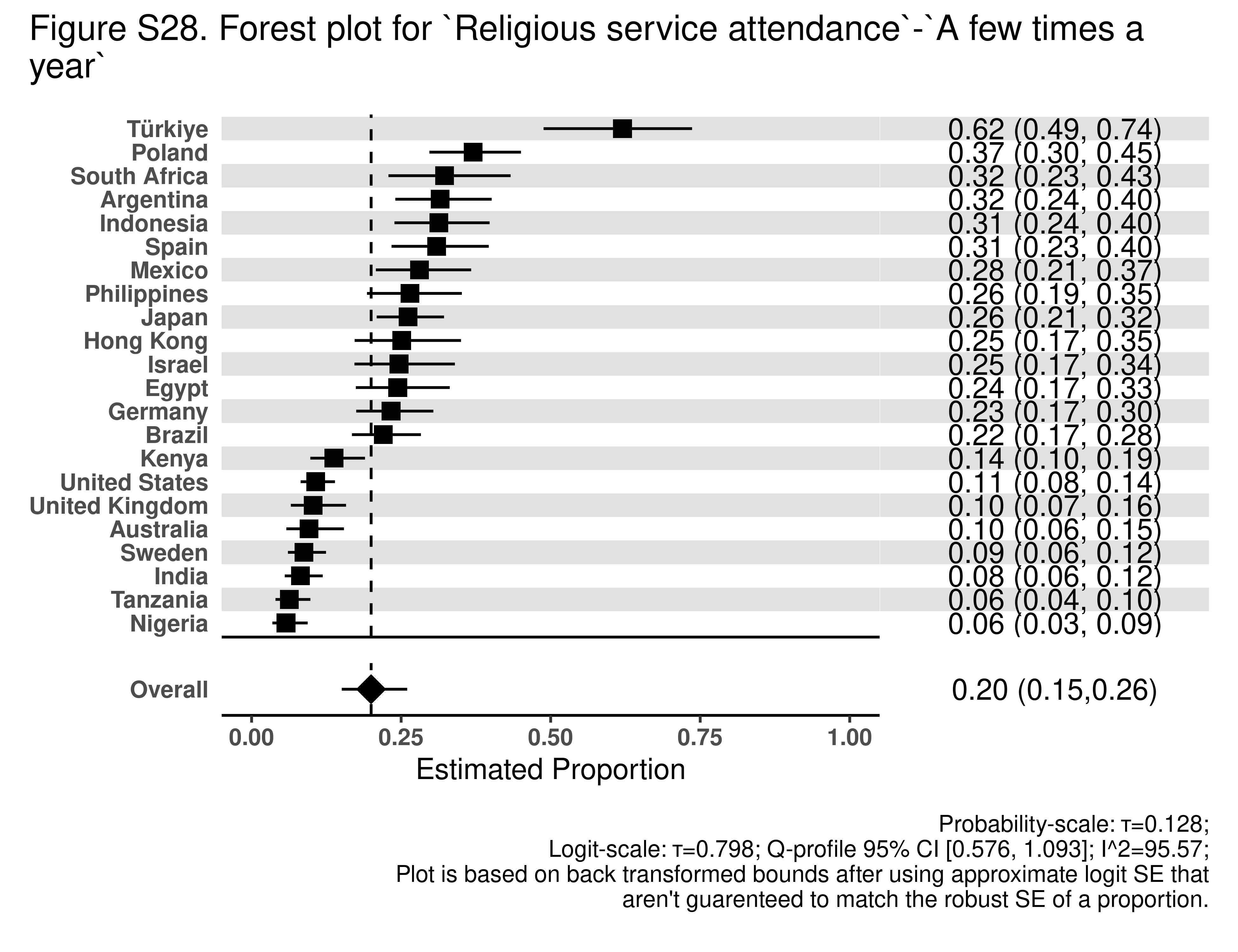

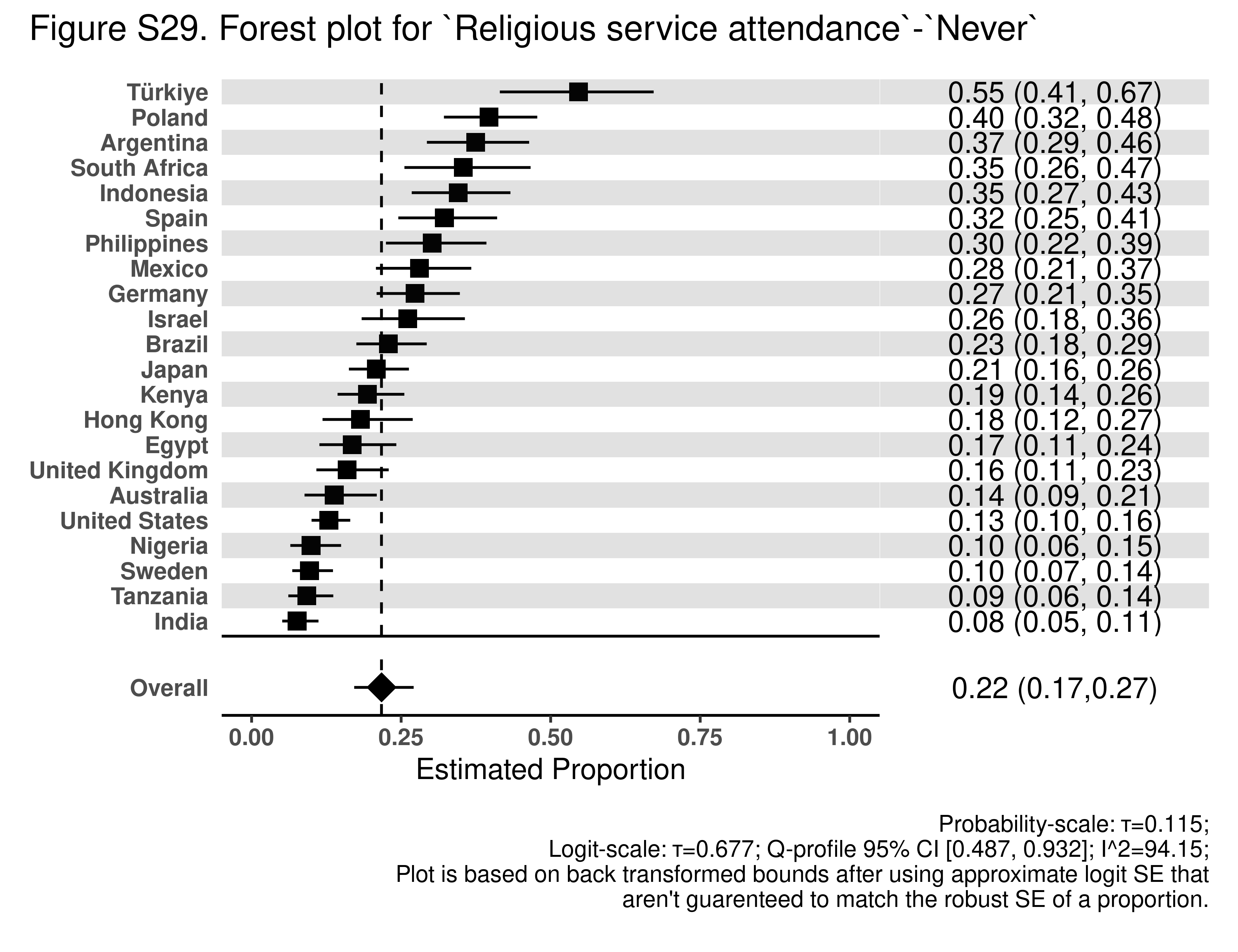

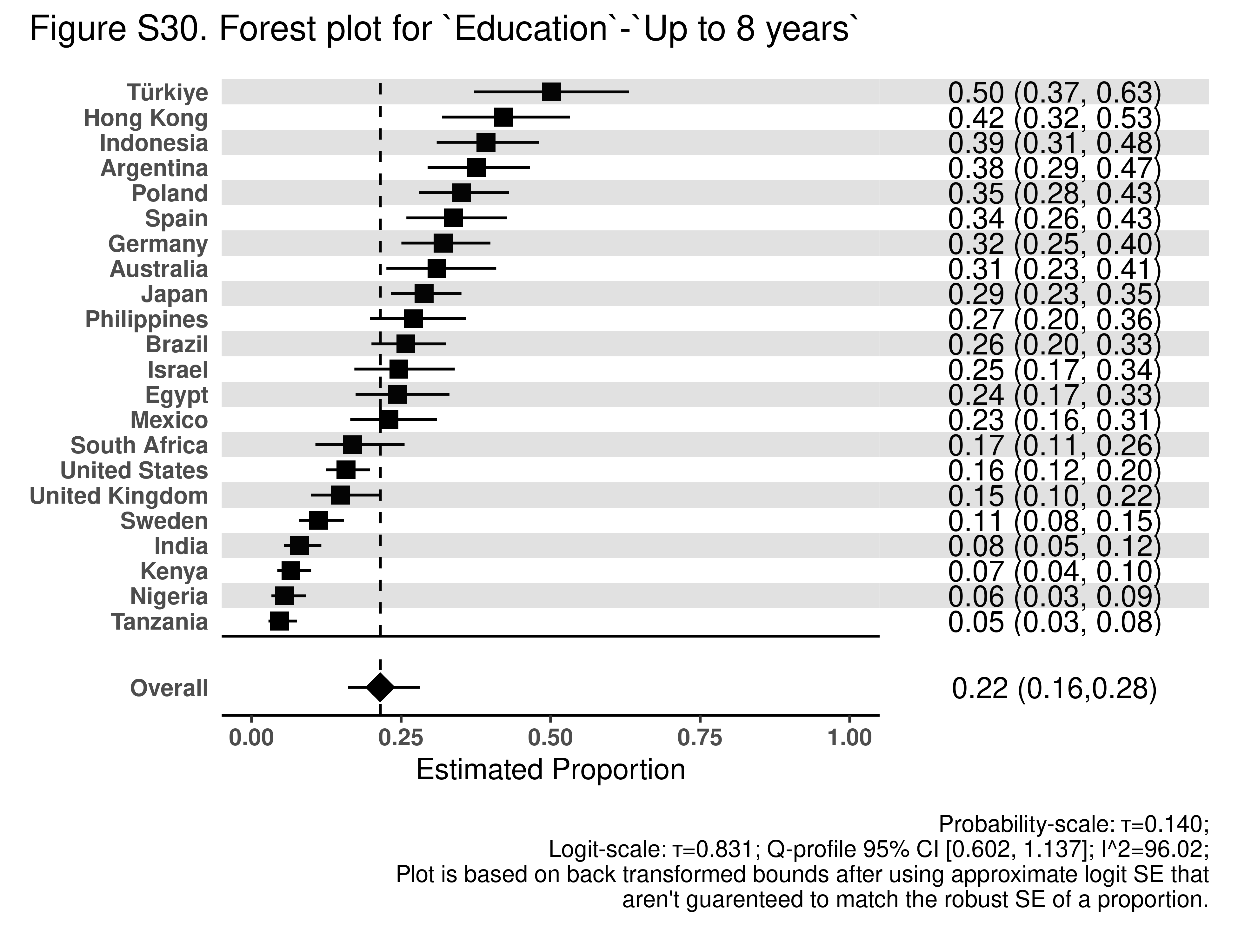

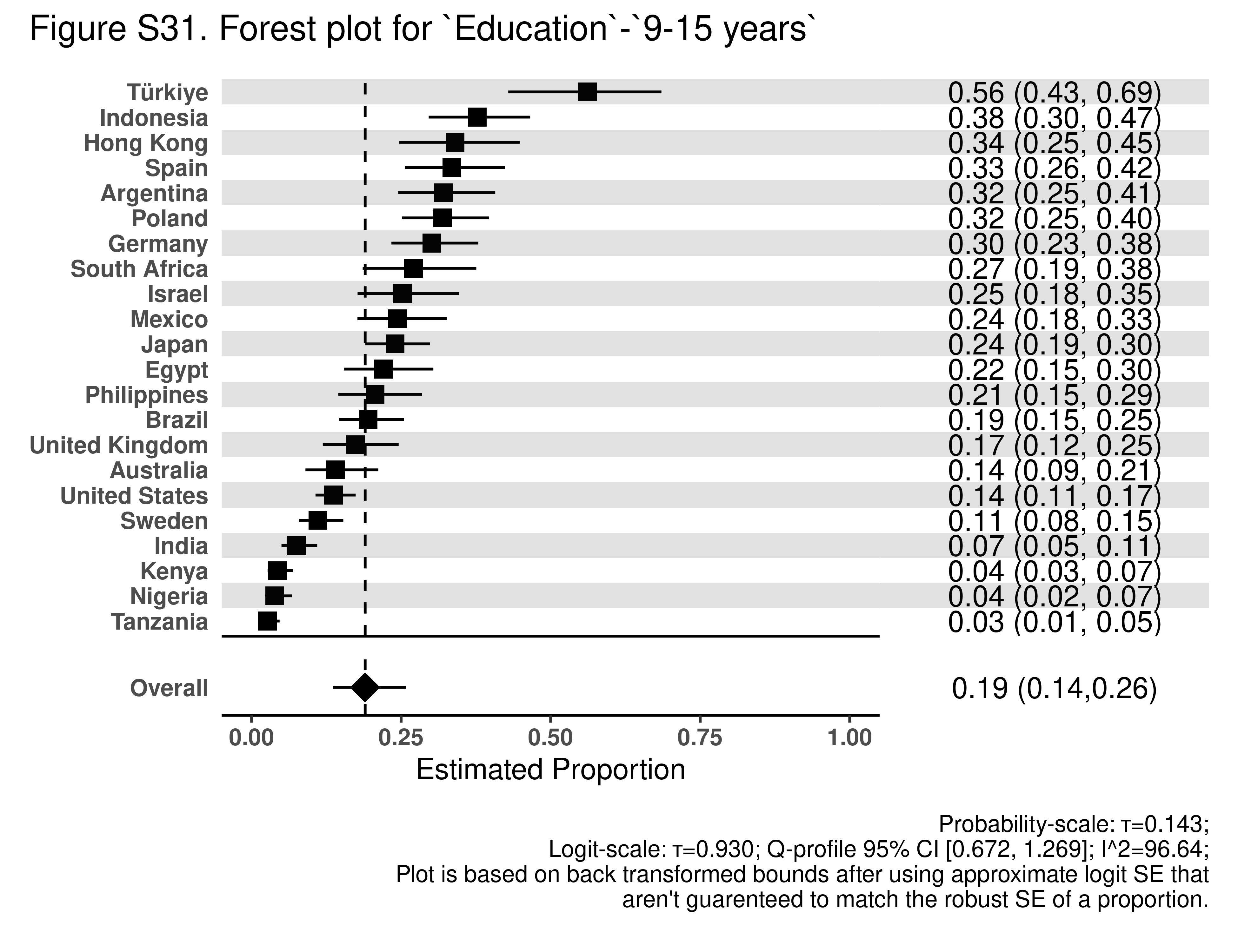

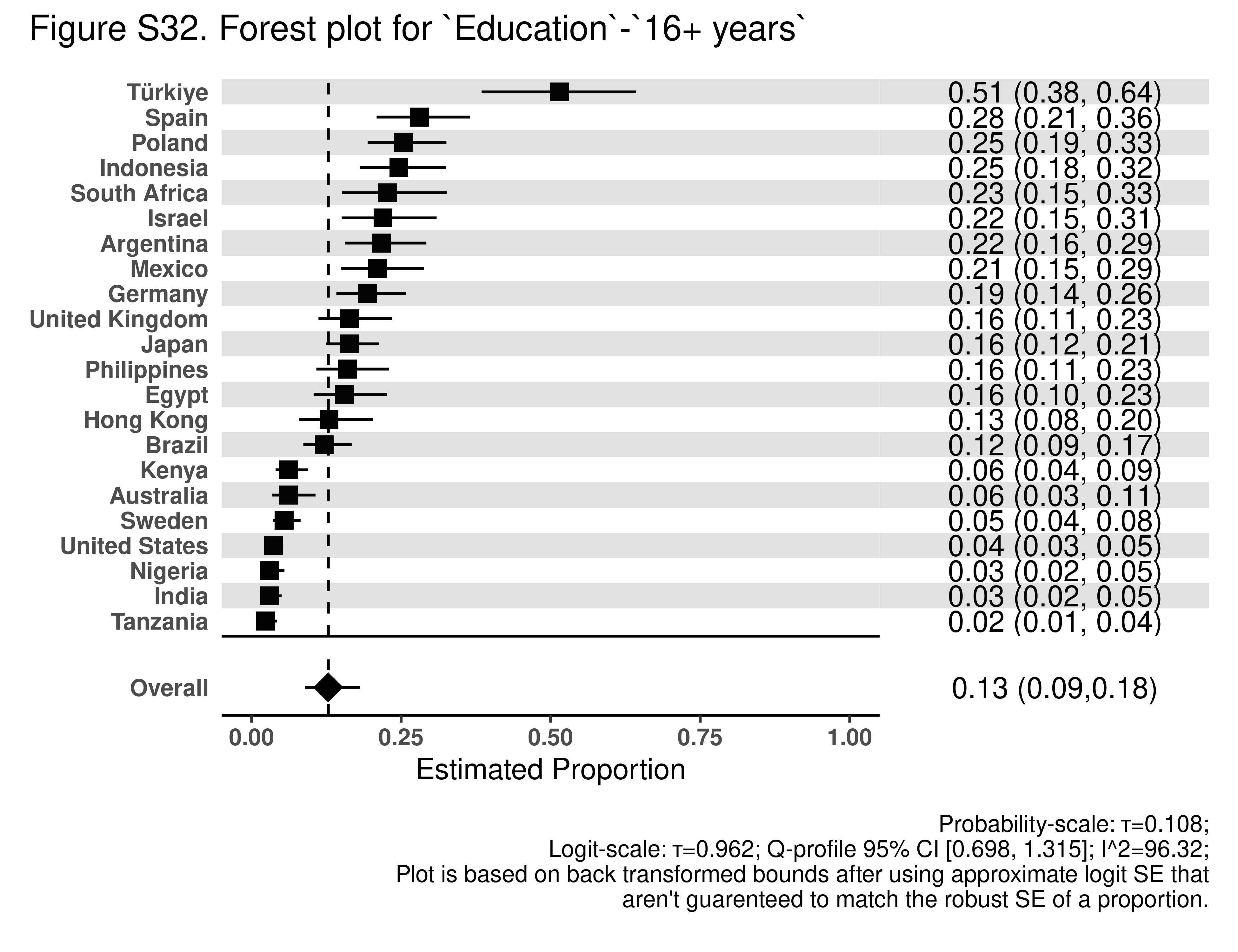


**Figure S70.**

**Figure S69.**

**Figure S72.**

**Figure S71.**

**Figure S74.**

**Figure S73.**

**Figure S76.**

**Figure S75.**

**Figure S78.**

**Figure S77.**

**Figure S79.**

**Figure S80.**

**Figure S82.**

**Figure S81.**

**Figure S84.**

**Figure S83.**

**Figure S85.**

**Figure S86.**

**Figure S87.**

**Figure S88.**

**Figure S90.**

**Figure S89.**

**Figure S92.**

**Figure S91.**

**Figure S94.**

**Figure S93.**

**Figure S96.**

**Figure S95.**

**Figure S98.**

**Figure S97.**

**Figure S100.**

**Figure S99.**

**Figure S102.**

**Figure S101.**
